# Phosphine-catalyzed divergent domino processes between γ-substituted allenoates and carbonyl-activated alkenes†

**DOI:** 10.1039/d1sc06364b

**Published:** 2022-02-11

**Authors:** Mingyue Wu, Zhaobin Han, Huanzhen Ni, Nengzhong Wang, Kuiling Ding, Yixin Lu

**Affiliations:** Department of Chemistry, National University of Singapore 3 Science Drive 3 Singapore 117543 Singapore chmlyx@nus.edu.sg; State Key Laboratory of Organometallic Chemistry, Shanghai Institute of Organic Chemistry, Chinese Academy of Sciences 345 Lingling Road Shanghai 200032 China kding@mail.sioc.ac.cn

## Abstract

Highly enantioselective and chemodivergent domino reactions between γ-substituted allenoates and activated alkenes have been developed. In the presence of NUSIOC-Phos, triketone enone substrates smoothly reacted with γ-substituted allenoates to form bicyclic furofurans in good yields with high stereoselectivities. Alternatively, the reaction between diester-activated enone substrates and γ-substituted allenoates formed chiral conjugated 1,3-dienes in good yields with excellent enantioselectivities. Notably, by employing substrates with subtle structural difference, under virtually identical reaction conditions, we were able to access two types of chiral products, which are of biological relevance and synthetic importance.

## Abstract 2

Highly enantioselective and chemodivergent domino reactions between γ-substituted allenoates and activated alkenes have been developed.

## Graphical abstract

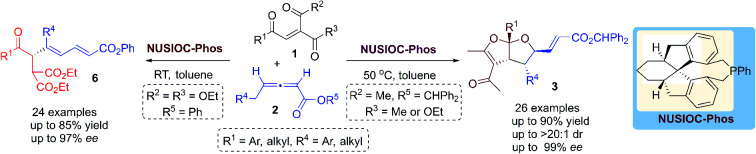

## Introduction

**Fig. 1:**
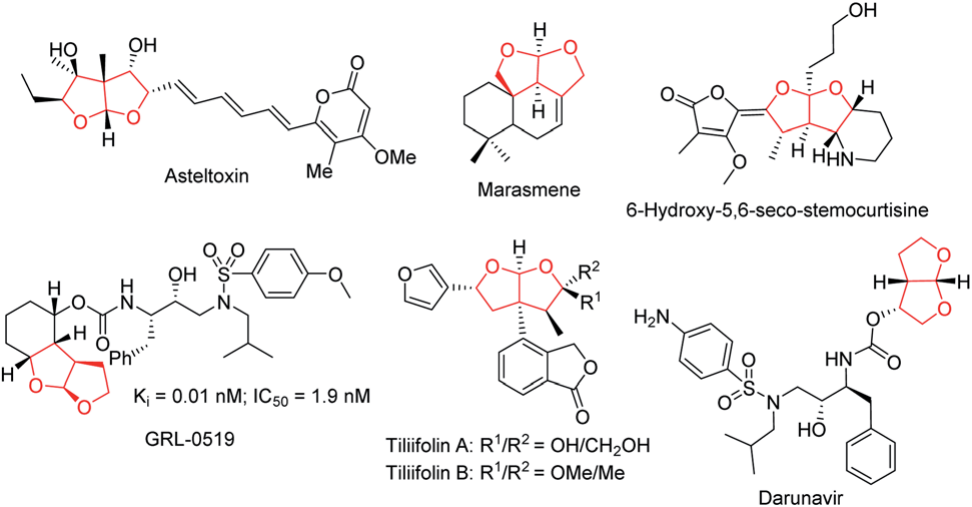
Natural products and bioactive molecules containing a fused bicyclic acetal.

Ever since Lu's seminal report^1^ on phosphine-catalyzed [3 + 2] annulation and Trost's disclosure^2^ of phosphine-mediated “Umpolung” addition in the mid 1990s, the past few decades have seen tremendous progress of asymmetric phosphine catalysis.^3^ Phosphine-mediated reactions have attracted attention from synthetic chemists and have been widely used for the creation of a broad range of molecular architectures.^4^ Phosphine-catalyzed annulation reactions are the most common reaction types, and various [3 + 2],^5^ [4 + 1],^6^ and [4 + 2]^7^ annulation reactions are found to be useful for building up five- or six-membered ring systems. Moreover, phosphine-catalyzed γ-additions,^8^ Michael additions,^9^ Morita–Baylis–Hillman (MBH) reactions,^10^ and Rauhut–Currier (RC) reactions^11^ have also been shown to be synthetically useful. In our continuous pursuit of phosphine catalysis, we are particularly interested in the effective asymmetric creation of chiral scaffolds of biological significance. Fused bicyclic chiral acetals are widely present in nature. Some representative examples are illustrated in Fig. 1, including asteltoxin,^12^ marasmene,^13^ 6-hydroxy-5,6-seco-stemocurtisine,^14^ GRL-0519,^15^ tiliifolin A/B,^16^ and darunavir.^17^ The biological activities of these compounds are often dependent on the substitution pattern of the chiral acetal moiety, thus the asymmetric synthesis of these molecules is highly desirable. While there are a number of reports on the synthesis of benzofused acetals,^18^ efficient catalytic asymmetric preparation of bicyclic furofurans was barely explored.^19^ We recently introduced a C2-symmetric chiral phosphine catalyst NUSIOC-Phos, which effectively promoted the formation of tricyclic γ-lactams *via* an enantioselective domino process.^20^ We therefore wondered whether a tandem reaction employing suitable substrates may be developed to access chiral bicyclic furofuran structural motifs.

**Fig. 2:**
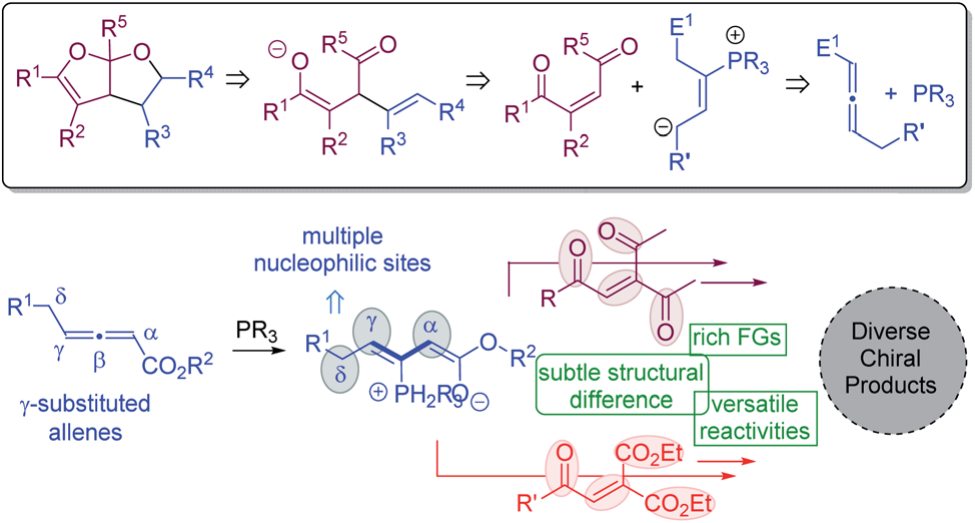
Our hypothesis.

The Paal–Knorr synthesis is a classic method for the preparation of furans from 1,4-diketones upon treatment with strong mineral acids.^21^ We envisioned that a modified Paal–Knorr synthesis may be utilized to construct bicyclic furofuran cores (Fig. 2). If the oxygen anion resulting from the initial enolate-induced ring closure is trapped by an intramolecular electrophilic reaction partner, then bicyclic furofurans can be created. To generate the enolate species for the furofuran formation, substrates bearing a double bond flanked by two carbonyl groups appear to be ideal. The zwitterionic intermediate derived from a phosphine catalyst and an allene substrate is anticipated to play two distinct roles: (1) to undergo a nucleophilic attack on the enone to form the key enolated species; (2) to electrophilically trap the oxygen anion to form bicyclic furofurans. Moreover, if γ-substituted allenes^22^ are employed, upon phosphine attack, an intermediate with three potential nucleophilic sites at the α, γ, and δ-positions is generated, which will add in great flexibility in creating molecular complexity. For the alkene substrates bearing 1,4-dicarbonyl groups, different carbonyl functional groups may be utilized, and such subtle structural differences may lead to different reactivities, making it feasible to create diverse chiral products. Herein, we document phosphine-catalyzed divergent domino reactions between γ-substituted allenoates and alkenes that are activated by carbonyl groups. Hinging on the nature of electron-withdrawing groups, the reaction proceeded through different pathways, and either fused bicyclic furofurans or 1,3-conjugate dienes were obtained in high yields with good to excellent stereoselectivities.

## Results and discussion

### Construction of bicyclic furofurans

We first evaluated the reaction between 3-acetyl-1-aryl-2-pentene-1,4-diones 1a and γ-substituted allenoate 2a in the presence of different phosphine catalysts (Table 1). Amino acid-based bifunctional phosphines bearing different hydrogen bond donors (5a–5d) failed to yield the desired product (entries 1–4). While other mono-functional phosphines (5e–5h) at most led to the formation of the desired products in low yields (entries 5–8), we were delighted to discover that our earlier developed^23^ NUSIOC-Phos (5j) was a good catalyst; bicyclic furofuran 3a was obtained in moderate yield with good diastereoselectivity and excellent enantioselectivity (entry 10). When the reaction was performed at 50 °C, the chemical yield was substantially improved, with virtually maintained stereoselectivities (entry 11). Different solvents were also screened (entries 12–17), and toluene remained to the solvent of choice.

**Table 1:** Reaction screening^a^

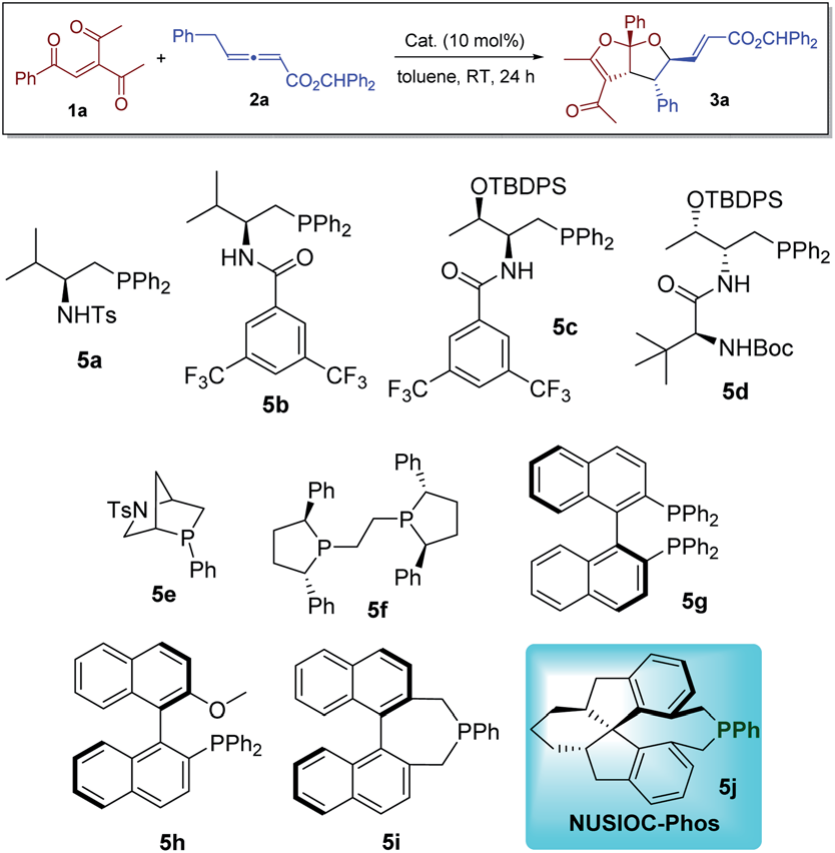					
Entry	Cat.	Solvent	dr^b^	Yield^c^ [%]	ee^d^ [%]
1	5a	Toluene	—	Trace	—
2	5b	Toluene	—	Trace	—
3	5c	Toluene	—	Trace	—
4	5d	Toluene	—	Trace	—
5	5e	Toluene	—	Trace	—
6	5f	Toluene	1 : 1	10	−21
7	5g	Toluene	1.8 : 1	41	13
8	5h	Toluene	1.6 : 1	38	−20
9	5i	Toluene	—	Trace	—
10	5j	Toluene	5.7 : 1	65	99
11^e^	5j	Toluene	5.4 : 1	82	99
12^e^	5j	CH_2_Cl_2_	3.7 : 1	75	99
13^e^	5j	EtOAc	4.3 : 1	72	98
14^e^	5j	CHCl_3_	3.7 : 1	74	99
15^e^	5j	1,4-Dioxane	5.3 : 1	73	97
16^e^	5j	THF	3.0 : 1	27	97
17^e^	5j	MTBE	5.0 : 1	71	98

a Reactions were performed with 1a (0.1 mmol), 2a (0.15 mmol), and the catalyst (0.01 mmol) in the solvent specified (1.0 mL) at room temperature for 24 h.

b Determined by crude ^1^H NMR analysis.

c Isolated yield of the major isomer.

d Determined by HPLC analysis on a chiral stationary phase.

e The reaction was performed at 50 °C for 48 h.

**Scheme 1:**
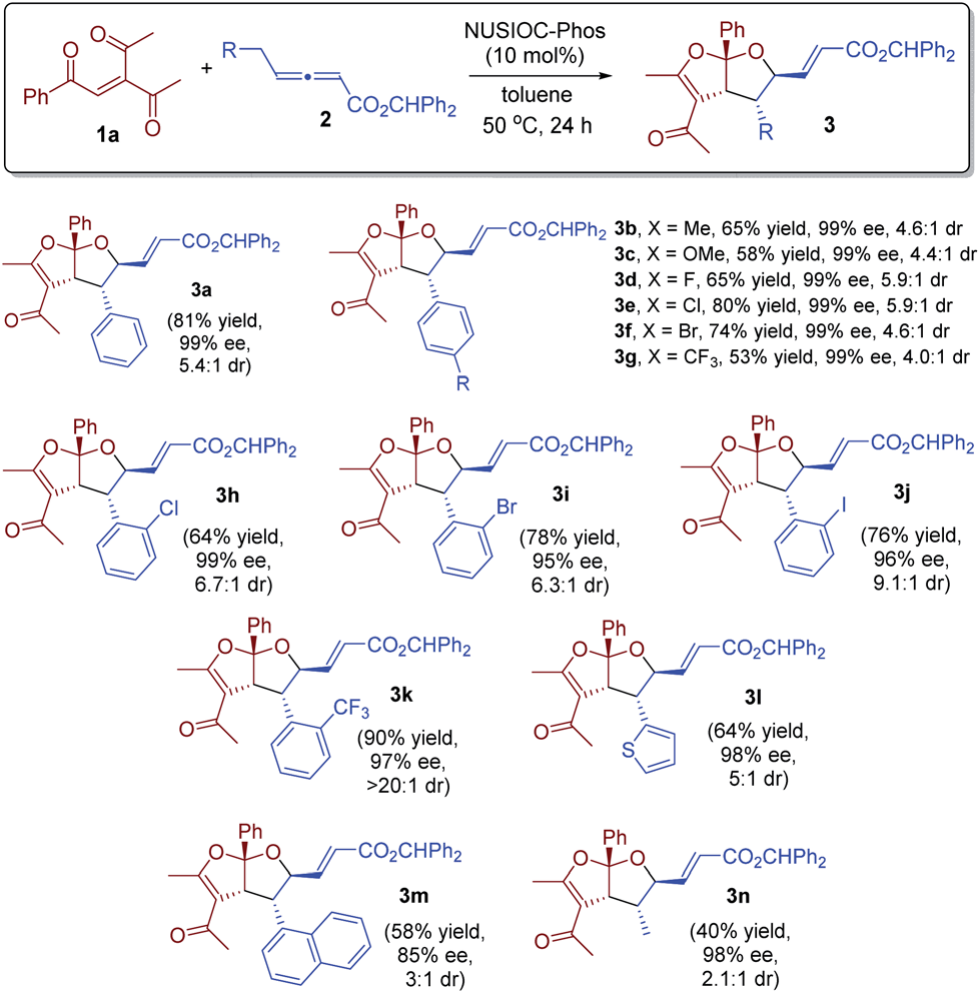
Reaction scope. Conditions: 1a (0.1 mmol), 2 (0.15 mmol), and NUSIOC-Phos (0.01 mmol) in toluene (1.0 mL) at 50 °C for 24 hours. The dr values were determined by crude ^1^H NMR analysis. Yields given are the isolated yields of the main isomer. The ee values were determined by HPLC analysis on a chiral stationary phase. For 3g–3k, 20% catalyst and 40% 4-methoxyphenol were used. For 3n, 20% catalyst was employed.

With the optimized reaction conditions in hand, we investigated the scope of this domino process by employing different γ-substituted allenoates 2, and the results are summarized in Scheme 1. γ-Benzyl-substituted allenoates bearing a *para*-substituent on the phenyl are well-tolerated, and the desired products were obtained in high yields with excellent enantioselectivities and good diastereoselectivities (65% to 81% yields, 99% ee, up to >5.9 : 1 dr, 3a–3g). The allenoates containing an *ortho*-substituted phenyl proved to be excellent substrates; excellent ee values and higher dr ratios were attainable (3h–3j). In particular, when an *ortho*-CF_3_-phenyl containing γ-benzylic allenoate was employed, only one diastereoisomer was observed (3k). Allenoates bearing a thiophene (3l) or a naphthyl substituent (3m) were also found to be suitable substrates, although the diastereoselectivity was slightly lower for the latter. Moreover, the substituent of γ-allenoates could also be aliphatic, and the desired acetal was obtained in 40% yield with 98% ee and 2:1 dr (3n). The absolute configurations of the fused bicyclic furofurans were assigned on the basis of X-ray crystallographic analysis of 3d.^24^

**Scheme 2:**
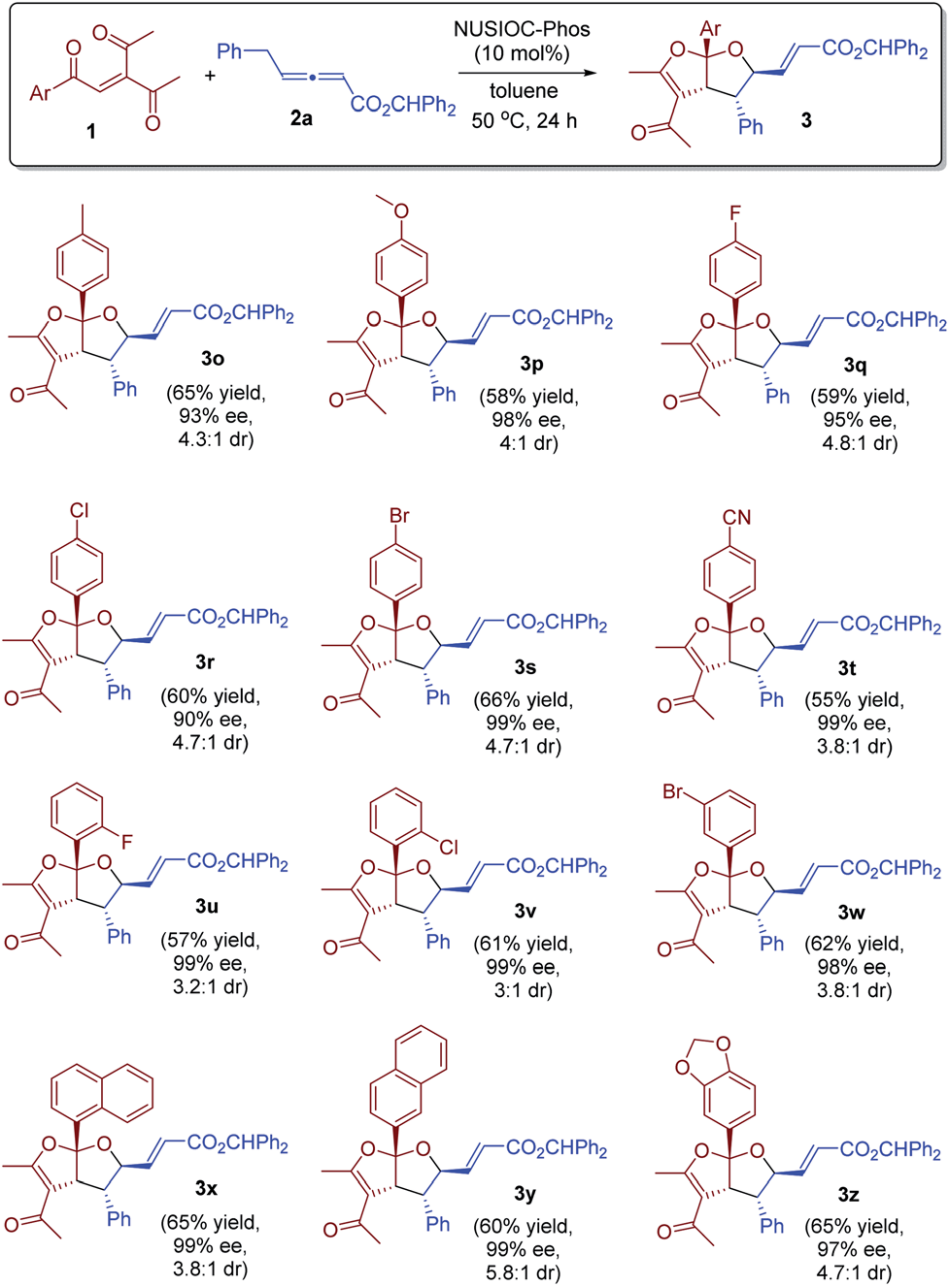
Further scope exploration. Conditions: 1 (0.1 mmol), 2a (0.15 mmol), 4-methoxyphenol (0.02 mmol), and NUSIOC-Phos (0.02 mmol) in toluene (1.0 mL) at 50 °C for 24 hours. The dr values were determined by crude ^1^H NMR analysis. Yields given are the isolated yields of the main isomer. The ee values were determined by HPLC analysis on a chiral stationary phase.

We further examined the reaction scope by utilizing a range of enone substrates 1 bearing different substituents (Scheme 2). In general, the electronic nature and substitution pattern of different phenyl groups had little influence on the reaction, and the desired bicyclic furofurans were obtained in reasonable yields, with excellent enantioselectivities and good diastereoselectivities (3o–3w). Moreover, enones containing 1-naphthyl, 2-naphthyl, or 5-piperonyl groups were all suitable substrates, and the desired products were derived in satisfactory yields and with high stereoselectivities (3x–3z).

### Synthesis of conjugated 1,3-dienes

From the synthetic viewpoint, divergent pathways from the same/similar substrates, under the catalysis of similar/same catalysts, would be quite ideal. In this context, we have keen interest in developing divergent asymmetric synthetic approaches.^25^ At the outset, we wanted to examine the domino reaction of γ-substituted allenoates with other carbonyl-activated alkenes. Such structural variation is mechanistically interesting; for instance, the ester-derived enolate will unlikely undergo intramolecular cyclization with another ester moiety and thus offer opportunities for deriving different products.

We examined this domino process by employing other types of activated alkenes, under the optimal conditions that we have established for the synthesis of bicyclic furofurans. When one ketone moiety was replaced by an ester group, both 1a′-Z and 1a′-E were found to be suitable for the reaction, forming acetal product 3a′ in slightly decreased yield with similar stereoselectivities (eqn (1)). However, if one ketone moiety was deleted (1a′′), the corresponding bicyclic furofuran was not formed (eqn (2)). Interestingly, when enone 4a bearing two ester groups was treated with allene 2a in the presence of NUSIOC-Phos, conjugated 1,3-diene 6a was obtained in 90% yield and with 71% ee (eqn (3)). Apparently, a different mechanistic pathway is in operation, due to the structural and electronic differences of the activating groups in the enones. The conjugated 1,3-dienes are useful structural motifs that are often found in natural products and bioactive molecules,^26^ and they are also of special importance in polymer chemistry and materials science.^27^ Synthetically, 1,3-dienes are extremely valuable, and thus intensive efforts have been devoted to their efficient synthesis.^28^ We were then wondering whether we could establish an efficient asymmetric synthetic approach to access chiral building blocks containing conjugated 1,3-dienes.1
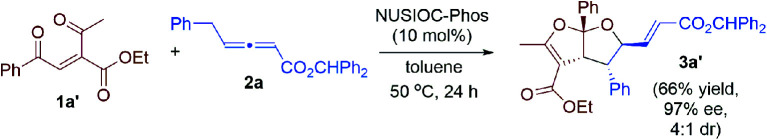
2
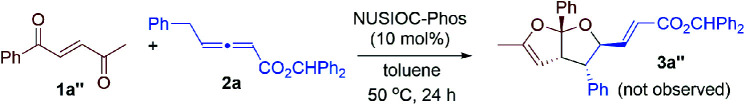
3
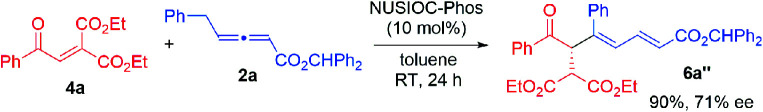


We examined the reaction between diester-activated alkene 4b and allenoates 2′, under the catalysis of NUSIOC-Phos, and the results are summarized in Table 2. As we anticipated that certain proton transfer processes are likely to be involved during the formation of 1,3-diene products (*vide infra* for the proposed reaction mechanism), we thus evaluated the influence of adding a number of proton donors to the reaction system. The addition of phenol led to a substantial increase in enantioselectivity (entry 2). While *p*-methoxylphenol and benzoic acid were both effective, *o*-methoxylphenol appeared to be slightly better (entries 3–5). A quick solvent screening confirmed that toluene was most ideal (entries 6–9). We next explored allenoates with different ester moieties and discovered that the employment of phenyl allenoate led to best enantioselectivity (entries 10–13). When the reaction was performed at lower temperature under the above optimal conditions, the desired 1,3-diene 5 was obtained in 80% yield with 93% ee (entry 14).

**Table 2:** Optimization for the δ-carbon Michael addition/isomerization reaction^a^

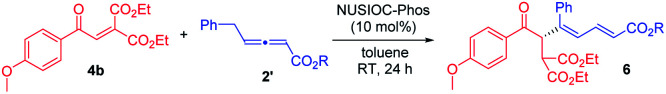					
Entry	Solvent	Additive	R	Yield^b^ [%]	ee^c^ [%]
1	Toluene	No	CHPh_2_	90	71
2	Toluene	Phenol	CHPh_2_	86	80
3	Toluene	*p*-OMePhOH	CHPh_2_	87	81
4	Toluene	*o*-OMePhOH	CHPh_2_	87	83
5	Toluene	PhCO_2_H	CHPh_2_	73	79
6	Mesitylene	*o*-OMePhOH	CHPh_2_	88	82
7	MTBE	*o*-OMePhOH	CHPh_2_	71	81
8	PhCl	*o*-OMePhOH	CHPh_2_	86	80
9	Dioxane	*o*-OMePhOH	CHPh_2_	82	82
10	Toluene	*o*-OMePhOH	Me	86	87
11	Toluene	*o*-OMePhOH	Ph	82	88
12	Toluene	*o*-OMePhOH	Bn	83	85
13	Toluene	*o*-OMePhOH	^*t*^Bu	76	77
14^d^	**Toluene**	***o*-OMePhOH**	**Ph**	**80**	**93**

a Reactions were performed with 4b (0.1 mmol), 2′ (0.15 mmol), the additive (0.05 mmol) and NUSIOC-Phos (0.01 mmol) in the solvent specified (1.0 mL) at 25 °C for 24 h.

b Isolated yield.

c Determined by HPLC analysis on a chiral stationary phase.

d The reaction mixture was first stirred at 0 °C for 6 h and then at 25 °C for 24 h.

**Scheme 3:**
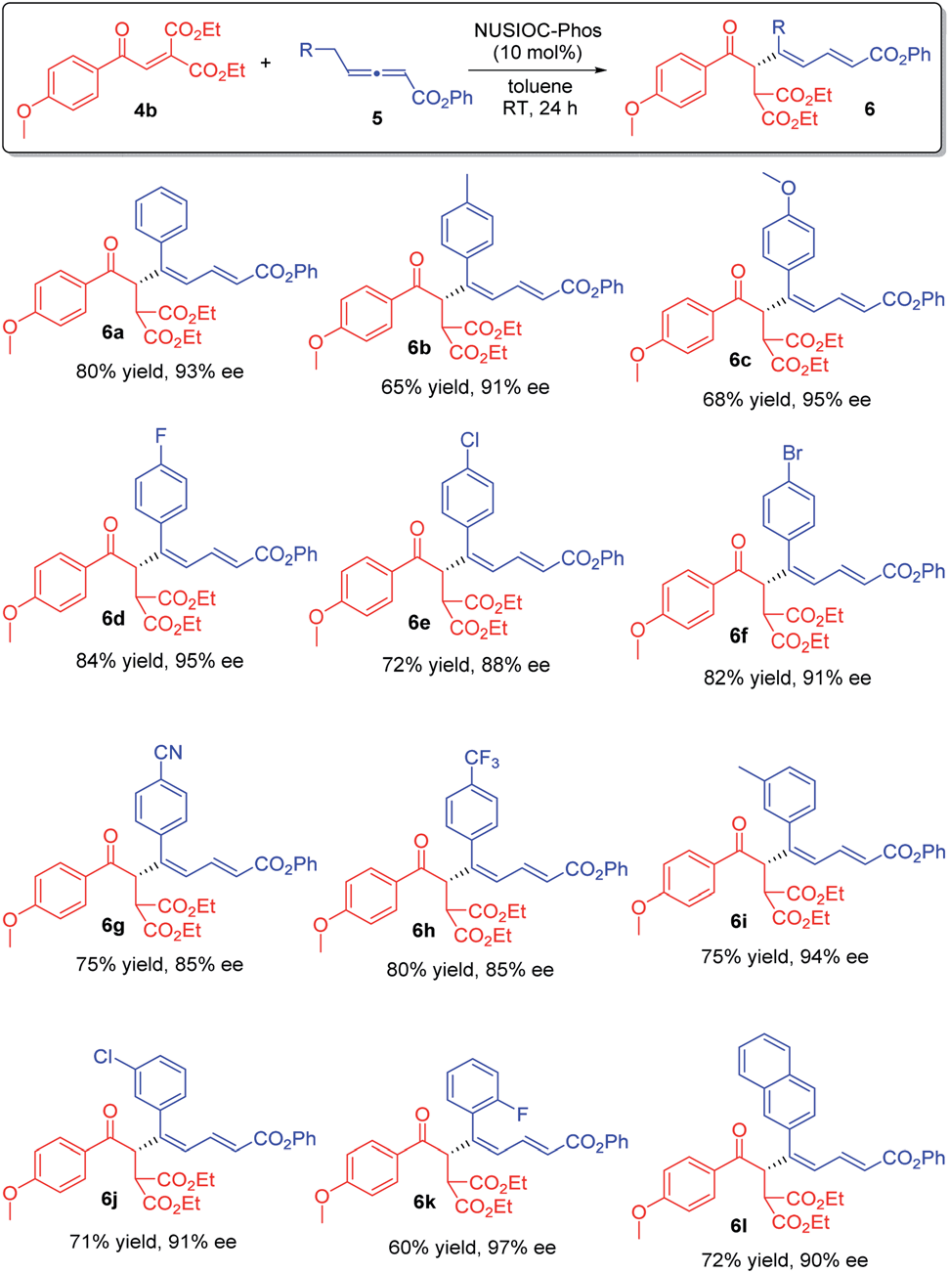
Synthesis of chiral 1,3-dienes: reaction scope. Conditions: 4b (0.1 mmol), 5 (0.15 mmol), 2-methoxyphenol (0.05 mmol) and NUSIOC-Phos (0.01 mmol) in toluene (1.0 mL) at 0 °C for 6 h, and subsequently at 25 °C for 24 h; yields given were isolated yields; the ee values were determined by HPLC analysis on a chiral stationary phase.

We subsequently applied the optimum conditions to establish the reaction scope (Scheme 3). In general, different benzylic types of γ-substituted allenoates 5 could be utilized, and regardless of the electronic nature and substitution patterns of the aryl moieties, conjugated 1,3-dienes 6 were obtained in decent yields and with excellent enantioselectivities (6a–6l).

**Scheme 4:**
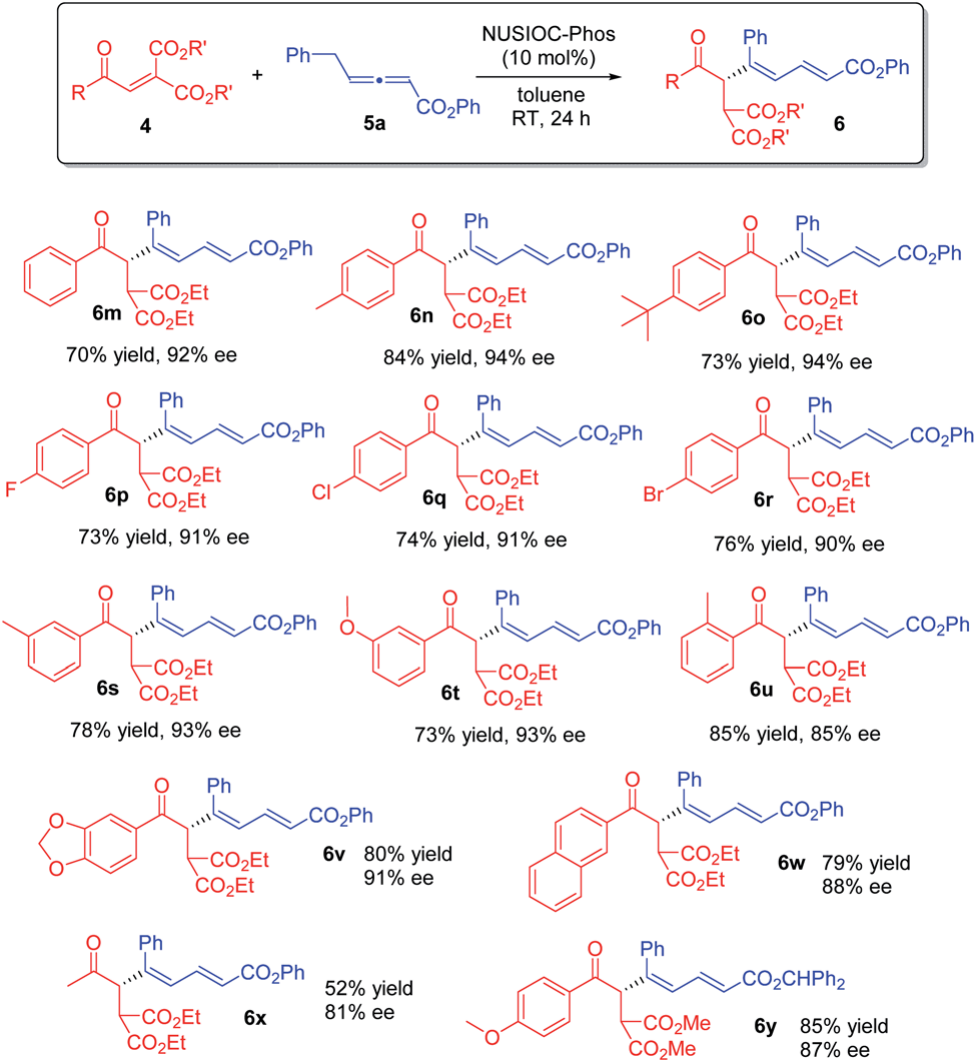
Further scope. Conditions: 4 (0.1 mmol), 5a (0.15 mmol), 2-methoxyphenol (0.04 mmol), and NUSIOC-Phos (0.01 mmol) in toluene (1.0 mL) at 0 °C for 6 h, and subsequently at 25 °C for 24 h; yields given were isolated yields; the ee values were determined by HPLC analysis on a chiral stationary phase.

Furthermore, we examined the reaction scope by employing different diester enones 4 (Scheme 4). Enones containing an electron donating group on the phenyl ring with different substitution patterns were well-tolerated (6n, 6o, and 6s–6u), and high enantioselectivities were obtained. Similarly, aryl enones bearing different halogen atoms were also found to be suitable (6p–6r). Moreover, the reaction was applicable to 5-piperonyl and 2-naphthyl enone substrates, and the desired 1,3-dienes were obtained in good yields with high enantioselectivities (6v & 6w). Finally, when an aliphatic enone was employed, the reaction proceeded smoothly, affording product 6x in moderate yield with good enantioselectivity. The absolute configuration of 1,3-dienes 6 was assigned on the basis of X-ray crystallographic analysis of 6y (see the ESI† for more details).

### Proposed reaction mechanisms

**Fig. 3:**
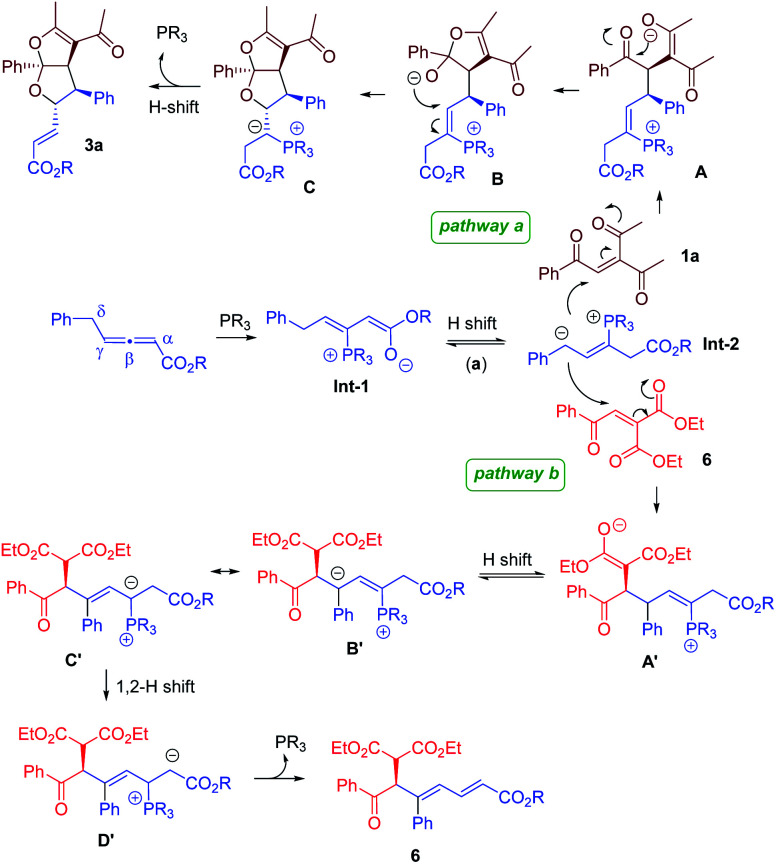
Proposed mechanism.

Plausible mechanistic pathways are proposed in Fig. 3. Phosphine attack on the γ-substituted allenoate generates zwitterionic intermediate Int-1, which is nucleophilic at either the α- or the γ- position. Subsequently, a proton shift leads to the formation of δ-anionic Int-2. When the triketone enone 1a is used, the conjugate addition of Int-2 to 1a forms enolate A, which attacks the ketone function intramolecularly to yield B. A second intramolecular addition then takes place to afford the bicyclic acetal core structure C. Another proton shift, followed by the elimination and re-generation of phosphine, forms the bicyclic furofuran product 3a (pathway a). With the employment of diester-containing enone 6, the reaction proceeds through different mechanistic steps. The conjugate addition of Int-2 to 6 creates intermediate A′, which undergoes a proton transfer to form B′. Finally, a 1,2-proton shift, followed by re-generation of the phosphine catalyst, furnishes chiral 1,3-diene 6 (pathway b).4
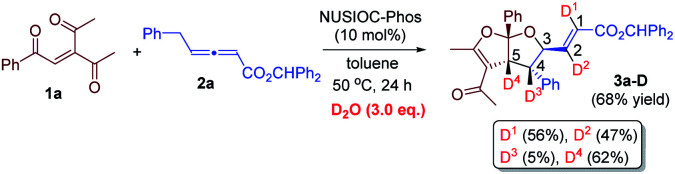
5
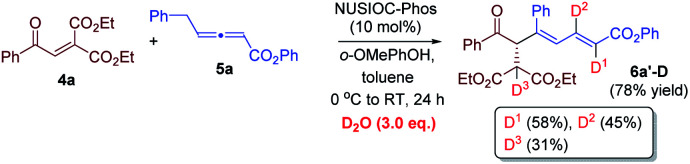
6
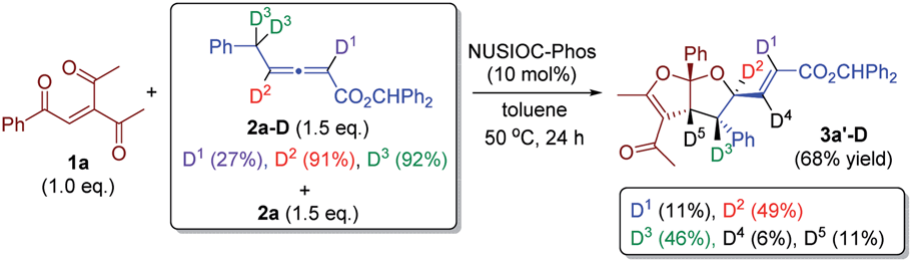
7
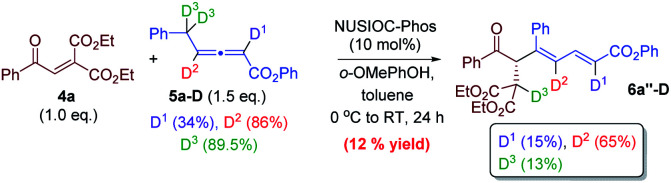


We have performed a few deuterium-labelling experiments to further probe our proposed reaction mechanisms. When bicyclic acetal 3a was synthesized in the presence of D_2_O and under otherwise identical reaction conditions, deuterated product 3a-D with multiple deuterium incorporations was obtained (eqn (4)). Notably, 62% deuterium incorporation at the C5 position of 3a-D was observed, which is likely due to the facile tautomerization of ketone intermediate A to its enol form. When the reaction leading to the formation of conjugated 1,3-dienes was performed in the presence of D_2_O, similar deuterium incorporations into 6a′-D were observed (eqn (5)). Notably, the sites where the deuterium atoms were incorporated into the products are at the proposed positions whereby the proton transfers take place, consistent with our proposed mechanistic pathways. When enone 1a was reacted with equal amounts of allenoate 2a and deuterated 2a-D, around half deuterium incorporation in bicyclic acetal 3a′-D was observed (eqn (6)), suggesting that both deuterated and non-deuterated allenes have similar reaction rates. For the 1,3-diene forming reaction, if a deuterated allenoate (5a-D) was employed under otherwise the same reaction conditions, product 6a′′-D with much less deuterium incorporation was obtained in only 12% yield (eqn (7)), and this is in stark contrast to a similar reaction of employing non-deuterated 5a, whereby 78% of 6a′-D was attainable (eqn (5)).^29^ The kinetic isotope effects observed in the above experiment clearly suggest that the rate-determining step for the conjugated 1,3-diene formation involves the proton transfer process.

## Conclusions

In summary, we have developed phosphine-catalyzed divergent domino processes between γ-substituted allenoates and carbonyl-activated alkenes. When triketone enone substrates were reacted with allenoates in the presence of NUSIOC-Phos, a domino reaction process led to the formation of diastereoselective bicyclic furofurans in decent yields with excellent enantioselectivities. If enone substrates bearing two ester groups are utilized under similar reaction conditions, a different cascade sequence was in operation, forming chiral conjugated 1,3-dienes in high yields with excellent enantioselectivities. Notably, through careful design and precise control of catalytic processes, by employing substrates with subtle structural differences, we were able to develop highly efficient asymmetric synthetic methodologies that allow for quick access to important chiral molecular architectures. We are currently applying the synthetic methodologies disclosed herein to the synthesis of molecules of biological significance and shall report our findings in due course.

## Data availability

All experimental procedures, characterization, copies of NMR spectra for all new compounds related to this article can be found in the ESI.†

## Author contributions

M. W. discovered and performed most of the experiments and wrote the first draft of the manuscript. Z. H. and H. N contributed to some experiments (synthesis of some NUSIOC-Phos intermediates). N. W. analyzed the 2-D NMR spectrum and discussed the results. K. D. and Y. L. supervised the work, finalized the manuscript, and coordinated the overall project.

## Conflicts of interest

There are no conflicts to declare.

## Supplementary Material

SC-013-D1SC06364B-s001

SC-013-D1SC06364B-s002
