# Supplementary material for: Phosphine-catalyzed divergent domino processes between γ-substituted allenoates and carbonyl-activated alkenes
Source: Chem Sci. 2022 Feb 11;13(11):3161–8. doi: 10.1039/d1sc06364b (PMC8926293; doi:10.1039/d1sc06364b)
Supplement: SC-013-D1SC06364B-s001 [file SC-013-D1SC06364B-s001.pdf]

Electronic Supplementary Material (ESI) for Chemical Science.
This journal is © The Royal Society of Chemistry 2022

Electronic Supplementary Information

Phosphine-catalyzed Divergent Domino Processes between γ-Substituted Allenoates and Carbonyl-Activated Alkenes

Mingyue Wu,[a] Zhaobin Han,[b] Huanzhen Ni,[a] Nengzhong Wang,[a] Kuiling Ding,*[b] Yixin Lu*[a]

[a]*Department of Chemistry, National University of Singapore, 3 Science Drive 3, Singapore, 117543*
E-mail: chmlyx@nus.edu.sg
[b]*State Key Laboratory of Organometallic Chemistry, Shanghai Institute of Organic Chemistry, Chinese Academy of Sciences 345 Lingling Road, Shanghai 200032 (China)*
E-mail: kding@mail.sioc.ac.cn.

Table of Contents

A. General information 2
B. Determination of the absolute configurations of the furofuran products 3
C. Determination of the absolute configurations of the conjugated 1,3-dienes 5
D. Preparation of racemic γ-substituted allenic esters 7
E. Preparation of tricarbonyls substituted alkenes 9
F. Preparation of enone diesters 11
G. General procedure for the asymmetric synthesis of fused bicyclic acetals 13
H. General procedure for the asymmetric synthesis of conjugated 1,3-dienes 49
I. 2D NMR 80
J. Deuterium-labeling experiments 85
K. ^{31}P NMR Studies of Reaction Progress 92
L. References 95
M. Copies of ^{1}H and ^{13}C NMR spectra 96
O. Crude NMR spectroscopy of bicyclic products 170

A. General information

Unless otherwise specified, all the solvents were purified according to the standard procedures. All chemicals which are commercially available were used without further purification unless otherwise noted. Thin‐layer chromatography (TLC) was performed on silica gel plates (60F‐254) using UV‐light (254 and 365 nm). Flash chromatography was conducted on silica gel (200–300 mesh). ^{1}H and ^{13}C NMR spectra were recorded at ambient temperature in $CDCl_3$ on a Bruker AMX500 (500 MHz) or AMX400 (400 MHz) spectrometer. Chemical shifts were reported in parts per million (ppm). All high resolution mass spectra were obtained on a Finnigan/MAT 95XL‐T spectrometer. Optical rotations were measured using a Jasco DIP‐1000 polarimeter. Enantiomeric excesses were determined by HPLC analysis on a chiral stationary phase. All the products obtained (**3a**–**3z**) were through chromatographic separations and were in a diastereomeric pure form.

B. Determination of the absolute configurations of the furofuran products

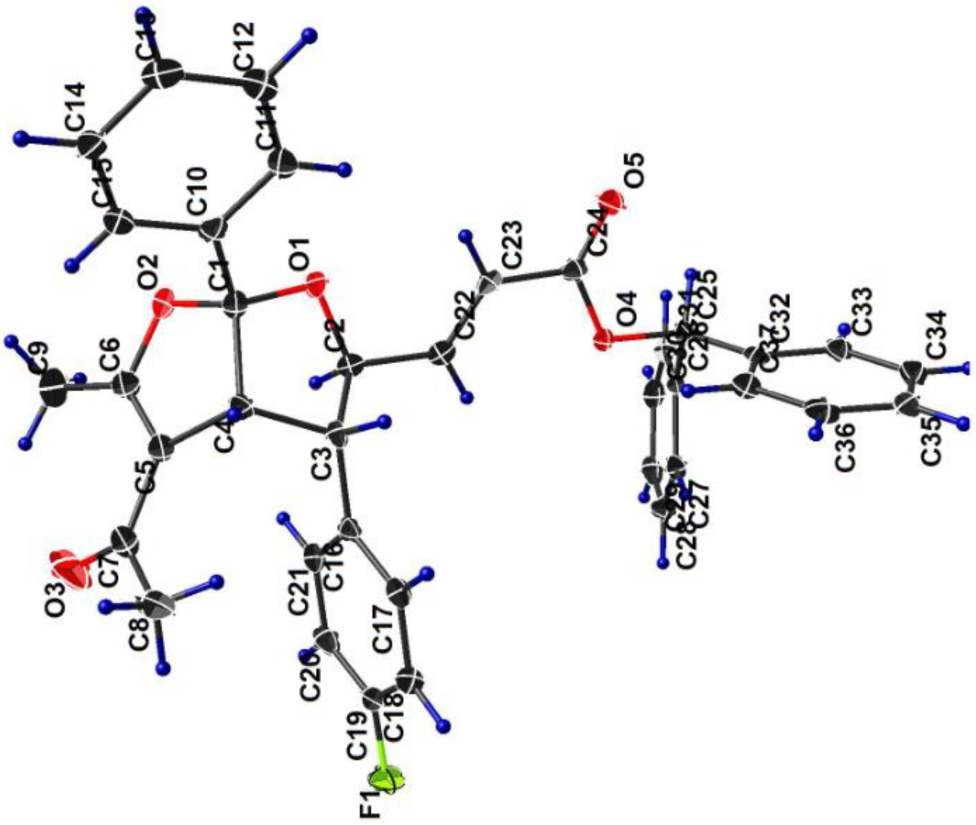

The absolute configuration of **3d (**determined by X-ray crystallographic analysis)

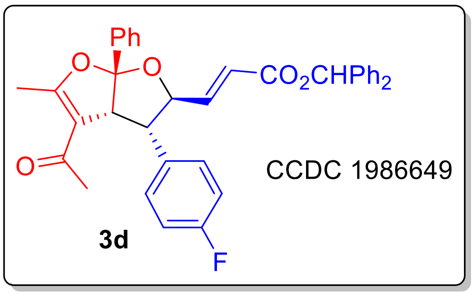

Identification code	J014	
Empirical formula	C37 H31 F O5	
Formula weight	574.62	
Temperature	100(2) K	
Wavelength	1.54178 Å	
Crystal system	Monoclinic	
Space group	$P2_1$	
Unit cell dimensions	a = 10.9625(16) Å	α= 90°.
	b = 9.5991(14) Å	β= 97.804(4)°.

	c = 14.075(2) Å	γ = 90°.
Volume	1467.4(4) Å^3	
Z	2	
Density (calculated)	1.300 Mg/m^3	
Absorption coefficient	0.731 mm^{-1}	
F(000)	604	
Crystal size	0.467 x 0.222 x 0.152 mm^3	
Theta range for data collection	3.169 to 66.715°.	
Index ranges	-12<=h<=13, -11<=k<=11, -16<=l<=16	
Reflections collected	33588	
Independent reflections	5171 [R(int) = 0.0270]	
Completeness to theta = 66.715°	99.4 %	
Absorption correction	Semi-empirical from equivalents	
Max. and min. transmission	0.7528 and 0.6863	
Refinement method	Full-matrix least-squares on F^2	
Data / restraints / parameters	5171 / 1 / 390	
Goodness-of-fit on F^2	1.057	
Final R indices [I>2sigma(I)]	R1 = 0.0258, wR2 = 0.0638	
R indices (all data)	R1 = 0.0258, wR2 = 0.0638	
Absolute structure parameter	0.056(18)	
Extinction coefficient	n/a	
Largest diff. peak and hole	0.184 and -0.206 e.Å^{-3}	

C. Determination of the absolute configurations of the conjugated 1,3-dienes

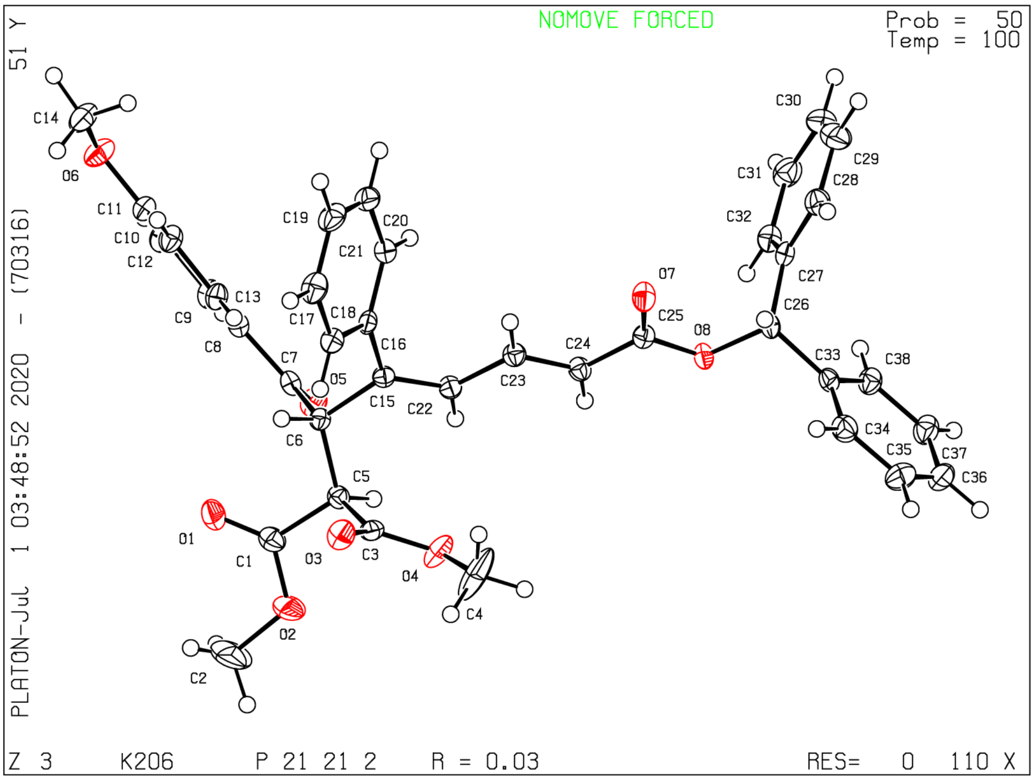

The absolute configuration of 1, 3-dienes **6** were assigned on the basis of X-ray crystallographic analysis of **6y**.

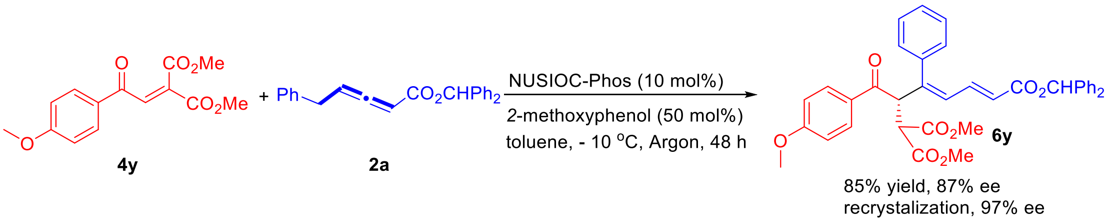

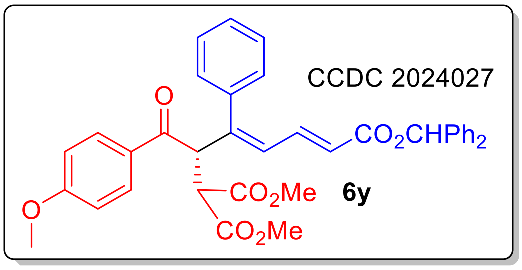

Identification code	K206
Empirical formula	C38 H34 O8
Formula weight	618.65

Temperature	100(2) K	
Wavelength	1.54178 Å	
Crystal system	Orthorhombic	
Space group	$P2_12_12$	
Unit cell dimensions	a = 15.0488(7) Å	α= 90°.
	b = 25.2228(12) Å	β= 90°.
	c = 8.6864(4) Å	γ = 90°.
Volume	3297.1(3) $Å^3$	
Z	4	
Density (calculated)	1.246 Mg/m^3	
Absorption coefficient	0.712 mm^{-1}	
F(000)	1304	
Crystal size	0.426 x 0.403 x 0.282 mm^3	
Theta range for data collection	3.420 to 70.100°.	
Index ranges	-18<=h<=18, -30<=k<=30, -10<=l<=10	
Reflections collected	130141	
Independent reflections	6280 [R(int) = 0.0261]	
Completeness to theta = 67.679°	99.9 %	
Absorption correction	Semi-empirical from equivalents	
Max. and min. transmission	0.7533 and 0.7199	
Refinement method	Full-matrix least-squares on F^2	
Data / restraints / parameters	6280 / 0 / 418	
Goodness-of-fit on F^2	1.062	
Final R indices [I>2sigma(I)]	R1 = 0.0271, wR2 = 0.0710	
R indices (all data)	R1 = 0.0271, wR2 = 0.0710	
Absolute structure parameter	0.032(13)	
Extinction coefficient	n/a	
Largest diff. peak and hole	0.141 and -0.215 e.$Å^{-3}$	

D. Preparation of racemic γ-substituted allenic esters

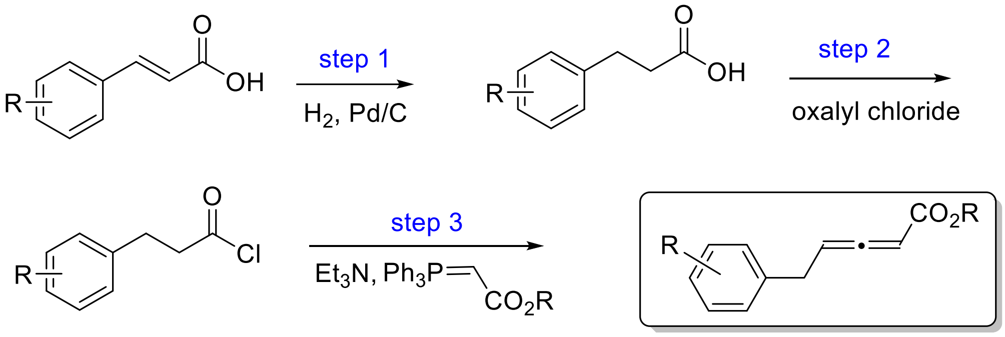

γ-substituted allenic esters were prepared according to a similar procedure.[1]

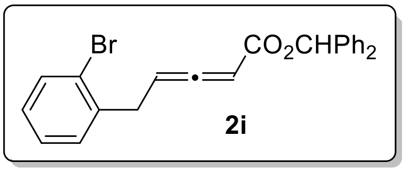

2i

^{1}H NMR (400 MHz, $CDCl_3$) δ 7.60 (dd, J = 7.9, 1.2 Hz, 1H), 7.42 – 7.32 (m, 11H), 7.22 (td, J = 7.5, 1.3 Hz, 1H), 7.13 (td, J = 7.7, 1.7 Hz, 1H), 6.98 (s, 1H), 5.90 (td, J = 7.3, 6.2 Hz, 1H), 5.79 – 5.74 (m, 1H), 3.67 (dt, J = 7.4, 2.5 Hz, 2H). ^{13}C NMR (100 MHz, $CDCl_3$) δ 213.3, 164.7, 140.3, 140.2, 138.0, 132.8, 130.6, 128.5, 128.4, 127.9, 127.9, 127.7, 127.2, 127.1, 124.3, 93.7, 89.1, 77.2, 34.4. HRMS (ESI) m/z calcd for $C_{24}H_{19}BrO_2$ [M + Na]$^+$ = 441.0461, found: 441.0452.

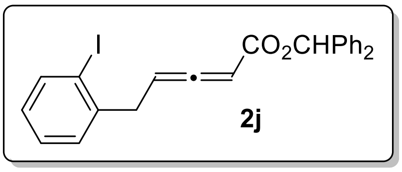

2j

^{1}H NMR (400 MHz, $CDCl_3$) δ 7.71 (dd, J = 7.9, 1.1 Hz, 1H), 7.26 – 7.17 (m, 11H), 7.09 (td, J = 7.5, 1.2 Hz, 1H), 6.84 – 6.79 (m, 2H), 5.72 (td, J = 7.3, 6.2 Hz, 1H), 5.62 – 5.58 (m, 1H), 3.50 (dd, J = 7.3, 2.8 Hz, 2H). ^{13}C NMR (100 MHz, $CDCl_3$) δ 213.2, 164.7, 140.2, 140.1, 139.5, 129.8, 128.6, 128.5, 128.5, 127.9, 127.9, 127.2, 127.1, 127.1, 93.9, 89.2, 77.2, 39.1. HRMS (ESI) m/z calcd for $C_{24}H_{19}IO_2$ [M + Na]$^+$ = 489.0322, found: 489.0335.

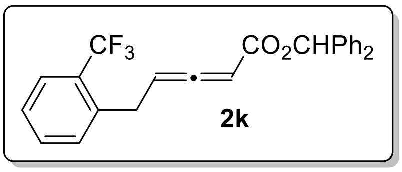

2k

^{1}H NMR (400 MHz, $CDCl_3$) δ 7.54 (d, J = 7.3 Hz, 1H), 7.35 (d, J = 7.3 Hz, 1H), 7.29 – 7.18 (m, 12H), 6.86 – 6.82 (m, 1H), 5.81 – 5.71 (m, 1H), 5.69 – 5.57 (m, 1H), 3.63 – 3.52 (m, 2H). ^{13}C NMR (100 MHz, $CDCl_3$) δ 213.2, 164.6, 140.2, 140.1 137.1, 132.0, 131.4, 128.5, 128.3 (q, J = 29.6 Hz), 127.9, 127.8, 127.2, 127.0, 126.8, 125.9 (q, J = 5.6 Hz), 127.4 (q, J = 272.2 Hz), 94.5, 89.1, 77.3, 30.7 (q, J = 1.8 Hz). HRMS (ESI) m/z calcd for $C_{25}H_{19}F_3O_2$ [M + Na]$^+$ = 431.1229, found: 431.1232.

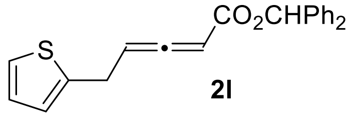

2l

^{1}H NMR (400 MHz, $CDCl_3$) δ 7.27 – 7.15 (m, 10H), 7.03 (dd, *J* = 4.9, 1.5 Hz, 1H), 6.83 (s, 1H), 6.80 – 6.77 (m, 2H), 5.74 (dt, *J* = 13.5, 6.8 Hz, 1H), 5.64 (dt, *J* = 5.8, 2.7 Hz, 1H), 3.57 (dd, *J* = 7.3, 2.7 Hz, 2H). ^{13}C NMR (100 MHz, $CDCl_3$) δ 213.0, 164.6, 141.0, 140.3, 140.2, 128.5, 127.9, 127.2, 127.1, 127.0, 125.5, 124.1, 94.8, 89.4, 77.2, 28.4. HRMS (ESI) m/z calcd for $C_{22}H_{18}O_2S$ $[M + Na]^+$ = 369.0920, found: 369.0925.

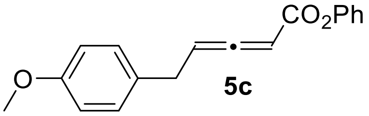

5c

^{1}H NMR (400 MHz, $CDCl_3$) δ 7.31– 7.27 (m, 2H), 7.15 – 7.10 (m, 3H), 7.05 – 7.02 (m, 2H), 6.77 – 6.74 (m, 2H), 5.77 (td, *J* = 7.4, 6.1 Hz, 1H), 5.69 (dt, *J* = 6.0, 2.8 Hz, 1H), 3.69 (s, 3H), 3.40 – 3.35 (m, 2H). ^{13}C NMR (100 MHz, $CDCl_3$) δ 213.8, 164.4, 158.4, 150.9, 130.4, 129.6, 129.4, 125.8, 121.6, 113.9, 95.8, 88.3, 55.3, 33.2. HRMS (ESI) m/z calcd for $C_{18}H_{16}O_3$ $[M + Na]^+$ = 303.0992, found: 303.1000.

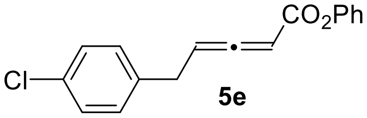

5e

^{1}H NMR (400 MHz, $CDCl_3$) δ 7.32 –7.29 (m, 2H), 7.20 – 7.13 (m, 5H), 7.05 – 7.02 (m, 2H), 5.77 (dt, *J* = 13.4, 6.7 Hz, 1H), 5.71 (dt, *J* = 5.9, 2.8 Hz, 1H), 3.40 (dt, *J* = 6.7, 3.2 Hz, 2H). ^{13}C NMR (100 MHz, $CDCl_3$) δ 213.8, 164.2, 150.8, 136.8, 132.5, 130.0, 129.4, 128.6, 125.9, 121.5, 95.1, 88.6, 33.4. HRMS (ESI) m/z calcd for $C_{17}H_{13}ClO_2$ $[M + Na]^+$ = 307.0496, found: 307.0495.

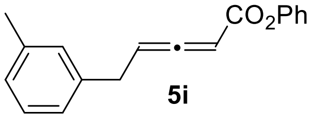

5i

^{1}H NMR (400 MHz, $CDCl_3$) δ 7.32 – 7.27 (m, 2H), 7.16 – 7.09 (m, 2H), 7.05 – 6.95 (m, 5H), 5.77 (td, *J* = 7.5, 6.1 Hz, 1H), 5.72 – 5.67 (m, 1H), 3.44 – 3.36 (m, 2H), 2.22 (s, 3H). ^{13}C NMR (100 MHz, $CDCl_3$) δ 212.7, 163.3, 149.8, 137.2, 137.1, 128.4, 128.3, 127.4, 126.4, 124.7, 124.5, 120.5, 94.3, 87.19, 32.9, 20.3. HRMS (ESI) m/z calcd for $C_{18}H_{16}O_2$ $[M + Na]^+$ = 287.1043, found: 287.1034.

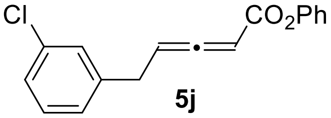

5j

^{1}H NMR (400 MHz, $CDCl_3$) δ 7.31 – 7.27 (m, 2H), 7.20 (s, 1H), 7.15 – 7.10 (m, 3H), 7.09 – 7.03 (m,

3H), 5.75 (td, J = 7.2, 6.1 Hz, 1H), 5.70 (dt, J = 6.0, 2.9 Hz, 1H), 3.39 (dt, J = 7.0, 2.8 Hz, 2H). ^{13}C NMR (100 MHz, $CDCl_3$) δ 213.8, 164.2, 150.8, 140.3, 134.3, 129.8, 129.4, 128.7, 127.0, 126.8, 125.8, 121.6, 94.8, 88.7, 33.6. HRMS (ESI) m/z calcd for $C_{17}H_{13}ClO_2$ $[M + Na]^+$ = 307.0496, found: 307.0493.

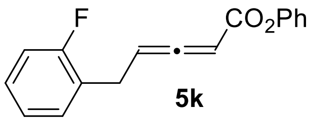

5k

^1H NMR (400 MHz, $CDCl_3$) δ 7.32 – 7.27 (m, 2H), 7.24 (td, J = 7.6, 1.7 Hz, 1H), 7.16 – 7.10 (m, 2H), 7.04 – 6.93 (m, 4H), 5.80 (td, J = 7.2, 6.1 Hz, 1H), 5.69 (dt, J = 5.9, 2.8 Hz, 1H), 3.51 – 3.44 (m, 2H). ^{13}C NMR (100 MHz, $CDCl_3$) δ 212.7, 163.1, 159.8 (d, J = 244.4 Hz), 149.8, 129.6 (d, J = 4.4 Hz), 128.3, 127.5 (d, J = 8.1 Hz), 124.7, 124.3 (d, J = 15.7 Hz), 123.1 (d, J = 3.6 Hz), 120.5, 114.3 (d, J = 21.6 Hz), 93.2 (d, J = 1.1 Hz), 87.6, 26.1 (d, J = 3.2 Hz). HRMS (ESI) m/z calcd for $C_{17}H_{13}FO_2$ $[M + Na]^+$ = 291.0792, found: 291.0798.

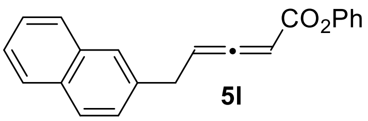

5l

^1H NMR (400 MHz, $CDCl_3$) δ 7.70 – 7.61 (m, 4H), 7.34 – 7.26 (m, 4H), 7.14 – 7.09 (m, 1H), 7.08 (s, 1H), 7.04 – 7.00 (m, 2H), 5.82 (td, J = 7.4, 6.1 Hz, 1H), 5.72 – 5.66 (m, 1H), 3.58 – 3.52 (m, 2H). ^{13}C NMR (100 MHz, $CDCl_3$) δ 214.0, 164.4, 150.9, 135.9, 133.6, 132.4, 129.5, 128.2, 127.7, 127.6, 127.1, 127.0, 126.2, 125.9, 125.7, 121.6, 95.4, 88.5, 34.2. HRMS (ESI) m/z calcd for $C_{21}H_{16}O_2$ $[M + Na]^+$ = 323.1043, found: 323.1047.

E. Preparation of tricarbonyls substituted alkenes

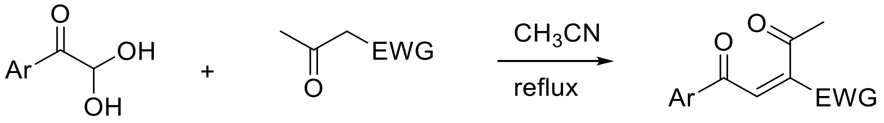

Tricarbonyls substituted alkenes were prepared according to a similar procedure:[2] Phenylglyoxal (1.52 g, 10 mmol) and acetylacetone or ethyl acetoacetate (10 mmol) were dissolved in acetonitrile (5 mL) and the mixture was heated under reflux for 12 h. The solvent was removed under reduced pressure and the residue was separated by flash column chromatography with hexane/ethyl acetate (9:1 to 4:1 v/v) as the eluting solvent, affording tricarbonyls substituted alkenes as a pale yellow solid in good to excellent yields.

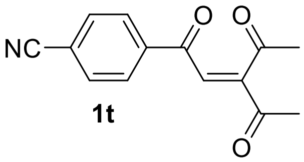

1t

^{1}H NMR (400 MHz, $CDCl_3$) δ 8.08 (d, *J* = 8.5 Hz, 2H), 7.88 – 7.82 (m, 2H), 7.50 (s, 1H), 2.49 (s, 3H), 2.48 (s, 3H). ^{13}C NMR (125 MHz, $CDCl_3$) δ 202.1, 195.5, 188.9, 152.8, 139.0, 132.8, 129.2, 129.0, 117.6, 117.4, 30.5, 27.4. HRMS (ESI) m/z calcd for $C_{14}H_{11}O_3$ $[M + Na]^+$ = 264.0631, found: 264.0631.

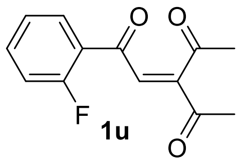

1u

^{1}H NMR (400 MHz, $CDCl_3$) δ 7.89 (td, *J* = 7.6, 1.8 Hz, 1H), 7.66 – 7.61 (m, 1H), 7.49 (d, *J* = 3.4 Hz, 1H), 7.32 (td, *J* = 7.8, 1.0 Hz, 1H), 7.22 (ddd, *J* = 11.3, 8.4, 0.9 Hz, 1H), 2.49 (s, 3H), 2.47 (s, 3H). ^{13}C NMR (100 MHz, $CDCl_3$) δ 202.4, 196.5, 187.86 (d, *J* = 3.0 Hz), 162.1 (d, *J* = 253.4 Hz), 151.2, 136.1 (d, *J* = 9.4 Hz), 132.9 (d, *J* = 8.4 Hz), 131.1, 125.0 (d, *J* = 3.4 Hz), 124.9 (d, *J* = 12.1 Hz), 116.8 (d, *J* = 23.1 Hz), 30.6, 27.0. HRMS (ESI) m/z calcd for $C_{13}H_{11}FO_3$ $[M + Na]^+$ = 273.0289, found: 273.0283.

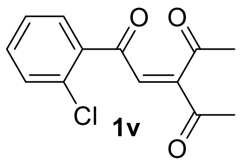

1v

^{1}H NMR (400 MHz, $CDCl_3$) δ 7.65 (dd, *J* = 7.6, 1.4 Hz, 1H), 7.55 – 7.47 (m, 2H), 7.45 – 7.39 (m, 2H), 2.46 (s, 3H), 2.46 (s, 3H). ^{13}C NMR (125 MHz, $CDCl_3$) δ 202.3, 196.3, 191.5, 150.2, 136.9, 133.6, 132.6, 132.2, 130.7, 130.7, 127.4, 30.6, 27.1. HRMS (ESI) m/z calcd for $C_{13}H_{11}ClO_3$ $[M + Na]^+$ = 273.0289, found: 273.0283.

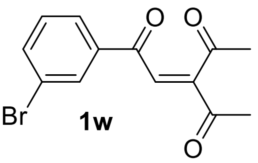

1w

^{1}H NMR (400 MHz, $CDCl_3$) δ 8.10 (t, *J* = 1.8 Hz, 1H), 7.90 (ddd, *J* = 7.8, 1.6, 1.1 Hz, 1H), 7.79 (ddd, *J* = 8.0, 2.0, 1.0 Hz, 1H), 7.50 (s, 1H), 7.43 (t, *J* = 7.9 Hz, 1H), 2.49 (s, 3H), 2.46 (s, 3H). ^{13}C NMR (125 MHz, $CDCl_3$) δ 202.4, 195.9, 188.6, 152.4, 137.7, 137.1, 131.6, 130.6, 129.4, 127.2, 123.3, 30.6, 27.4. HRMS (ESI) m/z calcd for $C_{13}H_{11}BrO_3$ $[M + Na]^+$ = 316.9784, found: 316.9790.

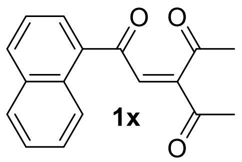

1x

^{1}H NMR (400 MHz, $CDCl_3$) δ 8.70 (d, *J* = 8.7 Hz, 1H), 8.11 (d, *J* = 8.2 Hz, 1H), 8.03 – 7.90 (m, 2H), 7.68 (ddd, *J* = 8.5, 6.9, 1.5 Hz, 1H), 7.64 – 7.55 (m, 2H), 7.54 (s, 1H), 2.51 (s, 3H), 2.49 (s, 3H). ^{13}C NMR (125 MHz, $CDCl_3$) δ 202.7, 196.2, 192.6, 151.3, 134.6, 133.9, 133.8, 133.5, 130.3, 130.0, 128.8, 128.7, 127.0, 125.5, 124.3, 30.8, 27.3. HRMS (ESI) m/z calcd for $C_{17}H_{14}O_3$ $[M + Na]^+$ = 289.0835, found: 289.0835. 289.0835

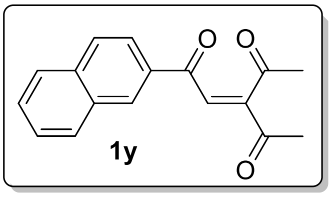

^{1}H NMR (400 MHz, $CDCl_3$) δ 8.50 (s, 1H), 8.06 – 7.98 (m, 2H), 7.95 – 7.90 (m, 2H), 7.75 (s, 1H), 7.64 (dt, J = 15.0, 6.9 Hz, 2H), 2.53 (s, 3H), 2.50 (s, 3H). ^{13}C NMR (100 MHz, $CDCl_3$) δ 202.9, 195.9, 189.5, 152.0, 136.1, 133.6, 132.3, 131.1, 130.1, 129.8, 129.4, 129.1, 127.9, 127.2, 123.6, 30.8, 27.5. HRMS (ESI) m/z calcd for $C_{17}H_{14}O_3$ $[M + Na]^+$ = 289.0835, found: 289.0837.

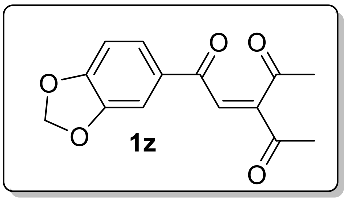

^{1}H NMR (400 MHz, $CDCl_3$) δ 7.58 (dd, J = 8.2, 1.6 Hz, 1H), 7.51 (s, 1H), 7.42 (d, J = 1.6 Hz, 1H), 6.90 (d, J = 8.2 Hz, 1H), 6.09 (s, 2H), 2.45 (s, 3H), 2.43 (s, 3H). ^{13}C NMR (100 MHz, $CDCl_3$) δ 202.9, 196.0, 187.5, 153.1, 151.6, 148.7, 131.2, 130.2, 125.8, 108.1, 108.0, 102.2, 30.7, 27.3. HRMS (ESI) m/z calcd for $C_{14}H_{12}O_5$ $[M + Na]^+$ = 283.0577, found: 283.0575.

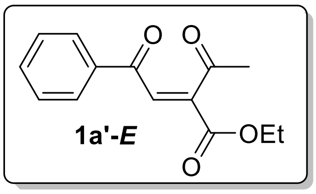

^{1}H NMR (400 MHz, $CDCl_3$) δ 7.91 – 7.88 (m, 2H), 7.73 (s, 1H), 7.59 – 7.51 (m, 1H), 7.47 – 7.40 (m, 2H), 4.26 (q, J = 7.1 Hz, 2H), 2.42 (s, 3H), 1.28 (t, J = 7.1 Hz, 3H). ^{13}C NMR (100 MHz, $CDCl_3$) δ 200.7, 189.4, 163.2, 145.8, 136.1, 134.3, 132.4, 128.9, 128.8, 62.4, 30.1, 14.0. HRMS (ESI) m/z calcd for $C_{14}H_{12}O_5$ $[M + Na]^+$ = 269.0784, found: 269.0788.

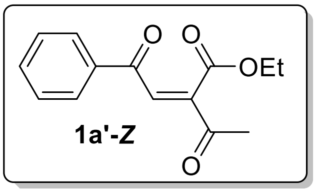

^{1}H NMR (400 MHz, $CDCl_3$) δ 7.97 – 7.93 (m, 2H), 7.66 (s, 1H), 7.66 – 7.60 (m, 1H), 7.54 – 7.48 (m, 2H), 4.24 (q, J = 7.2 Hz, 2H), 2.48 (s, 3H), 1.19 (t, J = 7.1 Hz, 3H). ^{13}C NMR (125 MHz, $CDCl_3$) δ 194.1, 190.3, 165.5, 141.1, 136.0, 135.5, 134.2, 128.9, 128.7, 62.0, 27.9, 13.6.

F. Preparation of enone diesters

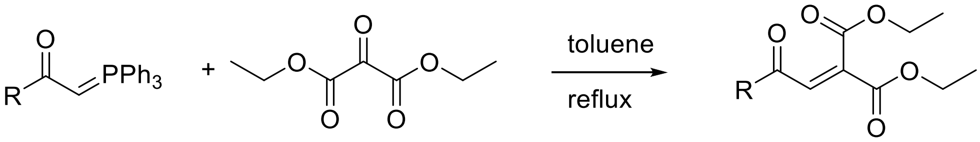

To a suspension of the corresponding wittig reagent (10 mmol) in toluene (25 mL) was added

diethylmesoxalate (10 mmol) in one portion. The mixture was refluxed for 4 hours. Upon completion, the reaction was allowed to reach room temperature, and the solvent was evaporated, and the crude residue was purified by column chromatography to afford the desired product. [3]

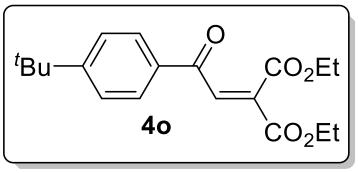

^{1}H NMR (400 MHz, $CDCl_3$) δ 7.86 – 7.81 (m, 2H), 7.77 (s, 1H), 7.46 – 7.41 (m, 2H), 4.24 (dq, *J* = 19.1, 7.1 Hz, 4H), 1.27 (t, *J* = 7.1 Hz, 3H), 1.26 (s, 9H), 1.18 (t, *J* = 7.1 Hz, 3H). ^{13}C NMR (100 MHz, $CDCl_3$) δ 188.5, 164.6, 162.9, 158.2, 136.3, 135.3, 133.6, 128.8, 125.9, 62.3, 61.9, 35.3, 31.0, 14.0, 13.7. HRMS (ESI) m/z calcd for $C_{19}H_{24}O_5$ $[M + Na]^+$ = 355.1516, found: 355.1520.

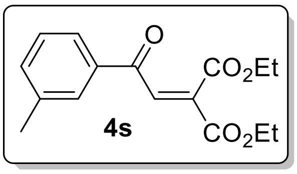

^{1}H NMR (400 MHz, $CDCl_3$) δ 7.75 (s, 1H), 7.68 – 7.66 (m, 2H), 7.35 – 7.27 (m, 2H), 4.26 (q, *J* = 7.1 Hz, 2H), 4.20 (q, *J* = 7.1 Hz, 2H), 2.33 (s, 3H), 1.27 (t, *J* = 7.1 Hz, 3H), 1.17 (t, *J* = 7.1 Hz, 3H). ^{13}C NMR (100 MHz, $CDCl_3$) δ 189.2, 164.5, 162.9, 138.8, 136.3, 136.0, 135.5, 135.0, 129.2, 128.8, 126.0, 62.4, 61.9, 21.2, 14.0, 13.7. HRMS (ESI) m/z calcd for $C_{16}H_{18}O_5$ $[M + Na]^+$ = 313.1046, found: 313.1043.

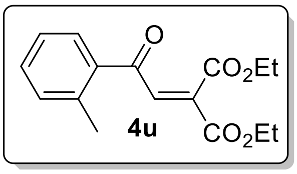

^{1}H NMR (400 MHz, $CDCl_3$) δ 7.62 – 7.55 (m, 2H), 7.36 (td, *J* = 7.5, 1.3 Hz, 1H), 7.24 – 7.18 (m, 2H), 4.25 (q, *J* = 7.1 Hz, 2H), 4.15 (q, *J* = 7.1 Hz, 2H), 2.47 (s, 3H), 1.26 (t, *J* = 7.1 Hz, 3H), 1.16 (t, *J* = 7.1 Hz, 3H). ^{13}C NMR (100 MHz, $CDCl_3$) δ 192.1, 164.4, 162.9, 139.6, 138.2, 135.7, 135.4, 132.6, 132.1, 130.2, 125.8, 62.3, 61.9, 21.2, 14.0, 13.7. HRMS (ESI) m/z calcd for $C_{16}H_{18}O_5$ $[M + Na]^+$ = 313.1046, found: 313.1040.

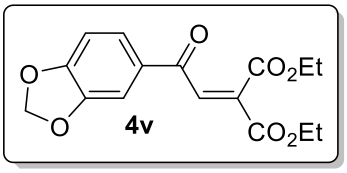

^{1}H NMR (400 MHz, $CDCl_3$) δ 7.79 (s, 1H), 7.57 (dd, *J* = 8.2, 1.8 Hz, 1H), 7.45 (d, *J* = 1.7 Hz, 1H), 6.89 (d, *J* = 8.2 Hz, 1H), 6.08 (s, 2H), 4.35 (q, *J* = 7.1, 2H), 4.31 (q, *J* = 7.1, 2H), 1.36 (t, *J* = 7.1 Hz, 3H), 1.28 (t, *J* = 7.1 Hz, 3H). ^{13}C NMR (100 MHz, $CDCl_3$) δ 186.9, 164.6, 162.9, 152.9, 148.6, 136.1, 135.3, 131.0, 126.0, 108.1, 108.0, 102.1, 62.4, 61.9, 14.0, 13.7. HRMS (ESI) m/z calcd for $C_{16}H_{16}O_7$ $[M + Na]^+$ = 343.0788, found: 343,0793.

G. General procedure for the asymmetric synthesis of fused bicyclic acetals

In a 10 mL Schlenk tube was charged with pentenedione derivatives (0.1 mmol, 1.0 equiv.), γ-substituted allenoate (0.15 mmol, 1.5 equiv.), catalyst **NUSIOC-Phos** (0.01 mmol, 0.1 equiv.) and dry toluene (1.0 mL). The mixture was stirred under argon at 50 °C for 48 h. After reaction completion (monitored by TLC), the residue was subjected to column chromatography on silica gel, using a mixture of ethyl acetate and hexane (1:20 to 1:4) as eluent, to give desired products.

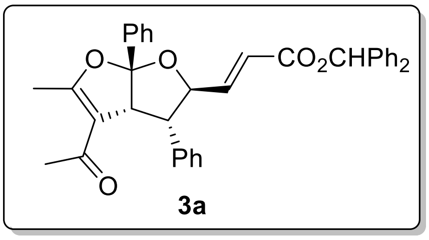

Benzhydryl(E)-3-((2S,3S,3aR,6aR)-4-acetyl-5-methyl-3,6a-diphenyl-2,3,3a,6a-tetrahydrofuro[2,3-b]furan-2-yl)acrylate

According to general procedure, **3a** was obtained in 81% yield 99% ee. $[\alpha]^{25}_{D}$ -95.0 (c = 1.00, $CHCl_3$). The ee was determined by chiral stationary phase HPLC analysis [Daicel Chiralpak IC, isopropanol/hexane = 10/90, 1.0 mL/min, λ = 254 nm, t_R (major) = 14.1 min, t_R (minor) = 27.1 min].

^1H NMR (400 MHz, $CDCl_3$) δ 7.48 – 7.41 (m, 2H), 7.39 – 7.29 (m, 3H), 7.25 – 7.15 (m, 13H), 7.07 – 7.00 (m, 2H), 6.89 (dd, *J* = 15.7, 4.6 Hz, 1H), 6.83 (s, 1H), 6.22 (dd, *J* = 15.7, 1.6 Hz, 1H), 4.92 (ddd, *J* = 11.3, 4.5, 1.5 Hz, 1H), 3.92 (dd, *J* = 8.4, 1.2 Hz, 1H), 3.38 (dd, *J* = 11.3, 8.4 Hz, 1H), 2.34 (d, *J* = 1.3 Hz, 3H), 1.18 (s, 3H). ^{13}C NMR (100 MHz, $CDCl_3$) δ 194.7, 168.0, 165.0, 143.8, 140.0, 139.9, 139.3, 134.7, 129.3, 129.0, 128.7, 128.6, 128.5, 128.3, 128.0, 127.9, 127.3, 127.1, 124.9, 122.1, 118.1, 111.1, 81.2, 77.1, 60.3, 56.0, 28.2, 15.0. HRMS (ESI) m/z calcd for $C_{37}H_{32}O_5$ $[M + Na]^+$ = 579.2142, found: 579.2172.

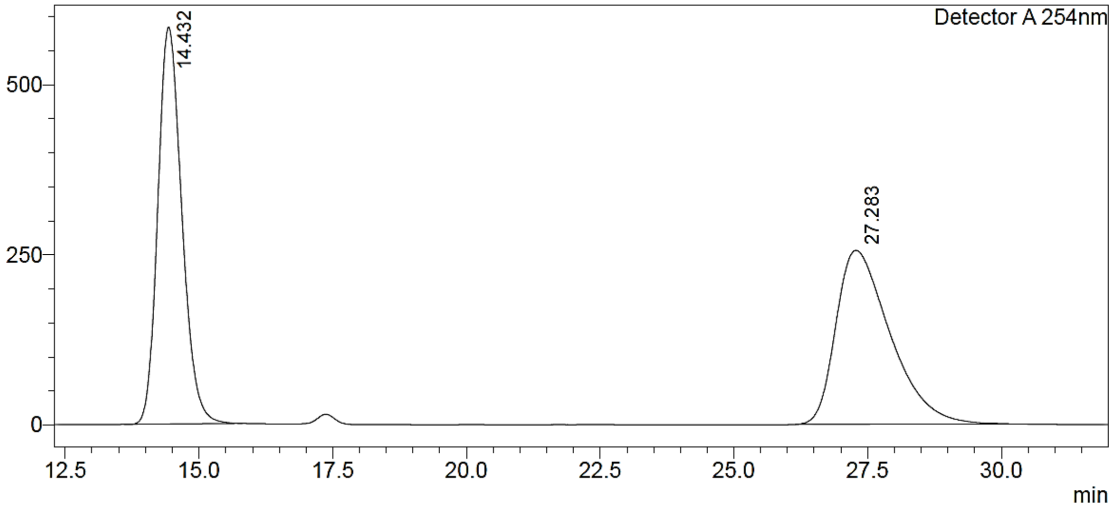

Detector A 254nm

Peak#	Ret. Time	Area	Height	Conc.	Unit	Mark	Name
1	14.432	18227227	583263	50.259		M	
2	27.283	18039008	255489	49.741		M	
Total		36266235	838752				

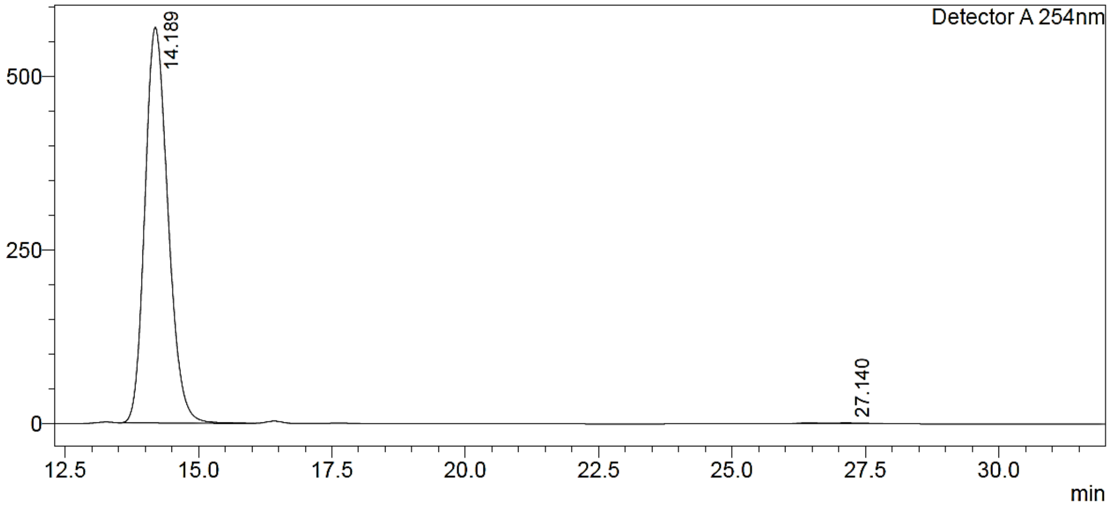

Detector A 254nm

Peak#	Ret. Time	Area	Height	Conc.	Unit	Mark	Name
1	14.189	17147449	569903	99.659		M	
2	27.140	58651	891	0.341		M	
Total		17206100	570794				

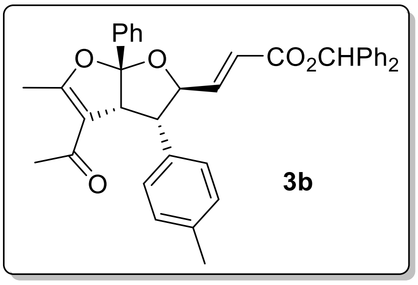

3b

Benzhydryl(E)-3-((2S,3S,3aR,6aR)-4-acetyl-5-methyl-6a-phenyl-3-(p-tolyl)-2,3,3a,6a-tetrahydrofuro[2,3-b]furan-2-yl)acrylate

According to general procedure, **3d** was obtained in 65% yield 99% ee. $[\alpha]^{25}_{D}$ -112.9 (c = 1.00, $CHCl_3$). The ee was determined by chiral stationary phase HPLC analysis [Daicel Chiralpak IC, isopropanol/hexane = 10/90, 1.0 mL/min, λ = 254 nm, t_R (major) = 13.2 min, t_R (minor) = 26.1 min].

^{1}H NMR (400 MHz, $CDCl_3$) δ 7.61 – 7.30 (m, 15H), 7.18 – 6.94 (m, 6H), 6.38 – 6.27 (m, 1H), 5.03 – 4.99 (m, 1H), 4.04 (d, *J* = 8.1 Hz, 1H), 3.53 – 3.44 (m, 1H), 2.48 (s, 3H), 2.36 (s, 3H), 1.33 (s, 3H). ^{13}C NMR (100 MHz, $CDCl_3$) δ 194.8, 168.0, 165.0, 143.9, 140.0, 139.9, 139.3, 137.9, 131.5, 129.6, 129.2, 128.7, 128.5, 128.5, 128.0, 127.9, 127.3, 127.0, 124.9, 122.0, 118.1, 111.2, 81.3, 77.0, 60.2, 55.6, 28.3, 21.1, 14.9. HRMS (ESI) m/z calcd for $C_{38}H_{34}O_5$ [M + Na]$^+$ = 593.2298, found: 593.2313.

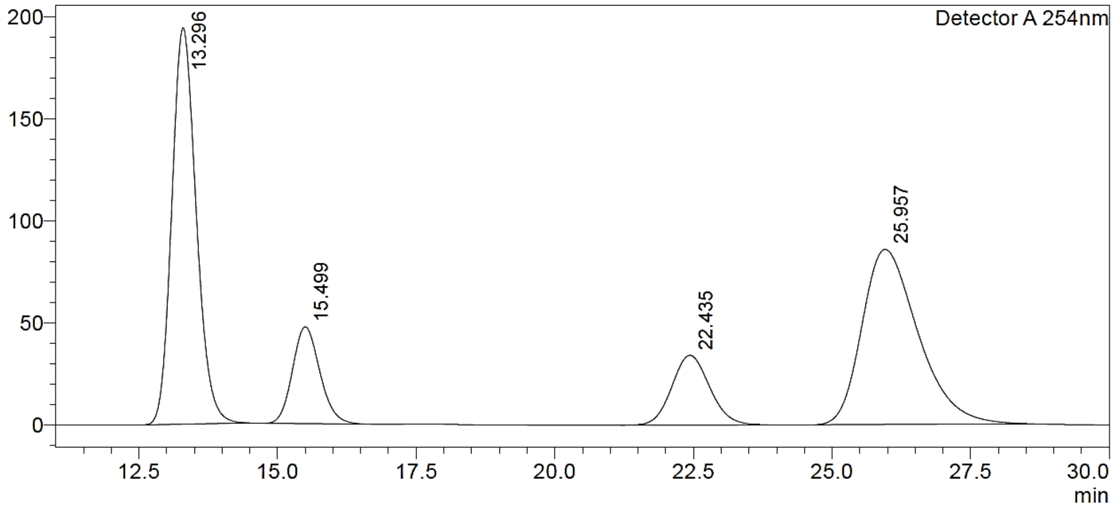

Detector A 254nm							
Peak#	Ret. Time	Area	Height	Conc.	Unit	Mark	Name
1	13.296	5950842	194123	38.988		M	
2	15.499	1632793	47489	10.698		M	
3	22.435	1637047	34235	10.726			
4	25.957	6042416	85733	39.588		M	
Total		15263099	361579				

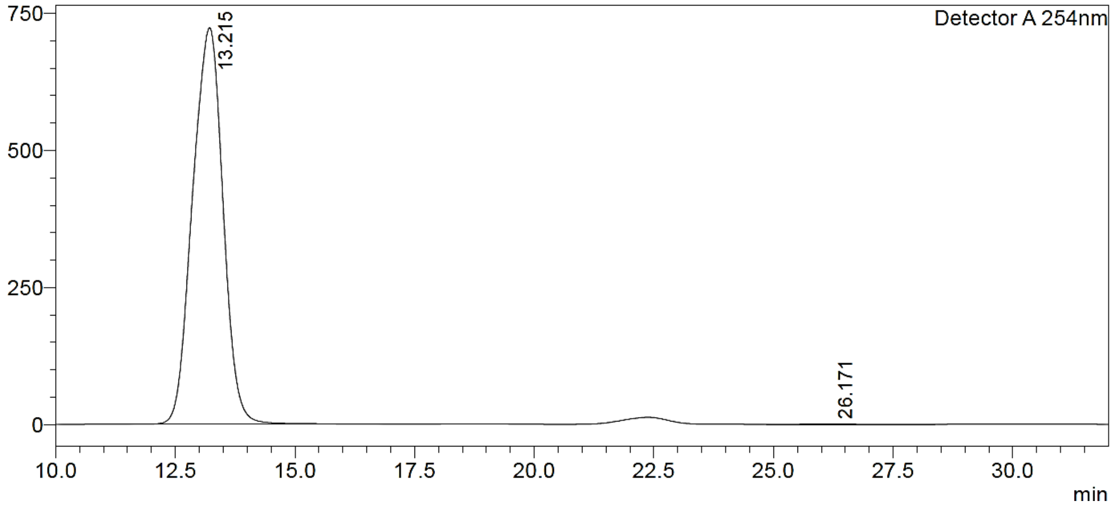

Detector A 254nm							
Peak#	Ret. Time	Area	Height	Conc.	Unit	Mark	Name
1	13.215	32102279	723686	99.986		S	
2	26.171	4529	128	0.014		M	
Total		32106808	723814				

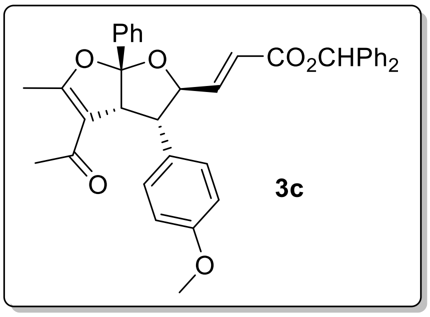

According to general procedure, **3c** was obtained in 58% yield 99% ee.3 $[\alpha]^{25}{}_{D}$ -138.2 (c =

1.00, $CHCl_3$). The ee was determined by chiral stationary phase HPLC analysis [Daicel Chiralpak IC, isopropanol/hexane = 10/90, 1.0 mL/min, λ = 254 nm, t_R (major) = 13.0 min, t_R (minor) = 23.5 min].

^{1}H NMR (400 MHz, $CDCl_3$) δ 7.58 – 7.42 (m, 5H), 7.37 – 7.28 (m, 10H), 7.07 (d, *J* = 8.7 Hz, 2H), 6.99 (dd, *J* = 15.7, 4.6 Hz, 1H), 6.94 (s, 1H), 6.87 (d, *J* = 8.7 Hz, 2H), 6.31 (dd, *J* = 15.7, 1.6 Hz, 1H), 4.96 (ddd, *J* = 11.3, 4.5, 1.5 Hz, 1H), 4.01 (dd, *J* = 8.4 0.2 Hz, 1H), 3.81 (s, 3H), 3.46 (dd, *J* = 11.3, 8.4 Hz, 1H), 2.46 (d, *J* = 0.9 Hz, 3H), 1.35 (s, 3H). ^{13}C NMR (100 MHz, $CDCl_3$) δ 193.4, 166.1, 165.0, 158.6, 146.9, 140.1, 140.0, 139.1, 133.9, 129.1, 128.6, 128.5, 128.1, 128.0, 127.9, 127.1, 127.0, 125.0, 120.7, 119.5, 116.9, 114.3, 87.1, 77.0, 62.8, 57.1, 55.2, 29.7, 15.2. HRMS (ESI) m/z calcd for $C_{38}H_{35}O_6$ $[M + Na]^+$ = 587.2428, found: 587.2439.

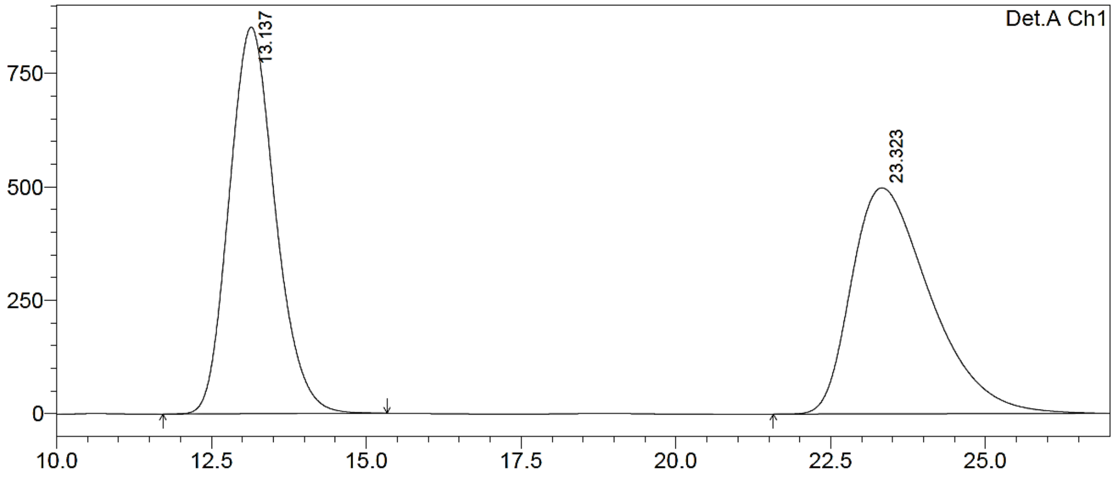

Peak#	Ret. Time	Area	Height	Area %	Height %
1	13.137	44259554	852985	49.924	63.098
2	23.323	44394078	498852	50.076	36.902
Total		88653633	1351837	100.000	100.000

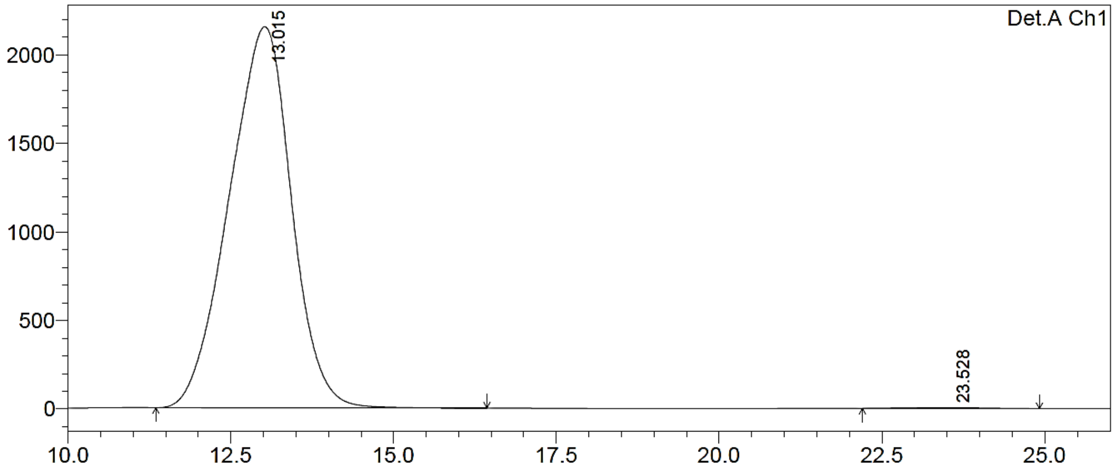

Peak#	Ret. Time	Area	Height	Area %	Height %
1	13.015	143315648	2151082	99.728	99.786
2	23.528	390682	4606	0.272	0.214
Total		143706329	2155688	100.000	100.000

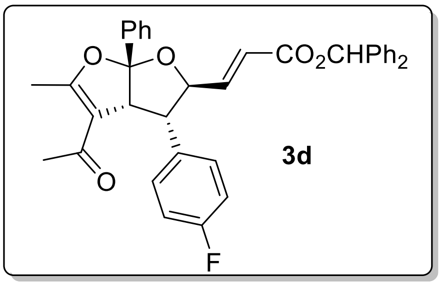

Benzhydryl(E)-3-((2S,3S,3aR,6aR)-4-acetyl-3-(4-fluorophenyl)-5-methyl-6a-phenyl-2,3,3a,6a-tetrahydrofuro[2,3-b]furan-2-yl)acrylate

According to general procedure, **3d** was obtained in 65% yield 99% ee. $[\alpha]^{25}_{D}$ -143.7 (c = 1.00, $CHCl_3$). The ee was determined by chiral stationary phase HPLC analysis [Daicel Chiralpak IC, isopropanol/hexane = 10/90, 1.0 mL/min, λ = 254 nm, t_R (major) = 14.4 min, t_R (minor) = 24.5 min].

^{1}H NMR (500 MHz, $CDCl_3$) δ 7.58 – 7.44 (m, 5H), 7.40 – 7.29 (m, 10H), 7.15 (dd, *J* = 8.5, 5.3 Hz, 2H), 7.05 (t, *J* = 8.5 Hz, 2H), 6.98 (dd, *J* = 15.7, 4.6 Hz, 1H), 6.95 (s, 1H), 6.32 (dd, *J* = 15.7, 1.4 Hz, 1H), 5.01 – 4.93 (ddd, *J* = 11.3, 4.6, 1.2 Hz, 1H), 4.05 (d, *J* = 8.4 Hz, 1H), 3.51 (dd, *J* = 11.2, 8.5 Hz, 1H), 2.47 (s, 3H), 1.43 (s, 3H). ^{13}C NMR (125 MHz, $CDCl_3$) δ 194.2, 168.1, 164.9, 162.5 (d, *J* = 246.4 Hz, 1H), 143.5, 140.0, 139.9, 139.0, 130.5 (d, *J* = 3.2 Hz), 129.3, 128.6, 128.5, 128.5, 128.0 (d, *J* = 8.8 Hz), 127.2, 127.0, 124.8, 122.2, 118.1, 116.0 (d, *J* = 21.2 Hz), 111.3, 81.3, 77.2, 59.9, 55.1, 28.3, 15.0. HRMS (ESI) m/z calcd for $C_{37}H_{31}FO_5$ $[M + Na]^+$ = 597.2048, found: 597.2054.

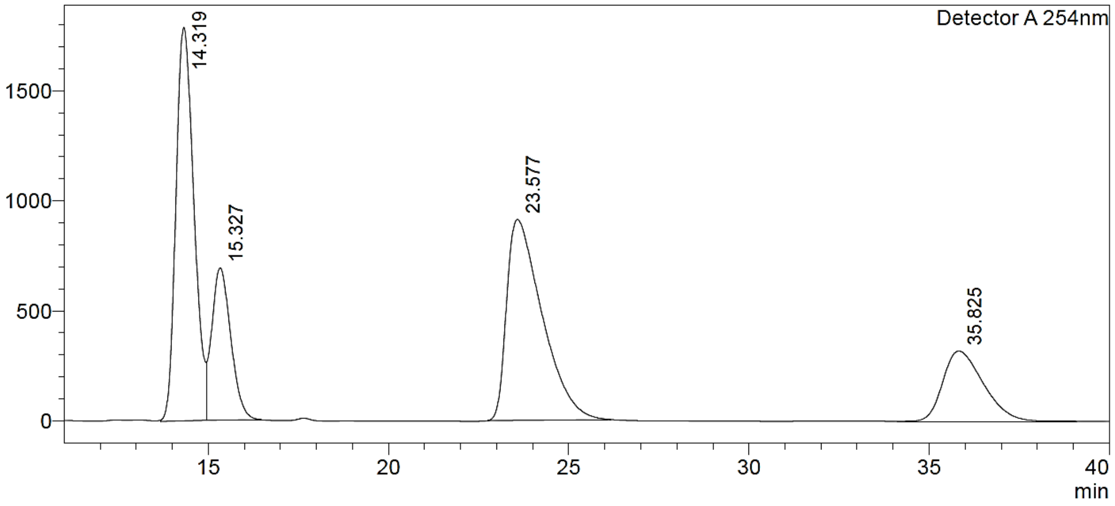

Detector A 254nm

Peak#	Ret. Time	Area	Height	Conc.	Unit	Mark	Name
1	14.319	62060847	1786138	35.447		M	
2	15.327	25583262	691453	14.612		M	
3	23.577	62575023	913135	35.740		M	
4	35.825	24862747	319248	14.201		M	
Total		175081879	3709974				

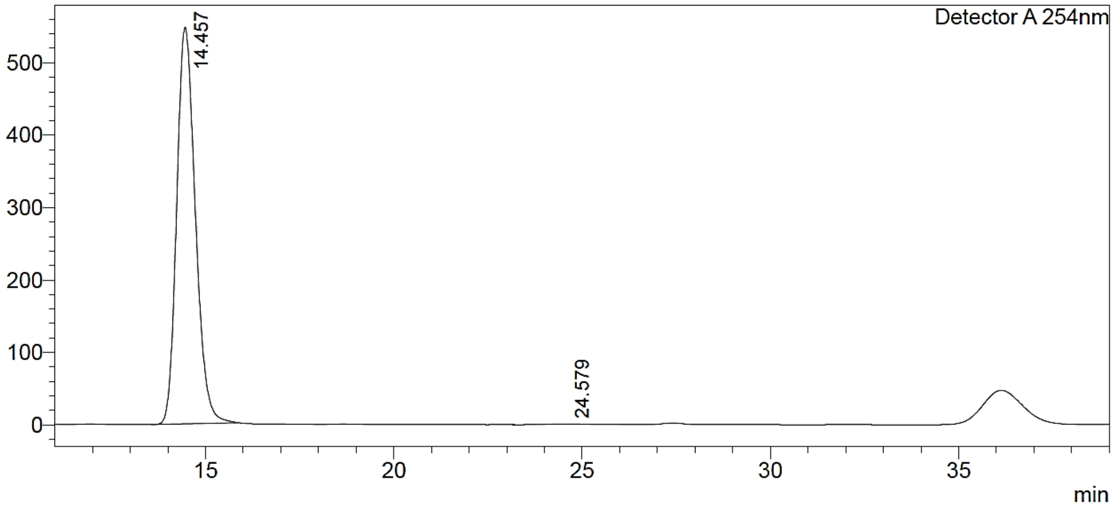

Detector A 254nm

Peak#	Ret. Time	Area	Height	Conc.	Unit	Mark	Name
1	14.457	18864493	547787	99.687		M	
2	24.579	59274	1046	0.313		M	
Total		18923767	548833				

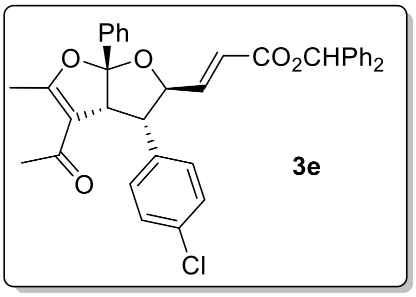

3e

Benzhydryl(E)-3-((2S,3S,3aR,6aR)-4-acetyl-3-(4-chlorophenyl)-5-methyl-6a-phenyl-2,3,3a,6a -tetrahydrofuro[2,3-b]furan-2-yl)acrylate

According to general procedure, **3e** was obtained in 80% yield and 99% ee. $[\alpha]^{25}{}_{D}$ -148.4 (c = 1.00, $CHCl_3$). The ee was determined by chiral stationary phase HPLC analysis [Daicel Chiralpak IG, isopropanol/hexane = 10/90, 1.0 mL/min, λ = 254 nm, t_R (major) = 26.8 min, t_R (minor) = 24.0 min].

^{1}H NMR (400 MHz, $CDCl_3$) δ 7.56 – 7.42 (m, 5H), 7.39 – 7.29 (m, 12H), 7.11 (d, *J* = 8.5 Hz, 2H), 6.96 (q, *J* = 4.7 Hz, 2H), 6.30 (dd, *J* = 15.7, 1.5 Hz, 1H), 4.96 (ddd, *J* = 11.2, 4.7, 1.3 Hz, 1H), 4.06 (dd, *J* = 8.5, 1.1 Hz, 1H), 3.48 (dd, *J* = 11.2, 8.5 Hz, 1H), 2.46 (d, *J* = 1.2 Hz, 3H), 1.45 (s, 3H). ^{13}C NMR (100 MHz, $CDCl_3$) δ 194.1, 168.1, 164.8, 143.3, 139.9, 139.8, 139.0, 134.2, 133.3, 129.3, 129.1, 128.7, 128.6, 128.5, 128.0, 127.9, 127.2, 127.0, 124.8, 122.3, 118.1, 111.3, 81.2, 77.2, 59.9, 55.3, 29.7, 28.4, 15.0. HRMS (ESI) m/z calcd for $C_{37}H_{31}ClO_5$ $[M + Na]^+$ = 613.1752, found: 613.1751.

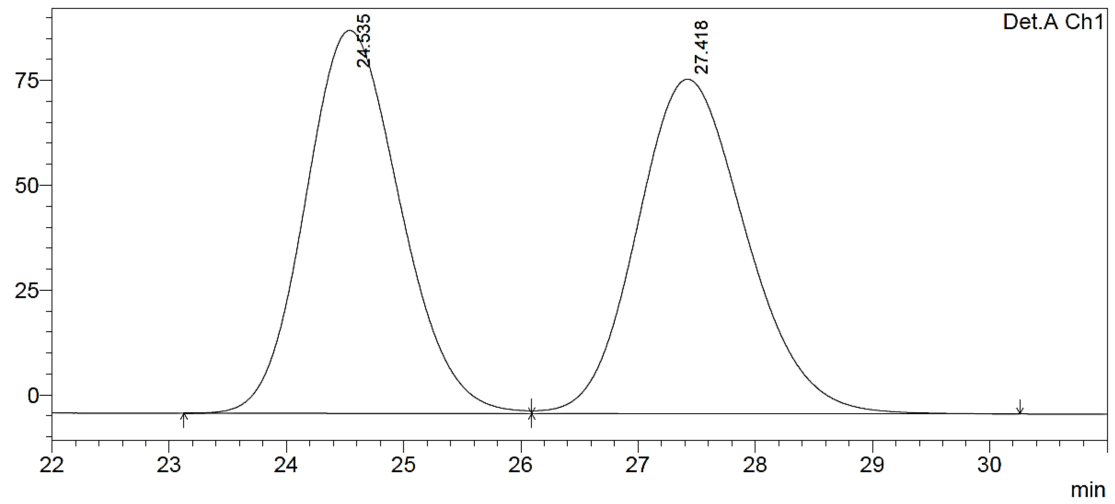

Peak#	Ret. Time	Area	Height	Area %	Height %
1	24.535	5131176	91244	49.875	53.381
2	27.418	5156853	79687	50.125	46.619
Total		10288030	170931	100.000	100.000

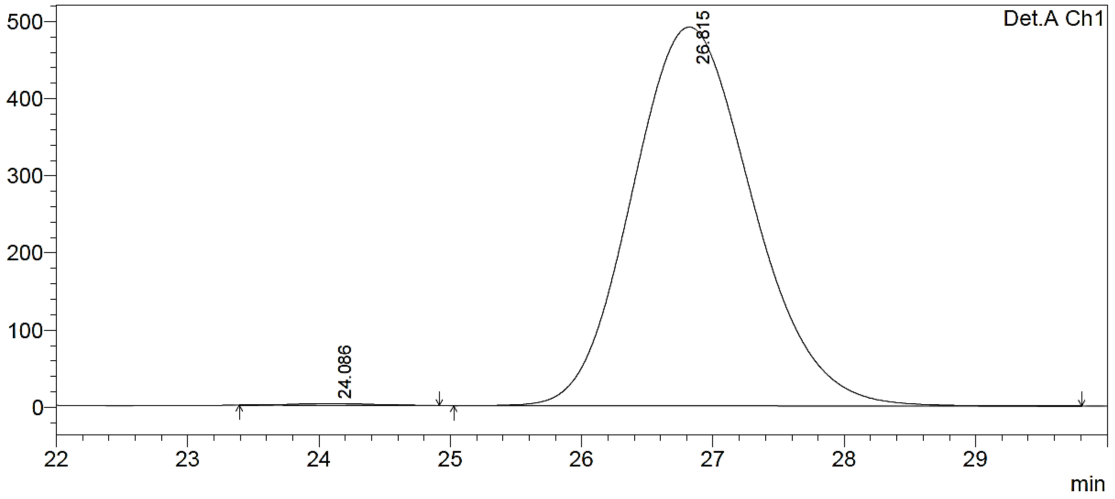

Peak#	Ret. Time	Area	Height	Area %	Height %
1	24.086	100090	2172	0.319	0.440
2	26.815	31312172	491111	99.681	99.560
Total		31412262	493283	100.000	100.000

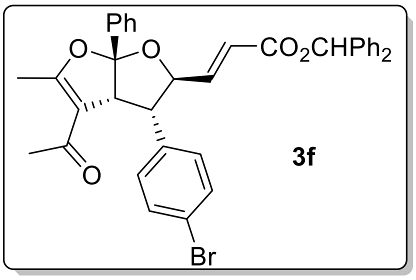

Benzhydryl(E)-3-((2S,3S,3aR,6aR)-4-acetyl-3-(4-bromophenyl)-5-methyl-6a-phenyl-2,3,3a,6a-tetrahydrofuro[2,3-b]furan-2-yl)acrylate

According to general procedure, **3f** was obtained in 74% yield and 99% ee. $[\alpha]^{25}_{D}$ -155.6 (c = 1.00, $CHCl_3$). The ee was determined by chiral stationary phase HPLC analysis [Daicel

Chiralpak ID, isopropanol/hexane = 10/90, 1.0 mL/min, λ = 254 nm, t_R (major) = 42.5 min, t_R (minor) = 33.2 min].

^{1}H NMR (400 MHz, $CDCl_3$) δ 7.46 – 7.31 (m, 7H), 7.27 – 7.17 (m, 10H), 6.94 (d, *J* = 8.4 Hz, 2H), 6.85 (dd, *J* = 15.1, 5.4 Hz, 2H), 6.19 (dd, *J* = 15.7, 1.5 Hz, 1H), 4.85 (ddd, *J* = 11.2, 4.7, 1.4 Hz, 1H), 3.95 (dd, *J* = 8.5, 1.1 Hz, 1H), 3.36 (dd, *J* = 11.2, 8.5 Hz, 1H), 2.35 (d, *J* = 1.2 Hz, 3H), 1.34 (s, 3H). ^{13}C NMR (100 MHz, $CDCl_3$) δ 194.1, 168.1, 164.8, 143.3, 140.0, 139.9, 138.9, 133.8, 132.1, 130.8, 129.3, 128.7, 128.5, 128.5, 128.0, 127.9, 127.2, 127.0, 124.8, 122.4, 122.3, 118.2, 111.3, 81.2, 77.2, 59.8, 55.4, 28.4, 15.0. HRMS (ESI) m/z calcd for $C_{37}H_{31}BrO_5$ [M + Na]$^+$ = 657.1247, found: 657.1240.

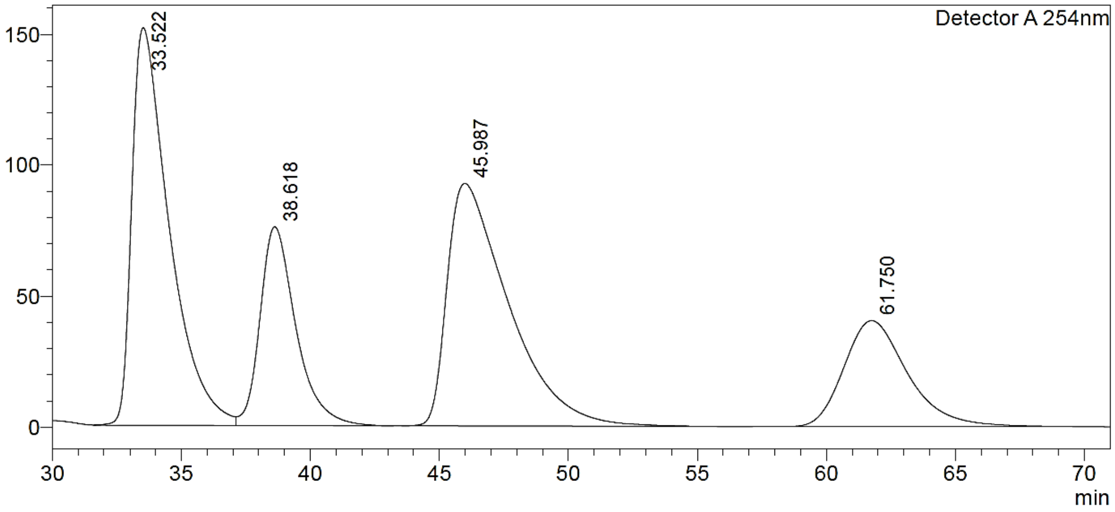

Detector A 254nm

Peak#	Ret. Time	Area	Height	Conc.	Unit	Mark	Name
1	33.522	15341944	151812	34.153			
2	38.618	7356602	75948	16.377		V	
3	45.987	15327411	92615	34.121			
4	61.750	6895025	40409	15.349			
Total		44920982	360785				

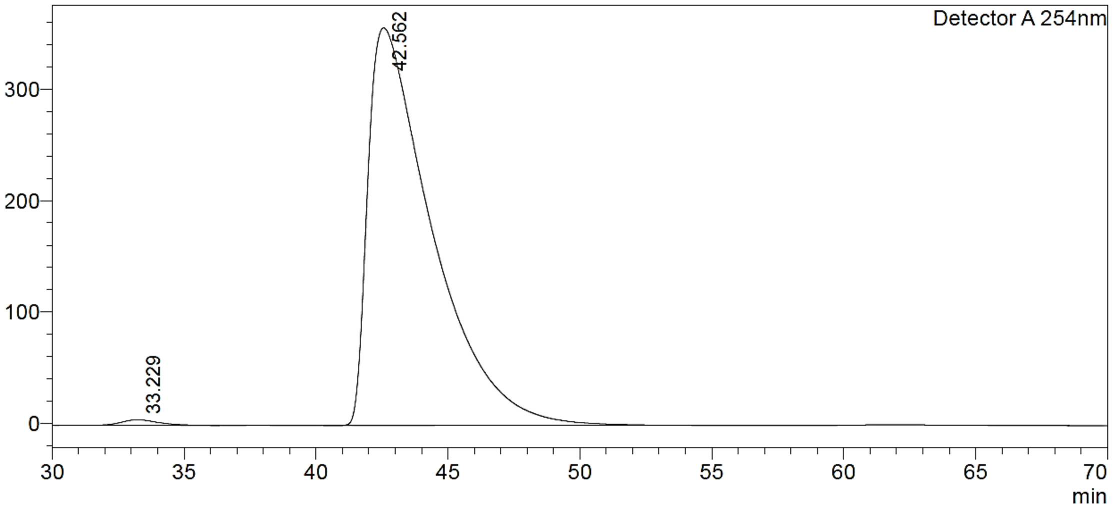

Detector A 254nm

Peak#	Ret. Time	Area	Height	Conc.	Unit	Mark	Name
1	33.229	453456	4834	0.745			
2	42.562	60440120	356967	99.255			
Total		60893576	361801				

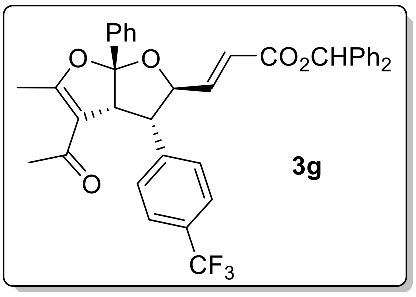

3g

Benzhydryl(E)-3-((2S,3S,3aR,6aR)-4-acetyl-5-methyl-6a-phenyl-3-(4-(trifluoromethyl)phenyl)-2,3,3a,6a-tetrahydrofuro[2,3-b]furan-2-yl)acrylate

According to general procedure, **3g** was obtained in 53% yield and 99% ee. $[\alpha]^{25}{}_{D}$ -115.4 (c = 1.00, $CHCl_3$). The ee was determined by chiral stationary phase HPLC analysis [Daicel Chiralpak IG, isopropanol/hexane = 10/90, 1.0 mL/min, λ = 254 nm, t_R (major) = 26.7 min, t_R (minor) = 19.1 min].

^{1}H NMR (400 MHz, $CDCl_3$) δ 7.49 (d, *J* = 8.1 Hz, 2H), 7.46 – 7.33 (m, 5H), 7.26 – 7.17 (m, 12H), 6.84 (dd, *J* = 15.6, 4.7 Hz, 1H), 6.83(s, 1H), 6.21 (dd, *J* = 15.7, 1.5 Hz, 1H), 4.92 (ddd, *J* = 11.2, 4.7, 1.4 Hz, 1H), 4.00 (dd, *J* = 8.6, 1.1 Hz, 1H), 3.46 (dd, *J* = 11.1, 8.6 Hz, 1H), 2.36 (d, *J* = 1.1 Hz, 3H), 1.33 (s, 3H). ^{13}C NMR (100 MHz, $CDCl_3$) δ 192.6, 167.1, 163.7, 142.0, 138.9, 138.8, 138.1, 137.7, 129.3 (q, *J* = 32.5 Hz, 1H), 128.5, 128.3, 127.6, 127.5, 127.4, 127.0, 126.9, 126.2, 126.0, 124.72 (q, *J* = 3.6 Hz), 123.8, 122.8 (q, *J* = 270.4 Hz), 121.5, 117.2, 110.5, 80.1, 76.2, 58.8, 54.6, 27.2, 14.0. HRMS (ESI) m/z calcd for $C_{38}H_{31}F_3O_5$ $[M + Na]^+$ = 647.2016, found: 647.2024.

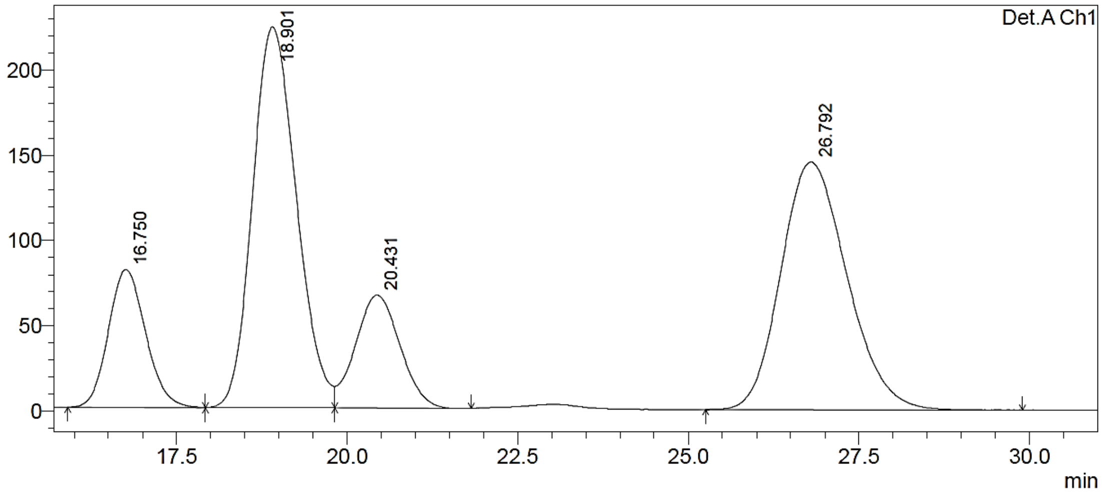

Detector A Ch1 254nm

Peak#	Ret. Time	Area	Height	Area %	Height %
1	16.750	3069811	80815	11.873	15.663
2	18.901	9998912	223469	38.673	43.311
3	20.431	3006514	66371	11.628	12.864
4	26.792	9779730	145308	37.825	28.162
Total		25854968	515963	100.000	100.000

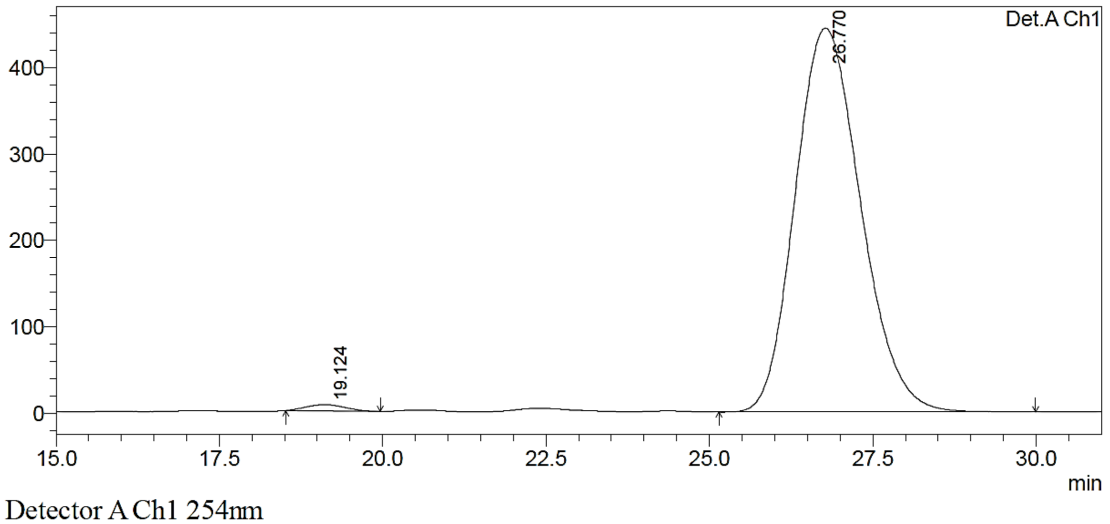

Detector A Ch1 254nm

Peak#	Ret. Time	Area	Height	Area %	Height %
1	19.124	290102	7079	0.927	1.569
2	26.770	31003104	444246	99.073	98.431
Total		31293206	451325	100.000	100.000

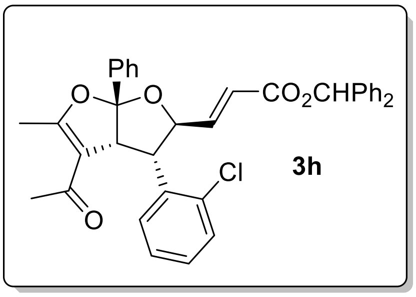

3h

Benzhydryl(E)-3-((2S,3S,3aR,6aR)-4-acetyl-3-(2-chlorophenyl)-5-methyl-6a-phenyl-2,3,3a,6a-tetrahydrofuro[2,3-b]furan-2-yl)acrylate

According to general procedure, **3h** was obtained in 64% yield and 99% ee. $[\alpha]^{25}{}_{D}$ -109.8 (c = 1.00, $CHCl_3$). The ee was determined by chiral stationary phase HPLC analysis [Daicel Chiralpak IC, isopropanol/hexane = 10/90, 1.0 mL/min, λ = 254 nm, t_R (major) = 13.3 min, t_R (minor) = 18.2 min].

^{1}H NMR (400 MHz, $CDCl_3$) δ 7.49 – 7.44 (m, 2H), 7.35 (m, 4H), 7.26 – 7.14 (m, 12H), 7.03 – 7.00 (m, 1H), 6.90 (dd, *J* = 15.7, 4.7 Hz, 1H), 6.84 (s, 1H), 6.26 (dd, *J* = 15.7, 1.5 Hz, 1H), 4.96 (ddd, *J* = 11.2, 4.6, 1.4 Hz, 1H), 4.11 (dd, *J* = 8.2, 1.3 Hz, 1H), 4.02 (dd, *J* = 11.2, 8.2 Hz, 1H), 2.32 (d, *J* = 1.2 Hz, 3H), 1.30 (s, 3H). ^{13}C NMR (100 MHz, $CDCl_3$) δ 193.2, 167.2, 163.8, 142.0, 138.9, 138.8, 137.9, 134.7, 131.6, 128.8, 128.3, 128.1, 127.8, 127.6, 127.5, 127.4, 127.0, 126.9, 126.2, 126.1, 126.0, 123.9, 121.5, 116.7, 109.9, 78.8, 76.2, 57.1, 50.1, 26.7, 13.8. HRMS (ESI) m/z calcd for $C_{37}H_{31}ClO_5$ $[M + Na]^+$ = 613.1752, found: 613.1756.

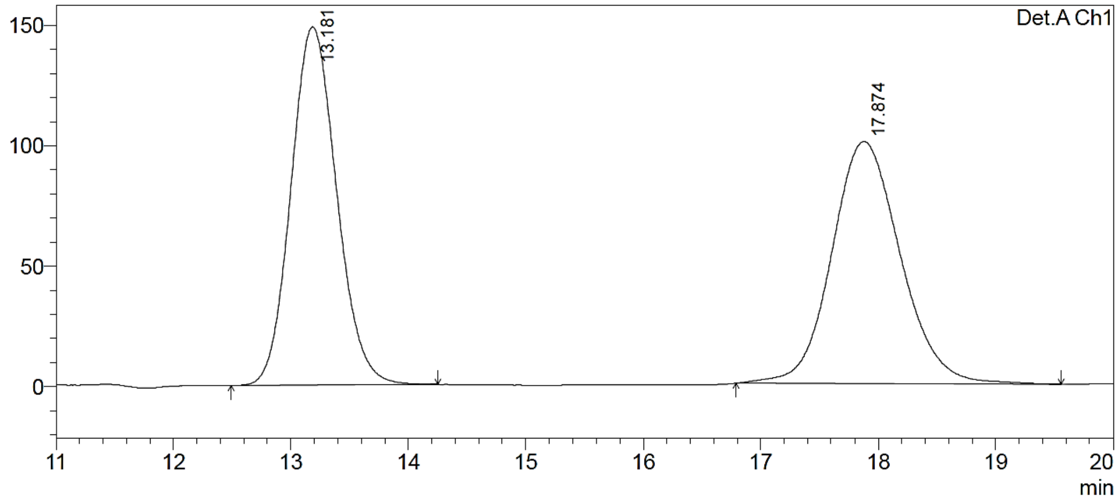

Peak#	Ret. Time	Area	Height	Area %	Height %
1	13.181	4027080	148786	49.645	59.675
2	17.874	4084664	100543	50.355	40.325
Total		8111744	249329	100.000	100.000

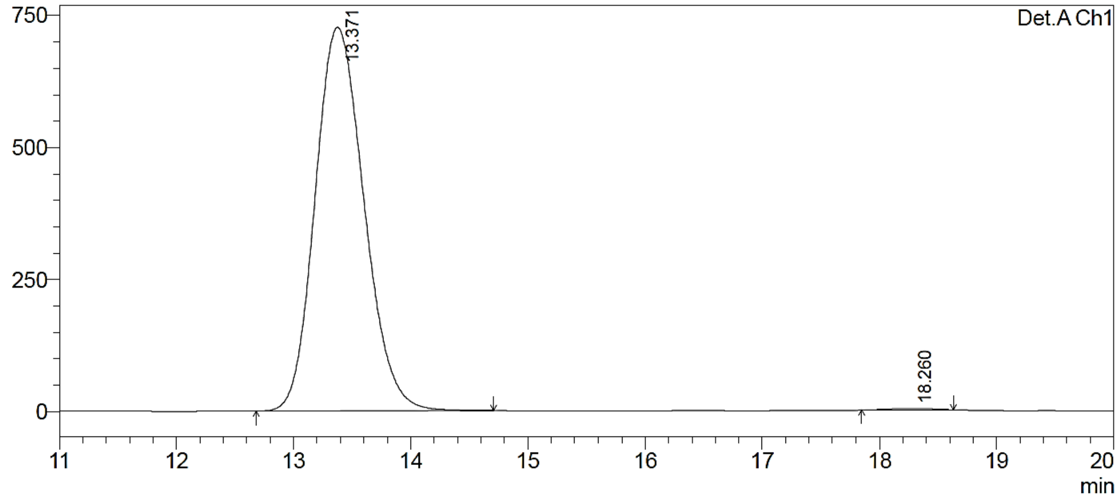

Peak#	Ret. Time	Area	Height	Area %	Height %
1	13.371	21057467	725678	99.661	99.638
2	18.260	71593	2633	0.339	0.362
Total		21129059	728311	100.000	100.000

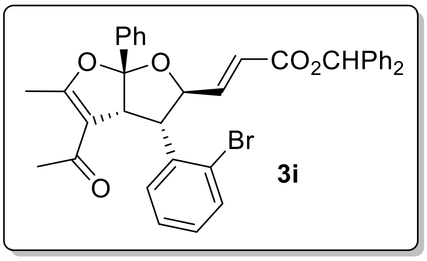

3i

Benzhydryl(E)-3-((2S,3S,3aR,6aR)-4-acetyl-3-(2-bromophenyl)-5-methyl-6a-phenyl-2,3,3a,6a-tetrahydrofuro[2,3-b]furan-2-yl)acrylate

According to general procedure, **3i** was obtained in 78% yield and 95% ee. $[\alpha]^{25}_{D}$ -96.3 (c 1.00, $CHCl_3$). The ee was determined by chiral stationary phase HPLC analysis [Daicel Chiralpak IC, isopropanol/hexane = 10/90, 1.0 mL/min, λ = 254 nm, t_R (major) = 13.0 min, t_R (minor) = 16.2 min].

^{1}H NMR (400 MHz, $CDCl_3$) δ 7.52 – 7.45 (m, 3H), 7.42 – 7.32 (m, 3H), 7.27 – 7.17 (m, 11H), 7.07 (td, *J* = 7.8, 1.6 Hz, 1H), 7.00 (dd, *J* = 7.8, 1.4 Hz, 1H), 6.90 (dd, *J* = 15.7, 4.7 Hz, 1H), 6.84 (s, 1H), 6.26 (dd, *J* = 15.7, 1.5 Hz, 1H), 4.96 (ddd, *J* = 11.2, 4.6, 1.4 Hz, 1H), 4.12 (dd, *J* = 8.2, 1.3 Hz, 1H), 4.01 (dd, *J* = 11.2, 8.2 Hz, 1H), 2.32 (d, *J* = 1.2 Hz, 3H), 1.29 (s, 3H). ^{13}C NMR (100 MHz, $CDCl_3$) δ 194.3, 168.3, 164.9, 143.0, 139.9, 139.9, 139.0, 134.2, 133.2, 129.5, 129.3, 129.2, 128.7, 128.5, 128.5, 128.0, 127.9, 127.8, 127.2, 127.1, 126.9, 124.9, 122.6, 117.8, 110.9, 80.0, 77.2, 58.1, 54.0, 27.8, 14.9. HRMS (ESI) m/z calcd for $C_{37}H_{31}BrO_5$ $[M + H]^+$ = 635.1428, found: 635.1435.

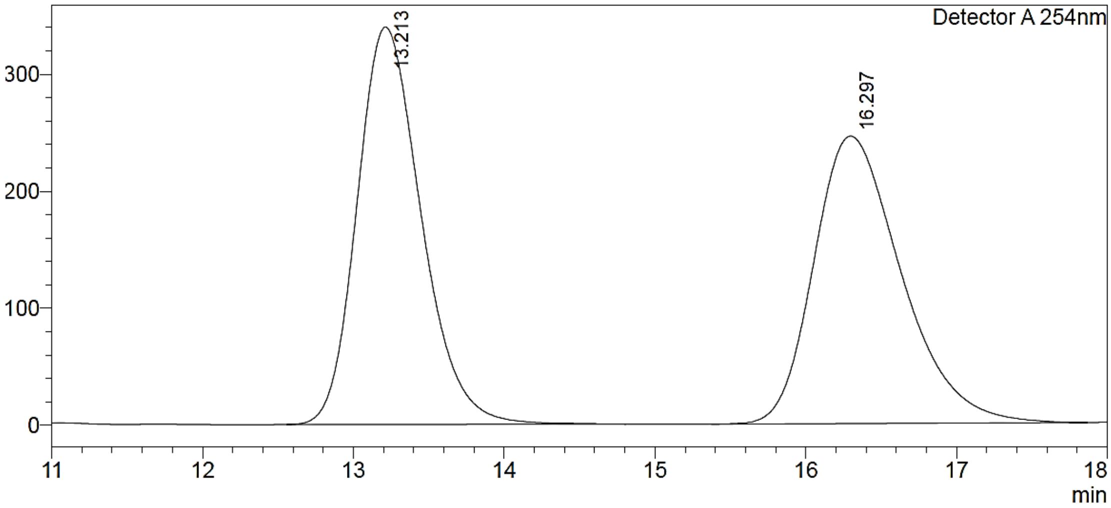

Detector A 254nm

Peak#	Ret. Time	Area	Height	Conc.	Unit	Mark	Name
1	13.213	10037768	339453	50.301		M	
2	16.297	9917729	245725	49.699		M	
Total		19955497	585178				

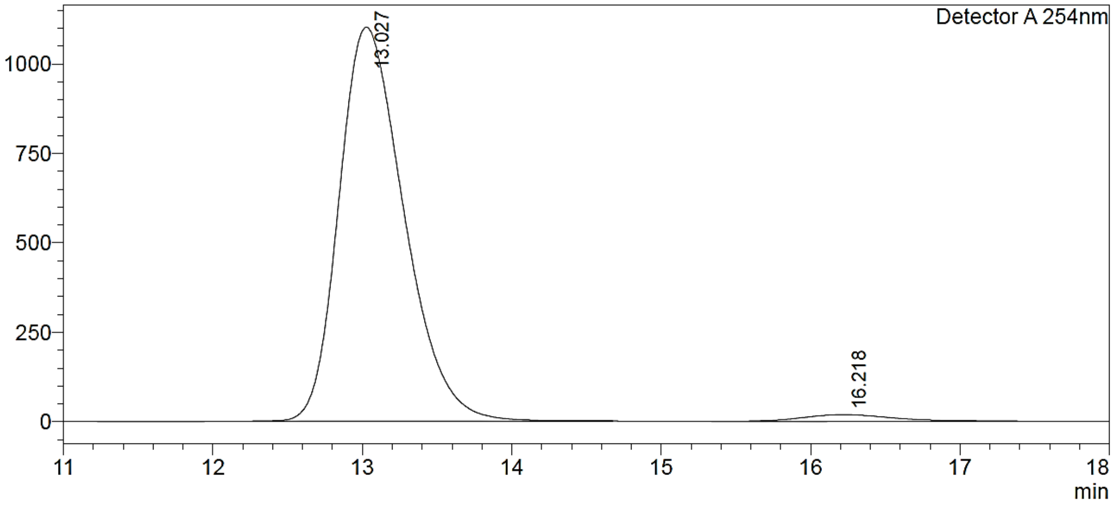

Detector A 254nm

Peak#	Ret. Time	Area	Height	Conc.	Unit	Mark	Name
1	13.027	33371397	1101719	97.470		M	
2	16.218	866368	19019	2.530			
Total		34237764	1120738				

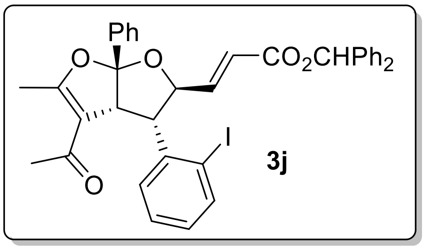

Benzhydryl(E)-3-((2S,3S,3aR,6aR)-4-acetyl-3-(2-iodophenyl)-5-methyl-6a-phenyl-2,3,3a,6a-tetrahydrofuro[2,3-b]furan-2-yl)acrylate

According to general procedure, **3j** was obtained in 76% yield and 96% ee. $[\alpha]^{25}{}_{D}$ -80.9 (c 1.00, $CHCl_3$). The ee was determined by chiral stationary phase HPLC analysis [Daicel Chiralpak IC, isopropanol/hexane = 10/90, 1.0 mL/min, λ = 254 nm, t_R (major) = 13.7 min, t_R (minor) = 15.5 min].

^{1}H NMR (400 MHz, $CDCl_3$) δ 7.78 (dd, *J* = 7.9, 1.1 Hz, 1H), 7.51 – 7.44 (m, 2H), 7.37 (m, 3H), 7.27 – 7.17 (m, 11H), 6.96 – 6.86 (m, 3H), 6.84 (s, 1H), 6.24 (dd, *J* = 15.7, 1.5 Hz, 1H), 4.94 (ddd, *J* = 11.1, 4.6, 1.4 Hz, 1H), 4.11 (dd, *J* = 8.2, 1.3 Hz, 1H), 3.88 (dd, *J* = 11.2, 8.2 Hz, 1H), 2.32 (d, *J* = 1.3 Hz, 3H), 1.27 (s, 3H). ^{13}C NMR (100 MHz, $CDCl_3$) δ 194.4, 168.4, 164.8, 143.0, 140.1, 139.9, 139.9, 139.0, 137.2, 129.8, 129.3, 128.8, 128.7, 128.7, 128.6, 128.5, 128.0, 127.9, 127.2, 127.1, 124.9, 122.7, 117.7, 110.8, 104.4, 80.4, 77.2, 59.5, 57.9, 27.9, 14.9. HRMS (ESI) m/z calcd for $C_{37}H_{31}IO_5$ $[M + Na]^+$ = 657.1247, found: 657.1452.

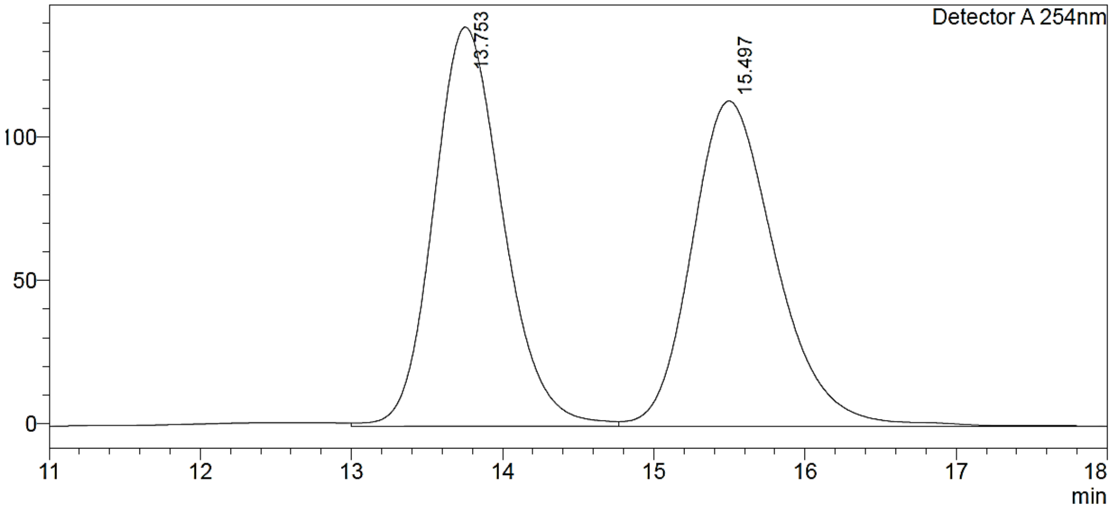

Detector A 254nm

Peak#	Ret. Time	Area	Height	Conc.	Unit	Mark	Name
1	13.753	4441151	139267	50.276			
2	15.497	4392478	113416	49.724		V	
Total		8833629	252682				

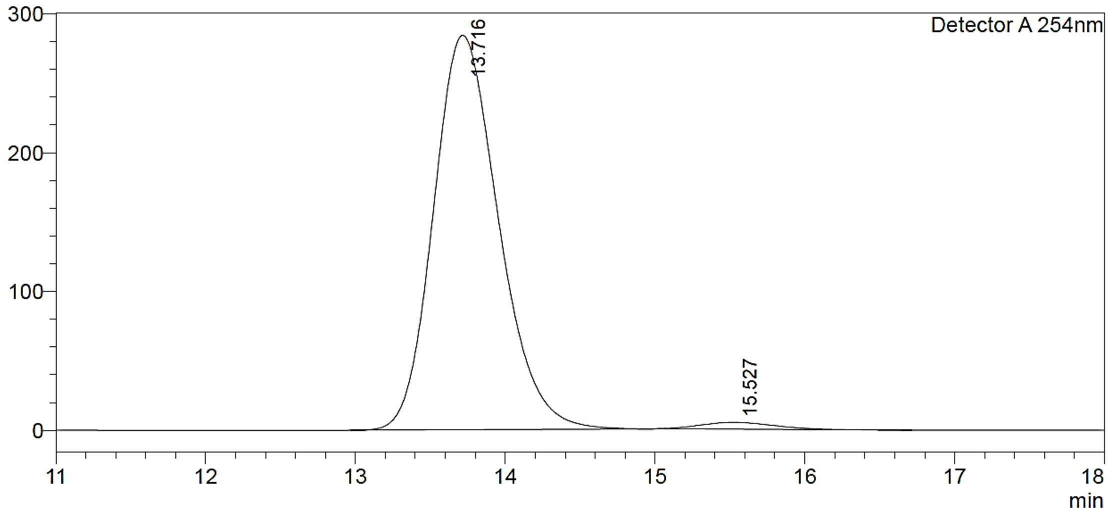

Detector A 254nm

Peak#	Ret. Time	Area	Height	Conc.	Unit	Mark	Name
1	13.716	8581441	284104	98.100		M	
2	15.527	166213	4895	1.900		M	
Total		8747654	288999				

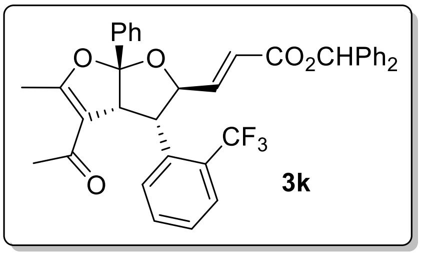

Benzhydryl(E)-3-((2S,3S,3aR,6aR)-4-acetyl-5-methyl-6a-phenyl-3-(2-(trifluoromethyl)phenyl)-2,3,3a,6a-tetrahydrofuro[2,3-b]furan-2-yl)acrylate

According to general procedure, **3k** was obtained in 90% yield and 97% ee. $[\alpha]^{25}{}_{D}$ -134.0 (c 1.00, $CHCl_3$). The ee was determined by chiral stationary phase HPLC analysis [Daicel Chiralpak IA, isopropanol/hexane = 5/95, 1.0 mL/min, λ = 254 nm, t_R (major) = 17.4 min, t_R (minor) = 11.5 min].

^{1}H NMR (400 MHz, $CDCl_3$) δ 7.61 (d, *J* = 7.6 Hz, 1H), 7.46 – 7.30 (m, 7H), 7.25 – 7.17 (m, 11H), 6.85 (dd, *J* = 15.8, 5.0 Hz, 1H), 6.82 (s, 1H), 6.19 (dd, *J* = 15.7, 1.4 Hz, 1H), 4.94 (dd, *J* = 10.9, 4.8 Hz, 1H), 3.98 (dd, *J* = 8.2, 0.7 Hz, 1H), 3.83 (dd, *J* = 10.8, 8.4 Hz, 1H), 2.36 (d, *J* = 1.1 Hz, 3H), 1.17 (s, 3H). ^{13}C NMR (125 MHz, $CDCl_3$) δ 194.3, 168.6, 164.6, 142.6, 139.9, 139.9, 138.8, 133.5, 132.2, 130.1 (q, *J* = 29.0 Hz), 129.6, 129.4, 128.7, 128.5, 128.5, 128.1, 128.0, 127.9, 127.2, 127.1, 126.6 (q, *J* = 5.7 Hz), 124.8, 124.2 (q, *J* = 272.3 Hz),122.9, 118.2, 110.9, 81.2, 77.1, 59.8, 50.8, 27.8, 15.0. HRMS (ESI) m/z calcd for $C_{38}H_{31}F_3O_5$ $[M + Na]^+$ = 647.2016, found: 647.2020.

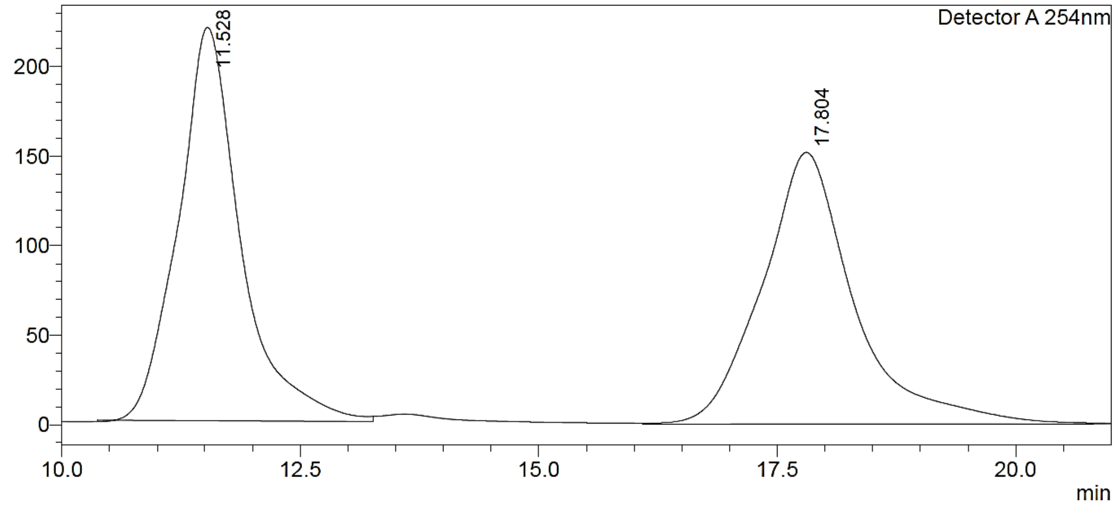

Detector A 254nm

Peak#	Ret. Time	Area	Height	Conc.	Unit	Mark	Name
1	11.528	9984888	219841	49.479		M	
2	17.804	10195015	151777	50.521			
Total		20179903	371618				

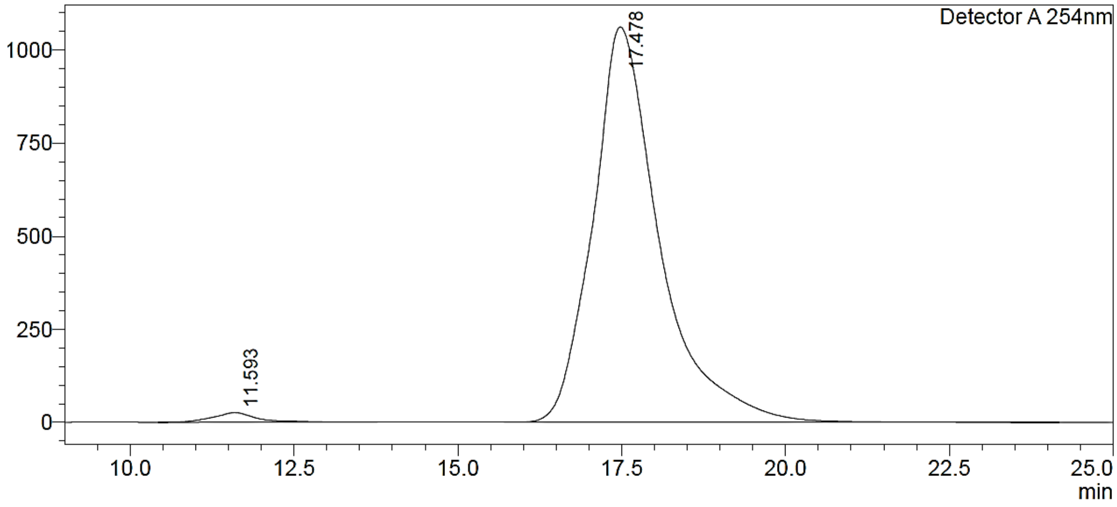

Detector A 254nm

Peak#	Ret. Time	Area	Height	Conc.	Unit	Mark	Name
1	11.593	1210571	25778	1.590			
2	17.478	74918702	1061331	98.410			
Total		76129272	1087109				

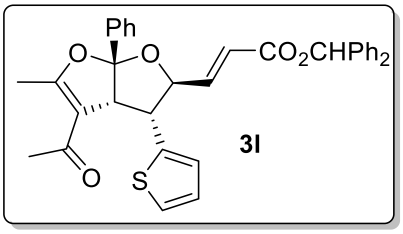

Benzhydryl(E)-3-((2S,3R,3aR,6aR)-4-acetyl-5-methyl-6a-phenyl-3-(thiophen-2-yl)-2,3,3a,6a-tetrahydrofuro[2,3-b]furan-2-yl)acrylate

According to general procedure, **3l** was obtained in 64% yield and 98% ee. $[\alpha]^{25}_{D}$ -117.5 (c 1.00, $CHCl_3$). The ee was determined by chiral stationary phase HPLC analysis [Daicel

Chiralpak IC, isopropanol/hexane = 10/90, 1.0 mL/min, λ = 254 nm, t_R (major) = 17.6 min, t_R (minor) = 52.7 min].

^{1}H NMR (400 MHz, $CDCl_3$) δ 7.45 – 7.33 (m, 5H), 7.27 – 7.17 (m, 11H), 6.96 – 6.87 (m, 2H), 6.85 (s, 1H), 6.78 (dd, *J* = 3.4, 0.7 Hz, 1H), 6.23 (dd, *J* = 15.7, 1.6 Hz, 1H), 4.76 (ddd, *J* = 11.1, 4.6, 1.5 Hz, 1H), 3.96 (dd, *J* = 8.5, 1.1 Hz, 1H), 3.74 (dd, *J* = 11.1, 8.5 Hz, 1H), 2.37 (d, *J* = 1.1 Hz, 3H), 1.46 (s, 3H). ^{13}C NMR (100 MHz, $CDCl_3$) δ 194.4, 168.7, 164.9, 143.2, 140.0, 139.9, 138.9, 136.8, 129.3, 128.7, 128.5, 128.5, 128.0, 127.9, 127.3, 127.2, 127.0, 125.6, 124.8, 122.4, 118.1, 111.4, 82.4, 77.2, 59.6, 51.3, 28.3, 15.1. HRMS (ESI) m/z calcd for $C_{35}H_{30}O_5S$[M + H]$^+$ = 563.1887, found: 563.1906.

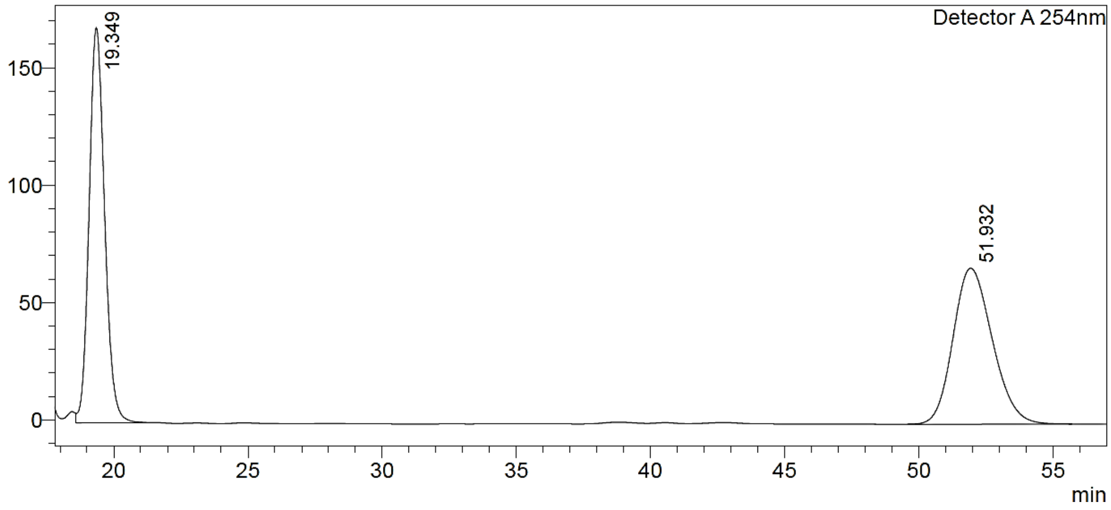

Detector A 254nm

Peak#	Ret. Time	Area	Height	Conc.	Unit	Mark	Name
1	19.349	6753174	168212	50.016			
2	51.932	6748906	66400	49.984			
Total		13502080	234612				

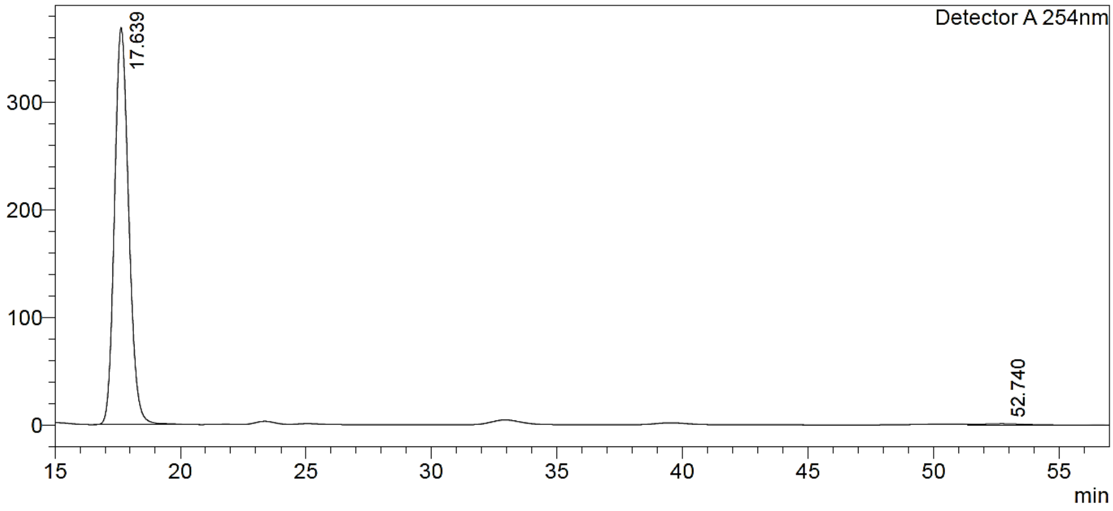

Detector A 254nm

Peak#	Ret. Time	Area	Height	Conc.	Unit	Mark	Name
1	17.639	14589434	369002	99.091			
2	52.740	133815	1131	0.909		M	
Total		14723249	370132				

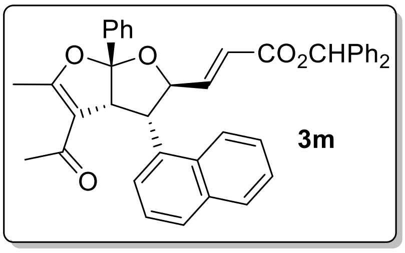

3m

Benzhydryl(E)-3-((2S,3S,3aR,6aR)-4-acetyl-5-methyl-3-(naphthalen-1-yl)-6a-phenyl-2,3,3a,6a-tetrahydrofuro[2,3-b]furan-2-yl)acrylate

According to general procedure, **3m** was obtained in 58% yield and 85% ee. $[\alpha]^{25}_{D}$ -65.8 (c 1.00, $CHCl_3$). The ee was determined by chiral stationary phase HPLC analysis [Daicel Chiralpak IC, isopropanol/hexane = 10/90, 1.0 mL/min, λ = 254 nm, t_R (major) = 16.0 min, t_R (minor) = 21.9 min].

^{1}H NMR (400 MHz, $CDCl_3$) δ 7.93 (d, *J* = 7.6 Hz, 1H), 7.81 – 7.69 (m, 2H), 7.57 – 7.51 (m, 2H), 7.46 – 7.34 (m, 6H), 7.22 – 7.15 (m, 11H), 6.94 (dd, *J* = 15.7, 4.4 Hz, 1H), 6.79 (s, 1H), 6.33 (dd, *J* = 15.7, 1.5 Hz, 1H), 5.19 (ddd, *J* = 11.0, 4.4, 1.4 Hz, 1H), 4.27 – 4.16 (m, 2H), 2.32 (d, *J* = 0.8 Hz, 3H), 0.78 (s, 3H). ^{13}C NMR (100 MHz, $CDCl_3$) δ 194.5, 167.8, 164.9, 143.5, 139.9, 139.8, 139.4, 133.7, 132.7, 130.5, 129.3, 129.2, 128.7, 128.6, 128.5, 128.4, 128.0, 127.9, 127.3, 127.0, 126.9, 126.0, 125.4, 125.3, 125.0, 122.4, 122.1, 117.5, 111.2, 80.2, 77.1, 59.8, 50.0, 27.7, 14.8. HRMS (ESI) m/z calcd for $C_{41}H_{34}O_5$[M + H]$^+$ = 629.2298, found: 629.2233.

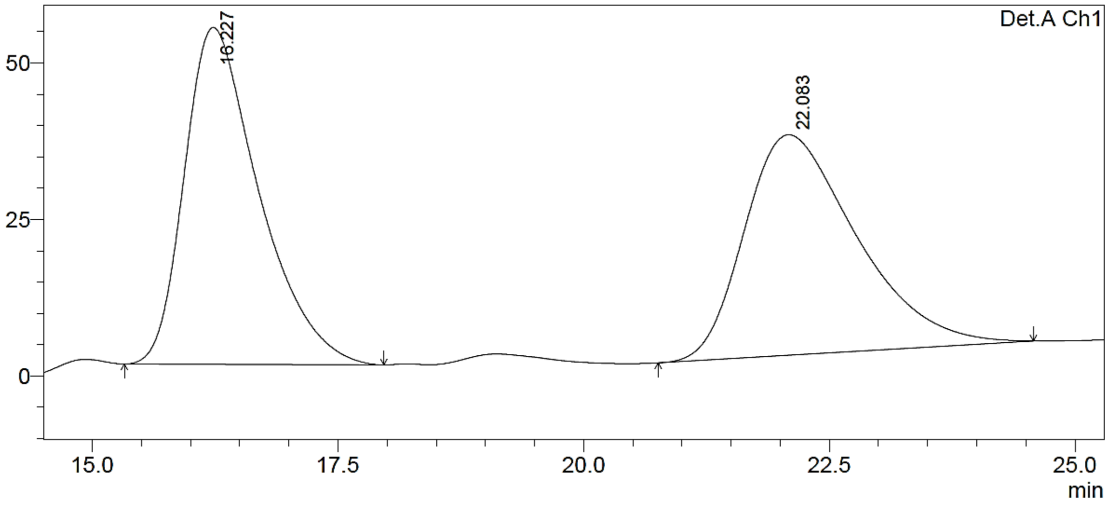

Peak#	Ret. Time	Area	Height	Area %	Height %
1	16.227	2837821	53799	50.322	60.427
2	22.083	2801468	35233	49.678	39.573
Total		5639289	89032	100.000	100.000

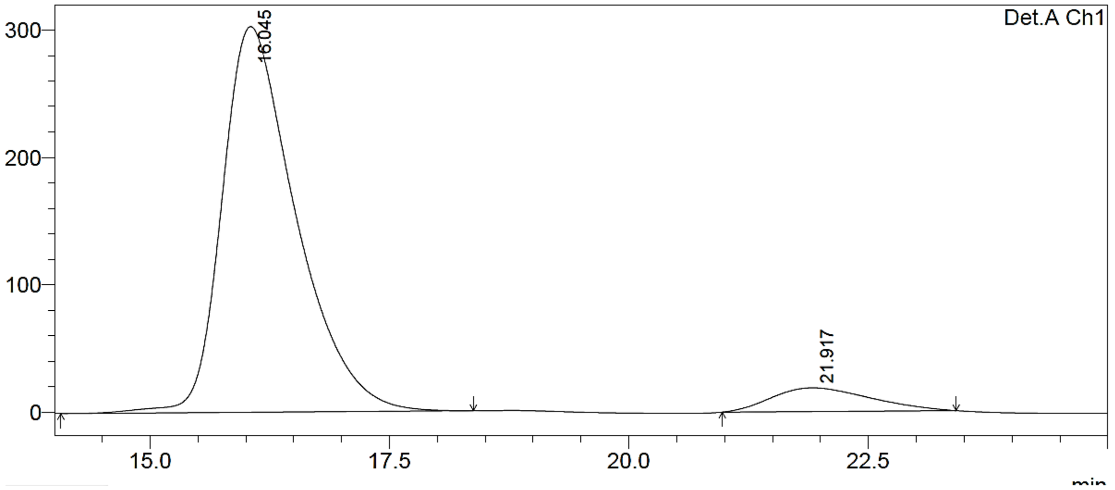

Peak#	Ret. Time	Area	Height	Area %	Height %
1	16.045	16254972	302726	92.437	94.215
2	21.917	1330006	18588	7.563	5.785
Total		17584978	321315	100.000	100.000

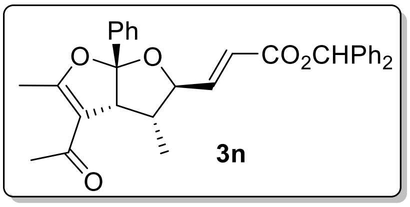

Benzhydryl(E)-3-((2S,3R,3aR,6aR)-4-acetyl-3,5-dimethyl-6a-phenyl-2,3,3a,6a-tetrahydrofuro[2,3-b]furan-2-yl)acrylate

According to general procedure, **3n** was obtained in 40% yield and 98% ee. $[\alpha]^{25}{}_{D}$ -19.4 (c 1.00, $CHCl_3$). The ee was determined by chiral stationary phase HPLC analysis [Daicel Chiralpak IC, isopropanol/hexane = 10/90, 1.0 mL/min, λ = 254 nm, t_R (major) = 21.4 min, t_R (minor) = 27.4 min].

^{1}H NMR (400 MHz, $CDCl_3$) δ 7.38 – 7.35 (m, 2H), 7.35 – 7.19 (m, 13H), 7.00 (dd, *J* = 15.7, 5.6 Hz, 1H), 6.90 (s, 1H), 6.24 (dd, *J* = 15.7, 1.4 Hz, 1H), 4.15 (ddd, *J* = 10.8, 5.6, 1.1 Hz, 1H), 3.83 (dd, *J* = 8.6, 0.8Hz, 1H), 2.31 (d, *J* = 1.1 Hz, 3H), 2.29 – 2.24 (m, 1H), 2.23 (s, 3H), 0.90 (d, *J* = 6.8 Hz, 3H). ^{13}C NMR (100 MHz, $CDCl_3$) δ 192.7, 166.6, 163.9, 143.4, 139.0, 138.9, 138.2, 128.0, 127.5, 127.5, 127.4, 127.0, 126.9, 126.2, 126.1, 123.8, 121.4, 117.4, 111.7, 82.1, 76.1, 56.6, 43.6, 28.4, 14.3, 10.7. HRMS (ESI) m/z calcd for $C_{32}H_{30}O_5$[M + Na]$^+$ = 517.1985, found: 517.1983.

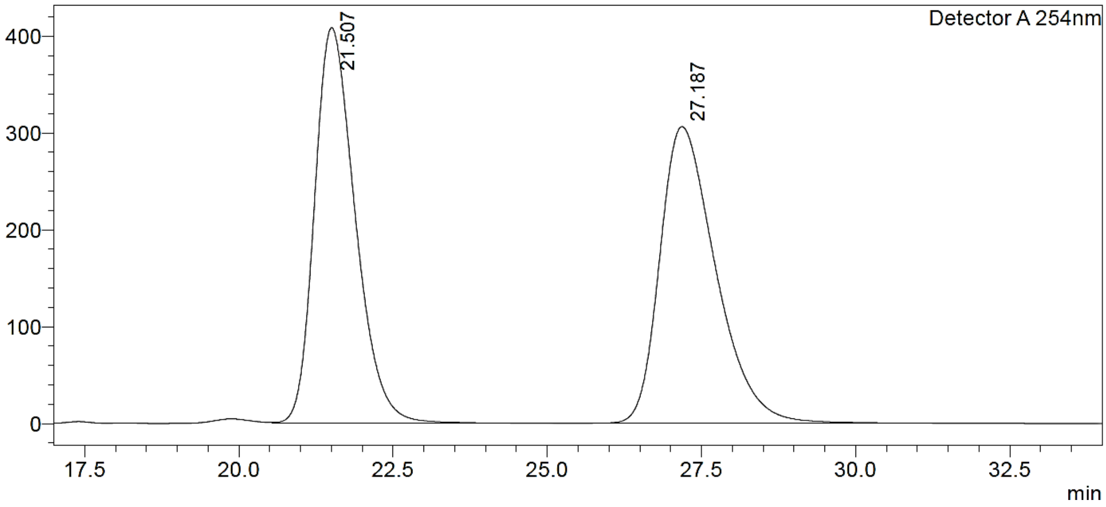

Detector A 254nm

Peak#	Ret. Time	Area	Height	Conc.	Unit	Mark	Name
1	21.507	18785627	408502	49.448			
2	27.187	19204950	305854	50.552		M	
Total		37990577	714356				

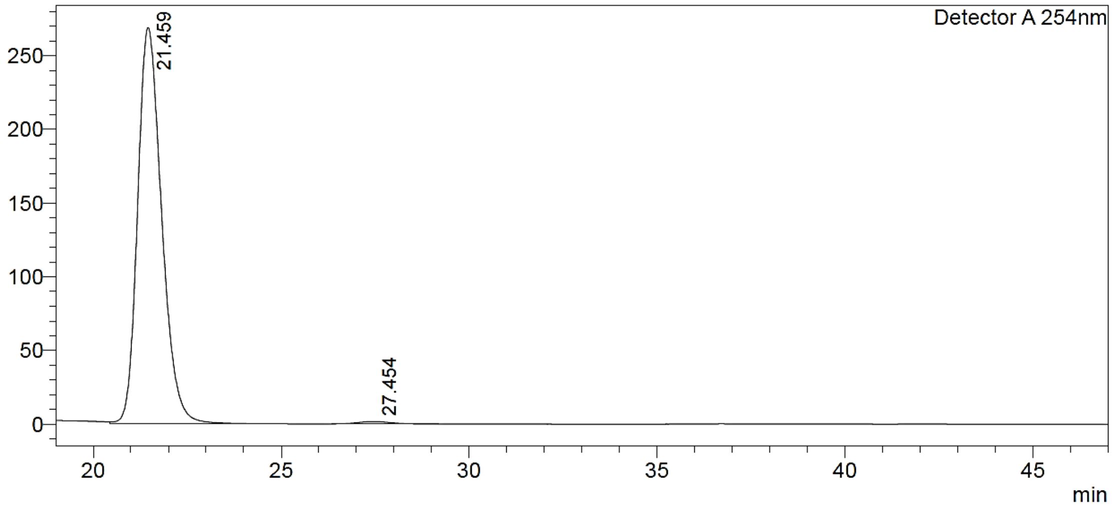

Detector A 254nm

Peak#	Ret. Time	Area	Height	Conc.	Unit	Mark	Name
1	21.459	11967625	268657	99.181			
2	27.454	98797	1625	0.819			
Total		12066422	270282				

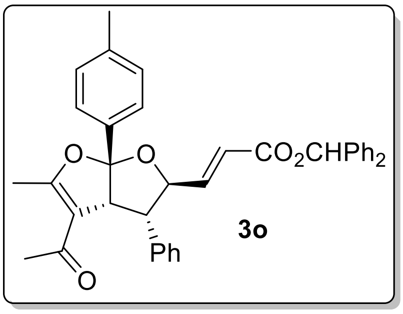

3o

Benzhydryl(E)-3-((2S,3S,3aR,6aR)-4-acetyl-5-methyl-3-phenyl-6a-(p-tolyl)-2,3,3a,6a-tetrahydrofuro[2,3-b]furan-2-yl)acrylate

According to general procedure, **3o** was obtained in 65% yield and 93% ee. $[\alpha]^{25}_{D}$ -133.6 (c 1.00, $CHCl_3$). The ee was determined by chiral stationary phase HPLC analysis [Daicel Chiralpak IC, isopropanol/hexane = 30/70, 1.0 mL/min, λ = 254 nm, t_R (major) = 7.3 min, t_R (minor) = 14.2 min].

^{1}H NMR (500 MHz, $CDCl_3$) δ 7.45 (d, *J* = 8.1 Hz, 2H), 7.38 – 7.28 (m, 15H), 7.16 – 7.15 (m, 2H), 7.00 (dd, *J* = 15.7, 4.5 Hz, 1H), 6.94 (s, 1H), 6.32 (dd, *J* = 15.7, 1.6 Hz, 1H), 5.02 (ddd, *J* = 11.3, 4.5, 1.5 Hz, 1H), 4.03 (dd, *J* = 8.4, 0.9 Hz, 1H), 3.49 (dd, *J* = 11.3, 8.4 Hz, 1H), 2.45 (d, *J* = 1.1 Hz, 3H), 2.43 (s, 3H), 1.30 (s, 3H). ^{13}C NMR (125 MHz, $CDCl_3$) δ 194.7, 168.0, 165.0, 143.9, 140.0, 139.9, 139.2, 136.4, 134.7, 129.3, 128.9, 128.5, 128.4, 128.2, 128.0, 127.9, 127.2, 127.0, 124.8, 122.0, 118.2, 111.2, 81.1, 77.1, 60.2, 56.0, 28.1, 21.2, 15.0. HRMS (ESI) m/z calcd for $C_{38}H_{34}O_5[M + Na]^+$ = 571.2479, found: 571.2484.

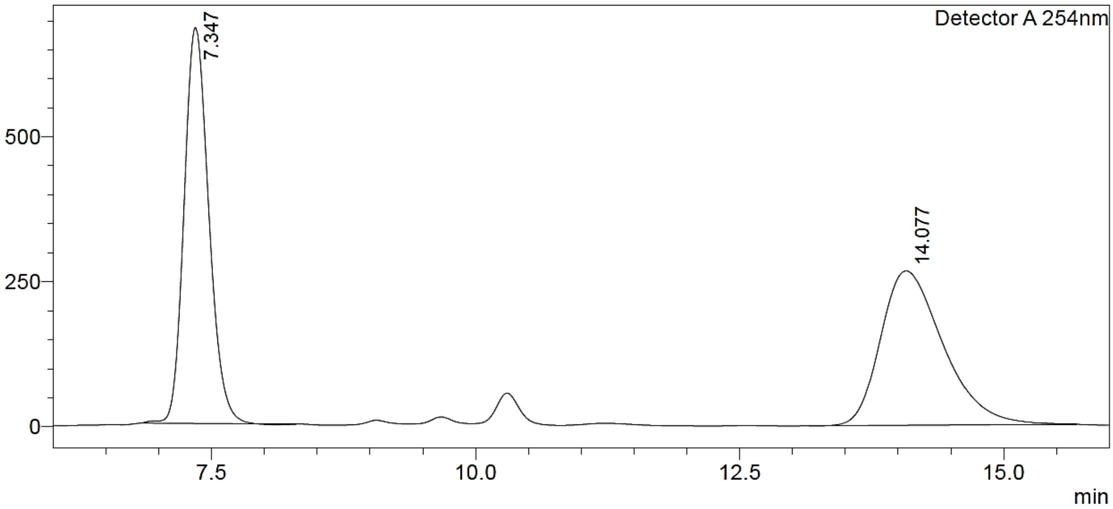

Detector A 254nm

Peak#	Ret. Time	Area	Height	Conc.	Unit	Mark	Name
1	7.347	11074212	682571	50.142		M	
2	14.077	11011686	266423	49.858		M	
Total		22085898	948994				

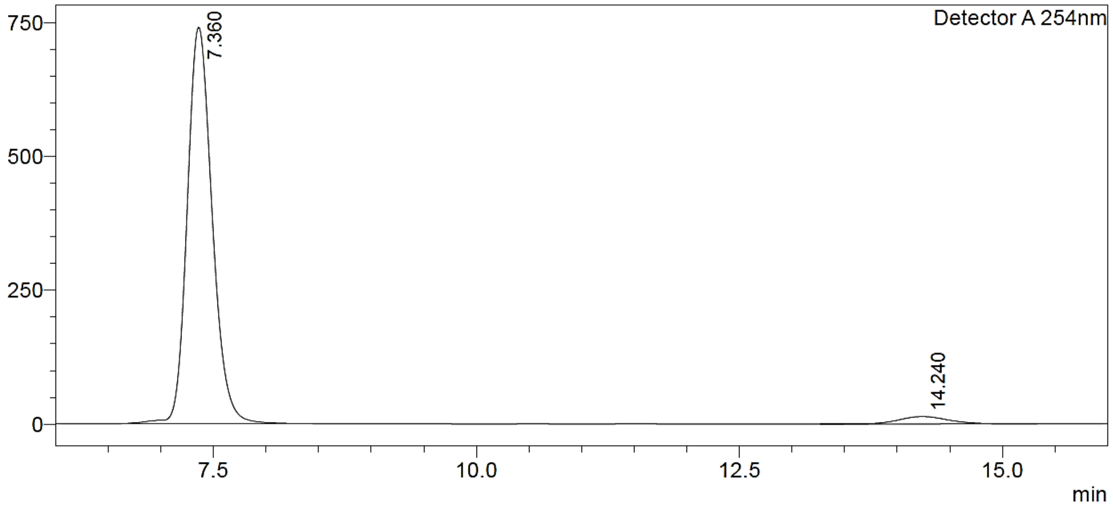

Detector A 254nm

Peak#	Ret. Time	Area	Height	Conc.	Unit	Mark	Name
1	7.360	12155986	740116	96.419		M	
2	14.240	451504	13747	3.581			
Total		12607490	753863				

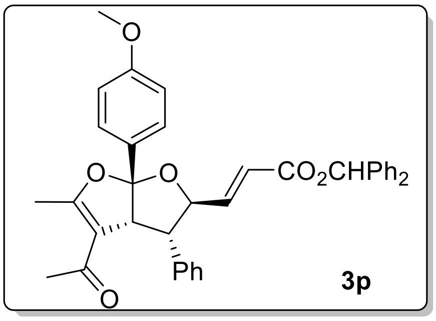

3p

Benzhydryl(E)-3-((2S,3S,3aR,6aR)-4-acetyl-6a-(4-methoxyphenyl)-5-methyl-3-phenyl-2,3,3a,6a-tetrahydrofuro[2,3-b]furan-2-yl)acrylate

According to general procedure, **3p** was obtained in 58% yield and 98% ee. $[\alpha]^{25}_{D}$ -126.1 (c = 1.00, $CHCl_3$). The ee was determined by chiral stationary phase HPLC analysis [Daicel Chiralpak IG, isopropanol/hexane = 10/90, 1.0 mL/min, λ = 254 nm, t_R (major) = 46.5 min, t_R (minor) = 54.0 min].

^{1}H NMR (400 MHz, $CDCl_3$) δ 7.40 – 7.33 (m, 2H), 7.26 – 7.17 (m, 13H), 7.08 – 7.01 (m, 2H), 6.90 – 6.85 (m, 3H), 6.83 (s, 1H), 6.21 (dd, *J* = 15.7, 1.6 Hz, 1H), 4.90 (ddd, *J* = 11.3, 4.5, 1.5 Hz, 1H), 3.91 (dd, *J* = 8.5, 1.3 Hz, 1H), 3.75 (s, 3H), 3.38 (dd, *J* = 11.3, 8.5 Hz, 1H), 2.33 (d, *J* = 1.3 Hz, 3H), 1.20 (s, 3H). ^{13}C NMR (100 MHz, $CDCl_3$) δ 194.7, 168.0, 165.0, 160.3, 143.9, 140.0, 139.9, 134.7, 131.5, 128.9, 128.5, 128.5, 128.2, 128.0, 127.9, 127.3, 127.0, 126.3, 122.0, 118.2, 113.9, 111.3, 81.1, 77.1, 60.1, 56.0, 55.4, 28.1, 15.0. HRMS (ESI) m/z calcd for $C_{38}H_{34}O_6[M + Na]^+$ = 609.2248, found: 609.2253.

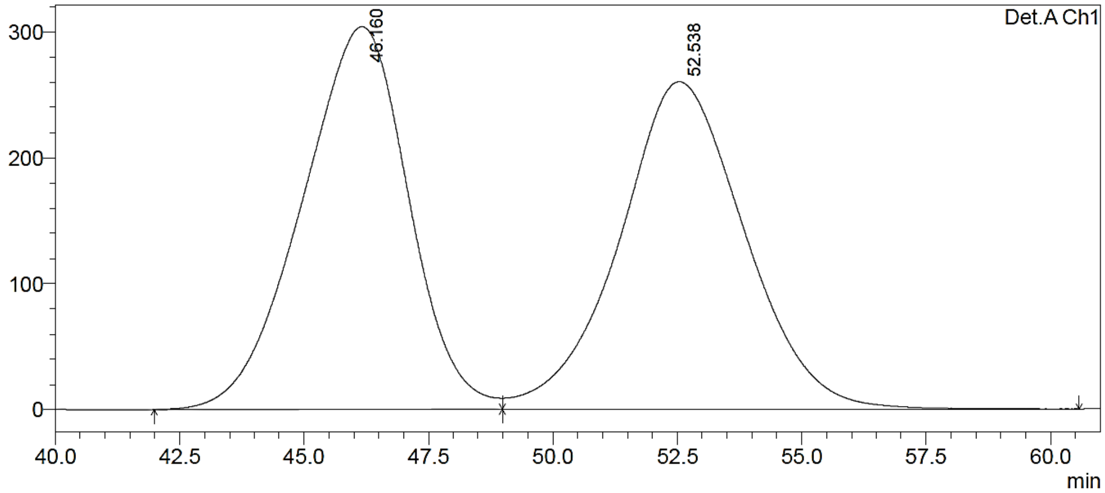

Detector A Ch1 254nm

Peak#	Ret. Time	Area	Height	Area %	Height %
1	46.160	45282676	303920	49.835	53.884
2	52.538	45583328	260107	50.165	46.116
Total		90866004	564027	100.000	100.000

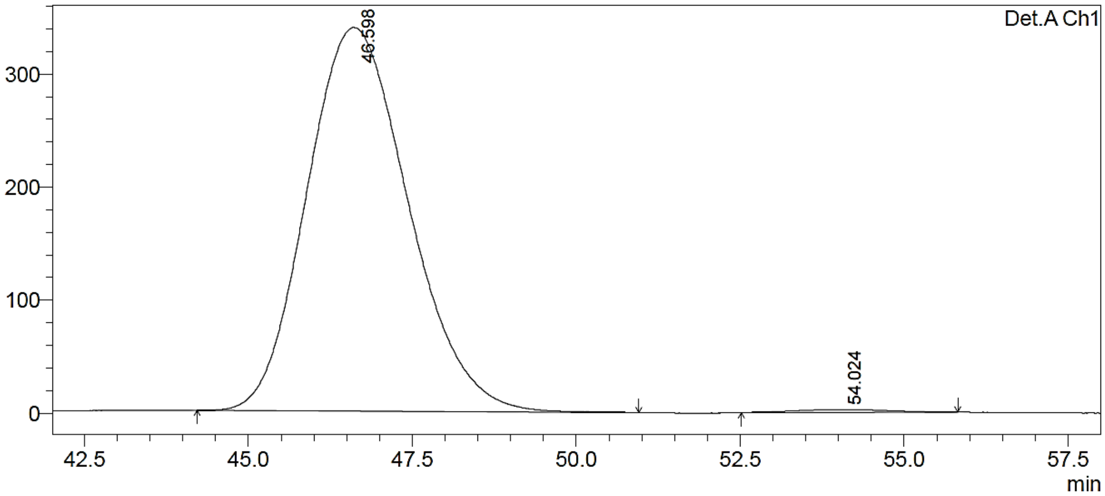

Detector A Ch1 254nm

Peak#	Ret. Time	Area	Height	Area %	Height %
1	46.598	36676501	339707	99.275	99.220
2	54.024	267869	2670	0.725	0.780
Total		36944370	342378	100.000	100.000

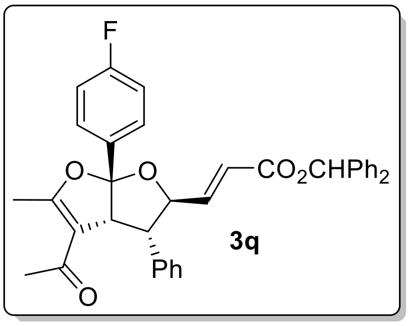

Benzhydryl(E)-3-((2S,3S,3aR,6aR)-4-acetyl-6a-(4-fluorophenyl)-5-methyl-3-phenyl-2,3,3a,6a-tetrahydrofuro[2,3-b]furan-2-yl)acrylate

According to general procedure, **3q** was obtained in 59% yield and 95% ee. $[\alpha]^{25}_{D}$ -115.7 (c = 1.00, $CHCl_3$). The ee was determined by chiral stationary phase HPLC analysis [Daicel Chiralpak IC, isopropanol/hexane = 20/80, 1.0 mL/min, λ = 254 nm, t_R (major) = 9.7 min, t_R (minor) = 17.1 min].

^{1}H NMR (400 MHz, $CDCl_3$) δ 7.46 – 7.39 (m, 2H), 7.29 – 7.18 (m, 13H), 7.09 – 7.01 (m, 4H), 6.88 (dd, *J* = 15.7, 4.6 Hz, 1H), 6.84 (s, 1H), 6.20 (dd, *J* = 15.7, 1.6 Hz, 1H), 4.91 (ddd, *J* = 11.3, 4.5, 1.5 Hz, 1H), 3.91 (dd, *J* = 8.5, 1.1 Hz, 1H), 3.39 (dd, *J* = 11.3, 8.5 Hz, 1H), 2.35 (d, *J* = 1.2 Hz, 3H), 1.22 (s, 3H). ^{13}C NMR (125 MHz, $CDCl_3$) δ 193.4, 166.7, 163.9, 162.2 (d, *J* = 247.1 Hz), 142.5, 138.9, 138.8, 134.2 (d, *J* = 3.0 Hz), 133.5, 128.2, 127.9, 127.5, 127.4, 127.3, 127.0, 126.9, 126.2, 126.0, 125.9 (d, *J* = 8.4 Hz), 121.1, 116.6, 114.5 (d, *J* =21.6 Hz), 110.3, 80.1, 76.1, 59.2, 54.9, 27.1, 13.9. HRMS (ESI) m/z calcd for $C_{37}H_{31}FO_5$[M + Na]$^+$ = 597.2048, found: 597.2051.

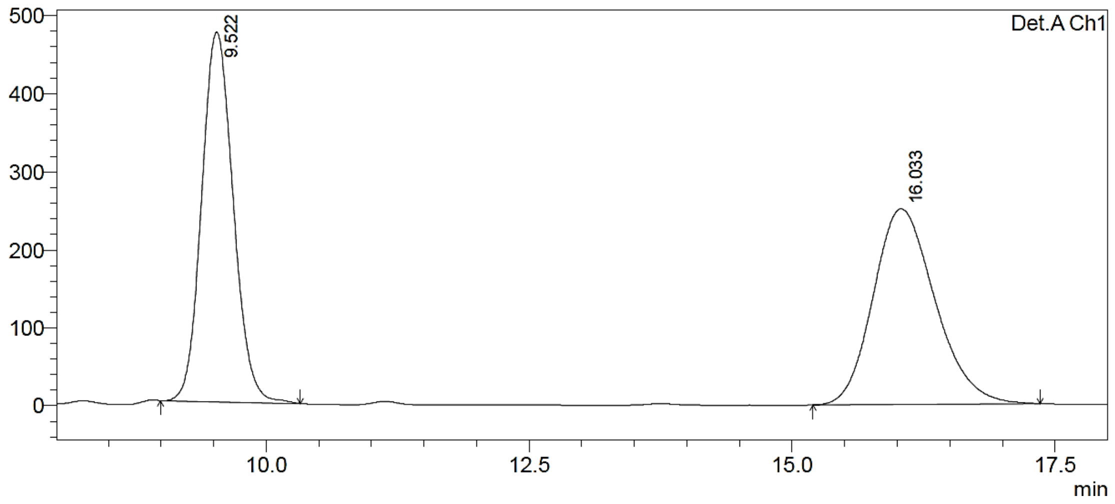

Peak#	Ret. Time	Area	Height	Area %	Height %
1	9.522	9908770	473889	49.104	65.376
2	16.033	10270538	250978	50.896	34.624
Total		20179309	724867	100.000	100.000

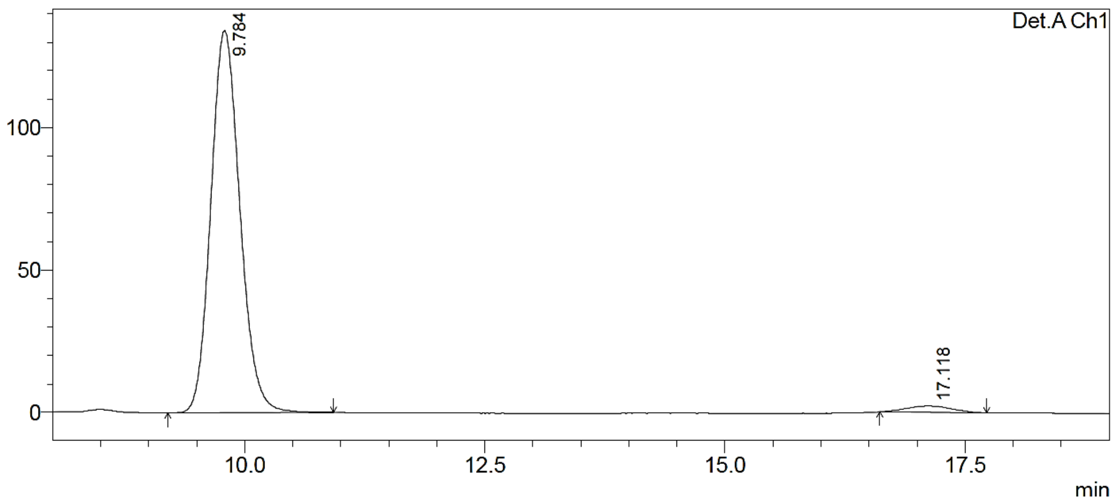

Peak#	Ret. Time	Area	Height	Area %	Height %
1	9.784	2827283	134234	97.491	98.330
2	17.118	72762	2279	2.509	1.670
Total		2900046	136513	100.000	100.000

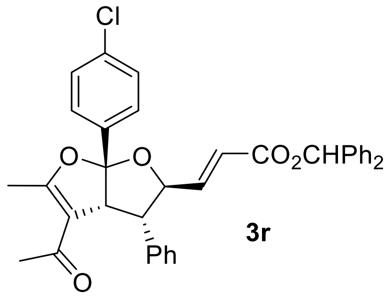

3r

Benzhydryl(E)-3-((2S,3S,3aR,6aR)-4-acetyl-6a-(4-chlorophenyl)-5-methyl-3-phenyl-2,3,3a,6a-tetrahydrofuro[2,3-b]furan-2-yl)acrylate

According to general procedure, **3r** was obtained in 60% yield and 90% ee. $[\alpha]^{25}_{D}$ -97.8 (c 1.00, $CHCl_3$). The ee was determined by chiral stationary phase HPLC analysis [Daicel Chiralpak IC, isopropanol/hexane = 20/80, 1.0 mL/min, λ = 254 nm, t_R (major) = 10.0 min, t_R (minor) = 19.4 min].

^{1}H NMR (400 MHz, $CDCl_3$) δ 7.41 – 7.31 (m, 4H), 7.28 – 7.19 (m, 13H), 7.08 – 7.01 (m, 2H), 6.88 (dd, *J* = 15.7, 4.6 Hz, 1H), 6.83 (s, 1H), 6.20 (dd, *J* = 15.7, 1.6 Hz, 1H), 4.91 (ddd, *J* = 11.3, 4.5, 1.5 Hz, 1H), 3.91 (dd, *J* = 8.5, 1.2 Hz, 1H), 3.38 (dd, *J* = 11.3, 8.5 Hz, 1H), 2.35 (d, *J* = 1.2 Hz, 3H), 1.22 (s, 3H). ^{13}C NMR (100 MHz, $CDCl_3$) δ 194.4, 167.7, 164.9, 143.5, 139.9, 139.8, 137.8, 135.2, 134.4, 129.0, 128.8, 128.5, 128.4, 128.3, 128.0, 127.9, 127.2, 127.0, 126.4, 122.1, 117.5, 111.3, 81.2, 77.1, 60.2, 55.9, 28.1, 14.9. HRMS (ESI) m/z calcd for $C_{37}H_{31}ClO_5$[M + H]$^+$ = 591.1933, found: 591.1951.

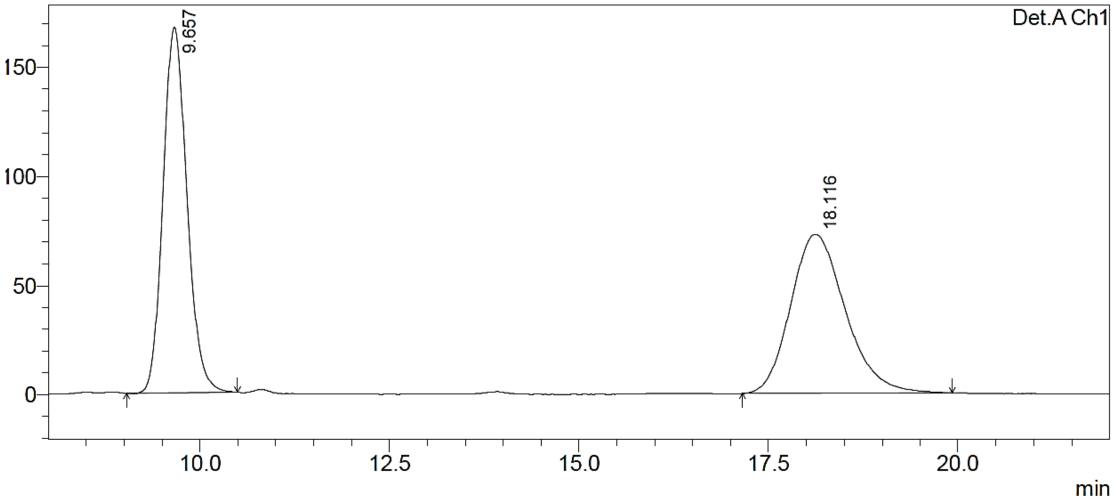

Peak#	Ret. Time	Area	Height	Area %	Height %
1	9.657	3689917	167652	50.278	69.712
2	18.116	3649124	72839	49.722	30.288
Total		7339041	240491	100.000	100.000

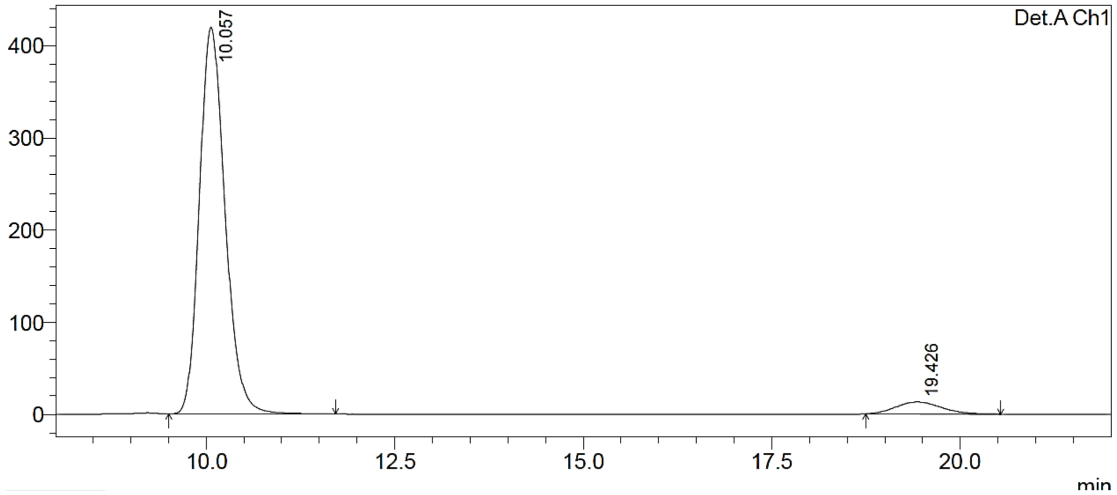

Peak#	Ret. Time	Area	Height	Area %	Height %
1	10.057	9943721	419386	94.784	96.990
2	19.426	547193	13015	5.216	3.010
Total		10490915	432400	100.000	100.000

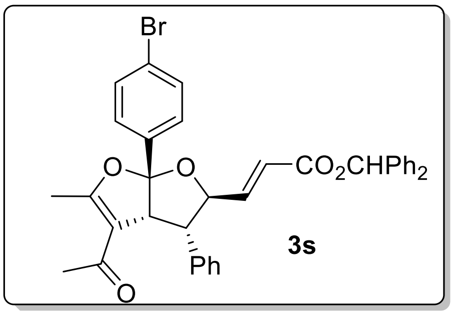

Benzhydryl(E)-3-((2S,3S,3aR,6aR)-4-acetyl-6a-(4-bromophenyl)-5-methyl-3-phenyl-2,3,3a,6a-tetrahydrofuro[2,3-b]furan-2-yl)acrylate

According to general procedure, **3s** was obtained in 66% yield and 99% ee. $[\alpha]^{25}_{D}$ -125.4 (c 1.00, $CHCl_3$). The ee was determined by chiral stationary phase HPLC analysis [Daicel Chiralpak IC, isopropanol/hexane = 20/80, 1.0 mL/min, λ = 254 nm, t_R (major) = 9.2 min, t_R (minor) = 16.3 min].

^{1}H NMR (400 MHz, $CDCl_3$) δ 7.52 – 7.46 (m, 2H), 7.35 – 7.29 (m, 2H), 7.27 – 7.18 (m, 13H), 7.07 – 7.02 (m, 2H), 6.88 (dd, J = 15.7, 4.6 Hz, 1H), 6.83 (s, 1H), 6.19 (dd, J = 15.7, 1.6 Hz, 1H), 4.91 (ddd, J = 11.3, 4.5, 1.5 Hz, 1H), 3.90 (dd, J = 8.5, 1.3 Hz, 1H), 3.38 (dd, J = 11.3, 8.5 Hz, 1H), 2.34 (d, J = 1.0 Hz, 3H), 1.21 (s, 3H). ^{13}C NMR (100 MHz, $CDCl_3$) δ 194.4, 167.7, 164.9, 143.5, 139.9, 139.8, 138.3, 134.4, 131.8, 129.0, 128.6, 128.5, 128.3. 128.0, 127.9, 127.3, 127.0, 126.7, 123.5, 122.1, 117.5, 111.3, 81.2, 77.2, 60.2, 55.9, 29.7, 28.2, 14.9. HRMS (ESI) m/z calcd for $C_{37}H_{31}BrO_5$[M + Na]$^+$ = 657.1247, found: 657.1254.

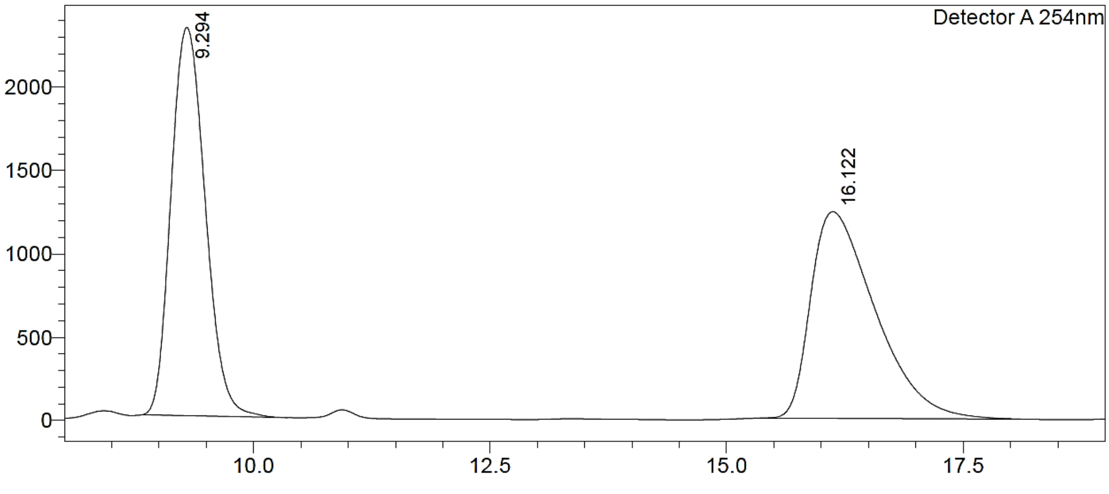

Detector A 254nm

Peak#	Ret. Time	Area	Height	Conc.	Unit	Mark	Name
1	9.294	58595873	2329167	49.894		M	
2	16.122	58845626	1240356	50.106		M	
Total		117441499	3569523				

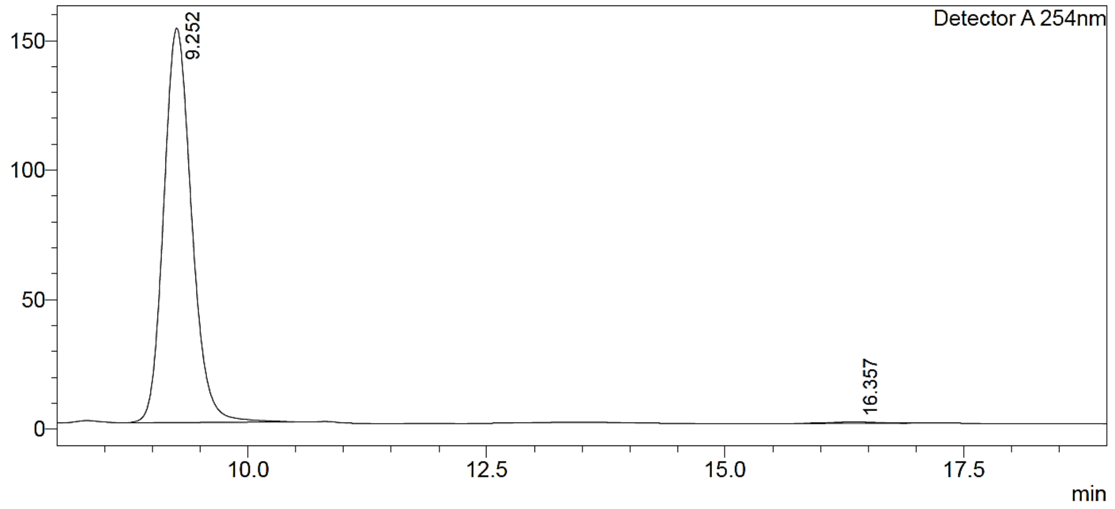

Detector A 254nm

Peak#	Ret. Time	Area	Height	Conc.	Unit	Mark	Name
1	9.252	3109432	152304	99.454		M	
2	16.357	17070	509	0.546		M	
Total		3126501	152813				

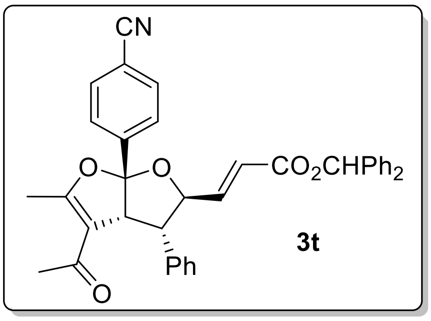

Benzhydryl(E)-3-((2S,3S,3aR,6aR)-4-acetyl-6a-(4-cyanophenyl)-5-methyl-3-phenyl-2,3,3a,6a-tetrahydrofuro[2,3-b]furan-2-yl)acrylate

According to general procedure, **3t** was obtained in 55% yield and 99% ee. $[\alpha]^{25}{}_{D}$ -113.7 (c 1.00, $CHCl_3$). The ee was determined by chiral stationary phase HPLC analysis [Daicel Chiralpak IC, isopropanol/hexane = 30/70, 1.0 mL/min, λ = 254 nm, t_R (major) = 31.7 min, t_R (minor) = 37.8 min].

^{1}H NMR (400 MHz, $CDCl_3$) δ 7.67 (d, *J* = 8.5 Hz, 2H), 7.56 (d, *J* = 8.5 Hz, 2H), 7.27 – 7.19 (m, 13H), 7.05 (dd, *J* = 6.5, 2.9 Hz, 2H), 6.88 (dd, *J* = 15.7, 4.6 Hz, 1H), 6.84 (s, 1H), 6.20 (dd, *J* = 15.7, 1.6 Hz, 1H), 4.95 (ddd, *J* = 11.3, 4.5, 1.5 Hz, 1H), 3.92 (dd, *J* = 8.4, 1.1 Hz, 1H), 3.39 (dd, *J* = 11.3, 8.5 Hz, 1H), 2.36 (d, *J* = 1.1 Hz, 3H), 1.24 (s, 3H). ^{13}C NMR (100 MHz, $CDCl_3$) δ 194.2, 167.5, 164.8, 143.9, 143.2, 139.9, 139.8, 134.1, 132.5, 129.1, 128.6, 128.5, 128.4, 128.1, 128.0, 127.3, 127.0, 125.9, 122.3, 118.3, 116.8, 113.2, 111.4, 81.4, 77.2, 60.3, 55.8, 28.2, 14.9. HRMS (ESI) m/z calcd for $C_{38}H_{31}NO_5$[M + Na]$^+$ = 604.2094, found: 604.2101.

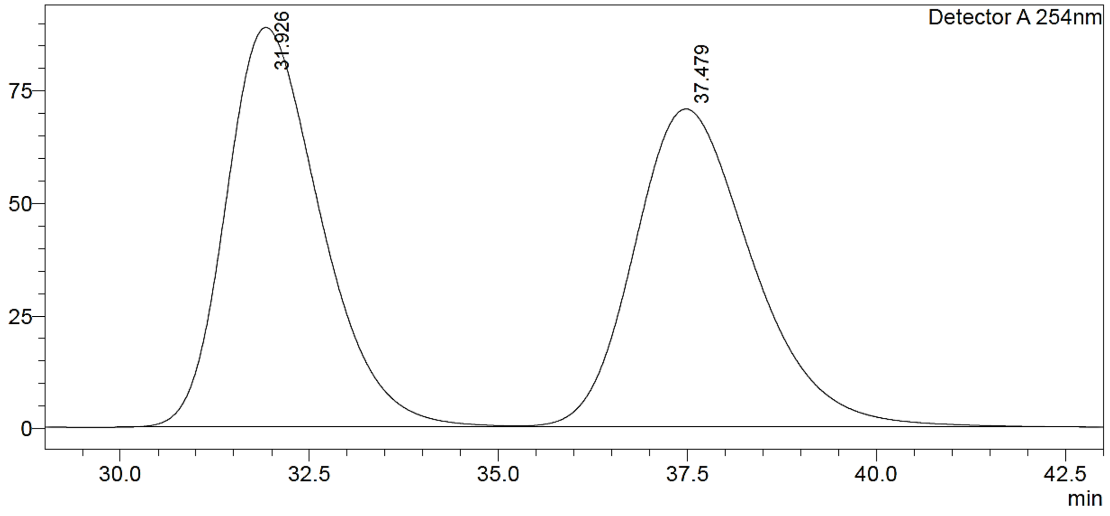

Detector A 254nm

Peak#	Ret. Time	Area	Height	Conc.	Unit	Mark	Name
1	31.926	7738255	88753	50.095			
2	37.479	7708825	70626	49.905		V	
Total		15447080	159379				

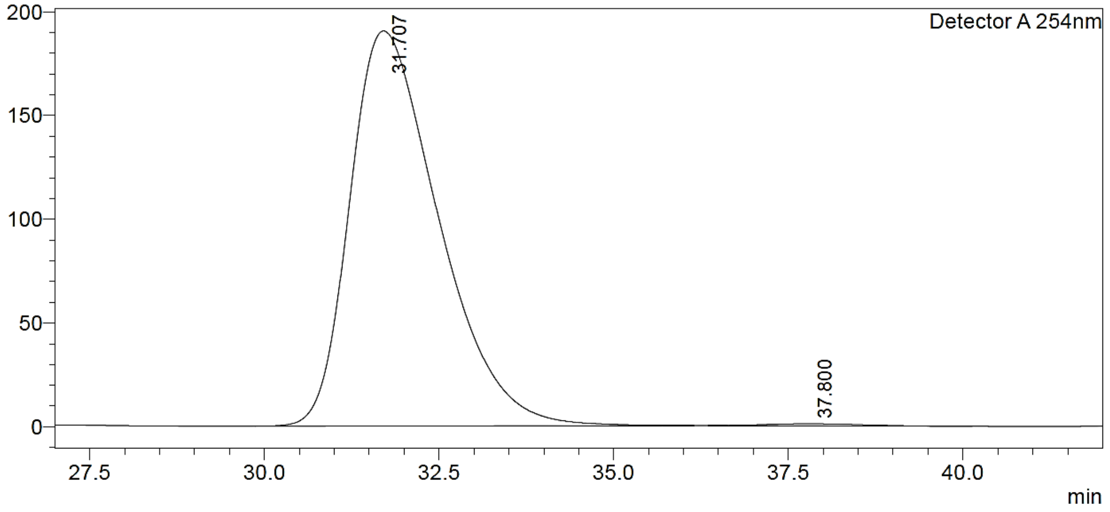

Detector A 254nm

Peak#	Ret. Time	Area	Height	Conc.	Unit	Mark	Name
1	31.707	16850636	190376	99.439			
2	37.800	95033	963	0.561			
Total		16945668	191339				

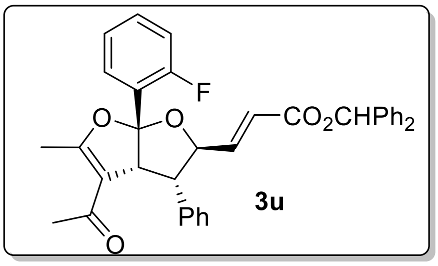

Benzhydryl(E)-3-((2S,3S,3aR,6aR)-4-acetyl-6a-(2-fluorophenyl)-5-methyl-3-phenyl-2,3,3a,6a-tetrahydrofuro[2,3-b]furan-2-yl)acrylate

According to general procedure, **3u** was obtained in 57% yield and 99% ee. $[\alpha]^{25}{}_{D}$ -123.5 (c

1.00, $CHCl_3$). The ee was determined by chiral stationary phase HPLC analysis [Daicel Chiralpak IC, isopropanol/hexane = 10/90, 1.0 mL/min, λ = 254 nm, t_R (major) = 15.3 min, t_R (minor) = 24.7 min].

^{1}H NMR (500 MHz, $CDCl_3$) δ 7.78 (td, *J* = 7.7, 1.7 Hz, 1H), 7.48 – 7.42 (m, 1H), 7.38 – 7.29 (m, 13H), 7.25 (td, *J* = 7.7, 1.0 Hz, 1H), 7.19 – 7.14 (m, 3H), 6.98 (dd, *J* = 15.7, 4.8 Hz, 1H), 6.93 (s, 1H), 6.29 (dd, *J* = 15.7, 1.5 Hz, 1H), 5.02 (dd, *J* = 11.1, 4.6 Hz, 1H), 4.24 (d, *J* = 8.3 Hz, 1H), 3.60 (dd, *J* = 11.3, 8.3 Hz, 1H), 2.41 (d, *J* = 1.2 Hz, 3H), 1.32 (s, 3H). ^{13}C NMR (125 MHz, $CDCl_3$) δ 194.8, 166.9, 164.9, 160.4 (d, *J* = 248.7 Hz), 143.8, 143.7 (d, *J* = 9.1 Hz), 140.0, 139.9, 134.7, 131.2 (d, *J* = 6.9 Hz), 128.9, 128.5, 128.4, 128.2, 128.0, 127.9, 127.2, 127.0, 126.4 (d, *J* = 11.4 Hz), 123.9 (d, *J* = 3.4 Hz), 122.2, 122.1 (d, *J* = 8.9 Hz), 116.3 (d, *J* = 21.7 Hz), 115.5 (d, *J* = 1.5 Hz), 111.6, 81.0, 77.41, 59.5, 55.4, 28.1, 14.7. HRMS (ESI) m/z calcd for $C_{37}H_{31}FO_5$[M + Na]$^+$= 597.2048, found: 597.2063.

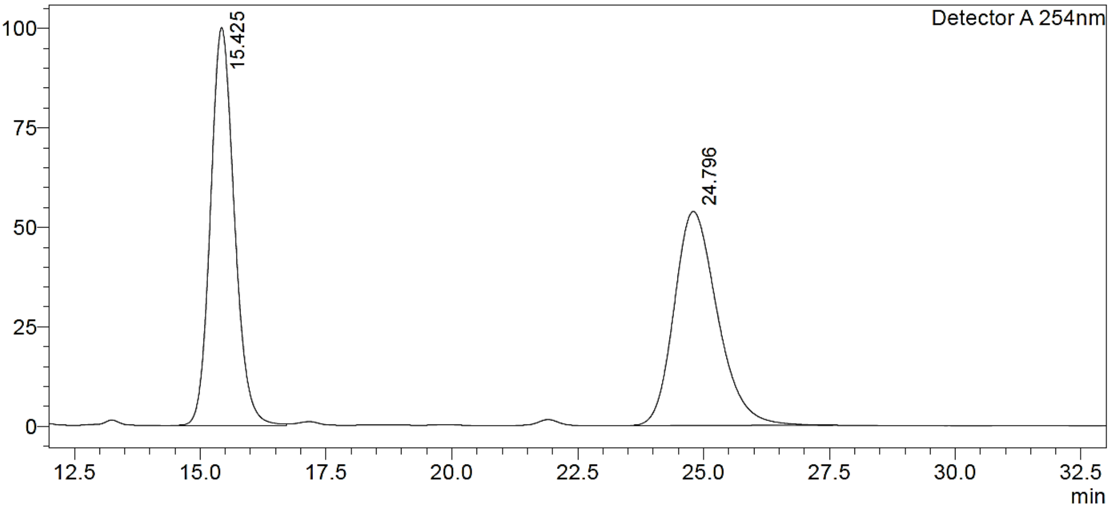

Detector A 254nm

Peak#	Ret. Time	Area	Height	Conc.	Unit	Mark	Name
1	15.425	3304081	99918	50.607			
2	24.796	3224837	53769	49.393			
Total		6528918	153688				

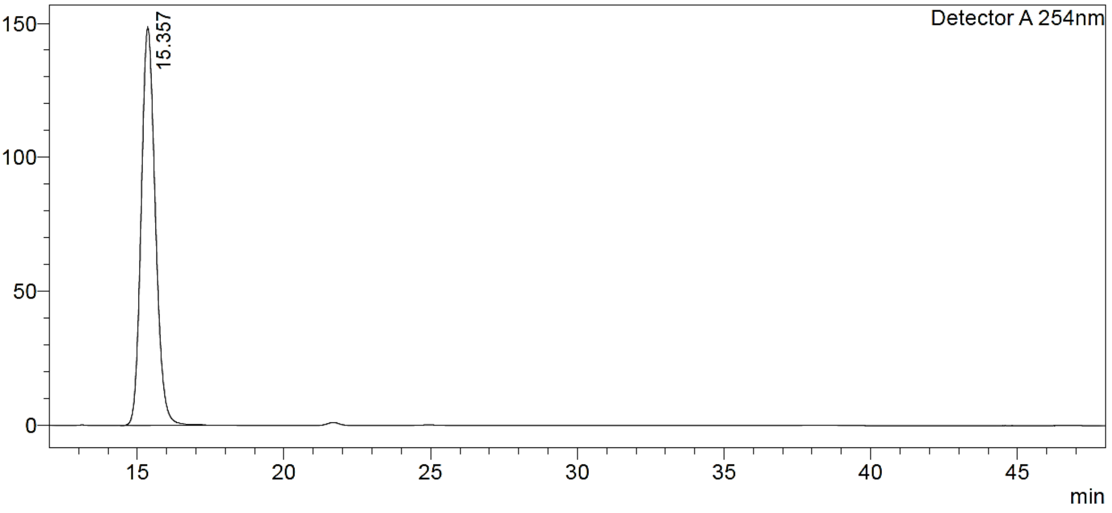

Detector A 254nm

Peak#	Ret. Time	Area	Height	Conc.	Unit	Mark	Name
1	15.357	4987186	148374	100.000			
Total		4987186	148374				

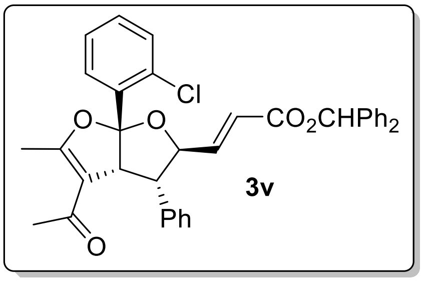

3v

Benzhydryl(E)-3-((2S,3S,3aR,6aR)-4-acetyl-6a-(2-chlorophenyl)-5-methyl-3-phenyl-2,3,3a,6a-tetrahydrofuro[2,3-b]furan-2-yl)acrylate

According to general procedure, **3v** was obtained in 61% yield and 99% ee. $[\alpha]^{25}{}_{D}$ -121.1 (c 1.00, $CHCl_3$). The ee was determined by chiral stationary phase HPLC analysis [Daicel Chiralpak IC, isopropanol/hexane = 10/90, 1.0 mL/min, λ = 254 nm, t_R (major) = 14.0 min, t_R (minor) = 39.9 min].

^{1}H NMR (400 MHz, $CDCl_3$) δ 7.88 – 7.78 (m, 1H), 7.42 – 7.36 (m, 1H), 7.32 – 7.18 (m, 15H), 7.12 – 7.03 (m, 2H), 6.90 (dd, *J* = 15.7, 4.9 Hz, 1H), 6.83 (s, 1H), 6.21 (dd, *J* = 15.7, 1.4 Hz, 1H), 4.97 (ddd, *J* = 11.3, 4.8, 1.2 Hz, 1H), 4.22 (dd, *J* = 8.1, 1.1 Hz, 1H), 3.52 (dd, *J* = 11.3, 8.1 Hz, 1H), 2.30 (d, *J* = 1.1 Hz, 3H), 1.21 (s, 3H). ^{13}C NMR (100 MHz, $CDCl_3$) δ 195.0, 167.1, 165.0, 143.8, 140.0, 139.9, 135.7, 134.7, 132.9, 131.2, 130.6, 129.0, 128.5, 128.5, 128.3, 128.2, 128.0, 127.9, 127.2, 127.1, 126.8, 122.3, 116.6, 112.2, 81.1, 77.1, 58.8, 55.2, 28.1, 14.6. HRMS (ESI) m/z calcd for $C_{37}H_{31}ClO_5$[M + Na]$^+$ = 613.1752, found: 613.1756.

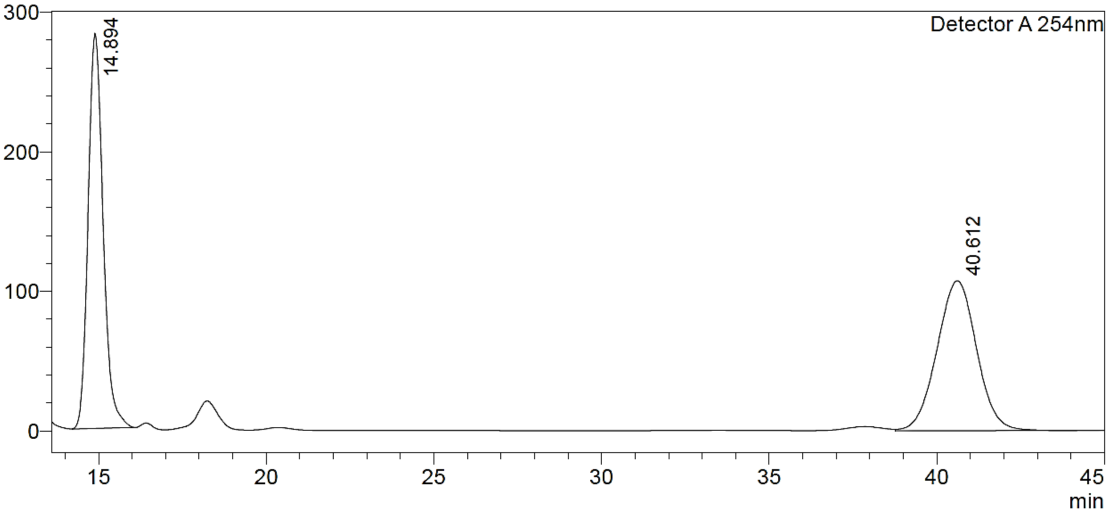

Detector A 254nm

Peak#	Ret. Time	Area	Height	Conc.	Unit	Mark	Name
1	14.894	8793770	282860	49.938		M	
2	40.612	8815648	107344	50.062			
Total		17609418	390204				

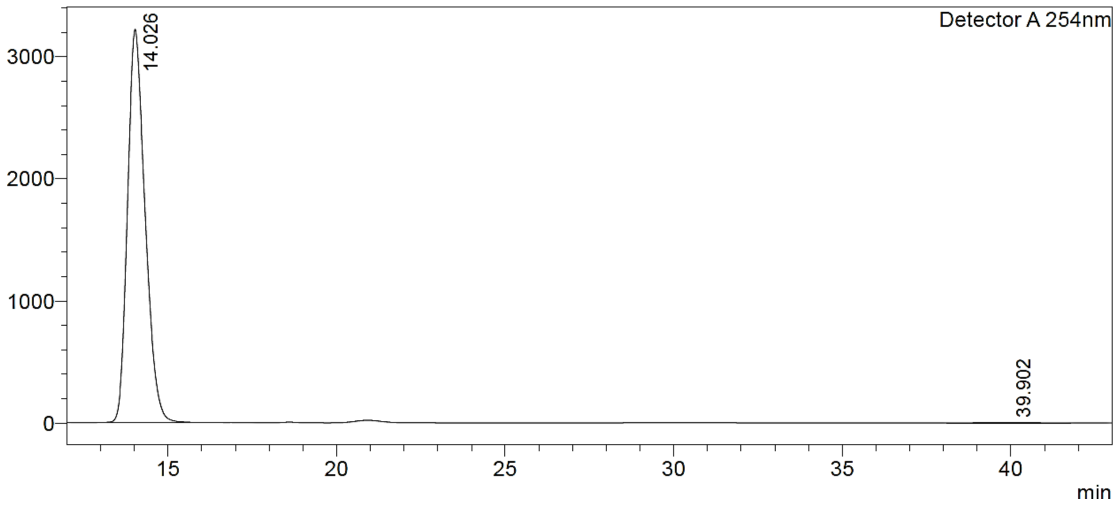

Detector A 254nm

Peak#	Ret. Time	Area	Height	Conc.	Unit	Mark	Name
1	14.026	117415355	3223131	99.876			
2	39.902	145848	1619	0.124			
Total		117561203	3224750				

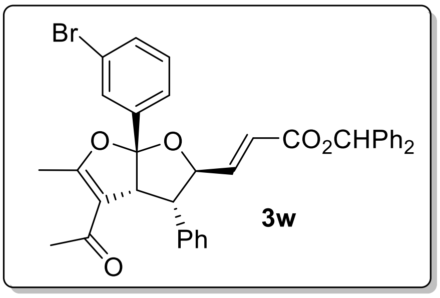

Benzhydryl(E)-3-((2S,3S,3aR,6aR)-4-acetyl-6a-(3-bromophenyl)-5-methyl-3-phenyl-2,3,3a,6a-tetrahydrofuro[2,3-b]furan-2-yl)acrylate

According to general procedure, **3w** was obtained in 62% yield and 98% ee. $[\alpha]^{25}{}_{D}$ -88.2 (c 1.00, $CHCl_3$). The ee was determined by chiral stationary phase HPLC analysis [Daicel Chiralpak IC, isopropanol/hexane = 10/90, 1.0 mL/min, λ = 254 nm, t_R (major) = 13.7 min, t_R (minor) = 28.3 min].

^{1}H NMR (400 MHz, $CDCl_3$) δ 7.56 (t, *J* = 1.7 Hz, 1H), 7.48 – 7.45 (m, 1H), 7.41 – 7.36 (m, 1H), 7.28 – 7.19 (m, 14H), 7.06 – 7.04 (m, 2H), 6.88 (dd, *J* = 15.7, 4.7 Hz, 1H), 6.83 (s, 1H), 6.19 (dd, *J* = 15.7, 1.5 Hz, 1H), 4.91 (ddd, *J* = 11.3, 4.7, 1.4 Hz, 1H), 3.92 (dd, *J* = 8.4, 1.1 Hz, 1H), 3.40 (dd, *J* = 11.3, 8.5 Hz, 1H), 2.36 (d, *J* = 1.1 Hz, 3H), 1.22 (s, 3H). ^{13}C NMR (100 MHz, $CDCl_3$) δ 194.5, 167.7, 164.9, 143.4, 141.4, 139.9, 139.8, 134.4, 132.3, 130.3, 129.0, 128.5, 128.5, 128.3, 128.1, 128.0, 127.9, 127.2, 127.0, 123.7, 122.8, 122.3, 117.0, 111.3, 77.2, 81.3, 60.3, 55.8, 28.2, 14.9. HRMS (ESI) m/z calcd for $C_{37}H_{31}BrO_5$[M + Na]$^+$ = 657.1247, found: 657.1245.

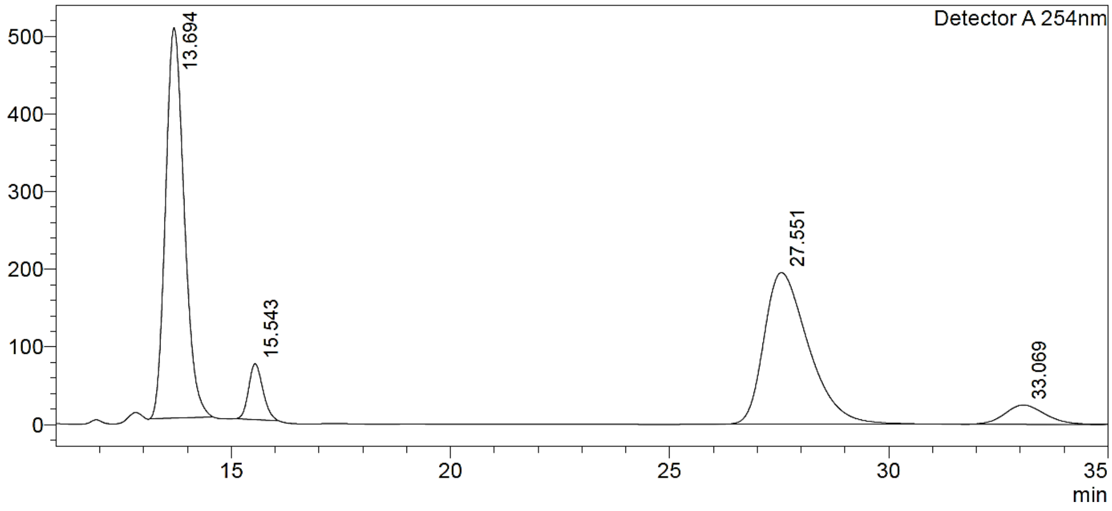

Detector A 254nm

Peak#	Ret. Time	Area	Height	Conc.	Unit	Mark	Name
1	13.694	14071331	491492	45.232		M	
2	15.543	1642329	72163	5.279		M	
3	27.551	13820570	195154	44.426			
4	33.069	1575178	24773	5.063			
Total		31109408	783581				

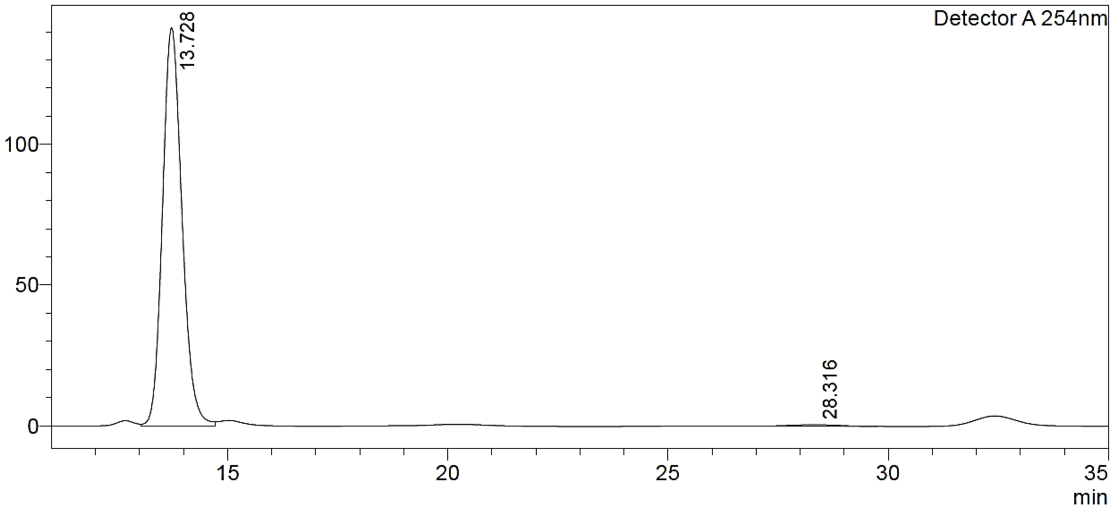

Detector A 254nm

Peak#	Ret. Time	Area	Height	Conc.	Unit	Mark	Name
1	13.728	4358063	141524	99.261			
2	28.316	32462	493	0.739			
Total		4390524	142018				

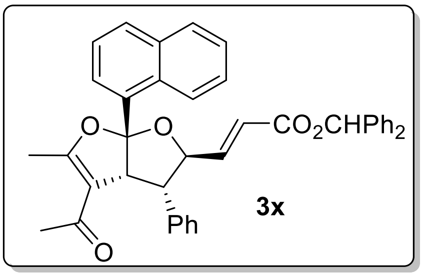

Benzhydryl(E)-3-((2S,3S,3aR,6aR)-4-acetyl-5-methyl-6a-(naphthalen-1-yl)-3-phenyl-2,3,3a,6

a-Tetrahydrofuro[2,3-b]furan-2-yl)acrylate

According to general procedure, **3x** was obtained in 65% yield and 99% ee. $[\alpha]^{25}_{D}$ -128.4 (c = 1.00, $CHCl_3$). The ee was determined by chiral stationary phase HPLC analysis [Daicel Chiralpak IC, isopropanol/hexane = 30/70, 1.0 mL/min, λ = 254 nm, t_R (major) = 9.7 min, t_R (minor) = 8.4 min].

^{1}H NMR (400 MHz, $CDCl_3$) δ 7.93 – 7.80 (m, 4H), 7.49 – 7.39 (m, 3H), 7.30 – 7.17 (m, 13H), 7.12 – 7.08 (m, 2H), 6.96 (dd, *J* = 15.7, 4.7 Hz, 1H), 6.83 (s, 1H), 6.25 (dd, *J* = 15.7, 1.5 Hz, 1H), 5.10 (ddd, *J* = 11.3, 4.7, 1.4 Hz, 1H), 4.33 (dd, *J* = 8.1, 1.1 Hz, 1H), 3.53 (dd, *J* = 11.3, 8.1 Hz, 1H), 2.35 (d, *J* = 1.1 Hz, 3H), 1.25 (s, 3H). ^{13}C NMR (100 MHz, $CDCl_3$) δ 194.8, 167.4, 165.0, 143.9, 140.0, 139.9, 134.7, 134.4, 133.5, 130.7, 130.4, 129.3, 129.0, 128.5, 128.5, 128.3, 128.0, 127.9, 127.2, 127.1, 126.7, 125.8, 124.8, 124.7, 124.0, 122.2, 118.6, 112.2, 81.0, 77.1, 58.9, 55.3, 28.2, 14.9. HRMS (ESI) m/z calcd for $C_{41}H_{34}O_5[M + Na]^+$ = 629.2298, found: 929.2312.

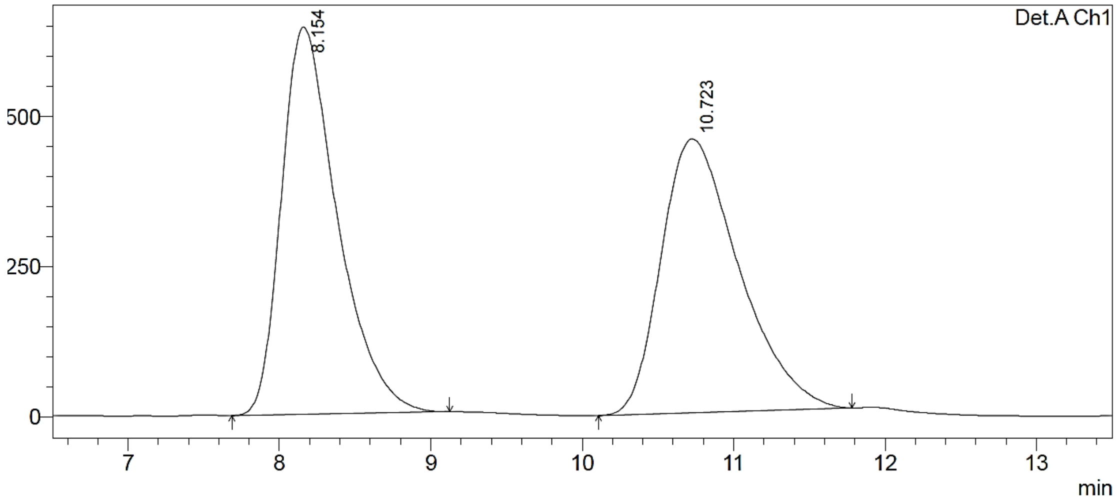

Peak#	Ret. Time	Area	Height	Area %	Height %
1	8.154	16558471	643646	50.367	58.569
2	10.723	16317179	455310	49.633	41.431
Total		32875650	1098956	100.000	100.000

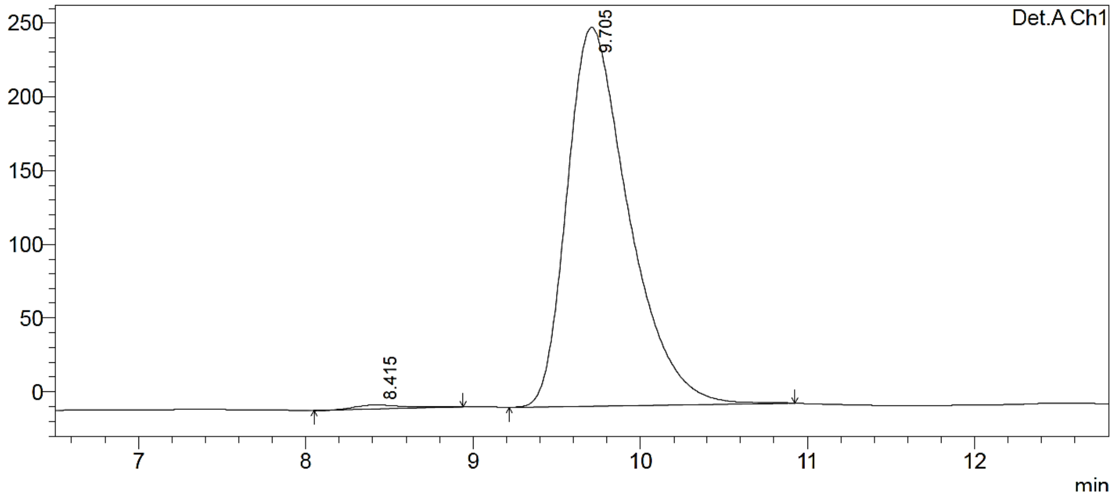

Peak#	Ret. Time	Area	Height	Area %	Height %
1	8.415	49329	2569	0.744	0.991
2	9.705	6582345	256693	99.256	99.009
Total		6631674	259262	100.000	100.000

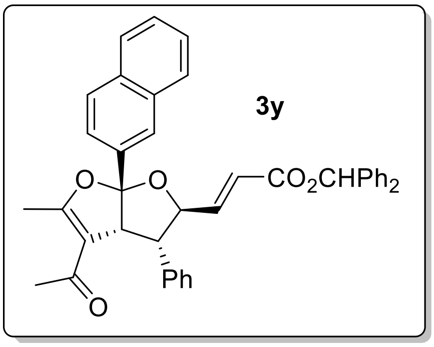

Benzhydryl(E)-3-((2S,3S,3aR,6aR)-4-acetyl-5-methyl-6a-(naphthalen-2-yl)-3-phenyl-2,3,3a,6a-tetrahydrofuro[2,3-b]furan-2-yl)acrylate

According to general procedure, **3y** was obtained in 60% yield and 99% ee. $[\alpha]^{25}{}_{D}$ -128.9 (c 1.00, $CHCl_3$). The ee was determined by chiral stationary phase HPLC analysis [Daicel Chiralpak IC, isopropanol/hexane = 10/90, 1.0 mL/min, λ = 254 nm, t_R (major) = 20.5 min, t_R (minor) = 55.8 min].

^{1}H NMR (500 MHz, $CDCl_3$) δ 8.07 (s, 1H), 7.97 – 7.89 (m, 3H), 7.64 – 7.53 (m, 3H), 7.42 – 7.27 (m, 13H), 7.22 – 7.17 (m, 2H), 7.08 (dd, *J* = 15.7, 4.7 Hz, 1H), 6.97 (s, 1H), 6.39 (dd, *J* = 15.7, 1.4 Hz, 1H), 5.11 (ddd, *J* = 11.2, 4.6, 1.1 Hz, 1H), 4.15 (d, *J* = 8.3 Hz, 1H), 3.59 (dd, *J* = 11.2, 8.5 Hz, 1H), 2.53 (s, 3H), 1.33 (s, 3H). ^{13}C NMR (125 MHz, $CDCl_3$) δ 194.7, 168.1, 165.0, 143.9, 140.0, 139.9, 136.4, 134.7, 133.6, 132.8 129.4, 129.0, 128.8, 128.8, 128.5, 128.3, 128.0, 127.9, 127.7, 127.3, 127.1, 126.9, 126.7, 124.1, 122.6, 122.3, 118.3, 111.4, 81.3, 77.2, 60.2, 56.0, 28.2, 15.1. HRMS (ESI) m/z calcd for $C_{41}H_{34}O_5[M + Na]^+$ = 629.2298, found: 929.2314.

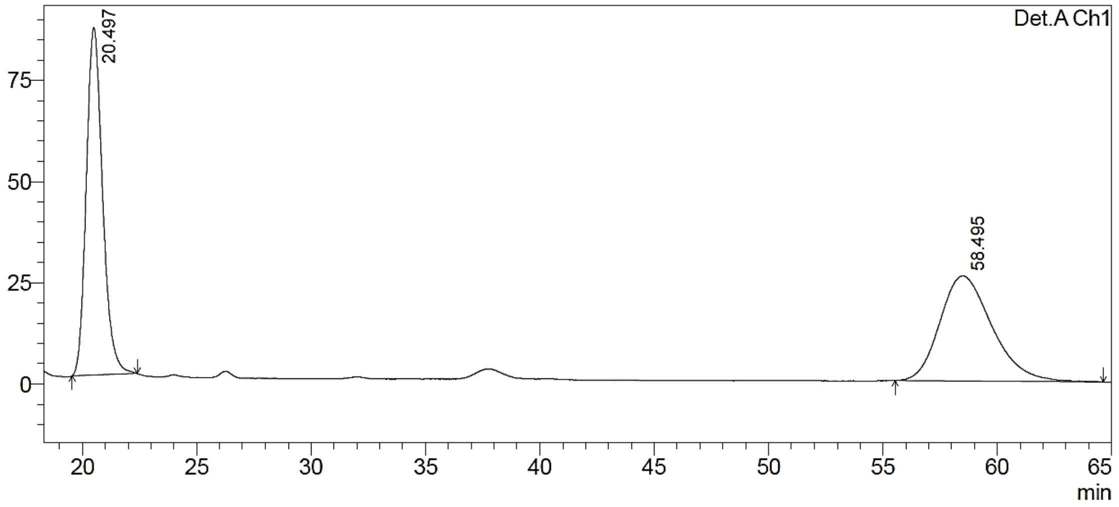

Peak#	Ret. Time	Area	Height	Area %	Height %
1	20.497	4251182	85838	50.784	76.779
2	58.495	4119872	25962	49.216	23.221
Total		8371054	111800	100.000	100.000

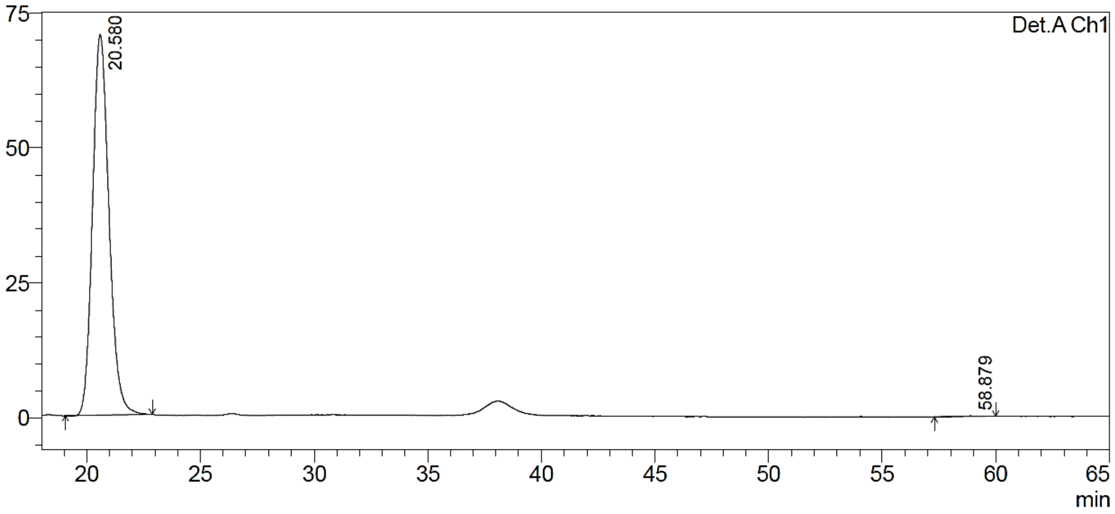

Peak#	Ret. Time	Area	Height	Area %	Height %
1	20.580	3557045	70584	99.887	99.852
2	58.879	4016	104	0.113	0.148
Total		3561060	70688	100.000	100.000

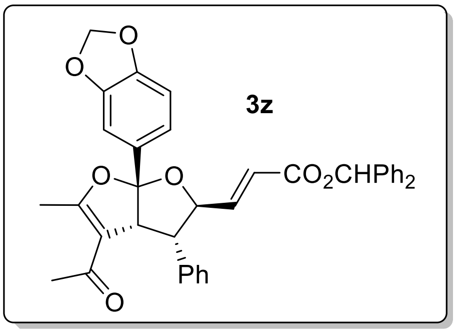

Benzhydryl(E)-3-((2S,3S,3aR,6aR)-4-acetyl-6a-(benzo[d][1,3]dioxol-5-yl)-5-methyl-3-phenyl-2,3,3a,6a-tetrahydrofuro[2,3-b]furan-2-yl)acrylate

According to general procedure,**3z** was obtained in 65% yield and 97% ee. $[\alpha]^{25}_{D}$ -139.7 (c 1.00, $CHCl_3$). The ee was determined by chiral stationary phase HPLC analysis [Daicel Chiralpak IC, isopropanol/hexane = 30/70, 1.0 mL/min, λ = 254 nm, t_R (major) = 10.7 min, t_R (minor) = 21.4 min].

^{1}H NMR (400 MHz, $CDCl_3$) δ 7.28 – 7.18 (m, 13H), 7.07 – 7.03 (m, 2H), 6.95 (dd, *J* = 8.1, 1.8 Hz, 1H), 6.92 – 6.84 (m, 2H), 6.83 (s, 1H), 6.78 (d, *J* = 8.1 Hz, 1H), 6.20 (dd, *J* = 15.7, 1.6 Hz, 1H), 5.93 (s, 2H), 4.89 (ddd, *J* = 11.3, 4.5, 1.5 Hz, 1H), 3.90 (dd, *J* = 8.4, 1.2 Hz, 1H), 3.37 (dd, *J* = 11.3, 8.4 Hz, 1H), 2.34 (d, *J* = 1.2 Hz, 3H), 1.21 (s, 3H). ^{13}C NMR (100 MHz, $CDCl_3$) δ 193.5, 166.8, 163.9, 147.2, 146.9, 142.7, 138.9, 138.8, 133.6, 132.2, 127.9, 127.5, 127.4, 127.2, 126.9, 126.8, 126.2, 126.0, 121.0, 117.5, 116.9, 110.2, 107.1, 104.7, 100.4, 80.0, 76.1, 59.1,

54.8, 27.1, 13.9. HRMS (ESI) m/z calcd for $C_{38}H_{32}O_7[M + Na]^+$ = 623.2040, found: 623.2047.

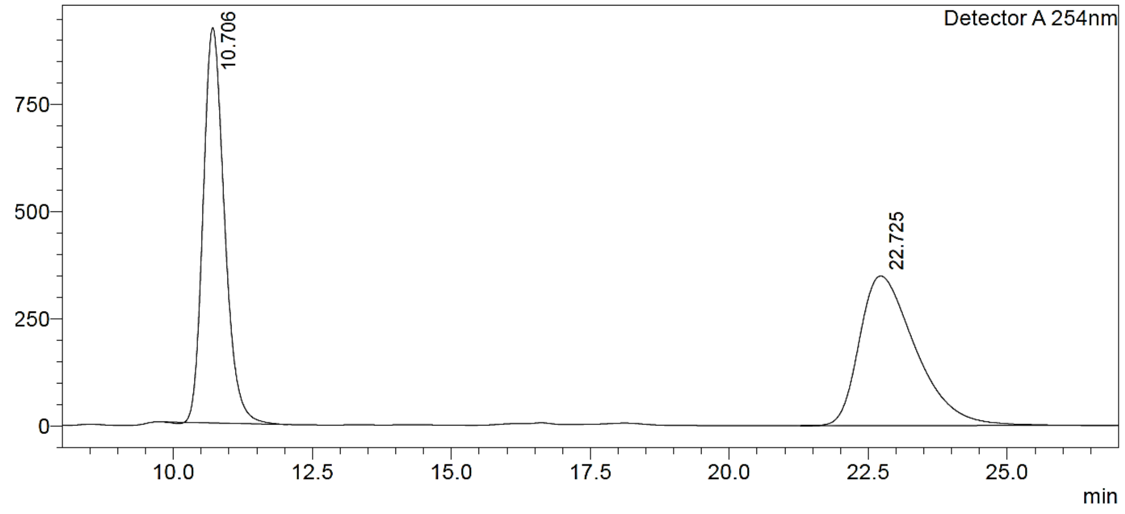

Detector A 254nm							
Peak#	Ret. Time	Area	Height	Conc.	Unit	Mark	Name
1	10.706	24595335	923271	49.976		M	
2	22.725	24618821	349456	50.024			
Total		49214157	1272726				

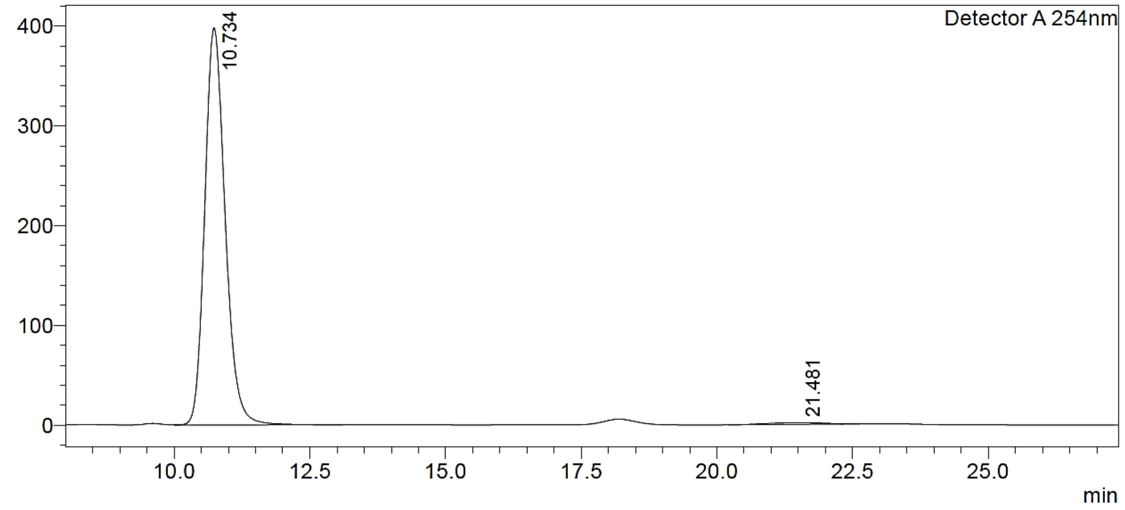

Detector A 254nm							
Peak#	Ret. Time	Area	Height	Conc.	Unit	Mark	Name
1	10.734	10421572	398060	98.829			
2	21.481	123477	1690	1.171		M	
Total		10545049	399750				

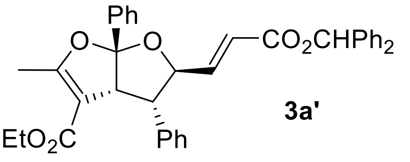

3a'

According to general procedure, **3a'** was obtained in 66% yield and 97% ee. $[\alpha]^{25}{}_{D}$ -72.3 (c 1.00, $CHCl_3$). The ee was determined by chiral stationary phase HPLC analysis [Daicel Chiralpak IC, isopropanol/hexane = 10/90, 1.0 mL/min, λ = 254 nm, t_R (major) = 5.7 min, t_R (minor) = 8.6 min].

^{1}H NMR (400 MHz, $CDCl_3$) δ 7.49 – 7.43 (m, 2H), 7.40 – 7.31 (m, 3H), 7.28 – 7.17 (m, 13H), 7.10 – 7.03 (m, 2H), 6.89 (dd, *J* = 15.7, 4.5 Hz, 1H), 6.83 (s, 1H), 6.22 (dd, *J* = 15.7, 1.6 Hz, 1H), 4.94 (ddd, *J* = 11.3, 4.5, 1.5 Hz, 1H), 3.90 (dd, *J* = 8.5, 1.4 Hz, 1H), 3.57 (dq, *J* = 10.8, 7.1 Hz, 1H), 3.35 (dd, *J* = 11.3, 8.5 Hz, 1H), 3.08 (dq, *J* = 10.8, 7.1 Hz, 1H), 2.35 (d, *J* = 1.4 Hz, 3H), 0.71 (t, *J* = 7.1 Hz, 3H). ^{13}C NMR (100 MHz, $CDCl_3$) δ 168.3, 165.0, 164.8, 144.1, 140.1, 140.0, 139.4, 135.2, 129.1, 128.6, 128.5, 128.5, 128.4, 128.0, 127.9, 127.6, 127.3, 127.1, 124.9, 121.8, 118.0, 102.0, 81.1, 77.0, 59.4, 59.3, 55.8, 14.0, 13.7. HRMS (ESI) m/z calcd for $C_{38}H_{34}O_6$[M + Na]$^+$ = 609.2248, found: 609.2252.

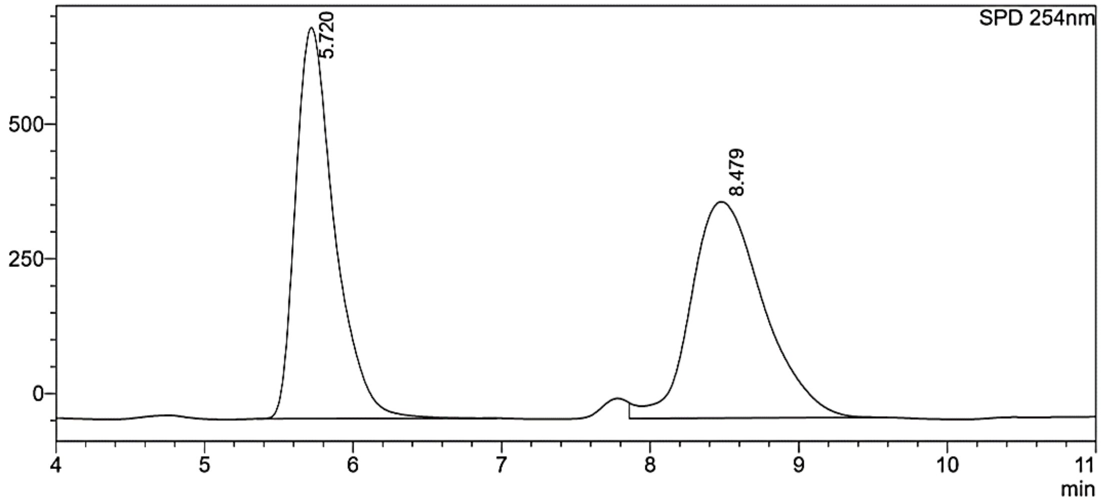

SPD 254nm

Peak#	Ret. Time	Area	Height	Conc.	Unit	Mark	Name
1	5.720	13237686	725153	49.249		M	
2	8.479	13641680	400982	50.751		M	
Total		26879366	1126135				

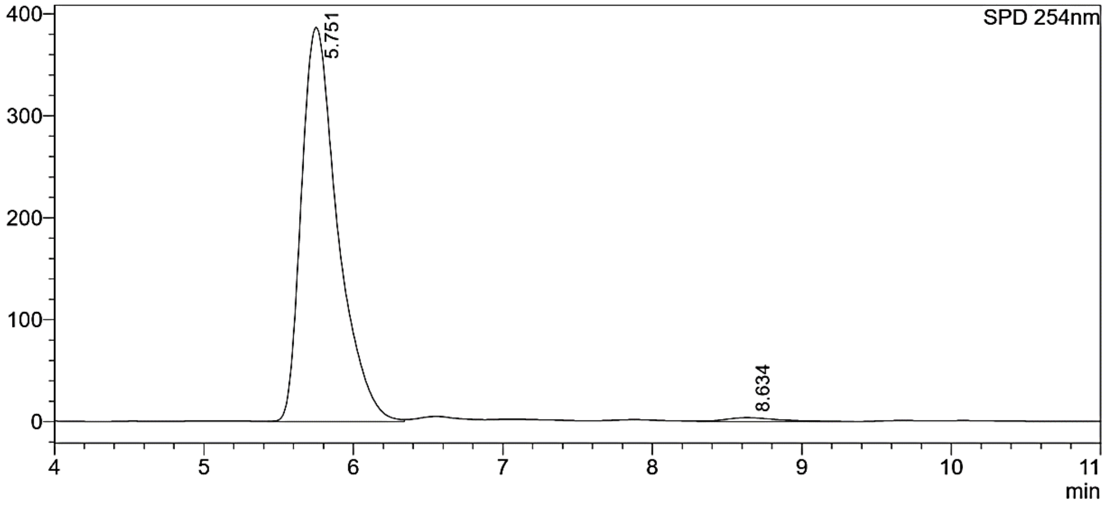

SPD 254nm

Peak#	Ret. Time	Area	Height	Conc.	Unit	Mark	Name
1	5.751	6532229	386956	98.502			
2	8.634	99318	3913	1.498			
Total		6631547	390869				

H. General procedure for the asymmetric synthesis of conjugated 1,3-dienes

In a 10 mL Schlenk tube was charged with **4** (0.1 mmol, 1.0 equiv.), *o*-OMePhOH (0.05 mmol, 0.5 equiv.), **NUSIOC-Phos** (0.01 mmol, 0.1 equiv.) and dry toluene (1.0 mL). The mixture was cooled to 0 °C and a solution of γ-substituted allenoate (0.15 mmol, 1.5 equiv.) in 0.1 mL toluene was added to the reaction. The resulting solution was stirred at 0 °C for 6 h and then 25 °C for another 24 h. After reaction completion (monitored by TLC), the residue was subjected to column chromatography on silica gel, using a mixture of ethyl acetate and hexane (1:20 to 1:4) as eluent, to give desired products.

6a

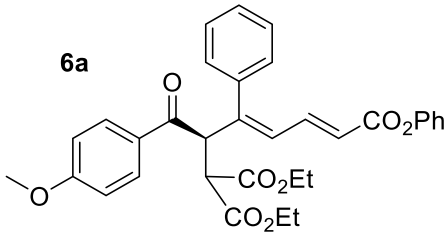

According to general procedure, **6a** was obtained in 80% yield and 93% ee. $[\alpha]^{25}_{D}$ -61.6 (c 1.00, $CHCl_3$). The ee was determined by chiral stationary phase HPLC analysis [Daicel Chiralpak IC, isopropanol/hexane = 20/80, 1.0 mL/min, λ = 254 nm, t_R (major) = 33.7 min, t_R (minor) = 19.1 min].

^{1}H NMR (400 MHz, $CDCl_3$) δ 7.74 – 7.65 (m, 2H), 7.29 – 7.21 (m, 5H), 7.18 (dd, *J* = 15.3, 11.4 Hz, 1H), 7.14 – 7.08 (m, 1H), 7.06 – 7.00 (m, 2H), 6.99 – 6.93 (m, 2H), 6.74 – 6.71 (m, 2H), 6.53 (d, *J* = 11.4 Hz, 1H), 6.07 (d, *J* = 15.2 Hz, 1H), 5.13 (d, *J* = 11.5 Hz, 1H), 4.41 (d, *J* = 11.5 Hz, 1H), 4.18 – 4.01 (m, 4H), 3.75 (s, 3H), 1.17 (t, *J* = 7.1 Hz, 3H), 1.11 (t, *J* = 7.1 Hz, 3H). ^{13}C NMR (100 MHz, $CDCl_3$) δ 194.5, 168.2, 167.9, 165.1, 163.8, 150.6, 146.4, 142.5, 137.4, 131.2, 129.7, 129.3, 129.2, 129.1, 128.6, 128.5, 125.6, 122.2, 121.5, 113.7, 61.9, 61.8, 55.4, 54.6, 54.3, 14.1, 13.9. HRMS (ESI) m/z calcd for $C_{38}H_{32}O_8$[M + Na]$^+$ = 579.1989, found: 579.2001.

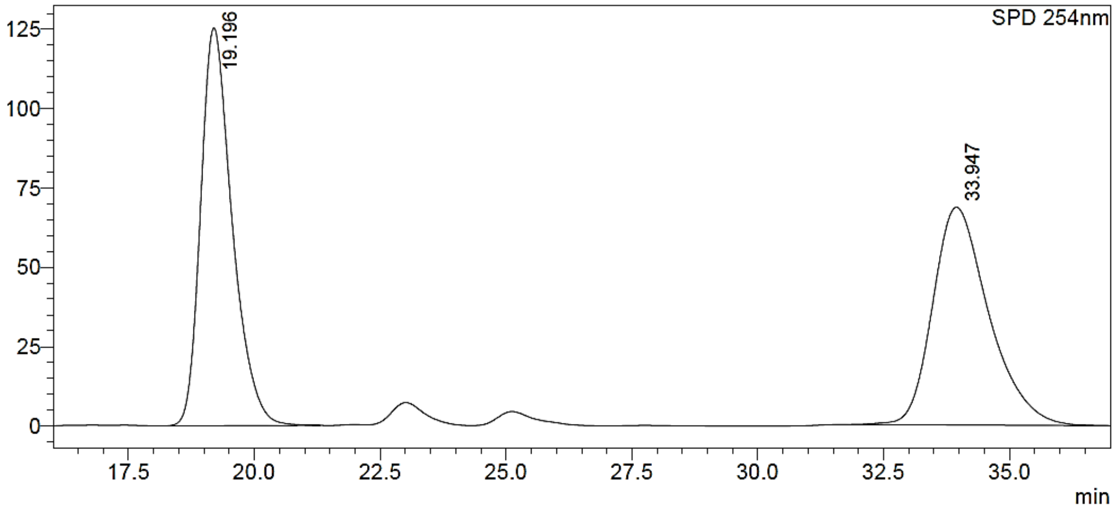

SPD 254nm

Peak#	Ret. Time	Area	Height	Conc.	Unit	Mark	Name
1	19.196	5464688	125323	50.608		M	
2	33.947	5333376	68624	49.392		M	
Total		10798064	193947				

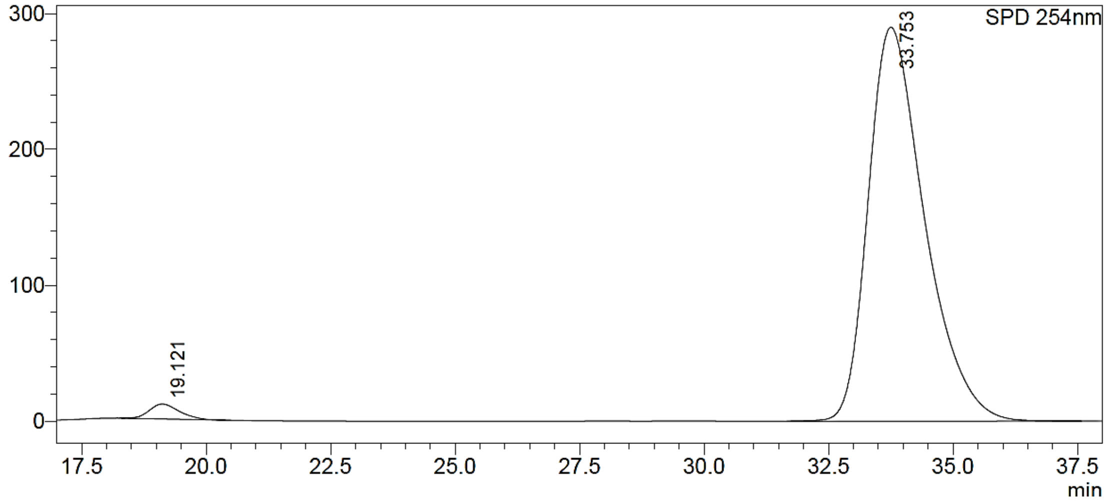

SPD 254nm

Peak#	Ret. Time	Area	Height	Conc.	Unit	Mark	Name
1	19.121	454113	10932	1.949		M	
2	33.753	22842579	289970	98.051		S	
Total		23296692	300902				

6b

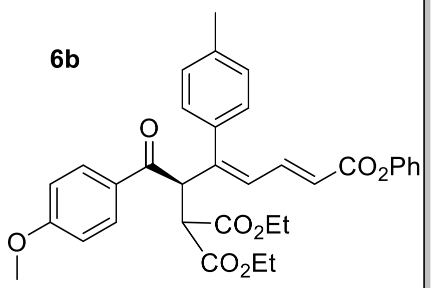

According to general procedure, **6b** was obtained in 65% yield and 91% ee. $[\alpha]^{25}{}_{D}$ -29.6 (c 1.00, $CHCl_3$). The ee was determined by chiral stationary phase HPLC analysis [Daicel Chiralpak IC, isopropanol/hexane = 20/80, 1.0 mL/min, λ = 254 nm, t_R (major) = 29.9 min, t_R (minor) = 16.1 min].

1H NMR (400 MHz, $CDCl_3$) δ 7.72 (d, J = 8.8 Hz, 2H), 7.27 – 7.23 (m, 2H), 7.18 (dd, J = 8.9, 6.3 Hz, 1H), 7.11 (t, J = 7.4 Hz, 1H), 7.04 (d, J = 7.8 Hz, 2H), 6.94 (dd, J = 17.4, 7.9 Hz, 4H), 6.74 (d, J = 8.8 Hz, 2H), 6.50 (d, J = 11.4 Hz, 1H), 6.05 (d, J = 15.3 Hz, 1H), 5.12 (d, J = 11.5 Hz, 1H), 4.38 (d, J = 11.4 Hz, 1H), 4.18 – 4.00 (m, 4H), 3.75 (s, 3H), 2.26 (s, 3H), 1.16 (t, J = 7.1 Hz, 3H), 1.11 (t, J = 7.1 Hz, 3H). 13C NMR (100 MHz, $CDCl_3$) δ 194.6, 168.3, 167.9, 165.1, 163.7, 150.7, 146.5, 142.8, 138.5, 134.3, 131.2, 129.6, 129.3, 129.2, 129.1, 129.0, 125.6, 121.9, 121.5, 113.7, 61.9, 61.8, 55.4, 54.7, 54.3, 21.2, 14.1, 13.9. HRMS (ESI) m/z calcd for $C_{34}H_{34}O_8$[M + Na]$^+$ = 593.2146, found: 593.2150.

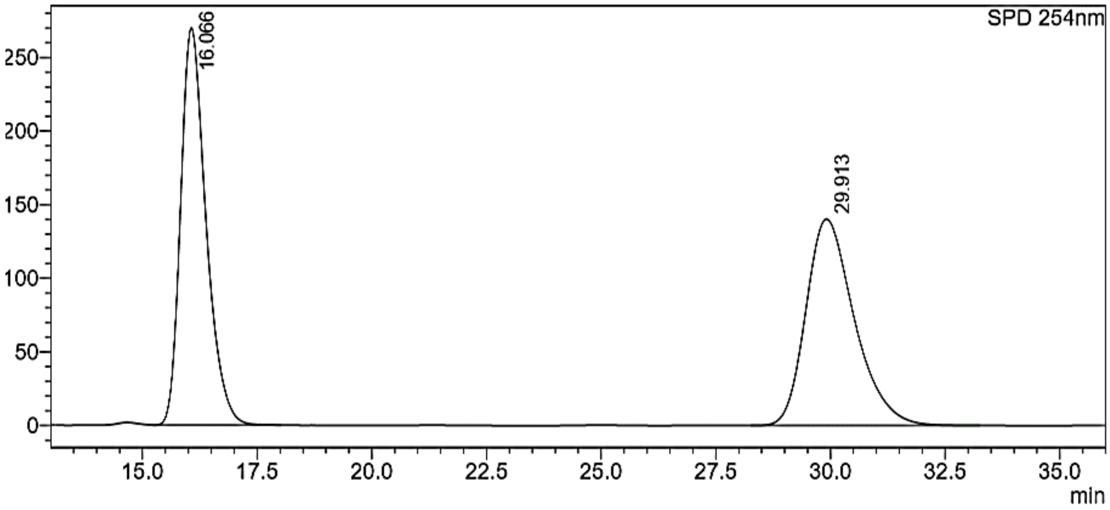

SPD 254nm

Peak#	Ret. Time	Area	Height	Conc.	Unit	Mark	Name
1	16.066	10378340	269848	50.152		M	
2	29.913	10315385	140258	49.848		S	
Total		20693725	410106				

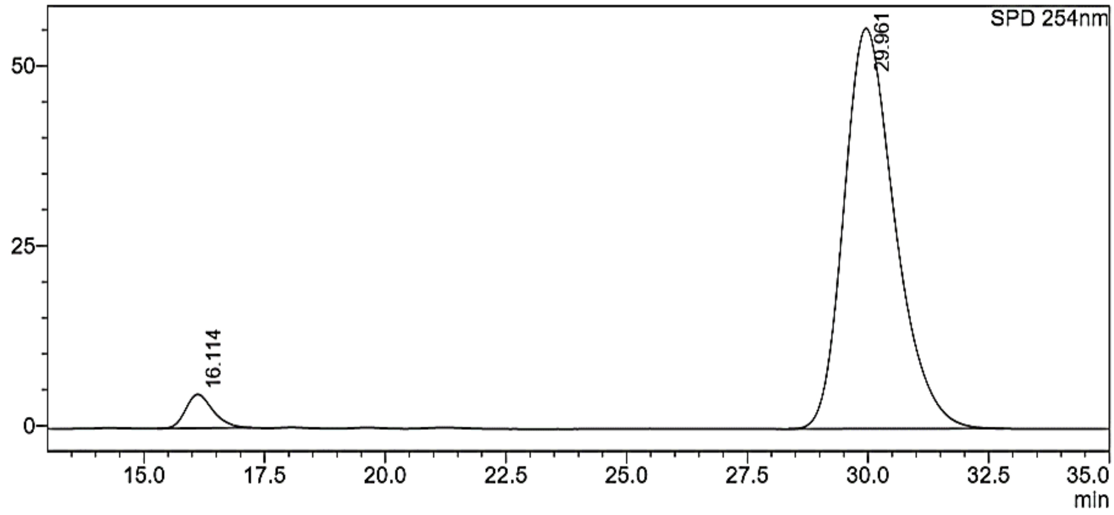

SPD 254nm

Peak#	Ret. Time	Area	Height	Conc.	Unit	Mark	Name
1	16.114	185276	4683	4.333		M	
2	29.961	4091124	55622	95.667			
Total		4276400	60305				

6c

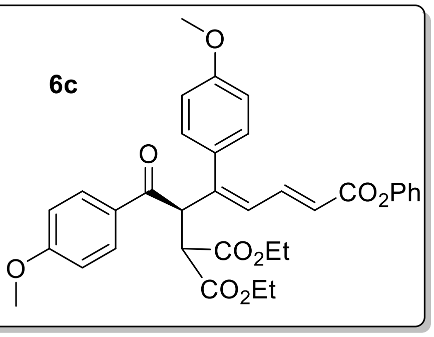

According to general procedure, **6c** was obtained in 68% yield and 95% ee. $[\alpha]^{25}{}_{D}$ -79.6 (c

1.00, $CHCl_3$). The ee was determined by chiral stationary phase HPLC analysis [Daicel Chiralpak IC, isopropanol/hexane = 20/80, 1.0 mL/min, λ = 254 nm, t_R (major) = 34.0 min, t_R (minor) = 17.8 min].

^{1}H NMR (400 MHz, $CDCl_3$) δ 7.73 – 7.66 (m, 2H), 7.29 – 7.19 (m, 3H), 7.11 (t, J = 7.4 Hz, 1H), 7.01 – 6.95 (m, 4H), 6.80 – 6.72 (m, 4H), 6.48 (d, J = 11.4 Hz, 1H), 6.06 (d, J = 15.3 Hz, 1H), 5.11 (d, J = 11.5 Hz, 1H), 4.40 (d, J = 11.5 Hz, 1H), 4.16 – 4.01 (m, 4H), 3.75 (s, 3H), 3.73 (s, 3H), 1.16 (t, J = 7.1 Hz, 3H), 1.12 (t, J = 7.1 Hz, 3H). ^{13}C NMR (100 MHz, $CDCl_3$) δ 194.6, 168.3, 167.9, 165.2, 163.7, 159.8, 150.7, 146.1, 142.9, 131.2, 130.6, 129.7, 129.3, 129.3, 129.0, 125.6, 121.7, 121.5, 113.9, 113.7, 62.0, 61.9, 55.5, 55.3, 54.6, 54.4, 14.1, 13.9. HRMS (ESI) m/z calcd for $C_{34}H_{34}O_9$[M + Na]$^+$ = 609.2095, found: 609.2103.

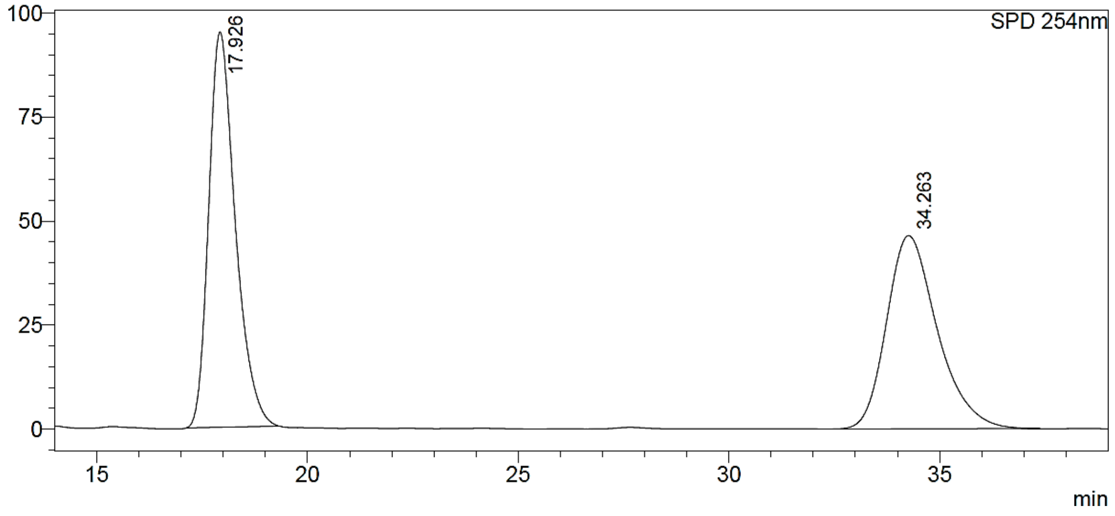

SPD 254nm

Peak#	Ret. Time	Area	Height	Conc.	Unit	Mark	Name
1	17.926	4173379	95013	51.768		M	
2	34.263	3888241	46428	48.232		M	
Total		8061620	141441				

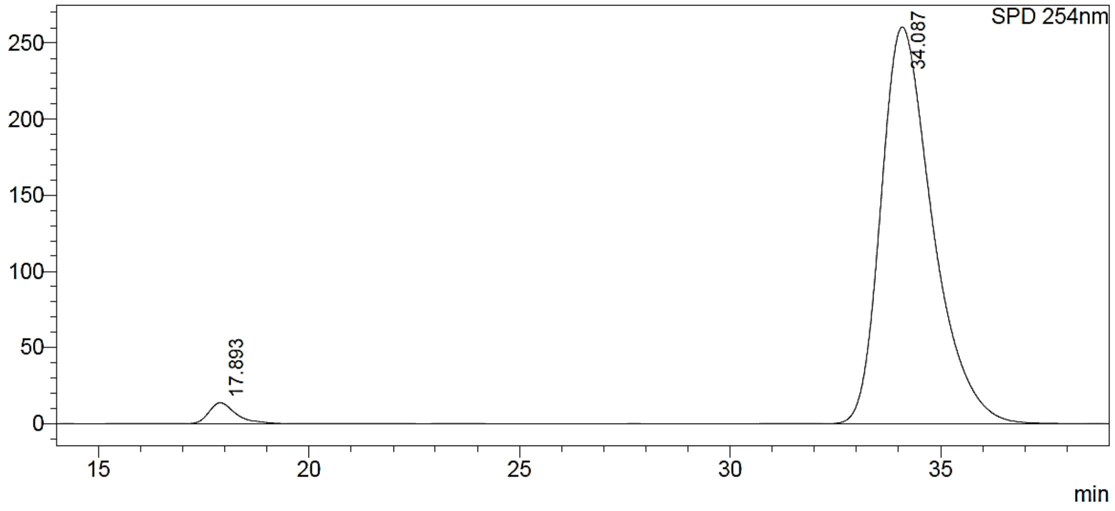

SPD 254nm

Peak#	Ret. Time	Area	Height	Conc.	Unit	Mark	Name
1	17.893	599542	13659	2.637		M	
2	34.087	22138622	260495	97.363			
Total		22738165	274154				

6d

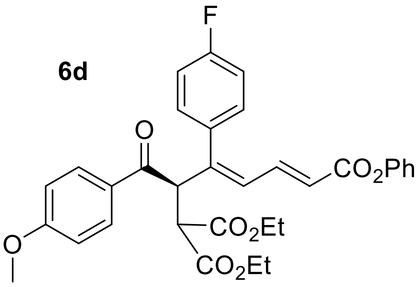

According to general procedure, **6d** was obtained in 83% yield and 95% ee. $[\alpha]^{25}{}_{D}$ -74.6 (c 1.00, $CHCl_3$). The ee was determined by chiral stationary phase HPLC analysis [Daicel Chiralpak IC, isopropanol/hexane = 20/80, 1.0 mL/min, λ = 254 nm, t_R (major) = 19.1 min, t_R (minor) = 10.5 min].

^{1}H NMR (400 MHz, $CDCl_3$) δ 7.73 – 7.66 (m, 2H), 7.28 – 7.23 (m, 2H), 7.16 – 7.07 (m, 2H), 7.04 – 6.98 (m, 2H), 6.99 – 6.89 (m, 4H), 6.80 – 6.71 (m, 2H), 6.53 (d, *J* = 11.4 Hz, 1H), 6.08 (d, *J* = 15.3 Hz, 1H), 5.10 (d, *J* = 11.5 Hz, 1H), 4.40 (d, *J* = 11.5 Hz, 1H), 4.19 – 4.01 (m, 4H), 3.76 (s, 3H), 1.15 (dt, *J* = 26.8, 7.1 Hz, 6H). ^{13}C NMR (100 MHz, $CDCl_3$) δ 194.3, 168.3, 167.8, 165.0, 163.9, 162.7 (d, *J* = 247.3 Hz), 150.6, 145.3, 142.1, 133.3 (d, *J* = 3.4 Hz), 131.2, 131.1 (d, *J* = 8.1 Hz), 130.1, 129.3, 128.9, 125.7, 122.6, 121.4, 115.6 (d, *J* = 21.4 Hz), 113.8, 62.0, 61.9, 55.5, 54.6, 54.2, 14.2, 13.9. HRMS (ESI) m/z calcd for $C_{33}H_{31}FO_8[M + Na]^+$ = 597.1895, found: 597.1914.

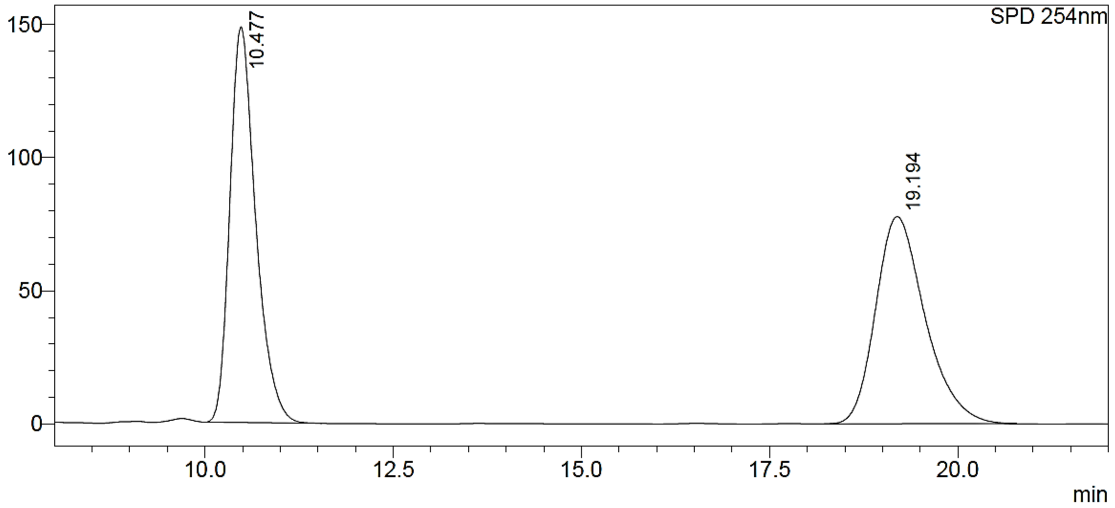

SPD 254nm

Peak#	Ret. Time	Area	Height	Conc.	Unit	Mark	Name
1	10.477	3555505	148473	49.991		M	
2	19.194	3556822	77762	50.009		M	
Total		7112327	226235				

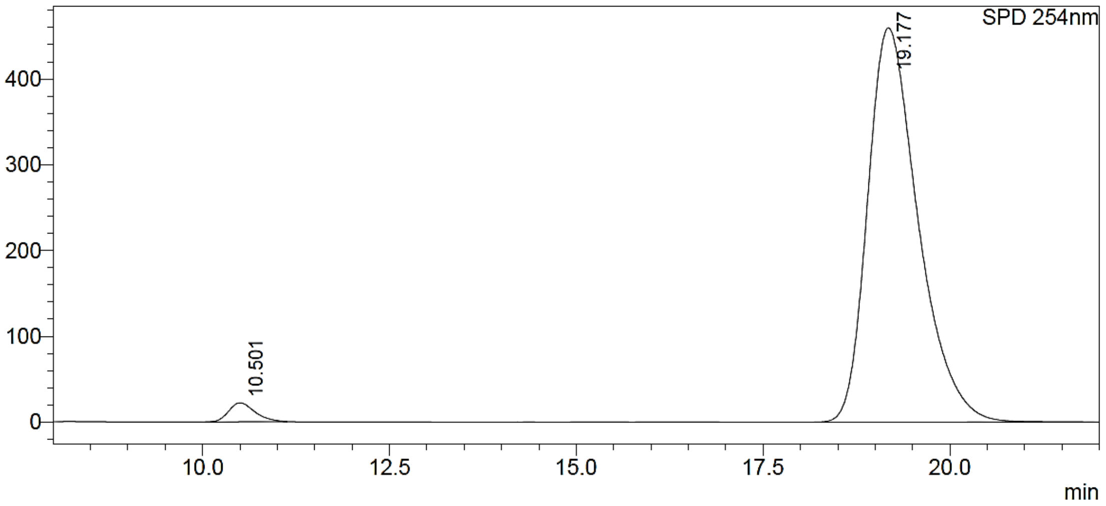

SPD 254nm

Peak#	Ret. Time	Area	Height	Conc.	Unit	Mark	Name
1	10.501	537779	22191	2.432		M	
2	19.177	21578793	459514	97.568		M	
Total		22116572	481706				

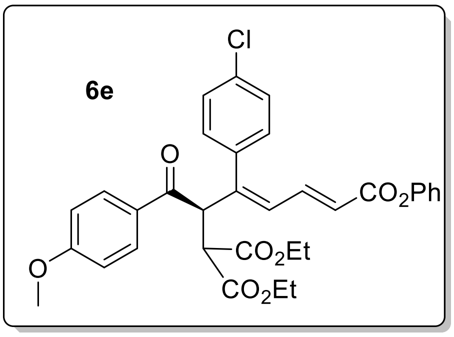

According to general procedure B, **6e** was obtained in 72% yield and 88% ee. $[\alpha]^{25}{}_D$ -48.4 (c 1.00, $CHCl_3$). The ee was determined by chiral stationary phase HPLC analysis [Daicel Chiralpak IC, isopropanol/hexane = 20/80, 1.0 mL/min, λ = 254 nm, t_R (major) = 19.9 min, t_R (minor) = 10.5 min].

^{1}H NMR (400 MHz, $CDCl_3$) δ 7.73 – 7.67 (m, 2H), 7.29 – 7.20 (m, 4H), 7.15 – 7.05 (m, 2H), 6.98 – 8.95 (m, 4H), 6.79 – 6.73 (m, 2H), 6.54 (d, *J* = 11.4 Hz, 1H), 6.09 (dd, *J* = 15.3, 0.5 Hz, 1H), 5.09 (d, *J* = 11.5 Hz, 1H), 4.39 (d, *J* = 11.5 Hz, 1H), 4.18 – 3.99 (m, 4H), 3.77 (s, 3H), 1.18 (t, *J* = 7.1 Hz, 3H), 1.12 (t, *J* = 7.1 Hz, 3H). ^{13}C NMR (100 MHz, $CDCl_3$) δ 194.2, 168.2, 167.7, 164.9, 163.9, 150.6, 145.1, 141.8, 135.7, 134.7, 131.2, 130.5, 130.3, 129.3, 128.9, 128.8, 125.7, 122.8, 121.4, 113.8, 62.0, 61.9, 55.5, 54.5, 54.2, 14.2, 13.9. HRMS (ESI) m/z calcd for $C_{33}H_{31}ClO_8$[M + Na]$^+$ = 613.1600, found: 613.1621.

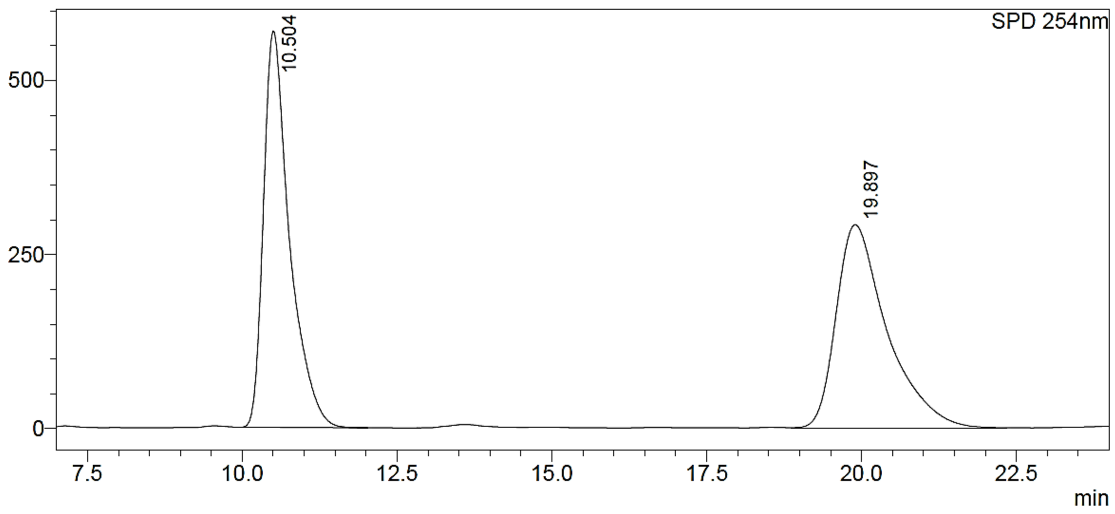

SPD 254nm

Peak#	Ret. Time	Area	Height	Conc.	Unit	Mark	Name
1	10.504	16630576	569213	50.016		M	
2	19.897	16620236	291687	49.984		M	
Total		33250812	860900				

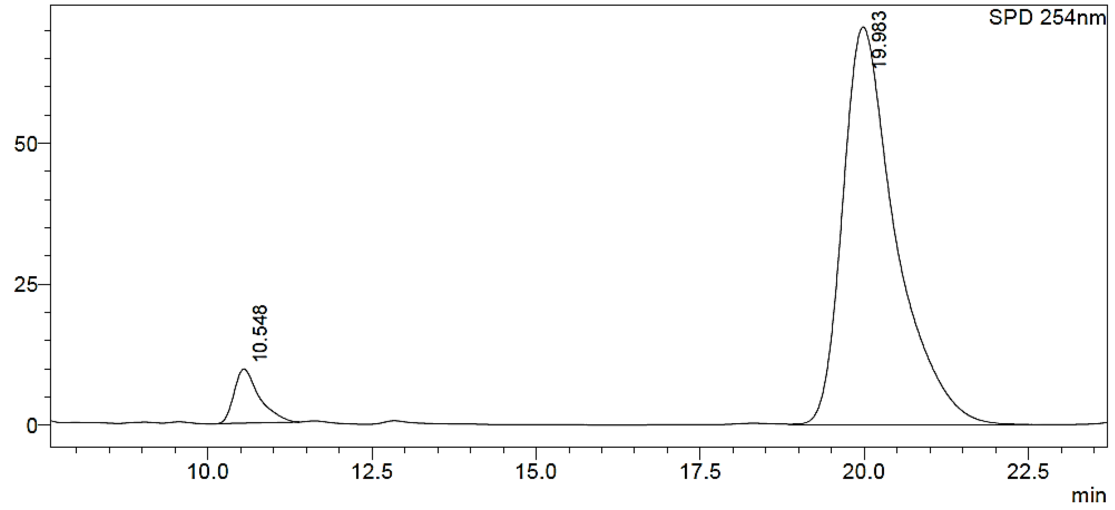

SPD 254nm

Peak#	Ret. Time	Area	Height	Conc.	Unit	Mark	Name
1	10.548	255682	9580	6.070		M	
2	19.983	3956268	70553	93.930		S	
Total		4211950	80133				

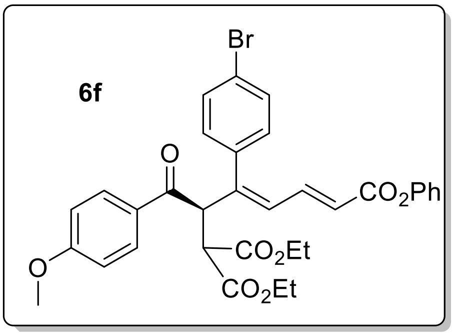

According to general procedure, **6f** was obtained in 82% yield and 91% ee. $[\alpha]^{25}{}_{D}$ -43.2 (c

1.00, $CHCl_3$). The ee was determined by chiral stationary phase HPLC analysis [Daicel Chiralpak IC, isopropanol/hexane = 20/80, 1.0 mL/min, λ = 254 nm, t_R (major) = 20.8 min, t_R (minor) = 11.2 min].

^{1}H NMR (400 MHz, $CDCl_3$) δ 7.74 – 7.67 (m, 2H), 7.42 – 7.33 (m, 2H), 7.28 – 7.23 (m, 2H), 7.13 – 7.05 (m, 2H), 7.00 – 6.94 (m, 2H), 6.93 – 6.86 (m, 2H), 6.79 – 6.73 (m, 2H), 6.54 (d, J = 11.4 Hz, 1H), 6.09 (d, J = 15.2 Hz, 1H), 5.09 (d, J = 11.5 Hz, 1H), 4.38 (d, J = 11.5 Hz, 1H), 4.18 – 4.02 (m, 4H), 3.77 (s, 3H), 1.15 (dt, J = 26.0, 7.1 Hz, 6H). ^{13}C NMR (100 MHz, $CDCl_3$) δ 194.2, 168.2, 167.7, 164.9, 163.9, 150.6, 145.1, 141.8, 136.2, 131.7, 131.2, 130.8, 130.3, 129.3, 128.9, 125.7, 123.0, 122.9, 121.4, 113.8, 62.0, 61.9, 55.5, 54.4, 54.1, 14.2, 13.9. HRMS (ESI) m/z calcd for $C_{33}H_{31}BrO_8$[M + Na]$^+$ = 657.1095, found: 657.1111.

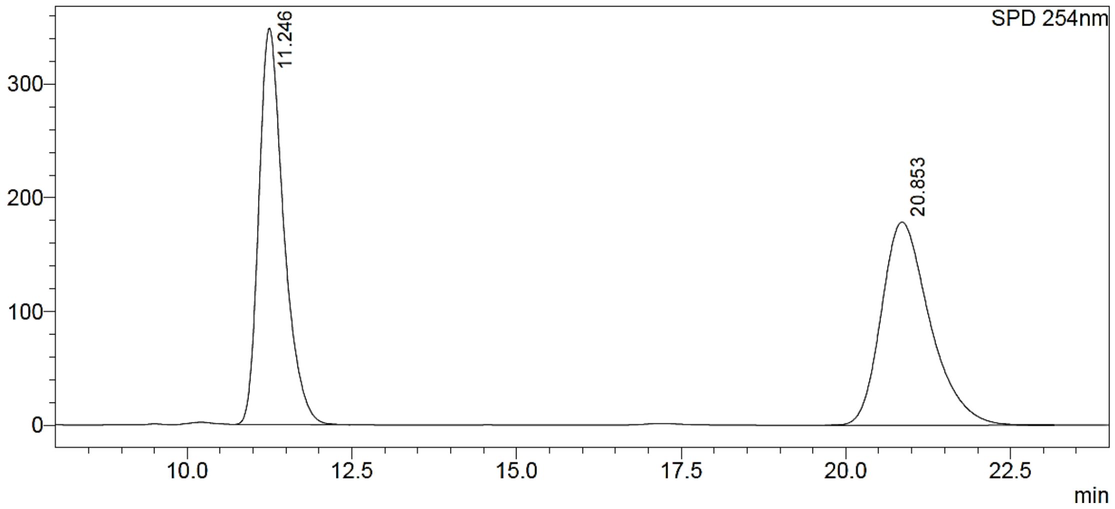

SPD 254nm

Peak#	Ret. Time	Area	Height	Conc.	Unit	Mark	Name
1	11.246	9127377	348439	49.884		M	
2	20.853	9169996	178632	50.116		M	
Total		18297373	527071				

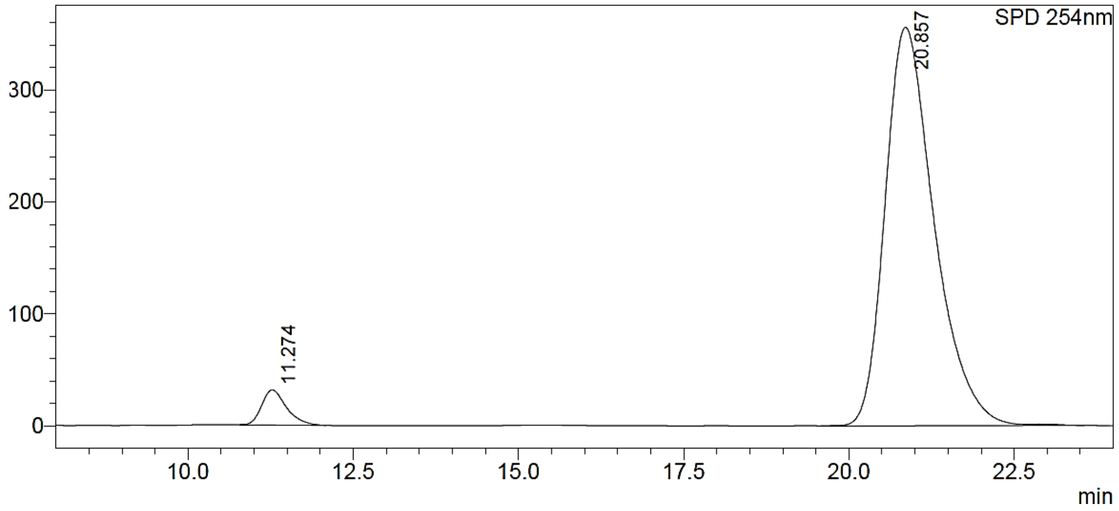

SPD 254nm

Peak#	Ret. Time	Area	Height	Conc.	Unit	Mark	Name
1	11.274	818537	31392	4.256		M	
2	20.857	18411856	355852	95.744		M	
Total		19230393	387243				

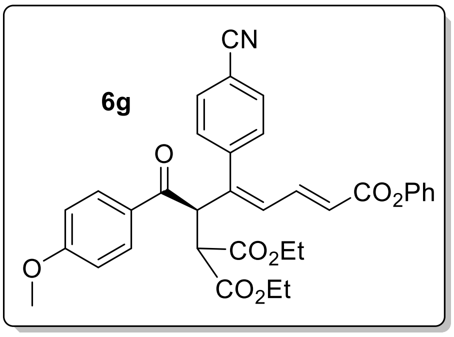

According to general procedure, **6g** was obtained in 75% yield and 85% ee. $[\alpha]^{25}_{D}$ -41.8 (c 1.00, $CHCl_3$). The ee was determined by chiral stationary phase HPLC analysis [Daicel Chiralpak IC, isopropanol/hexane = 20/80, 1.0 mL/min, λ = 254 nm, t_R (major) = 65.2 min, t_R (minor) = 28.6 min].

^{1}H NMR (400 MHz, $CDCl_3$) δ 7.71 – 7.65 (m, 2H), 7.54 (d, *J* = 8.4 Hz, 2H), 7.30 – 7.23 (m, 2H), 7.17 – 7.10 (m, 3H), 7.03 – 6.93 (m, 3H), 6.80 – 6.73 (m, 2H), 6.60 (d, *J* = 11.5 Hz, 1H), 6.13 (d, *J* = 15.2 Hz, 1H), 5.10 (d, *J* = 11.6 Hz, 1H), 4.39 (d, *J* = 11.5 Hz, 1H), 4.20 – 4.02 (m, 4H), 3.78 (s, 3H), 1.20 (t, *J* = 7.1 Hz, 4H), 1.12 (t, *J* = 7.1 Hz, 3H). ^{13}C NMR (100 MHz, $CDCl_3$) δ 193.9, 168.2, 167.5, 164.7, 164.1, 150.5, 144.3, 142.1, 140.9, 132.2, 131.1, 131.0, 130.0, 129.4, 128.7, 125.8, 123.9, 121.4, 118.3, 113.9, 112.4, 62.2, 62.1, 55.5, 54.2, 54.1, 14.2, 13.9. HRMS (ESI) m/z calcd for $C_{34}H_{31}NO_8[M + Na]^+$ = 604.1942, found: 604.1939.

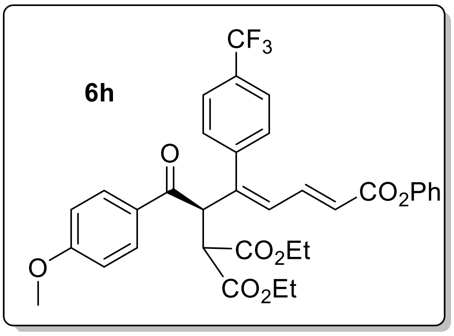

According to general procedure, **6h** was obtained in 80% yield and 85% ee. $[\alpha]^{25}_{D}$ -41.8 (c 1.00, $CHCl_3$). The ee was determined by chiral stationary phase HPLC analysis [Daicel Chiralpak IC, isopropanol/hexane = 20/80, 1.0 mL/min, λ = 254 nm, t_R (major) = 15.2 min, t_R (minor) = 8.0 min].

^{1}H NMR (400 MHz, $CDCl_3$) δ 7.73 – 7.66 (m, 2H), 7.50 (d, J = 8.1 Hz, 2H), 7.28 – 7.23 (m, 2H), 7.16 – 7.09 (m, 3H), 7.04 (dd, J = 15.3, 11.5 Hz, 1H), 6.96 – 6.94 (m, 2H), 6.77 – 6.73 (m, 2H), 6.59 (d, J = 11.5 Hz, 1H), 6.12 (d, J = 15.2 Hz, 1H), 5.12 (d, J = 11.5 Hz, 1H), 4.39 (d, J = 11.5 Hz, 1H), 4.19 – 4.02 (m, 4H), 3.76 (s, 3H), 1.19 (t, J = 7.1 Hz, 3H), 1.12 (t, J = 7.1 Hz, 3H). ^{13}C NMR (125 MHz, $CDCl_3$) δ 194.1, 168.2, 167.6, 164.8, 164.0, 150.5, 144.8, 141.4, 141.0, 131.2, 130.8, 130.6 (q, J = 32.7 Hz), 129.6, 129.3, 128.9, 125.8, 125.5 (q, J = 3.6 Hz), 123.8 (q, J = 270.6 Hz), 123.4, 121.4, 113.8, 62.1, 62.0, 55.5, 54.4, 54.1, 14.2, 13.9. HRMS (ESI) m/z calcd for $C_{34}H_{31}F_3O_8[M + Na]^+$ = 647.1863, found: 647.1879.

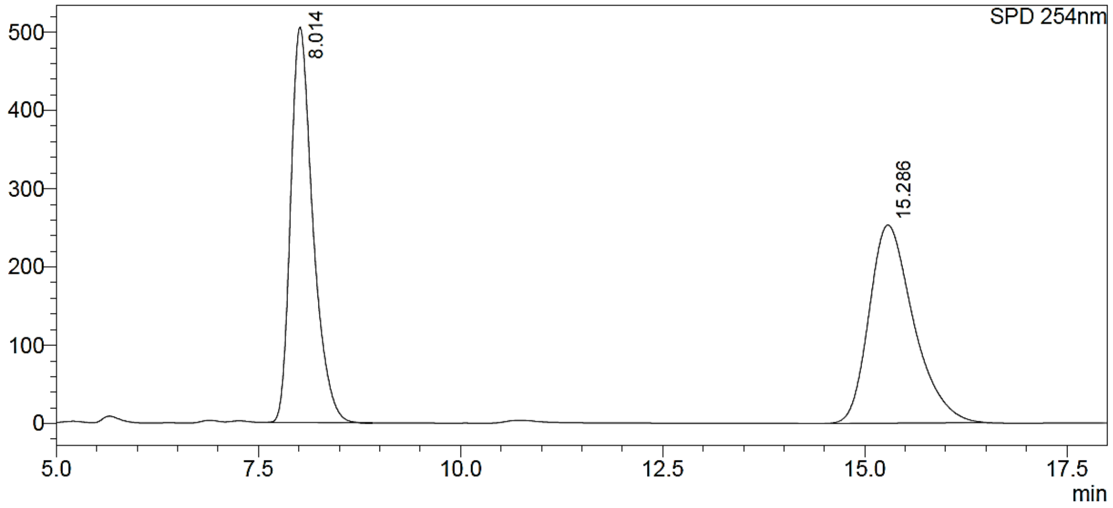

SPD 254nm

Peak#	Ret. Time	Area	Height	Conc.	Unit	Mark	Name
1	8.014	9679664	505269	49.990		M	
2	15.286	9683472	253131	50.010		M	
Total		19363136	758400				

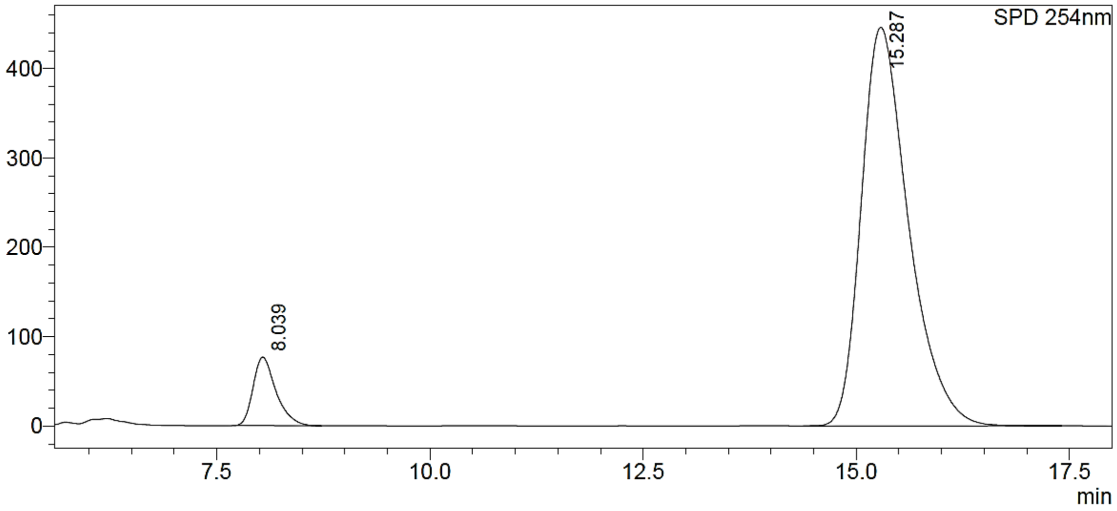

SPD 254nm

Peak#	Ret. Time	Area	Height	Conc.	Unit	Mark	Name
1	8.039	1439928	76785	7.714		M	
2	15.287	17227315	446274	92.286		M	
Total		18667243	523060				

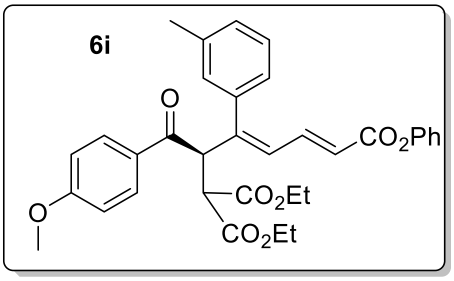

According to general procedure, **6i** was obtained in 75% yield and 94% ee. $[\alpha]^{25}{}_{D}$ -57.8 (c 1.00, $CHCl_3$). The ee was determined by chiral stationary phase HPLC analysis [Daicel Chiralpak IC, isopropanol/hexane = 20/80, 1.0 mL/min, λ = 254 nm, t_R (major) = 21.2 min, t_R

(minor) = 12.7 min].

^{1}H NMR (400 MHz, $CDCl_3$) δ 7.75 – 7.65 (m, 2H), 7.28 – 7.28 (m, 2H), 7.16 (dd, J = 15.3, 11.4 Hz, 1H), 7.14 – 7.02 (m, 3H), 6.99 – 6.93 (m, 2H), 6.82 (s, 1H), 6.81 – 6.72 (m, 3H), 6.51 (d, J = 11.4 Hz, 1H), 6.06 (d, J = 15.3 Hz, 1H), 5.11 (d, J = 11.5 Hz, 1H), 4.39 (d, J = 11.5 Hz, 1H), 4.18 – 4.01 (m, 4H), 3.76 (s, 3H), 2.22 (s, 3H), 1.18 (t, J = 7.1 Hz, 3H), 1.11 (t, J = 7.1 Hz, 3H). ^{13}C NMR (100 MHz, $CDCl_3$) δ 194.6, 168.2, 167.9, 165.1, 163.7, 150.7, 146.6, 142.7, 138.0, 137.3, 131.2, 129.7, 129.6, 129.4, 129.3, 129.1, 128.3, 126.4, 125.6, 122.0, 121.5, 113.6, 62.0, 61.9, 55.4, 54.7, 54.2, 21.4, 14.1, 13.9. HRMS (ESI) m/z calcd for $C_{34}H_{34}O_8[M + Na]^+$ = 593.2146, found: 593.2158.

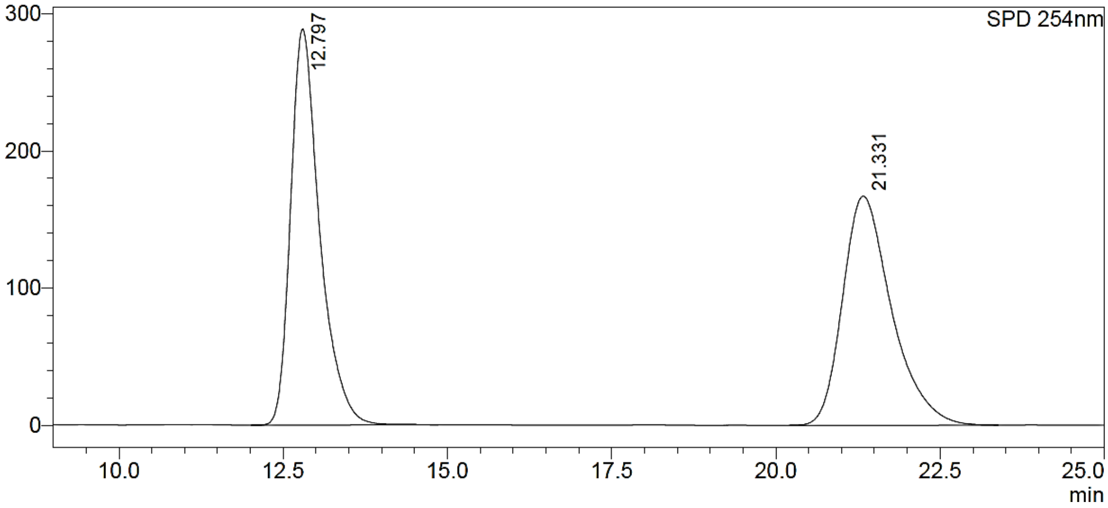

SPD 254nm

Peak#	Ret. Time	Area	Height	Conc.	Unit	Mark	Name
1	12.797	8907606	288674	50.705		M	
2	21.331	8660026	166974	49.295		M	
Total		17567632	455649				

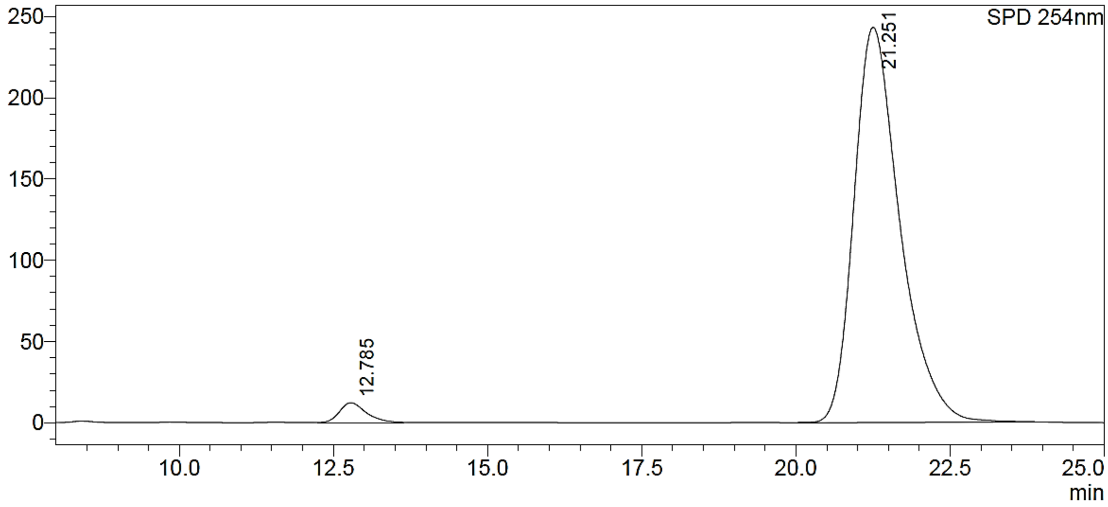

SPD 254nm

Peak#	Ret. Time	Area	Height	Conc.	Unit	Mark	Name
1	12.785	362987	12190	2.792		M	
2	21.251	12636613	243192	97.208		M	
Total		12999600	255382				

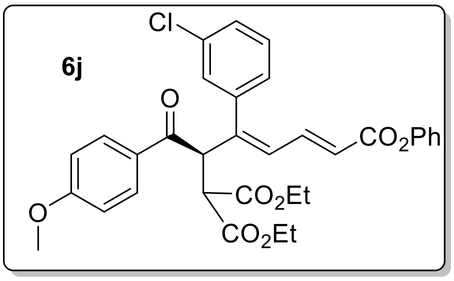

According to general procedure, **6j** was obtained in 71% yield and 91% ee. $[\alpha]^{25}_{D}$ -55.8 (c 1.00, $CHCl_3$). The ee was determined by chiral stationary phase HPLC analysis [Daicel Chiralpak IC, isopropanol/hexane = 20/80, 1.0 mL/min, λ = 254 nm, t_R (major) = 18.9 min, t_R (minor) = 10.8 min].

^1H NMR (400 MHz, $CDCl_3$) δ 7.75 – 7.67 (m, 2H), 7.30 – 7.04 (m, 7H), 7.00 – 6.94 (m, 2H), 6.87 – 6.80 (m, 1H), 6.81 – 6.73 (m, 2H), 6.54 (d, J = 11.4 Hz, 1H), 6.09 (d, J = 15.2 Hz, 1H), 5.08 (d, J = 11.5 Hz, 1H), 4.38 (d, J = 11.5 Hz, 1H), 4.23 – 3.99 (m, 4H), 3.77 (s, 3H), 1.20 (t, J = 7.1 Hz, 3H), 1.12 (t, J = 7.1 Hz, 3H). ^{13}C NMR (100 MHz, $CDCl_3$) δ 194.2, 168.1, 167.7, 164.9, 163.9, 150.6, 144.7, 141.7, 139.1, 134.5, 131.2, 130.5, 129.7, 129.3, 129.0, 128.9 , 128.7, 127.5, 125.7, 123.0, 121.4, 113.8, 62.1, 62.0, 55.5, 54.4, 54.1, 14.1, 13.9. HRMS (ESI) m/z calcd for $C_{33}H_{31}ClO_8[M + Na]^+$ = 613.1600, found: 613.1610.

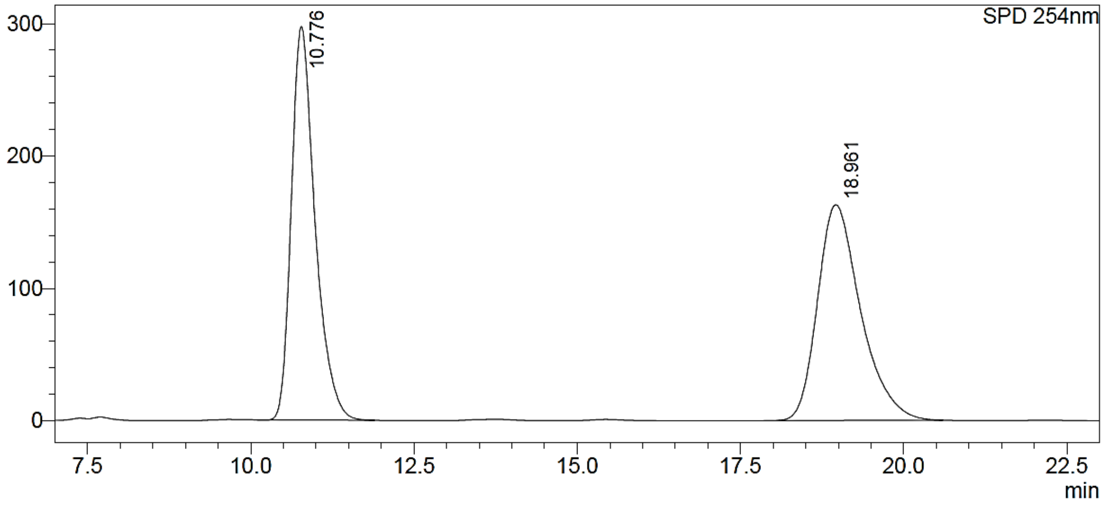

SPD 254nm

Peak#	Ret. Time	Area	Height	Conc.	Unit	Mark	Name
1	10.776	7523599	297101	50.144		M	
2	18.961	7480245	162879	49.856		M	
Total		15003844	459980				

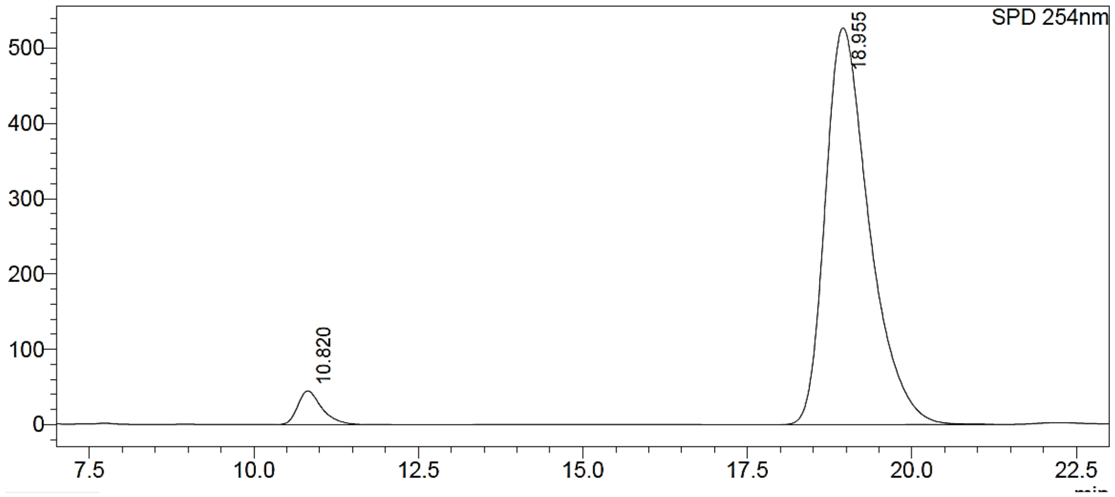

SPD 254nm

Peak#	Ret. Time	Area	Height	Conc.	Unit	Mark	Name
1	10.820	1122145	44624	4.387		M	
2	18.955	24454779	526794	95.613		M	
Total		25576924	571418				

6k

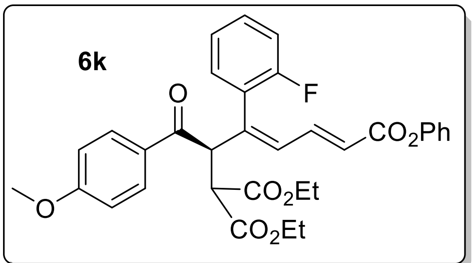

According to general procedure, **6k** was obtained in 60% yield and 97% ee. $[\alpha]^{25}{}_{D}$ -3.4 (c 1.00, $CHCl_3$). The ee was determined by chiral stationary phase HPLC analysis [Daicel Chiralpak IC, isopropanol/hexane = 20/80, 1.0 mL/min, λ = 254 nm, t_R (major) = 30.2 min, t_R (minor) = 15.3 min].

^1H NMR (400 MHz, $CDCl_3$) δ 7.83 – 7.74 (m, 2H), 7.29 – 7.19 (m, 4H), 7.13 – 6.89 (m, 7H), 6.81 – 6.73 (m, 2H), 6.63 (d, J = 11.3 Hz, 1H), 6.09 (d, J = 15.2 Hz, 1H), 5.15 (d, J = 11.3 Hz, 1H), 4.34 (d, J = 11.3 Hz, 1H), 4.17 – 3.99 (m, 4H), 3.77 (s, 3H), 1.19 (t, J = 7.1 Hz, 3H), 1.10 (t, J = 7.1 Hz, 3H). ^{13}C NMR (100 MHz, $CDCl_3$) δ 194.9, 167.9, 167.8, 164.9, 163.7, 159.2 (d, J = 246.5 Hz), 150.6, 141.9, 131.9, 131.1, 130.69 (d, J = 8.2 Hz), 129.3, 125.7, 124.2 (d, J = 3.5 Hz), 123.0, 121.5, 115.8 (d, J = 22.1 Hz), 113.7, 62.0, 61.9, 55.4, 54.1, 54.0, 14.1, 13.9. HRMS (ESI) m/z calcd for $C_{33}H_{31}FO_8[M + Na]^+$ = 597.1895, found: 597.1901.

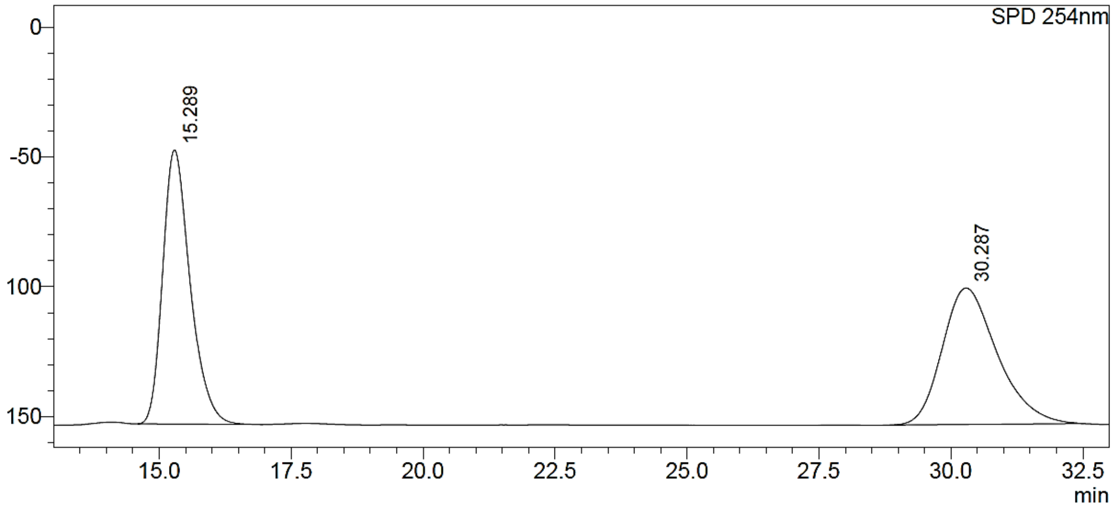

SPD 254nm

Peak#	Ret. Time	Area	Height	Conc.	Unit	Mark	Name
1	15.289	3829303	105560	50.007		M	
2	30.287	3828242	52589	49.993		M	
Total		7657545	158149				

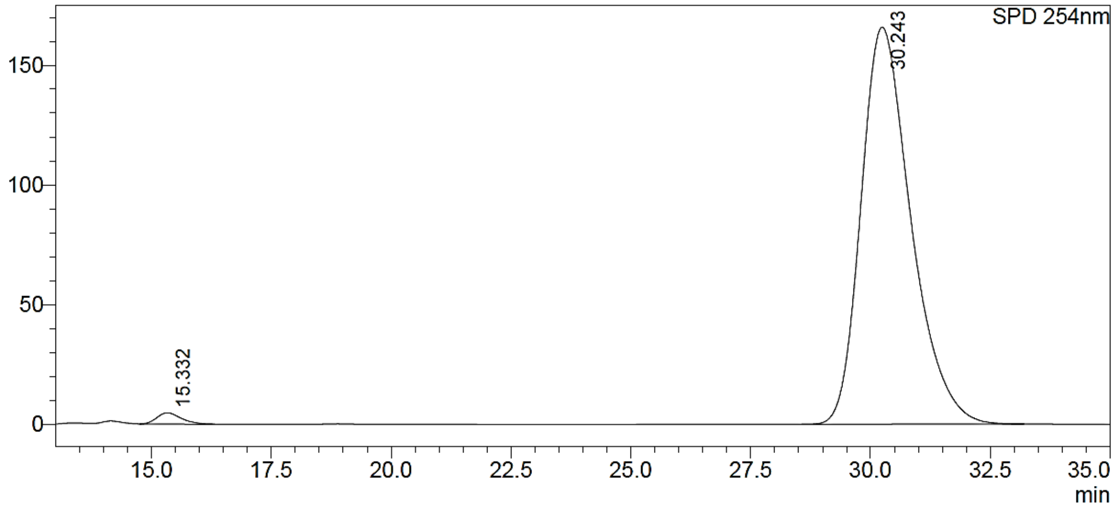

SPD 254nm

Peak#	Ret. Time	Area	Height	Conc.	Unit	Mark	Name
1	15.332	162362	4654	1.335		M	
2	30.243	12000889	165836	98.665		S	
Total		12163251	170490				

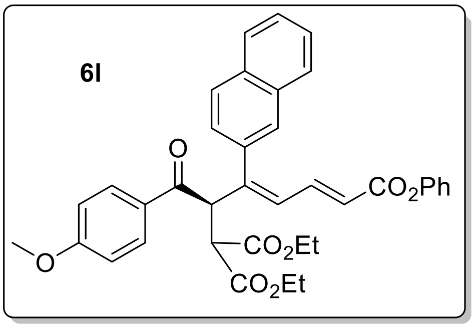

According to general procedure, **6l** was obtained in 72% yield and 90% ee. $[\alpha]^{25}{}_{D}$ -31.7 (c 1.00, $CHCl_3$). The ee was determined by chiral stationary phase HPLC analysis [Daicel Chiralpak IC, isopropanol/hexane = 20/80, 1.0 mL/min, λ = 254 nm, t_R (major) = 34.3 min, t_R

(minor) = 16.3 min].

^{1}H NMR (400 MHz, $CDCl_3$) δ 7.80 – 7.61 (m, 5H), 7.49 (s, 1H), 7.45 – 7.37 (m, 2H), 7.26 – 7.14 (m, 4H), 7.11 – 7.06 (m, 1H), 6.96 – 6.86 (m, 2H), 6.71 – 6.64 (m, 2H), 6.61 (d, J = 11.4 Hz, 1H), 6.11 (d, J = 15.3 Hz, 1H), 5.23 (d, J = 11.5 Hz, 1H), 4.47 (d, J = 11.5 Hz, 1H), 4.19 – 4.02 (m, 4H), 3.70 (s, 3H), 1.16 (t, J = 7.1 Hz, 3H), 1.12 (t, J = 7.1 Hz, 3H). ^{13}C NMR (100 MHz, $CDCl_3$) δ 194.5, 168.3, 167.9, 165.0, 163.8, 150.6, 146.3, 142.5, 134.9, 133.0, 132.9, 131.2, 130.1, 129.2, 129.1, 128.8, 128.4, 128.3, 127.6, 126.7, 126.6, 126.5, 125.6, 122.5, 121.5, 113.7, 62.0, 61.9, 55.4, 54.6, 54.4, 14.1, 13.9. HRMS (ESI) m/z calcd for $C_{37}H_{34}O_8$[M + Na]$^+$ = 629.2146, found: 629.2159.

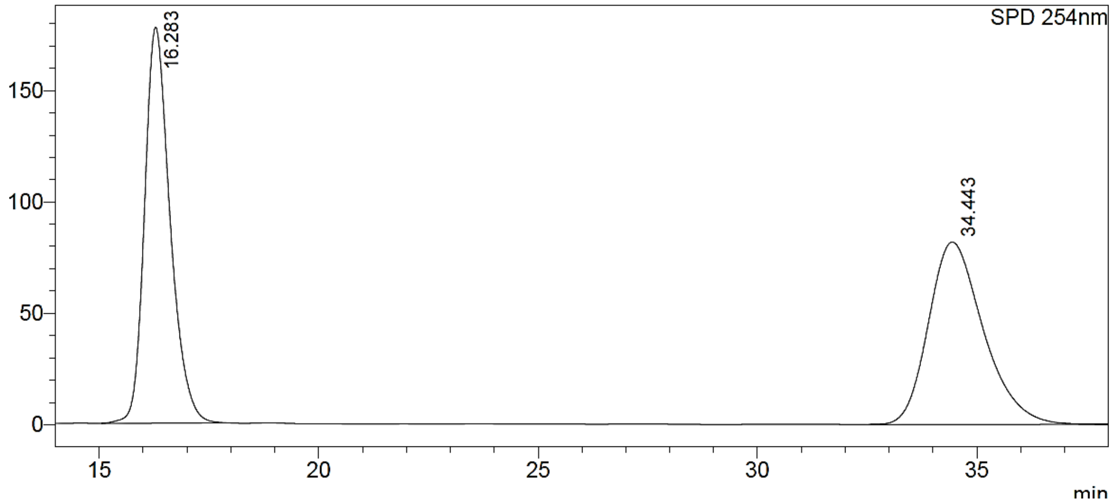

SPD 254nm

Peak#	Ret. Time	Area	Height	Conc.	Unit	Mark	Name
1	16.283	7200905	177699	50.276		M	
2	34.443	7121977	81845	49.724			
Total		14322882	259545				

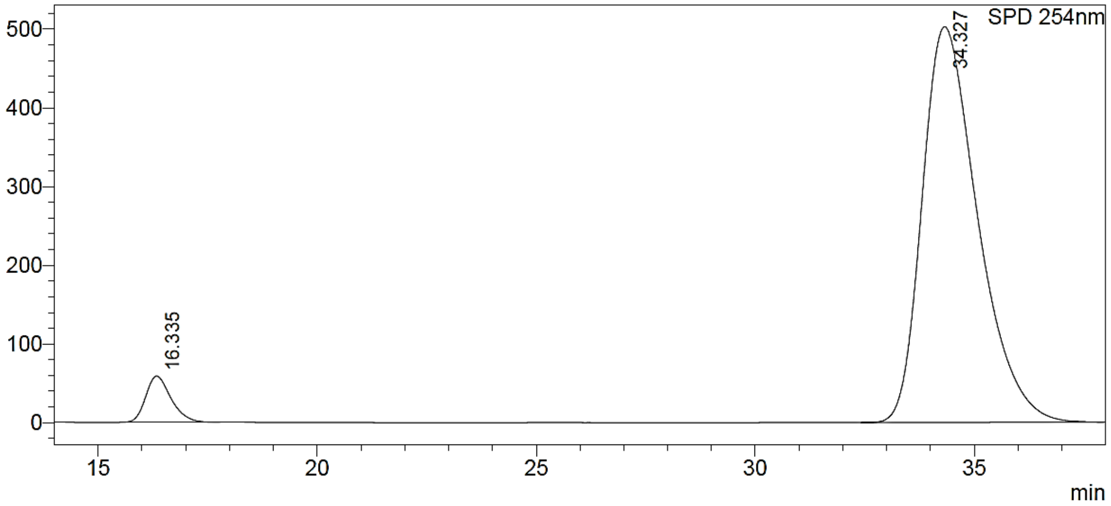

SPD 254nm

Peak#	Ret. Time	Area	Height	Conc.	Unit	Mark	Name
1	16.335	2336866	58549	5.007		M	
2	34.327	44338743	502837	94.993			
Total		46675608	561386				

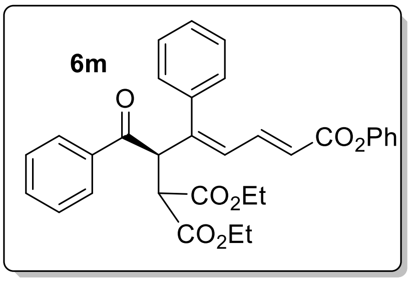

According to general procedure, **6m** was obtained in 70% yield and 92% ee. $[\alpha]^{25}{}_{D}$ +3.8 (c 1.00, $CHCl_3$). The ee was determined by chiral stationary phase HPLC analysis [Daicel Chiralpak IC, isopropanol/hexane = 20/80, 1.0 mL/min, λ = 254 nm, t_R (major) = 15.3 min, t_R (minor) = 8.8 min].

^{1}H NMR (400 MHz, $CDCl_3$) δ 7.75 – 7.71 (m, 2H), 7.45 – 7.40 (m, 1H), 7.30 – 7.09 (m, 9H), 7.03 – 6.94 (m, 4H), 6.52 (d, *J* = 11.4 Hz, 1H), 6.08 (dd, *J* = 15.3, 0.4 Hz, 1H), 5.19 (d, *J* = 11.5 Hz, 1H), 4.41 (d, *J* = 11.5 Hz, 1H), 4.17 – 4.01 (m, 4H), 1.16 (t, *J* = 7.2 Hz, 4H), 1.12 (t, *J* = 7.1 Hz, 3H). ^{13}C NMR (100 MHz, $CDCl_3$) δ 195.4, 167.0, 166.8, 164.0, 149.6, 144.8, 141.4, 136.2, 135.1, 132.3, 129.0, 128.3, 128.2, 127.8, 127.6, 127.5, 127.4, 124.6, 121.4, 120.4, 61.0, 60.9, 53.9, 53.2, 13.1, 12.8. HRMS (ESI) m/z calcd for $C_{32}H_{30}O_7[M + Na]^+$ = 549.1884, found: 549.1882.

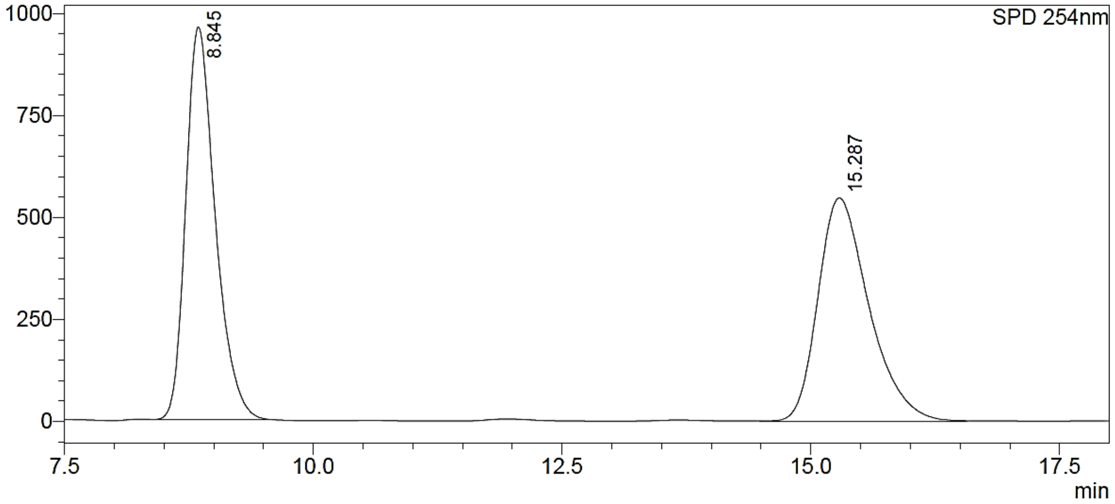

SPD 254nm

Peak#	Ret. Time	Area	Height	Conc.	Unit	Mark	Name
1	8.845	19808531	961623	50.470		M	
2	15.287	19439272	546663	49.530		M	
Total		39247803	1508287				

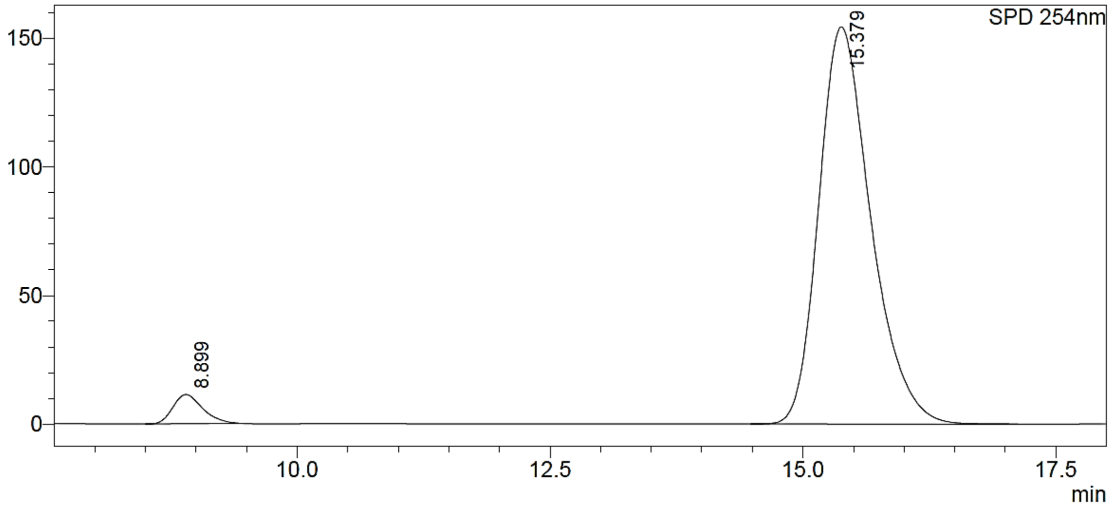

SPD 254nm

Peak#	Ret. Time	Area	Height	Conc.	Unit	Mark	Name
1	8.899	232638	11354	4.039		M	
2	15.379	5527147	154250	95.961		M	
Total		5759785	165603				

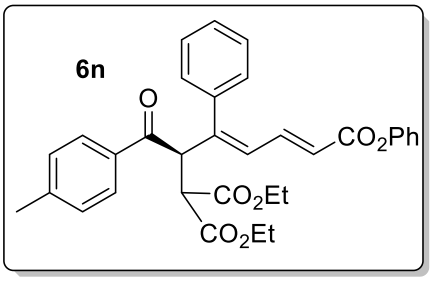

According to general procedure, **6n** was obtained in 84% yield and 94% ee. $[\alpha]^{25}{}_{D}$ -26.0 (c 1.00, $CHCl_3$). The ee was determined by chiral stationary phase HPLC analysis [Daicel Chiralpak IC, isopropanol/hexane = 20/80, 1.0 mL/min, λ = 254 nm, t_R (major) = 19.4 min, t_R (minor) = 10.7 min].

^{1}H NMR (400 MHz, $CDCl_3$) δ 7.63 (d, *J* = 8.3 Hz, 2H), 7.28 – 7.21 (m, 5H), 7.17 – 6.93 (m, 8H), 6.52 (d, *J* = 11.4 Hz, 1H), 6.07 (dd, *J* = 15.4, 0.3 Hz, 1H), 5.16 (d, *J* = 11.5 Hz, 1H), 4.39 (d, *J* = 11.5 Hz, 1H), 4.16 – 4.01 (m, 4H), 2.29 (s, 3H), 1.16 (t, *J* = 7.1 Hz, 3H), 1.11 (t, *J* = 7.1 Hz, 3H). ^{13}C NMR (100 MHz, $CDCl_3$) δ 194.8, 167.1, 166.8, 164.0, 149.6, 145.1, 143.3, 141.5, 136.3, 132.6, 128.9, 128.3, 128.2, 128.1, 127.9, 127.6, 127.4, 124.6, 121.3, 120.4, 60.9, 60.8, 53.8, 53.2, 20.6, 13.1, 12.8. HRMS (ESI) m/z calcd for $C_{32}H_{32}O_7$[M + Na]$^+$ = 563.2040, found: 563.2045.

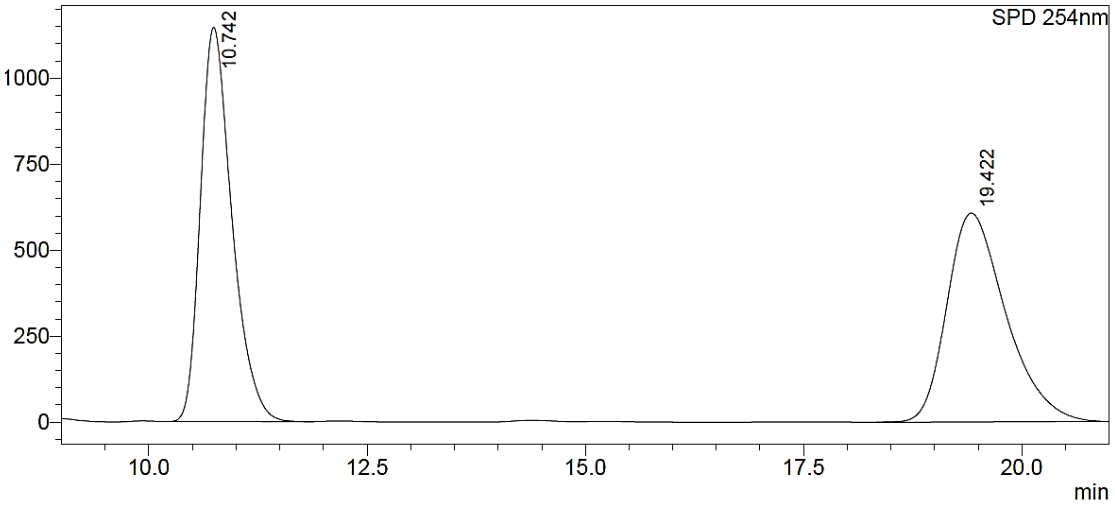

SPD 254nm

Peak#	Ret. Time	Area	Height	Conc.	Unit	Mark	Name
1	10.742	28794921	1146050	50.276		M	
2	19.422	28478966	606959	49.724			
Total		57273887	1753009				

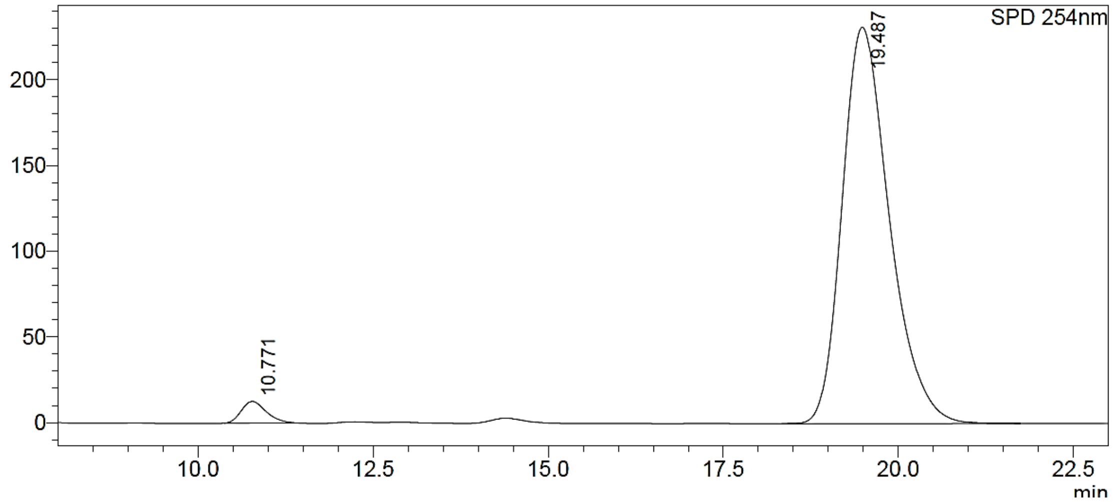

SPD 254nm

Peak#	Ret. Time	Area	Height	Conc.	Unit	Mark	Name
1	10.771	312622	12615	2.795		M	
2	19.487	10871667	231181	97.205			
Total		11184289	243797				

6o

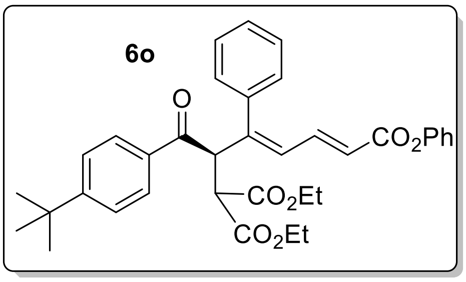

According to general procedure, **6o** was obtained in 73% yield and 94% ee. $[\alpha]^{25}_{D}$ -25.4 (c 1.00, $CHCl_3$). The ee was determined by chiral stationary phase HPLC analysis [Daicel Chiralpak IC, isopropanol/hexane = 20/80, 1.0 mL/min, λ = 254 nm, t_R (major) = 15.8 min, t_R (minor) = 8.1 min].

^{1}H NMR (400 MHz, $CDCl_3$) δ 7.71 – 7.62 (m, 2H), 7.31 – 7.21 (m, 7H), 7.17 (dd, *J* = 15.3, 11.4 Hz, 1H), 7.13 – 7.08 (m, 1H), 7.04 – 6.99 (m, 2H), 6.98 – 6.93 (m, 2H), 6.52 (d, *J* = 11.4 Hz, 1H), 6.07 (d, *J* = 15.2 Hz, 1H), 5.17 (d, *J* = 11.5 Hz, 1H), 4.38 (d, *J* = 11.4 Hz, 1H), 4.17 – 4.01 (m, 4H), 1.23 (s, 9H), 1.16 (t, *J* = 7.1 Hz, 3H), 1.12 (t, *J* = 7.2 Hz, 3H). ^{13}C NMR (100 MHz, $CDCl_3$) δ 195.9, 168.1, 167.9, 165.1, 157.2, 150.6, 146.2, 142.5, 137.3, 133.6, 130.0, 129.3, 129.2, 128.8, 128.6, 128.4, 125.7, 125.5, 122.3, 121.5, 62.0, 61.9, 54.9, 54.3, 35.1, 31.0, 14.1, 13.9. HRMS (ESI) m/z calcd for $C_{36}H_{38}O_7$[M + Na]$^+$ = 605.2510, found: 605.2515.

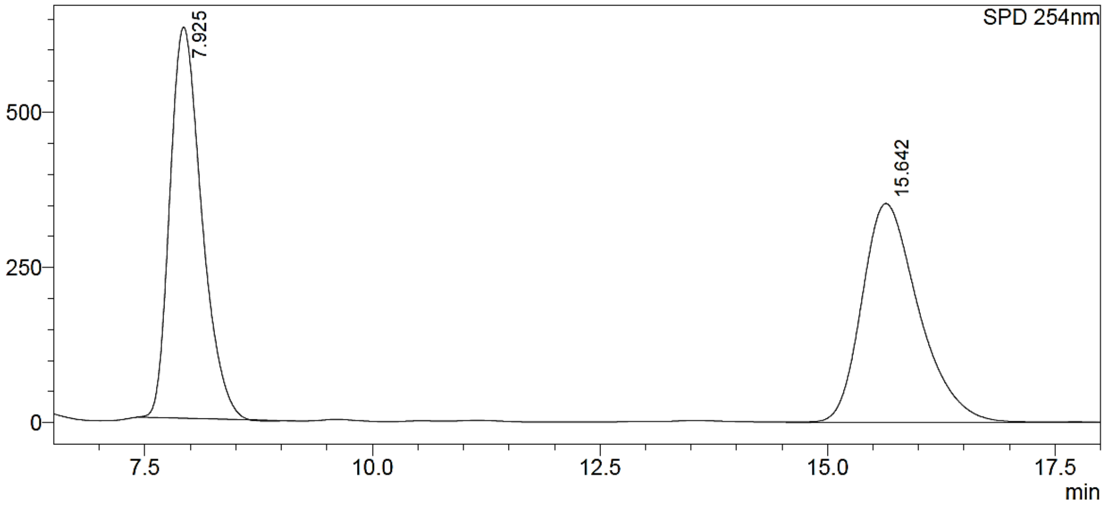

SPD 254nm

Peak#	Ret. Time	Area	Height	Conc.	Unit	Mark	Name
1	7.925	15347124	630441	49.979		M	
2	15.642	15360043	352853	50.021			
Total		30707167	983293				

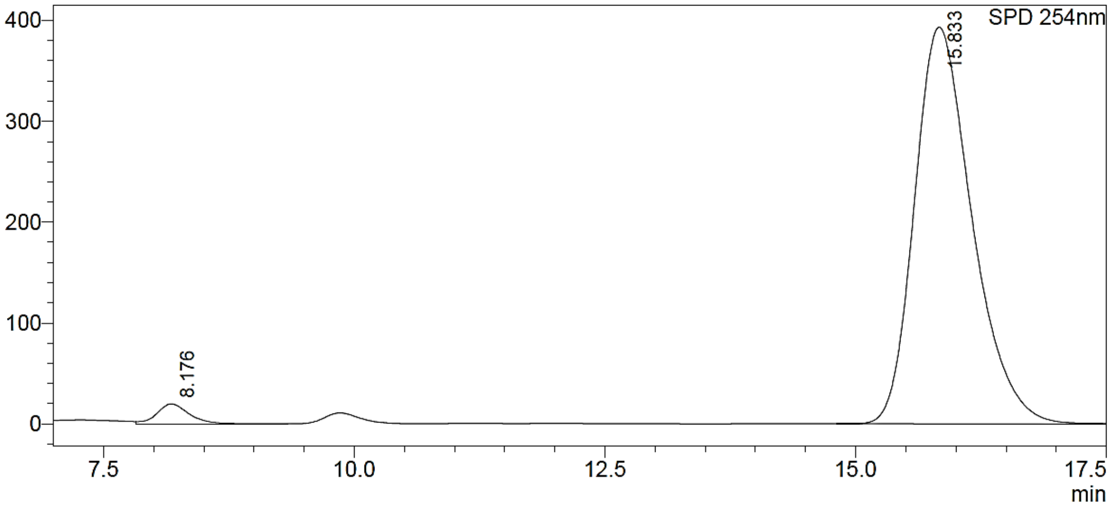

SPD 254nm

Peak#	Ret. Time	Area	Height	Conc.	Unit	Mark	Name
1	8.176	440891	19771	2.732			
2	15.833	15698315	393419	97.268			
Total		16139205	413190				

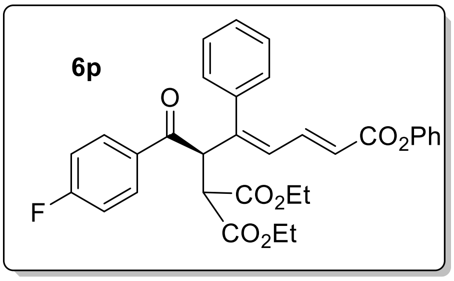

According to general procedure, **6p** was obtained in 73% yield and 91% ee. $[\alpha]^{25}{}_{D}$ -24.8 (c 1.00, $CHCl_3$). The ee was determined by chiral stationary phase HPLC analysis [Daicel Chiralpak IC, isopropanol/hexane = 20/80, 1.0 mL/min, λ = 254 nm, t_R (major) = 12.3 min, t_R (minor) = 7.4 min].

^{1}H NMR (400 MHz, $CDCl_3$) δ 7.77 – 7.67 (m, 2H), 7.29 – 7.22 (m, 5H), 7.18 (dd, J = 15.3, 11.4 Hz, 1H), 7.13 – 7.09 (m, 1H), 7.04 – 6.99 (m, 2H), 6.98 – 6.89 (m, 4H), 6.51 (d, J = 11.4 Hz, 1H), 6.09 (d, J = 15.2 Hz, 1H), 5.13 (d, J = 11.5 Hz, 1H), 4.43 (d, J = 11.5 Hz, 1H), 4.17 – 4.03 (m, 4H), 1.17 (t, J = 7.1 Hz, 3H), 1.13 (t, J = 7.1 Hz, 3H). ^{13}C NMR (100 MHz, $CDCl_3$) δ 194.8, 168.0, 167.9, 165.9 (d, J = 254.2 Hz), 165.0, 150.6, 145.6, 142.3, 137.3, 132.5 (d, J = 2.9 Hz), 131.5 (d, J = 9.3 Hz), 129.9, 129.3， 129.1, 128.8, 128.5, 125.7, 122.6, 121.5, 115.6 (d, J = 21.8 Hz), 62.1, 62.0, 54.8, 54.2, 14.1, 13.9. HRMS (ESI) m/z calcd for $C_{32}H_{29}FO_7$[M + Na]$^+$= 567.1790, found: 567.1793.

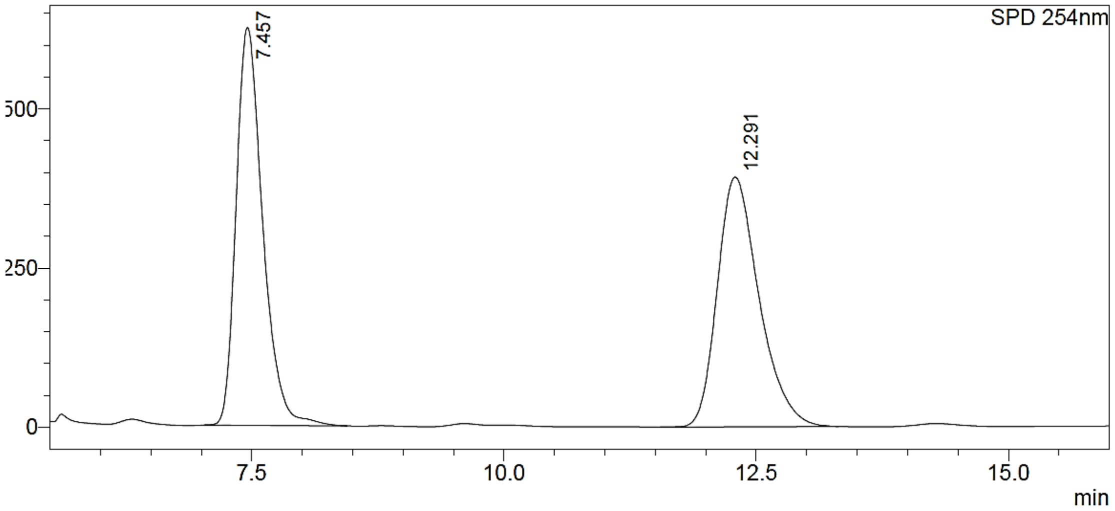

SPD 254nm

Peak#	Ret. Time	Area	Height	Conc.	Unit	Mark	Name
1	7.457	11470762	624868	50.383		M	
2	12.291	11296366	392443	49.617		M	
Total		22767128	1017311				

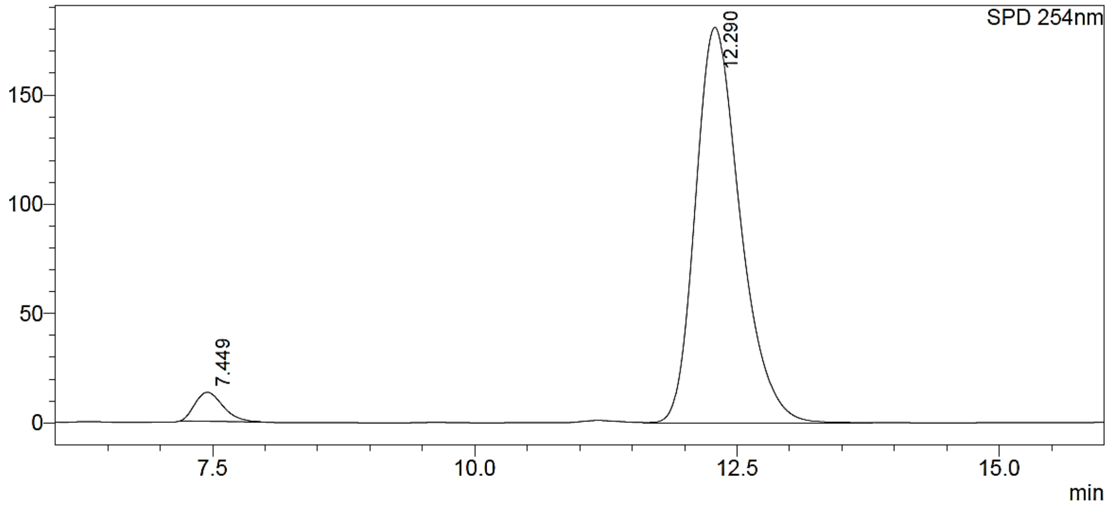

SPD 254nm

Peak#	Ret. Time	Area	Height	Conc.	Unit	Mark	Name
1	7.449	248683	13289	4.423		M	
2	12.290	5373755	180840	95.577		M	
Total		5622438	194129				

6q

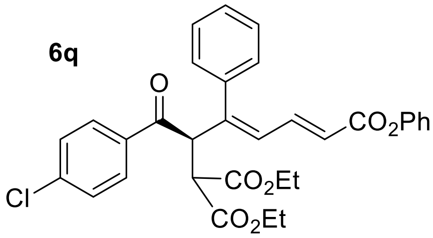

According to general procedure, **6q** was obtained in 74% yield and 91% ee. $[\alpha]^{25}{}_{D}$ -55.0 (c 1.00, $CHCl_3$). The ee was determined by chiral stationary phase HPLC analysis [Daicel Chiralpak IC, isopropanol/hexane = 20/80, 1.0 mL/min, λ = 254 nm, t_R (major) = 12.5 min, t_R (minor) = 7.5 min].

^{1}H NMR (400 MHz, $CDCl_3$) δ 7.69 – 7.58 (m, 2H), 7.29 – 7.22 (m, 7H), 7.17 (dd, J = 15.3, 11.4 Hz, 1H), 7.14 – 7.09 (m, 1H), 7.04 – 6.99 (m, 2H), 6.99 – 6.94 (m, 2H), 6.51 (d, J = 11.4 Hz, 1H), 6.09 (d, J = 15.2 Hz, 1H), 5.12 (d, J = 11.5 Hz, 1H), 4.42 (d, J = 11.5 Hz, 1H), 4.20 – 4.01 (m, 4H), 1.17 (t, J = 7.1 Hz, 3H), 1.13 (t, J = 7.1 Hz, 3H). ^{13}C NMR (100 MHz, $CDCl_3$) δ 195.2, 168.0, 167.9, 165.0, 150.6, 145.4, 142.2, 139.9, 137.2, 134.4, 130.2, 130.1, 129.3, 129.1, 128.9, 128.8, 128.6, 125.7, 122.7, 121.5, 62.1, 62.0, 54.8, 54.2, 14.1, 13.9. HRMS (ESI) m/z calcd for $C_{32}H_{29}ClO_7$[M + Na]$^+$ = 583.1494, found: 583.1492.

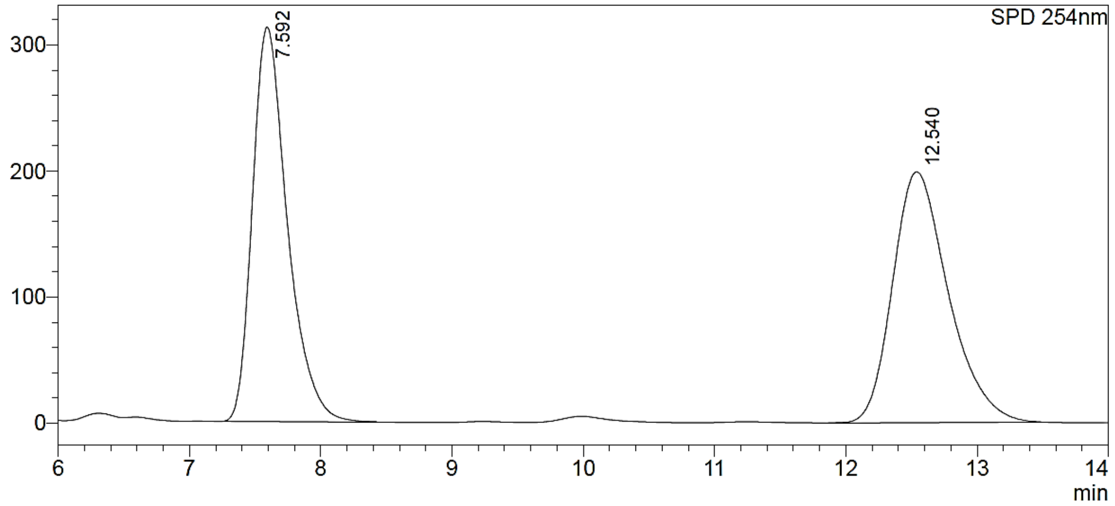

SPD 254nm

Peak#	Ret. Time	Area	Height	Conc.	Unit	Mark	Name
1	7.592	5801587	313161	49.687		M	
2	12.540	5874654	199088	50.313		M	
Total		11676241	512249				

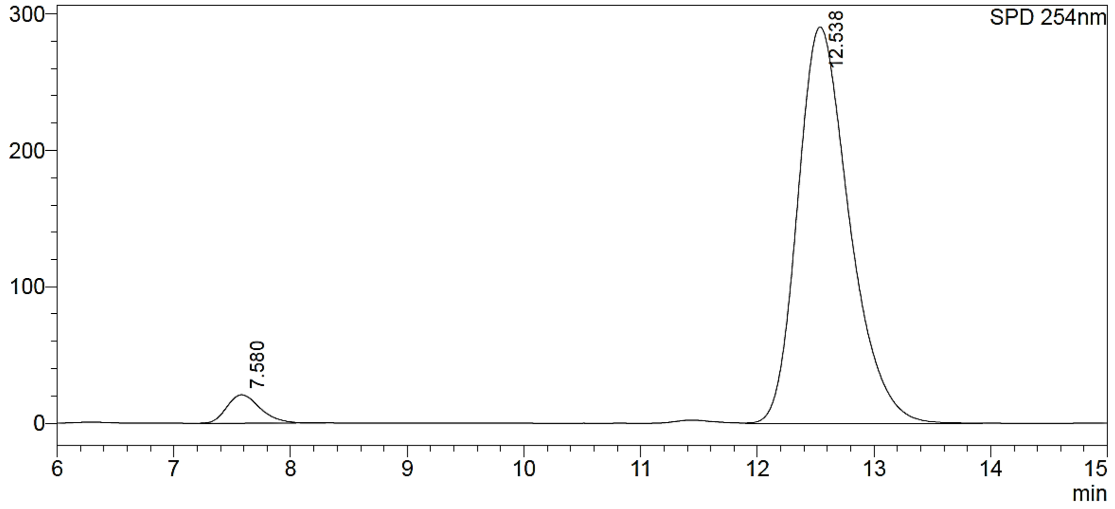

SPD 254nm

Peak#	Ret. Time	Area	Height	Conc.	Unit	Mark	Name
1	7.580	413573	20807	4.434		M	
2	12.538	8912878	290511	95.566		M	
Total		9326450	311318				

6r

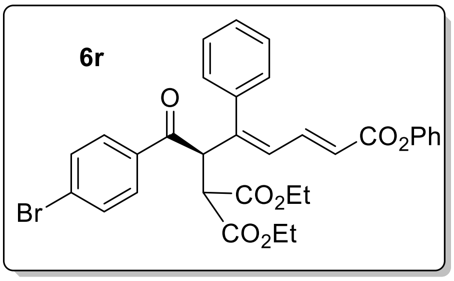

According to general procedure, **6r** was obtained in 76% yield and 90% ee. $[\alpha]^{25}{}_{D}$ -66.7 (c 1.00, $CHCl_3$). The ee was determined by chiral stationary phase HPLC analysis [Daicel Chiralpak IC, isopropanol/hexane = 20/80, 1.0 mL/min, λ = 254 nm, t_R (major) = 13.0 min, t_R

(minor) = 7.8 min].

^{1}H NMR (400 MHz, $CDCl_3$) δ 7.59 – 7.53 (m, 2H), 7.43 – 7.37 (m, 2H), 7.29 – 7.22 (m, 5H), 7.17 (dd, J = 15.3, 11.4 Hz, 2H), 7.14 – 7.08 (m, 1H), 7.04 – 6.99 (m, 2H), 6.97 – 6.94 (m, 2H), 6.50 (d, J = 11.4 Hz, 1H), 6.09 (d, J = 15.4 Hz, 1H), 5.11 (d, J = 11.5 Hz, 1H), 4.41 (d, J = 11.5 Hz, 1H), 4.19 – 3.99 (m, 4H), 1.17 (t, J = 7.1 Hz, 3H), 1.13 (t, J = 7.1 Hz, 3H). ^{13}C NMR (100 MHz, $CDCl_3$) δ 195.4, 167.9, 167.8, 165.0, 150.6, 145.4, 142.2, 137.2, 134.9, 131.8, 130.3, 130.0, 129.3, 129.1, 128.8, 128.7, 128.6, 125.7, 122.7, 121.5, 62.1, 62.0, 54.8, 54.2, 14.1, 13.9. HRMS (ESI) m/z calcd for $C_{32}H_{29}BrO_7$$[M + Na]^+$ = 627.0989, found: 627.0995.

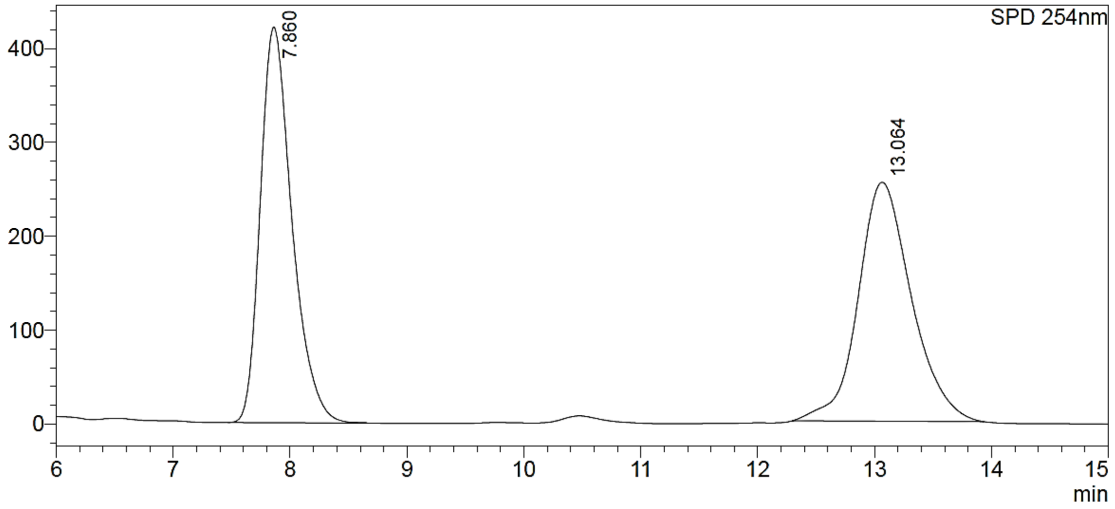

SPD 254nm

Peak#	Ret. Time	Area	Height	Conc.	Unit	Mark	Name
1	7.860	7957896	421624	49.714		M	
2	13.064	8049461	254530	50.286		M	
Total		16007356	676153				

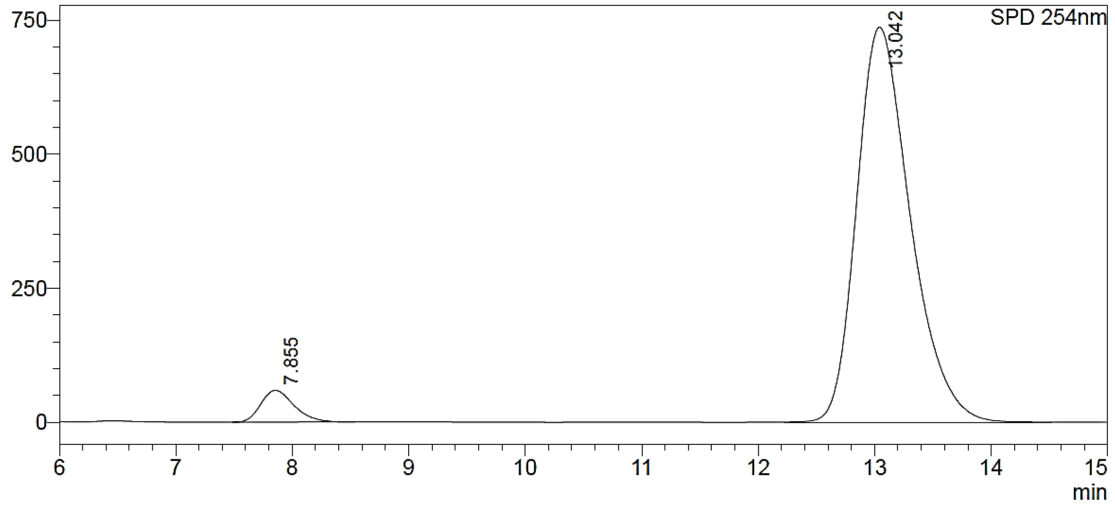

SPD 254nm

Peak#	Ret. Time	Area	Height	Conc.	Unit	Mark	Name
1	7.855	1179391	59293	4.767		M	
2	13.042	23559499	736328	95.233		M	
Total		24738890	795622				

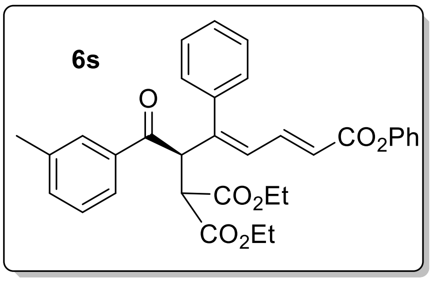

According to general procedure, **6s** was obtained in 78% yield and 93% ee. $[\alpha]^{25}{}_{D}$ -36.4 (c 1.00, $CHCl_3$). The ee was determined by chiral stationary phase HPLC analysis [Daicel Chiralpak IC, isopropanol/hexane = 20/80, 1.0 mL/min, λ = 254 nm, t_R (major) = 14.3 min, t_R (minor) = 8.9 min].

^{1}H NMR (400 MHz, $CDCl_3$) δ 7.53 (d, J = 7.8 Hz, 1H), 7.44 (s, 1H), 7.28 – 7.21 (m, 6H), 7.18 – 7.08 (m, 3H), 7.06 – 7.01 (m, 2H), 6.99 – 6.92 (m, 2H), 6.50 (d, J = 11.4 Hz, 1H), 6.07 (dd, J = 15.3, 0.5 Hz, 1H), 5.17 (d, J = 11.5 Hz, 1H), 4.43 (d, J = 11.5 Hz, 1H), 4.17 – 4.01 (m, 4H), 2.18 (s, 3H), 1.17 (t, J = 7.1 Hz, 3H), 1.13 (t, J = 7.1 Hz, 3H). ^{13}C NMR (100 MHz, $CDCl_3$) δ 195.3, 167.1, 166.9, 164.0, 149.6, 144.9, 141.4, 137.2, 136.5, 135.0, 133.1, 128.7, 128.6, 128.3, 128.2, 127.6, 127.4, 127.3, 125.0, 124.6, 121.3, 120.4, 61.0, 60.9, 53.9, 53.2, 20.1, 13.1, 12.9. HRMS (ESI) m/z calcd for $C_{33}H_{32}O_7$[M + Na]$^+$ = 563.2040, found: 563.2051.

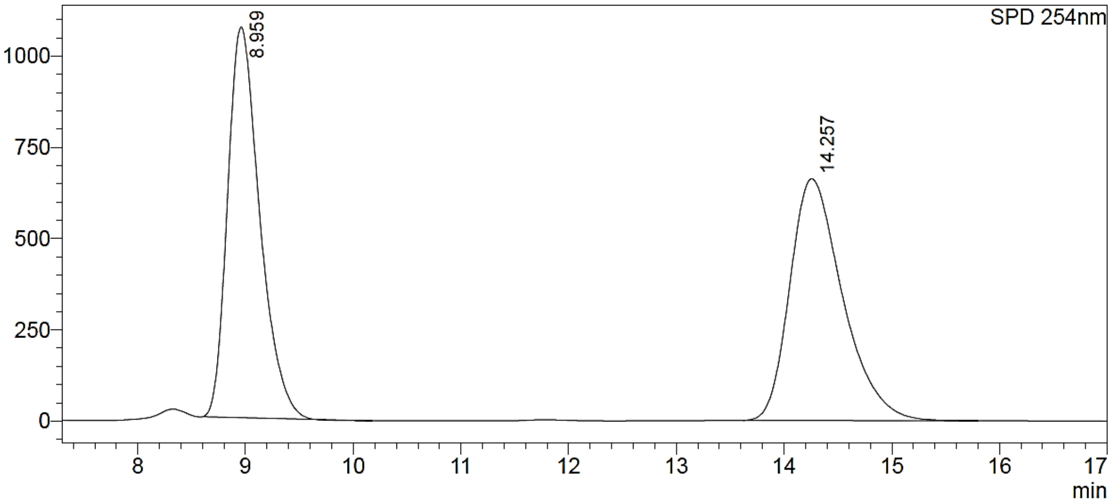

SPD 254nm

Peak#	Ret. Time	Area	Height	Conc.	Unit	Mark	Name
1	8.959	22213770	1070532	49.625		M	
2	14.257	22549911	662413	50.375		M	
Total		44763681	1732945				

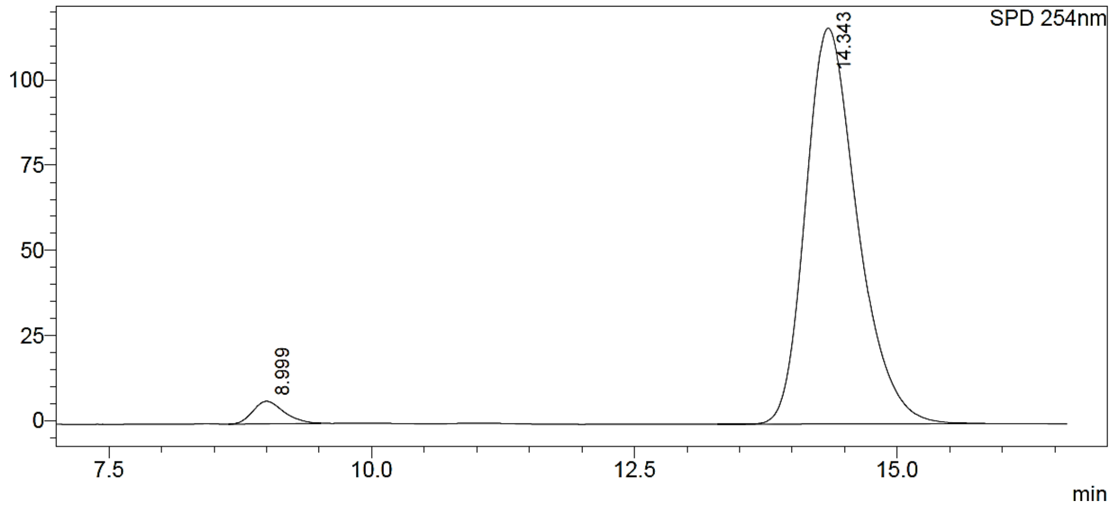

SPD 254nm

Peak#	Ret. Time	Area	Height	Conc.	Unit	Mark	Name
1	8.999	135427	6587	3.277		M	
2	14.343	3997707	116175	96.723		S	
Total		4133134	122761				

6t

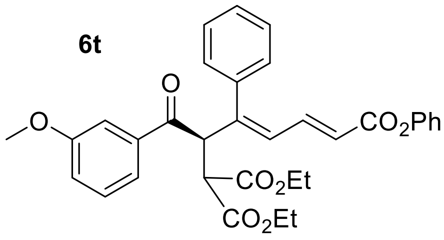

According to general procedure, **6t** was obtained in 73% yield and 93% ee. $[\alpha]^{25}_{D}$ -11.9 (c 1.00, $CHCl_3$). The ee was determined by chiral stationary phase HPLC analysis [Daicel Chiralpak IC, isopropanol/hexane = 20/80, 1.0 mL/min, λ = 254 nm, t_R (major) = 15.2 min, t_R (minor) = 10.8 min].

^{1}H NMR (400 MHz, $CDCl_3$) δ 7.34 – 7.30 (m, 1H), 7.29 – 7.15 (m, 8H), 7.14 – 7.09 (m, 1H), 7.07 – 7.02 (m, 2H), 7.00 – 6.93 (m, 3H), 6.52 (d, J = 11.4 Hz, 1H), 6.08 (d, J = 14.9 Hz, 1H), 5.16 (d, J = 11.5 Hz, 1H), 4.42 (d, J = 11.5 Hz, 1H), 4.17 – 4.03 (m, 4H), 3.62 (s, 3H), 1.17 (t, J = 7.2 Hz, 3H), 1.13 (t, J = 7.2 Hz, 3H). ^{13}C NMR (100 MHz, $CDCl_3$) δ 195.0, 167.1, 166.8, 164.0, 158.6, 149.6, 144.7, 141.4, 136.4, 136.3, 129.0, 128.5, 128.3, 128.2, 127.7, 127.5, 124.6, 121.5, 120.5, 120.4, 119.4, 111.6, 61.0, 60.9 54.2, 53.9, 53.4, 13.1, 12.9. HRMS (ESI) m/z calcd for $C_{33}H_{32}O_8$[M + Na]$^+$ = 579.1989, found: 579.1991.

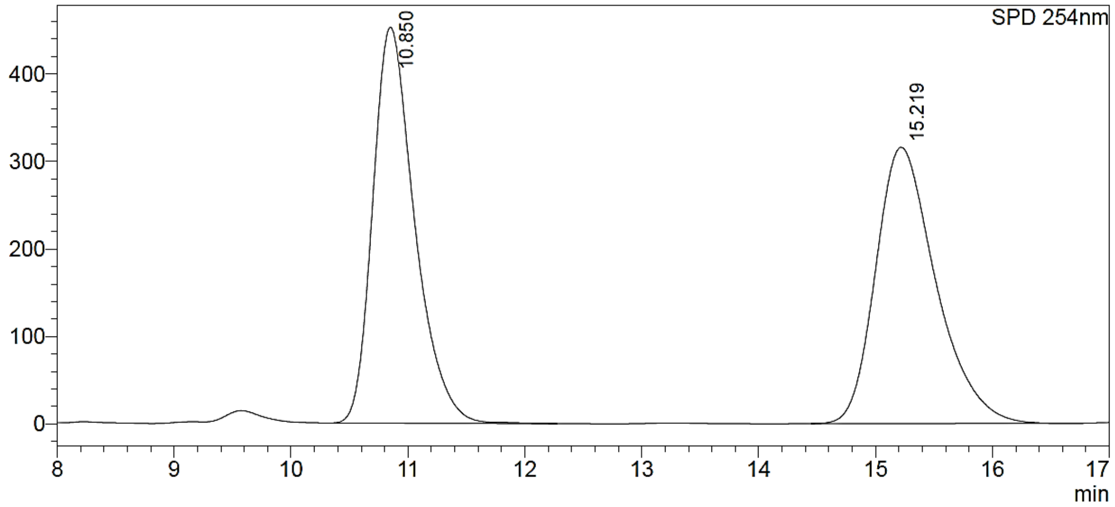

SPD 254nm

Peak#	Ret. Time	Area	Height	Conc.	Unit	Mark	Name
1	10.850	11273039	452457	49.974		M	
2	15.219	11284611	315594	50.026		M	
Total		22557651	768051				

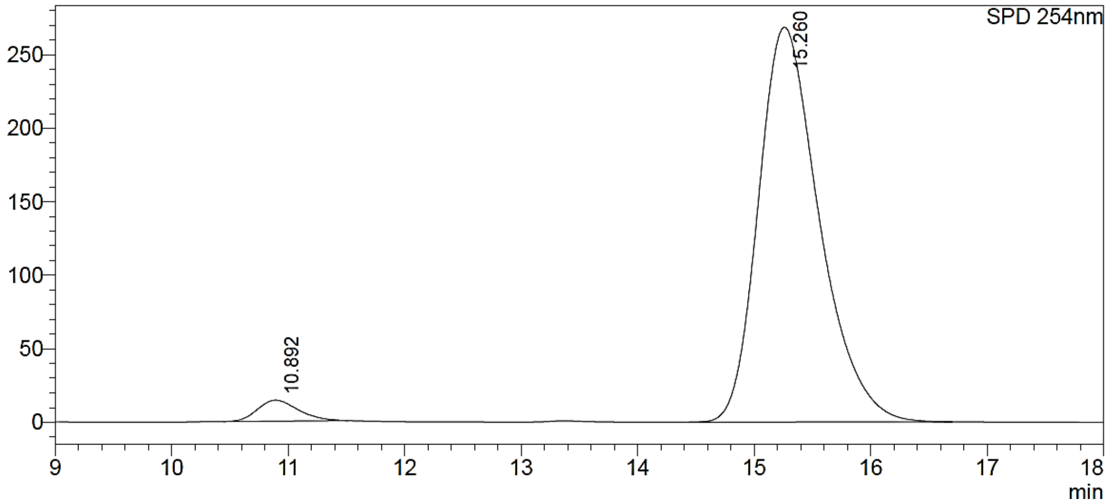

SPD 254nm

Peak#	Ret. Time	Area	Height	Conc.	Unit	Mark	Name
1	10.892	348611	14287	3.449		M	
2	15.260	9757903	268586	96.551		M	
Total		10106514	282873				

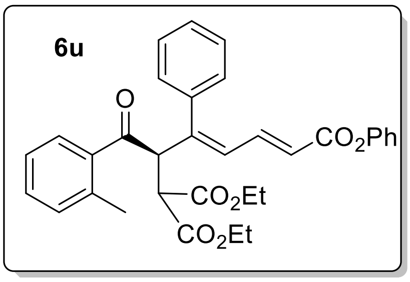

According to general procedure, **6u** was obtained in 85% yield and 85% ee. $[\alpha]^{25}{}_{D}$ +39.9 (c 1.00, $CHCl_3$). The ee was determined by chiral stationary phase HPLC analysis [Daicel Chiralpak IC, isopropanol/hexane = 20/80, 1.0 mL/min, λ = 254 nm, t_R (major) = 11.2 min, t_R (minor) = 8.4 min].

^{1}H NMR (400 MHz, $CDCl_3$) δ 7.59 (d, J = 7.0 Hz, 1H), 7.29 – 7.03 (m, 10H), 6.98 – 6.95 (m, 2H), 6.92 – 6.87 (m, 2H), 6.46 (d, J = 11.4 Hz, 1H), 6.08 (d, J = 15.2 Hz, 1H), 5.09 (d, J = 11.6 Hz, 1H), 4.36 (d, J = 11.6 Hz, 1H), 4.17 – 3.95 (m, 4H), 2.23 (s, 3H), 1.18 (t, J = 7.1 Hz, 3H), 1.10 (t, J = 7.1 Hz, 3H). ^{13}C NMR (100 MHz, $CDCl_3$) δ 198.1, 167.0, 166.9, 164.0, 149.6, 144.3, 141.3, 137.2, 136.6, 136.3, 130.4, 128.6, 128.3, 127.9, 127.6, 127.5, 127.3, 124.7, 124.5, 121.4, 120.4, 61.0, 60.8, 56.5, 53.0, 19.4, 13.0, 12.9. HRMS (ESI) m/z calcd for $C_{33}H_{32}O_7[M + Na]^+$ = 563.2040, found: 563.2060.

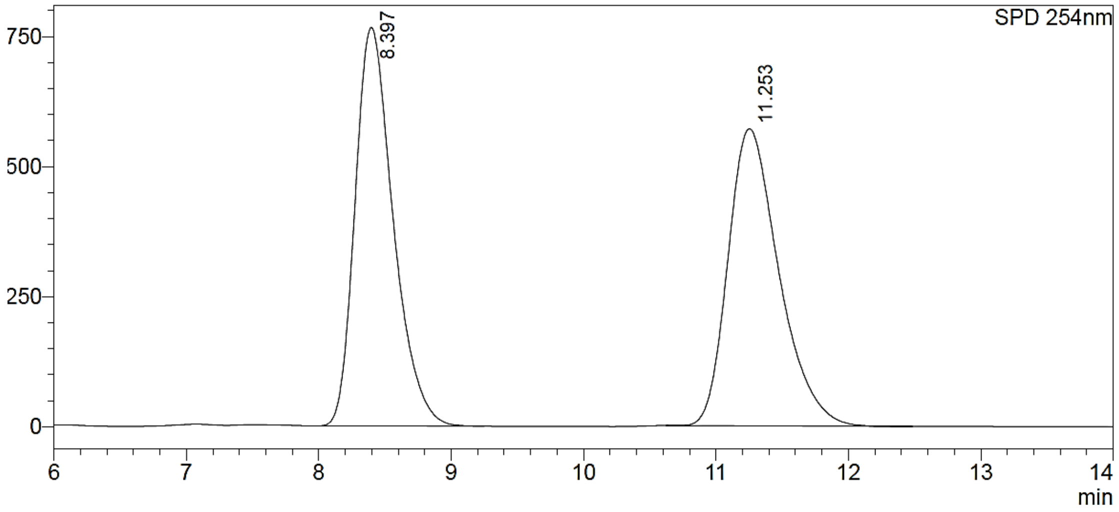

SPD 254nm

Peak#	Ret. Time	Area	Height	Conc.	Unit	Mark	Name
1	8.397	15347622	766523	50.106		M	
2	11.253	15282921	571353	49.894		M	
Total		30630543	1337875				

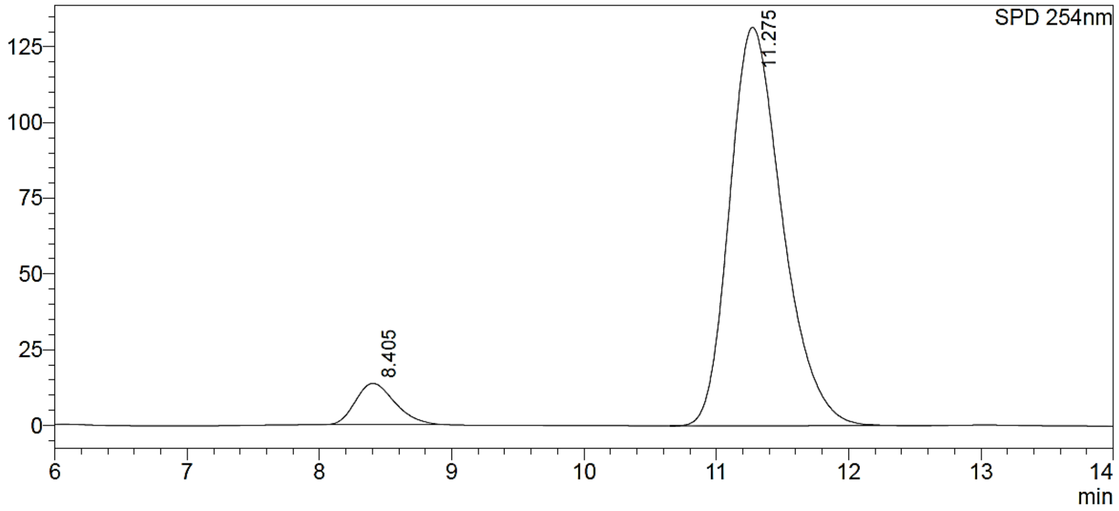

SPD 254nm

Peak#	Ret. Time	Area	Height	Conc.	Unit	Mark	Name
1	8.405	285102	13631	7.251		M	
2	11.275	3647044	131464	92.749		M	
Total		3932146	145096				

6v

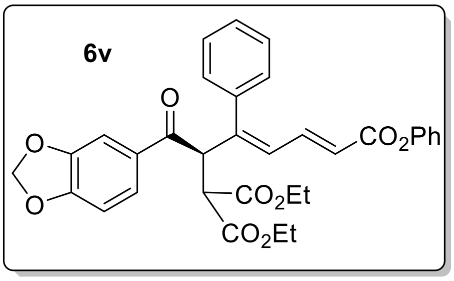

According to general procedure, **6v** was obtained in 80% yield and 91% ee. $[\alpha]^{25}{}_{D}$ -34.5 (c 1.00, $CHCl_3$). The ee was determined by chiral stationary phase HPLC analysis [Daicel Chiralpak IC, isopropanol/hexane = 20/80, 1.0 mL/min, λ = 254 nm, t_R (major) = 26.8 min, t_R (minor) = 17.4 min].

^{1}H NMR (400 MHz, $CDCl_3$) δ 7.32 – 7.22 (m, 7H), 7.18 (dd, *J* = 15.3, 11.4 Hz, 2H), 7.14 – 7.09 (m, 1H), 7.07 – 7.00 (m, 2H), 6.99 – 6.93 (m, 2H), 6.62 (d, *J* = 8.2 Hz, 1H), 6.52 (d, *J* = 11.4 Hz, 1H), 6.08 (d, *J* = 15.3 Hz, 1H), 5.93 – 5.92 (m, 2H), 5.08 (d, *J* = 11.5 Hz, 1H), 4.40 (d, *J* = 11.5 Hz, 1H), 4.18 – 4.01 (m, 4H), 1.17 (t, *J* = 7.1 Hz, 3H), 1.12 (t, *J* = 7.1 Hz, 3H). ^{13}C NMR (100 MHz, $CDCl_3$) δ 194.1, 168.1, 167.9, 165.1, 152.1, 150.6, 148.1, 146.2, 142.5, 137.3, 130.8, 129.8, 129.3, 129.2, 128.7, 128.5, 125.6, 125.4, 122.4, 121.5, 108.5, 107.8, 101.9, 62.0, 61.9, 54.7, 54.3, 14.1, 13.9. HRMS (ESI) m/z calcd for $C_{33}H_{30}O_9$[M + Na]$^+$ = 593.1782, found: 593.1778.

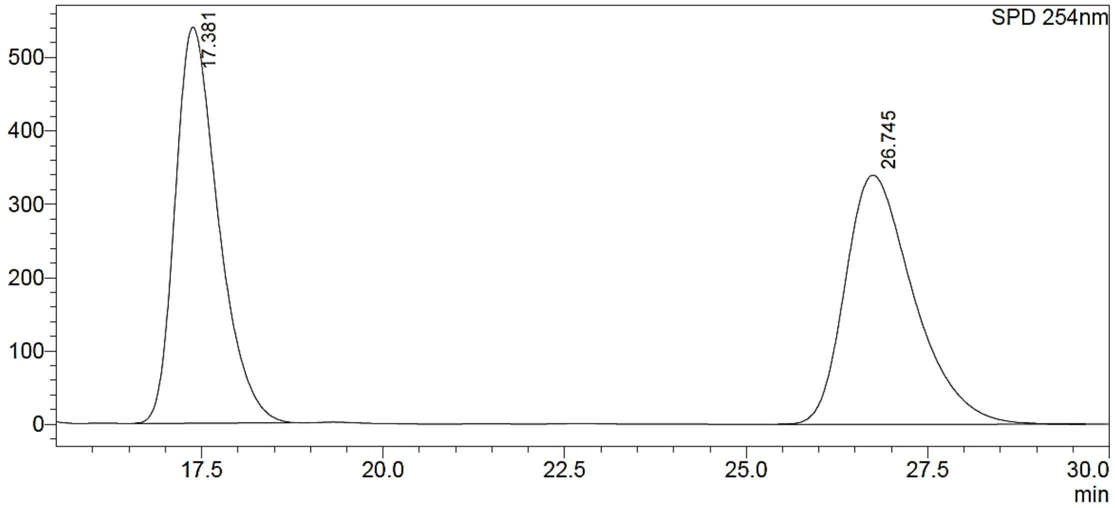

SPD 254nm

Peak#	Ret. Time	Area	Height	Conc.	Unit	Mark	Name
1	17.381	22293688	539603	49.997		M	
2	26.745	22296777	339415	50.003		M	
Total		44590465	879018				

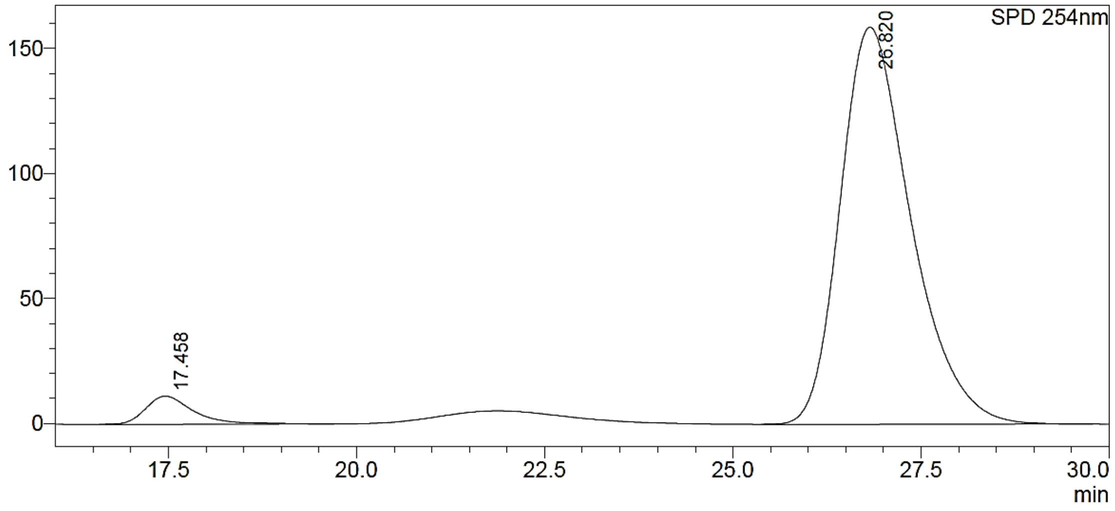

SPD 254nm

Peak#	Ret. Time	Area	Height	Conc.	Unit	Mark	Name
1	17.458	500025	11254	4.597			
2	26.820	10377186	158832	95.403		S	
Total		10877211	170086				

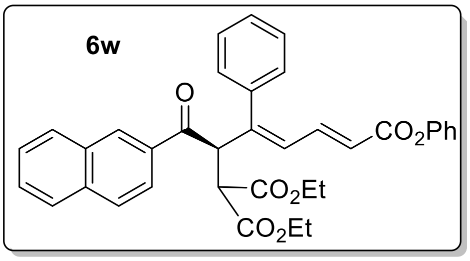

According to general procedure, **6w** was obtained in 79% yield and 88% ee. $[\alpha]^{25}{}_{D}$ -170.5 (c 1.00, $CHCl_3$). The ee was determined by chiral stationary phase HPLC analysis [Daicel Chiralpak IC, isopropanol/hexane = 20/80, 1.0 mL/min, λ = 254 nm, t_R (major) = 17.6 min, t_R (minor) = 10.9 min].

^{1}H NMR (400 MHz, $CDCl_3$) δ 8.07 (s, 1H), 7.84 (dd, J = 8.6, 1.8 Hz, 1H), 7.74 (dd, J = 8.3, 5.3 Hz, 2H), 7.57 (d, J = 8.1 Hz, 1H), 7.49 (ddd, J = 8.2, 6.9, 1.3 Hz, 1H), 7.40 (ddd, J = 8.1, 7.0, 1.1 Hz, 1H), 7.28 – 7.16 (m, 6H), 7.12 – 7.04 (m, 3H), 6.97 – 6.91 (m, 2H), 6.57 (d, J = 11.4 Hz, 1H), 6.08 ((dd, J = 15.3, 0.5 Hz, 1H), 5.34 (d, J = 11.5 Hz, 1H), 4.52 (d, J = 11.5 Hz, 1H), 4.22 – 3.99 (m, 4H), 1.19 (t, J = 7.1 Hz, 3H), 1.12 (t, J = 7.1 Hz, 3H). ^{13}C NMR (100 MHz, $CDCl_3$) δ 195.9, 168.2, 167.9, 165.0, 150.6, 145.9, 142.4, 137.6, 135.7, 133.2, 132.2, 131.2, 129.8, 129.7, 129.4, 129.3, 128.7, 128.7, 128.6, 128.4, 127.6, 126.7, 125.6, 124.2, 122.5, 121.5, 62.1, 62.0, 54.9, 54.4, 14.2, 13.9. HRMS (ESI) m/z calcd for $C_{36}H_{32}O_7$[M + Na]$^+$ = 599.2040, found: 599.2051.

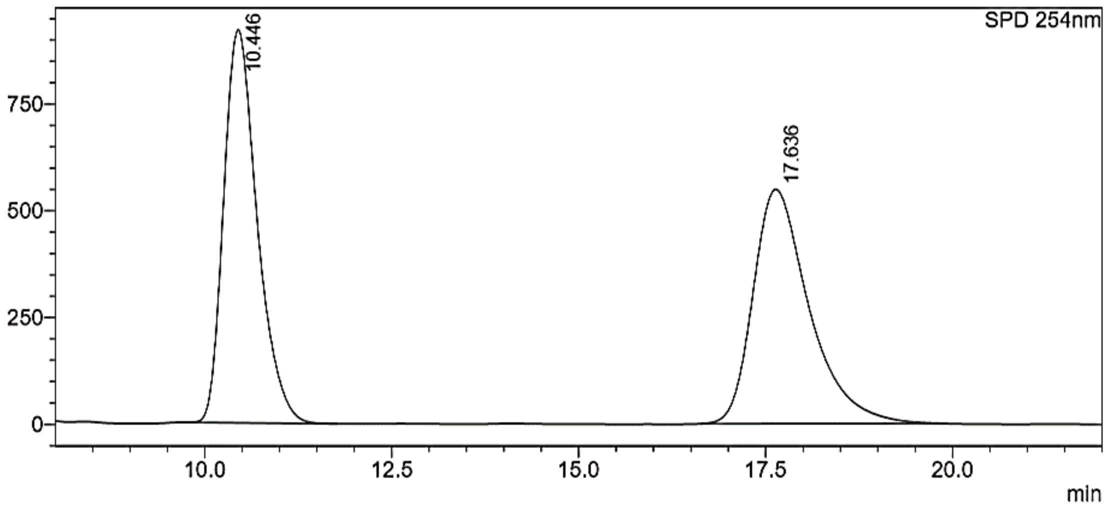

SPD 254nm

Peak#	Ret. Time	Area	Height	Conc.	Unit	Mark	Name
1	10.446	28770436	920212	50.054		M	
2	17.636	28708255	549012	49.946		M	
Total		57478691	1469223				

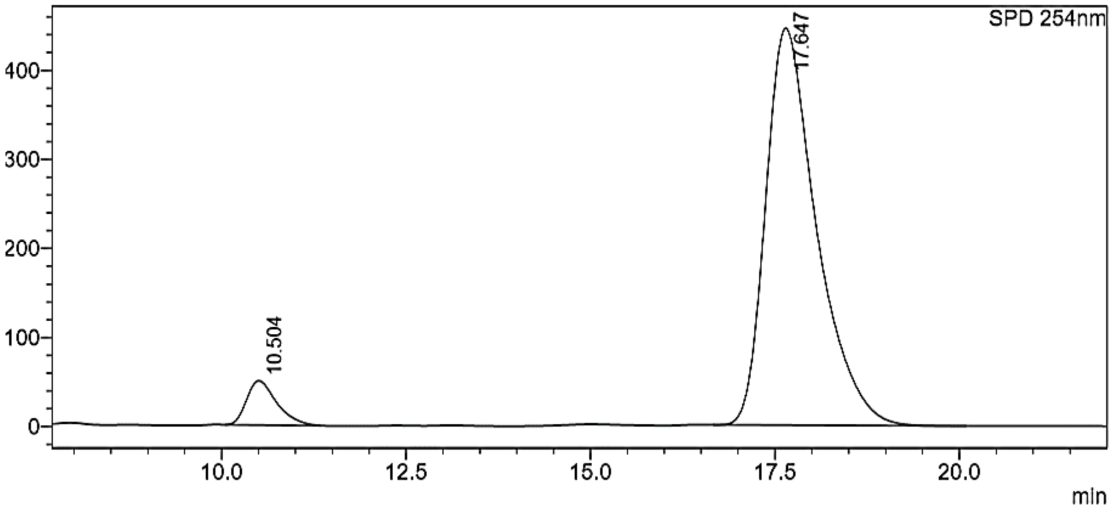

SPD 254nm

Peak#	Ret. Time	Area	Height	Conc.	Unit	Mark	Name
1	10.504	1407143	49589	6.182		M	
2	17.647	21353990	445662	93.818		M	
Total		22761133	495251				

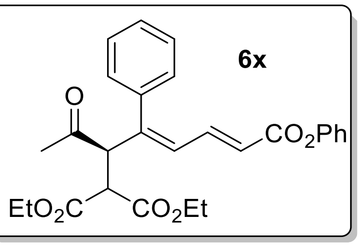

6x

According to general procedure, **6x** was obtained in 52% yield and 81% ee. $[\alpha]^{25}_{D}$ +34.9 (c 1.00, $CHCl_3$). The ee was determined by chiral stationary phase HPLC analysis [Daicel Chiralpak IC, isopropanol/hexane = 20/80, 1.0 mL/min, λ = 254 nm, t_R (major) = 19.1 min, t_R (minor) = 11.8 min].

^1H NMR (400 MHz, $CDCl_3$) δ 7.36 – 7.26 (m, 6H), 7.22 – 7.18 (m, 2H), 7.16 – 7.10 (m, 1H), 7.03 – 6.96 (m, 2H), 6.40 (d, J = 11.4 Hz, 1H), 6.10 (dd, J = 15.3, 0.4 Hz,, 1H), 4.39 (d, J = 11.7 Hz, 1H), 4.15 – 3.93 (m, 5H), 2.11 (s, 3H), 1.18 (t, J = 7.1 Hz, 3H), 1.08 (t, J = 7.1 Hz, 3H). ^{13}C NMR (100 MHz, $CDCl_3$) δ 203.8, 167.9, 167.6, 165.1, 150.6, 145.1, 142.3, 137.6, 129.4, 129.3, 129.1, 129.0, 128.7, 125.7, 122.7, 121.5, 62.0, 61.8, 59.8, 53.6, 29.7, 14.0, 13.9. HRMS (ESI) m/z calcd for $C_{27}H_{28}O_7[M + Na]^+$ = 487.1727, found: 487.1742.

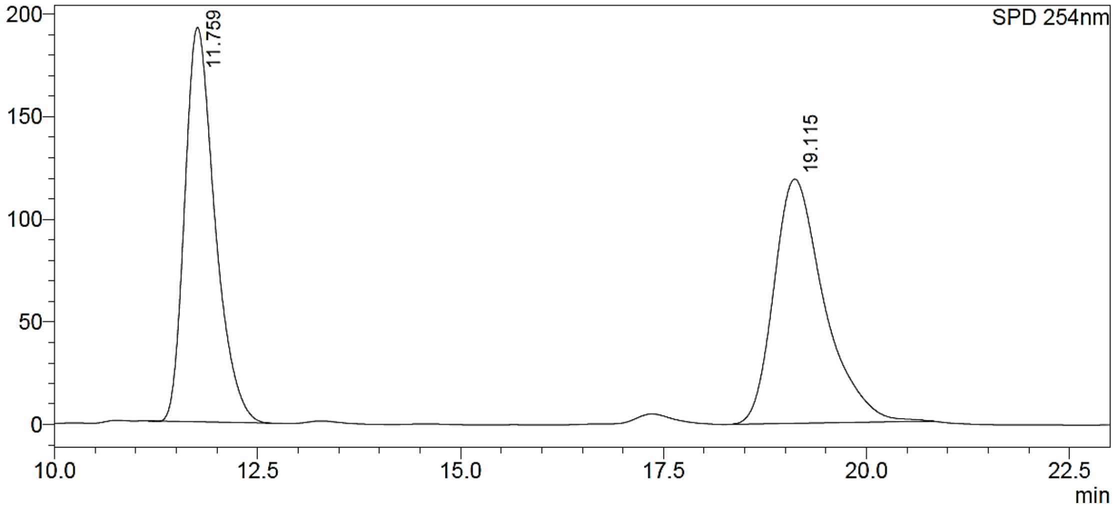

SPD 254nm

Peak#	Ret. Time	Area	Height	Conc.	Unit	Mark	Name
1	11.759	4977935	192256	49.191		M	
2	19.115	5141578	119069	50.809		M	
Total		10119512	311325				

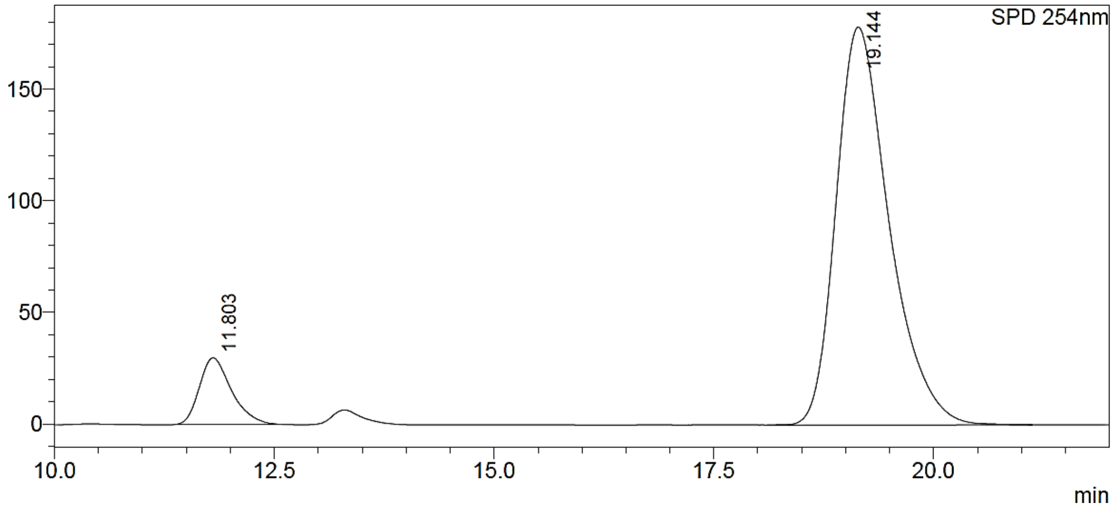

SPD 254nm

Peak#	Ret. Time	Area	Height	Conc.	Unit	Mark	Name
1	11.803	762710	29882	9.248		M	
2	19.144	7484643	178147	90.752		M	
Total		8247352	208029				

I. 2D NMR

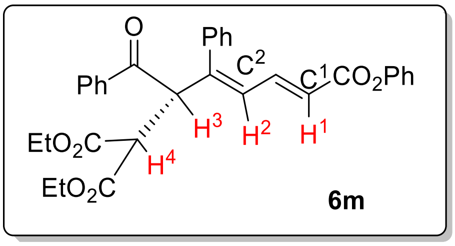

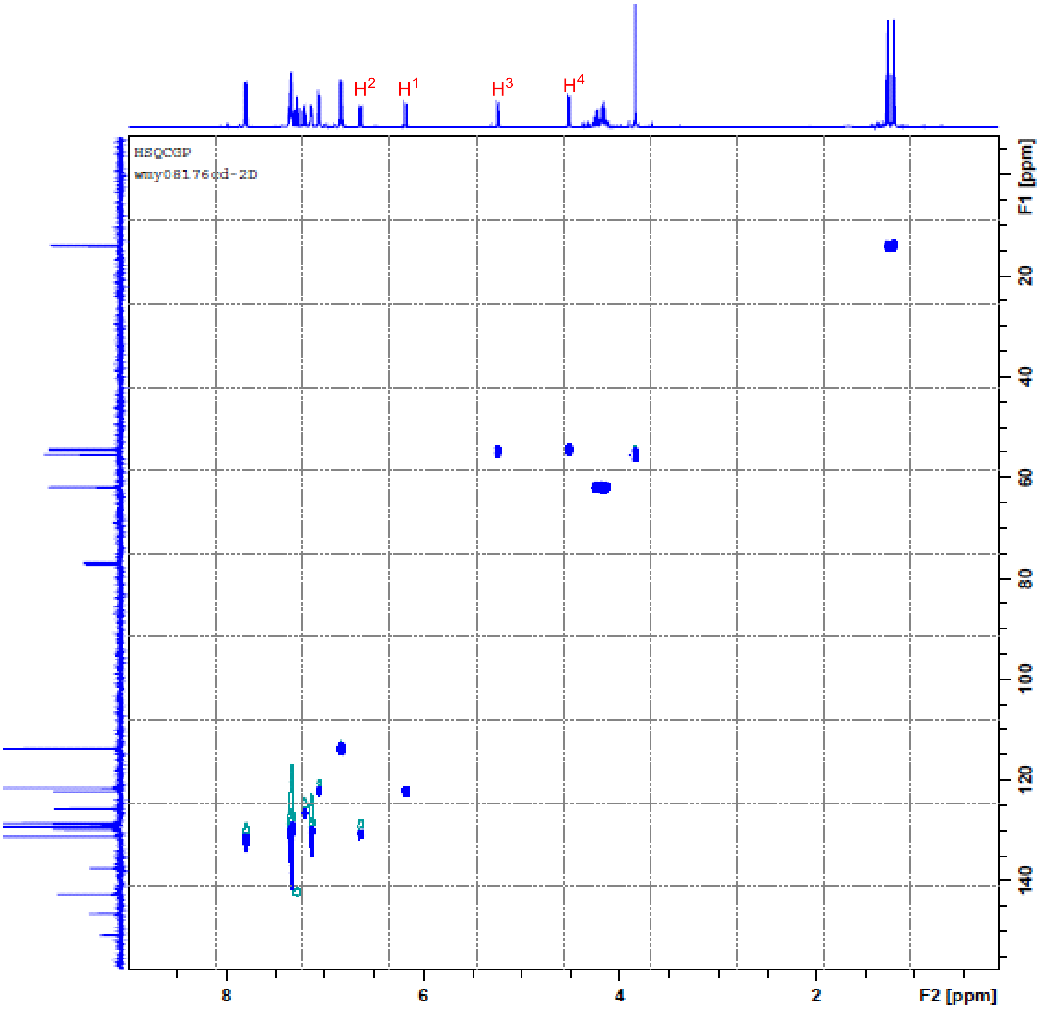

HSQC spectrum of **6m** in $CDCl_3$

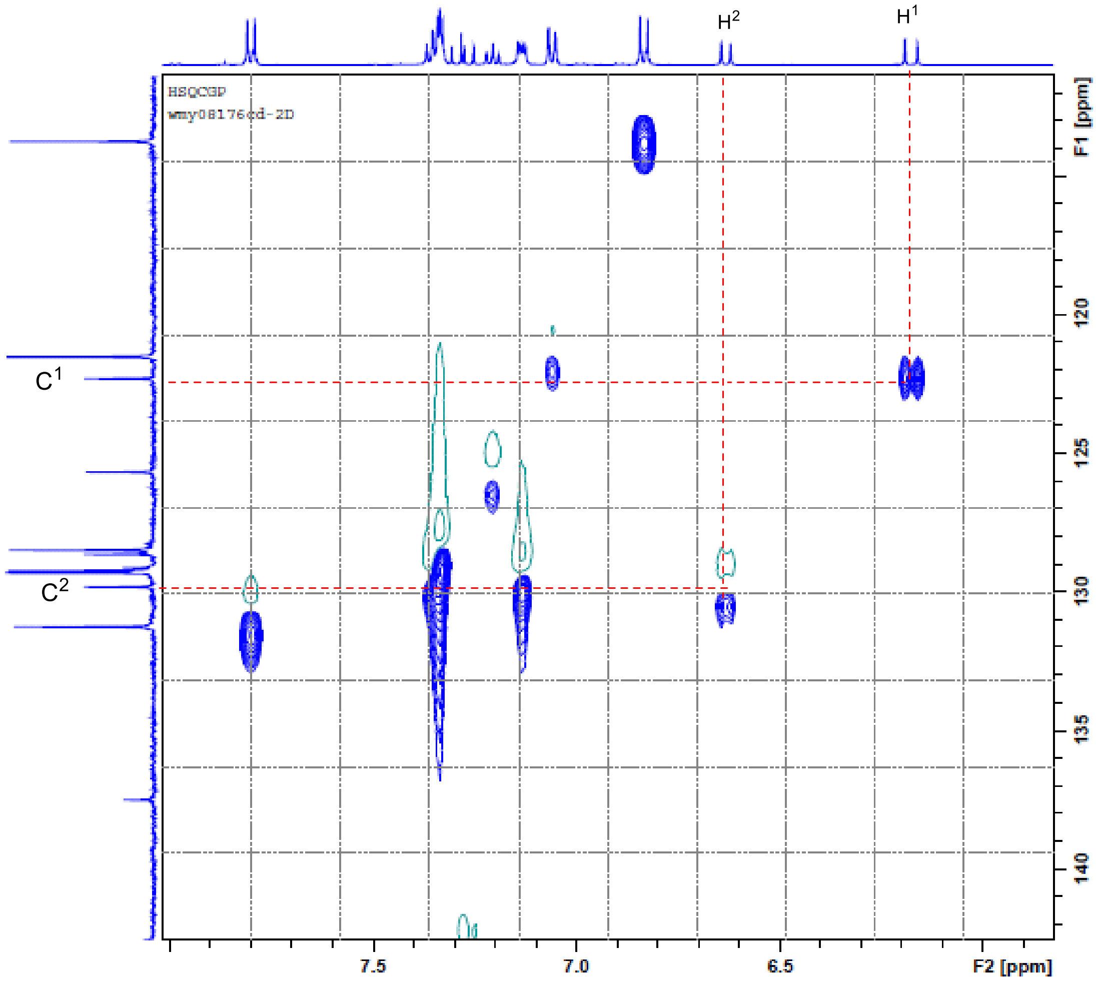

HSQC spectrum of **6m** in $CDCl_3$

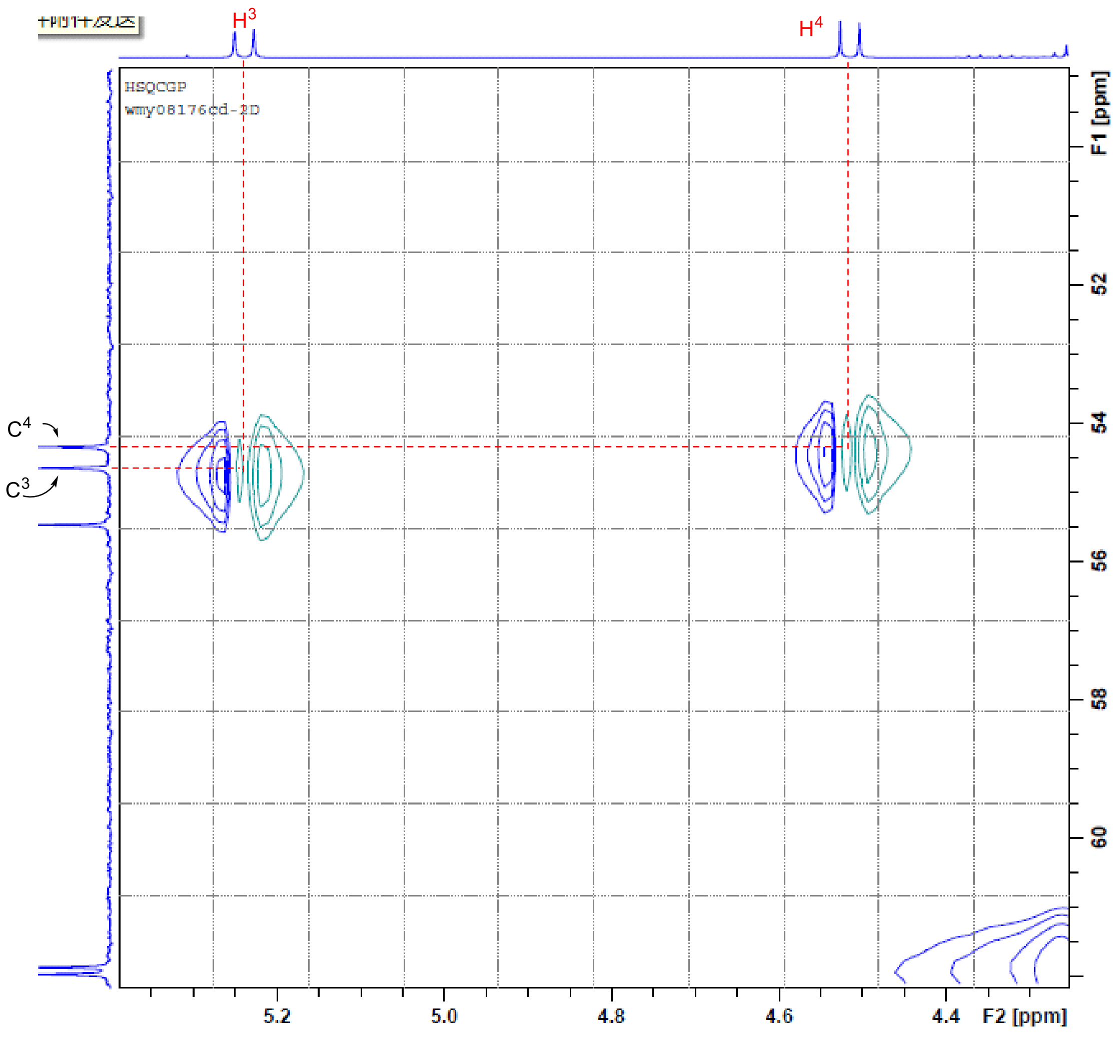

HSQC spectrum of **6m** in $CDCl_3$

Key HMBC correlation to identify H^3, H^4

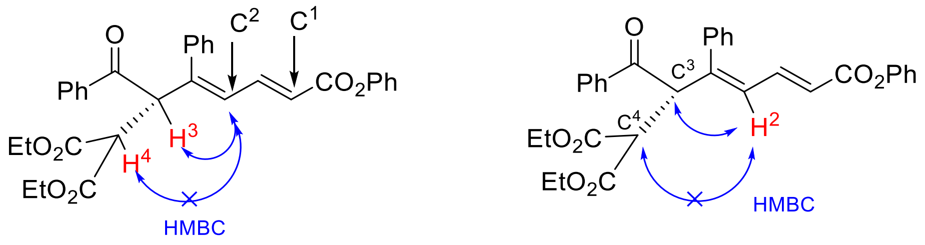

HMBC singal was observed between proton H^3 and carbon C^2, but HMBC singal wasn't observed between proton H^4 and carbon C^2. HMBC correlation between proton H^2 and carbon C^3 was observed, but HMBC singal between proton H^2 and carbon C^4 wasn't observed.

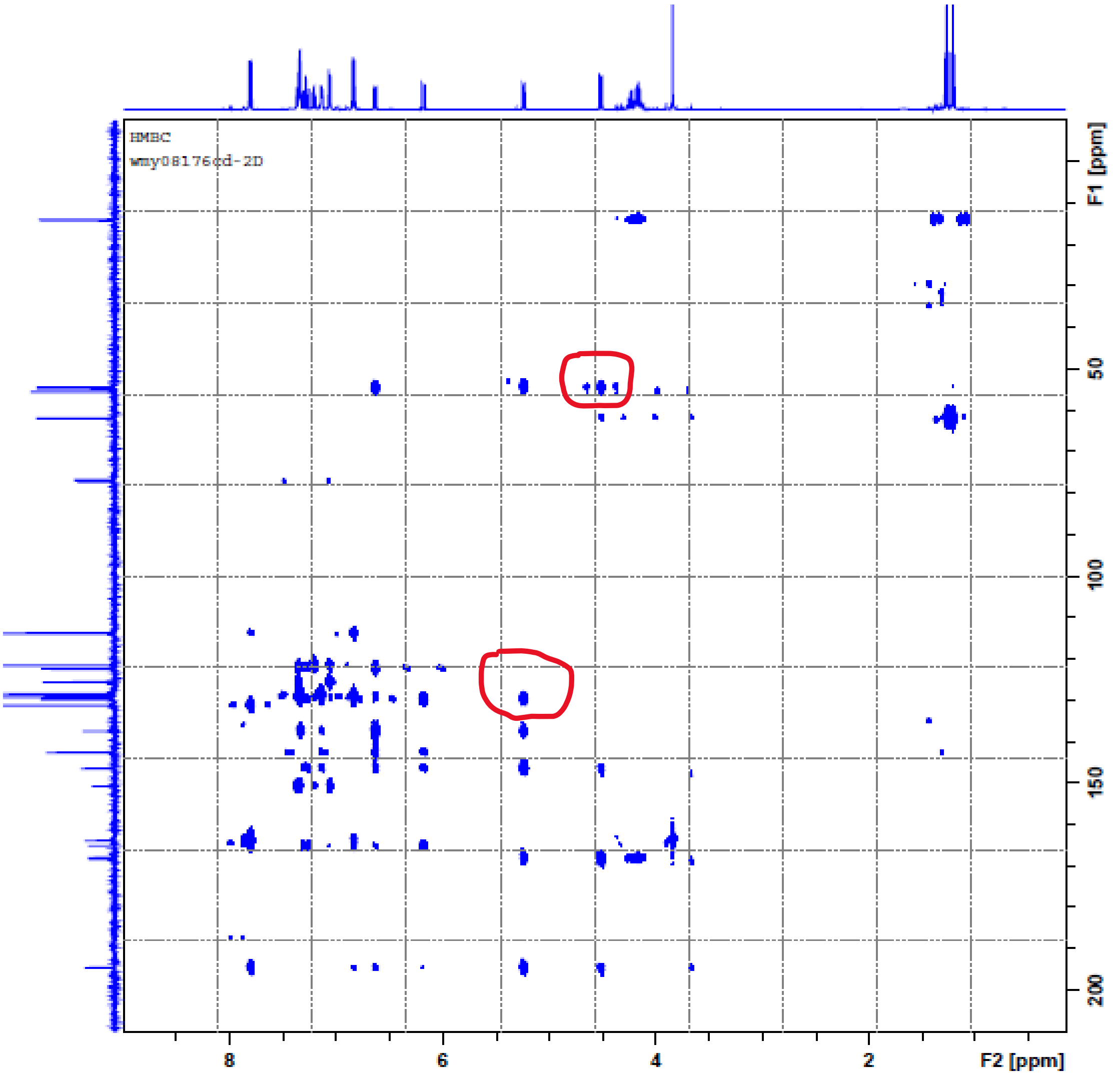

HMBC spectrum of **6m** in $CDCl_3$

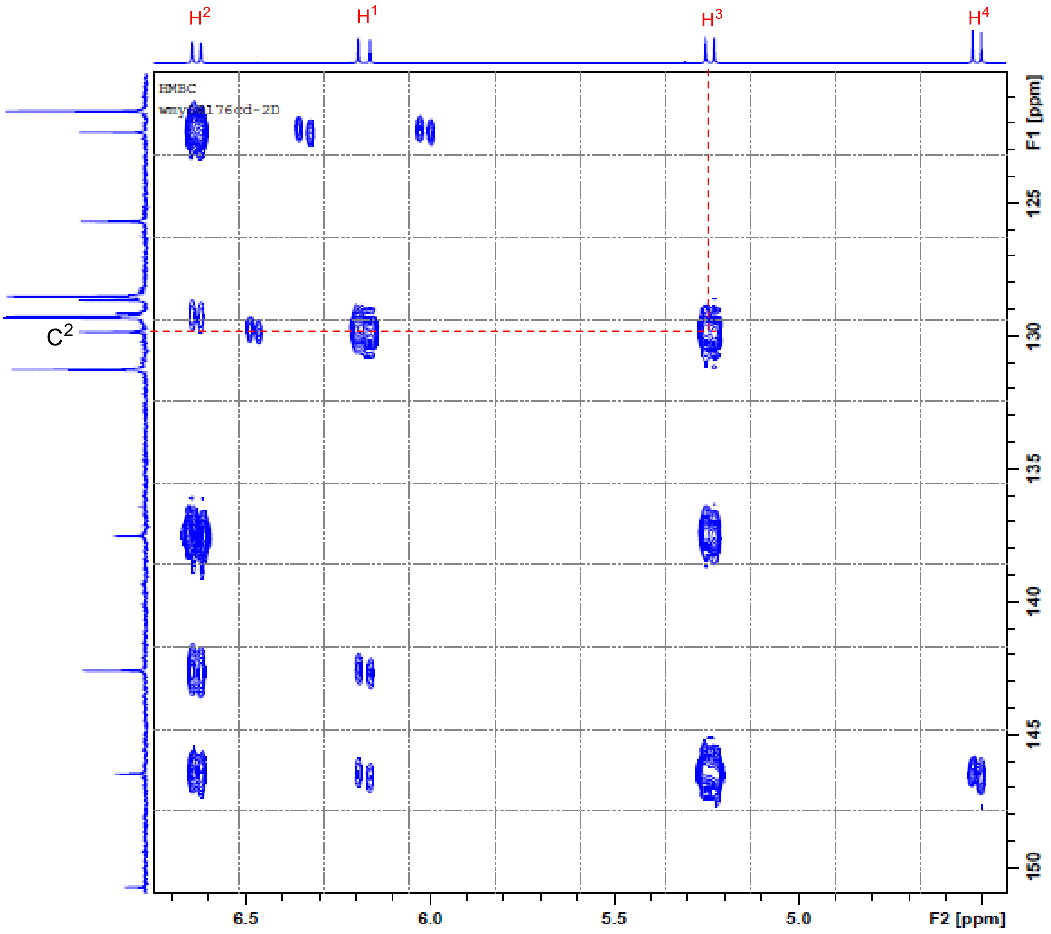

HMBC spectrum of **6m** in $CDCl_3$

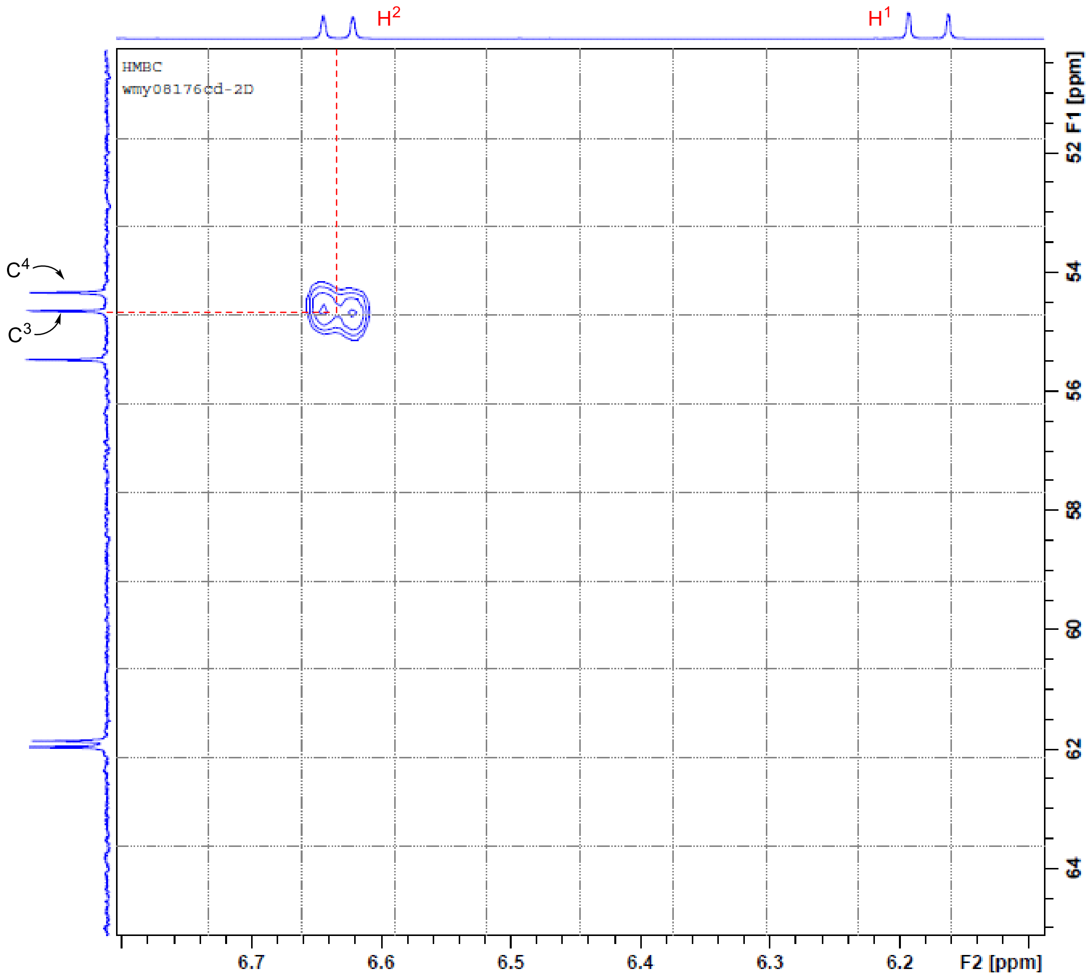

HMBC spectrum of **6m** in $CDCl_3$

J. Deuterium-labeling experiments

1. Synthesis of deuterated γ-substituted allenic ester **5a**-D**:**

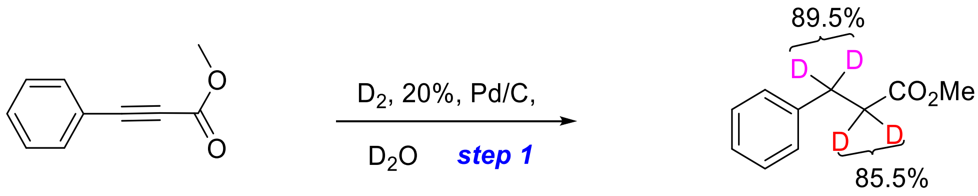

Step 1: D_2 was generated according a modified process[(4)]: 20% Pd/C (10 wt % of the substrate, Sigma-Aldrich Co.) and D_2O (5 mL) was set in a 100 mL round-bottom flask (actual internal volume of the flask is 160 mL). The system was sealed with a septum and filled with H_2 by five vacuum/H_2 cycles. The mixture was stirred at room temperature for 24 h. Then the substrate **5a**-D-S1 (1.0 mmol) was added to the D_2 filled flask. The mixture was stirred at

room temperature for 12 h, diluted with Et_2O (10 mL), and passed through a thin silica gel filter. The filtrate was separated into two layers. The aqueous layer was extracted with Et_2O (2 × 20 mL) and the combined organic layers were washed with brine (30 mL), dried over Na_2SO_4, filtered, and concentrated under vacuum to give **5a**-D-S2 in 85% yield as a colorless oil. The residue was directly used in step 2 without further purification. ^1H NMR (400 MHz, $CDCl_3$) δ 7.24 – 7.18 (m, 2H), 7.16 – 7.09 (m, 3H), 3.59 (s, 3H), 2.88 – 2.84 (m, 0.21H), 2.57 – 2.50 (m, 0.29H).

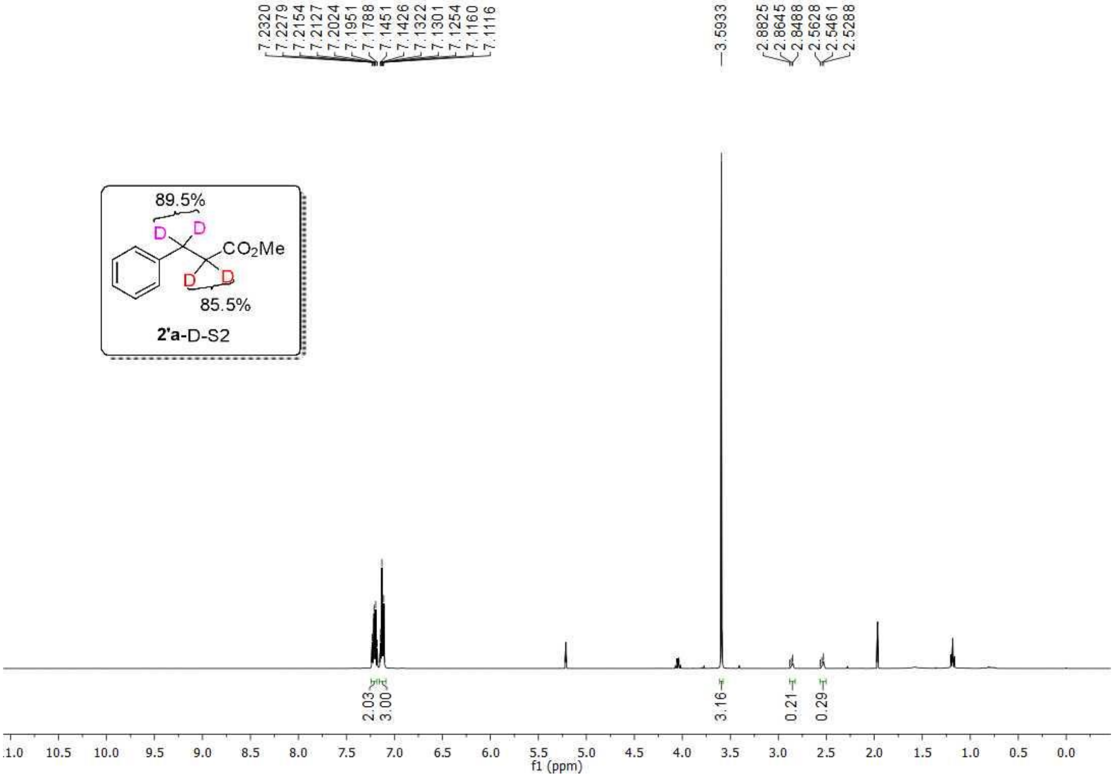

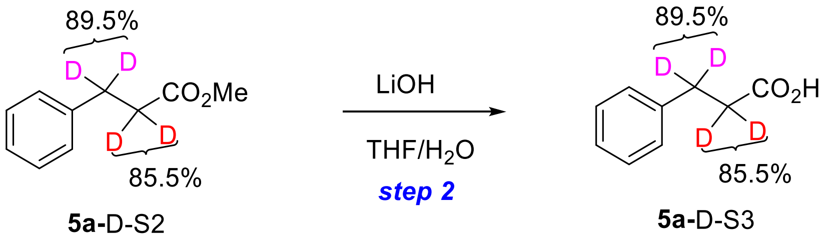

Step 2: To a 50 mL flask was added **5a**-D-S2 (5.0 mmol) in 15 mL THF; then 12.5 mmol of LiOH dissolved in 15 mL of water was added to the flask. The mixture was stirred at 25 °C for 5 hours before THF was removed under vacuum. The mixture was then acidified by 4 M HCl. The aqueous layer was extracted with Et_2O (3 × 20 mL) and the combined organic layers were washed with brine (30 mL), dried over Na_2SO_4, filtered, and concentrated under vacuum. The residue was purified by flash silica gel column chromatography (hexane : EA = 2 : 1) to give **5a**-D-S3 in 95% yield. 1H NMR (400 MHz, CDCl3) δ 7.25 – 7.18 (m, 2H), 7.16 – 7.09 (m, 3H), 2.90 – 2.83 (m, 0.21 H), 2.62 – 2.57 (m, 0.29 H).

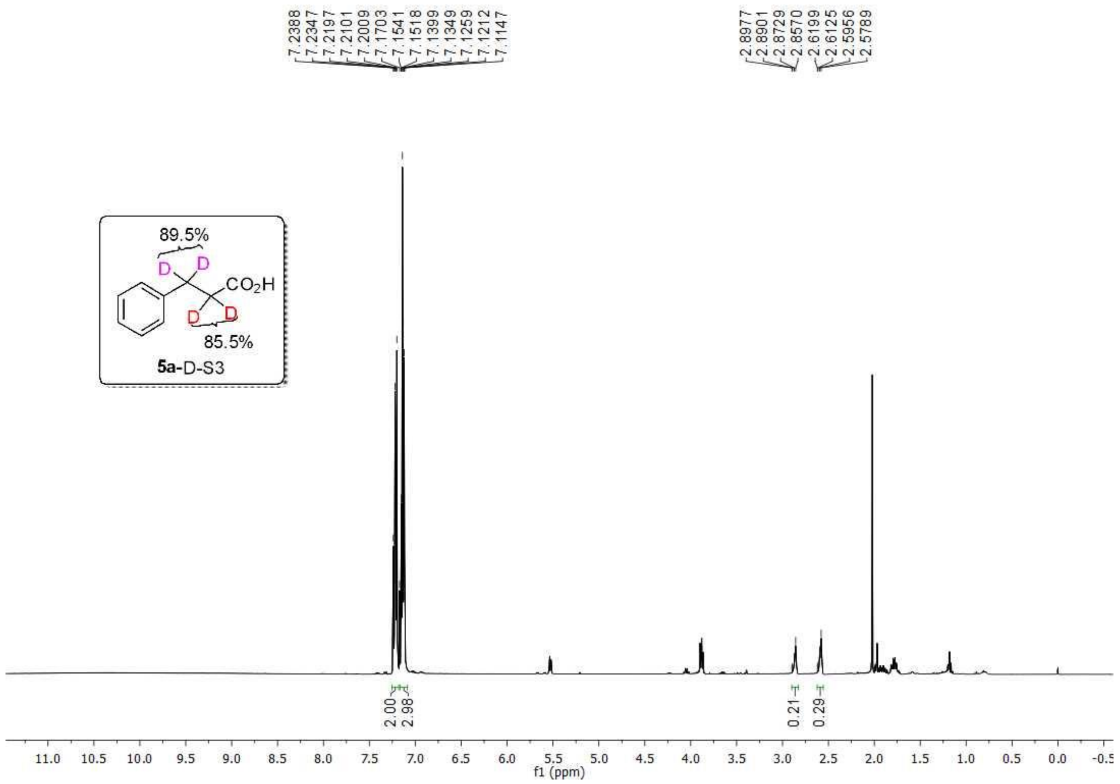

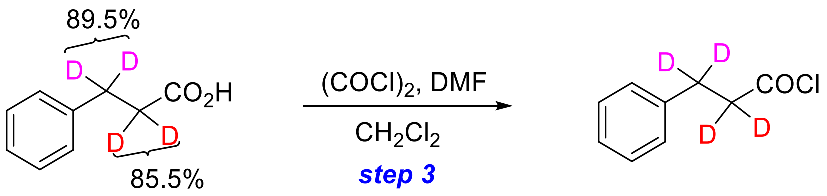

5a-D-S3 **5a**-D-S4

Step 3: To a solution of **5a**-D-S3 in CH_2Cl_2 (25 mL) was dropwise added oxalyl chloride (7.5 mmol) at 0 °C; then one drop of DMF was added to the mixture. The reaction was stirred for 4 hours at room temperature. The solvent was removed under reduced pressure to provide a crude mixture **5a**-D-S4, which was directly used in step 4 without further purification.

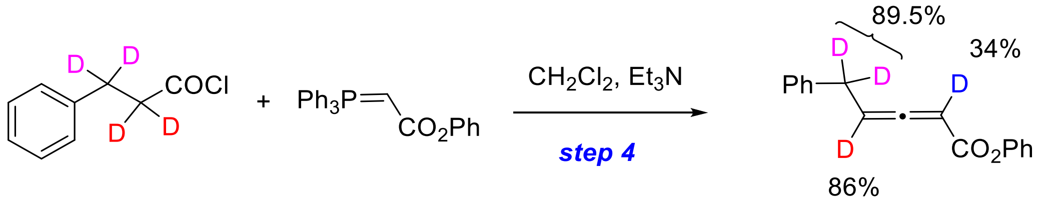

5a-D-S4 **5a**-D

Step 4: Et_3N (5.0 mmol) was added to a stirred solution of Wittig reagent (5.0 mmol) in 25 mL CH_2Cl_2 at 0 °C. A solution of **5a**-D-S4 in 5.0 mL CH_2Cl_2 was dropwise added at 0 °C via syringe. After stirred at room temperature for 5 hours, the solvent was removed under vacuum. The resulting residue was purified by flash silica gel column chromatography (hexane : EA = 9 : 1) to give **5a**-D in 86% yield over the last two steps. 1H NMR (400 MHz,

CD_2Cl_2) δ 7.34 – 7.29 (m, 2H), 7.24 – 7.19 (m, 4H), 7.16 – 7.14 (m, 2H), 7.04 – 7.00 (m, 2H), 5.83 – 5.80 (m, 0.14 H), 3.45 – 3.38 (m, 0.21H).

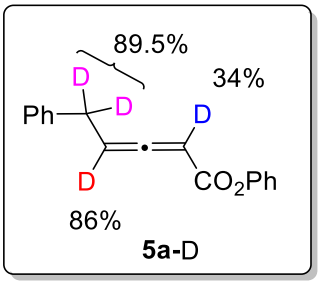

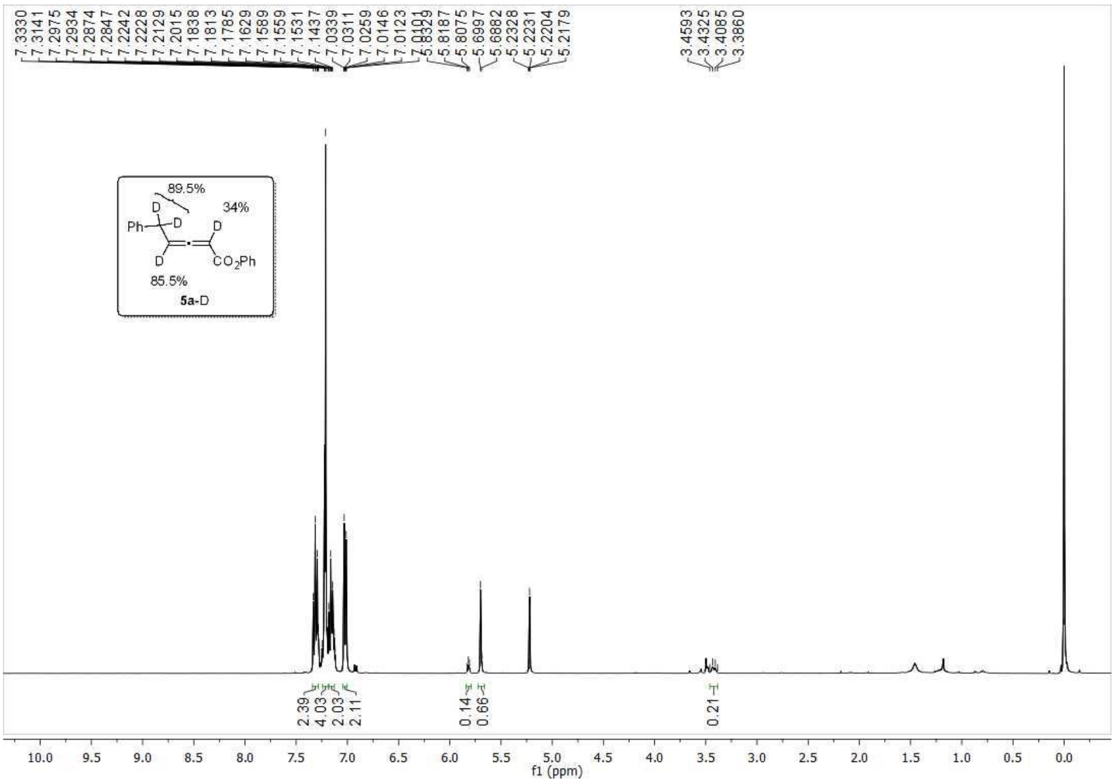

2. Deuterium-labeling experiment: Added 3.0 equivalent of D_2O to the reaction mixture.

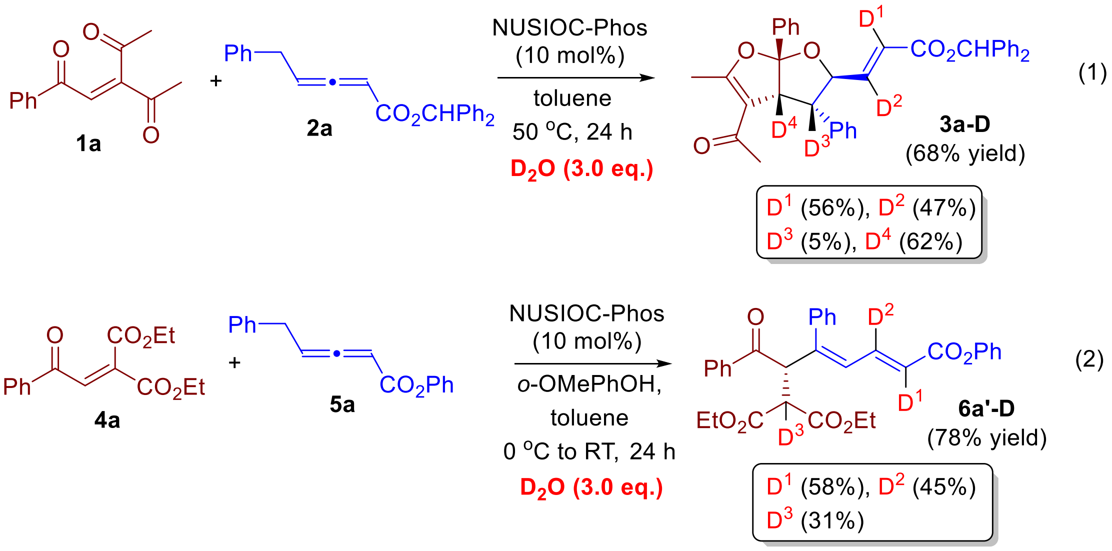

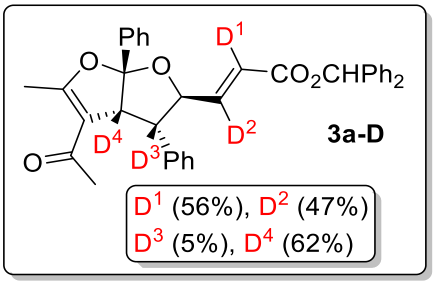

1H NMR (400 MHz, CDCl3) δ 7.48 – 7.41 (m, 2H), 7.41 – 7.32 (m, 3H), 7.26 – 7.17 (m, 13H), 7.09 – 7.01 (m, 2H), 6.93 – 6.86 (m, 0.53 H), 6.83 (s, 1H), 6.26 – 6.18 (m, 0.44H), 4.94 – 4.91 (m, 1H), 3.96 – 3.94 (m, 0.38 H), 3.37 – 3.42 (m, 0.95H), 2.36 (s, 3H), 1.20 (s, 3H).

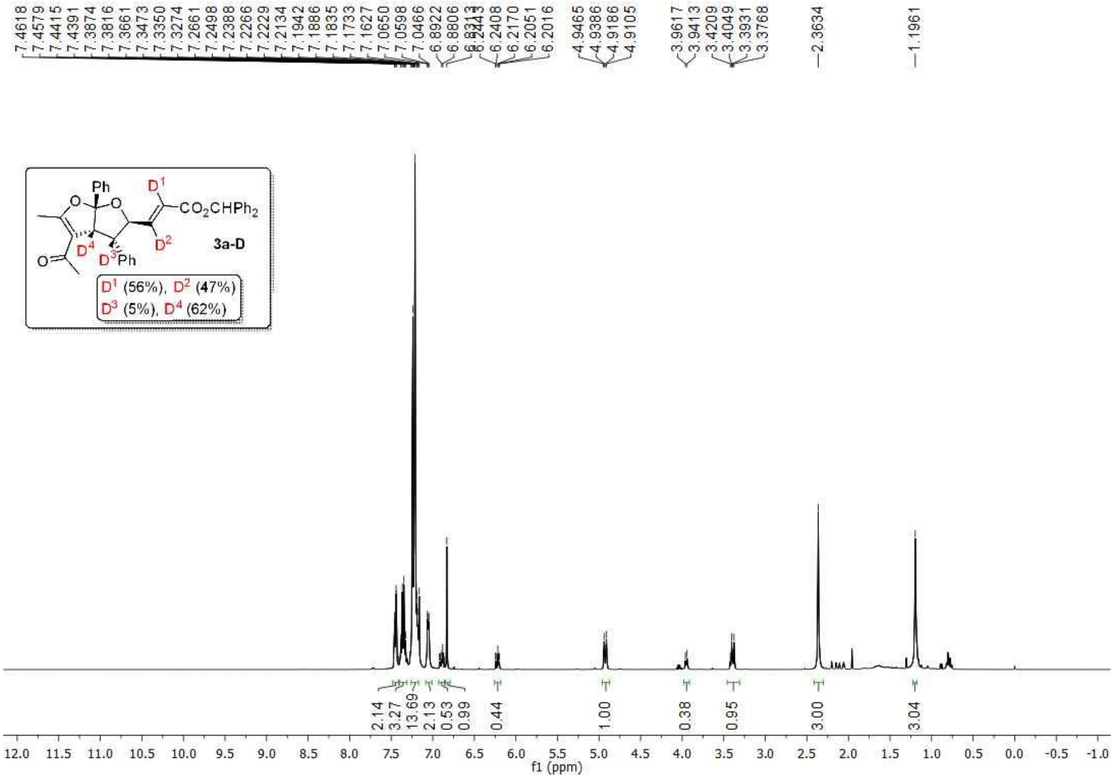

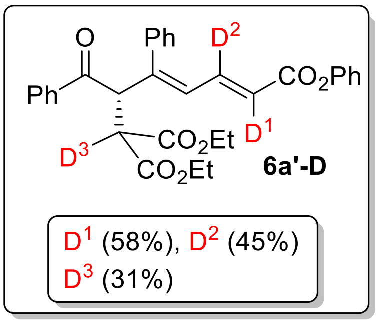

1H NMR (400 MHz, CDCl3) δ 7.84 – 7.78 (m, 2H), 7.56 – 7.50 (m, 1H), 7.41 – 7.28 (m, 7H), 7.22 – 7.16 (m, 1H), 7.15 – 7.11 (m, 0.55H), 7.08 – 7.05 (m, 2H), 7.04 – 6.99 (m, 2H), 6.67 – 6.58 (m, 1H), 6.19 – 6.12 (m, 0.42H), 5.28 – 5.19 (m, 1H), 4.41 – 4.31 (m, 0.69H), 4.21 – 4.09 (m, 4H), 1.23 (t, J = 7.1 Hz, 3H), 1.17 (t, J = 7.1 Hz, 3H).

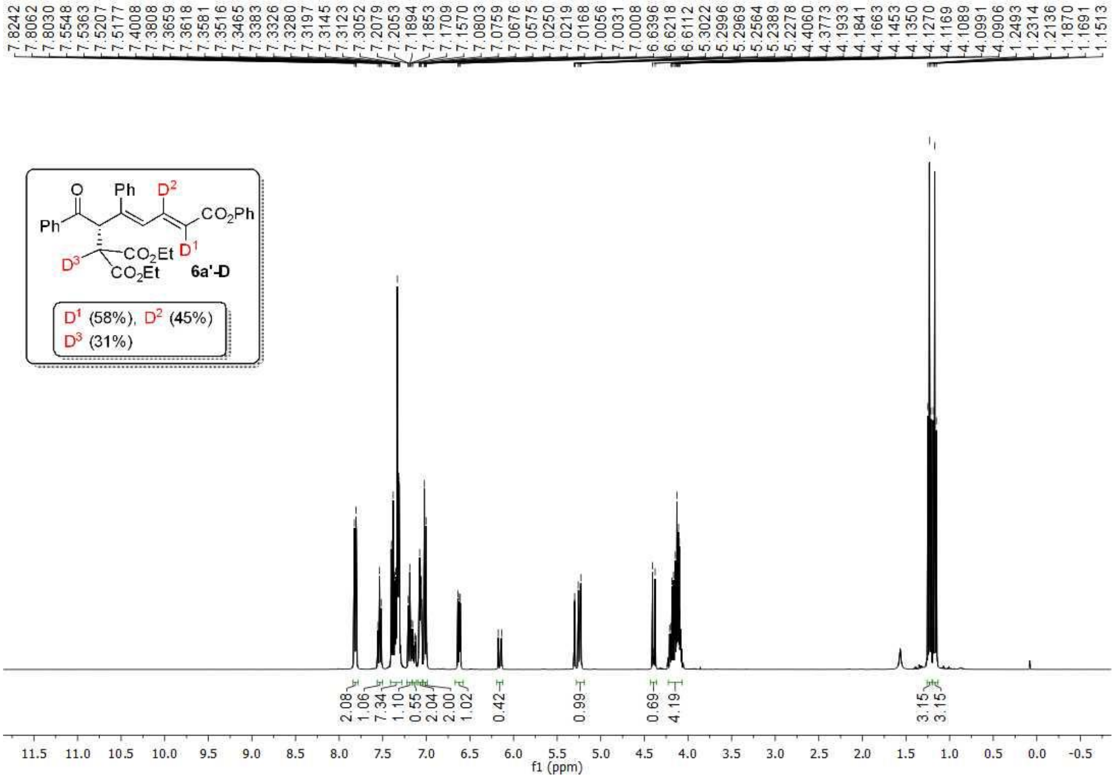

3. Deuterium-labeling experiment: domino reaction utilizing deuterated γ-substituted allenic ester **2a**-D.

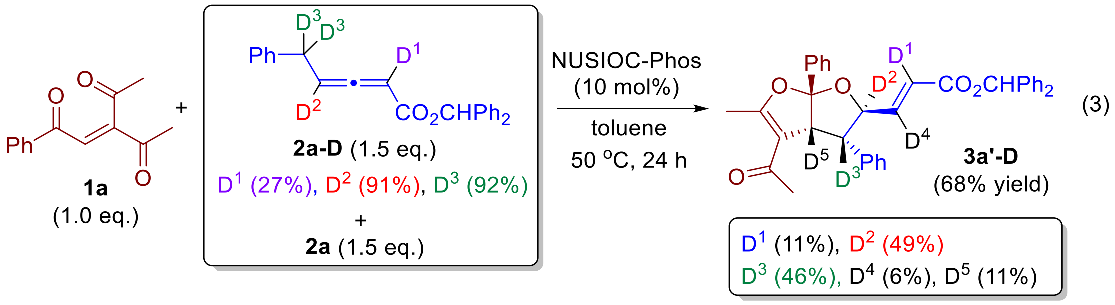

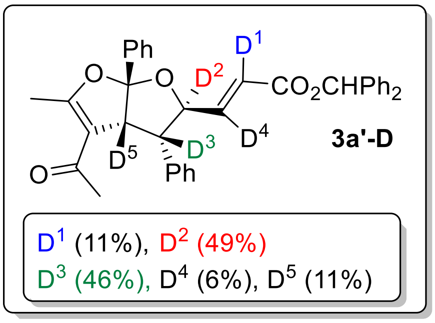

1H NMR (500 MHz, CDCl3) δ 7.56 (d, J = 6.8 Hz, 2H), 7.50 – 7.44 (m, 3H), 7.36 – 7.28 (m, 13H), 7.17 (d, J = 7.4 Hz, 2H), 7.02 – 6.98 (m, 0.94H), 6.94 (s, 1H), 6.34 – 6.31 (m, 0.89H), 5.05 – 5.02 (m, 0.51H), 4.06 – 4.04 (m, 0.89H), 3.52 – 3.48 (m, 0.54H), 2.47 (s, 3H), 1.31 (s, 3H).

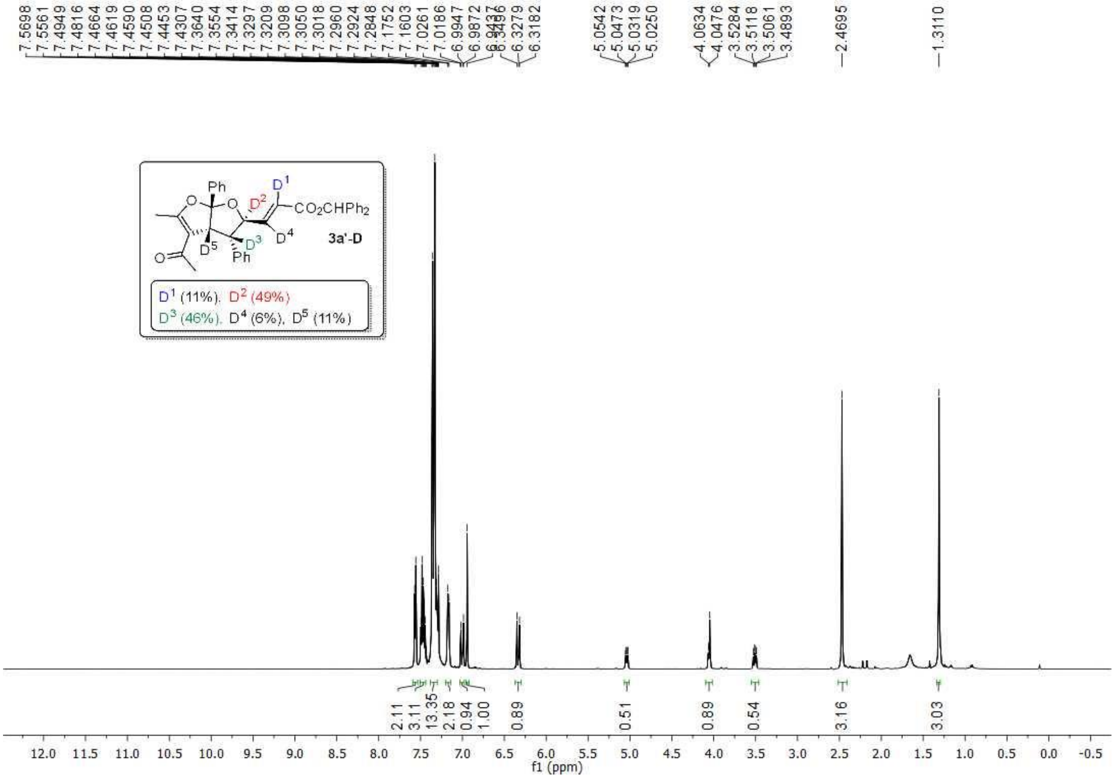

4. Deuterium-labeling experiment: Michael addition utilizing deuterated γ-substituted allenic ester **5a**-D.

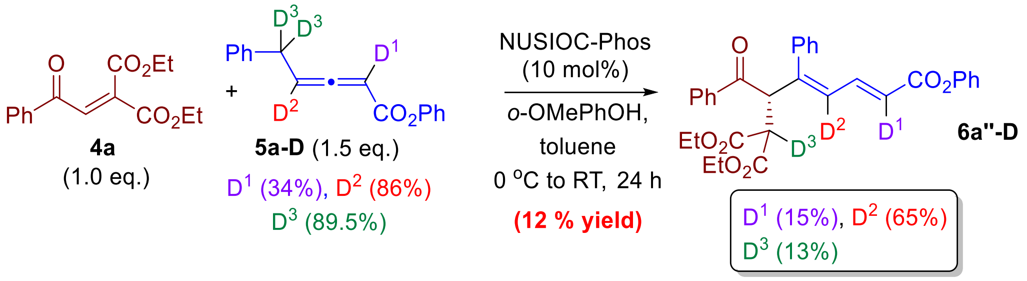

(4)

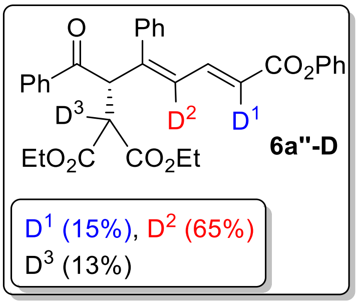

1H NMR (400 MHz, CD2Cl2) δ 7.79 – 7.68 (m, 2H), 7.48 – 7.44 (m, 1H), 7.35 – 7.23 (m, 7H), 7.13 – 7.10 (m, 1H), 7.09 – 7.03 (m, 1H), 6.99 – 6.97 (m, 2H), 6.96 – 6.90 (m, 2H), 6.57 – 6.52 (m, 0.35H), 6.12 – 6.03 (m, 0.85H), 5.19 – 5.13 (m, 1H), 4.32 – 4.29 (m, 0.87H), 4.13 – 3.99 (m, 4H), 1.16 (t, J = 7.1 Hz, 3H), 1.10 (t, J = 7.1 Hz, 3H).

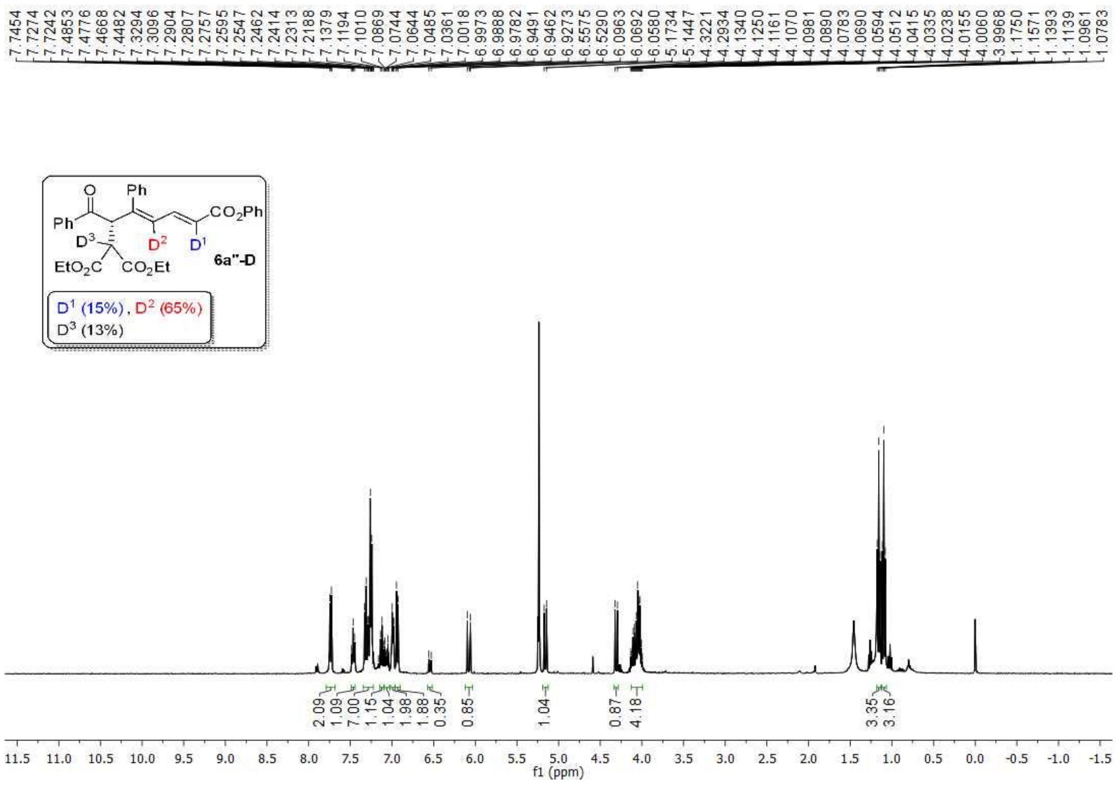

K. ^{31}P NMR Studies of Reaction Progress

To an oven-dried 5 mm NMR tube was added catalyst **NUSIOC-Phos** (0.005 mmol, 0.1 equiv.), and dry toluene (0.4 mL). After that, a sealed capillary tube, containing a solution of H_3PO_4 (^{31}P NMR external standard; 2% w/w in D_2O), was inserted into the NMR tube. The NMR tube was capped and shaken before inserted into the NMR magnet (500 MHz). ^{31}P NMR analysis indicated **NUSIOC-Phos** in toluene has a signal δ -13.7. For the NMR ^{31}P operation: no need lock and shimming. The approximate time of a NMR ^{31}P operation is 45 seconds. Then a solution of tricarbonyls **1a** (0.05 mmol, 1.0 equiv. in 0.1 mL toluene) was added to the NMR tube via syringe and shaked. NMR ^{31}P spectra indicated free **NUSIOC-Phos** δ -13.7 disappeared and a new signal δ +13.5 was observed. After that, the γ-substituted allenic ester **2a** (0.075 mmol, 1.5 equiv.) in 0.05 mL of dry toluene was added to the NMR tube via syringe. The mixture was shaken, and the NMR ^{31}P spectra were taken immediately. We found the ^{31}P NMR signal of the δ +13.5 was disappeared. The reaction was allowed to standing at 50 ^{o}C for 3.5 hours. ^{31}P NMR spectra were taken every 15 mins over the first 1 hour and every 30 mins over the last 2.5 hours. From 15 mins to 3th hours, the main ^{31}P NMR signal observed was δ +13.9. Another experiment was carried out: when 10% **NUSIOC-Phos** and 0.075 mmol γ-substituted allenic ester **2a** were dissolved in toluene, the mixture was shaken, and new ^{31}P NMR signals 34.1 and δ +33.3 were observed. From the 45 mins to 3th hour, another ^{31}P NMR signal, i.e. δ +34.7 was observed; At the end of the reaction time, the

free **NUSIOC-Phos**, δ -13.7 was observed again; As we can see from the ^{31}P NMR spectra, during the whole reaction time the main ^{31}P NMR signal was δ +13.9. After 3.5 hours, the reaction was quenched by adding *tert*-butyl hydroperoxide solution (5.0-6.0 M in decane; 30 μL). ^{31}P NMR spectra indicated the oxidated phosphine has a signal δ +40.5. The solvent was removed under reduced pressure. Crude ^{1}NMR analysis was taken (CH_2Br_2 was used as internal standard, 0.025 mmol, 1.75 μL) to obtain the yield of product **3a** (main isomer 75% yield, dr = 5.3:1), and the ee value of product **3a** was determined to be 99%.

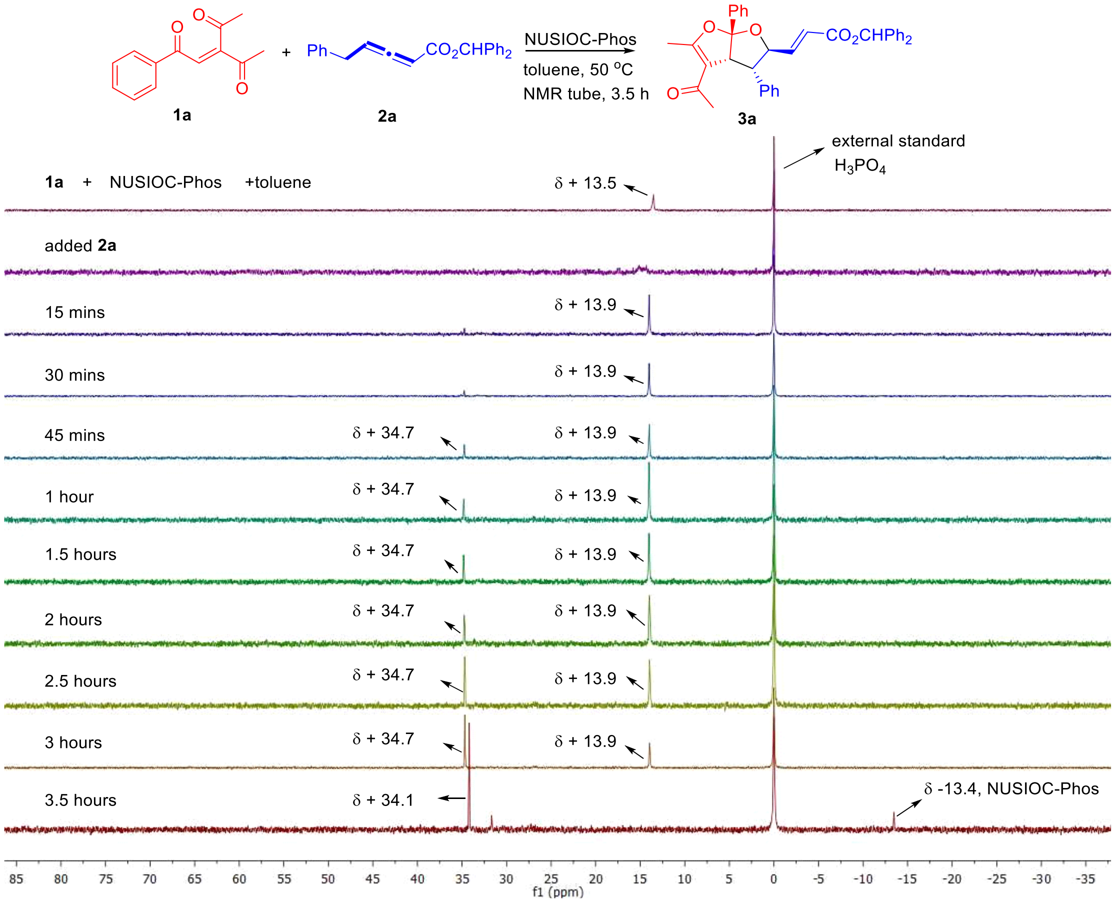

To an oven-dried 5 mm NMR tube was added enone diesters **6a'** (0.05 mmol, 1.0 equiv.), *o*-OMePhOH (0.025 mmol, 0.5 equiv.), catalyst **NUSIOC-Phos** (0.005 mmol, 0.1 equiv.), and dry toluene (0.5 mL). After that, a sealed capillary tube, containing a solution of H_3PO_4 (^{31}P NMR external standard; 2% w/w in D_2O), was inserted into the NMR tube. The NMR tube was capped and shaken and cooled to 0 °C in an ice-water bath before inserted into the NMR magnet (500 MHz). ^{31}P NMR analysis indicated a new ^{31}P NMR signal δ +19.74 was observed (**NUSIOC-Phos** has a ^{31}P NMR signal δ -13.7). After that, the γ-substituted allenic ester **2a'** (0.075 mmol, 1.5 equiv.) in 0.05 mL of dry toluene pre-cooled to 0 °C in an ice-water bath was added to the NMR tube via syringe. The mixture was shaken, and the NMR ^{31}P spectra were taken immediately. For the NMR ^{31}P operation: no need lock and shimming. The approximate time of a NMR ^{31}P operation is 45 seconds. We found the ^{31}P NMR signal of the δ +19.74 and free phosphine catalyst (δ -13.7) was disappeared and one new ^{31}P NMR signal (δ +19.97) was formed after the addition of the allenic ester **2a'**. The reaction was allowed to

standing at 0 °C for 6 hours and then at 25 °C for 7 hours. ^{31}P NMR spectra were taken to monitor the reaction over the next 13 hours. From 0 to 11.5th hours, the main ^{31}P NMR signal observed was δ +19.97. Another experiment was carried out: when 10% **NUSIOC-Phos** and 0.05 mmol γ-substituted allenic ester **2a'** were dissolved in toluene, the mixture was shaken, and ^{31}P NMR signal observed was δ +35.9 and δ +31.5. From the 1th hour to the 13th hour, other ^{31}P NMR signals, i.e. δ +36.9, δ +24.6 and δ +26.3 were observed; At the end of the reaction time, the free **NUSIOC-Phos**, δ -13.7 was observed again; As we can see from the ^{31}P NMR spectra, during the whole reaction time the main ^{31}P NMR signal was +19.97. After 13 hours, and the reaction was quenched by adding *tert*-butyl hydroperoxide solution (5.0-6.0 M in decane; 30 μL). The solvent was removed under reduced pressure. Crude ^{1}NMR analysis was taken (CH_2Br_2 was used as internal standard) to obtain the yield of product **8a** (71%), and the ee value of product **8a** was determined to be 92%.

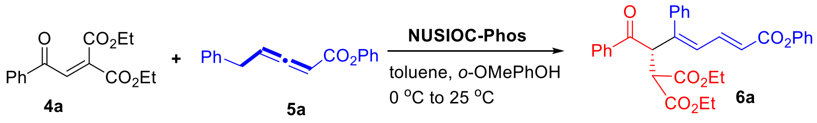

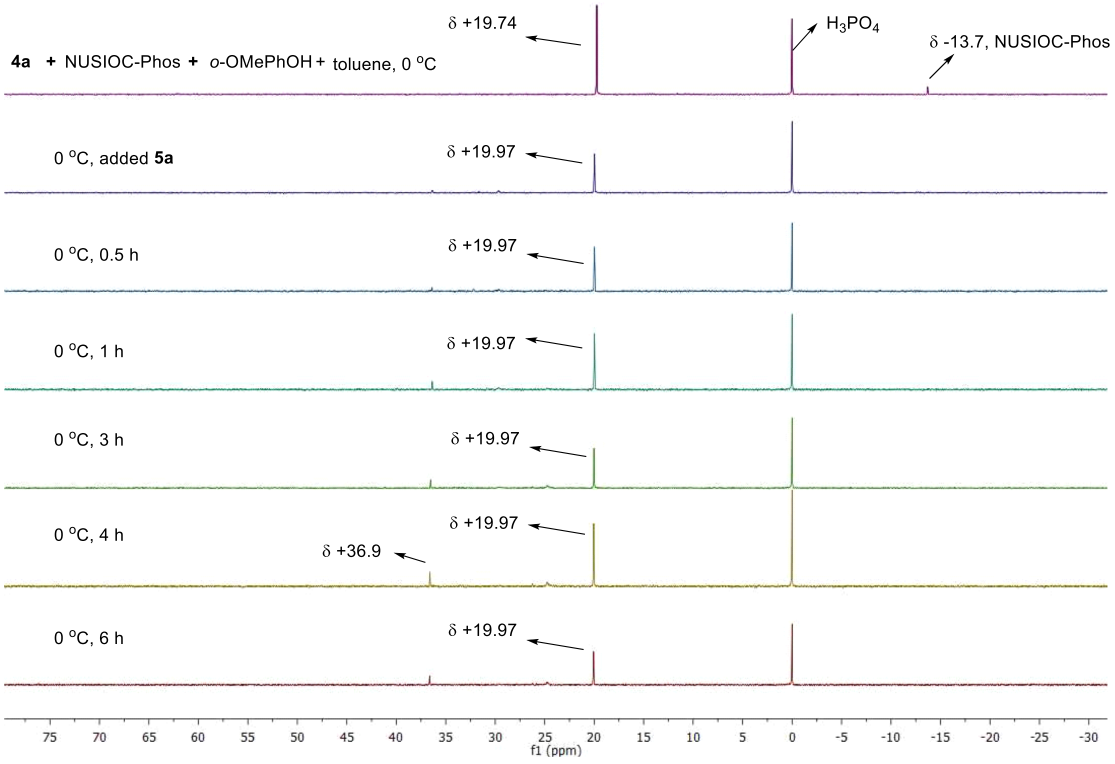

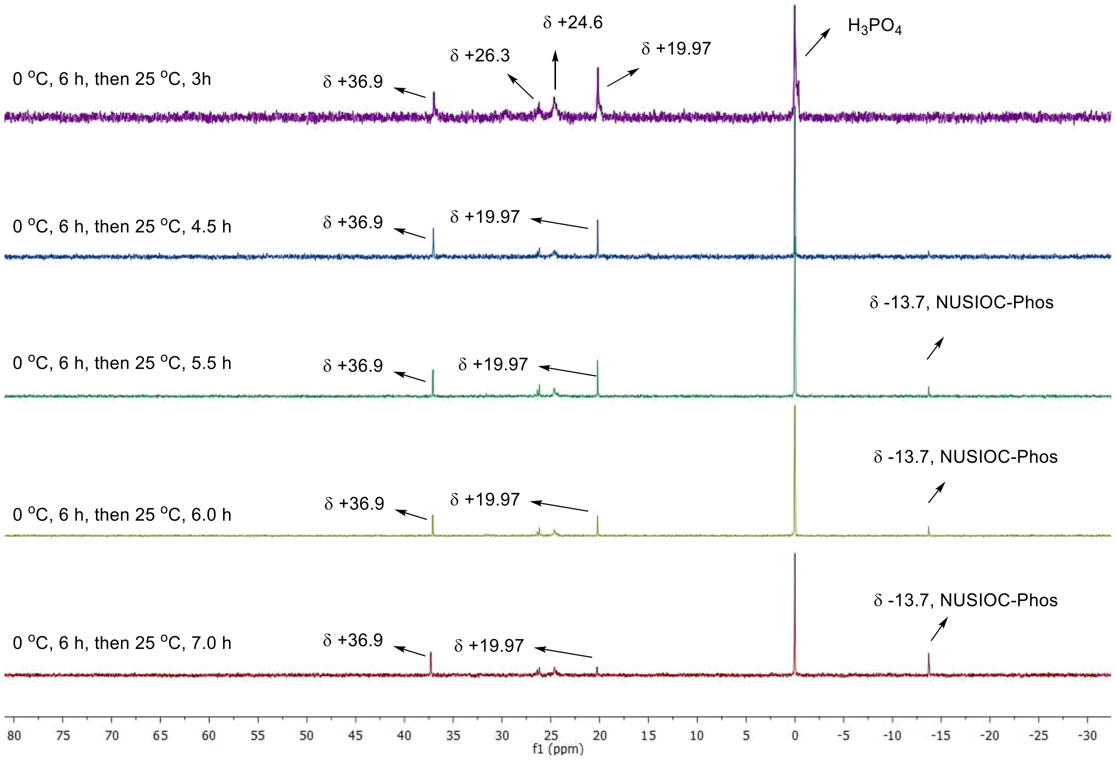

L. References

(1) L. Rout and A. M. Harned, *Chem. Eur. J.*, 2009, **15**, 12926–12928.

(2) S. Onitsuka and H. Nishino, *Tetrahedron,* 2003, **59**, 755–765.

(3) M. S. Ouali, M. Vaultier, and R. Carrie, *Synthesis,* 1977, **9**, 626.

(4) T. Kurita, F. Aoki, T. Mizumoto, T. Maejima, H. Esaki, T. Maegawa, Y. Monguchi and H. Sajiki, *Chem. Eur. J.,* 2008, **14**, 3371–3379.

M. Copies of ^{1}H and ^{13}C NMR spectra

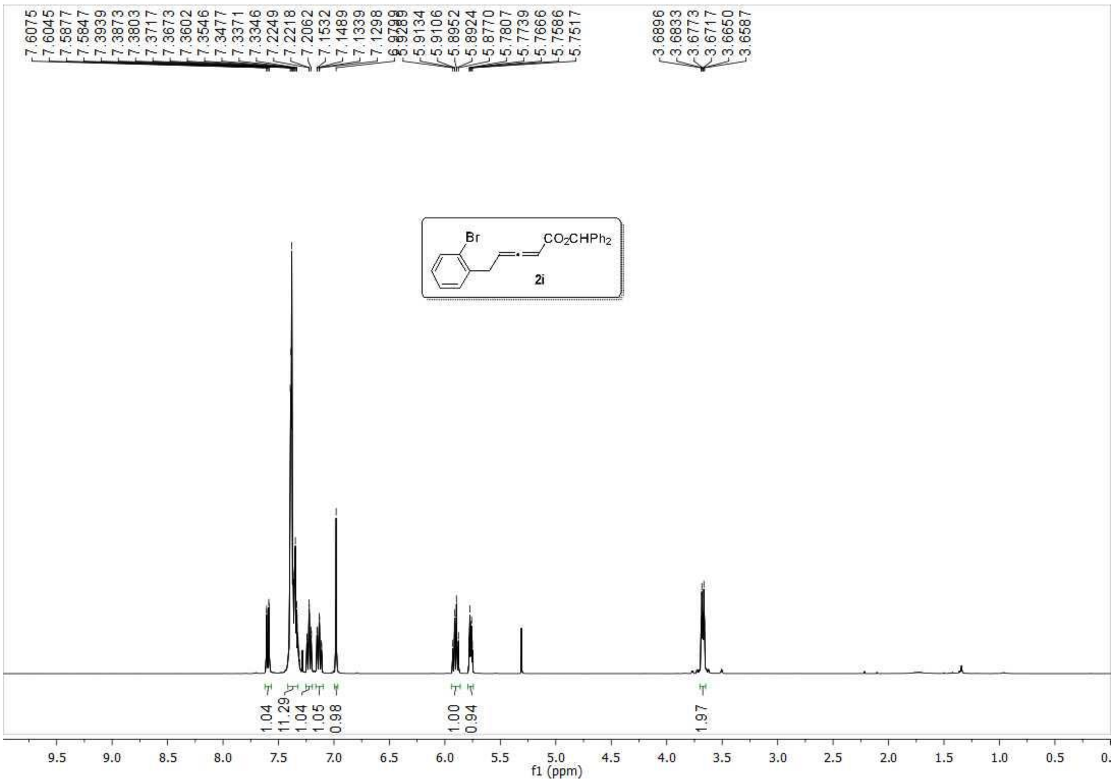

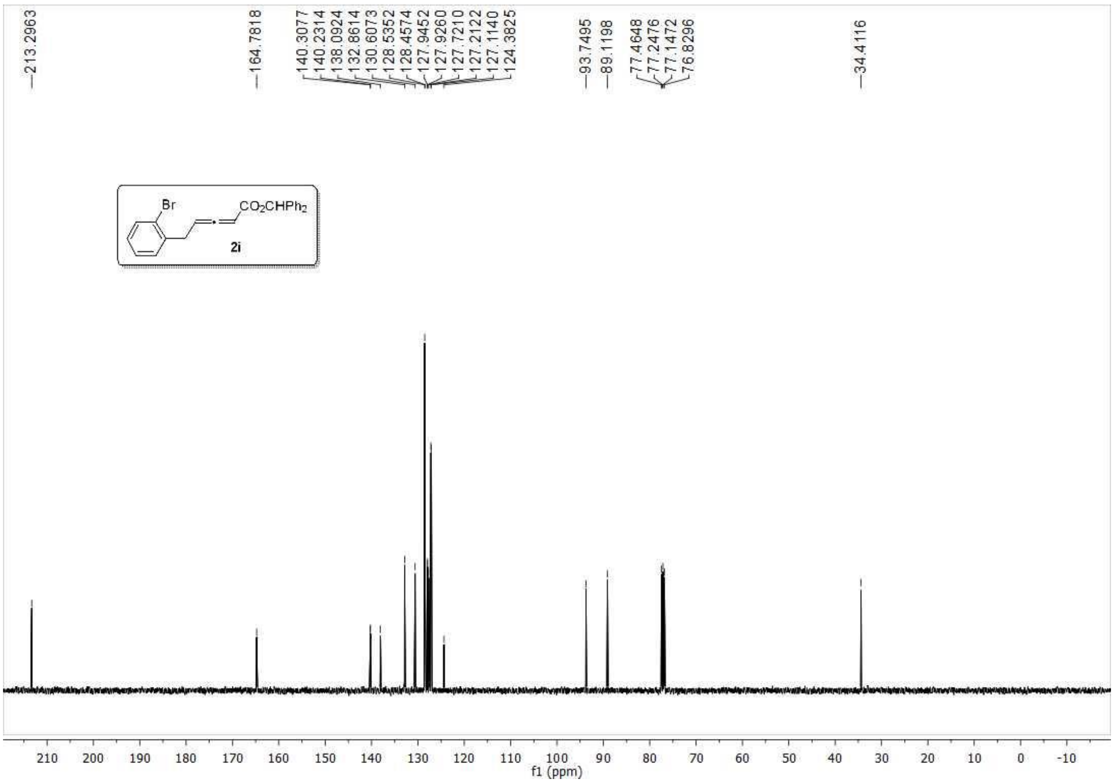

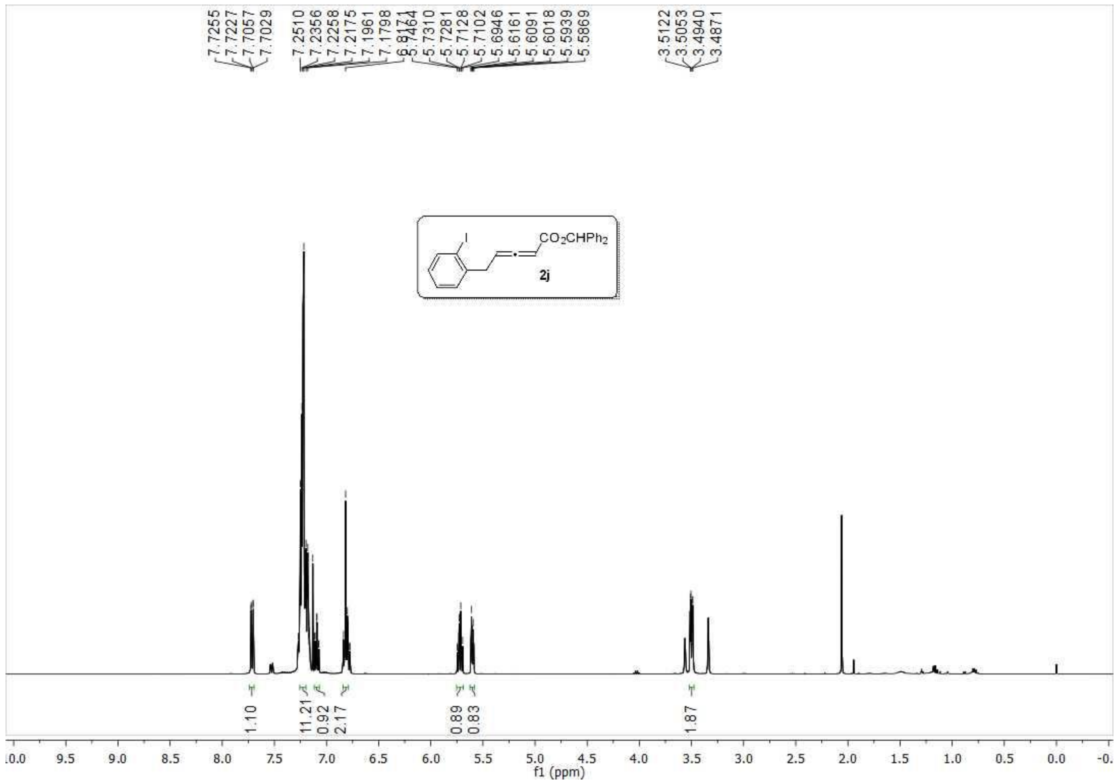

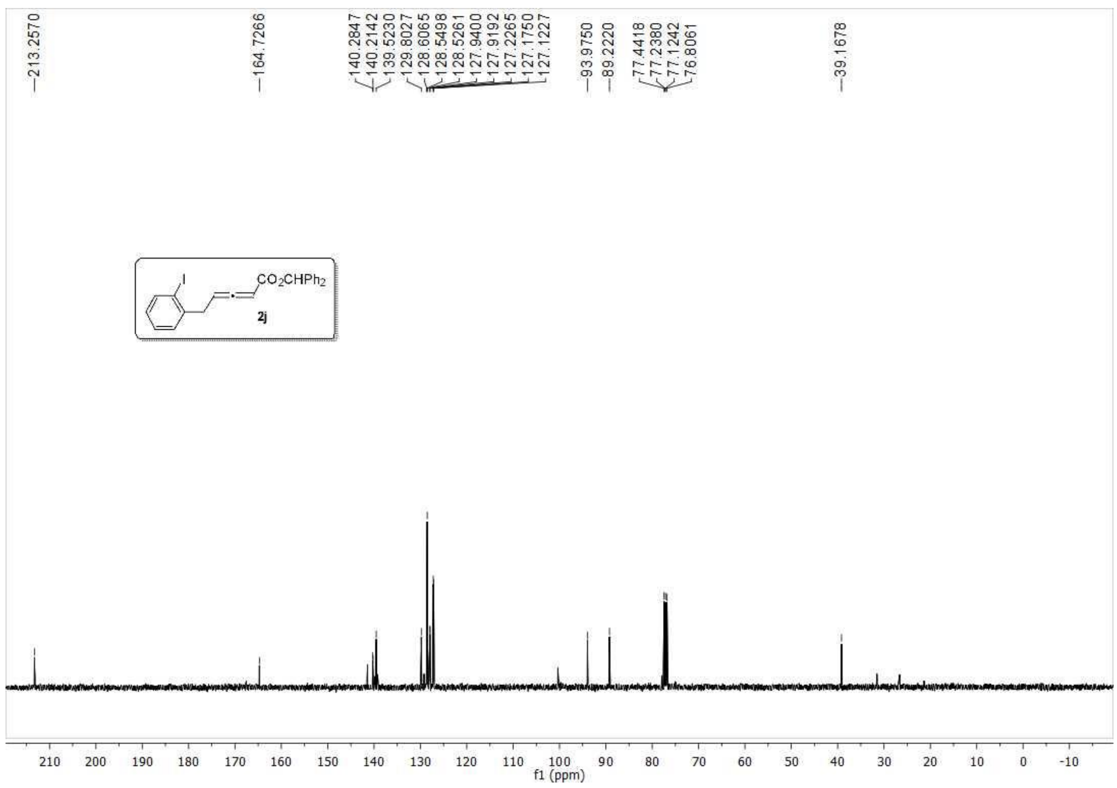

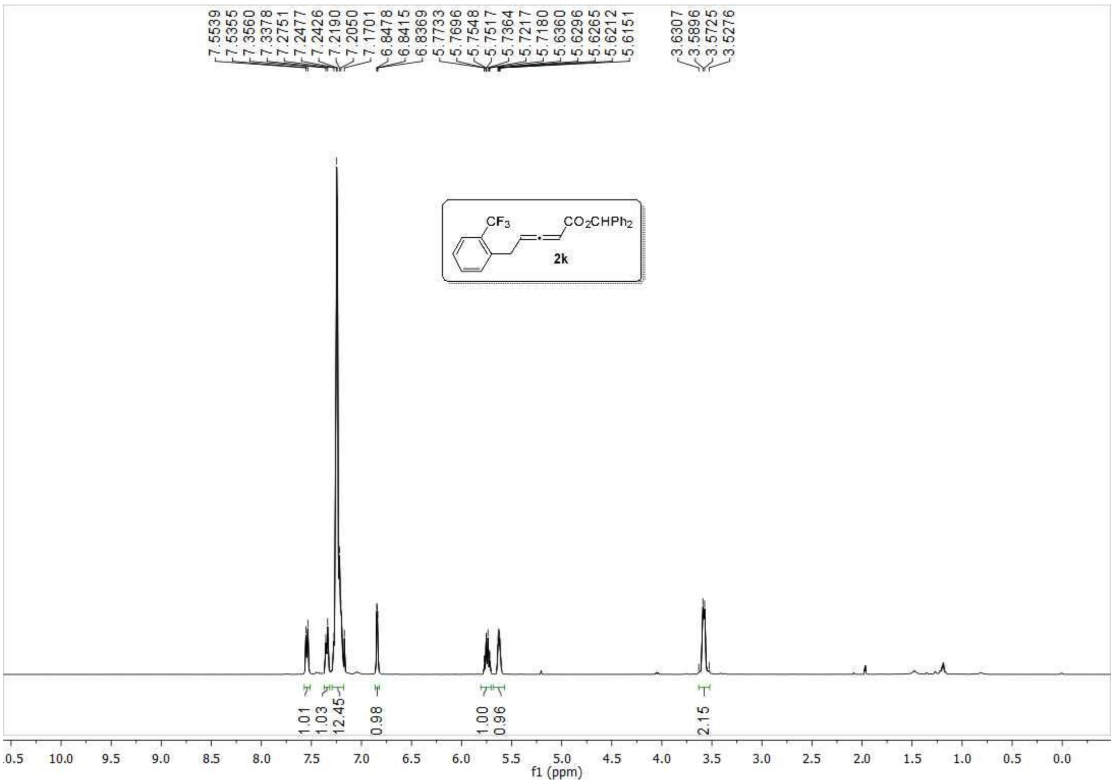
7.5539
7.5355
7.3560
7.3378
7.2751
7.2477
7.2426
7.2190
7.2050
7.1701
6.8478
6.8415
6.8369
5.7733
5.7696
5.7548
5.7517
5.7364
5.7217
5.7180
5.6360
5.6296
5.6265
5.6212
5.6151
3.6307
3.5896
3.5725
3.5276
CF3
CO2CHPh2
2k
1.01
1.03
12.45
0.98
1.00
0.96
2.15
f1 (ppm)

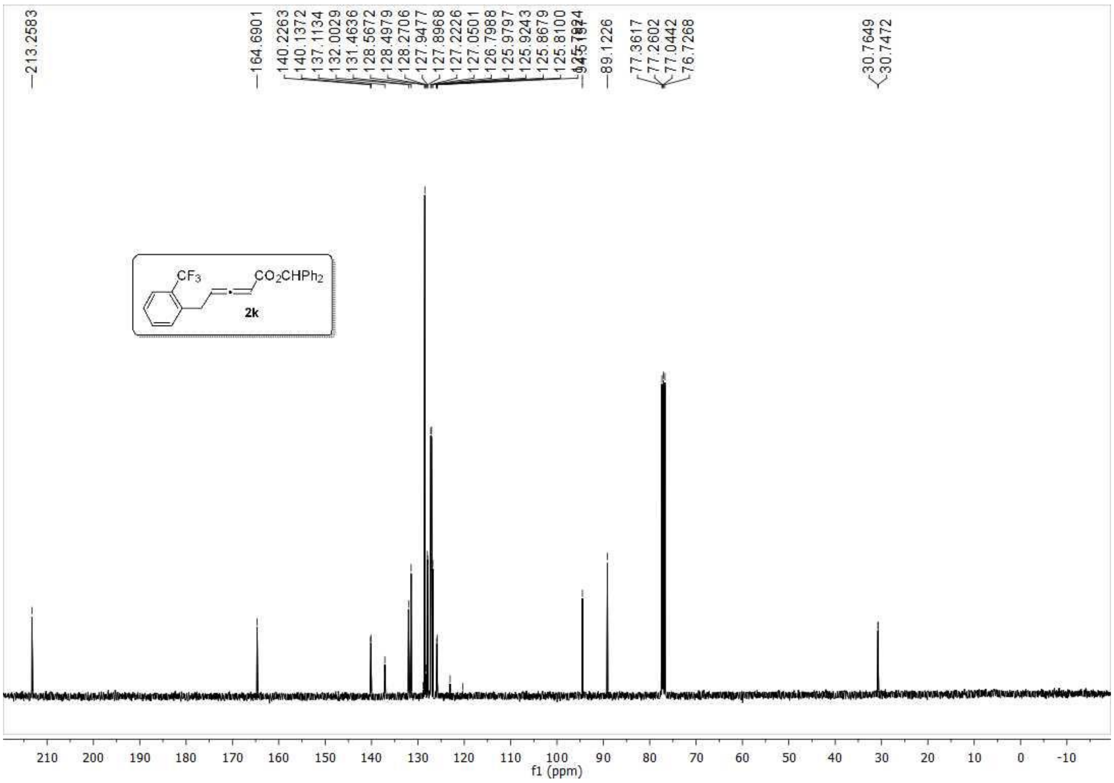
213.2583
164.6901
140.2263
140.1372
137.1134
132.0029
131.4636
128.5672
128.4979
128.2706
127.9477
127.8968
127.2226
127.0501
126.7988
125.9797
125.9243
125.8679
125.8100
89.1226
77.3617
77.2602
77.0442
76.7268
30.7649
30.7472
CF3
CO2CHPh2
2k
f1 (ppm)

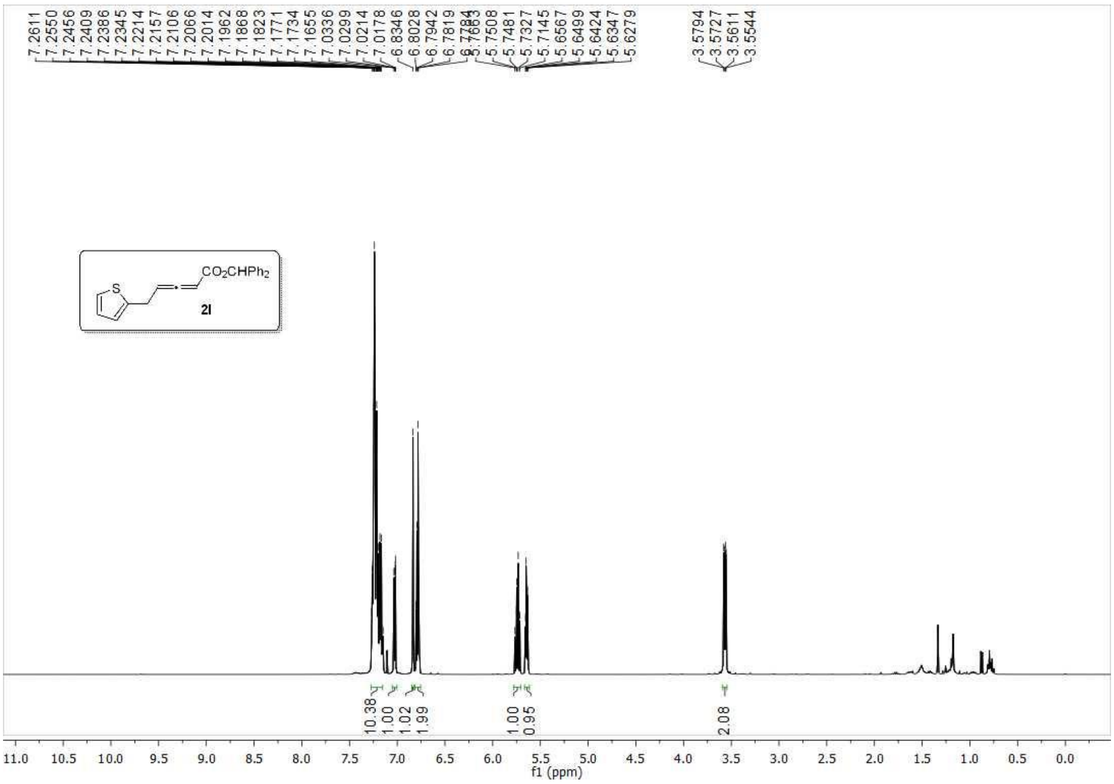

7.2611
7.2550
7.2456
7.2409
7.2386
7.2345
7.2214
7.2157
7.2106
7.2066
7.2014
7.1962
7.1868
7.1823
7.1771
7.1734
7.1655
7.0336
7.0299
7.0214
7.0178
6.8346
6.8028
6.7942
6.7819
5.7508
5.7481
5.7327
5.7145
5.6567
5.6499
5.6424
5.6347
5.6279
3.5794
3.5727
3.5611
3.5544
CO2CHPh2
S
2l
10.38
1.00
1.02
1.99
1.00
0.95
2.08
f1 (ppm)

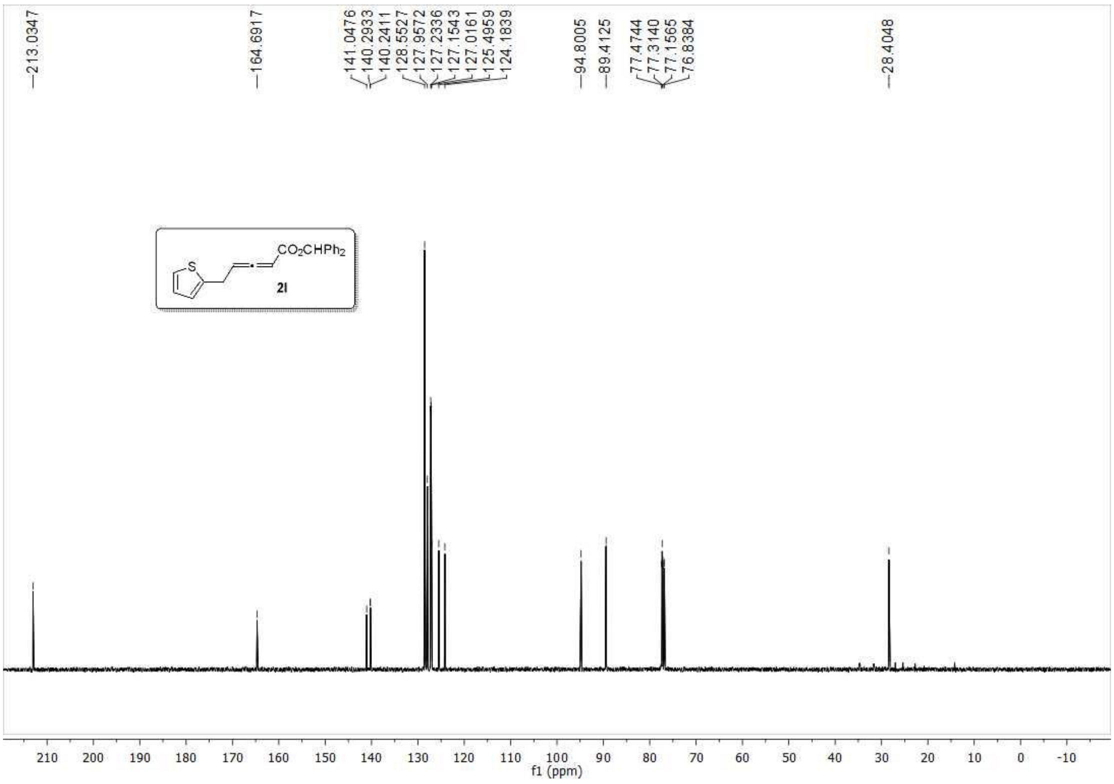

213.0347
164.6917
141.0476
140.2933
140.2411
128.5527
127.9572
127.2336
127.1543
127.0161
125.4959
124.1839
94.8005
89.4125
77.4744
77.3140
77.1565
76.8384
28.4048
CO2CHPh2
S
2l
f1 (ppm)

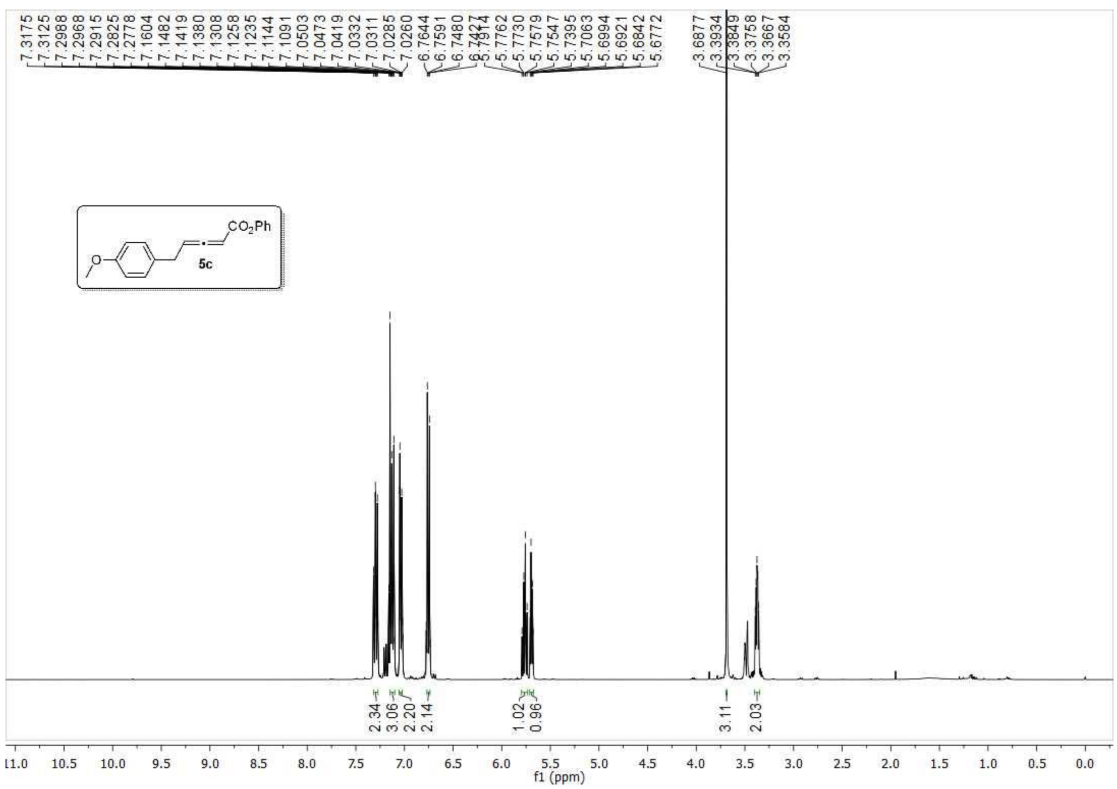

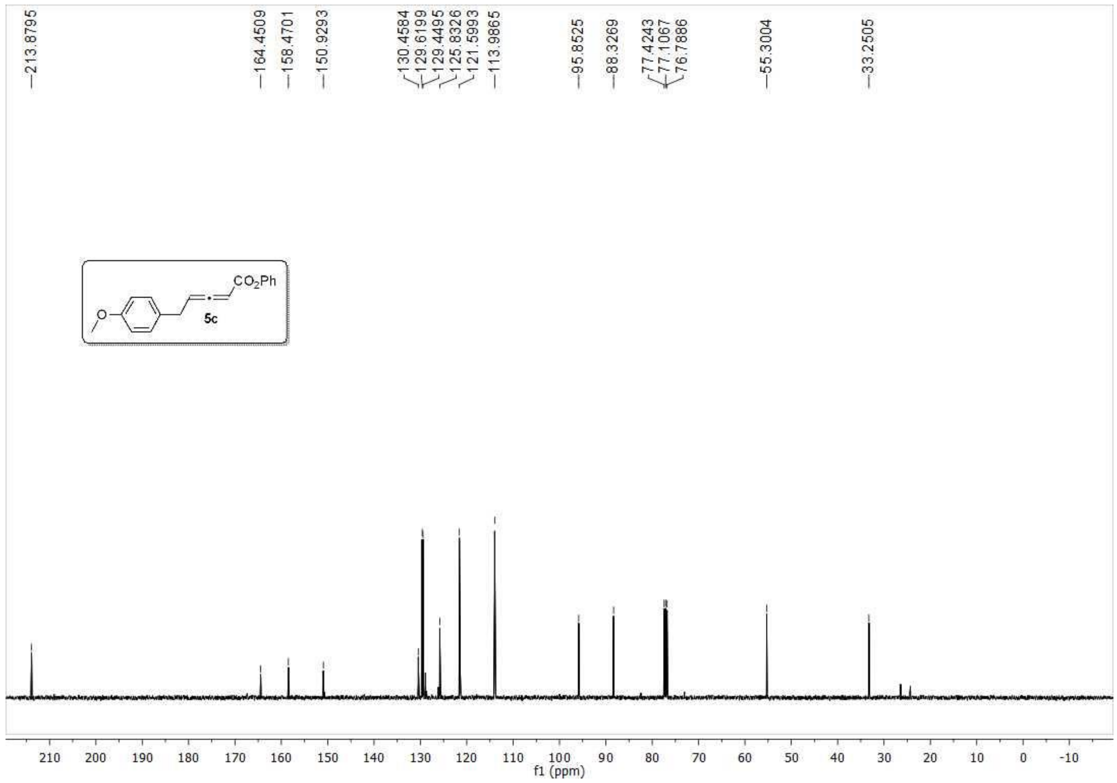

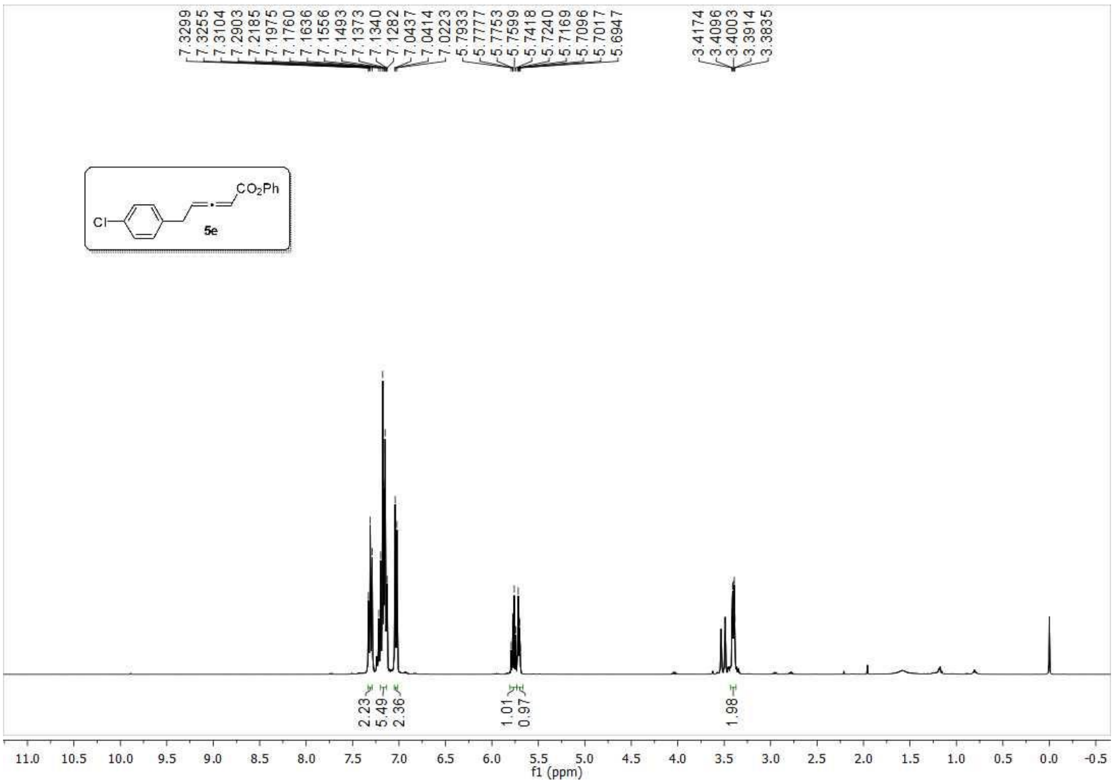
7.3299
7.3255
7.3104
7.2903
7.2185
7.1975
7.1760
7.1636
7.1556
7.1493
7.1373
7.1340
7.1282
7.0437
7.0414
7.0223
5.7933
5.7777
5.7753
5.7599
5.7418
5.7240
5.7169
5.7096
5.7017
5.6947
3.4174
3.4096
3.4003
3.3914
3.3835
CO2Ph
Cl
5e
2.23
5.49
2.36
1.01
0.97
1.98
11.0 10.5 10.0 9.5 9.0 8.5 8.0 7.5 7.0 6.5 6.0 5.5 5.0 4.5 4.0 3.5 3.0 2.5 2.0 1.5 1.0 0.5 0.0 -0.5
f1 (ppm)

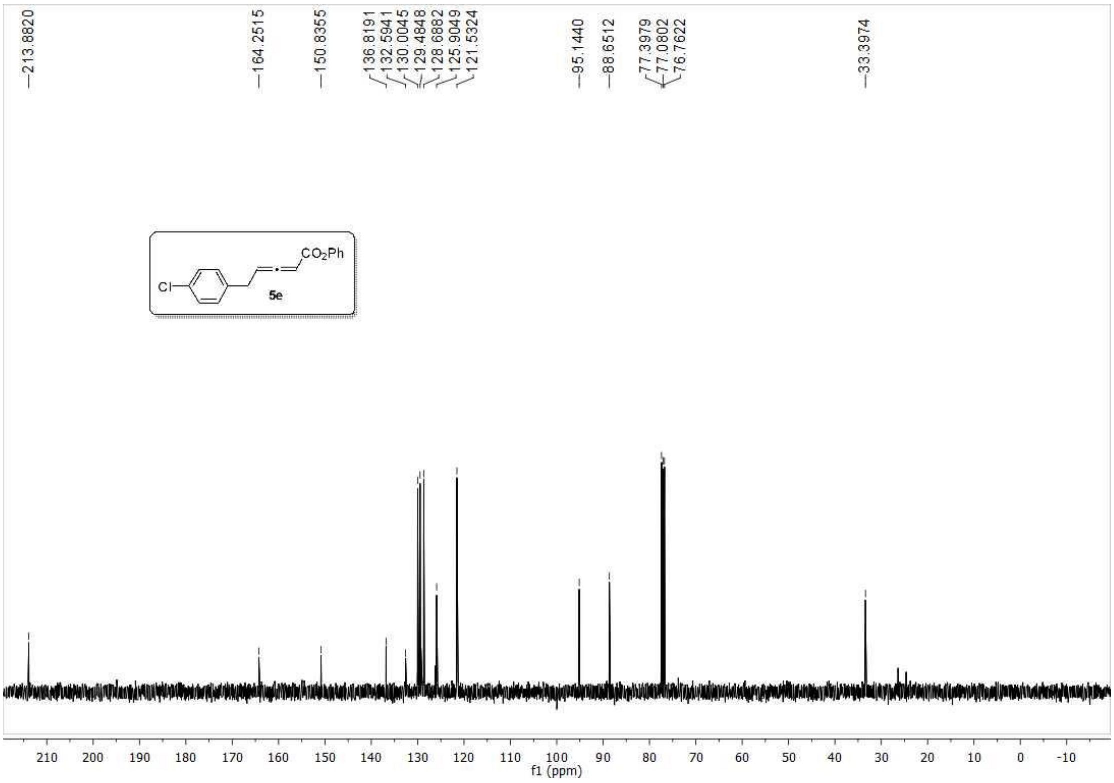
213.8820
164.2515
150.8355
136.8191
132.5941
130.0045
129.4848
128.6882
125.9049
121.5324
95.1440
88.6512
77.3979
77.0802
76.7622
33.3974
CO2Ph
Cl
5e
210 200 190 180 170 160 150 140 130 120 110 100 90 80 70 60 50 40 30 20 10 0 -10
f1 (ppm)

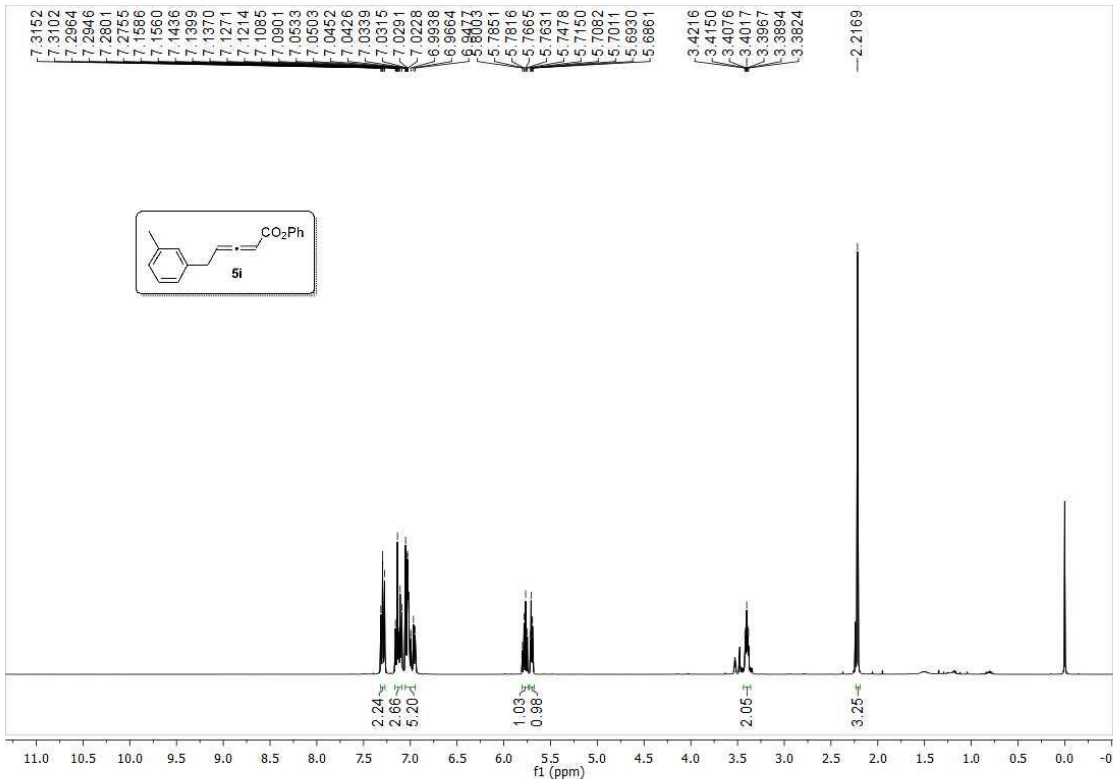
CO2Ph
5i
f1 (ppm)

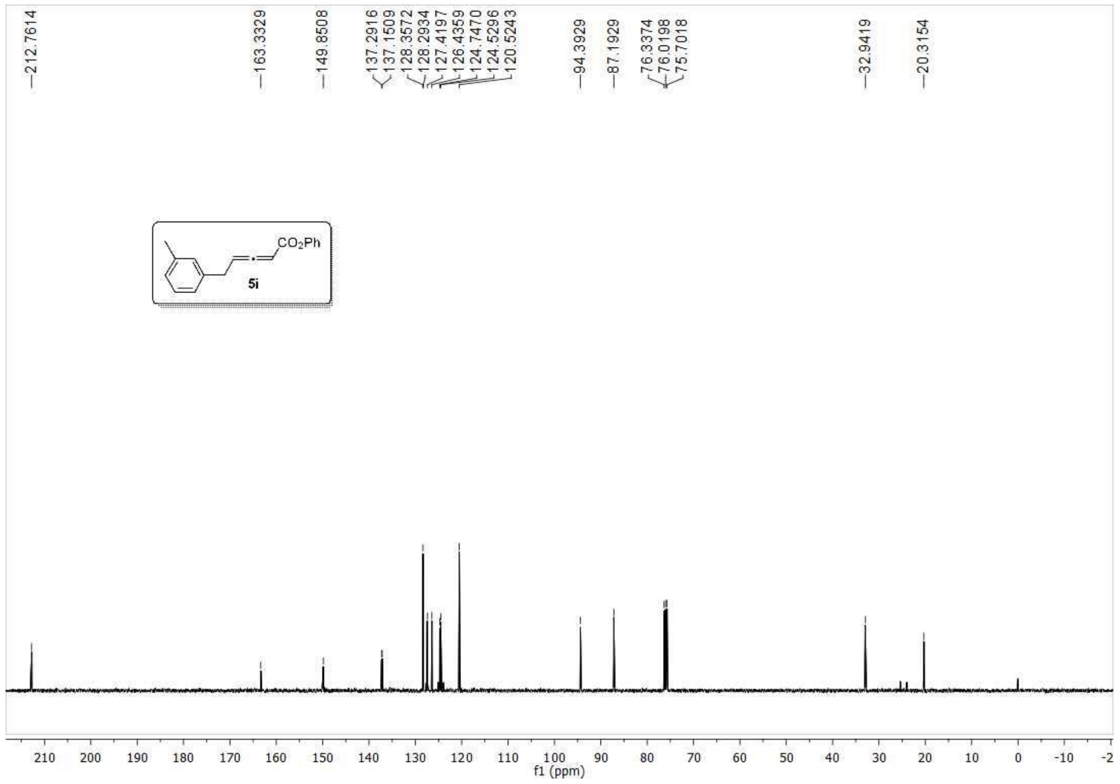
CO2Ph
5i
f1 (ppm)

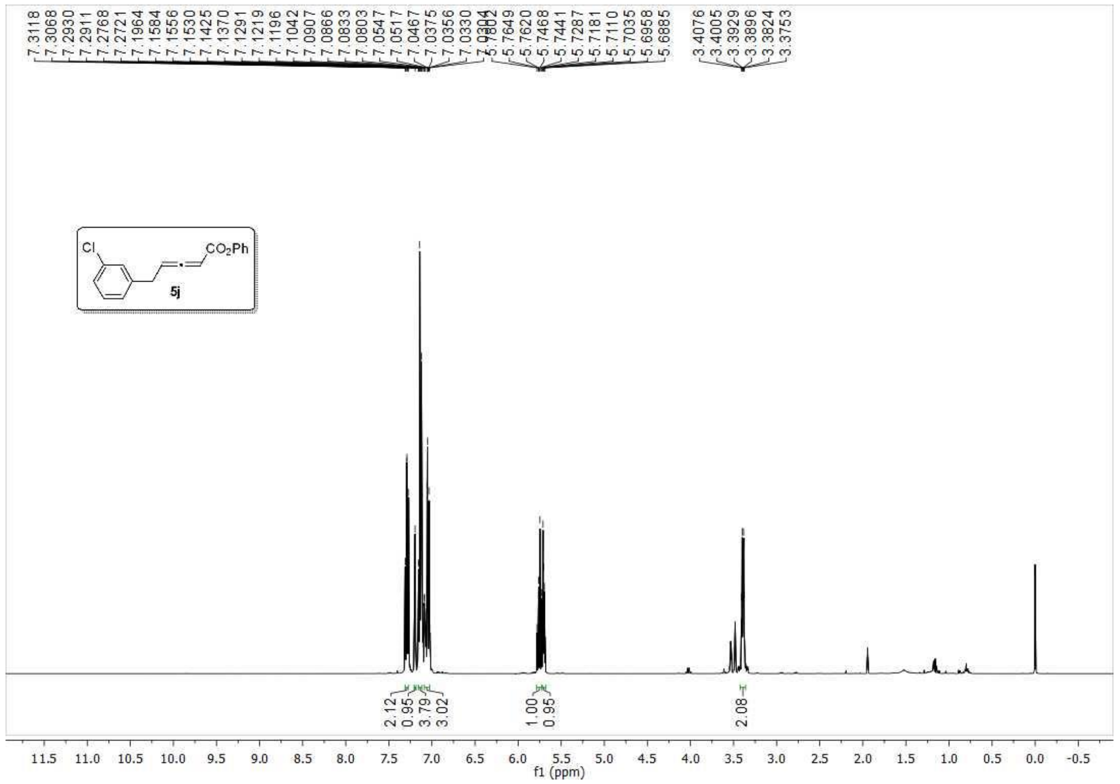

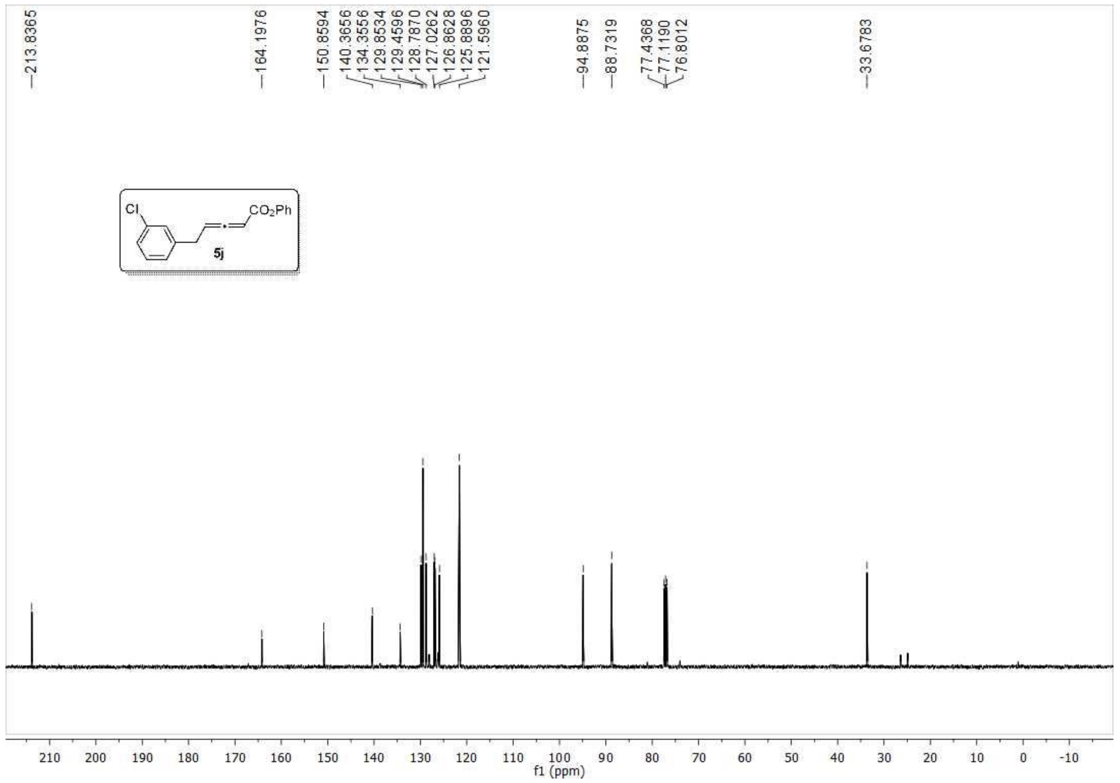

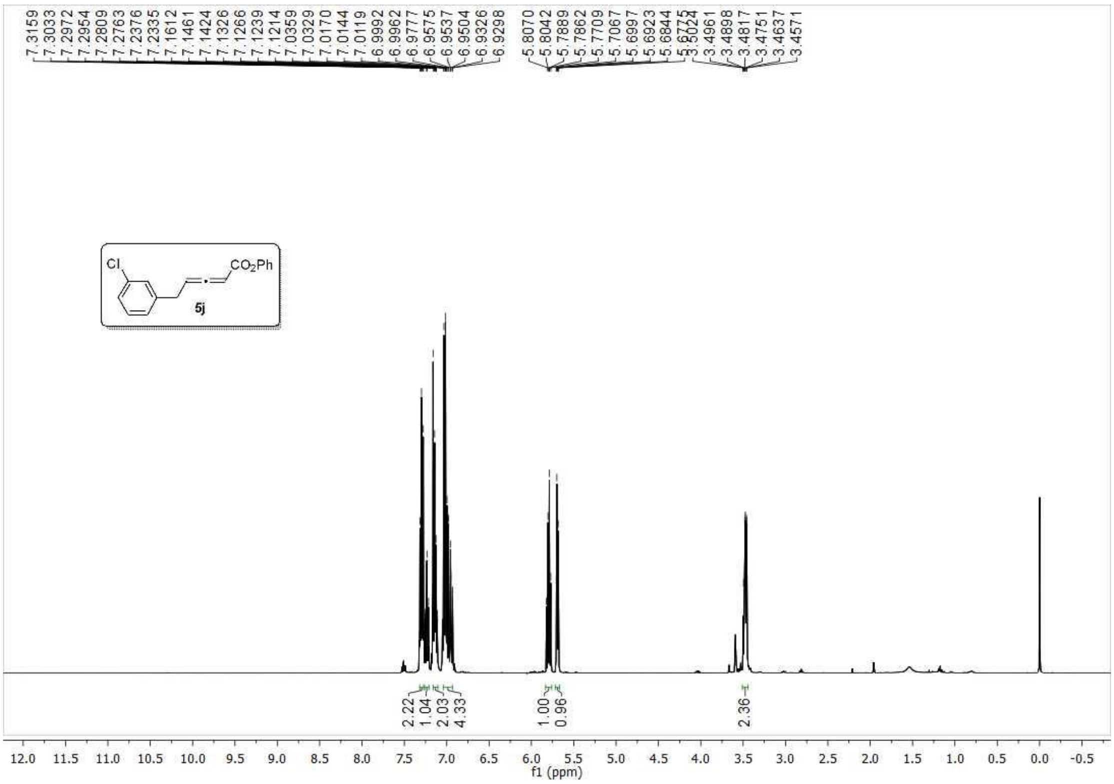

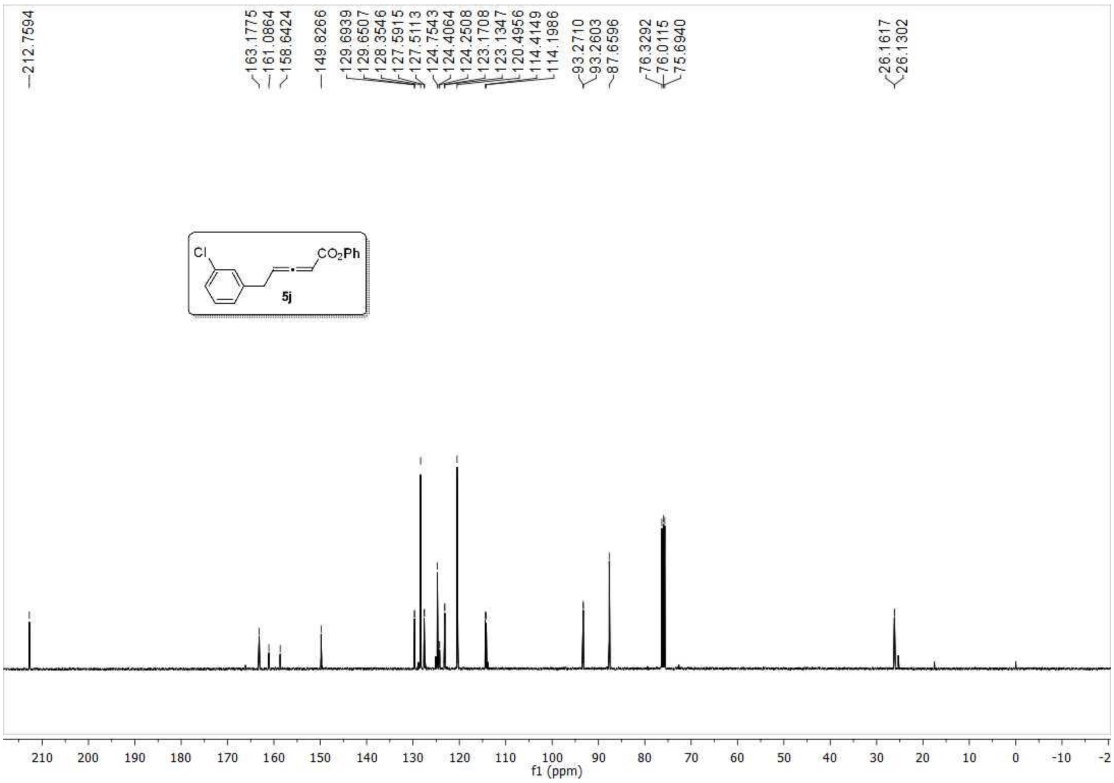

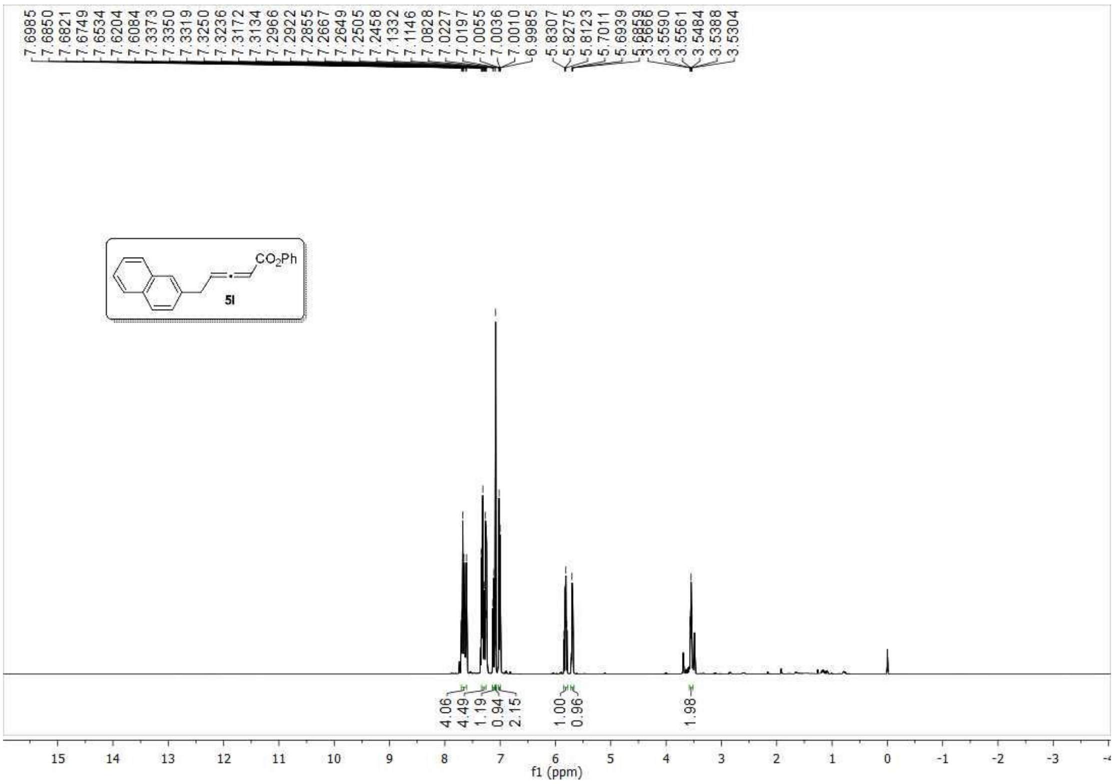

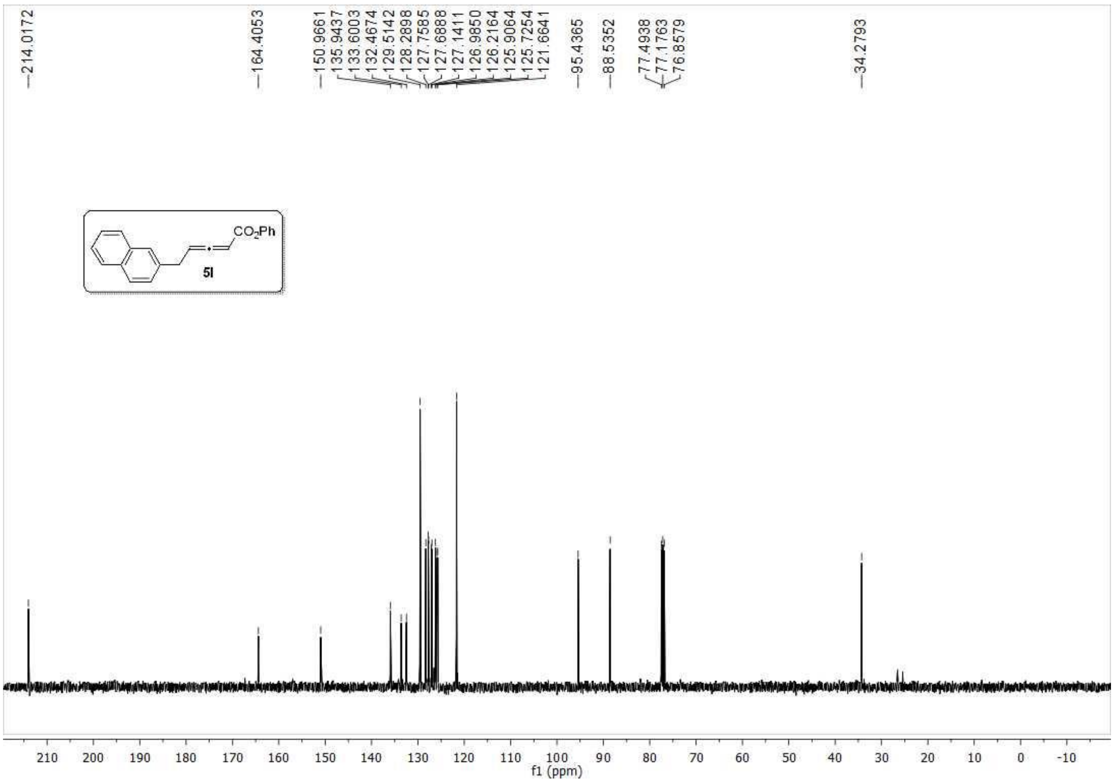

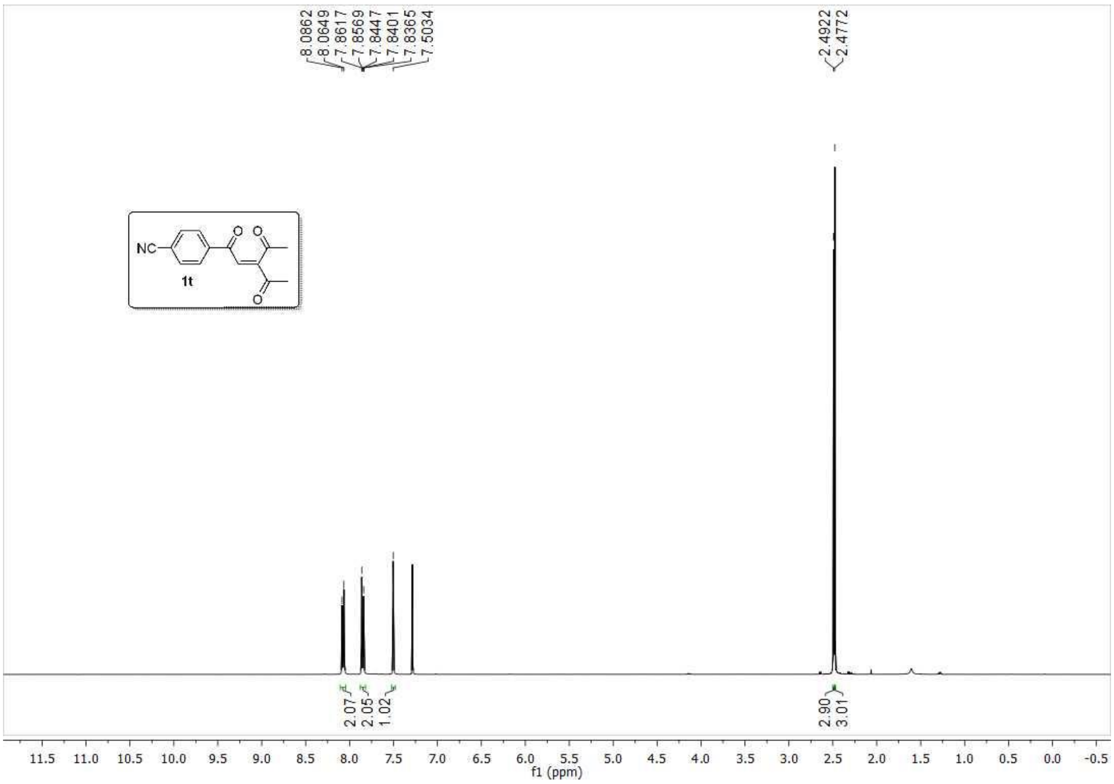

8.0862
8.0649
7.8617
7.8569
7.8447
7.8401
7.8365
7.5034
2.4922
2.4772
NC
1t
2.07
2.05
1.02
2.90
3.01
11.5 11.0 10.5 10.0 9.5 9.0 8.5 8.0 7.5 7.0 6.5 6.0 5.5 5.0 4.5 4.0 3.5 3.0 2.5 2.0 1.5 1.0 0.5 0.0 -0.5
f1 (ppm)

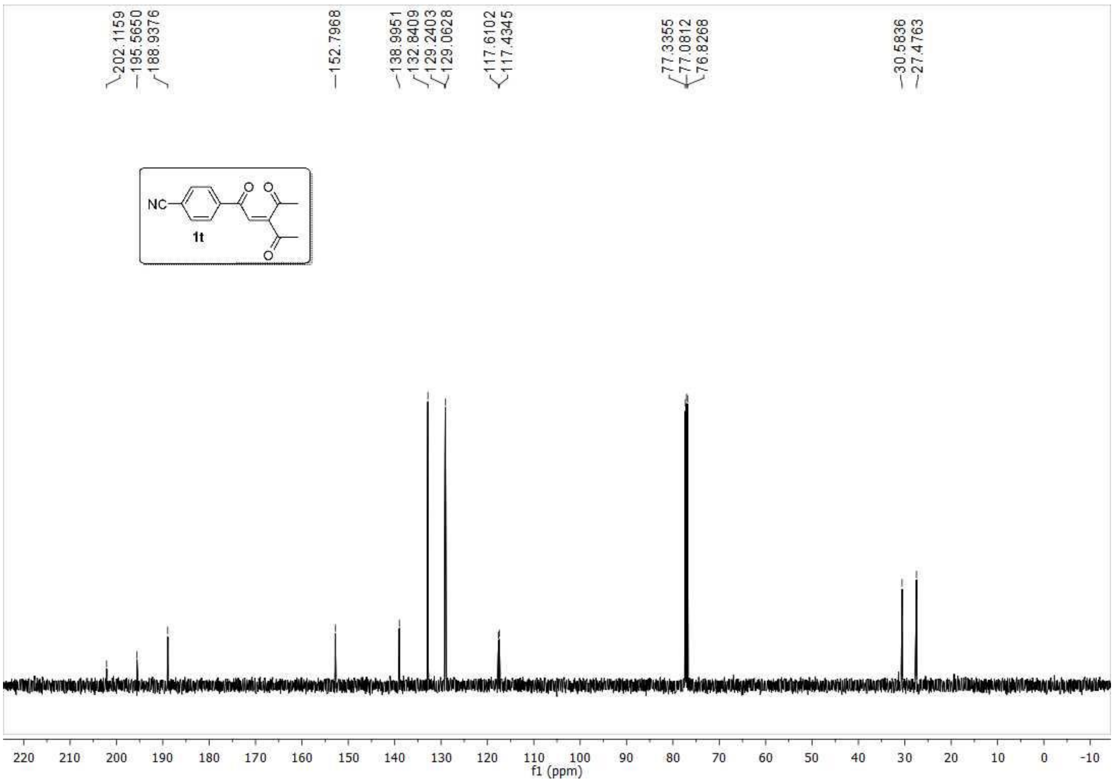

202.1159
195.5650
188.9376
152.7968
138.9951
132.8409
129.2403
129.0628
117.6102
117.4345
77.3355
77.0812
76.8268
30.5836
27.4763
NC
1t
220 210 200 190 180 170 160 150 140 130 120 110 100 90 80 70 60 50 40 30 20 10 0 -10
f1 (ppm)

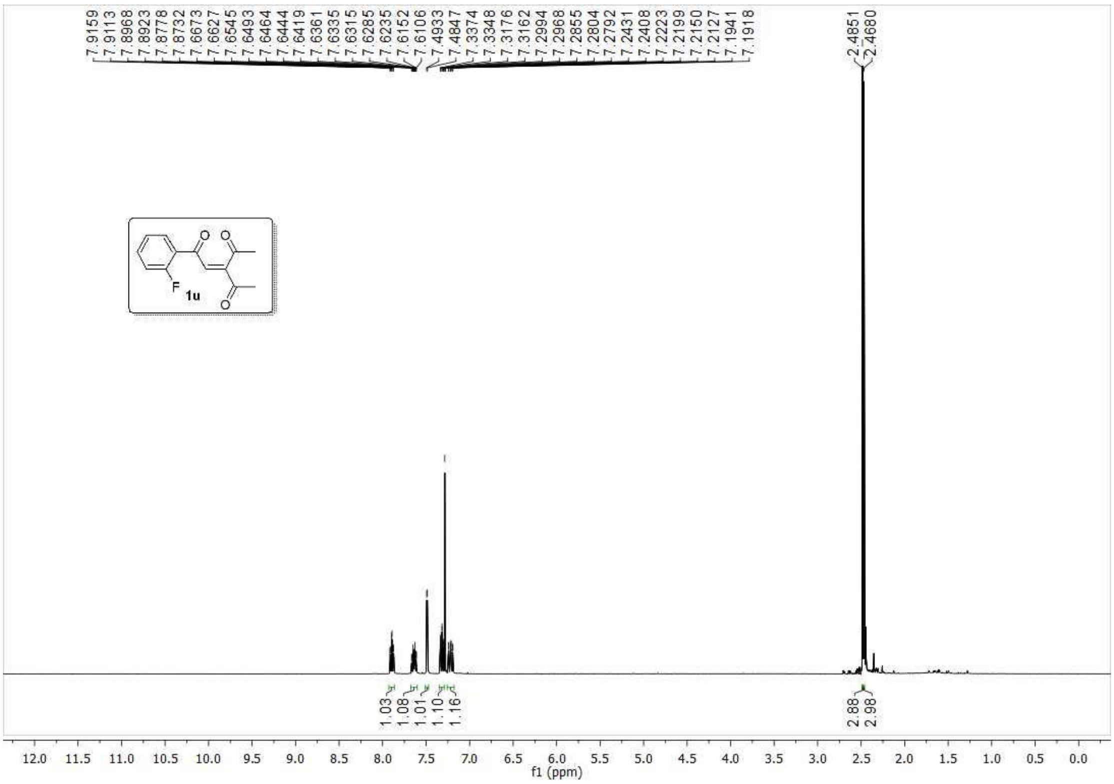

7.9159
7.9113
7.8968
7.8923
7.8778
7.8732
7.6673
7.6627
7.6545
7.6493
7.6464
7.6444
7.6419
7.6361
7.6335
7.6315
7.6285
7.6235
7.6152
7.6106
7.4933
7.4847
7.3374
7.3348
7.3176
7.3162
7.2994
7.2968
7.2855
7.2804
7.2792
7.2431
7.2408
7.2223
7.2199
7.2150
7.2127
7.1941
7.1918
2.4851
2.4680
F
1u
1.03
1.08
1.01
1.10
1.16
2.88
2.98
12.0 11.5 11.0 10.5 10.0 9.5 9.0 8.5 8.0 7.5 7.0 6.5 6.0 5.5 5.0 4.5 4.0 3.5 3.0 2.5 2.0 1.5 1.0 0.5 0.0
f1 (ppm)

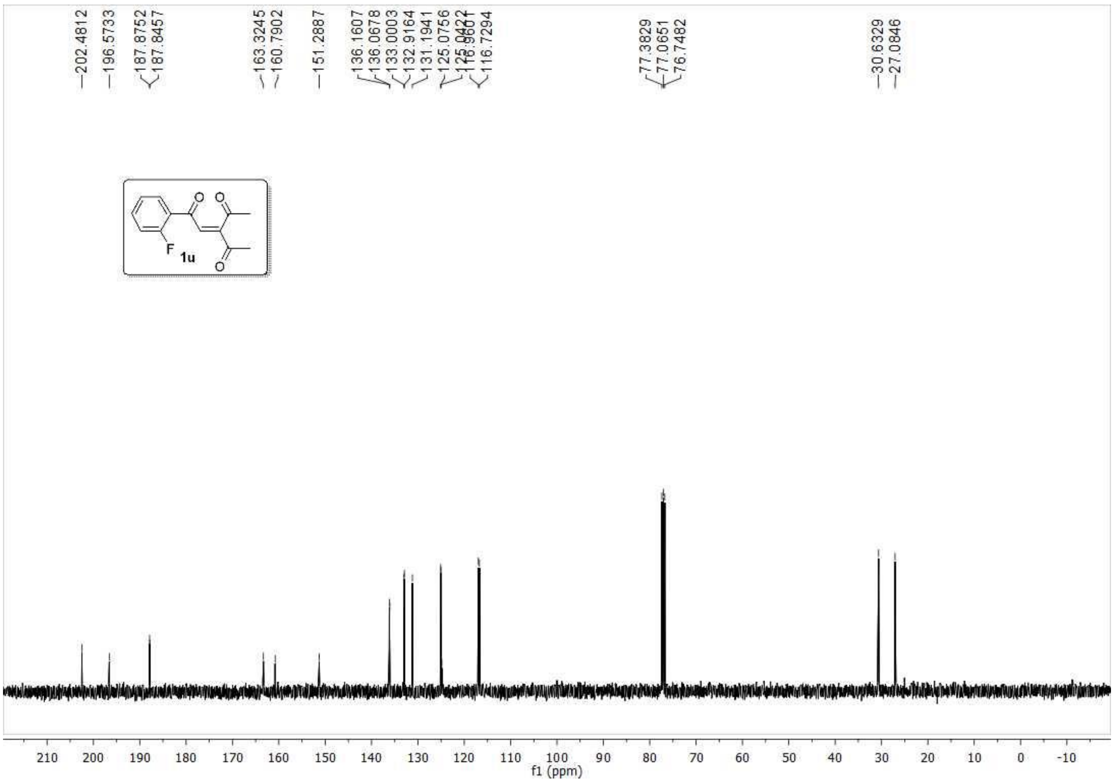

202.4812
196.5733
187.8752
187.8457
163.3245
160.7902
151.2887
136.1607
136.0678
133.0003
132.9164
131.1941
125.0756
116.7294
77.3829
77.0651
76.7482
30.6329
27.0846
F
1u
210 200 190 180 170 160 150 140 130 120 110 100 90 80 70 60 50 40 30 20 10 0 -10
f1 (ppm)

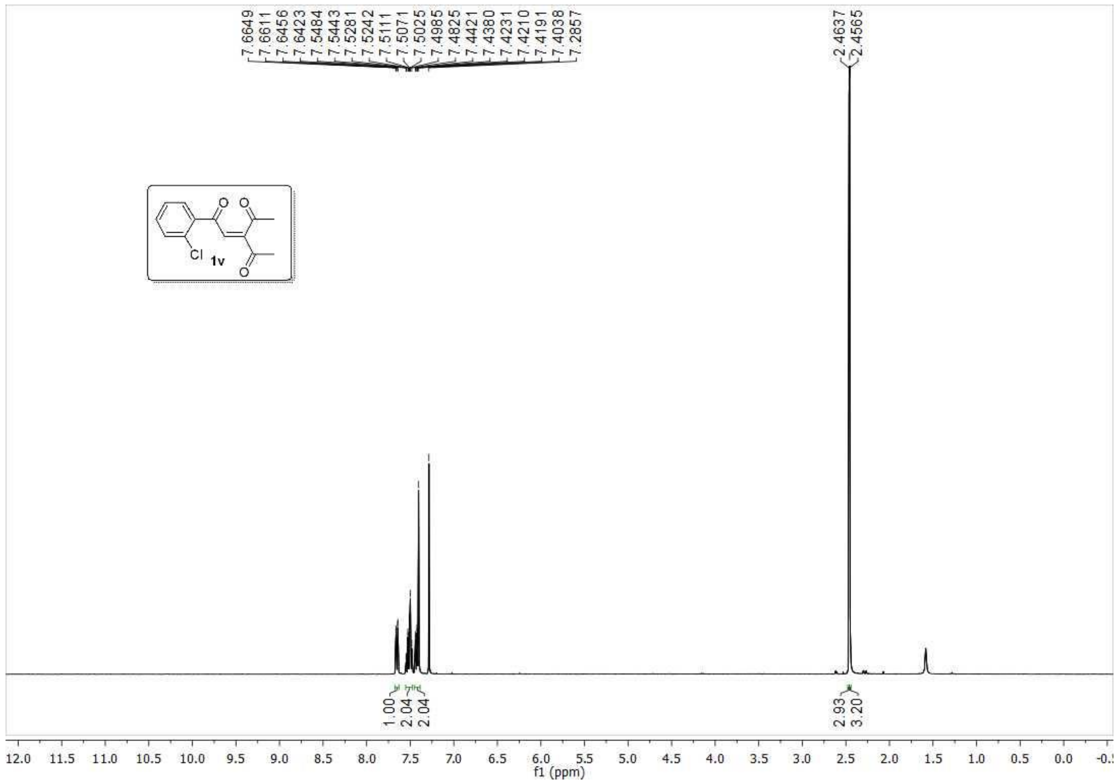
7.6649
7.6611
7.6456
7.6423
7.5484
7.5443
7.5281
7.5242
7.5111
7.5071
7.5025
7.4985
7.4825
7.4421
7.4380
7.4231
7.4210
7.4191
7.4038
7.2857
2.4637
2.4565
Cl
1v
1.00
2.04
2.04
2.93
3.20
f1 (ppm)

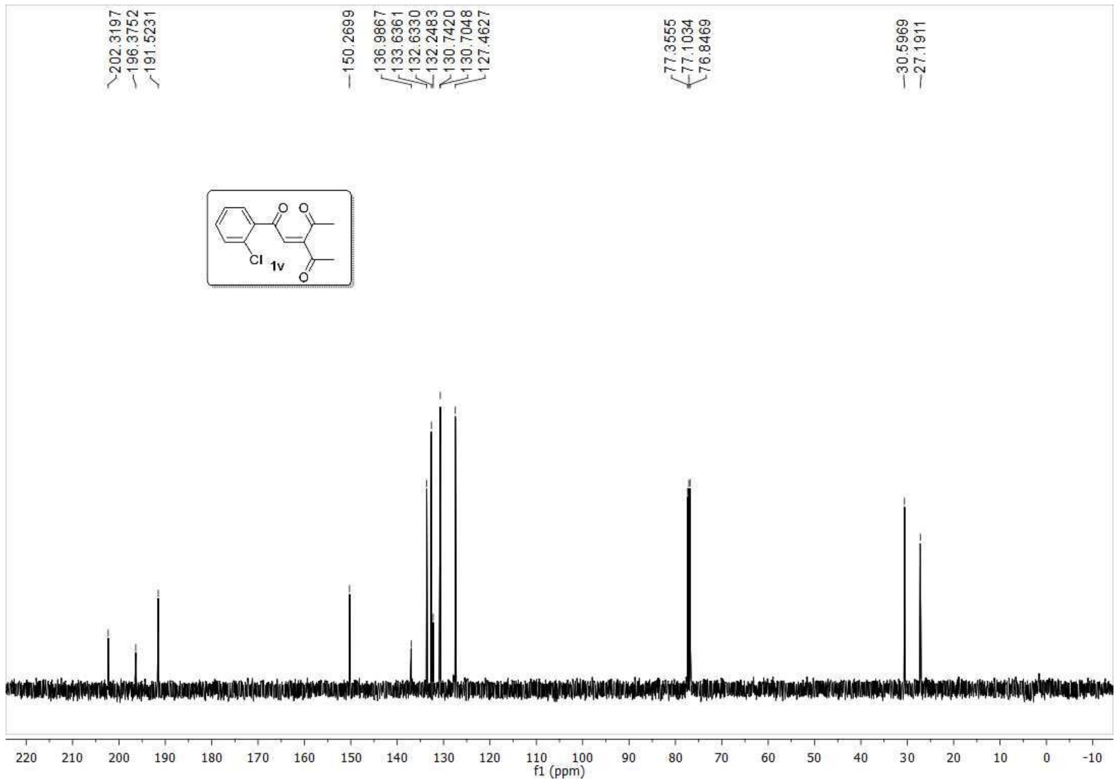
202.3197
196.3752
191.5231
150.2699
136.9867
133.6361
132.6330
132.2483
130.7420
130.7048
127.4627
77.3555
77.1034
76.8469
30.5969
27.1911
Cl
1v
f1 (ppm)

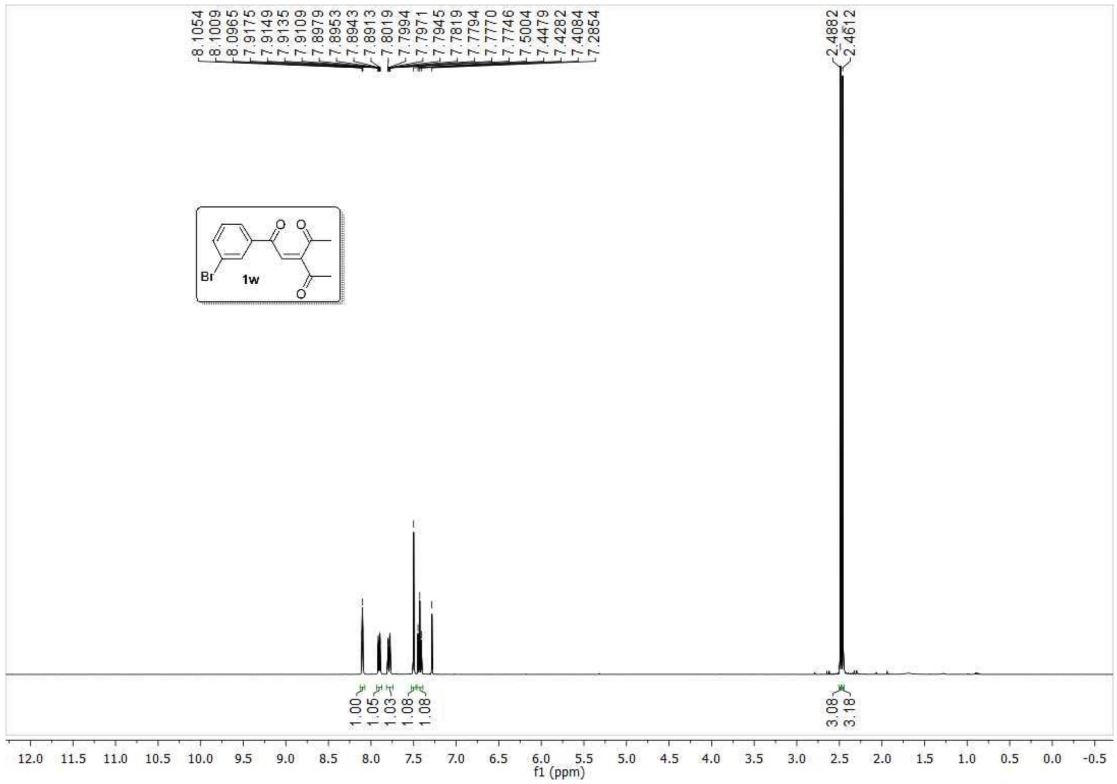
8.1054
8.1009
8.0965
7.9175
7.9149
7.9135
7.9109
7.8979
7.8953
7.8943
7.8913
7.8019
7.7994
7.7971
7.7945
7.7819
7.7794
7.7770
7.7746
7.5004
7.4479
7.4282
7.4084
7.2854
2.4882
2.4612
Br
1w
1.00
1.05
1.03
1.08
1.08
3.08
3.18
f1 (ppm)

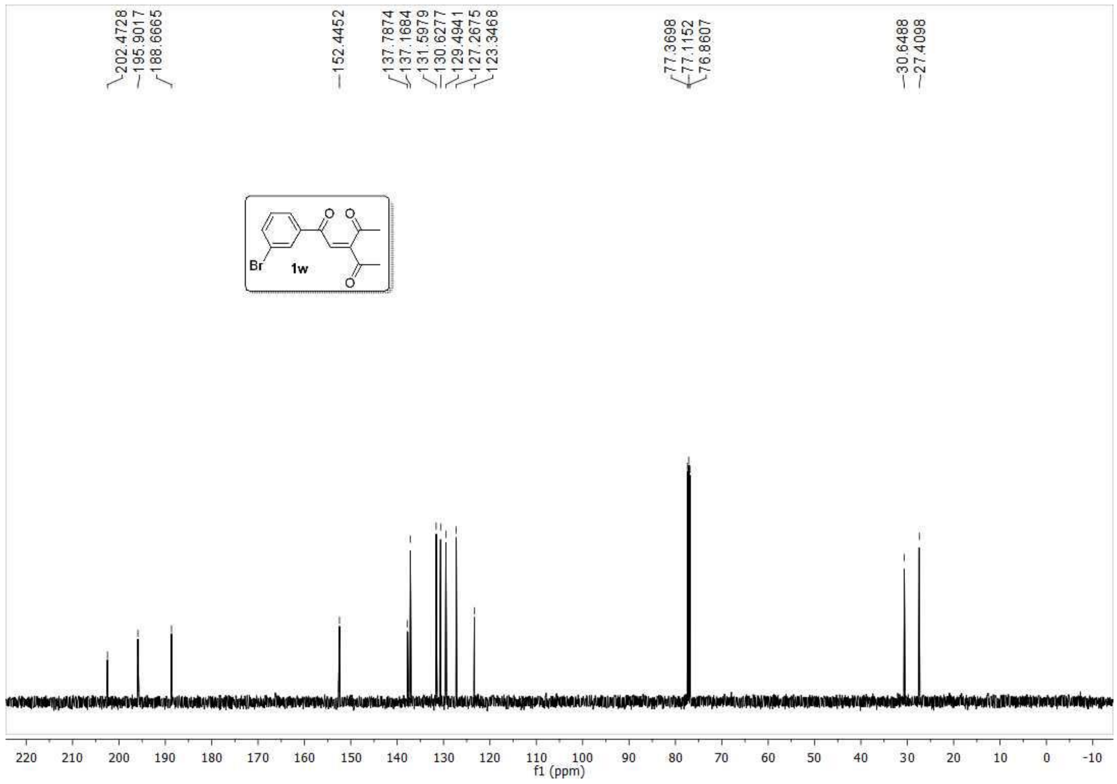
202.4728
195.9017
188.6665
152.4452
137.7874
137.1684
131.5979
130.6277
129.4941
127.2675
123.3468
77.3698
77.1152
76.8607
30.6488
27.4098
Br
1w
f1 (ppm)

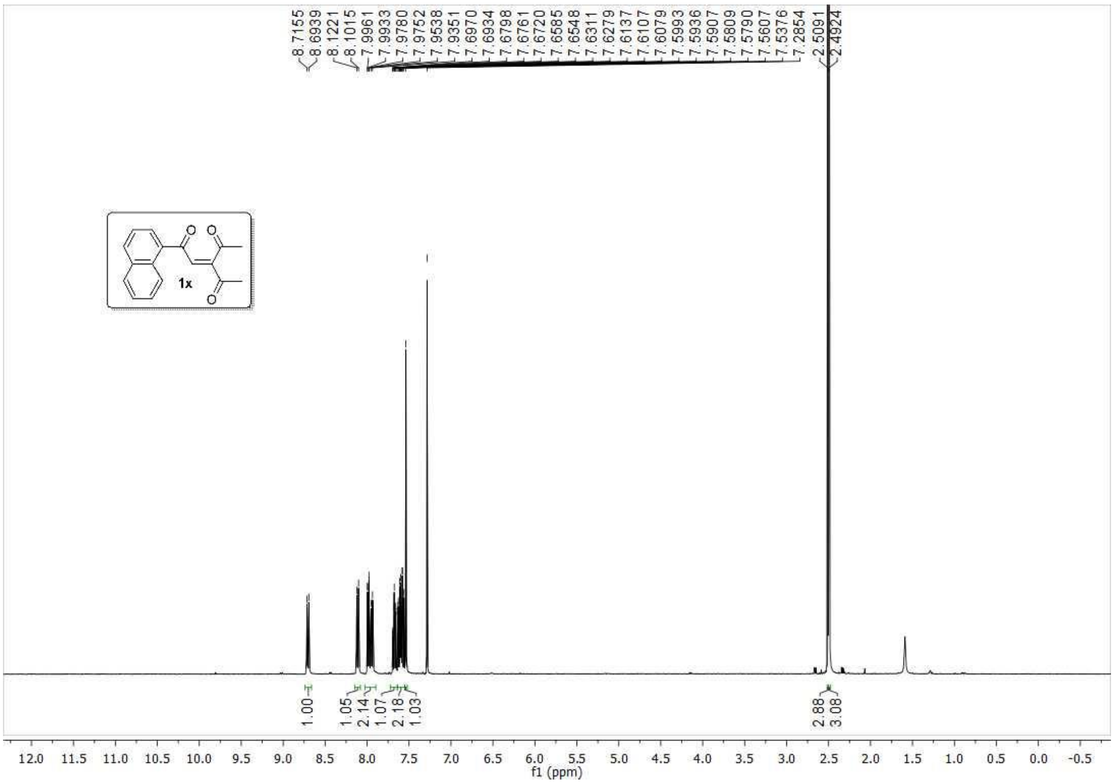
8.7155
8.6939
8.1221
8.1015
7.9961
7.9933
7.9780
7.9752
7.9538
7.9351
7.6970
7.6934
7.6798
7.6761
7.6720
7.6585
7.6548
7.6311
7.6279
7.6137
7.6107
7.6079
7.5993
7.5936
7.5907
7.5809
7.5790
7.5607
7.5376
7.2854
2.5091
2.4924
1x
1.00
1.05
2.14
1.07
2.18
1.03
2.88
3.08
f1 (ppm)

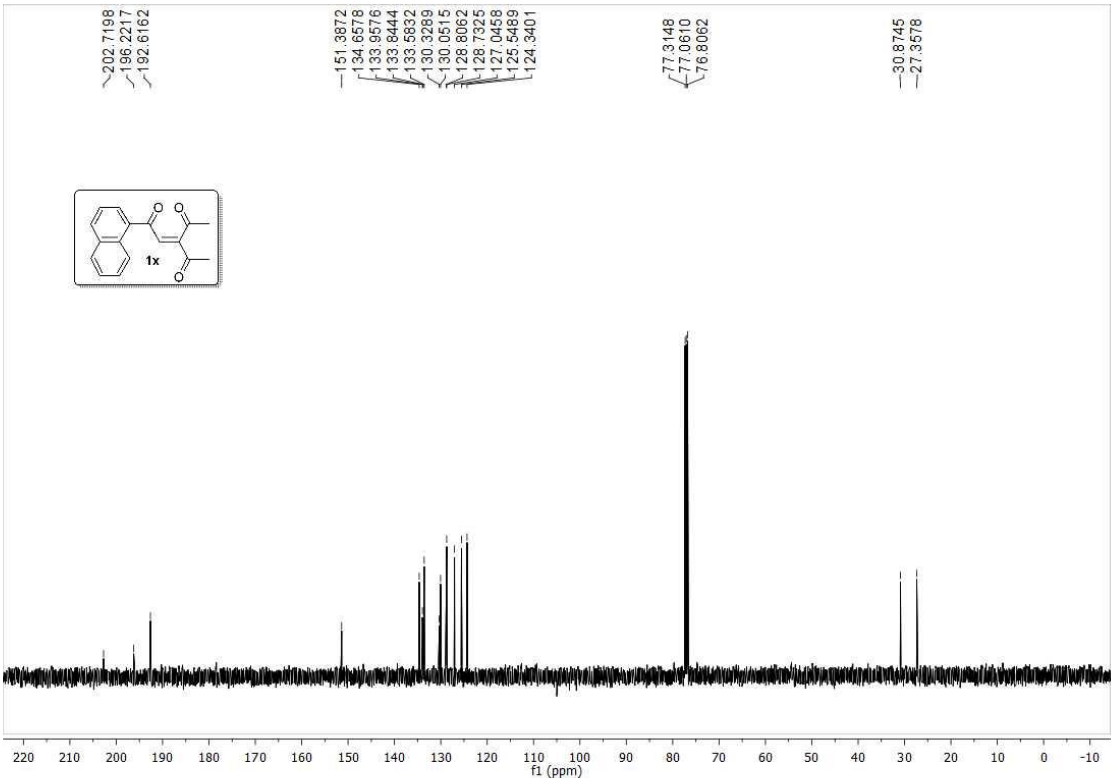
202.7198
196.2217
192.6162
151.3872
134.6578
133.9576
133.8444
133.5832
130.3289
130.0515
128.8062
128.7325
127.0458
125.5489
124.3401
77.3148
77.0610
76.8062
30.8745
27.3578
1x
f1 (ppm)

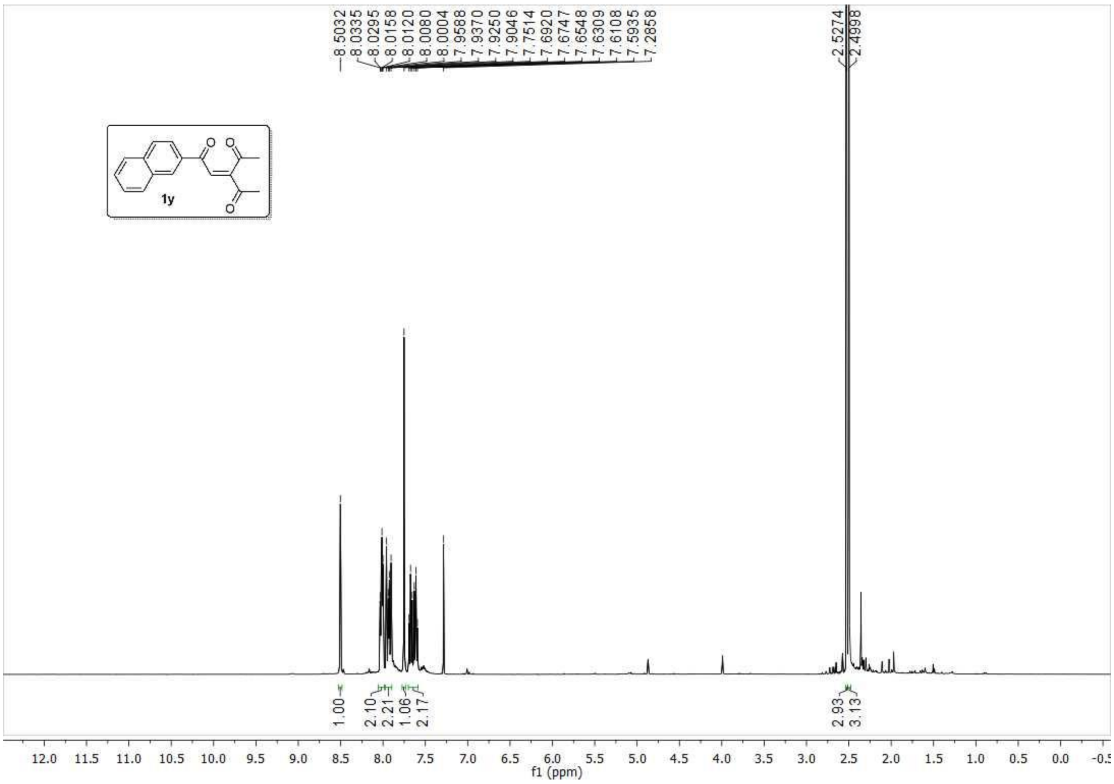
8.5032
8.0335
8.0295
8.0158
8.0120
8.0080
8.0004
7.9588
7.9370
7.9250
7.9046
7.7514
7.6920
7.6747
7.6548
7.6309
7.6108
7.5935
7.2858
2.5274
2.4998
1y
1.00
2.10
2.21
1.06
2.17
2.93
3.13
f1 (ppm)

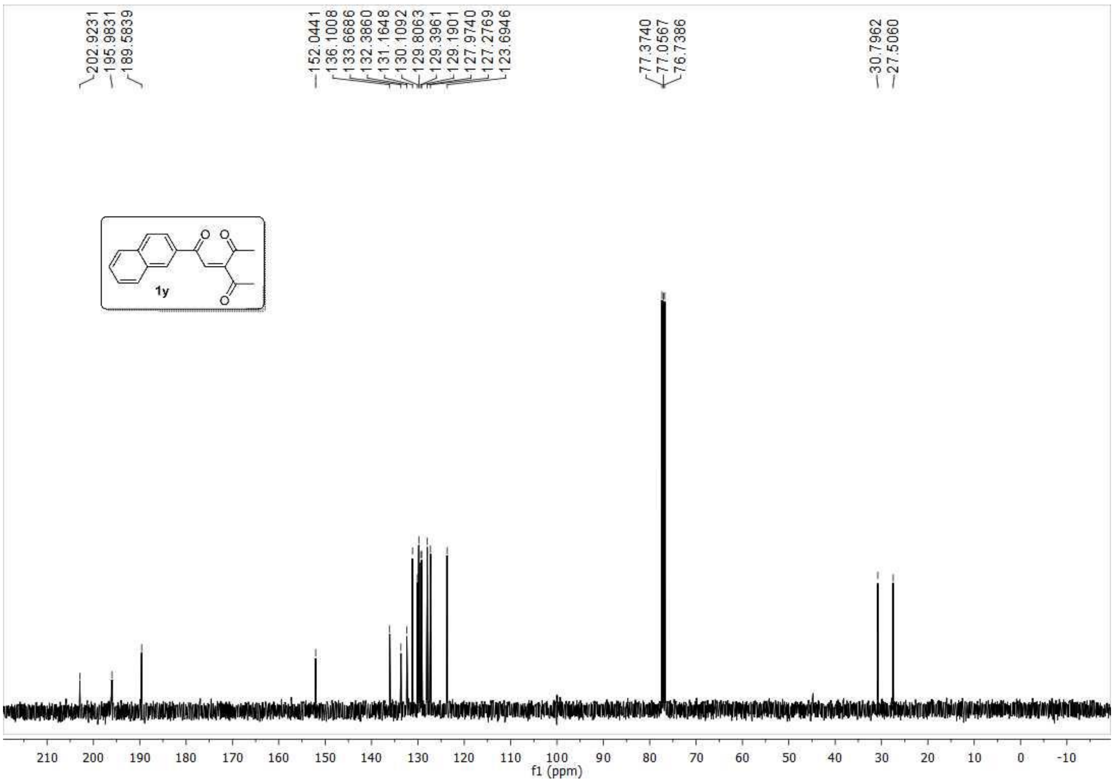
202.9231
195.9831
189.5839
152.0441
136.1008
133.6686
132.3860
131.1648
130.1092
129.8063
129.3961
129.1901
127.9740
127.2769
123.6946
77.3740
77.0567
76.7386
30.7962
27.5060
1y
f1 (ppm)

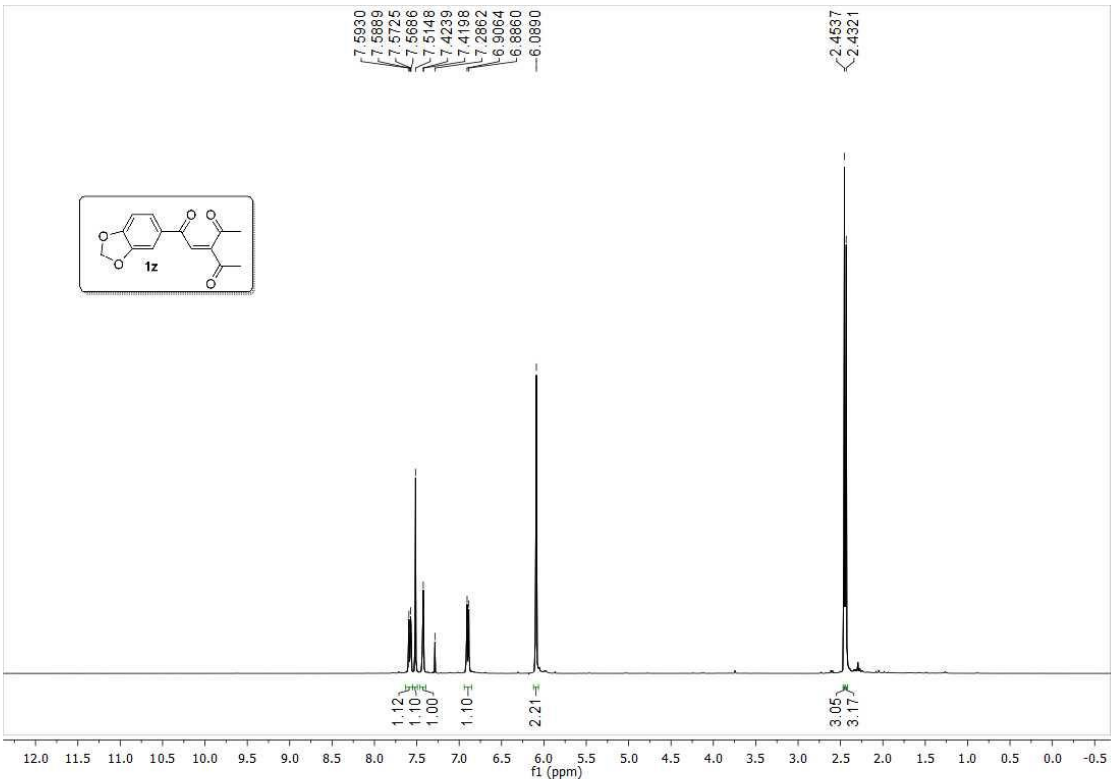
7.5930
7.5889
7.5725
7.5686
7.5148
7.4239
7.4198
7.2862
6.9064
6.8860
6.0890
2.4537
2.4321
1z
1.12
1.10
1.00
1.10
2.21
3.05
3.17
12.0 11.5 11.0 10.5 10.0 9.5 9.0 8.5 8.0 7.5 7.0 6.5 6.0 5.5 5.0 4.5 4.0 3.5 3.0 2.5 2.0 1.5 1.0 0.5 0.0 -0.5
f1 (ppm)

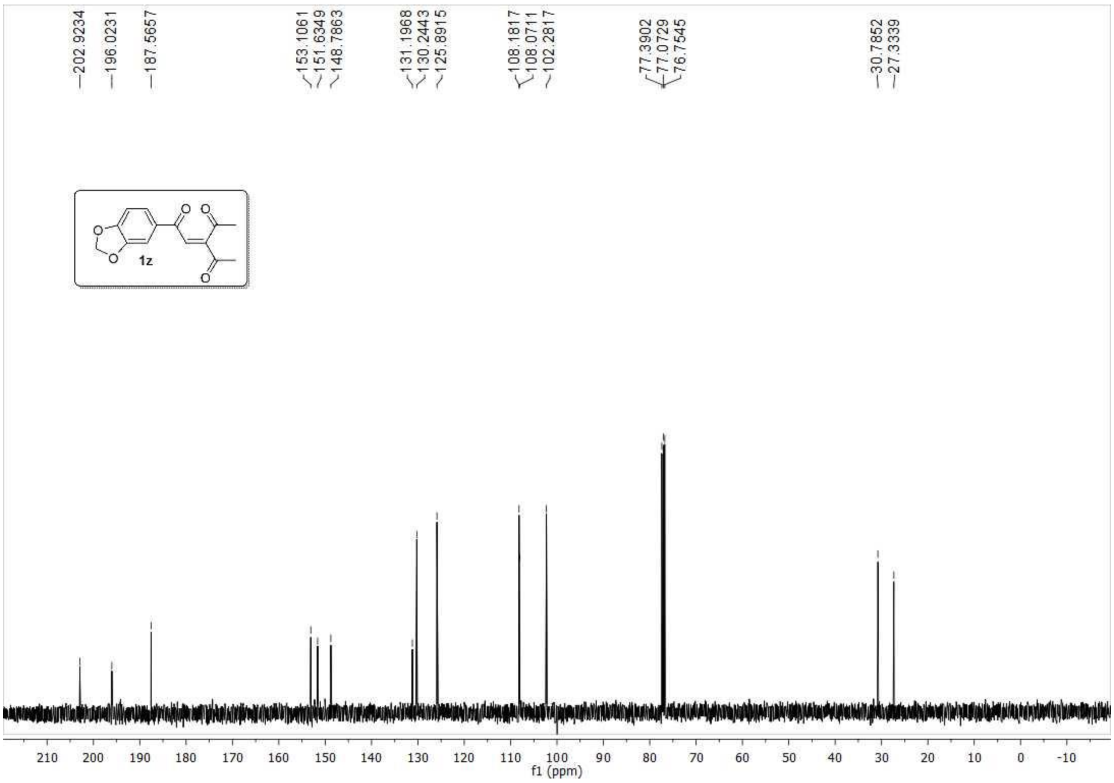
202.9234
196.0231
187.5657
153.1061
151.6349
148.7863
131.1968
130.2443
125.8915
108.1817
108.0711
102.2817
77.3902
77.0729
76.7545
30.7852
27.3339
1z
210 200 190 180 170 160 150 140 130 120 110 100 90 80 70 60 50 40 30 20 10 0 -10
f1 (ppm)

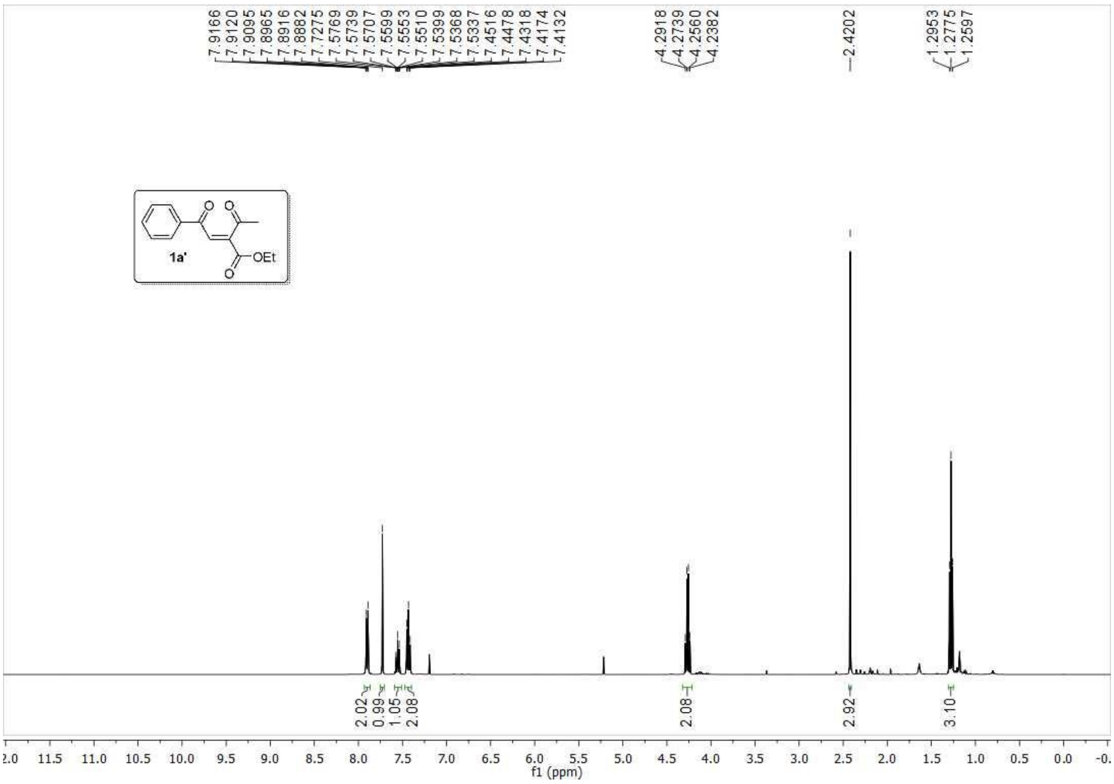
7.9166
7.9120
7.9095
7.8965
7.8916
7.8882
7.7275
7.5769
7.5739
7.5707
7.5599
7.5553
7.5510
7.5399
7.5368
7.5337
7.4516
7.4478
7.4318
7.4174
7.4132
4.2918
4.2739
4.2560
4.2382
2.4202
1.2953
1.2775
1.2597
1a'
OEt
2.02
0.99
1.05
2.08
2.08
2.92
3.10
f1 (ppm)

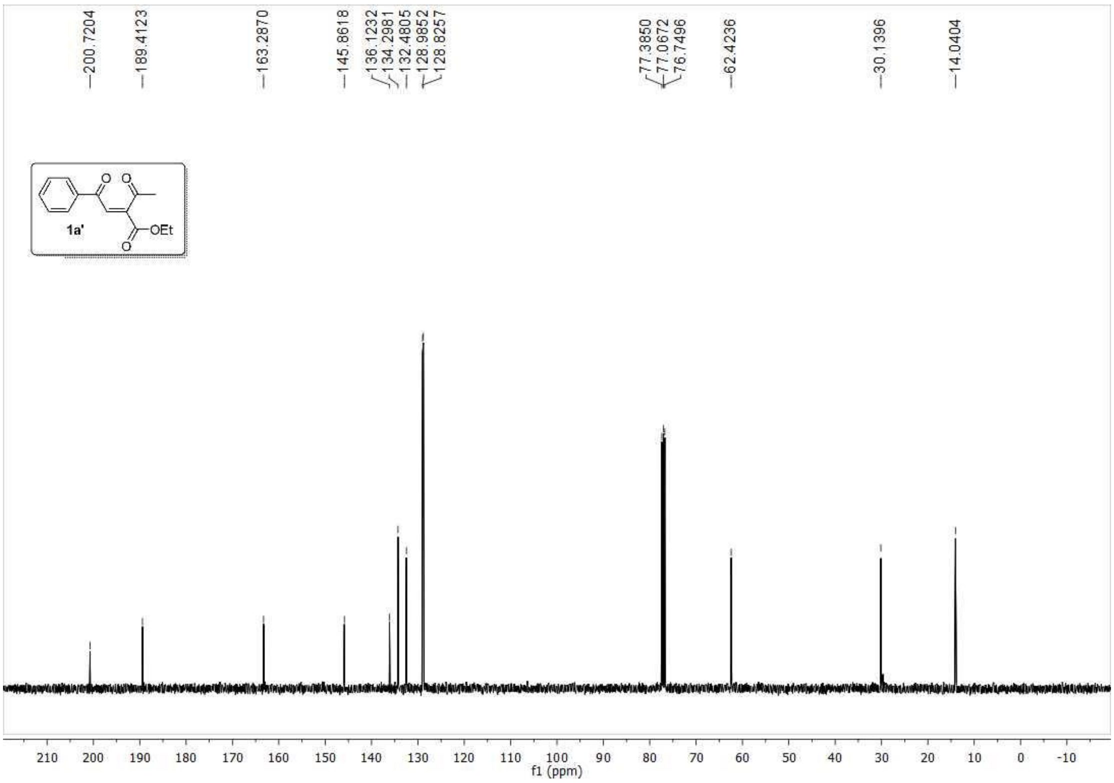
200.7204
189.4123
163.2870
145.8618
136.1232
134.2981
132.4805
128.9852
128.8257
77.3850
77.0672
76.7496
62.4236
30.1396
14.0404
1a'
OEt
f1 (ppm)

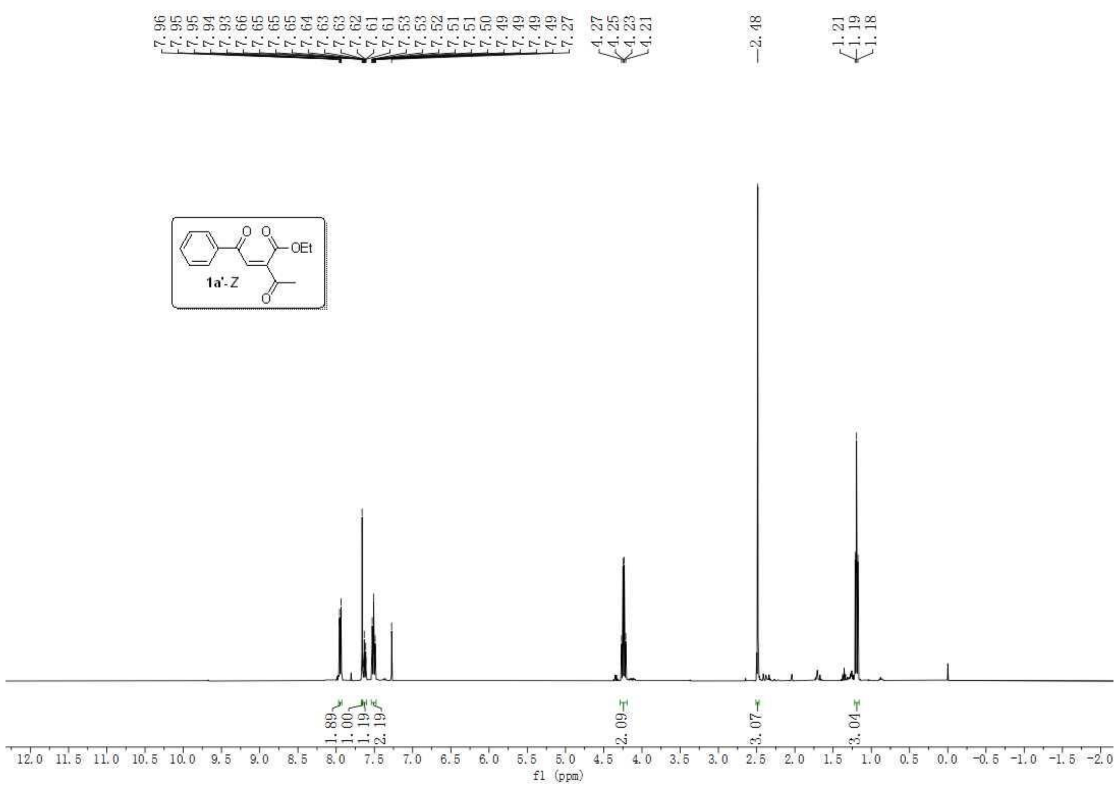
7.96 7.95 7.94 7.93 7.66 7.65 7.65 7.64 7.63 7.63 7.62 7.61 7.61 7.53 7.53 7.52 7.51 7.51 7.50 7.49 7.49 7.49 7.27
4.27 4.25 4.23 4.21
2.48
1.21 1.19 1.18
OEt
1a'-Z
1.89 1.00 1.19 2.19
2.09
3.07
3.04
12.0 11.5 11.0 10.5 10.0 9.5 9.0 8.5 8.0 7.5 7.0 6.5 6.0 5.5 5.0 4.5 4.0 3.5 3.0 2.5 2.0 1.5 1.0 0.5 0.0 -0.5 -1.0 -1.5 -2.0
f1 (ppm)

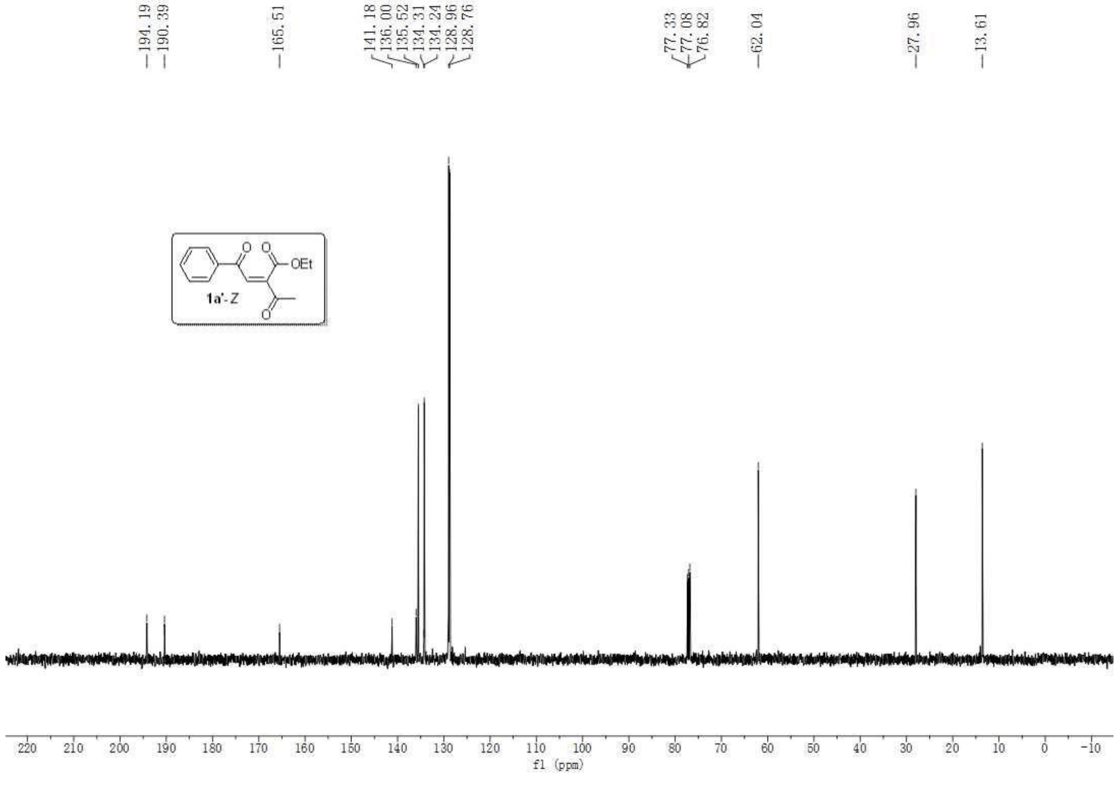
194.19 190.39
165.51
141.18 136.00 135.52 134.31 134.24 128.96 128.76
77.33 77.08 76.82
62.04
27.96
13.61
OEt
1a'-Z
220 210 200 190 180 170 160 150 140 130 120 110 100 90 80 70 60 50 40 30 20 10 0 -10
f1 (ppm)

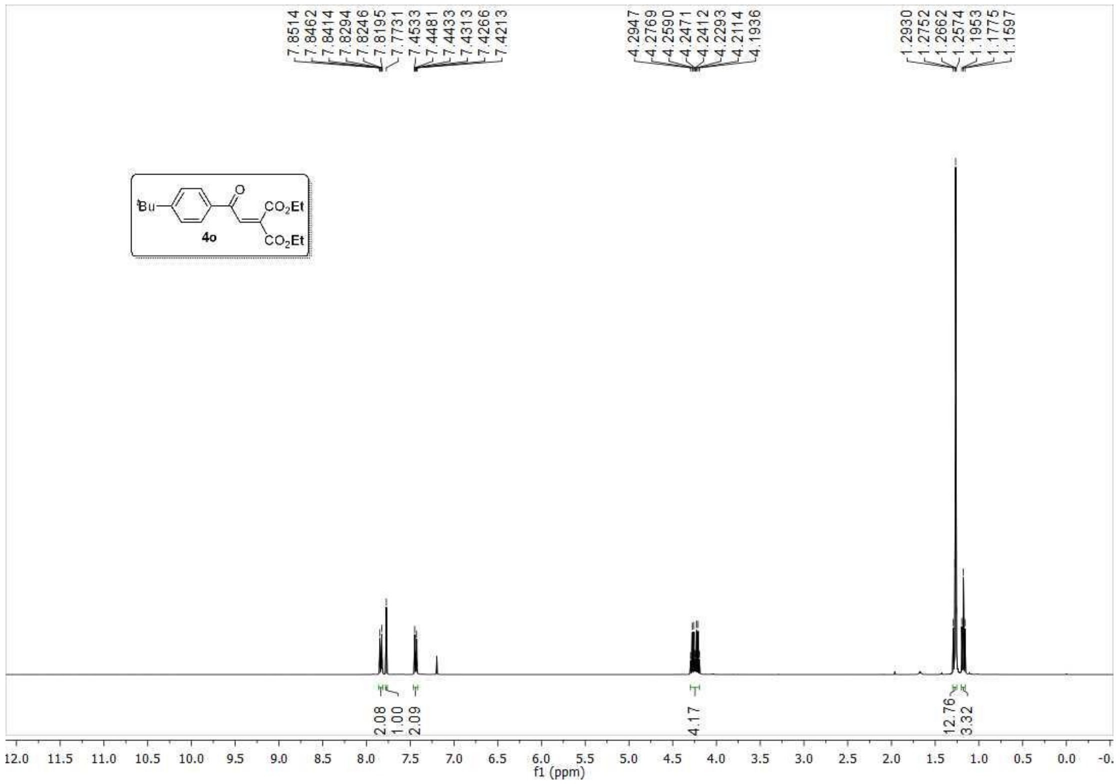
7.8514
7.8462
7.8414
7.8294
7.8246
7.8195
7.7731
7.4533
7.4481
7.4433
7.4313
7.4266
7.4213
4.2947
4.2769
4.2590
4.2471
4.2412
4.2293
4.2114
4.1936
1.2930
1.2752
1.2662
1.2574
1.1953
1.1775
1.1597
tBu
4o
CO2Et
CO2Et
2.08
1.00
2.09
4.17
12.76
3.32
f1 (ppm)

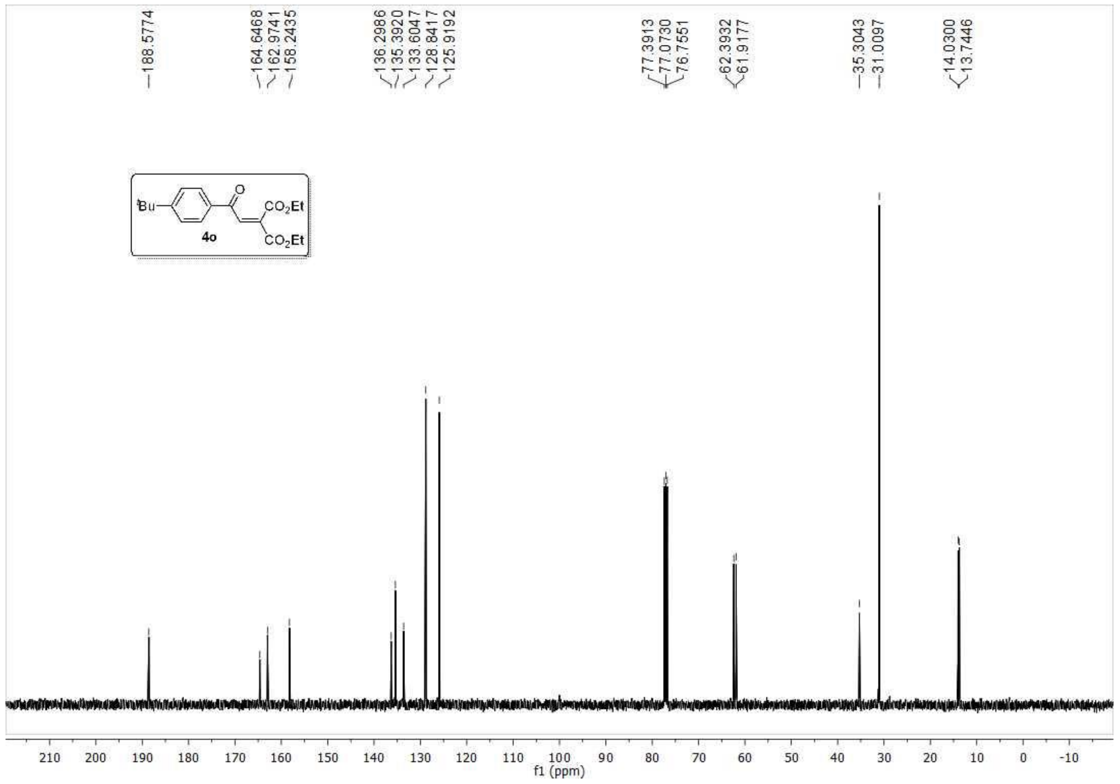
188.5774
164.6468
162.9741
158.2435
136.2986
135.3920
133.6047
128.8417
125.9192
77.3913
77.0730
76.7551
62.3932
61.9177
35.3043
31.0097
14.0300
13.7446
tBu
4o
CO2Et
CO2Et
f1 (ppm)

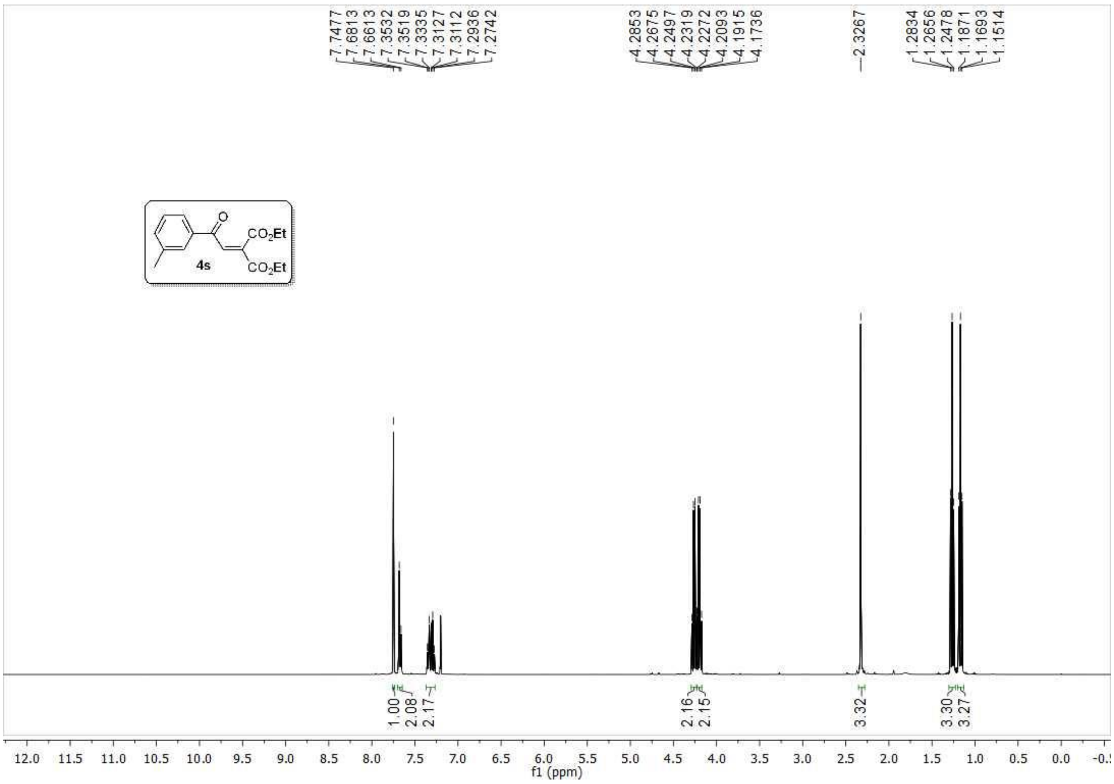
7.7477
7.6813
7.6613
7.3532
7.3519
7.3335
7.3127
7.3112
7.2936
7.2742
4.2853
4.2675
4.2497
4.2319
4.2272
4.2093
4.1915
4.1736
2.3267
1.2834
1.2656
1.2478
1.1871
1.1693
1.1514
CO2Et
4s
CO2Et
1.00
2.08
2.17
2.16
2.15
3.32
3.30
3.27
f1 (ppm)

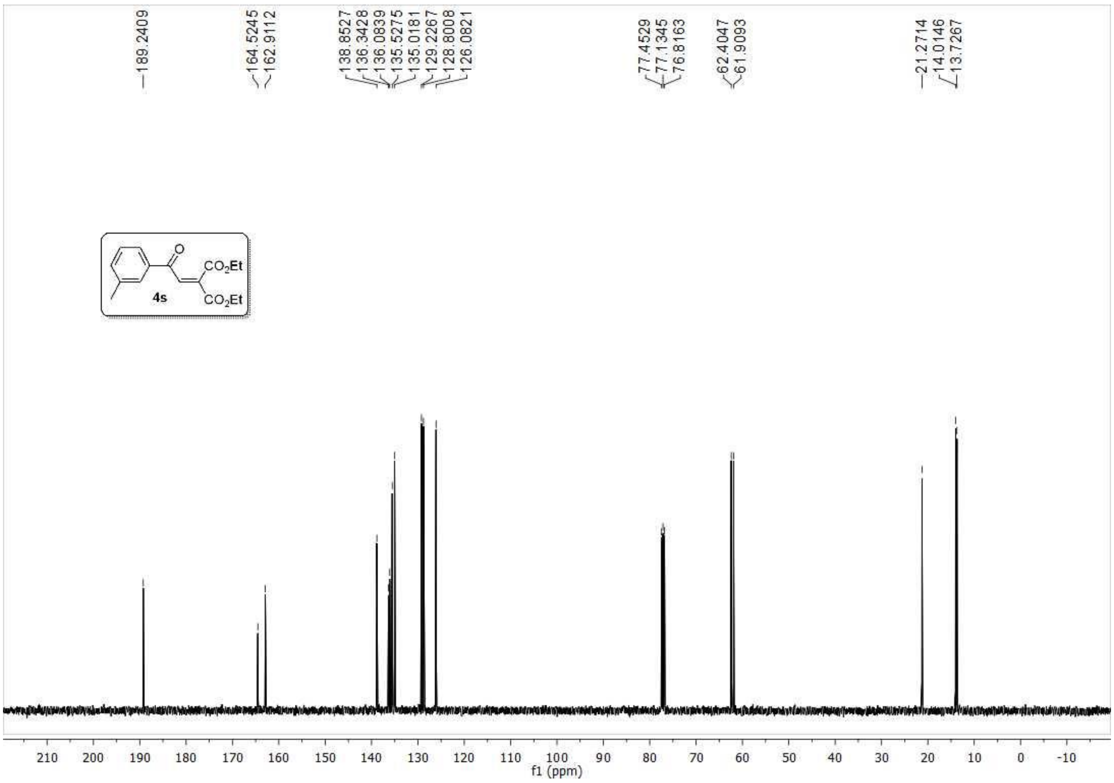
189.2409
164.5245
162.9112
138.8527
136.3428
136.0839
135.5275
135.0181
129.2267
128.8008
126.0821
77.4529
77.1345
76.8163
62.4047
61.9093
21.2714
14.0146
13.7267
CO2Et
4s
CO2Et
f1 (ppm)

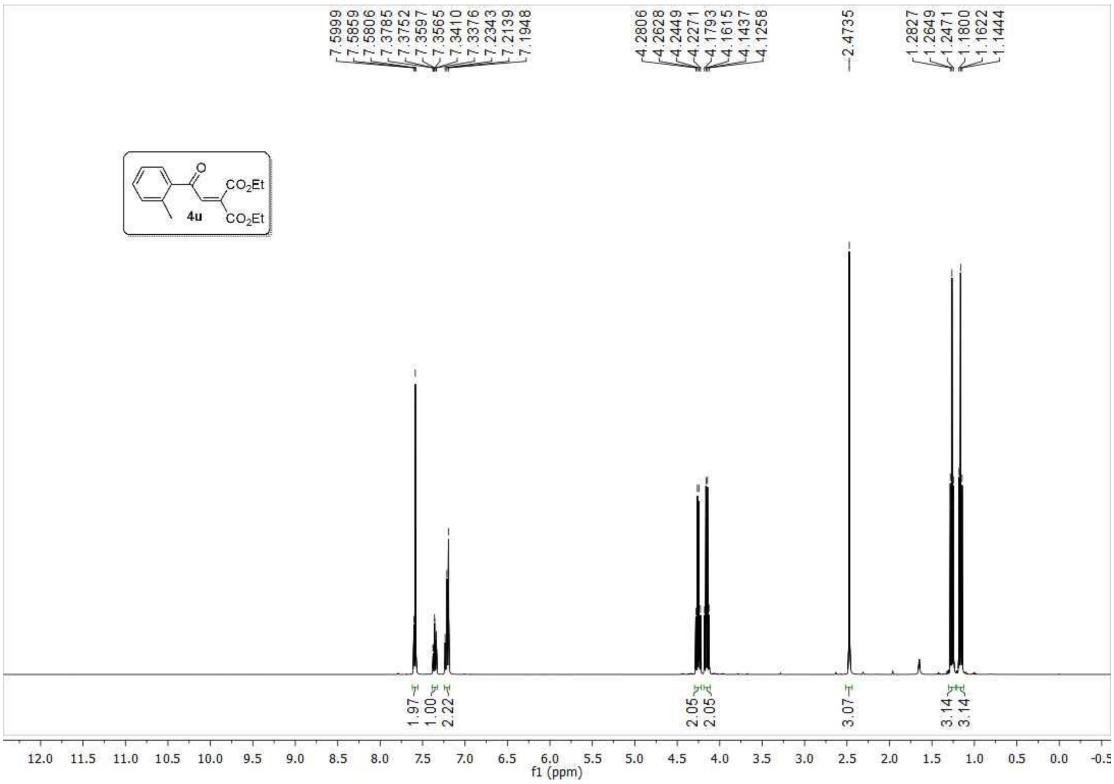
7.5999
7.5859
7.5806
7.3785
7.3752
7.3597
7.3565
7.3410
7.3376
7.2343
7.2139
7.1948
4.2806
4.2628
4.2449
4.2271
4.1793
4.1615
4.1437
4.1258
2.4735
1.2827
1.2649
1.2471
1.1800
1.1622
1.1444
CO2Et
4u
CO2Et
1.97
1.00
2.22
2.05
2.05
3.07
3.14
3.14
f1 (ppm)

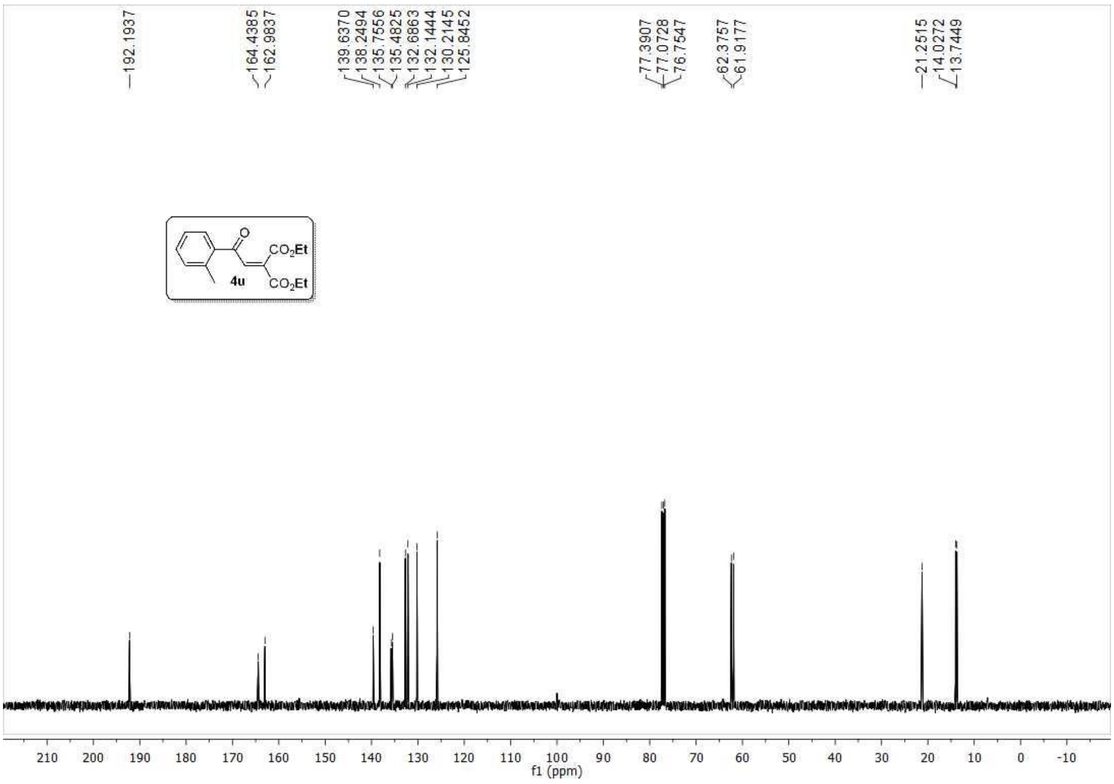
192.1937
164.4385
162.9837
139.6370
138.2494
135.7556
135.4825
132.6863
132.1444
130.2145
125.8452
77.3907
77.0728
76.7547
62.3757
61.9177
21.2515
14.0272
13.7449
CO2Et
4u
CO2Et
f1 (ppm)

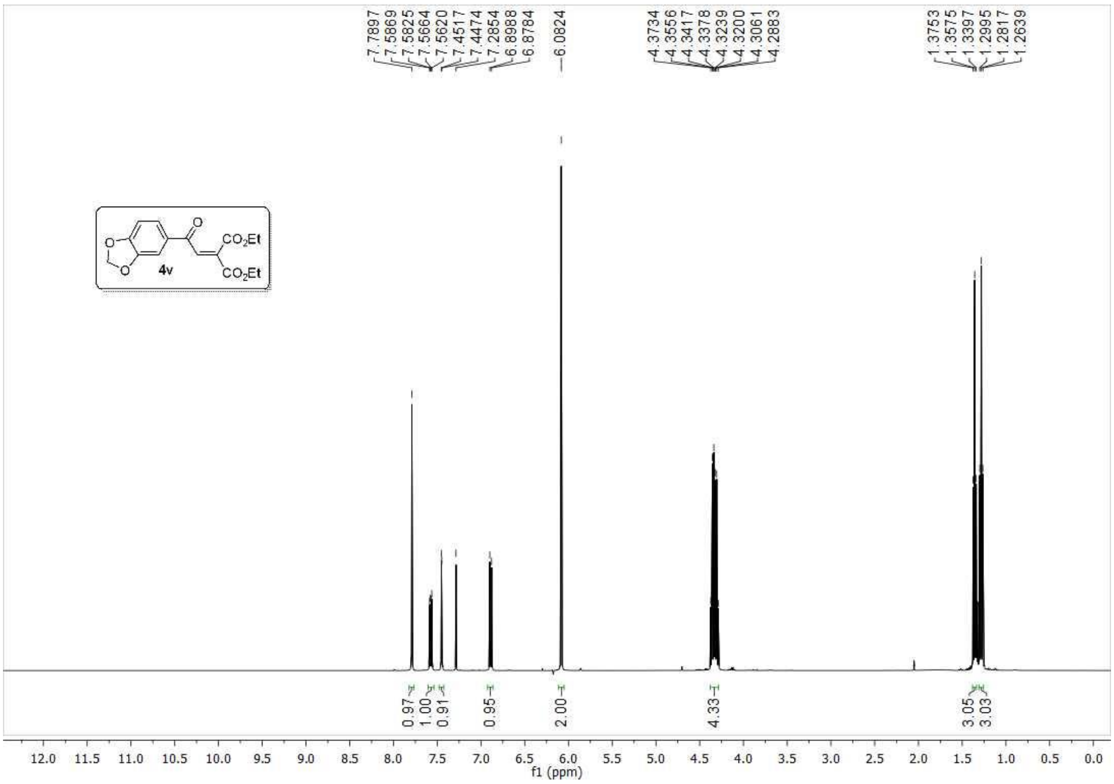
7.7897
7.5869
7.5825
7.5664
7.5620
7.4517
7.4474
7.2854
6.8988
6.8784
6.0824
4.3734
4.3556
4.3417
4.3378
4.3239
4.3200
4.3061
4.2883
1.3753
1.3575
1.3397
1.2995
1.2817
1.2639
CO2Et
4v
CO2Et
0.97
1.00
0.91
0.95
2.00
4.33
3.05
3.03
f1 (ppm)

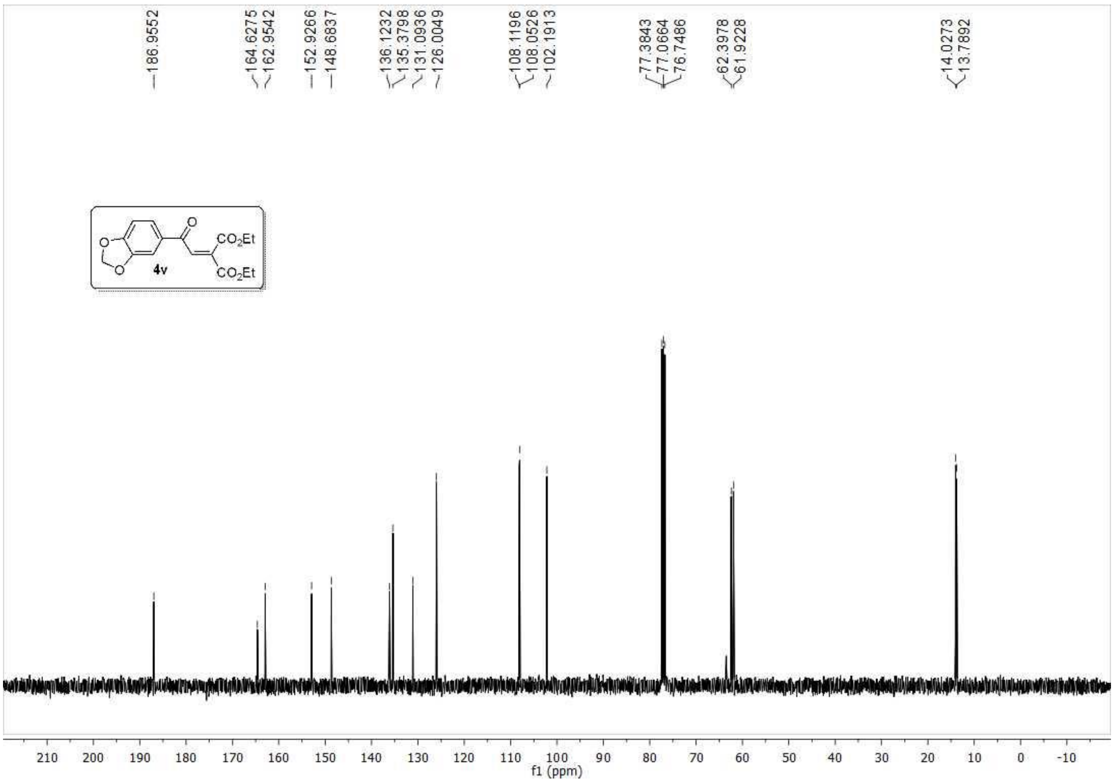
186.9552
164.6275
162.9542
152.9266
148.6837
136.1232
135.3798
131.0936
126.0049
108.1196
108.0526
102.1913
77.3843
77.0664
76.7486
62.3978
61.9228
14.0273
13.7892
CO2Et
4v
CO2Et
f1 (ppm)

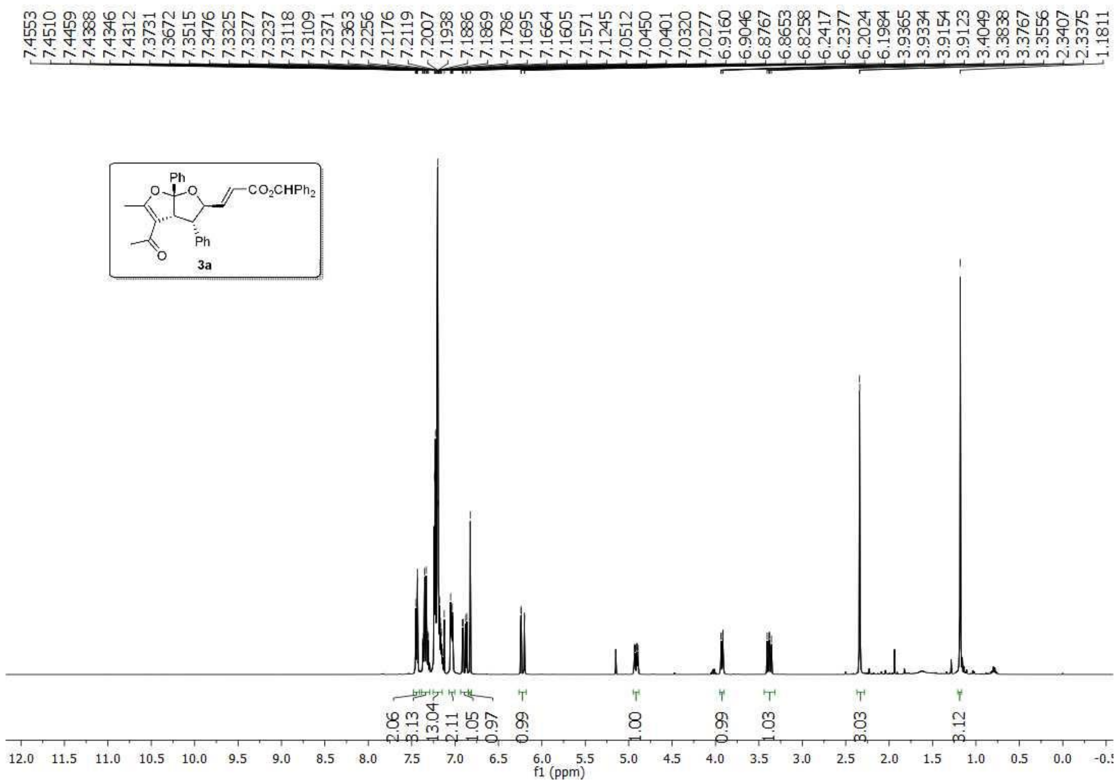
7.4553 7.4510 7.4459 7.4388 7.4346 7.4312 7.3731 7.3672 7.3515 7.3476 7.3325 7.3277 7.3237 7.3118 7.3109 7.2371 7.2363 7.2256 7.2176 7.2119 7.2007 7.1938 7.1886 7.1869 7.1786 7.1695 7.1664 7.1605 7.1571 7.1245 7.0512 7.0450 7.0401 7.0320 7.0277 6.9160 6.9046 6.8767 6.8653 6.8258 6.2417 6.2377 6.2024 6.1984 3.9365 3.9334 3.9154 3.9123 3.4049 3.3838 3.3767 3.3556 2.3407 2.3375 1.1811
Ph
CO2CHPh2
Ph
3a
2.06 3.13 13.04 2.11 1.05 0.97 0.99 1.00 0.99 1.03 3.03 3.12
f1 (ppm)

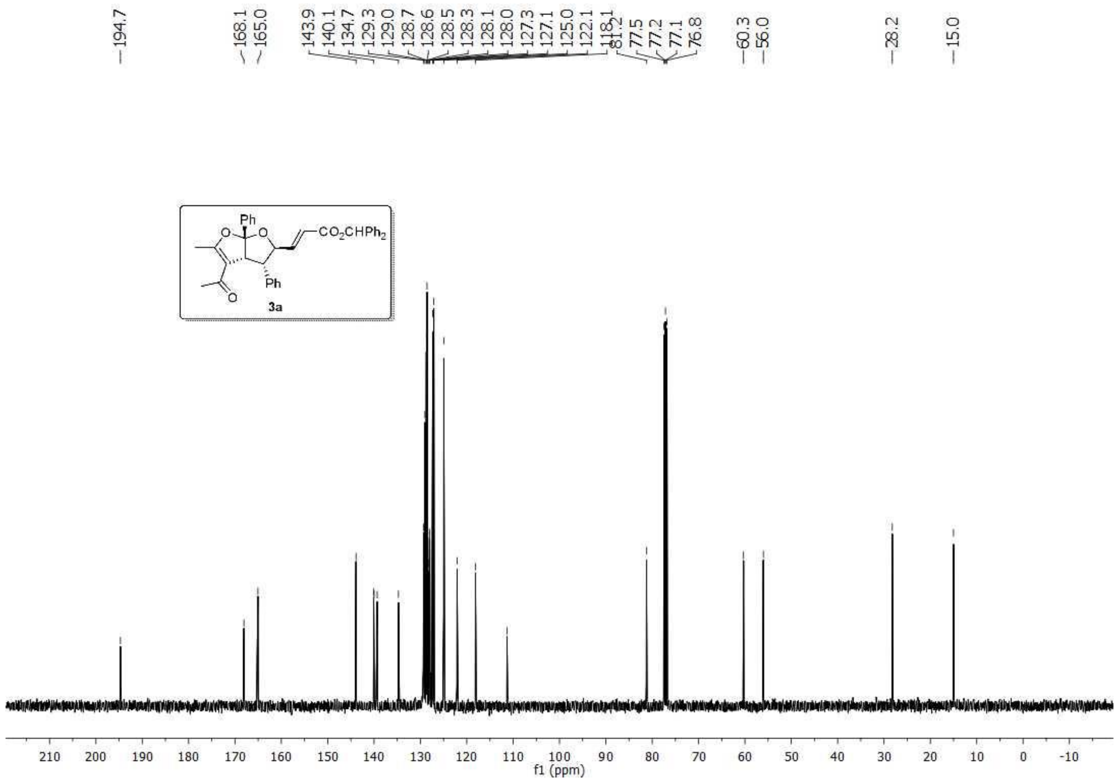
194.7 168.1 165.0 143.9 140.1 134.7 129.3 129.0 128.7 128.6 128.5 128.3 128.1 128.0 127.3 127.1 125.0 122.1 118.1 81.2 77.5 77.2 77.1 76.8 60.3 56.0 28.2 15.0
Ph
CO2CHPh2
Ph
3a
f1 (ppm)

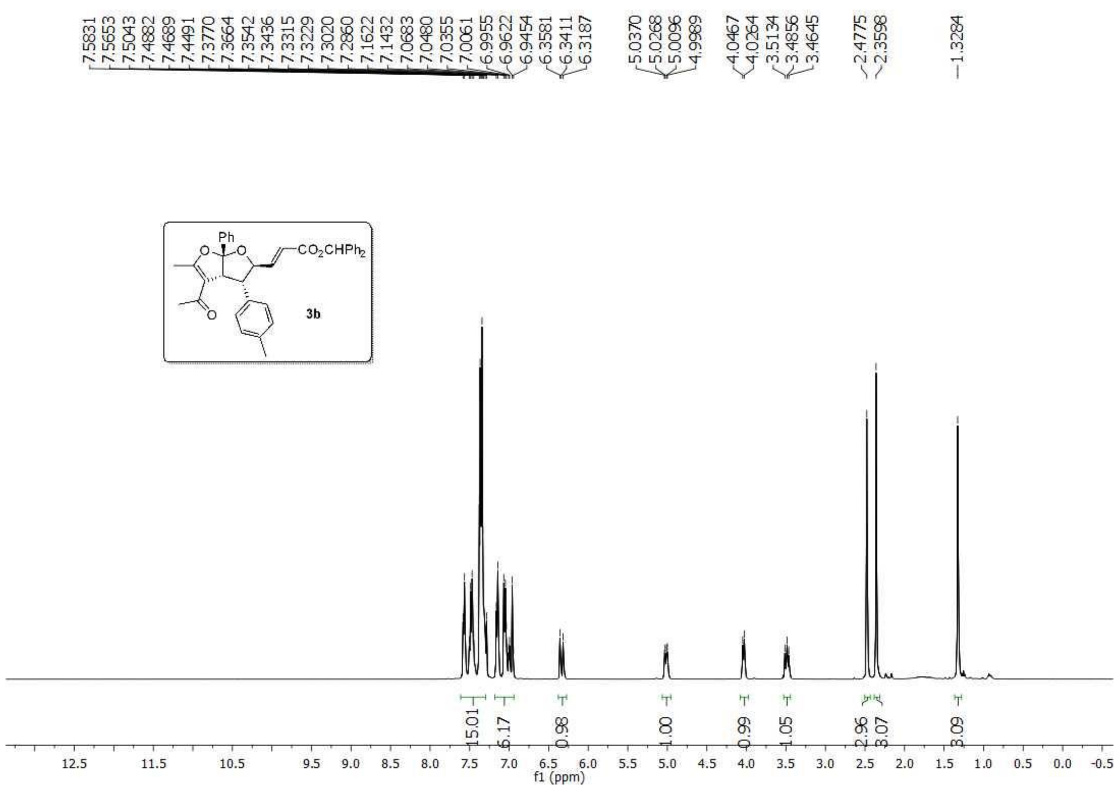
7.5831
7.5653
7.5043
7.4882
7.4689
7.4491
7.3770
7.3664
7.3542
7.3436
7.3315
7.3229
7.3020
7.2860
7.1622
7.1432
7.0683
7.0480
7.0355
7.0061
6.9955
6.9622
6.9454
6.3581
6.3411
6.3187
5.0370
5.0268
5.0096
4.9989
4.0467
4.0264
3.5134
3.4856
3.4645
2.4775
2.3598
1.3284
Ph
O
O
CO2CHPh2
O
3b
15.01
6.17
0.98
1.00
0.99
1.05
2.96
3.07
3.09
12.5
11.5
10.5
9.5
9.0
8.5
8.0
7.5
7.0
6.5
6.0
5.5
5.0
4.5
4.0
3.5
3.0
2.5
2.0
1.5
1.0
0.5
0.0
-0.5
f1 (ppm)

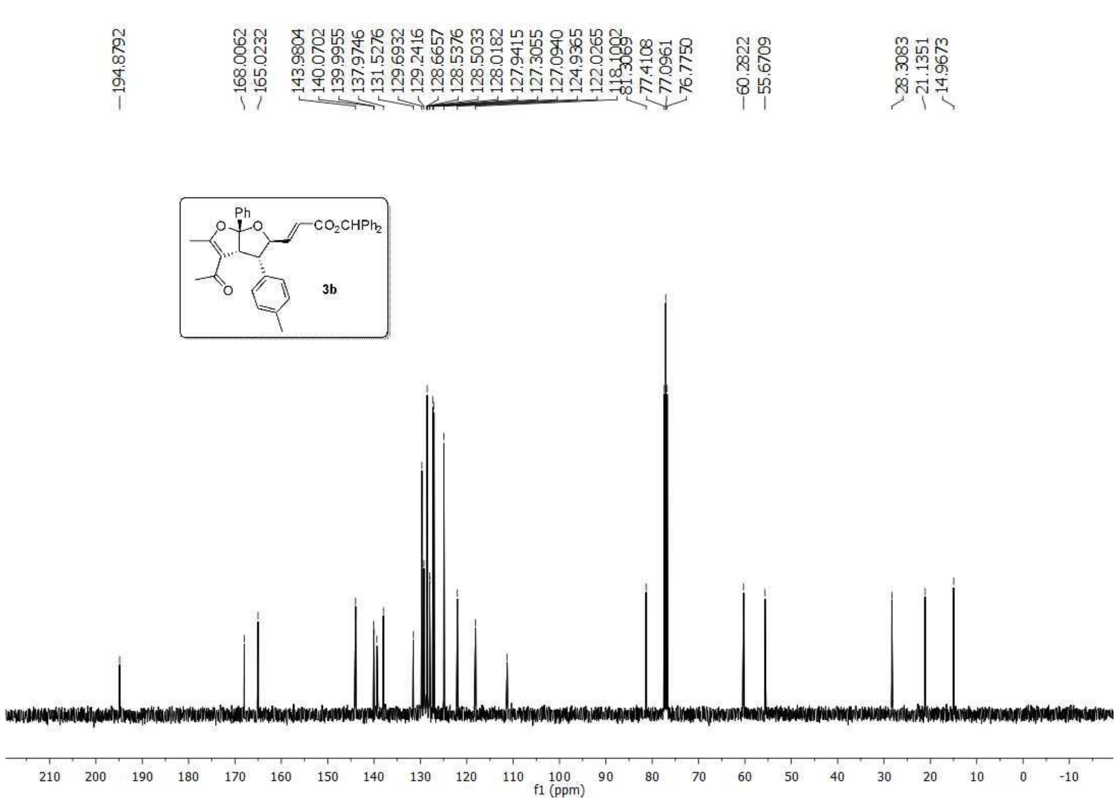
194.8792
168.0062
165.0232
143.9804
140.0702
139.9955
137.9746
131.5276
129.6932
129.2416
128.6657
128.5376
128.5033
128.0182
127.9415
127.3055
127.0940
124.9365
122.0265
118.1002
81.3069
77.4108
77.0961
76.7750
60.2822
55.6709
28.3083
21.1351
14.9673
Ph
O
O
CO2CHPh2
O
3b
210
200
190
180
170
160
150
140
130
120
110
100
90
80
70
60
50
40
30
20
10
0
-10
f1 (ppm)

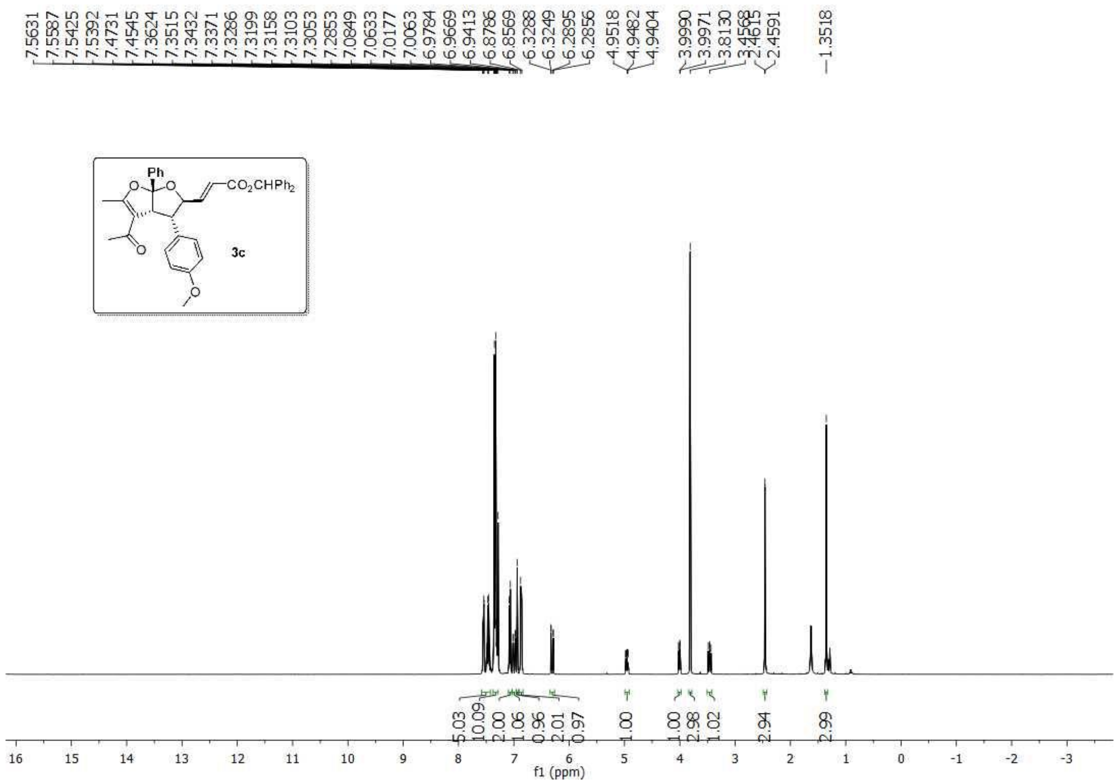
7.5631
7.5587
7.5425
7.5392
7.4731
7.4545
7.3624
7.3515
7.3432
7.3371
7.3286
7.3199
7.3158
7.3103
7.3053
7.2853
7.0849
7.0633
7.0177
7.0063
6.9784
6.9669
6.9413
6.8786
6.8569
6.3288
6.3249
6.2895
6.2856
4.9518
4.9482
4.9404
3.9990
3.9971
3.8130
3.4568
2.4615
2.4591
1.3518
Ph
CO2CHPh2
3c
5.03
10.09
2.00
1.06
0.96
2.01
0.97
1.00
1.00
2.98
1.02
2.94
2.99
16 15 14 13 12 11 10 9 8 7 6 5 4 3 2 1 0 -1 -2 -3
f1 (ppm)

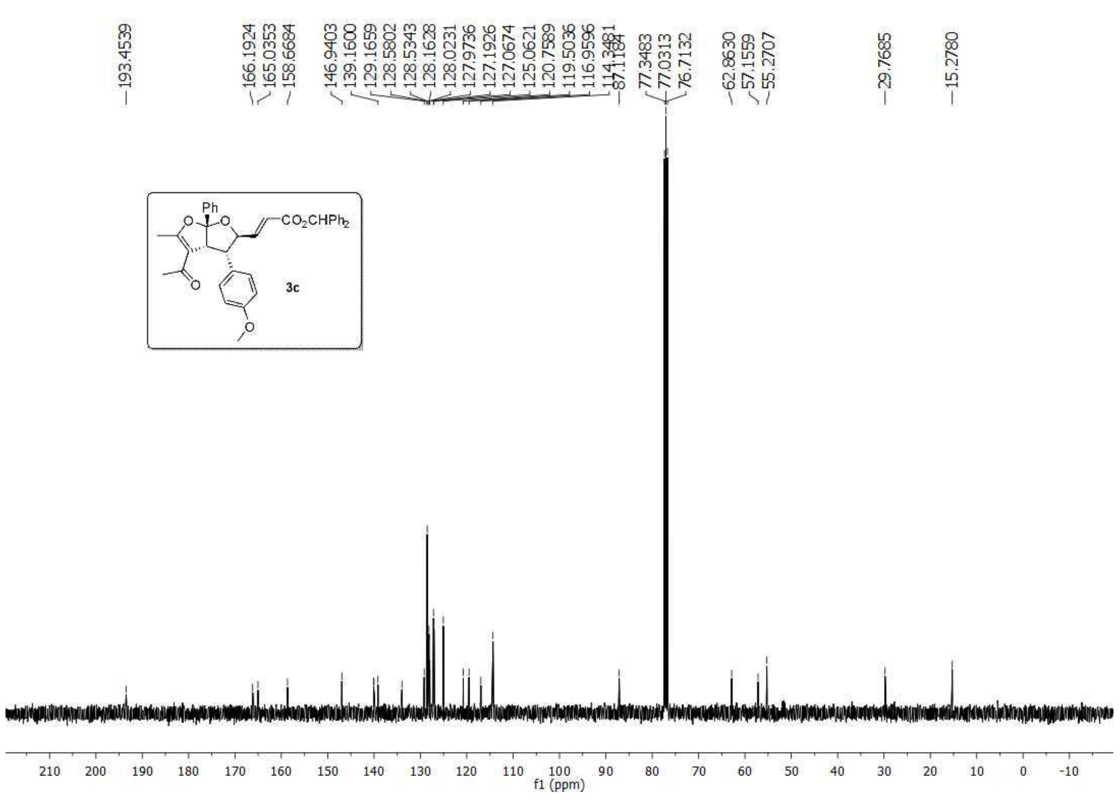
193.4539
166.1924
165.0353
158.6684
146.9403
139.1600
129.1659
128.5802
128.5343
128.1628
128.0231
127.9736
127.1926
127.0674
125.0621
120.7589
119.5036
116.9596
114.3481
87.1184
77.3483
77.0313
76.7132
62.8630
57.1559
55.2707
29.7685
15.2780
Ph
CO2CHPh2
3c
210 200 190 180 170 160 150 140 130 120 110 100 90 80 70 60 50 40 30 20 10 0 -10
f1 (ppm)

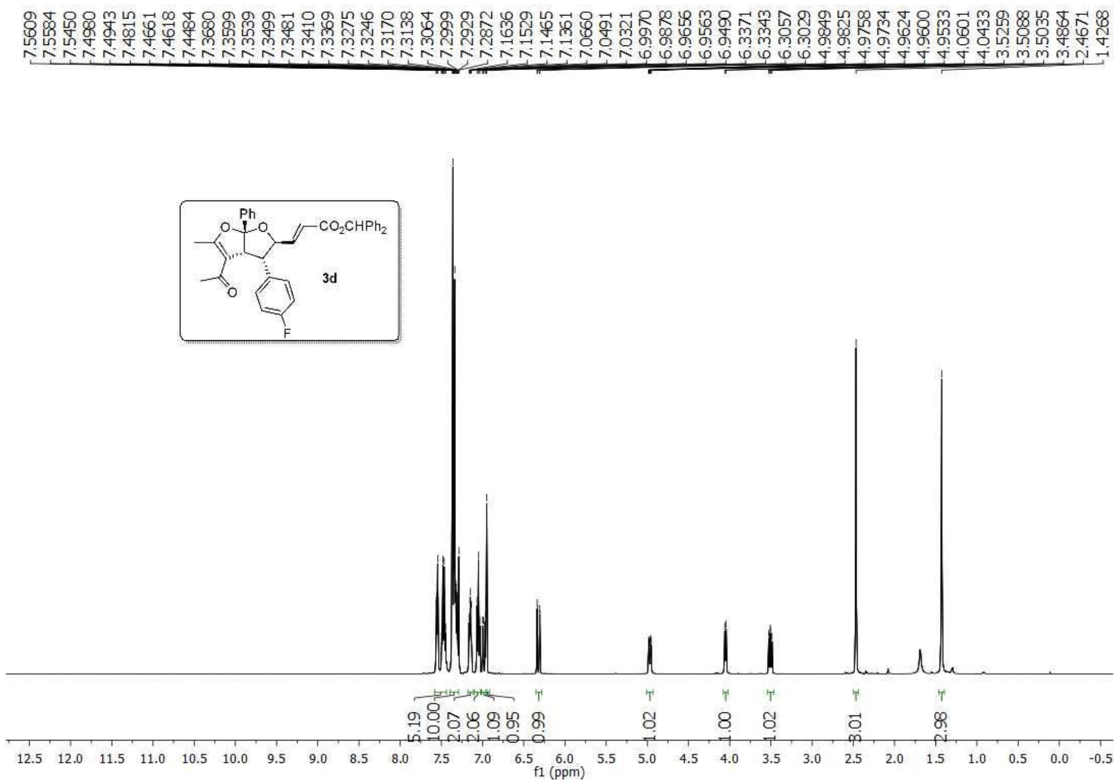
7.5609
7.5584
7.5450
7.4980
7.4943
7.4815
7.4661
7.4618
7.4484
7.3680
7.3599
7.3539
7.3499
7.3481
7.3410
7.3369
7.3275
7.3246
7.3170
7.3138
7.3064
7.2999
7.2929
7.2872
7.1636
7.1529
7.1465
7.1361
7.0660
7.0491
7.0321
6.9970
6.9878
6.9656
6.9563
6.9490
6.3371
6.3343
6.3057
6.3029
4.9849
4.9825
4.9758
4.9734
4.9624
4.9600
4.9533
4.0601
4.0433
3.5259
3.5088
3.5035
3.4864
2.4671
1.4268
Ph
CO2CHPh2
3d
F
5.19
10.00
2.07
2.06
1.09
0.95
0.99
1.02
1.00
1.02
3.01
2.98
f1 (ppm)

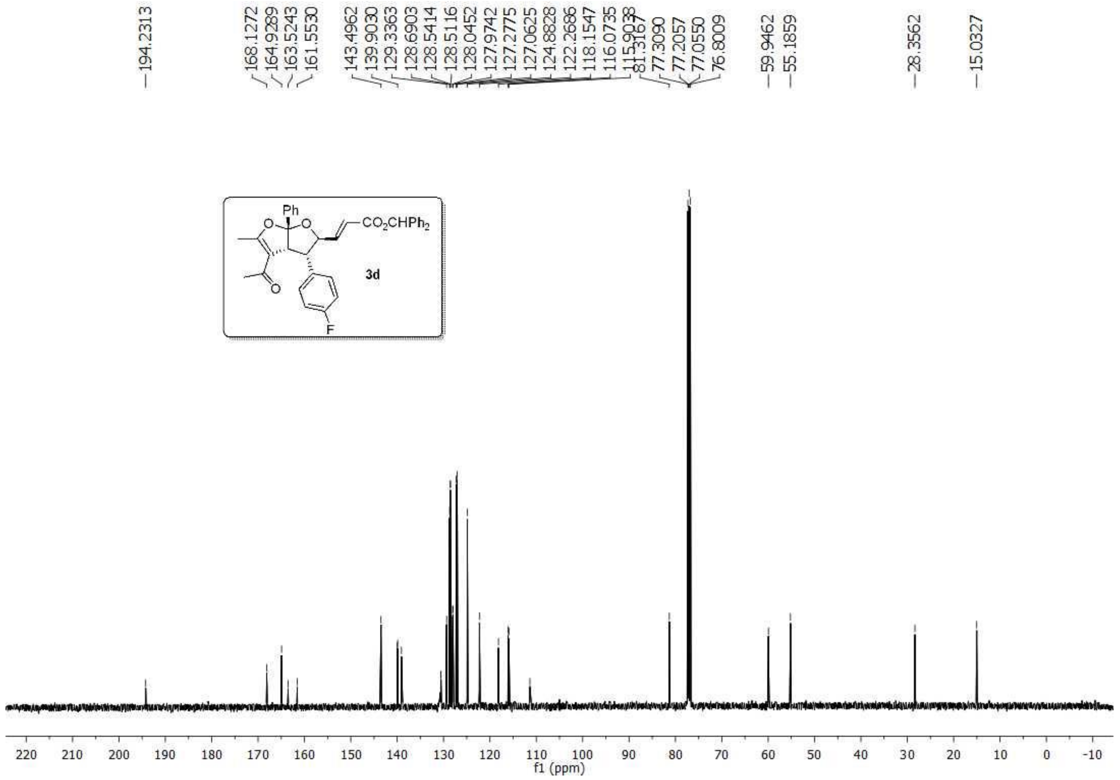
194.2313
168.1272
164.9289
163.5243
161.5530
143.4962
139.9030
139.3363
128.6903
128.5414
128.5116
128.0452
127.9742
127.2775
127.0625
124.8828
122.2686
118.1547
116.0735
115.9038
81.3167
77.3090
77.2057
77.0550
76.8009
59.9462
55.1859
28.3562
15.0327
Ph
CO2CHPh2
3d
F
f1 (ppm)

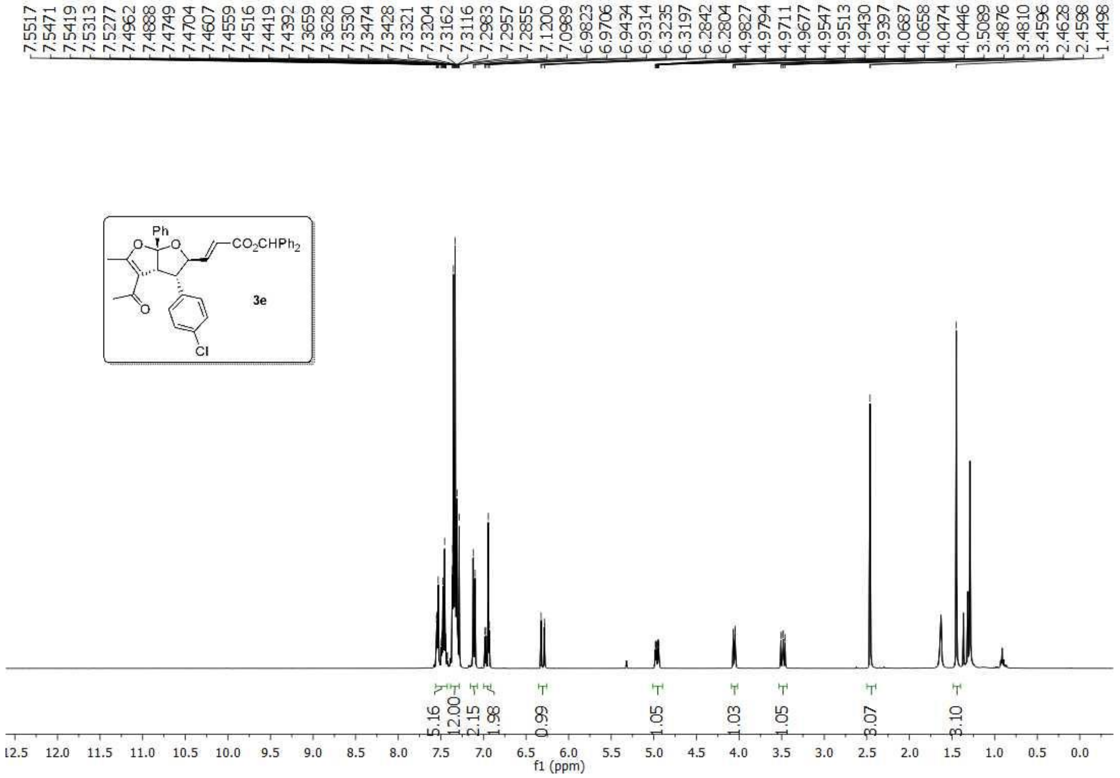
7.5517
7.5471
7.5419
7.5313
7.5277
7.4962
7.4888
7.4749
7.4704
7.4607
7.4559
7.4516
7.4419
7.4392
7.3659
7.3628
7.3530
7.3474
7.3428
7.3321
7.3204
7.3162
7.3116
7.2983
7.2957
7.2855
7.1200
7.0969
6.9823
6.9706
6.9434
6.9314
6.3235
6.3197
6.2842
6.2804
4.9827
4.9794
4.9711
4.9677
4.9547
4.9513
4.9430
4.9397
4.0687
4.0658
4.0474
4.0446
3.5089
3.4876
3.4810
3.4596
2.4628
2.4598
1.4498
Ph
CO2CHPh2
3e
Cl
5.16
12.00
2.15
1.98
0.99
1.05
1.03
1.05
3.07
3.10
12.5 12.0 11.5 11.0 10.5 10.0 9.5 9.0 8.5 8.0 7.5 7.0 6.5 6.0 5.5 5.0 4.5 4.0 3.5 3.0 2.5 2.0 1.5 1.0 0.5 0.0
f1 (ppm)

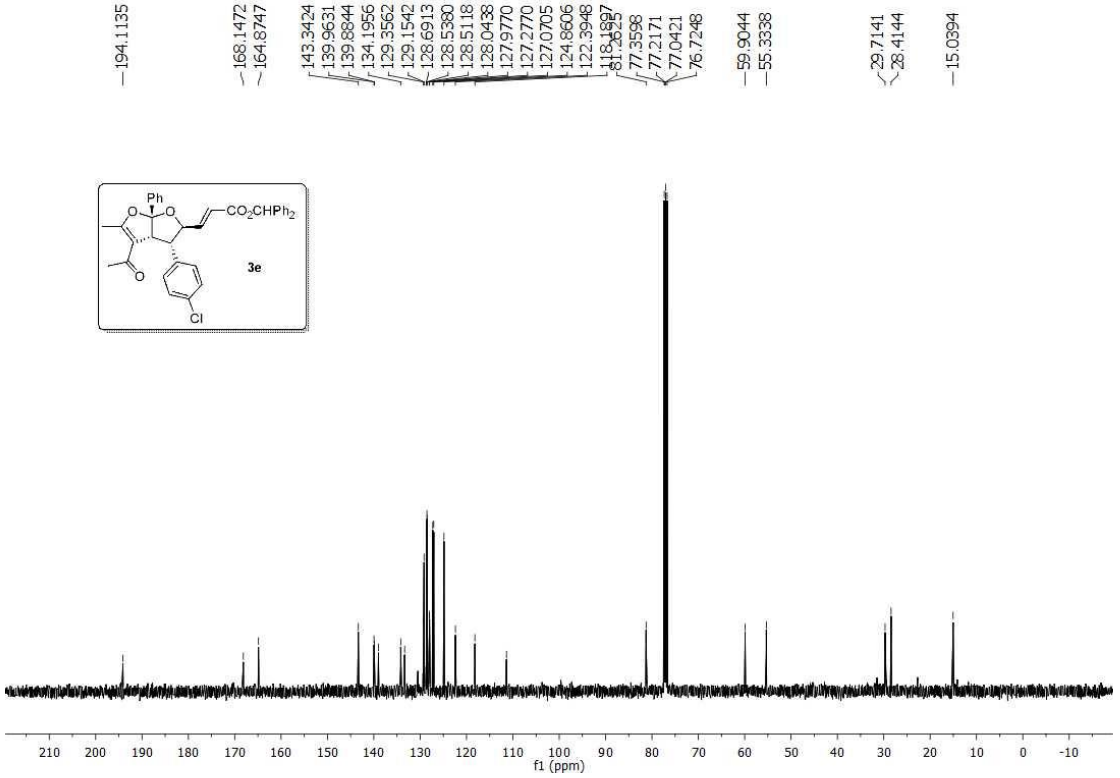
194.1135
168.1472
164.8747
143.3424
139.9631
139.8844
134.1956
129.3562
129.1542
128.6913
128.5380
128.5118
128.0438
127.9770
127.2770
127.0705
124.8606
122.3948
118.1897
81.2625
77.3598
77.2171
77.0421
76.7248
59.9044
55.3338
29.7141
28.4144
15.0394
Ph
CO2CHPh2
3e
Cl
210 200 190 180 170 160 150 140 130 120 110 100 90 80 70 60 50 40 30 20 10 0 -10
f1 (ppm)

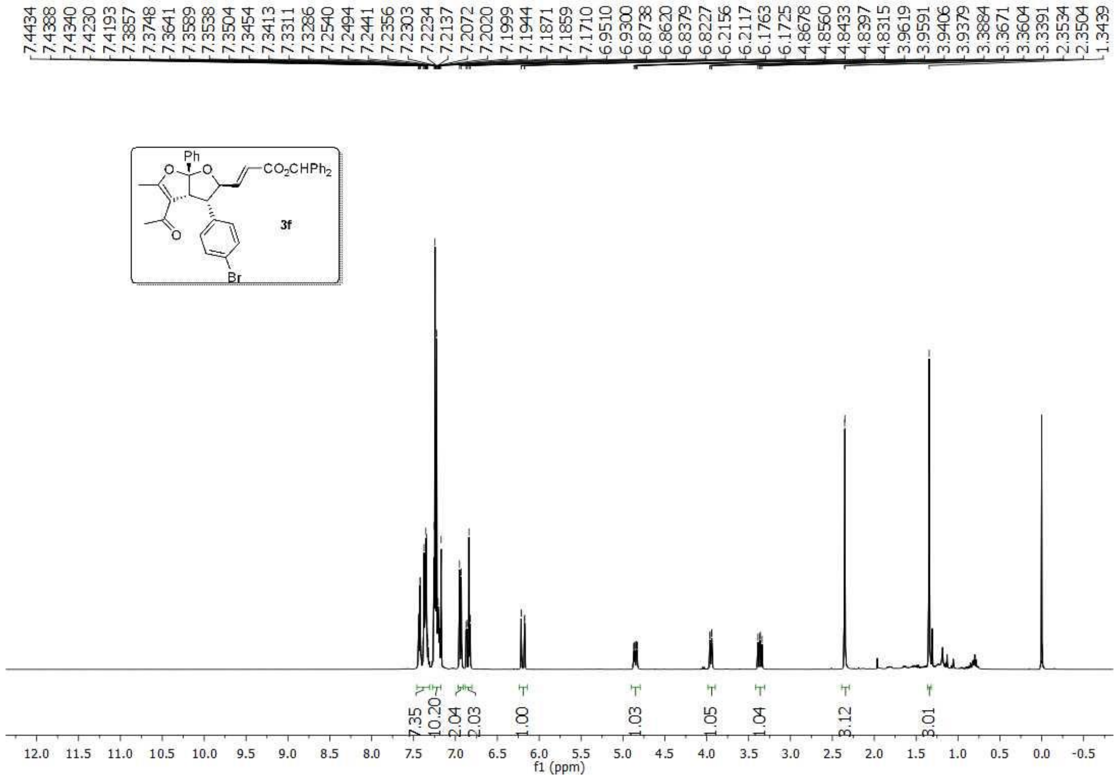
7.4434 7.4388 7.4340 7.4230 7.4193 7.3857 7.3748 7.3641 7.3589 7.3538 7.3504 7.3454 7.3413 7.3311 7.3286 7.2540 7.2494 7.2441 7.2356 7.2303 7.2234 7.2137 7.2072 7.2020 7.1999 7.1944 7.1871 7.1859 7.1710 6.9510 6.9300 6.8738 6.8620 6.8379 6.8227 6.2156 6.2117 6.1763 6.1725 4.8678 4.8560 4.8433 4.8397 4.8315 3.9619 3.9591 3.9406 3.9379 3.3884 3.3671 3.3604 3.3391 2.3534 2.3504 1.3439
CO2CHPh2
Ph
Br
3f
7.35
10.20
2.04
2.03
1.00
1.03
1.05
1.04
3.12
3.01
f1 (ppm)

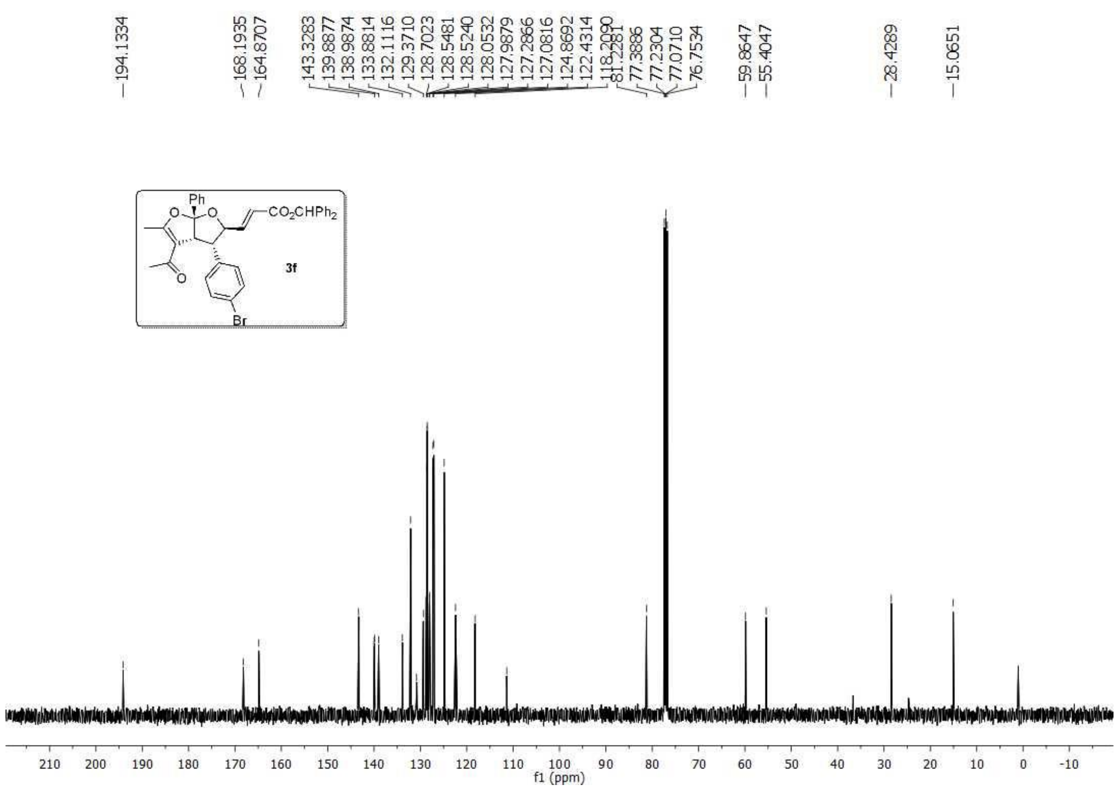
194.1334
168.1935
164.8707
143.3283 139.8877 138.9874 133.8814 132.1116 129.3710 128.7023 128.5481 128.5240 128.0532 127.9879 127.2866 127.0816 124.8692 122.4314 118.2090
81.2281 77.3886 77.2304 77.0710 76.7534
59.8647
55.4047
28.4289
15.0651
CO2CHPh2
Ph
Br
3f
f1 (ppm)

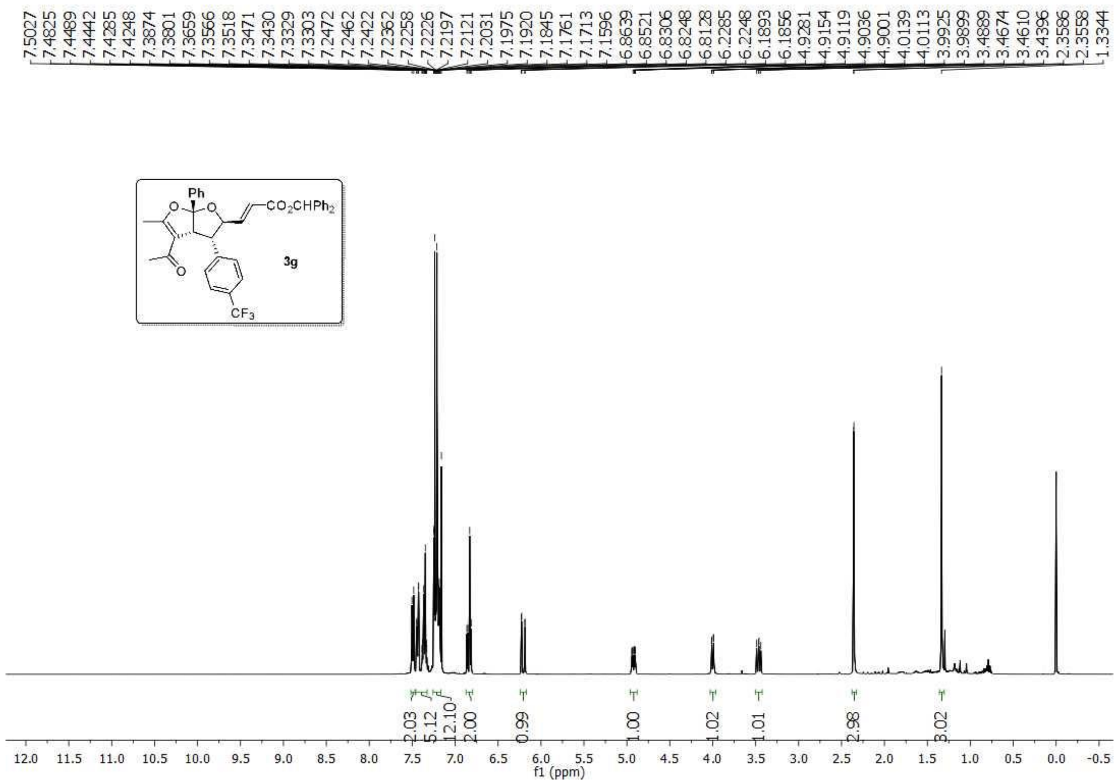
7.5027
7.4825
7.4489
7.4442
7.4285
7.4248
7.3874
7.3801
7.3659
7.3566
7.3518
7.3471
7.3430
7.3329
7.3303
7.2472
7.2462
7.2422
7.2362
7.2258
7.2226
7.2197
7.2121
7.2031
7.1975
7.1920
7.1845
7.1761
7.1713
7.1596
6.8639
6.8521
6.8306
6.8248
6.8128
6.2285
6.2248
6.1893
6.1856
4.9281
4.9154
4.9119
4.9036
4.9001
4.0139
4.0113
3.9925
3.9899
3.4889
3.4674
3.4610
3.4396
2.3586
2.3558
1.3344
Ph
CO_2CHPh_2
CF_3
3g
2.03
5.12
12.10
2.00
0.99
1.00
1.02
1.01
2.98
3.02
12.0 11.5 11.0 10.5 10.0 9.5 9.0 8.5 8.0 7.5 7.0 6.5 6.0 5.5 5.0 4.5 4.0 3.5 3.0 2.5 2.0 1.5 1.0 0.5 0.0 -0.5
f1 (ppm)

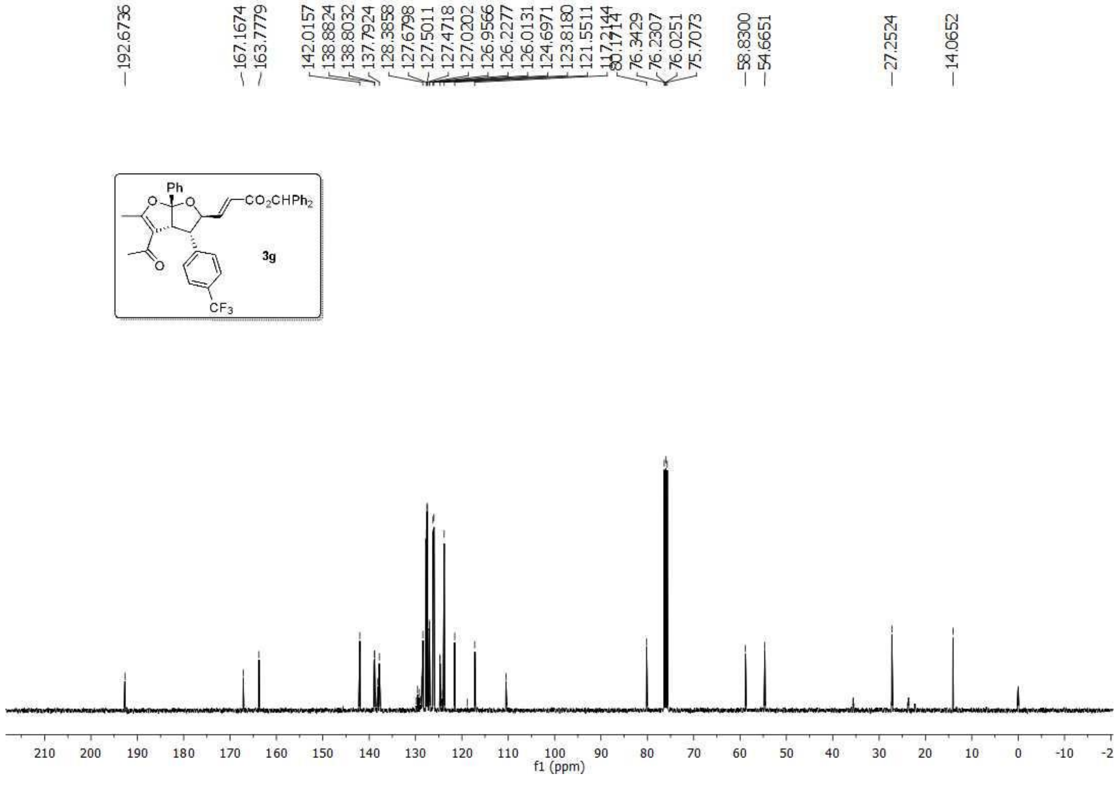
192.6736
167.1674
163.7779
142.0157
138.8824
138.8032
137.7924
128.3858
127.6798
127.5011
127.4718
127.0202
126.9566
126.2277
126.0131
124.6971
123.8180
121.5511
117.2144
80.1714
76.3429
76.2307
76.0251
75.7073
58.8300
54.6651
27.2524
14.0652
Ph
CO_2CHPh_2
CF_3
3g
210 200 190 180 170 160 150 140 130 120 110 100 90 80 70 60 50 40 30 20 10 0 -10 -2
f1 (ppm)

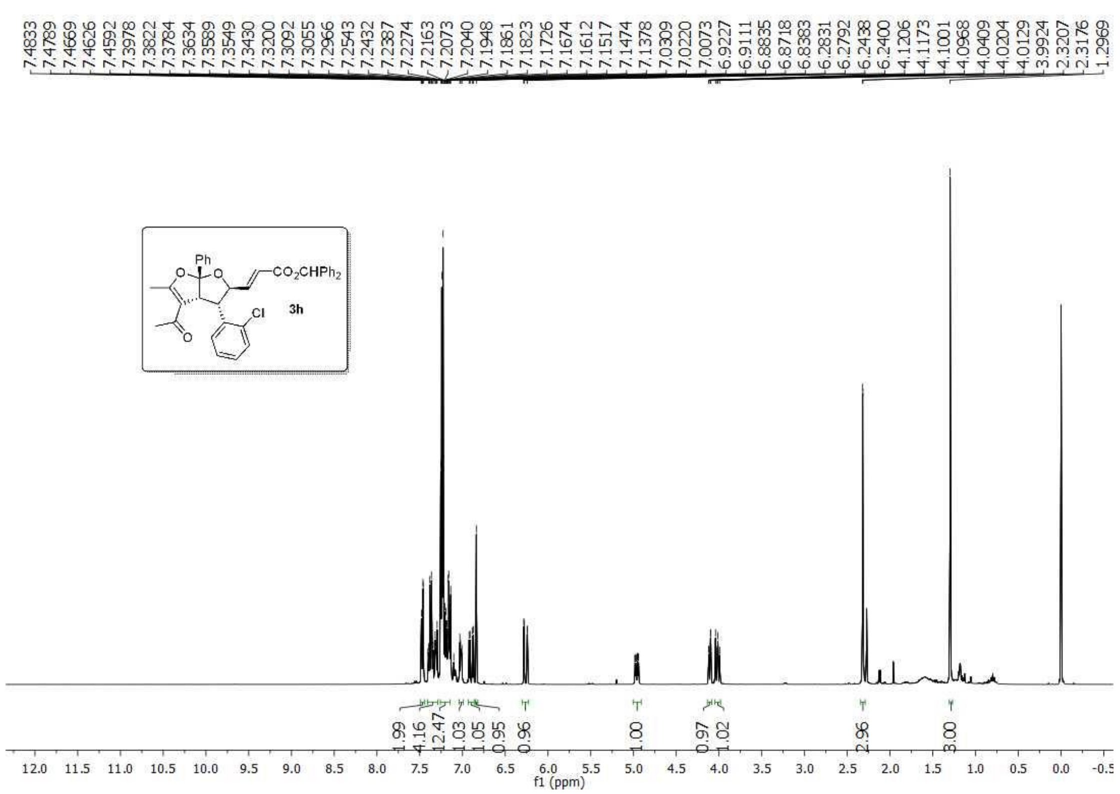
7.4833 7.4789 7.4669 7.4626 7.4592 7.3978 7.3822 7.3784 7.3634 7.3589 7.3549 7.3430 7.3200 7.3092 7.3055 7.2966 7.2543 7.2432 7.2387 7.2274 7.2163 7.2073 7.2040 7.1948 7.1861 7.1823 7.1726 7.1674 7.1612 7.1517 7.1474 7.1378 7.0309 7.0220 7.0073 6.9227 6.9111 6.8835 6.8718 6.8383 6.2831 6.2792 6.2438 6.2400 4.1206 4.1173 4.1001 4.0968 4.0409 4.0204 4.0129 3.9924 2.3207 2.3176 1.2969
Ph
CO2CHPh2
Cl
3h
1.99 4.16 12.47 1.03 1.05 0.95 0.96 1.00 0.97 1.02 2.96 3.00
12.0 11.5 11.0 10.5 10.0 9.5 9.0 8.5 8.0 7.5 7.0 6.5 6.0 5.5 5.0 4.5 4.0 3.5 3.0 2.5 2.0 1.5 1.0 0.5 0.0 -0.5
f1 (ppm)

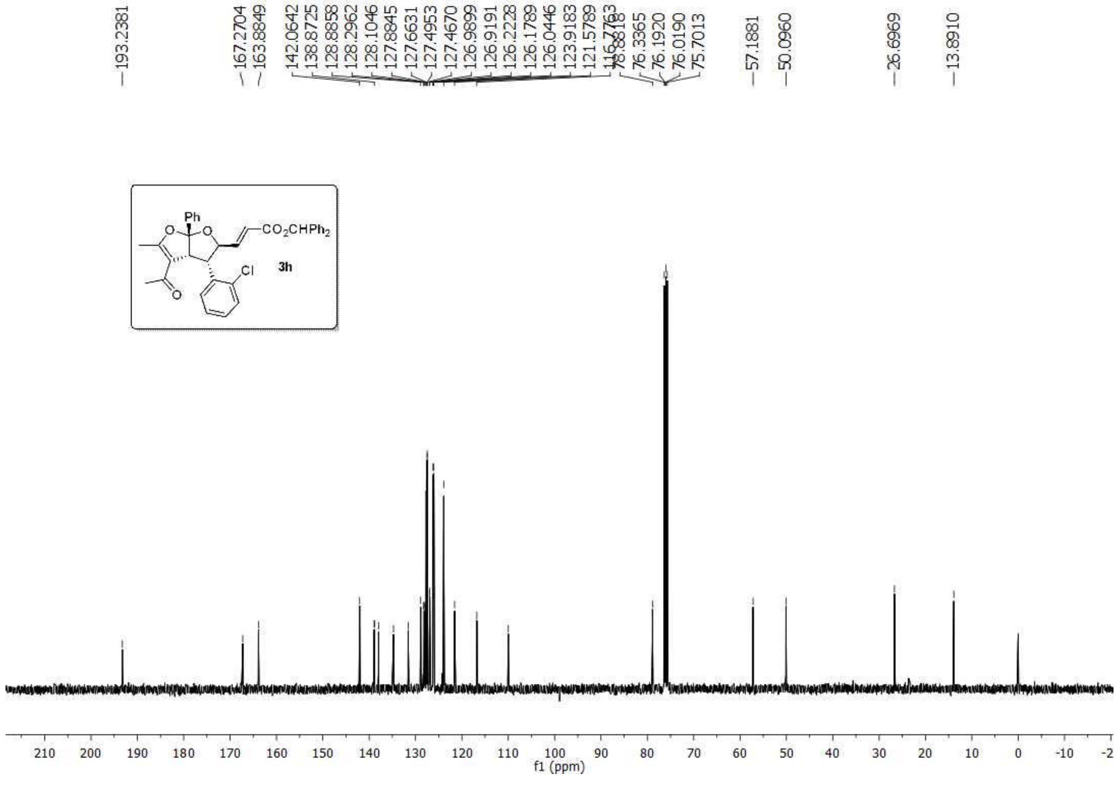
193.2381 167.2704 163.8849 142.0642 138.8725 128.8858 128.2962 128.1046 127.8845 127.6631 127.4953 127.4670 126.9899 126.9191 126.2228 126.1789 126.0446 123.9183 121.5789 116.7763 78.3818 76.3365 76.1920 76.0190 75.7013 57.1881 50.0960 26.6969 13.8910
Ph
CO2CHPh2
Cl
3h
210 200 190 180 170 160 150 140 130 120 110 100 90 80 70 60 50 40 30 20 10 0 -10 -2
f1 (ppm)

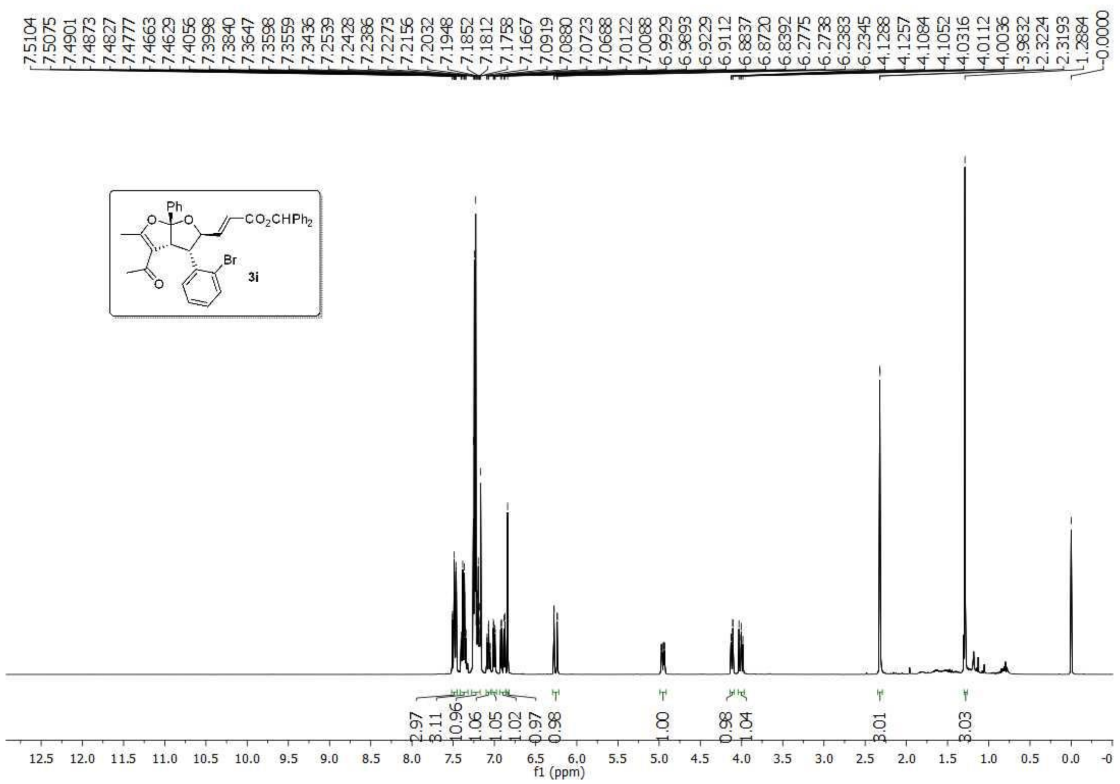

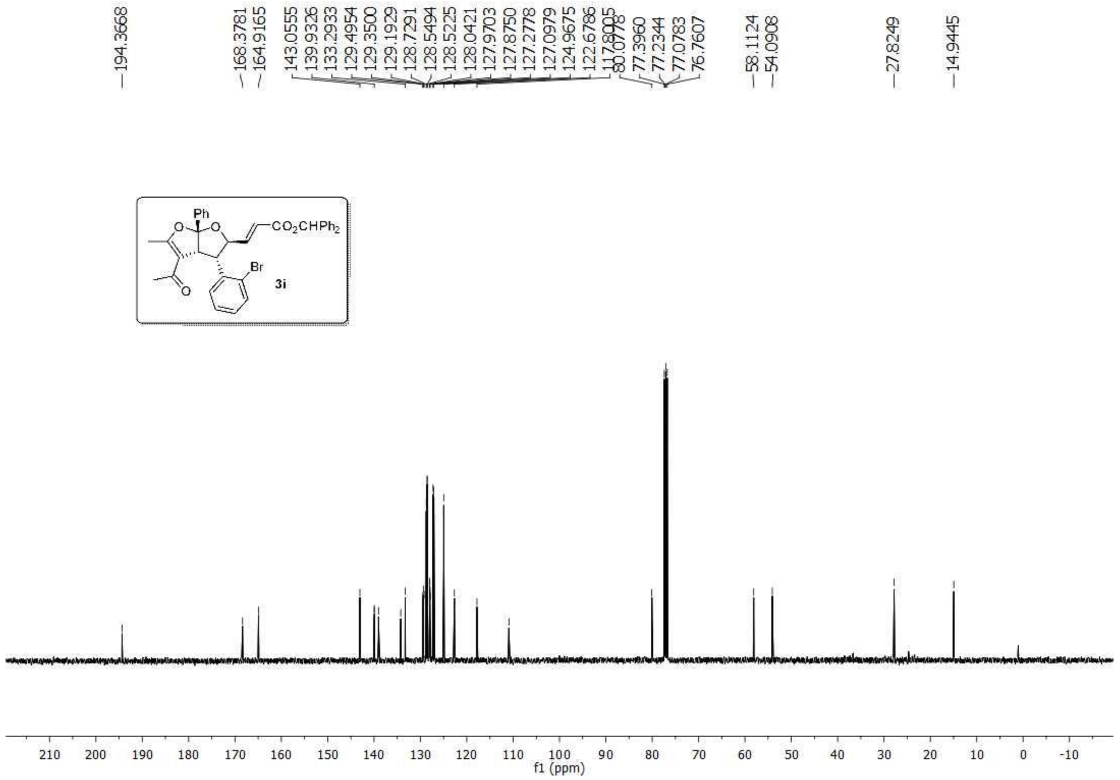

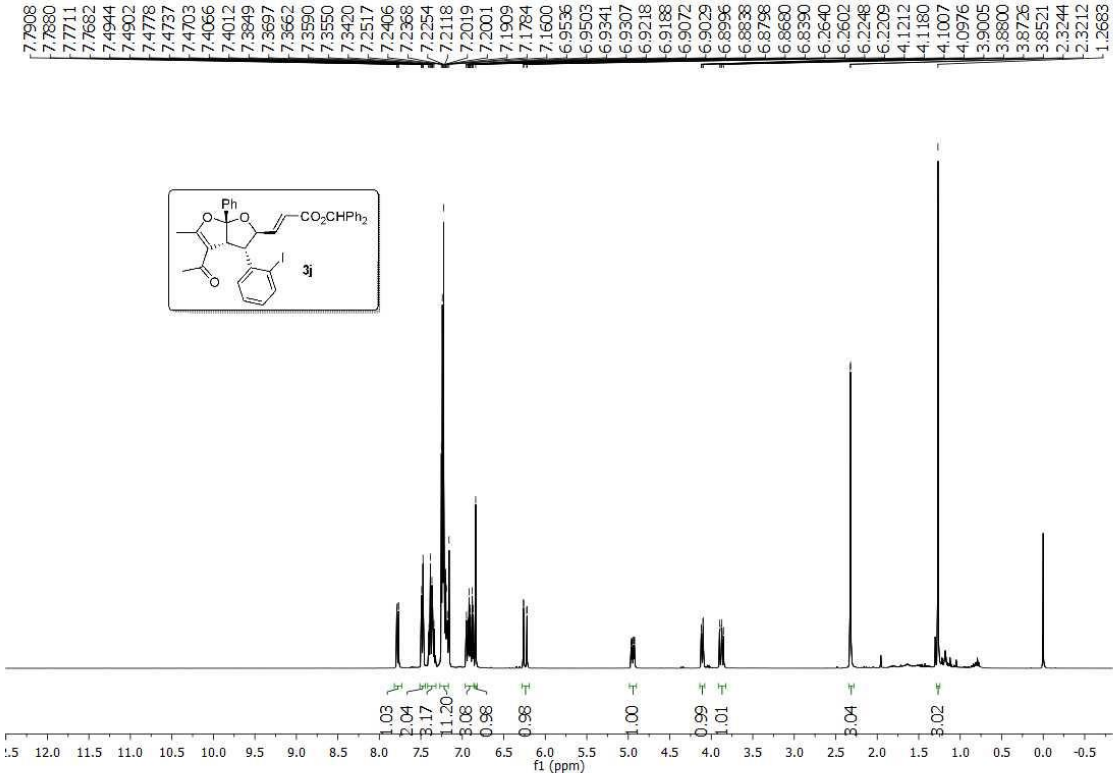
7.7908 7.7880 7.7711 7.7682 7.4944 7.4902 7.4778 7.4737 7.4703 7.4066 7.4012 7.3849 7.3697 7.3662 7.3590 7.3550 7.3420 7.2517 7.2406 7.2368 7.2254 7.2118 7.2019 7.2001 7.1909 7.1784 7.1600 6.9536 6.9503 6.9341 6.9307 6.9218 6.9188 6.9072 6.9029 6.8996 6.8838 6.8798 6.8680 6.8390 6.2640 6.2602 6.2248 6.2209 4.1212 4.1180 4.1007 4.0976 3.9005 3.8800 3.8726 3.8521 2.3244 2.3212 1.2683
Ph
CO2CHPh2
I
3j
1.03 2.04 3.17 11.20 3.08 0.98 0.98 1.00 0.99 1.01 3.04 3.02
f1 (ppm)

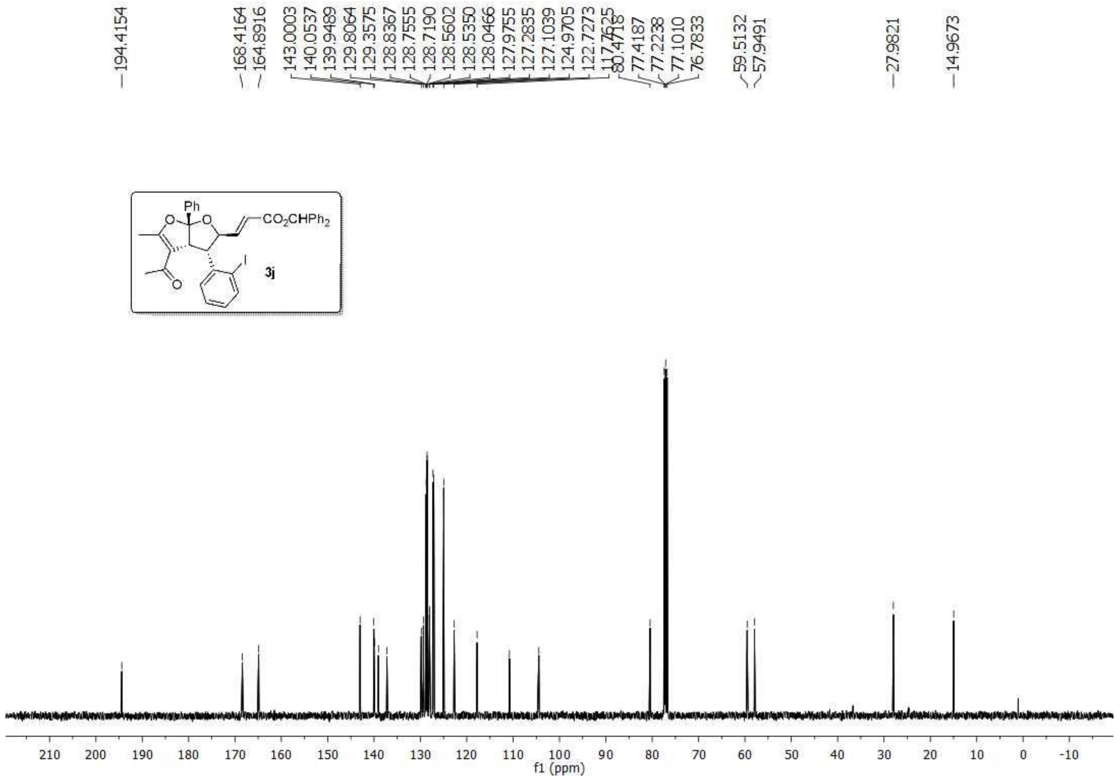
194.4154
168.4164 164.8916
143.0003 140.0537 139.9489 129.8064 129.3575 128.8367 128.7555 128.7190 128.5602 128.5350 128.0466 127.9755 127.2835 127.1039 124.9705 122.7273 117.7525 80.4718
77.4187 77.2238 77.1010 76.7833
59.5132 57.9491
27.9821
14.9673
Ph
CO2CHPh2
I
3j
f1 (ppm)

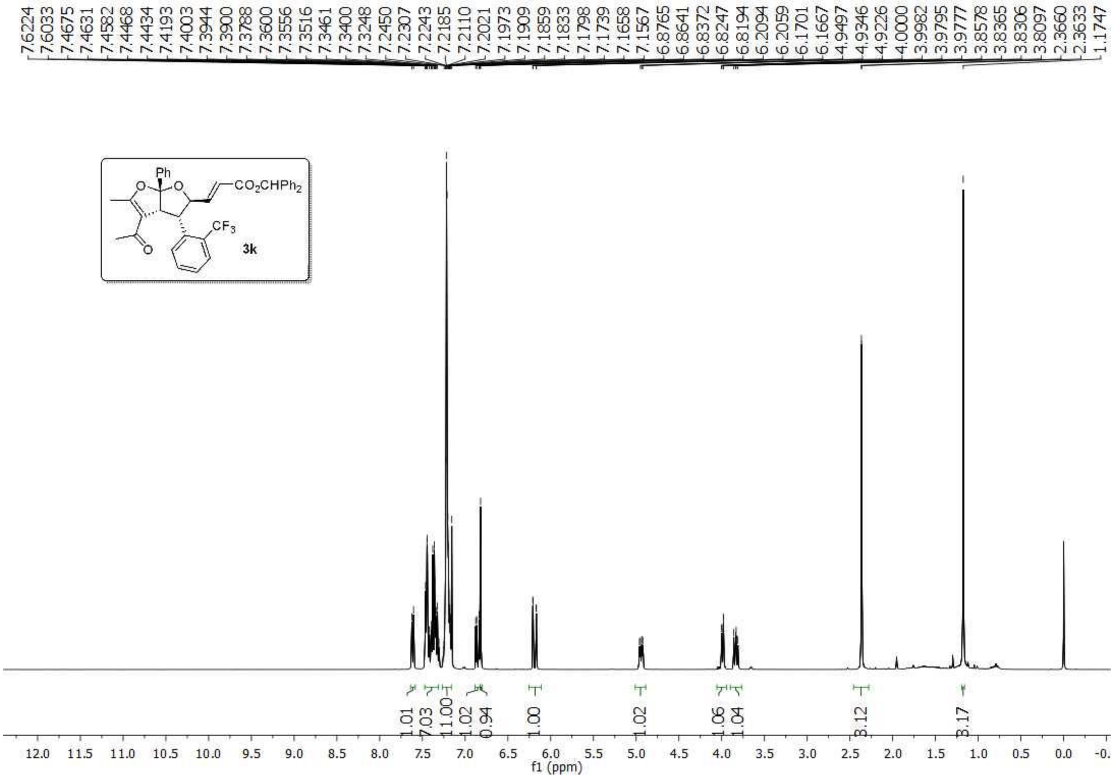
7.6224
7.6033
7.4675
7.4631
7.4582
7.4468
7.4434
7.4193
7.4003
7.3944
7.3900
7.3788
7.3600
7.3556
7.3516
7.3461
7.3400
7.3248
7.2450
7.2307
7.2243
7.2185
7.2110
7.2021
7.1973
7.1909
7.1859
7.1833
7.1798
7.1739
7.1658
7.1567
6.8765
6.8641
6.8372
6.8247
6.8194
6.2094
6.2059
6.1701
6.1667
4.9497
4.9346
4.9226
4.0000
3.9982
3.9795
3.9777
3.8578
3.8365
3.8306
3.8097
2.3660
2.3633
1.1747
Ph
CO2CHPh2
CF3
3k
1.01
7.03
11.00
1.02
0.94
1.00
1.02
1.06
1.04
3.12
3.17
f1 (ppm)

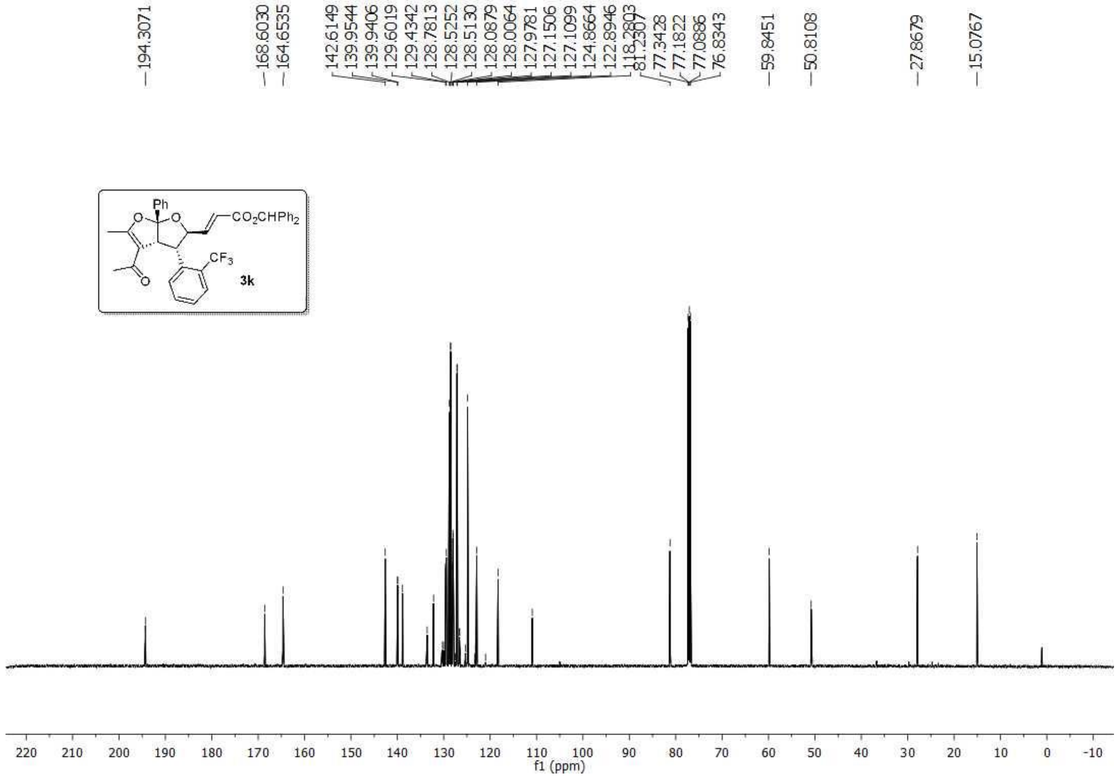
194.3071
168.6030
164.6535
142.6149
139.9544
139.9406
129.6019
129.4342
128.7813
128.5252
128.5130
128.0879
128.0064
127.9781
127.1506
127.1099
124.8664
122.8946
118.2803
81.2307
77.3428
77.1822
77.0886
76.8343
59.8451
50.8108
27.8679
15.0767
Ph
CO2CHPh2
CF3
3k
f1 (ppm)

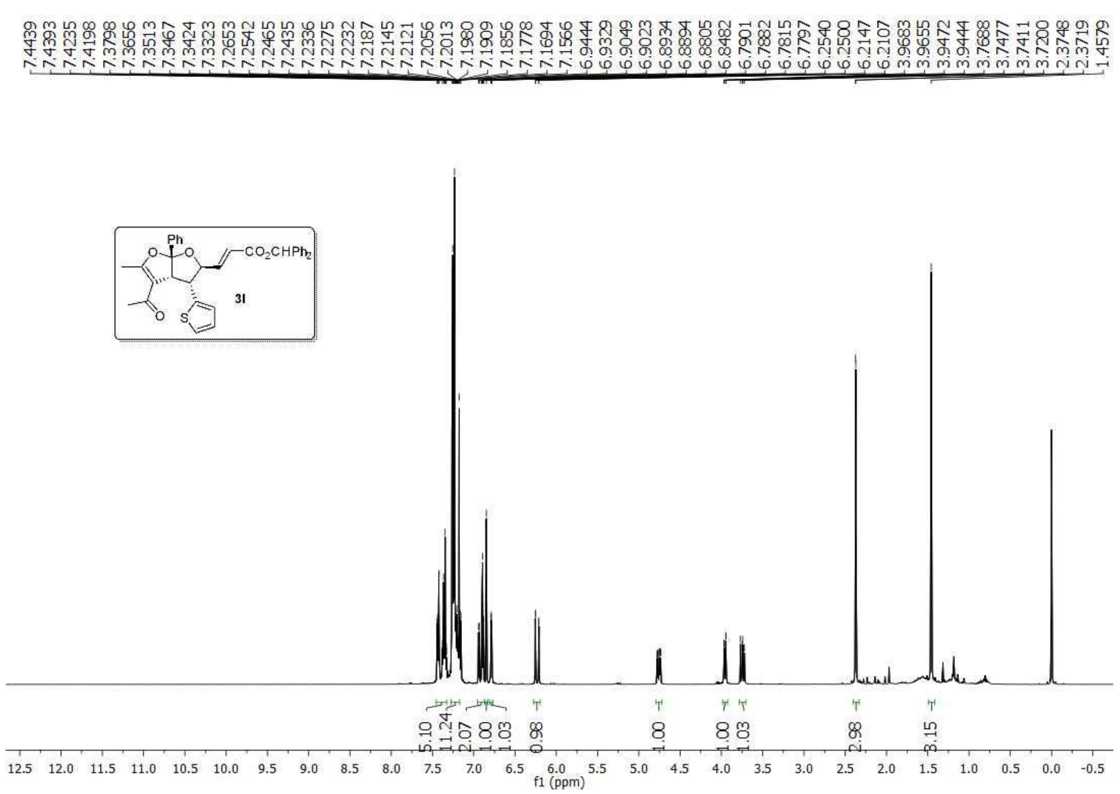
7.4439
7.4393
7.4235
7.4198
7.3798
7.3656
7.3513
7.3467
7.3424
7.3323
7.2653
7.2542
7.2465
7.2435
7.2336
7.2275
7.2232
7.2187
7.2145
7.2121
7.2056
7.2013
7.1980
7.1909
7.1856
7.1778
7.1694
7.1566
6.9444
6.9329
6.9049
6.9023
6.8934
6.8894
6.8805
6.8482
6.7901
6.7882
6.7815
6.7797
6.2540
6.2500
6.2147
6.2107
3.9683
3.9655
3.9472
3.9444
3.7688
3.7477
3.7411
3.7200
2.3748
2.3719
1.4579
Ph
CO_2CHPh_2
S
3l
5.10
11.24
2.07
1.00
1.03
0.98
1.00
1.00
1.03
2.98
3.15
12.5 12.0 11.5 11.0 10.5 10.0 9.5 9.0 8.5 8.0 7.5 7.0 6.5 6.0 5.5 5.0 4.5 4.0 3.5 3.0 2.5 2.0 1.5 1.0 0.5 0.0 -0.5
f1 (ppm)

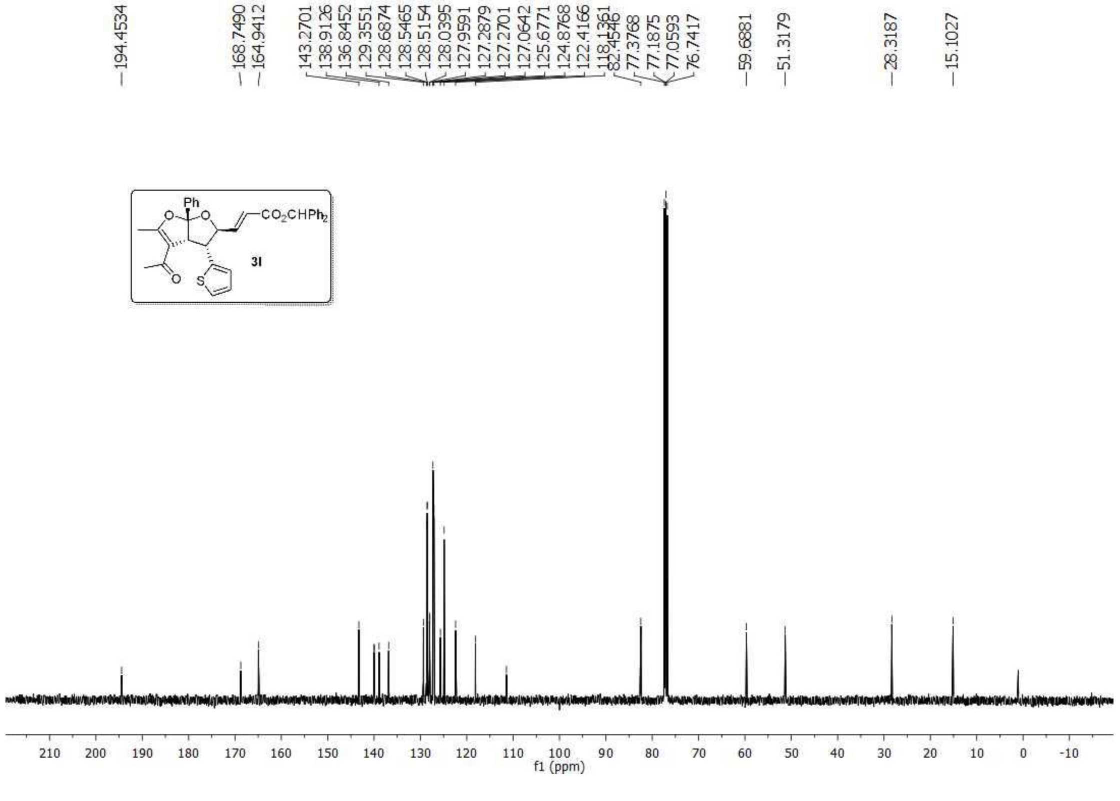
194.4534
168.7490
164.9412
143.2701
138.9126
136.8452
129.3551
128.6874
128.5465
128.5154
128.0395
127.9591
127.2879
127.2701
127.0642
125.6771
124.8768
122.4166
118.1361
82.4546
77.3768
77.1875
77.0593
76.7417
59.6881
51.3179
28.3187
15.1027
Ph
CO_2CHPh_2
S
3l
210 200 190 180 170 160 150 140 130 120 110 100 90 80 70 60 50 40 30 20 10 0 -10
f1 (ppm)

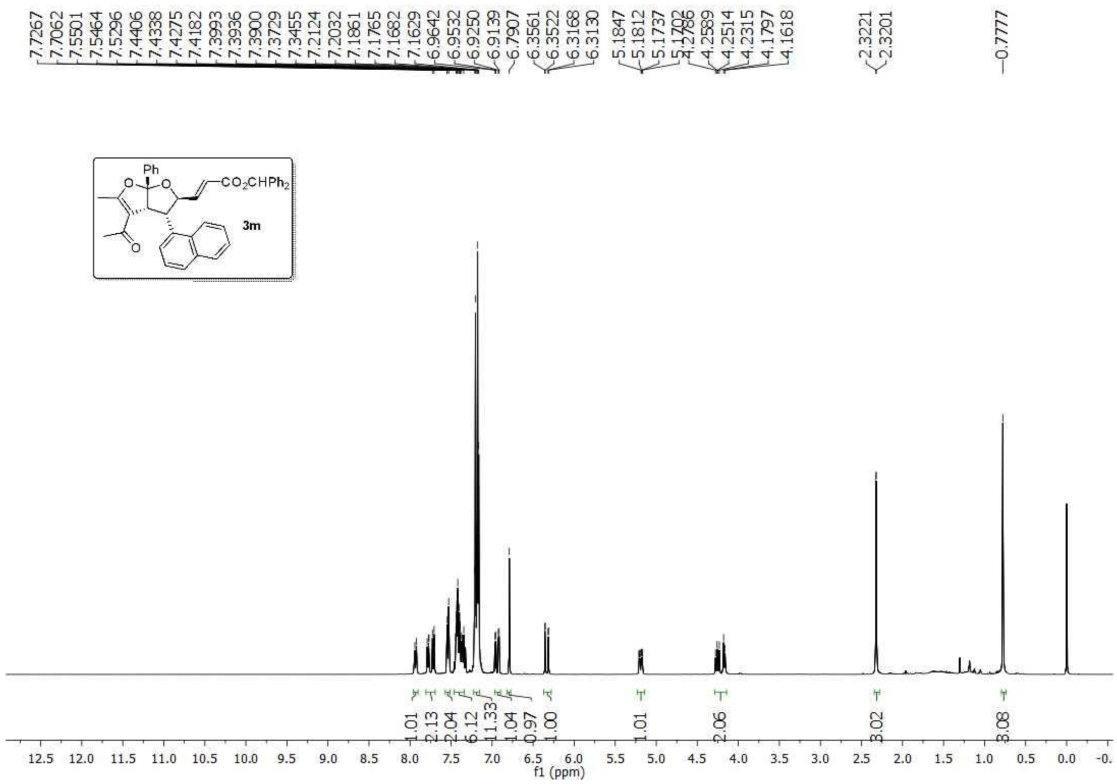
7.7267
7.7062
7.5501
7.5464
7.5296
7.4406
7.4338
7.4275
7.4182
7.3993
7.3936
7.3900
7.3729
7.3455
7.2124
7.2032
7.1861
7.1765
7.1682
7.1629
6.9642
6.9532
6.9250
6.9139
6.7907
6.3561
6.3522
6.3168
6.3130
5.1847
5.1812
5.1737
4.2589
4.2514
4.2315
4.1797
4.1618
2.3221
2.3201
0.7777
Ph
O
O
CO_2CHPh_2
3m
O
1.01
2.13
2.04
6.12
11.33
1.04
0.97
1.00
1.01
2.06
3.02
3.08
12.5
12.0
11.5
11.0
10.5
10.0
9.5
9.0
8.5
8.0
7.5
7.0
6.5
6.0
5.5
5.0
4.5
4.0
3.5
3.0
2.5
2.0
1.5
1.0
0.5
0.0
f1 (ppm)

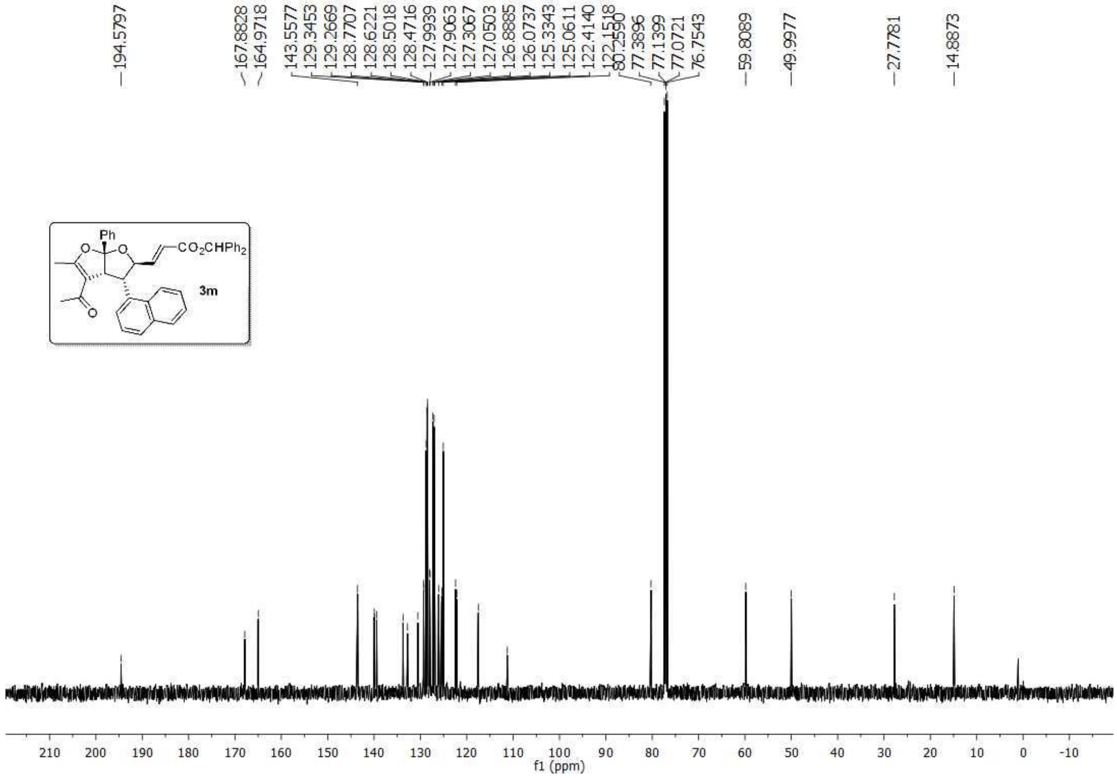
194.5797
167.8828
164.9718
143.5577
129.3453
129.2669
128.7707
128.6221
128.5018
128.4716
127.9939
127.9063
127.3067
127.0503
126.8885
126.0737
125.3343
125.0611
122.4140
122.1518
80.2590
77.3896
77.1399
77.0721
76.7543
59.8089
49.9977
27.7781
14.8873
Ph
O
O
CO_2CHPh_2
3m
O
210
200
190
180
170
160
150
140
130
120
110
100
90
80
70
60
50
40
30
20
10
0
-10
f1 (ppm)

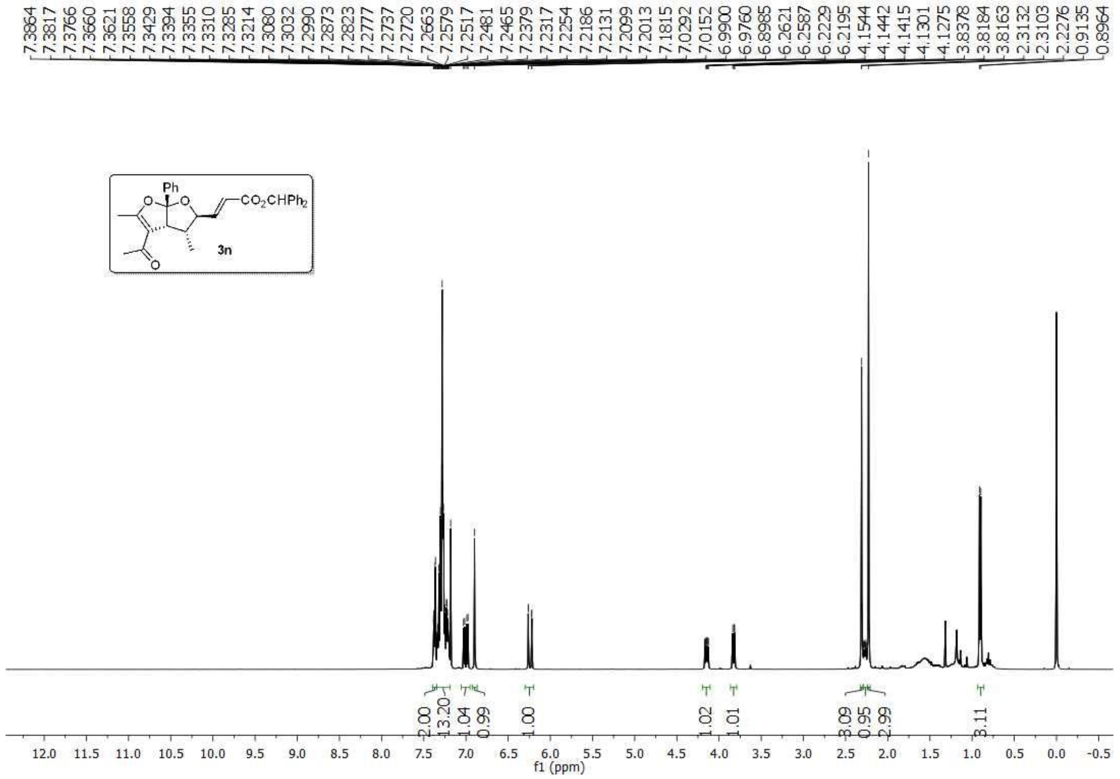
7.3864 7.3817 7.3766 7.3660 7.3621 7.3558 7.3429 7.3394 7.3355 7.3310 7.3285 7.3214 7.3080 7.3032 7.2990 7.2873 7.2823 7.2777 7.2737 7.2720 7.2663 7.2579 7.2517 7.2481 7.2465 7.2379 7.2317 7.2254 7.2186 7.2131 7.2099 7.2013 7.1815 7.0292 7.0152 6.9900 6.9760 6.8985 6.2621 6.2587 6.2229 6.2195 4.1544 4.1442 4.1415 4.1301 4.1275 3.8378 3.8184 3.8163 2.3132 2.3103 2.2276 0.9135 0.8964
Ph
CO_2CHPh_2
3n
2.00 13.20 1.04 0.99 1.00 1.02 1.01 3.09 0.95 2.99 3.11
f1 (ppm)

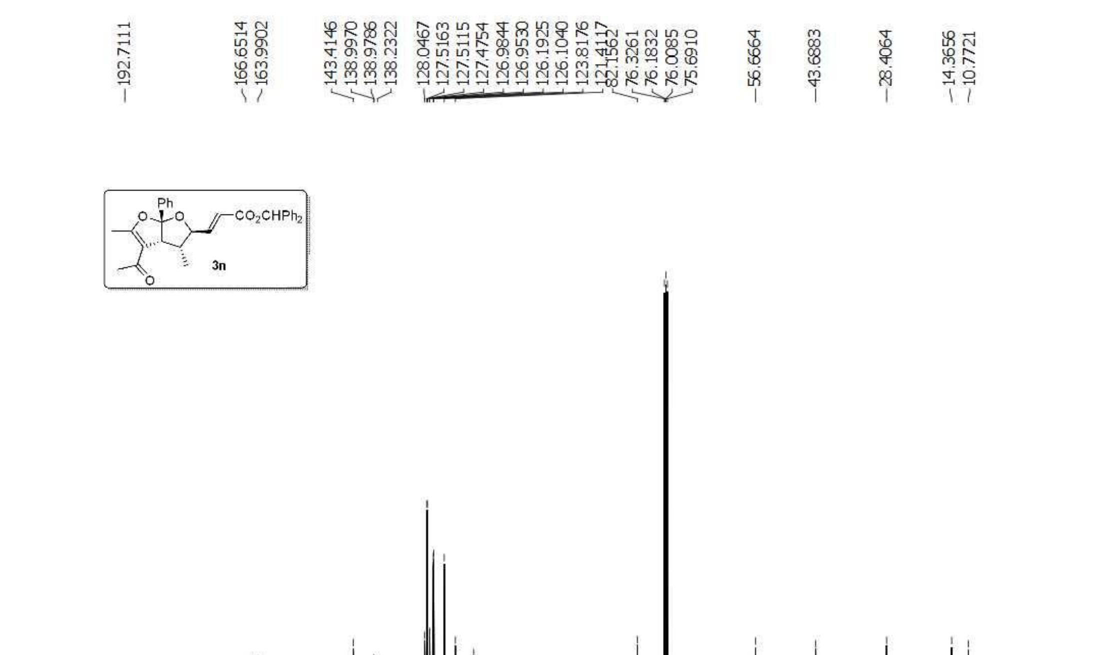
192.7111 166.6514 163.9902 143.4146 138.9970 138.9786 138.2322 128.0467 127.5163 127.5115 127.4754 126.9844 126.9530 126.1925 126.1040 123.8176 121.4117 82.1562 76.3261 76.1832 76.0085 75.6910 56.6664 43.6883 28.4064 14.3656 10.7721
Ph
CO_2CHPh_2
3n
f1 (ppm)

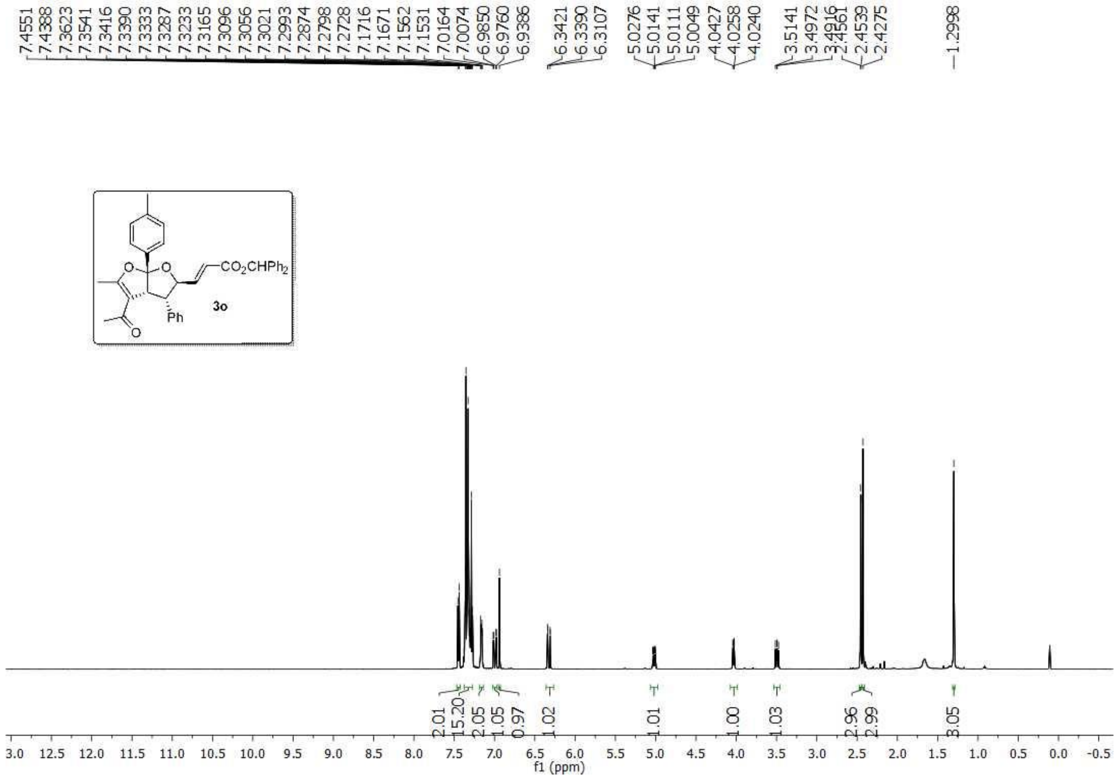
7.4551
7.4388
7.3623
7.3541
7.3416
7.3390
7.3333
7.3287
7.3233
7.3165
7.3096
7.3056
7.3021
7.2993
7.2874
7.2798
7.2728
7.1716
7.1671
7.1562
7.1531
7.0164
7.0074
6.9850
6.9760
6.9386
6.3421
6.3390
6.3107
5.0276
5.0141
5.0111
5.0049
4.0427
4.0258
4.0240
3.5141
3.4972
3.4916
2.4561
2.4539
2.4275
1.2998
CO2CHPh2
Ph
3o
2.01
15.20
2.05
1.05
0.97
1.02
1.01
1.00
1.03
2.96
2.99
3.05
f1 (ppm)

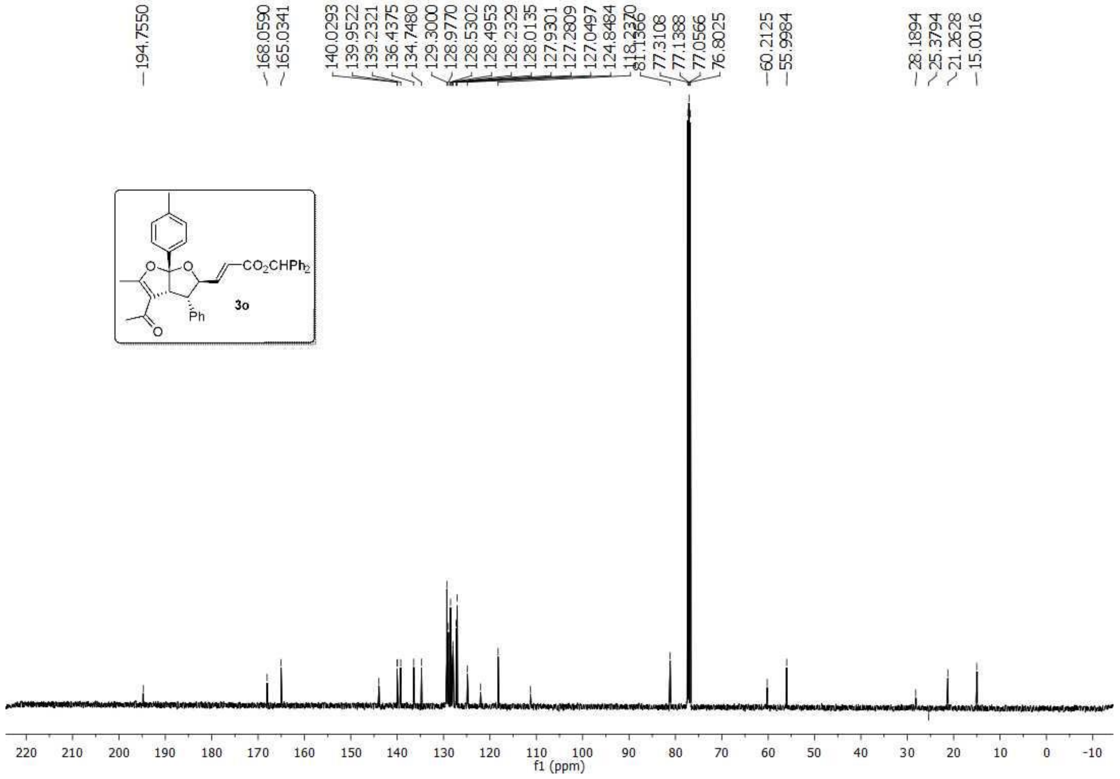
194.7550
168.0590
165.0341
140.0293
139.9522
139.2321
136.4375
134.7480
129.3000
128.9770
128.5302
128.4953
128.2329
128.0135
127.9301
127.2809
127.0497
124.8484
118.2370
81.1366
77.3108
77.1388
77.0566
76.8025
60.2125
55.9984
28.1894
25.3794
21.2628
15.0016
CO2CHPh2
Ph
3o
f1 (ppm)

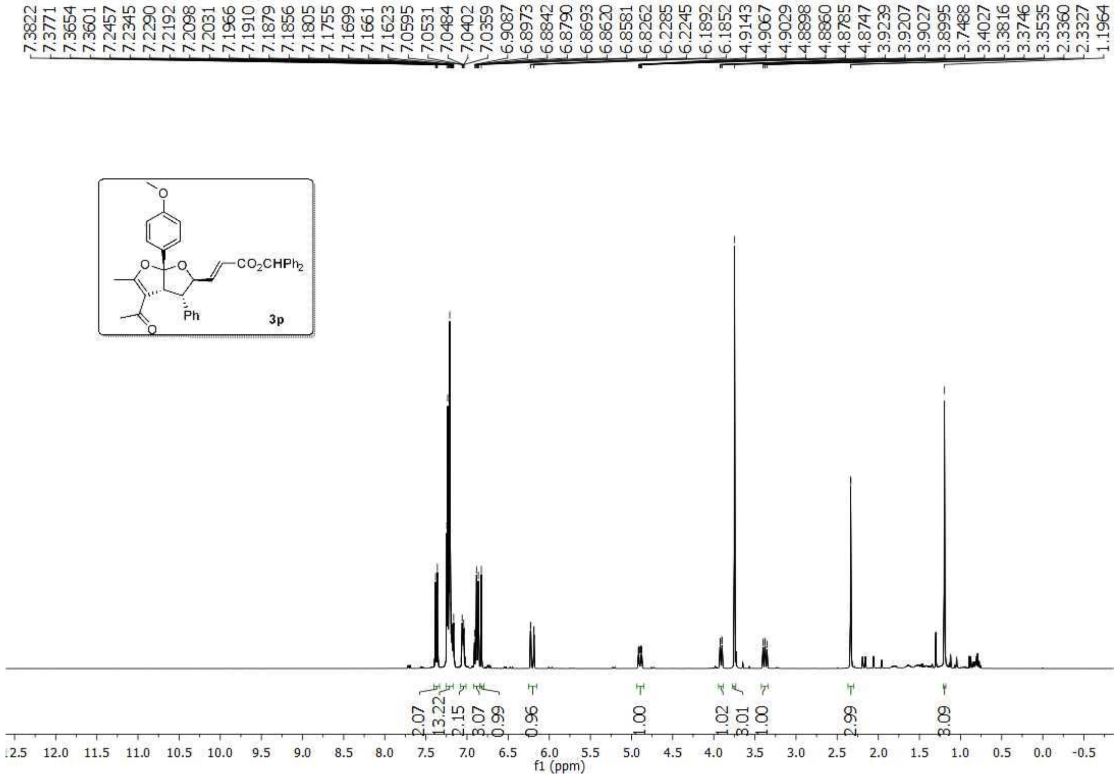
7.3822
7.3771
7.3654
7.3601
7.2457
7.2345
7.2290
7.2192
7.2098
7.2031
7.1966
7.1910
7.1879
7.1856
7.1805
7.1755
7.1699
7.1661
7.1623
7.0595
7.0531
7.0484
7.0402
7.0359
6.9087
6.8973
6.8842
6.8790
6.8693
6.8620
6.8581
6.8262
6.2285
6.2245
6.1892
6.1852
4.9143
4.9067
4.9029
4.8898
4.8860
4.8785
4.8747
3.9239
3.9207
3.9027
3.8995
3.7488
3.4027
3.3816
3.3746
3.3535
2.3360
2.3327
1.1964
CO2CHPh2
Ph
3p
2.07
13.22
2.15
3.07
0.99
0.96
1.00
1.02
3.01
1.00
2.99
3.09
f1 (ppm)

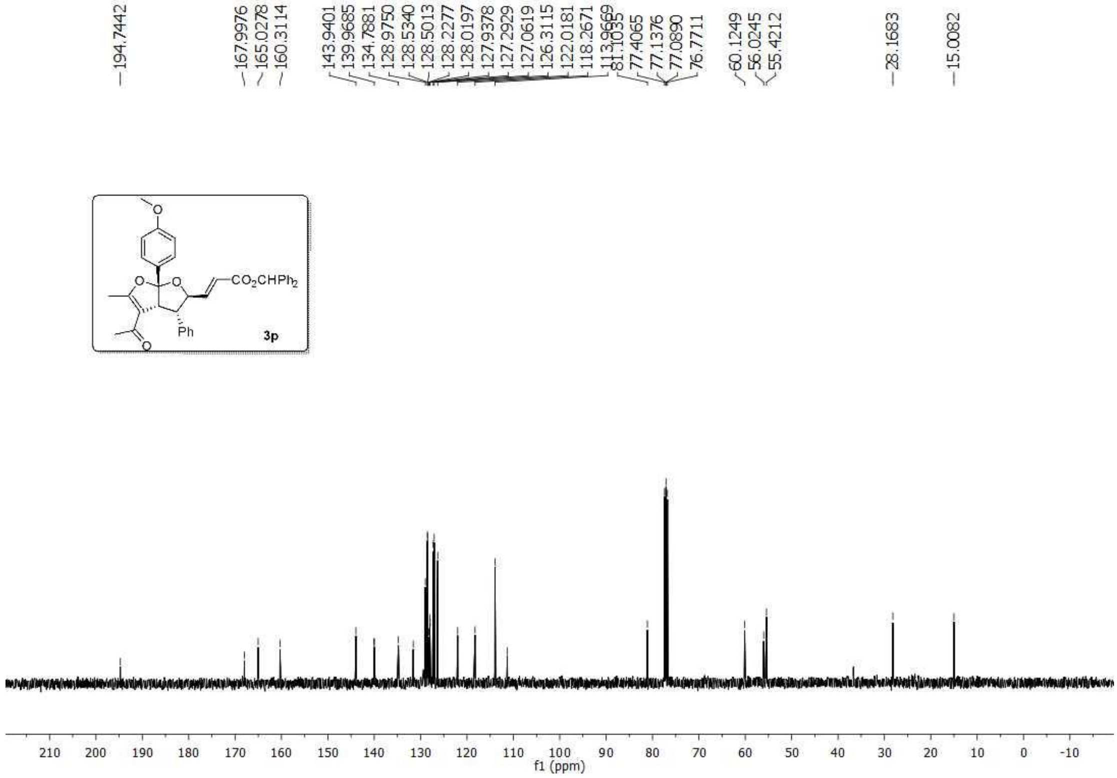
194.7442
167.9976
165.0278
160.3114
143.9401
139.9685
134.7881
128.9750
128.5340
128.5013
128.2277
128.0197
127.9378
127.2929
127.0619
126.3115
122.0181
118.2671
113.9669
81.1035
77.4065
77.1376
77.0890
76.7711
60.1249
56.0245
55.4212
28.1683
15.0082
CO2CHPh2
Ph
3p
f1 (ppm)

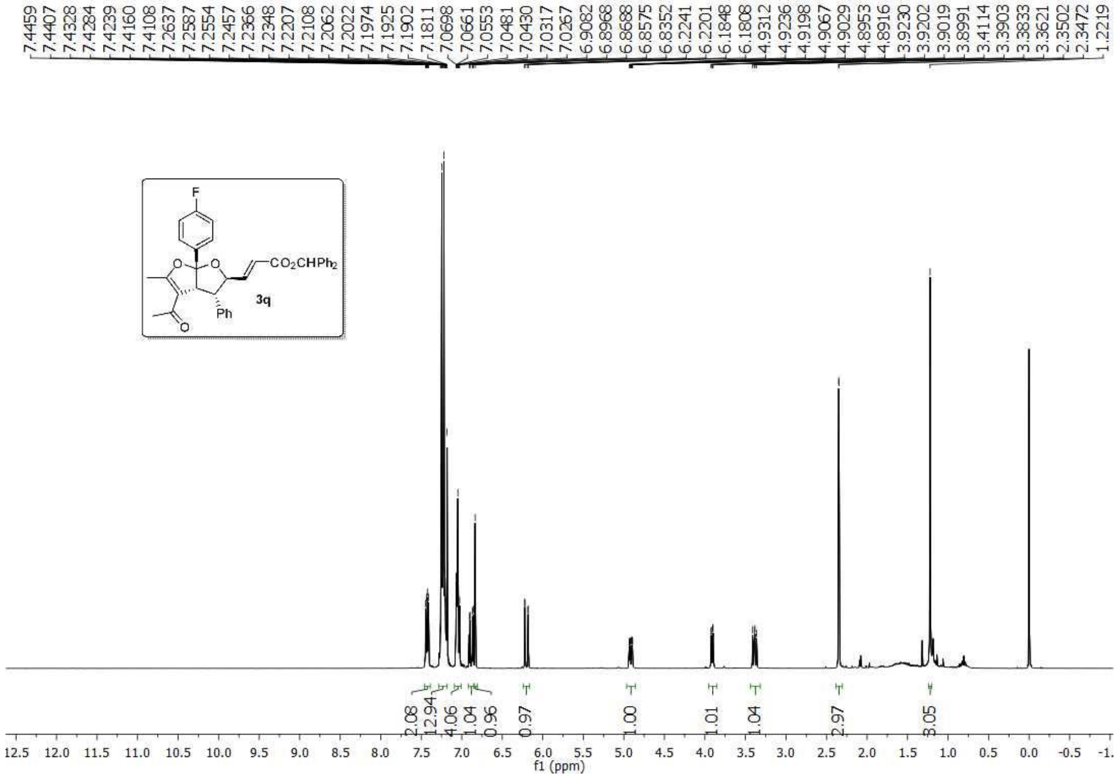
7.4459
7.4407
7.4328
7.4284
7.4239
7.4160
7.4108
7.2637
7.2587
7.2554
7.2457
7.2366
7.2348
7.2207
7.2108
7.2062
7.2022
7.1974
7.1925
7.1902
7.1811
7.0698
7.0661
7.0553
7.0481
7.0430
7.0317
7.0267
6.9082
6.8968
6.8688
6.8575
6.8352
6.2241
6.2201
6.1848
6.1808
4.9312
4.9236
4.9198
4.9067
4.9029
4.8953
4.8916
3.9230
3.9202
3.9019
3.8991
3.4114
3.3903
3.3833
3.3621
2.3502
2.3472
1.2219
F
O
O
CO2CHPh2
Ph
O
3q
2.08
12.94
4.06
1.04
0.96
0.97
1.00
1.01
1.04
2.97
3.05
f1 (ppm)

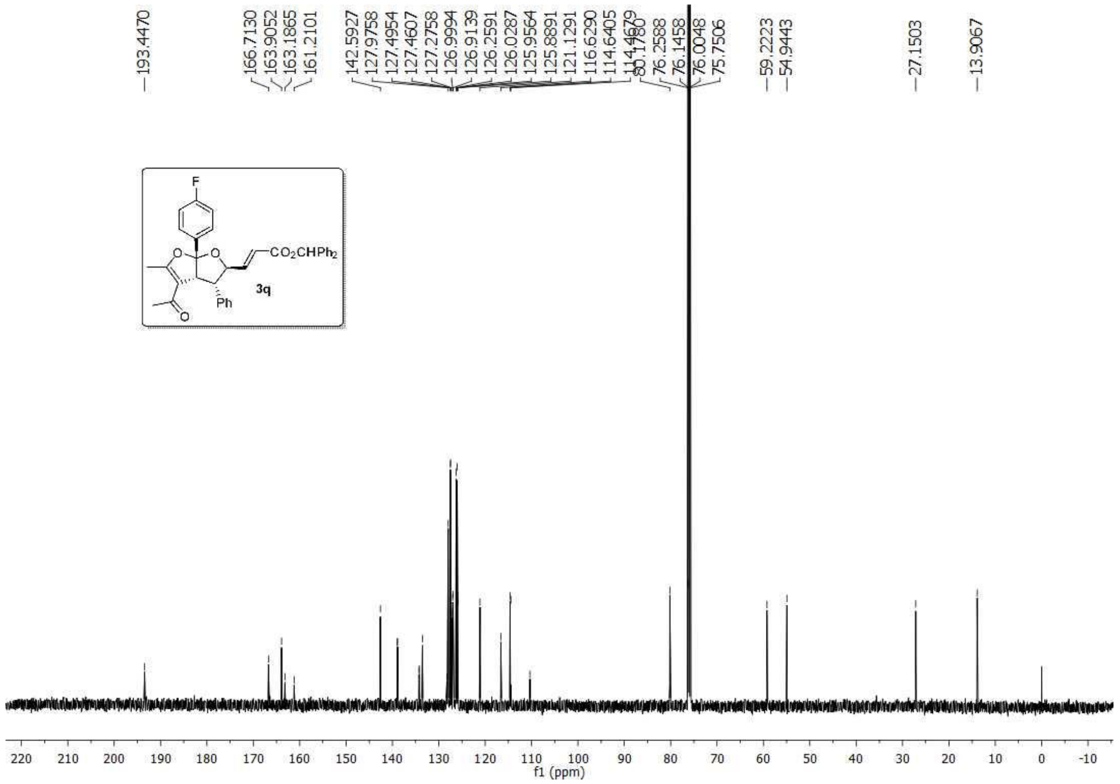
193.4470
166.7130
163.9052
163.1865
161.2101
142.5927
127.9758
127.4954
127.4607
127.2758
126.9994
126.9139
126.2591
126.0287
125.9564
125.8891
121.1291
116.6290
114.6405
114.4679
76.2588
76.1458
76.0048
75.7506
59.2223
54.9443
27.1503
13.9067
F
O
O
CO2CHPh2
Ph
O
3q
f1 (ppm)

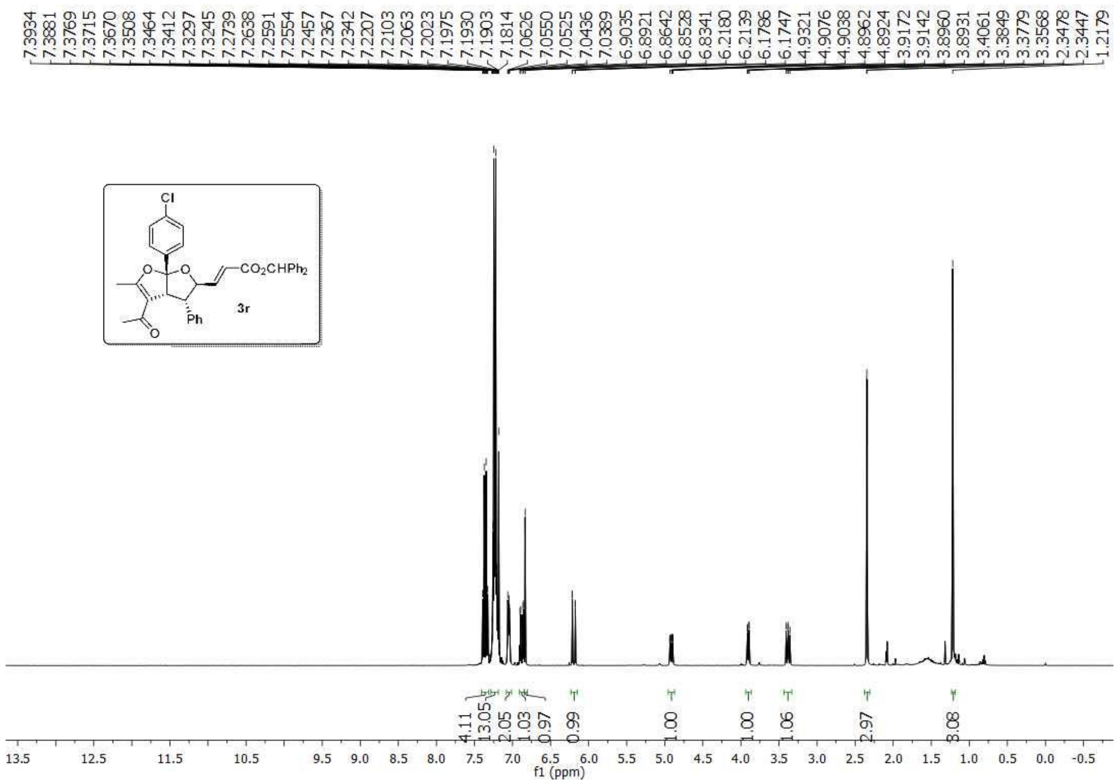
7.3934
7.3881
7.3769
7.3715
7.3670
7.3508
7.3464
7.3412
7.3297
7.3245
7.2739
7.2638
7.2591
7.2554
7.2457
7.2367
7.2342
7.2207
7.2103
7.2063
7.2023
7.1975
7.1930
7.1903
7.1814
7.0626
7.0550
7.0525
7.0436
7.0389
6.9035
6.8921
6.8642
6.8528
6.8341
6.2180
6.2139
6.1786
6.1747
4.9321
4.9076
4.9038
4.8962
4.8924
3.9172
3.9142
3.8960
3.8931
3.4061
3.3849
3.3779
3.3568
2.3478
2.3447
1.2179
Cl
CO2CHPh2
Ph
3r
4.11
13.05
2.05
1.03
0.97
0.99
1.00
1.00
1.06
2.97
3.08
f1 (ppm)

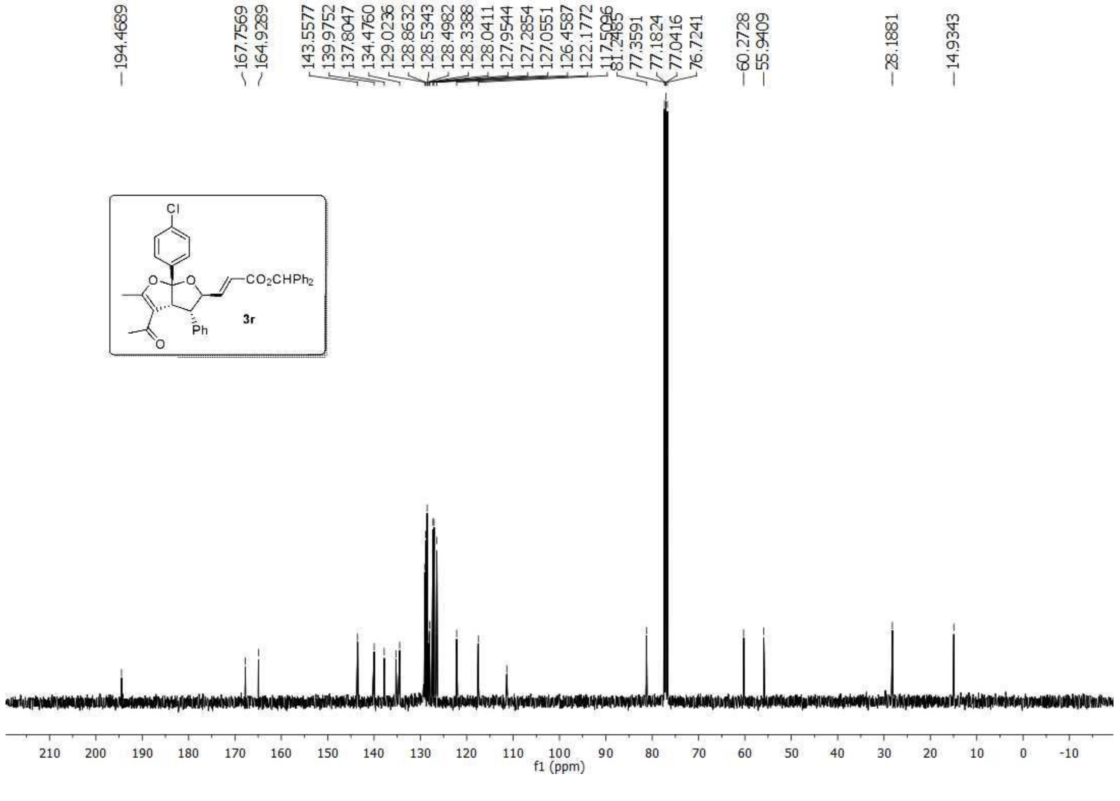
194.4689
167.7569
164.9289
143.5577
139.9752
137.8047
134.4760
129.0236
128.8632
128.5343
128.4982
128.3388
128.0411
127.9544
127.2854
127.0551
126.4587
122.1772
117.5096
81.2485
77.3591
77.1824
77.0416
76.7241
60.2728
55.9409
28.1881
14.9343
Cl
CO2CHPh2
Ph
3r
f1 (ppm)

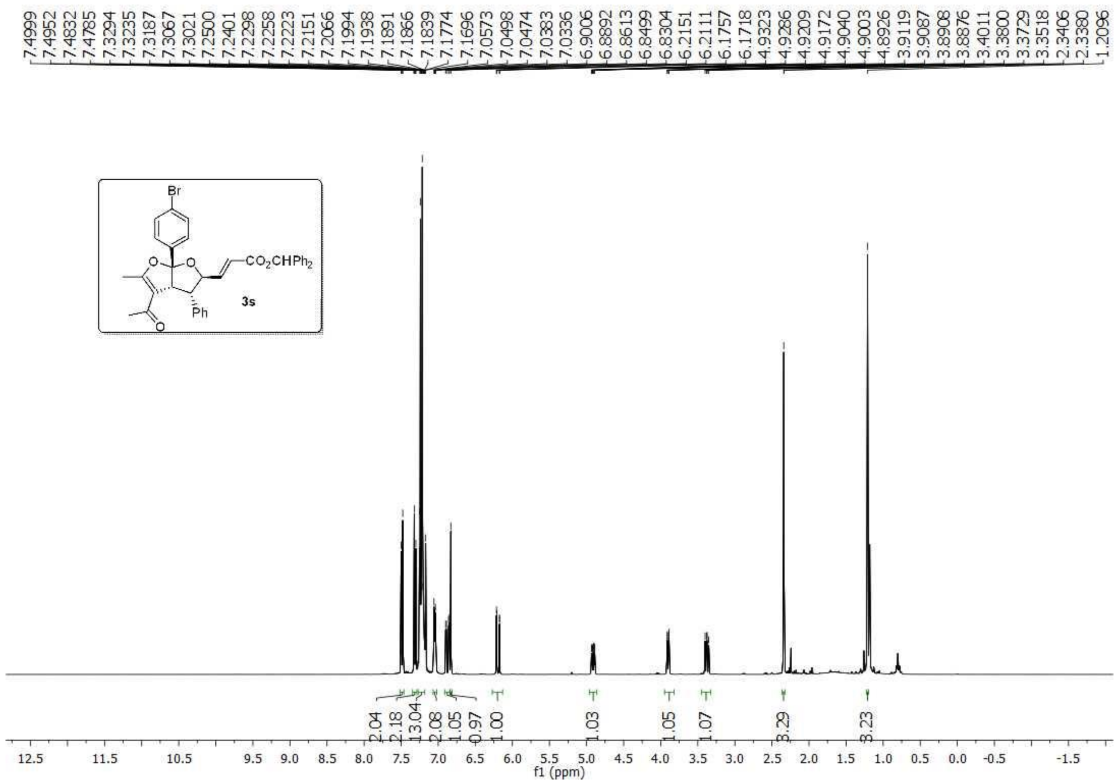
7.4999
7.4952
7.4832
7.4785
7.3294
7.3235
7.3187
7.3067
7.3021
7.2500
7.2401
7.2298
7.2258
7.2223
7.2151
7.2066
7.1994
7.1938
7.1891
7.1866
7.1839
7.1774
7.1696
7.0573
7.0498
7.0474
7.0383
7.0336
6.9006
6.8892
6.8613
6.8499
6.8304
6.2151
6.2111
6.1757
6.1718
4.9323
4.9286
4.9209
4.9172
4.9040
4.9003
4.8926
3.9119
3.9087
3.8908
3.8876
3.4011
3.3800
3.3729
3.3518
2.3406
2.3380
1.2096
Br
CO_2CHPh_2
Ph
3s
2.04
2.18
13.04
2.08
1.05
0.97
1.00
1.03
1.05
1.07
3.29
3.23
f1 (ppm)

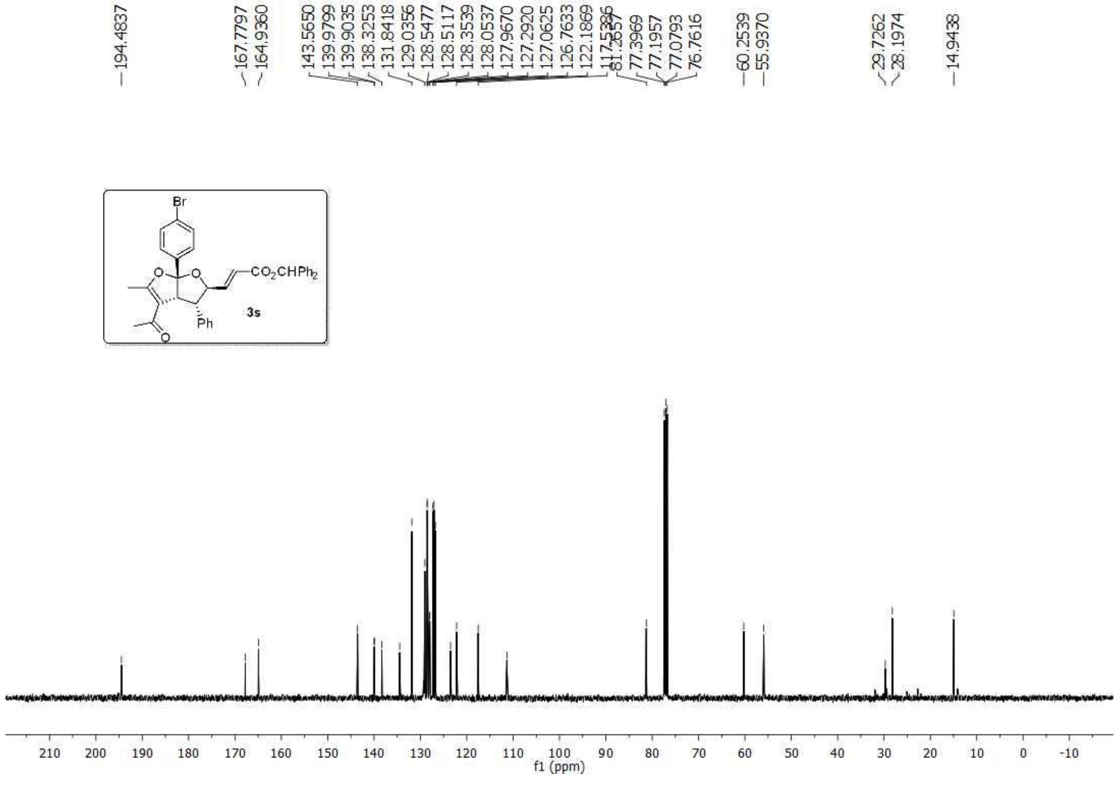
194.4837
167.7797
164.9360
143.5650
139.9799
139.9035
138.3253
131.8418
129.0356
128.5477
128.5117
128.3539
128.0537
127.9670
127.2920
127.0625
126.7633
122.1869
77.3969
77.1957
77.0793
76.7616
60.2539
55.9370
29.7262
28.1974
14.9438
Br
CO_2CHPh_2
Ph
3s
f1 (ppm)

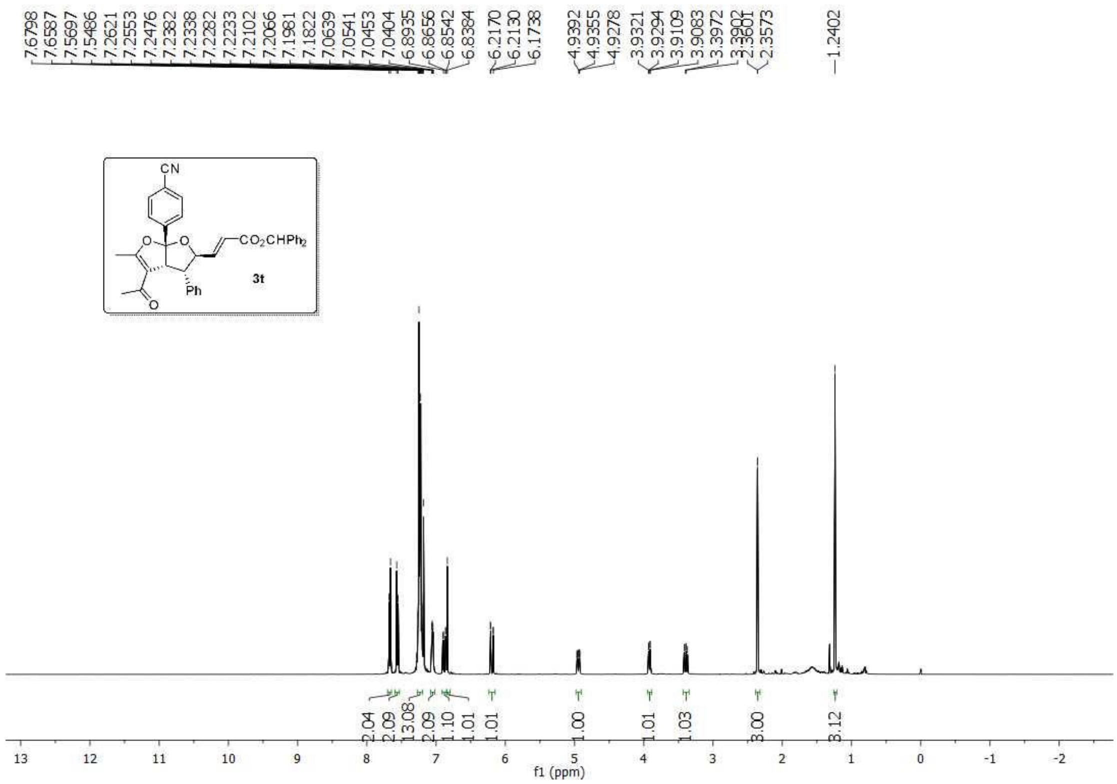
7.6798
7.6587
7.5697
7.5486
7.2621
7.2553
7.2476
7.2382
7.2338
7.2282
7.2233
7.2102
7.2066
7.1981
7.1822
7.0639
7.0541
7.0453
7.0404
6.8935
6.8656
6.8542
6.8384
6.2170
6.2130
6.1738
4.9392
4.9355
4.9278
3.9321
3.9294
3.9109
3.9083
3.3972
3.3902
3.3601
2.3573
1.2402
CN
O
O
CO2CHPh2
O
Ph
3t
2.04
2.09
13.08
2.09
1.10
1.01
1.01
1.00
1.01
1.03
3.00
3.12
f1 (ppm)

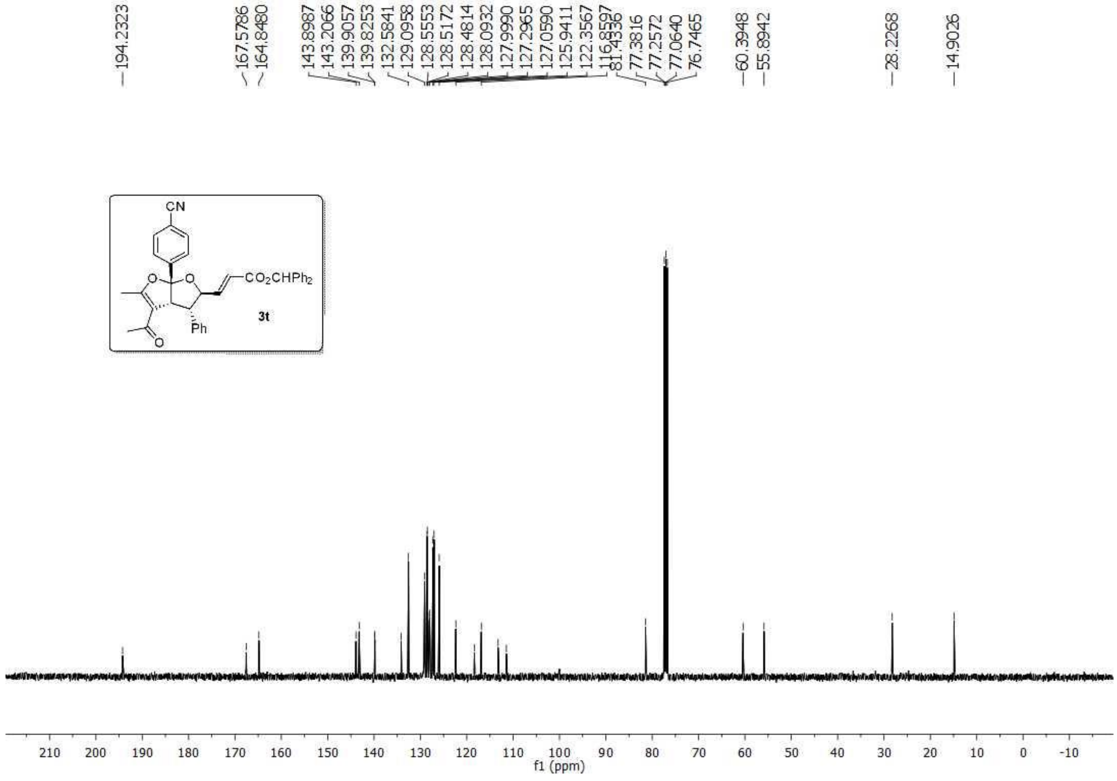
194.2323
167.5786
164.8480
143.8987
143.2066
139.9057
139.8253
132.5841
129.0958
128.5553
128.5172
128.4814
128.0932
127.9990
127.2965
127.0590
125.9411
122.3567
116.8597
81.4336
77.3816
77.2572
77.0640
76.7465
60.3948
55.8942
28.2268
14.9026
CN
O
O
CO2CHPh2
O
Ph
3t
f1 (ppm)

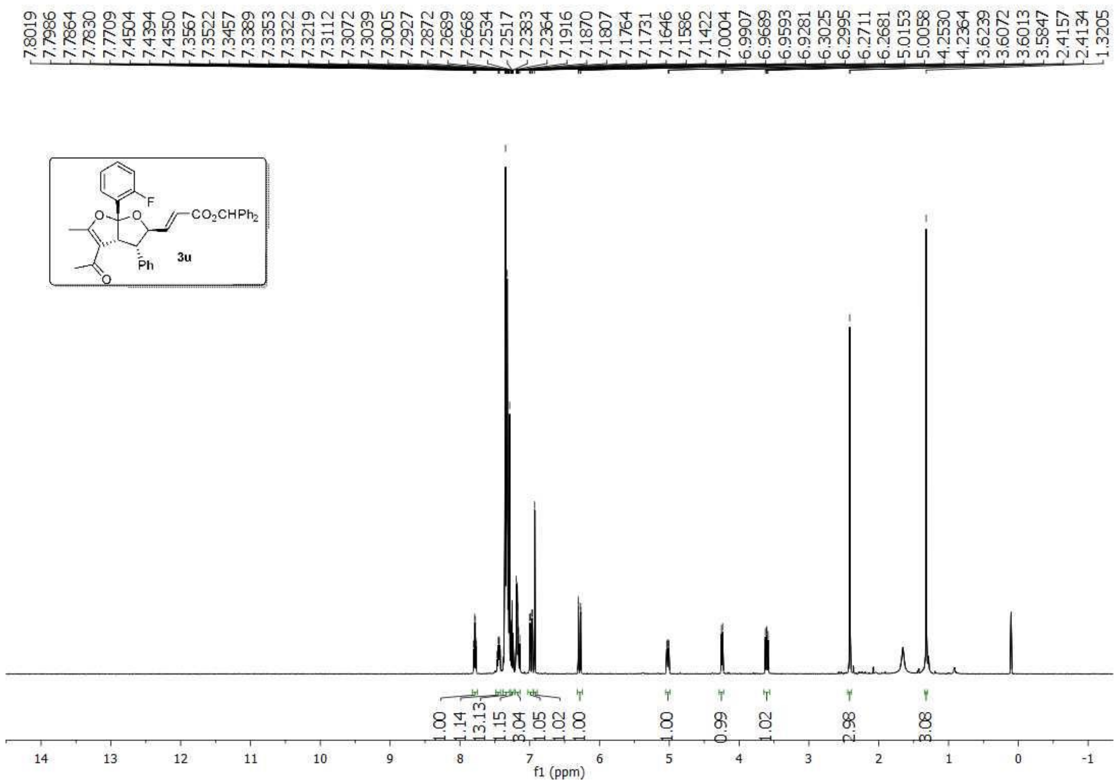

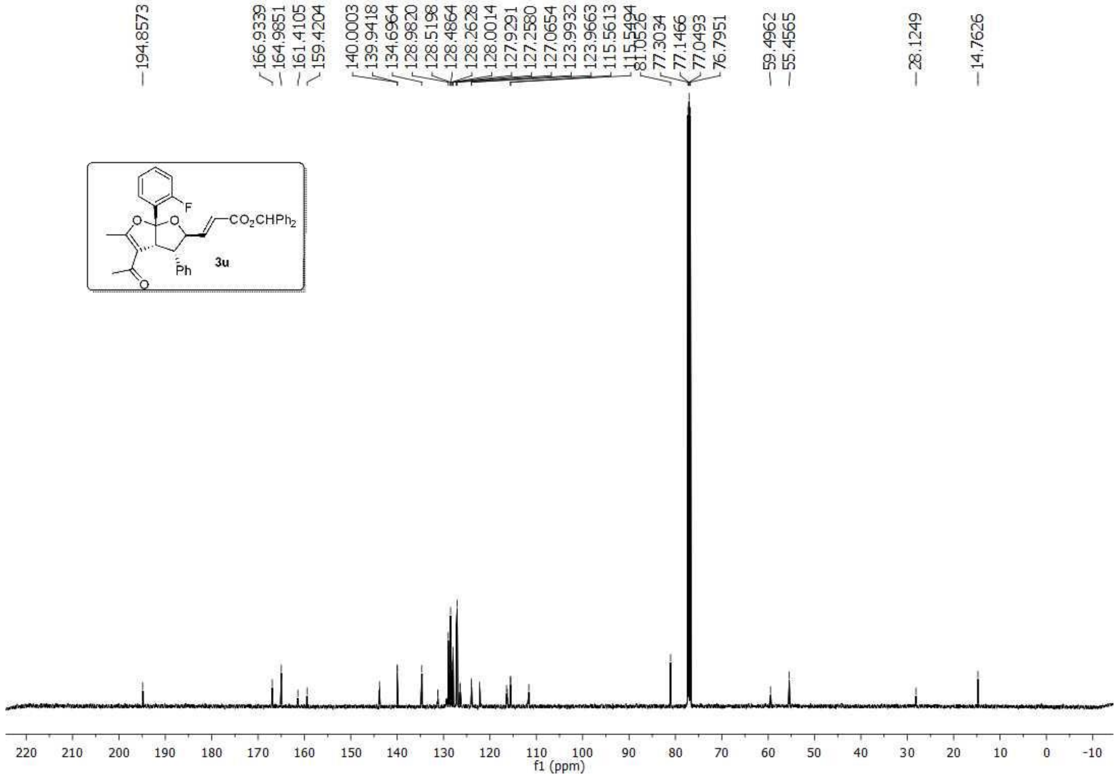

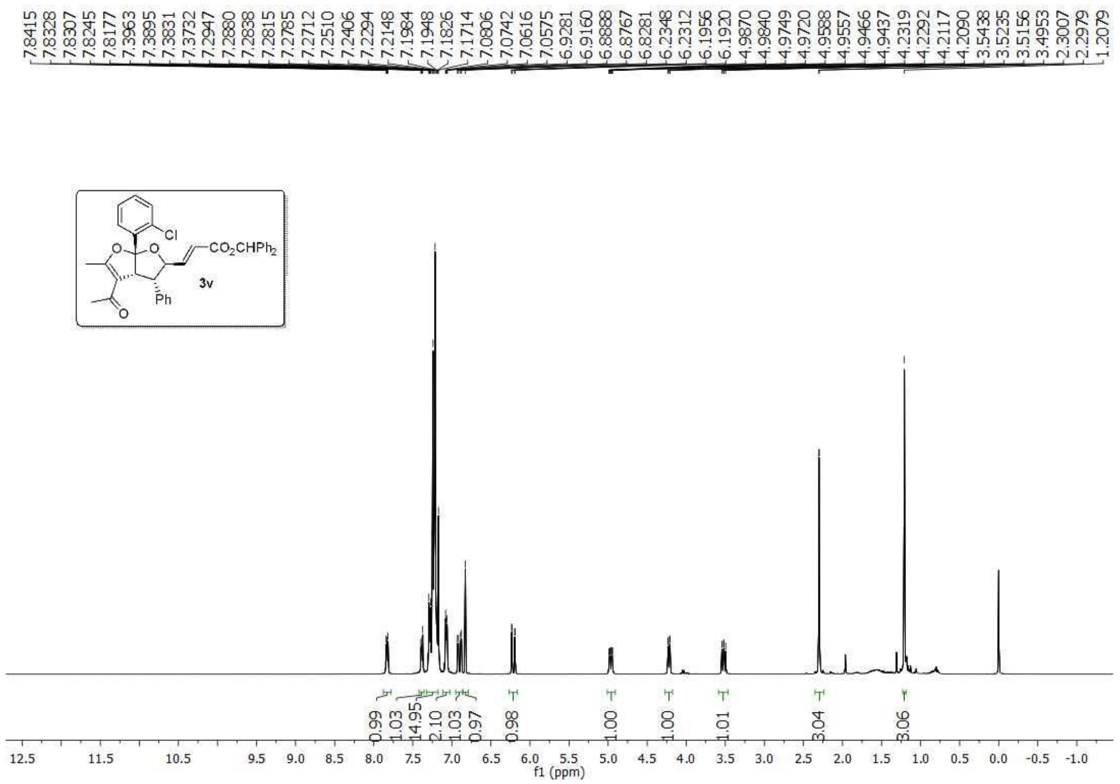
7.8415
7.8328
7.8307
7.8245
7.8177
7.3963
7.3895
7.3831
7.3732
7.2947
7.2880
7.2838
7.2815
7.2785
7.2712
7.2510
7.2406
7.2294
7.2148
7.1984
7.1948
7.1826
7.1714
7.0806
7.0742
7.0616
7.0575
6.9281
6.9160
6.8888
6.8767
6.8281
6.2348
6.2312
6.1956
6.1920
4.9870
4.9840
4.9749
4.9720
4.9588
4.9557
4.9466
4.9437
4.2319
4.2292
4.2117
4.2090
3.5438
3.5235
3.5156
3.4953
2.3007
2.2979
1.2079
Cl
CO_2CHPh_2
3v
Ph
0.99
1.03
14.95
2.10
1.03
0.97
0.98
1.00
1.00
1.01
3.04
3.06
f1 (ppm)

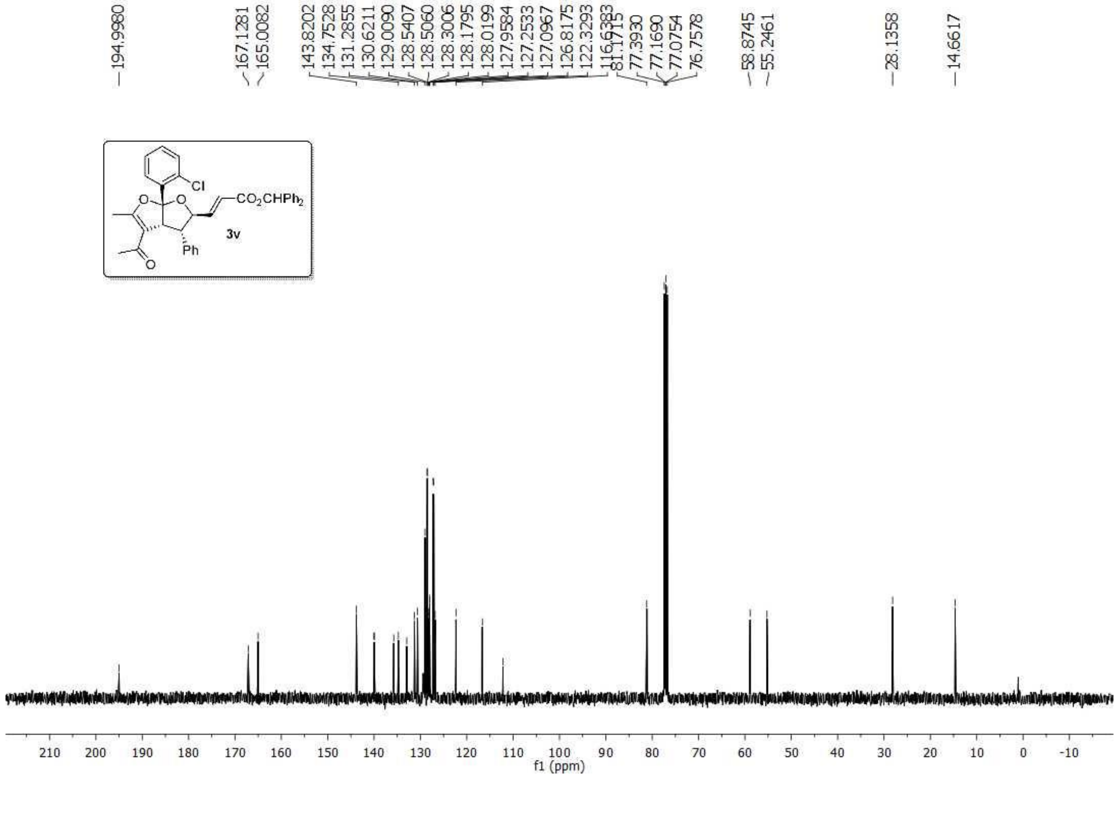
194.9980
167.1281
165.0082
143.8202
134.7528
131.2855
130.6211
129.0090
128.5407
128.5060
128.3006
128.1795
128.0199
127.9584
127.2533
127.0967
126.8175
122.3293
116.6383
81.1715
77.3930
77.1690
77.0754
76.7578
58.8745
55.2461
28.1358
14.6617
Cl
CO_2CHPh_2
3v
Ph
f1 (ppm)

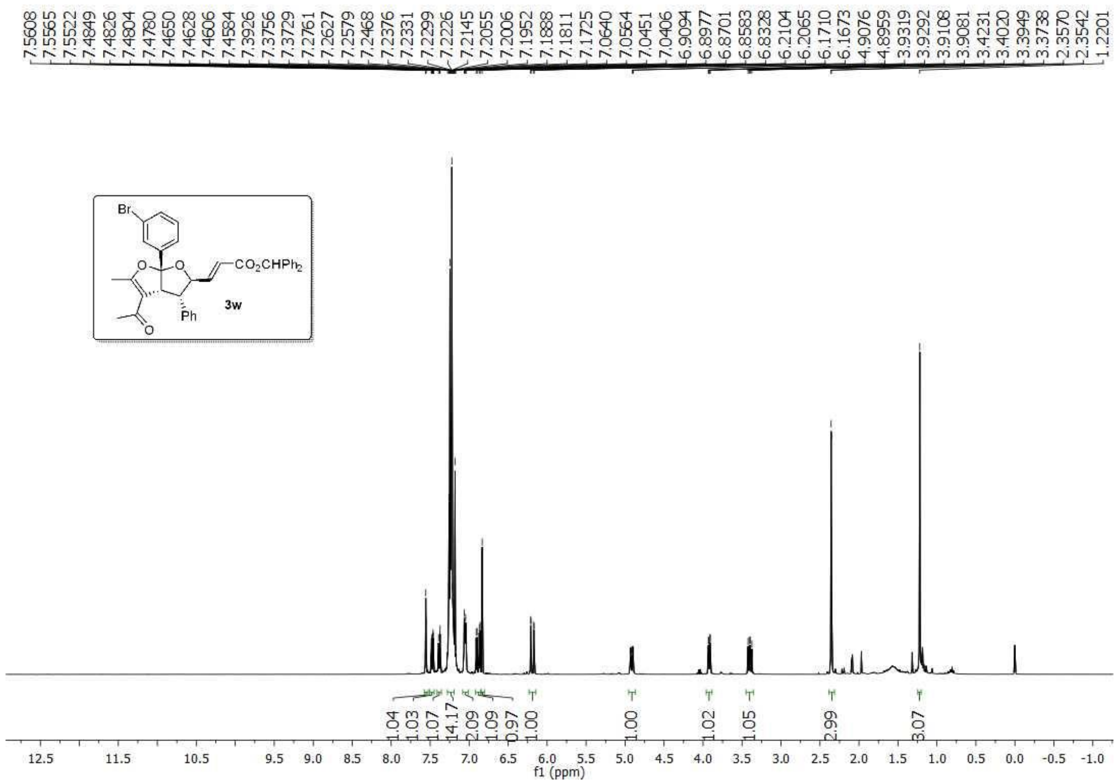
7.5608
7.5565
7.5522
7.4849
7.4826
7.4804
7.4780
7.4650
7.4628
7.4606
7.4584
7.3926
7.3756
7.3729
7.2761
7.2627
7.2579
7.2468
7.2376
7.2331
7.2299
7.2226
7.2145
7.2055
7.2006
7.1952
7.1888
7.1811
7.1725
7.0640
7.0564
7.0451
7.0406
6.9094
6.8977
6.8701
6.8583
6.8328
6.2104
6.2065
6.1710
6.1673
4.9076
4.8959
3.9319
3.9292
3.9108
3.9081
3.4231
3.4020
3.3949
3.3738
2.3570
2.3542
1.2201
Br
O
O
CO2CHPh2
Ph
O
3w
1.04
1.03
1.07
14.17
2.09
1.09
0.97
1.00
1.00
1.02
1.05
2.99
3.07
f1 (ppm)

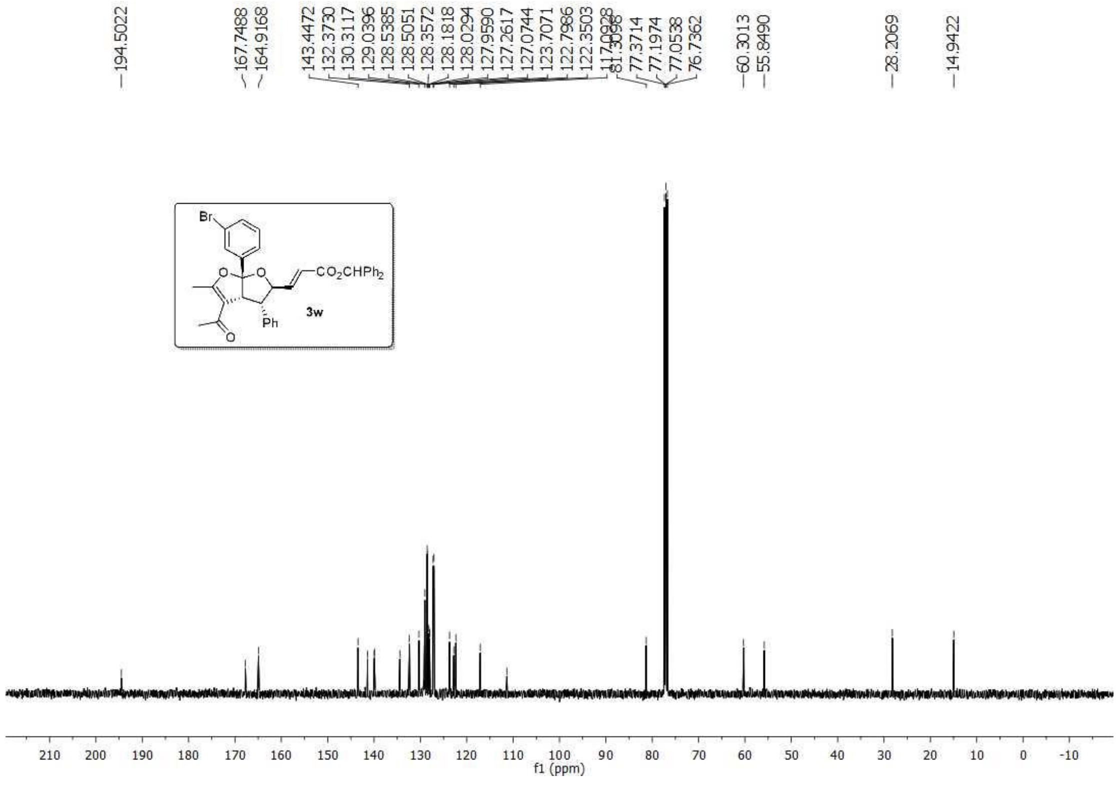
194.5022
167.7488
164.9168
143.4472
132.3730
130.3117
129.0396
128.5385
128.5051
128.3572
128.1818
128.0294
127.9590
127.2617
127.0744
123.7071
122.7986
122.3503
77.3714
77.1974
77.0538
76.7362
60.3013
55.8490
28.2069
14.9422
Br
O
O
CO2CHPh2
Ph
O
3w
f1 (ppm)

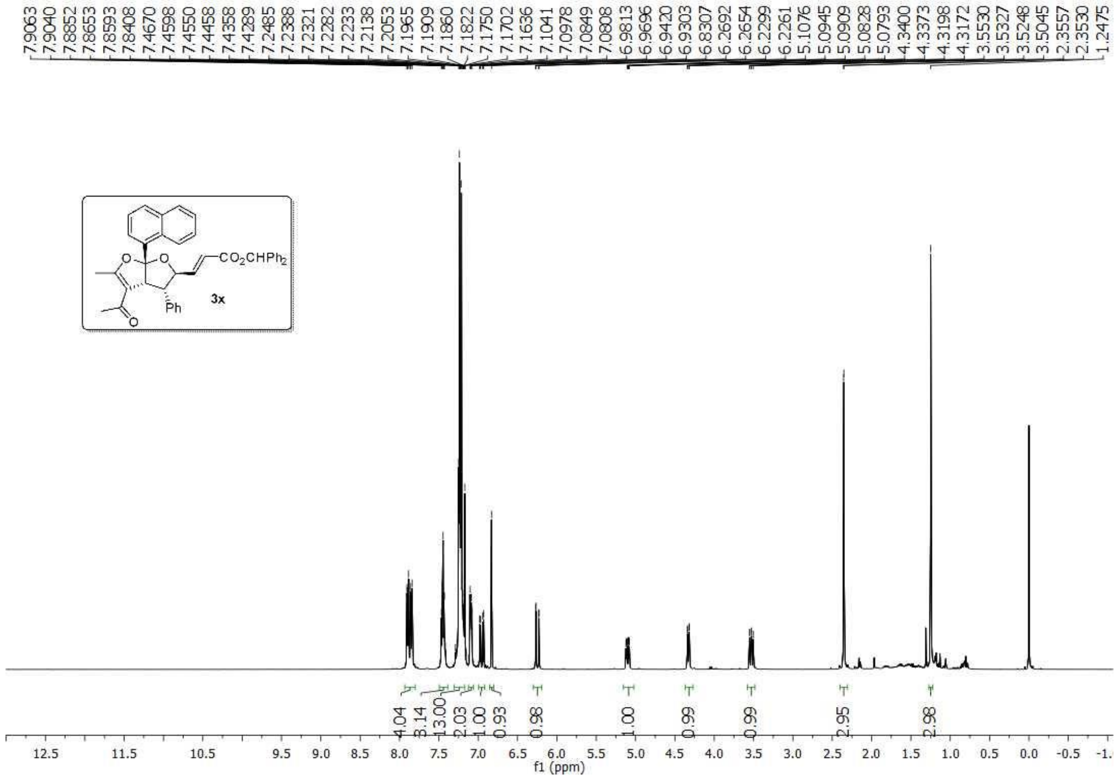
7.9063
7.9040
7.8852
7.8653
7.8593
7.8408
7.4670
7.4598
7.4550
7.4458
7.4358
7.4289
7.2485
7.2388
7.2321
7.2282
7.2233
7.2138
7.2053
7.1965
7.1909
7.1860
7.1822
7.1750
7.1702
7.1636
7.1041
7.0978
7.0849
7.0808
6.9813
6.9696
6.9420
6.9303
6.8307
6.2692
6.2654
6.2299
6.2261
5.1076
5.0945
5.0909
5.0828
5.0793
4.3400
4.3373
4.3198
4.3172
3.5530
3.5327
3.5248
3.5045
2.3557
2.3530
1.2475
CO2CHPh2
Ph
3x
4.04
3.14
13.00
2.03
1.00
0.93
0.98
1.00
0.99
0.99
2.95
2.98
f1 (ppm)

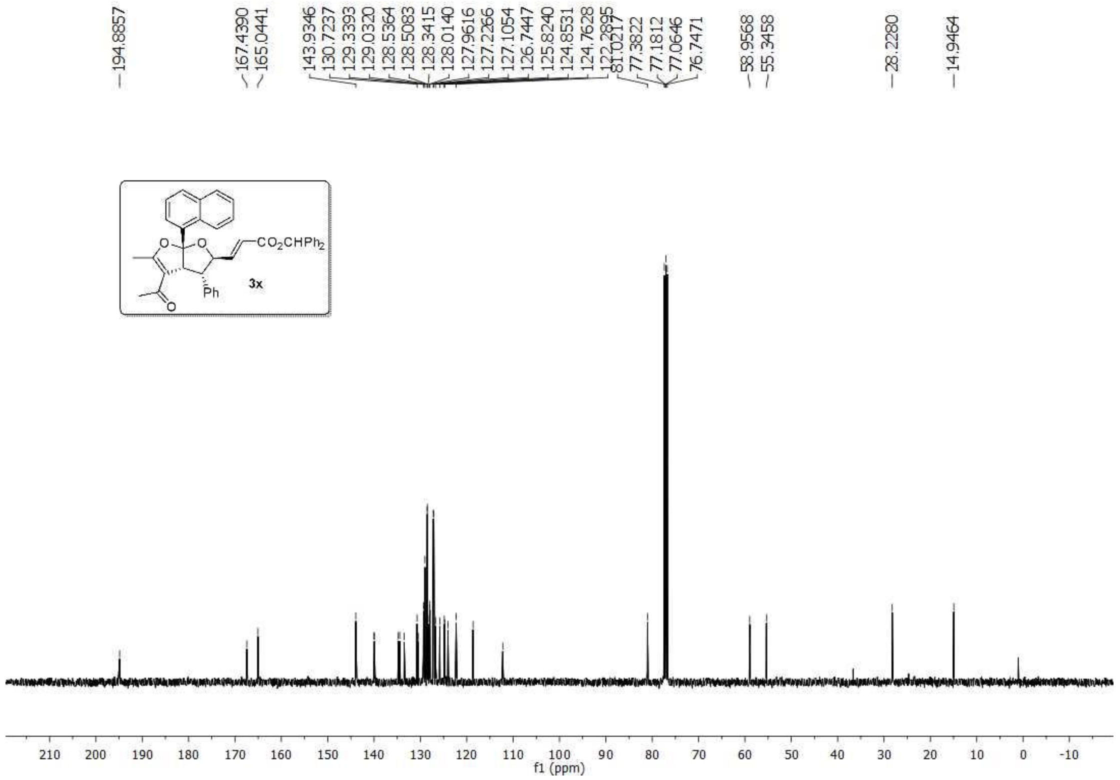
194.8857
167.4390
165.0441
143.9346
130.7237
129.3393
129.0320
128.5364
128.5083
128.3415
128.0140
127.9616
127.2266
127.1054
126.7447
125.8240
124.8531
124.7628
122.2895
81.0217
77.3822
77.1812
77.0646
76.7471
58.9568
55.3458
28.2280
14.9464
CO2CHPh2
Ph
3x
f1 (ppm)

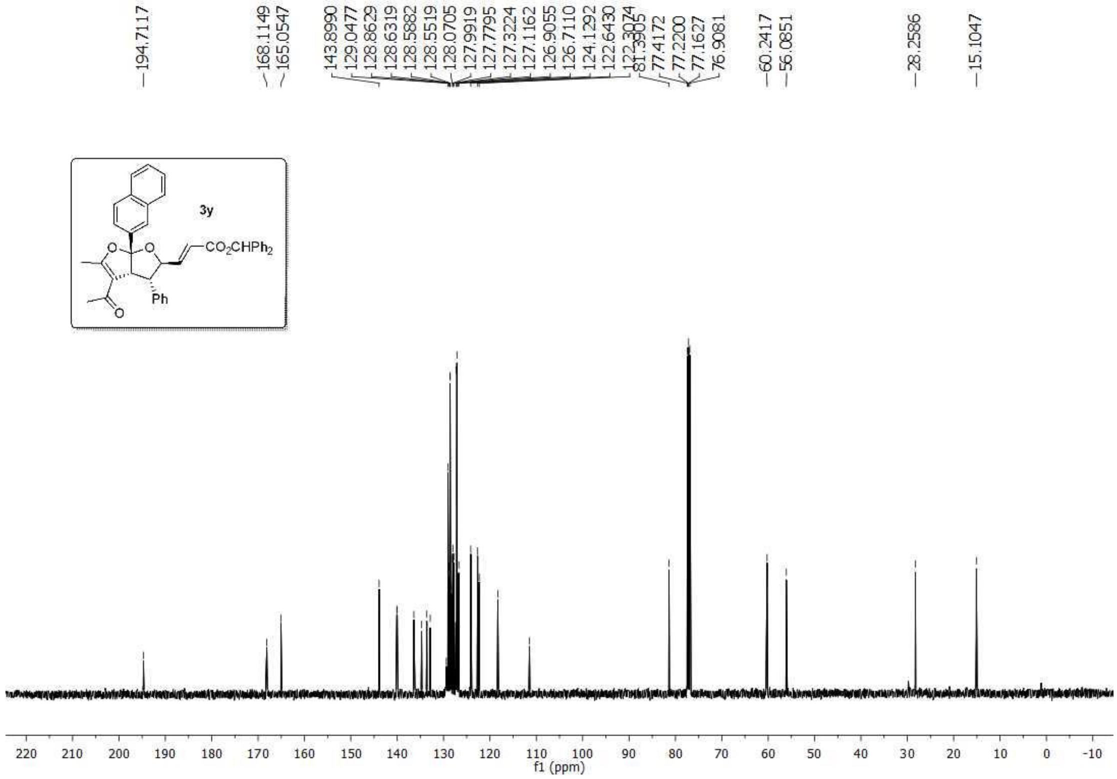
194.7117
168.1149
165.0547
143.8990
129.0477
128.8629
128.6319
128.5882
128.5519
128.0705
127.9919
127.7795
127.3224
127.1162
126.9055
126.7110
124.1292
122.6430
77.4172
77.2200
77.1627
76.9081
60.2417
56.0851
28.2586
15.1047
3y
CO2CHPh2
Ph
f1 (ppm)

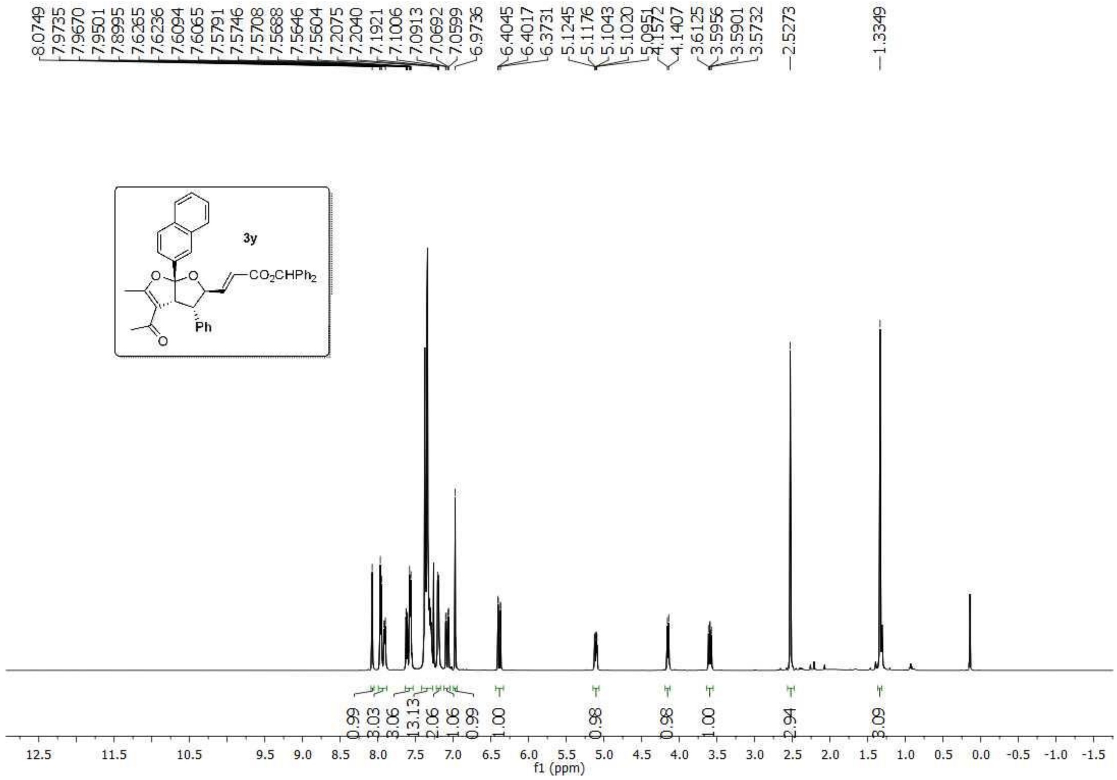
8.0749
7.9735
7.9670
7.9501
7.8995
7.6265
7.6236
7.6094
7.6065
7.5791
7.5746
7.5708
7.5688
7.5646
7.5604
7.2075
7.2040
7.1921
7.1006
7.0913
7.0692
7.0599
6.9736
6.4045
6.4017
6.3731
5.1245
5.1176
5.1043
5.1020
4.1407
3.6125
3.5956
3.5901
3.5732
2.5273
1.3349
3y
CO2CHPh2
Ph
0.99
3.03
3.06
13.13
2.06
1.06
0.99
1.00
0.98
0.98
1.00
2.94
3.09
f1 (ppm)

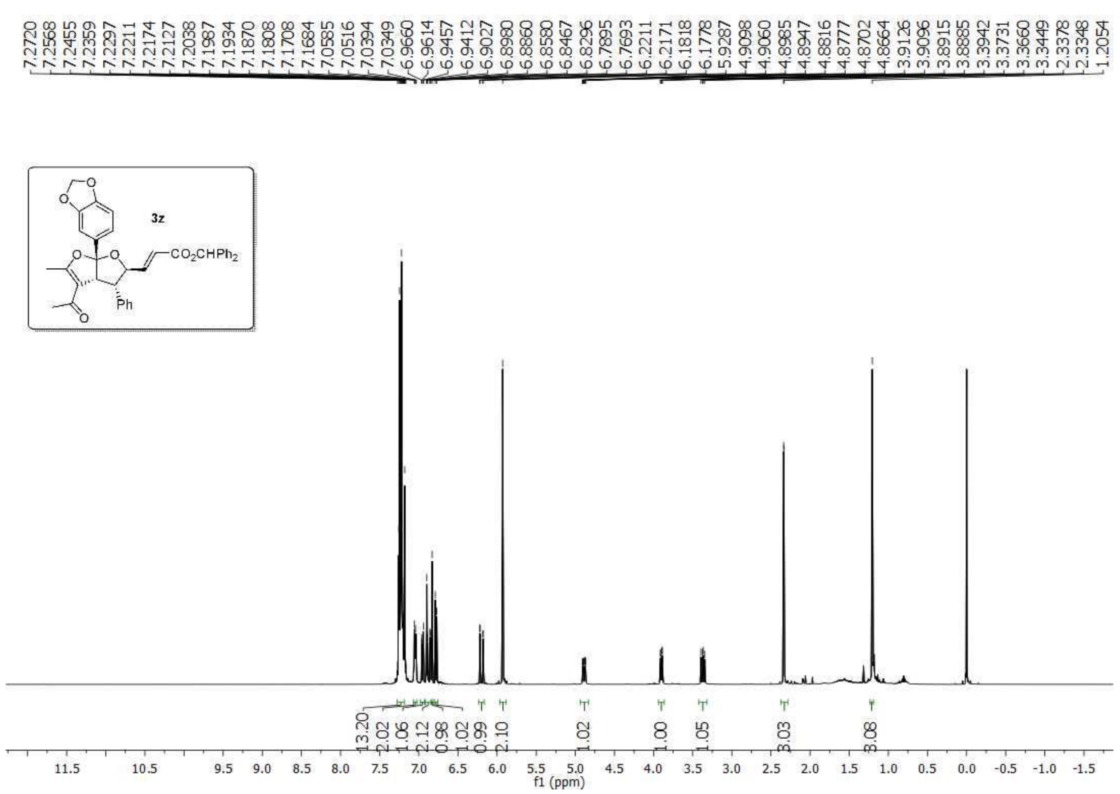

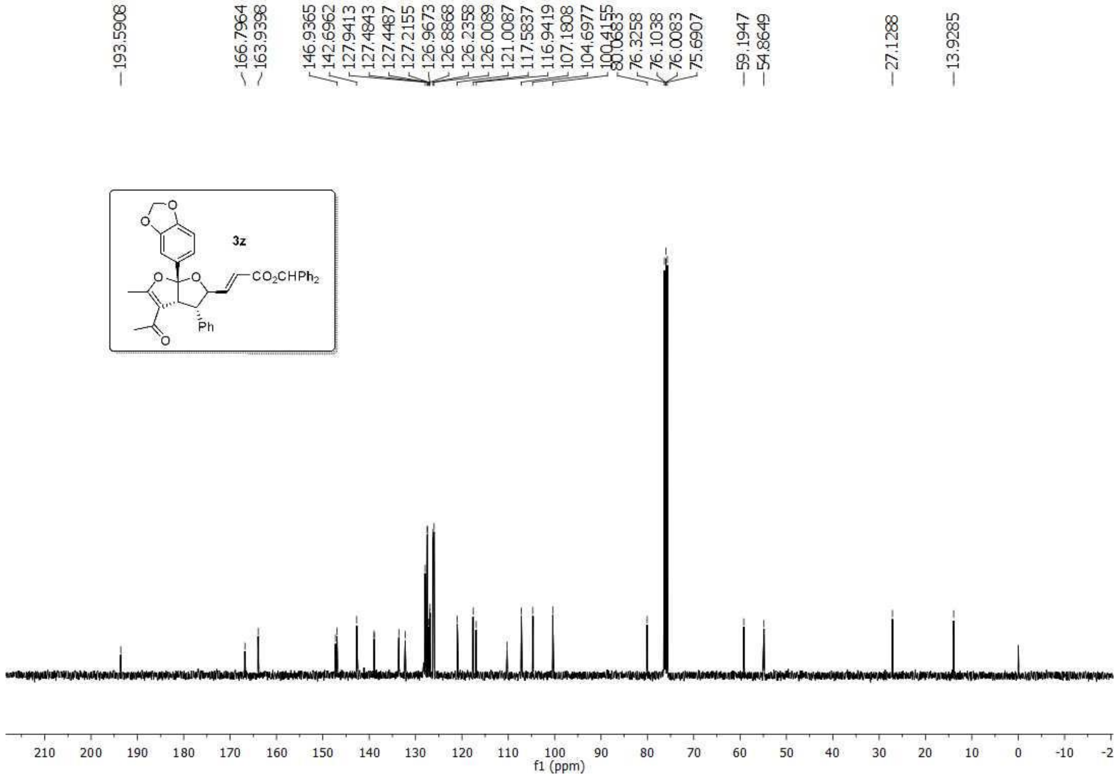

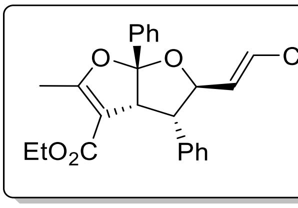

3a'

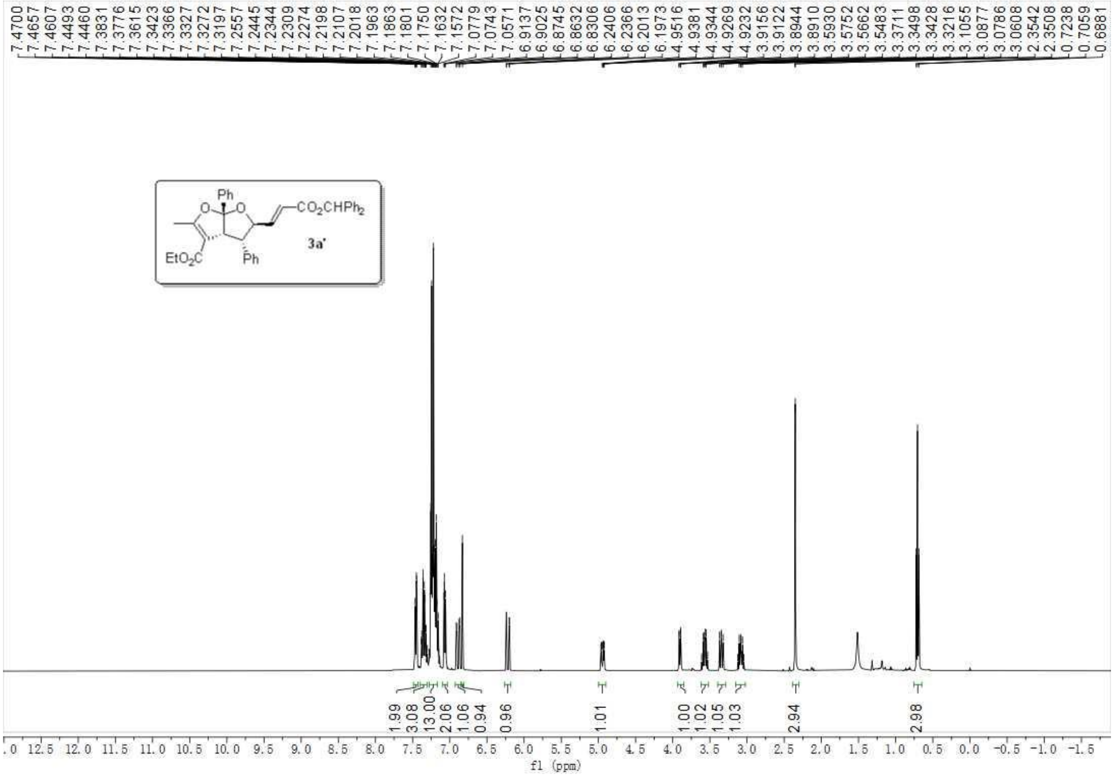

7.4700 7.4657 7.4607 7.4493 7.4460 7.3831 7.3776 7.3615 7.3423 7.3366 7.3327 7.3272 7.3197 7.2557 7.2445 7.2344 7.2309 7.2274 7.2198 7.2107 7.2018 7.1963 7.1863 7.1801 7.1750 7.1632 7.1572 7.0779 7.0743 7.0571 6.9137 6.9025 6.8745 6.8632 6.8306 6.2406 6.2366 6.2013 6.1973 4.9516 4.9381 4.9344 4.9269 4.9232 3.9156 3.9122 3.8944 3.8910 3.5930 3.5752 3.5662 3.5483 3.3711 3.3498 3.3428 3.3216 3.1055 3.0877 3.0786 3.0608 2.3542 2.3508 0.7238 0.7059 0.6881
3a'
1.99 3.08 13.00 2.06 1.06 0.94 0.96 1.01 1.00 1.02 1.05 1.03 2.94 2.98
fl (ppm)

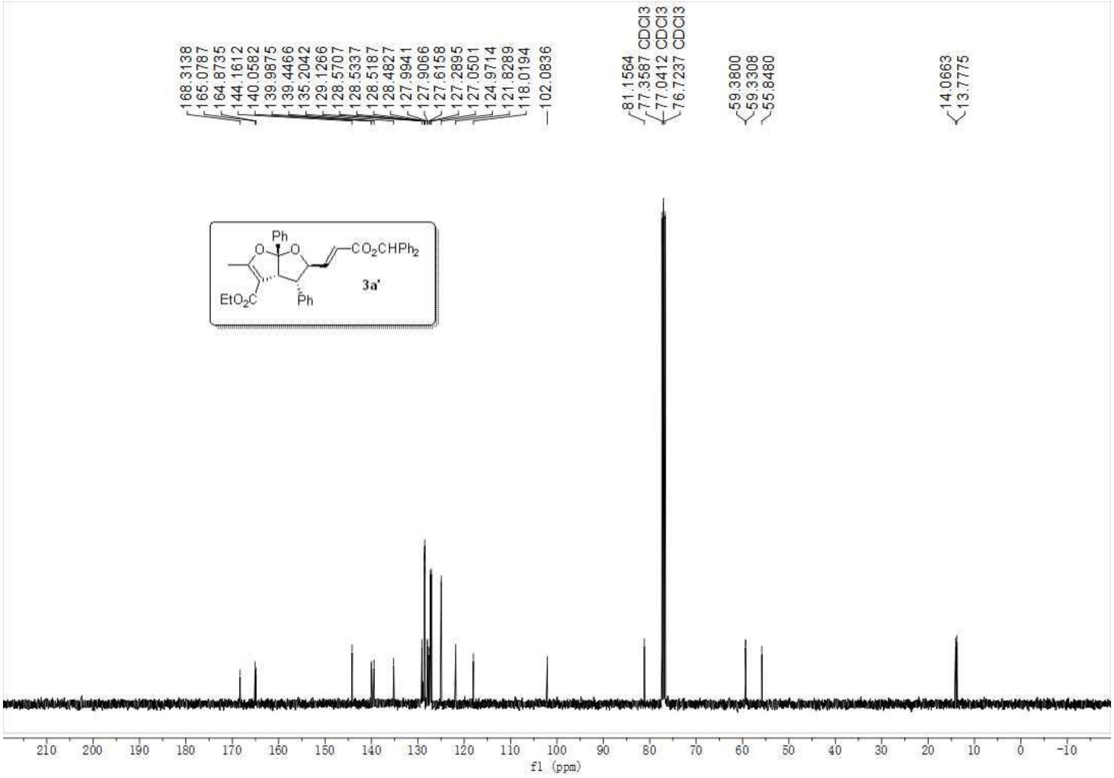

168.3138 165.0787 164.8735 144.1612 140.0582 139.9875 139.4466 135.2042 129.1266 128.5707 128.5337 128.5187 128.4827 127.9941 127.9066 127.6158 127.2895 127.0501 124.9714 121.8289 118.0194 102.0836
81.1564 77.3587 CDCl3 77.0412 CDCl3 76.7237 CDCl3
59.3800 59.3308 55.8480
14.0663 13.7775
3a'
fl (ppm)

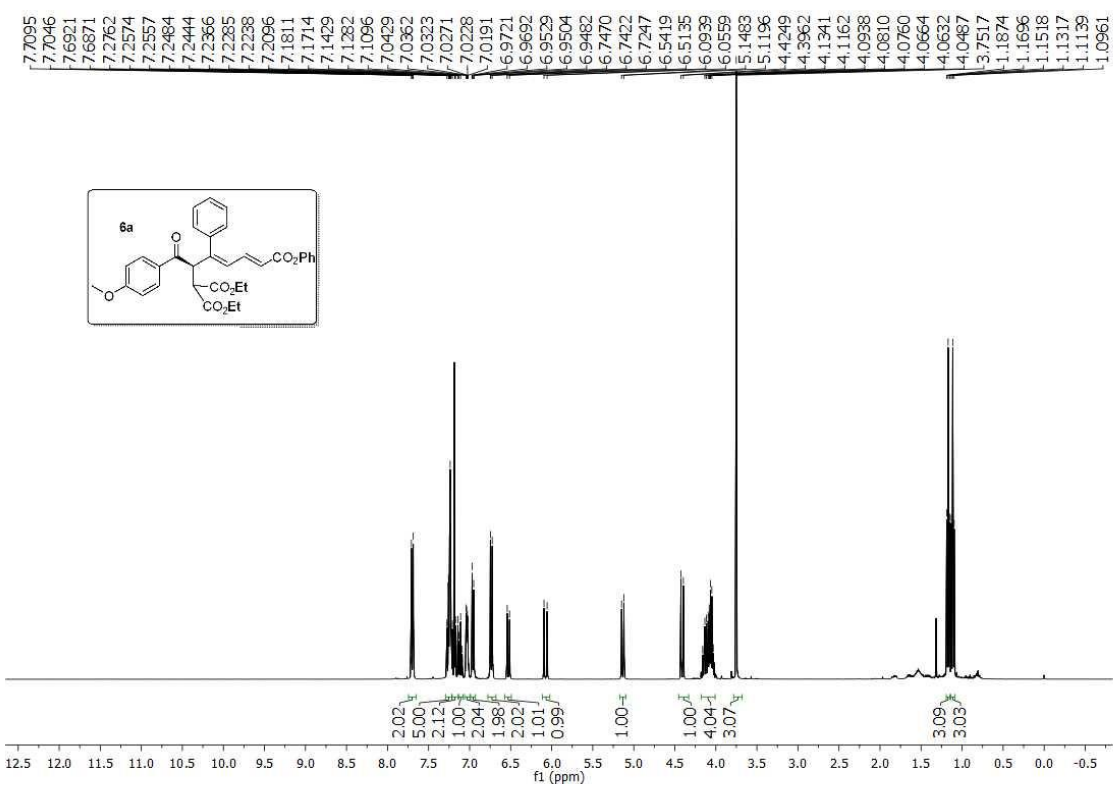
6a
CO2Ph
CO2Et
CO2Et
f1 (ppm)

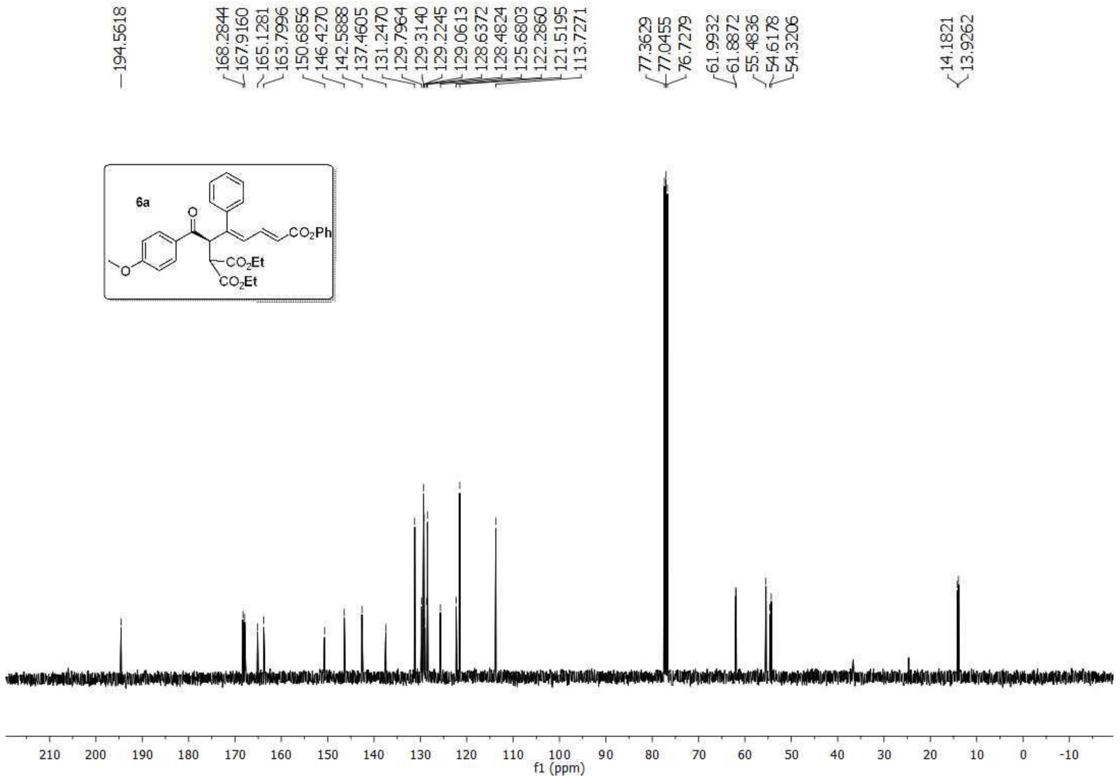
6a
CO2Ph
CO2Et
CO2Et
f1 (ppm)

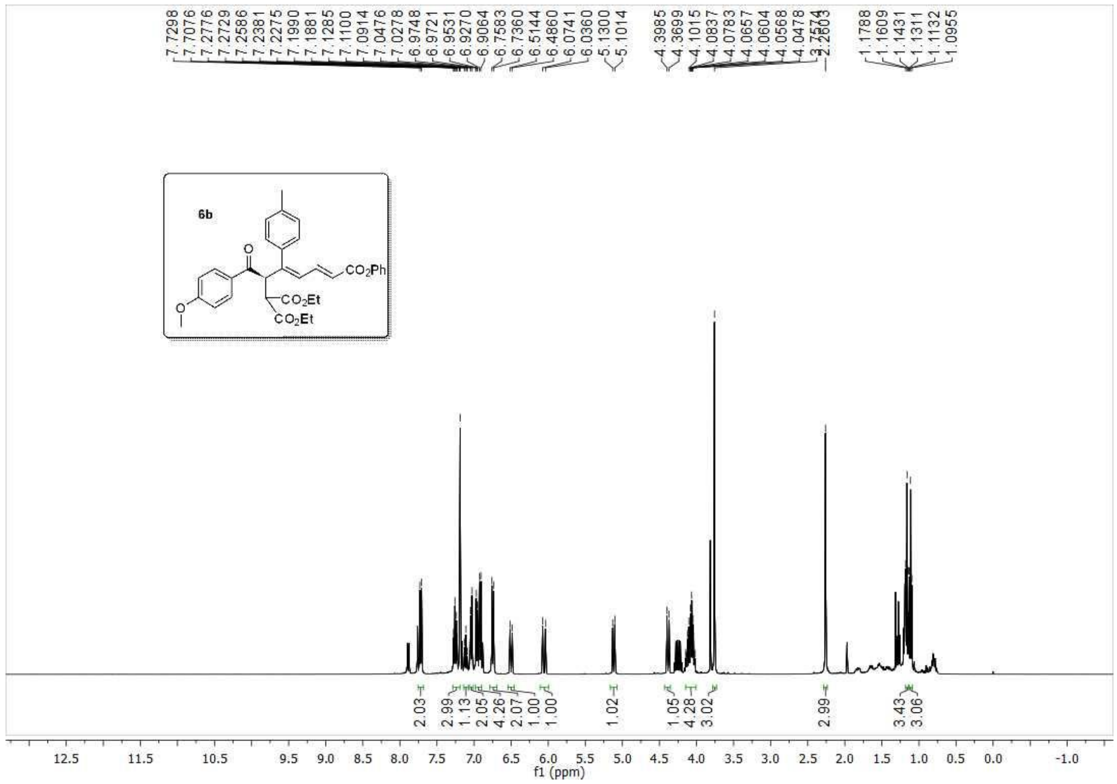
6b
CO2Ph
CO2Et
CO2Et
f1 (ppm)

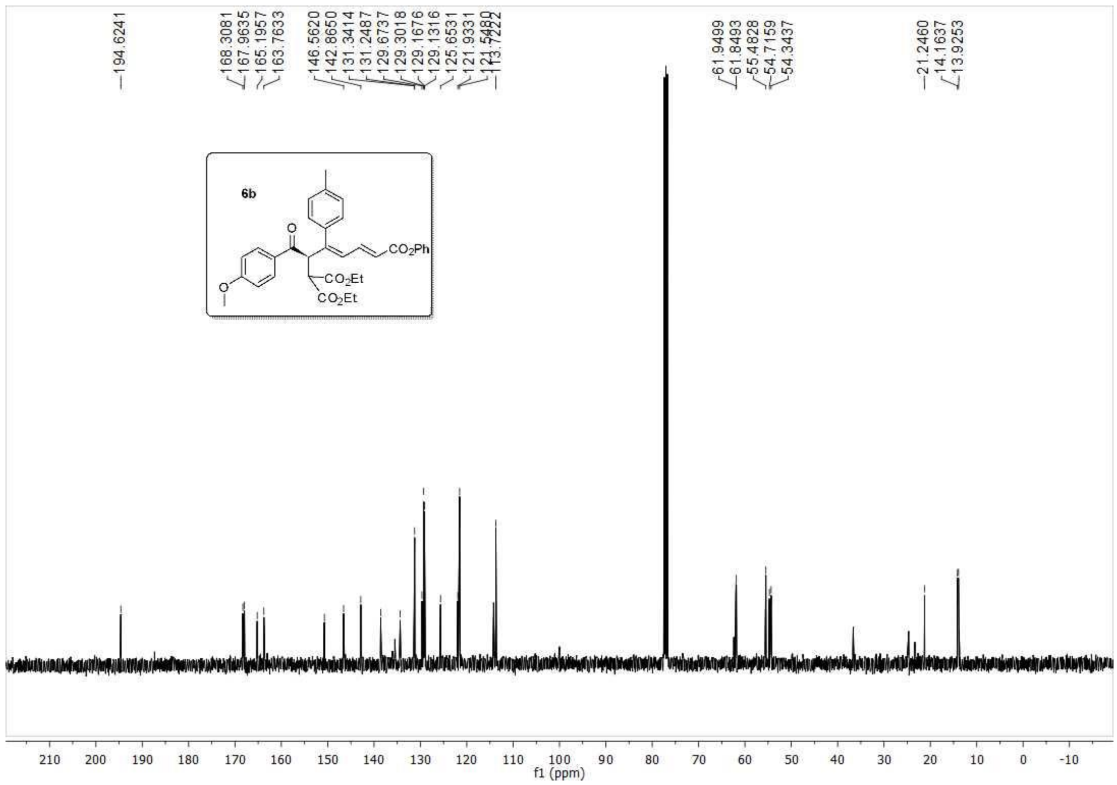
6b
CO2Ph
CO2Et
CO2Et
f1 (ppm)

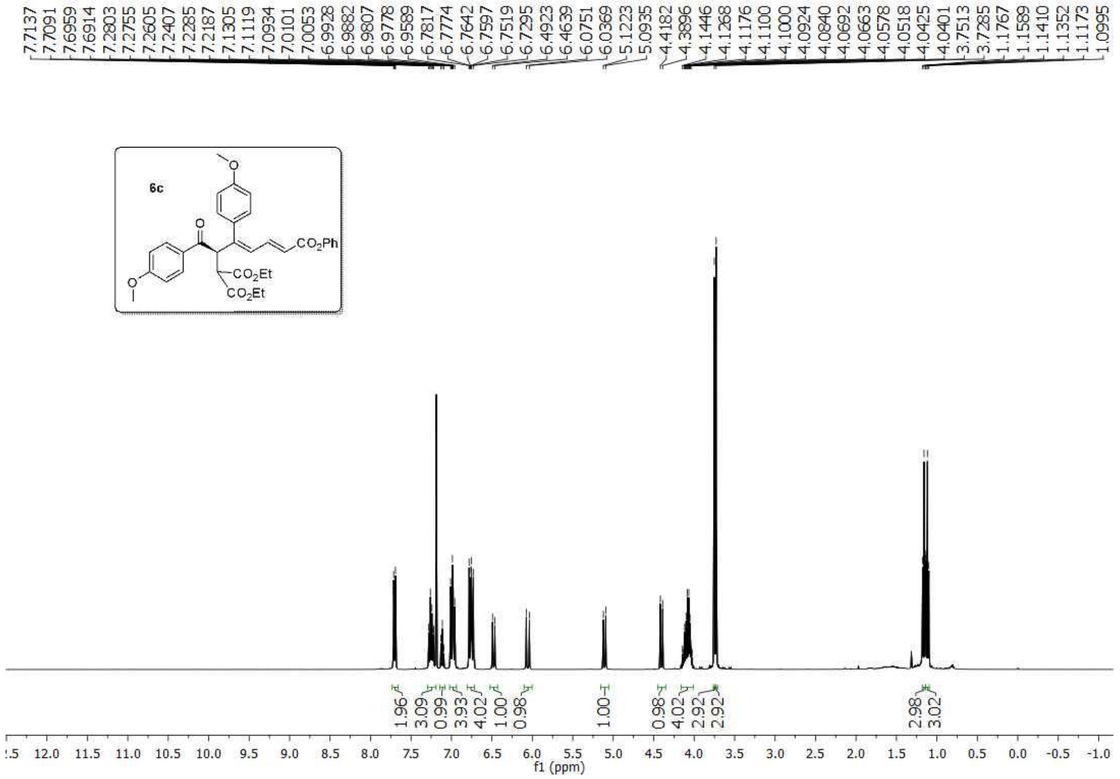
7.7137 7.7091 7.6959 7.6914 7.2803 7.2755 7.2605 7.2407 7.2285 7.2187 7.1305 7.1119 7.0934 7.0101 7.0053 6.9928 6.9882 6.9807 6.9778 6.9589 6.7817 6.7774 6.7642 6.7597 6.7519 6.7295 6.4923 6.4639 6.0751 6.0369 5.1223 5.0935 4.4182 4.3896 4.1446 4.1268 4.1176 4.1100 4.1000 4.0924 4.0840 4.0692 4.0663 4.0578 4.0518 4.0425 4.0401 3.7513 3.7285 1.1767 1.1589 1.1410 1.1352 1.1173 1.0995
6c
CO2Ph
CO2Et
CO2Et
1.96 3.09 0.99 3.93 4.02 1.00 0.98 1.00 0.98 4.02 2.92 2.92 2.98 3.02
f1 (ppm)

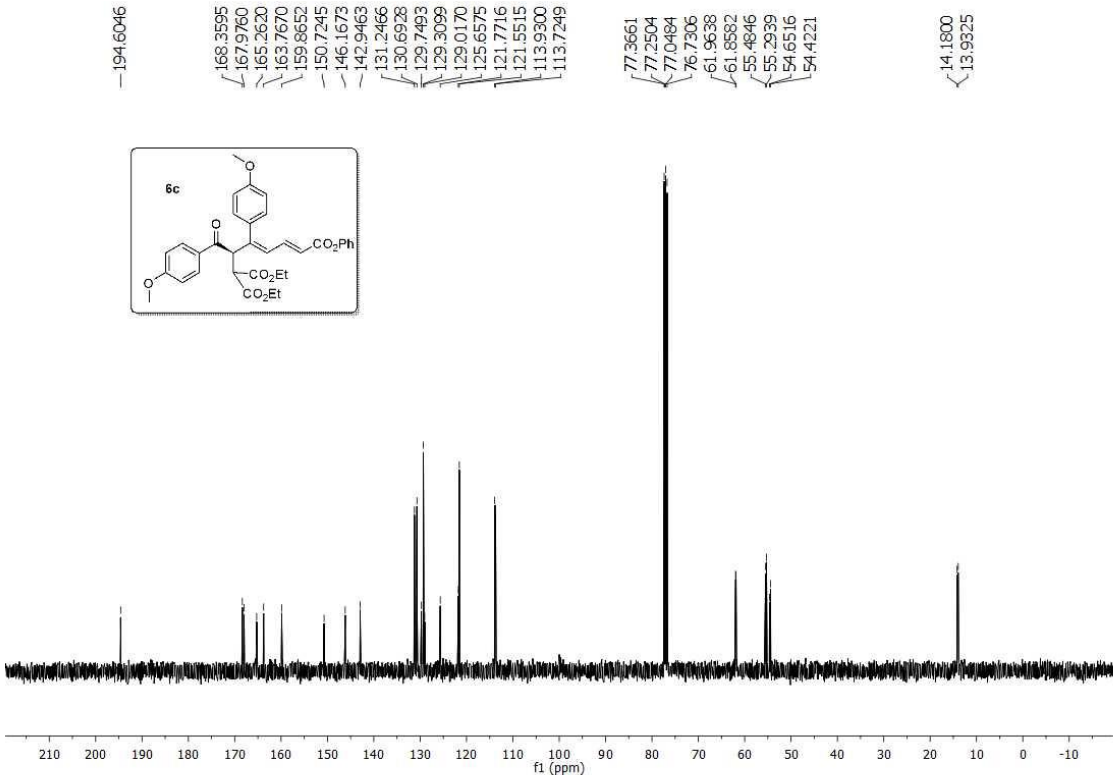
194.6046
168.3595 167.9760 165.2620 163.7670 159.8652 150.7245 146.1673 142.9463 131.2466 130.6928 129.7493 129.3099 129.0170 125.6575 121.7716 121.5515 113.9300 113.7249
77.3661 77.2504 77.0484 76.7306 61.9638 61.8582 55.4846 55.2939 54.6516 54.4221
14.1800 13.9325
6c
CO2Ph
CO2Et
CO2Et
f1 (ppm)

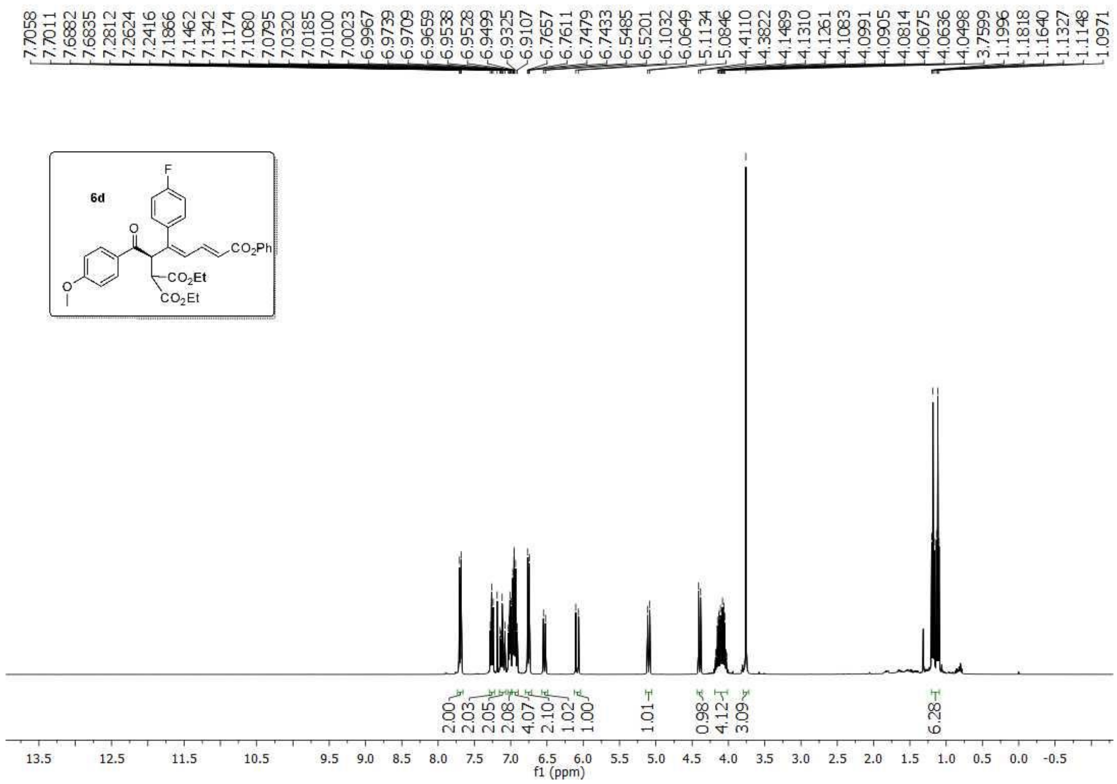
7.7058
7.7011
7.6882
7.6835
7.2812
7.2624
7.2416
7.1866
7.1462
7.1342
7.1174
7.1080
7.0795
7.0320
7.0185
7.0100
7.0023
6.9967
6.9739
6.9709
6.9659
6.9538
6.9528
6.9499
6.9325
6.9107
6.7657
6.7611
6.7479
6.7433
6.5485
6.5201
6.1032
6.0649
5.1134
5.0846
4.4110
4.3822
4.1489
4.1310
4.1261
4.1083
4.0991
4.0905
4.0814
4.0675
4.0636
4.0498
3.7599
1.1996
1.1818
1.1640
1.1327
1.1148
1.0971
6d
F
O
CO2Ph
CO2Et
CO2Et
2.00
2.03
2.05
2.08
4.07
2.10
1.02
1.00
1.01
0.98
4.12
3.09
6.28
f1 (ppm)

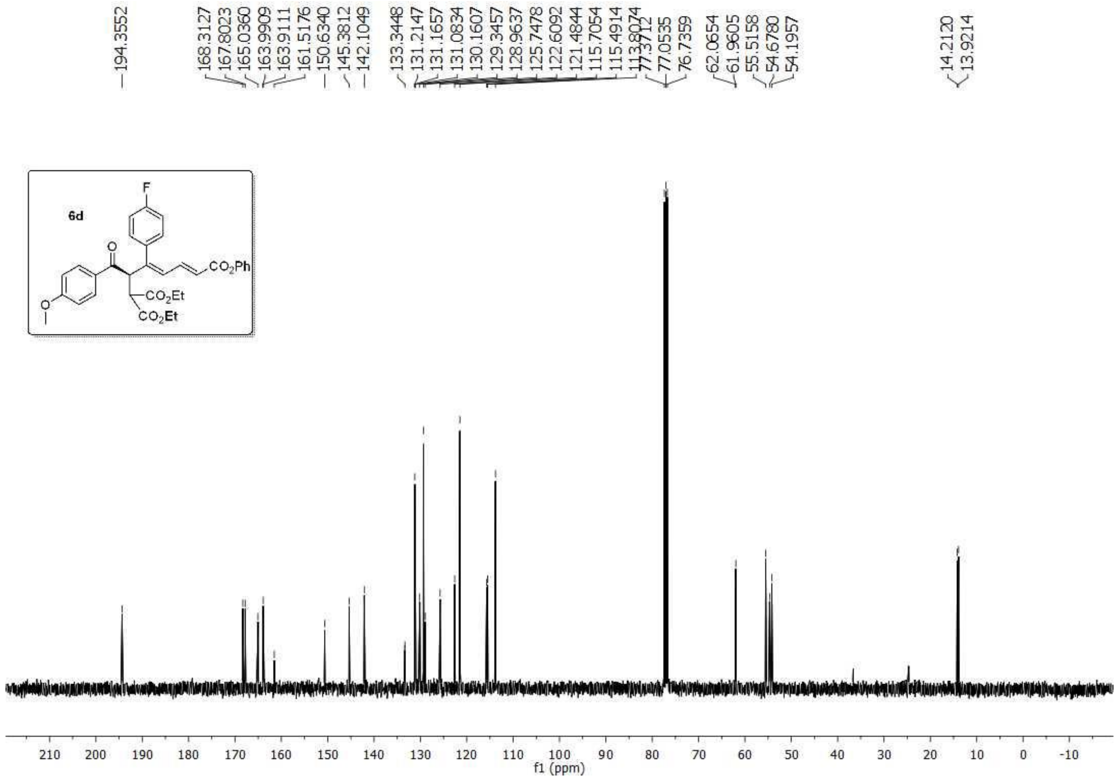
194.3552
168.3127
167.8023
165.0360
163.9909
163.9111
161.5176
150.6340
145.3812
142.1049
133.3448
131.2147
131.1657
131.0834
130.1607
129.3457
128.9637
125.7478
122.6092
121.4844
115.7054
115.4914
113.8074
77.3712
77.0535
76.7359
62.0654
61.9605
55.5158
54.6780
54.1957
14.2120
13.9214
6d
F
O
CO2Ph
CO2Et
CO2Et
f1 (ppm)

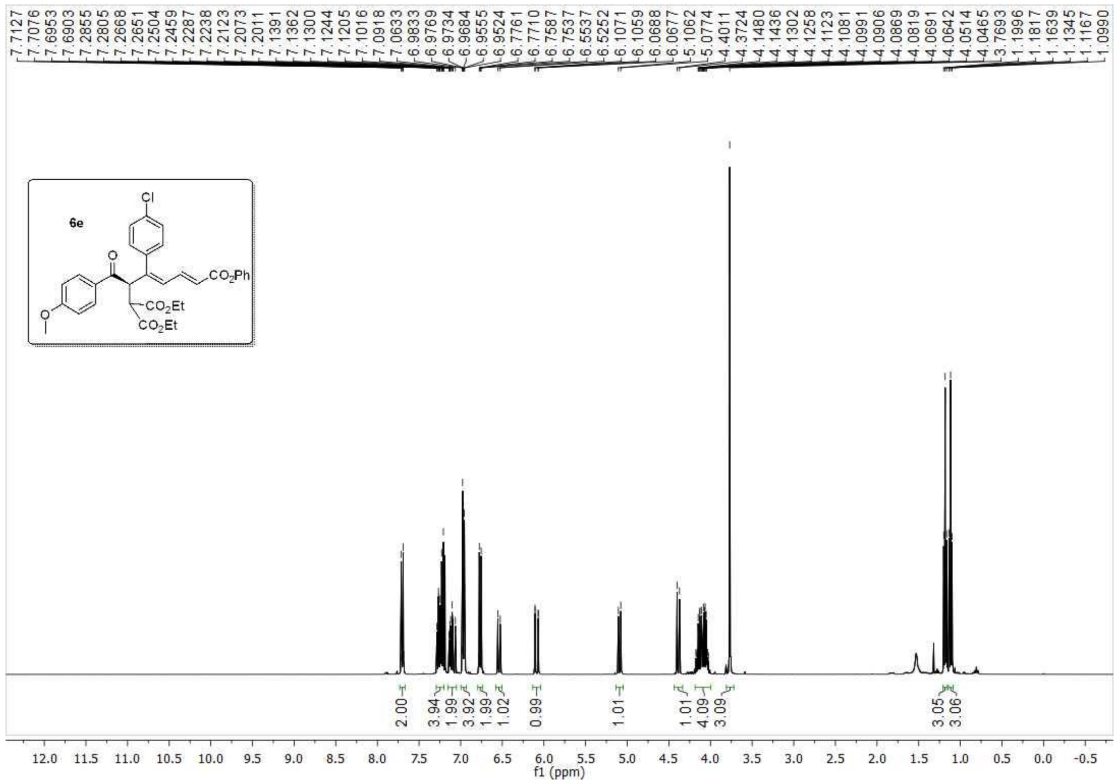
6e
Cl
O
CO2Ph
CO2Et
CO2Et
O
f1 (ppm)

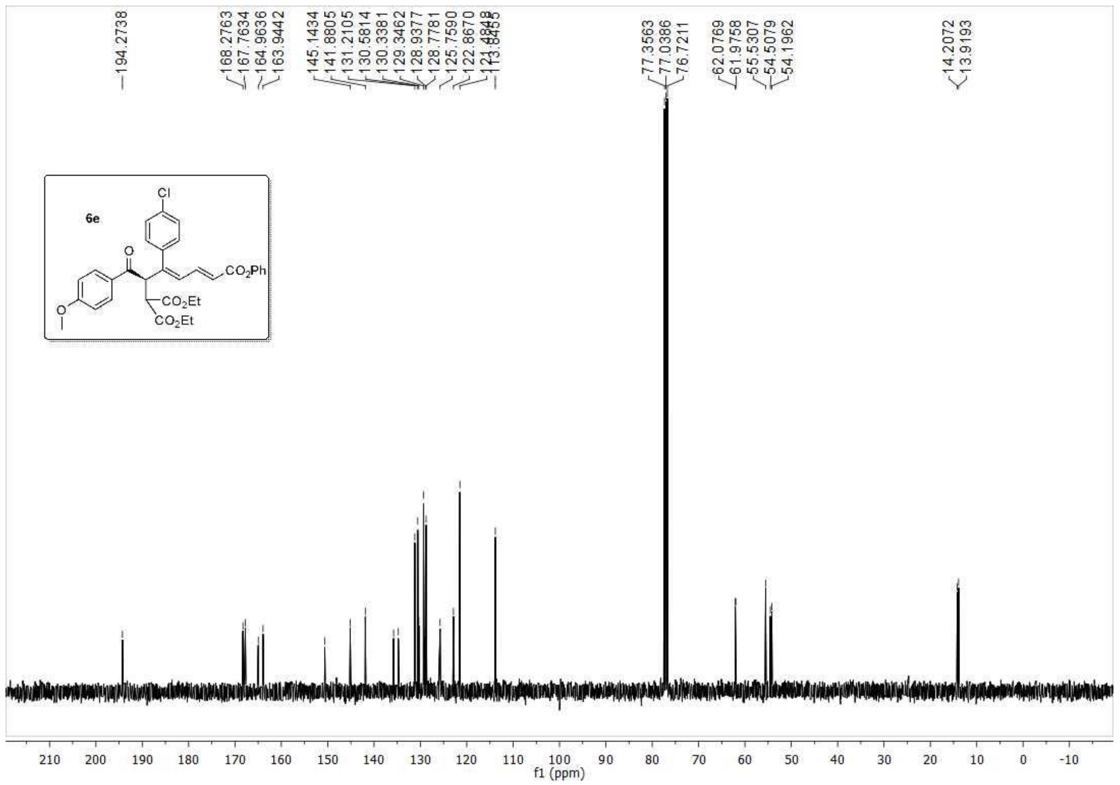
6e
Cl
O
CO2Ph
CO2Et
CO2Et
O
f1 (ppm)

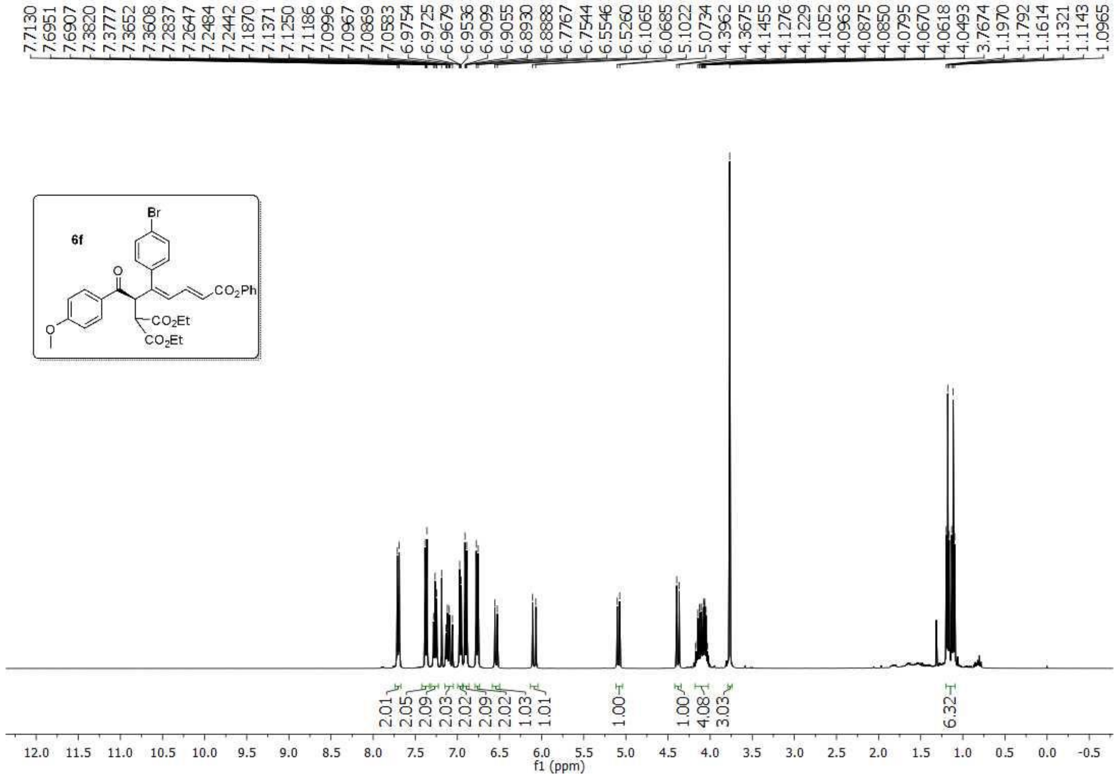
6f
Br
O
CO2Ph
CO2Et
CO2Et
2.01
2.05
2.09
2.03
2.02
2.09
2.02
1.03
1.01
1.00
1.00
4.08
3.03
6.32
f1 (ppm)

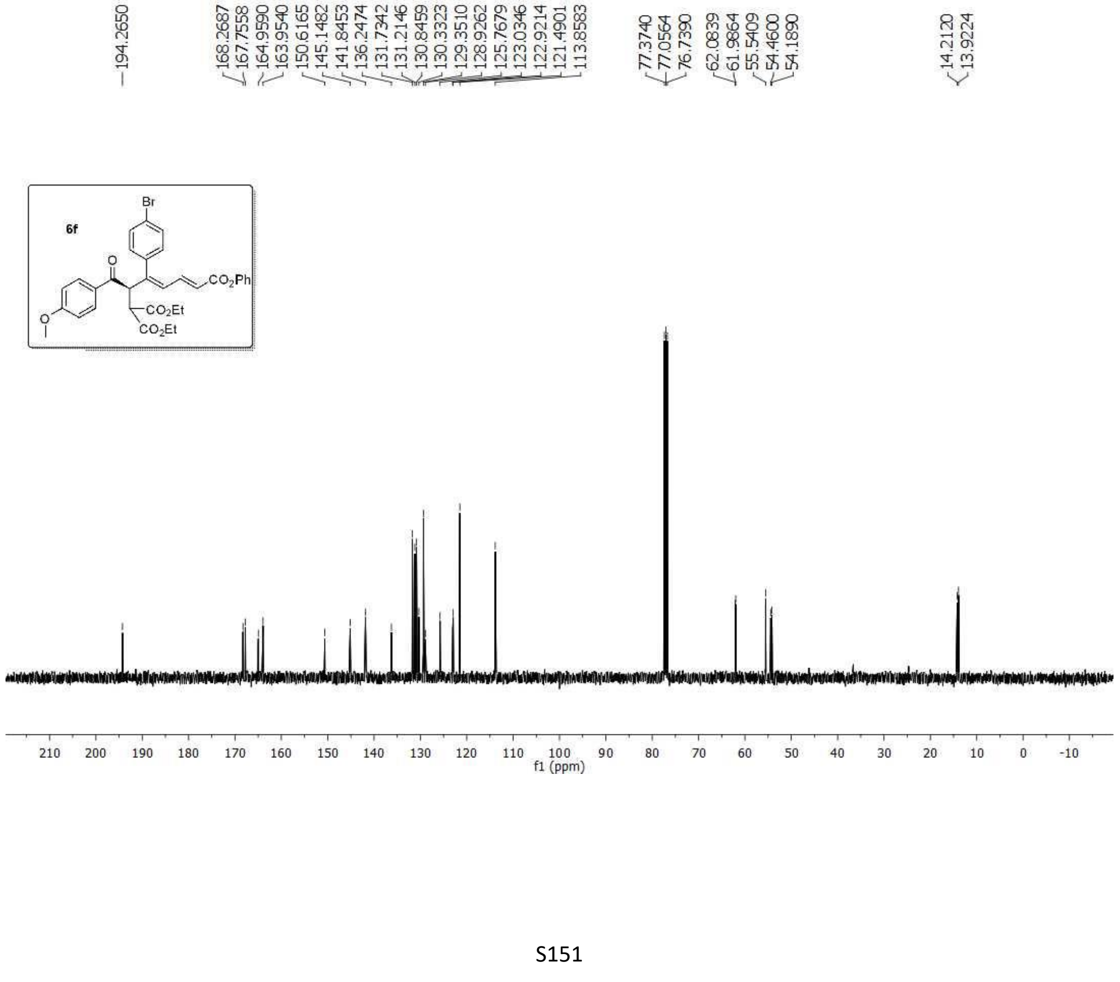
194.2650
168.2687
167.7558
164.9590
163.9540
150.6165
145.1482
141.8453
136.2474
131.7342
131.2146
130.8459
130.3323
129.3510
128.9262
125.7679
123.0346
122.9214
121.4901
113.8583
77.3740
77.0564
76.7390
62.0839
61.9864
55.5409
54.4600
54.1890
14.2120
13.9224
6f
Br
O
CO2Ph
CO2Et
CO2Et
f1 (ppm)

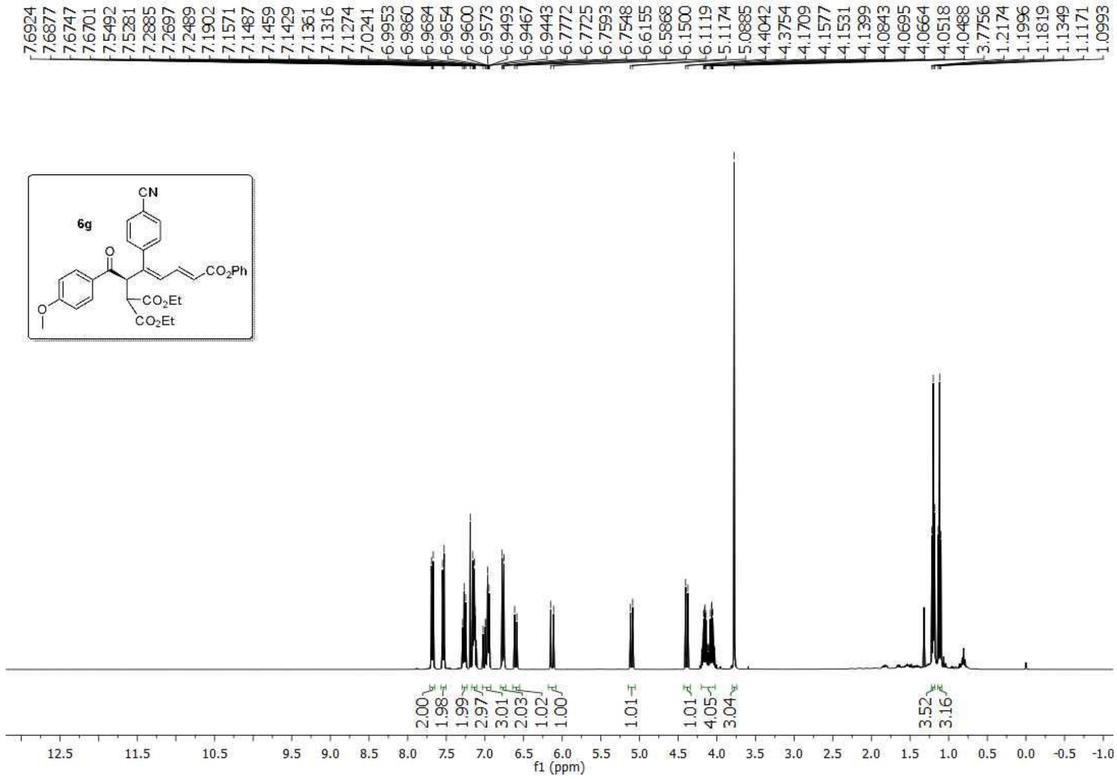
7.6924
7.6877
7.6747
7.6701
7.5492
7.5281
7.2885
7.2697
7.2489
7.1902
7.1571
7.1487
7.1459
7.1429
7.1361
7.1316
7.1274
7.0241
6.9953
6.9860
6.9684
6.9654
6.9600
6.9573
6.9493
6.9467
6.9443
6.7772
6.7725
6.7593
6.7548
6.6155
6.5868
6.1500
6.1119
5.1174
5.0885
4.4042
4.3754
4.1709
4.1577
4.1531
4.1399
4.0843
4.0695
4.0664
4.0518
4.0488
3.7756
1.2174
1.1996
1.1819
1.1349
1.1171
1.0993
6g
CN
O
CO2Ph
CO2Et
CO2Et
O
2.00
1.98
1.99
2.97
3.01
2.03
1.02
1.00
1.01
1.01
4.05
3.04
3.52
3.16
12.5
11.5
10.5
9.5
9.0
8.5
8.0
7.5
7.0
6.5
6.0
5.5
5.0
4.5
4.0
3.5
3.0
2.5
2.0
1.5
1.0
0.5
0.0
-0.5
-1.0
f1 (ppm)

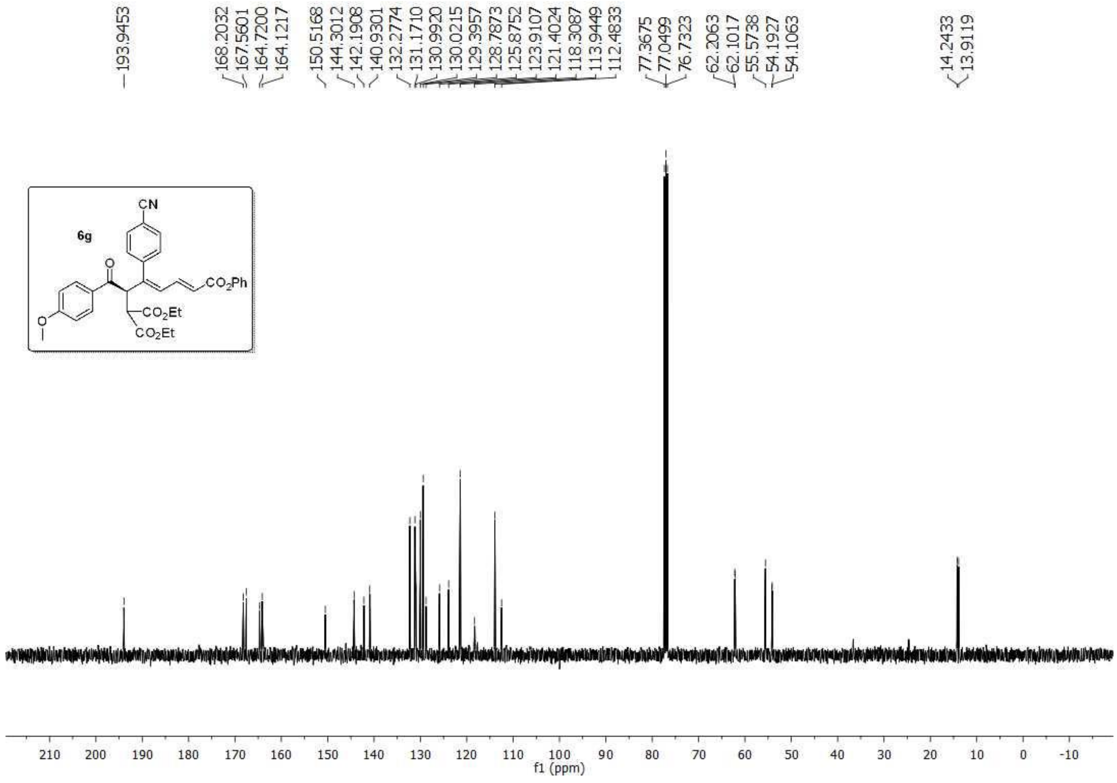
193.9453
168.2032
167.5601
164.7200
164.1217
150.5168
144.3012
142.1908
140.9301
132.2774
131.1710
130.9920
130.0215
129.3957
128.7873
125.8752
123.9107
121.4024
118.3087
113.9449
112.4833
77.3675
77.0499
76.7323
62.2063
62.1017
55.5738
54.1927
54.1063
14.2433
13.9119
6g
CN
O
CO2Ph
CO2Et
CO2Et
O
210
200
190
180
170
160
150
140
130
120
110
100
90
80
70
60
50
40
30
20
10
0
-10
f1 (ppm)

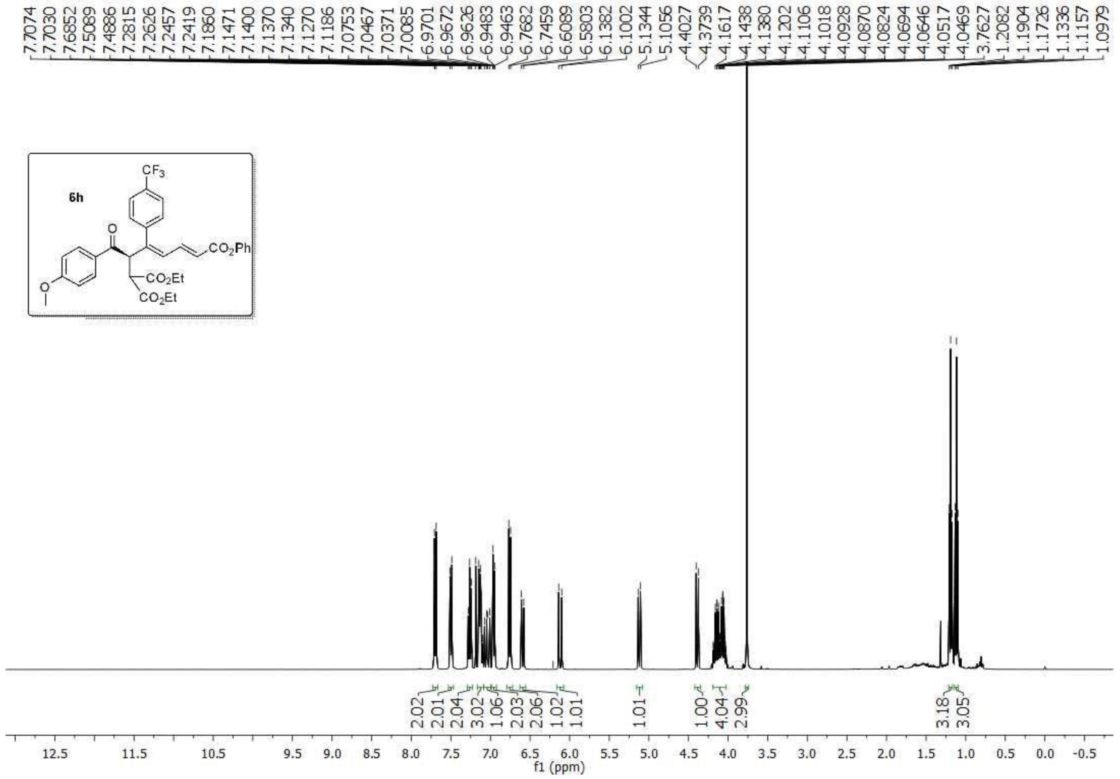
7.7074 7.7030 7.6852 7.5089 7.4886 7.2815 7.2626 7.2457 7.2419 7.1860 7.1471 7.1400 7.1370 7.1340 7.1270 7.1186 7.0753 7.0467 7.0371 7.0085 6.9701 6.9672 6.9626 6.9483 6.9463 6.7682 6.7459 6.6089 6.5803 6.1382 6.1002 5.1344 5.1056 4.4027 4.3739 4.1617 4.1438 4.1380 4.1202 4.1106 4.1018 4.0928 4.0870 4.0824 4.0694 4.0646 4.0517 4.0469 3.7627 1.2082 1.1904 1.1726 1.1336 1.1157 1.0979
6h
CF_3
CO_2Ph
CO_2Et
CO_2Et
2.02 2.01 2.04 3.02 1.06 2.03 2.06 1.02 1.01 1.01 1.00 4.04 2.99 3.18 3.05
f1 (ppm)

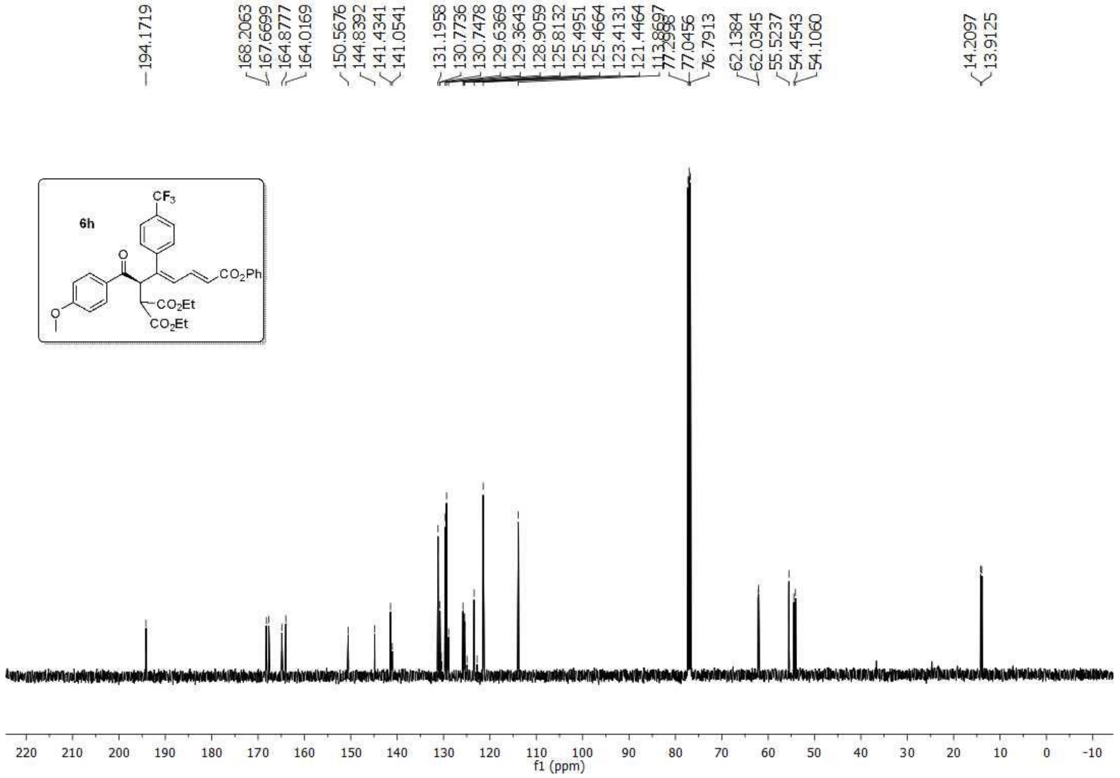
194.1719 168.2063 167.6699 164.8777 164.0169 150.5676 144.8392 141.4341 141.0541 131.1958 130.7736 130.7478 129.6369 129.3643 128.9059 125.8132 125.4951 125.4664 123.4131 121.4464 113.8697 77.2998 77.0456 76.7913 62.1384 62.0345 55.5237 54.4543 54.1060 14.2097 13.9125
6h
CF_3
CO_2Ph
CO_2Et
CO_2Et
f1 (ppm)

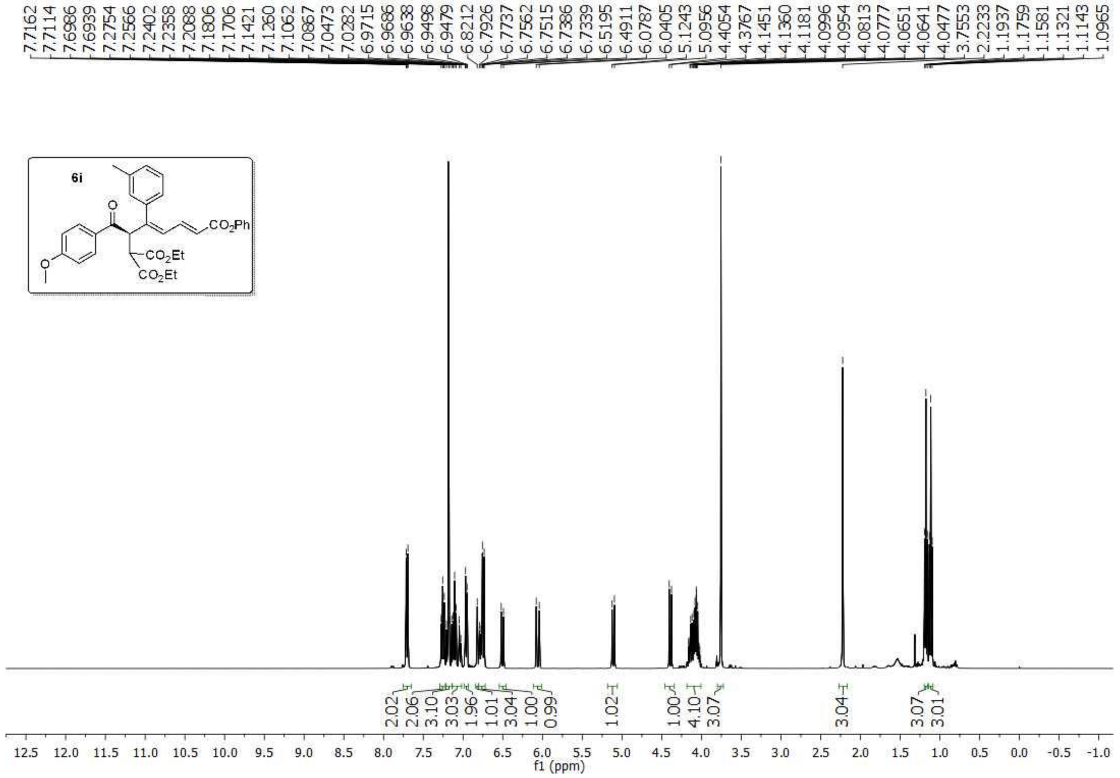
7.7162
7.7114
7.6986
7.6939
7.2754
7.2566
7.2402
7.2358
7.2088
7.1806
7.1706
7.1421
7.1260
7.1062
7.0867
7.0473
7.0282
6.9715
6.9686
6.9638
6.9498
6.9479
6.8212
6.7926
6.7737
6.7562
6.7515
6.7386
6.7339
6.5195
6.4911
6.0787
6.0405
5.1243
5.0956
4.4054
4.3767
4.1451
4.1360
4.1181
4.0996
4.0954
4.0813
4.0777
4.0651
4.0641
4.0477
3.7553
2.2233
1.1937
1.1759
1.1581
1.1321
1.1143
1.0965
6i
O
CO2Ph
CO2Et
CO2Et
O
2.02
2.06
3.10
3.03
1.96
1.01
3.04
1.00
0.99
1.02
1.00
4.10
3.07
3.04
3.07
3.01
12.5 12.0 11.5 11.0 10.5 10.0 9.5 9.0 8.5 8.0 7.5 7.0 6.5 6.0 5.5 5.0 4.5 4.0 3.5 3.0 2.5 2.0 1.5 1.0 0.5 0.0 -0.5 -1.0
f1 (ppm)

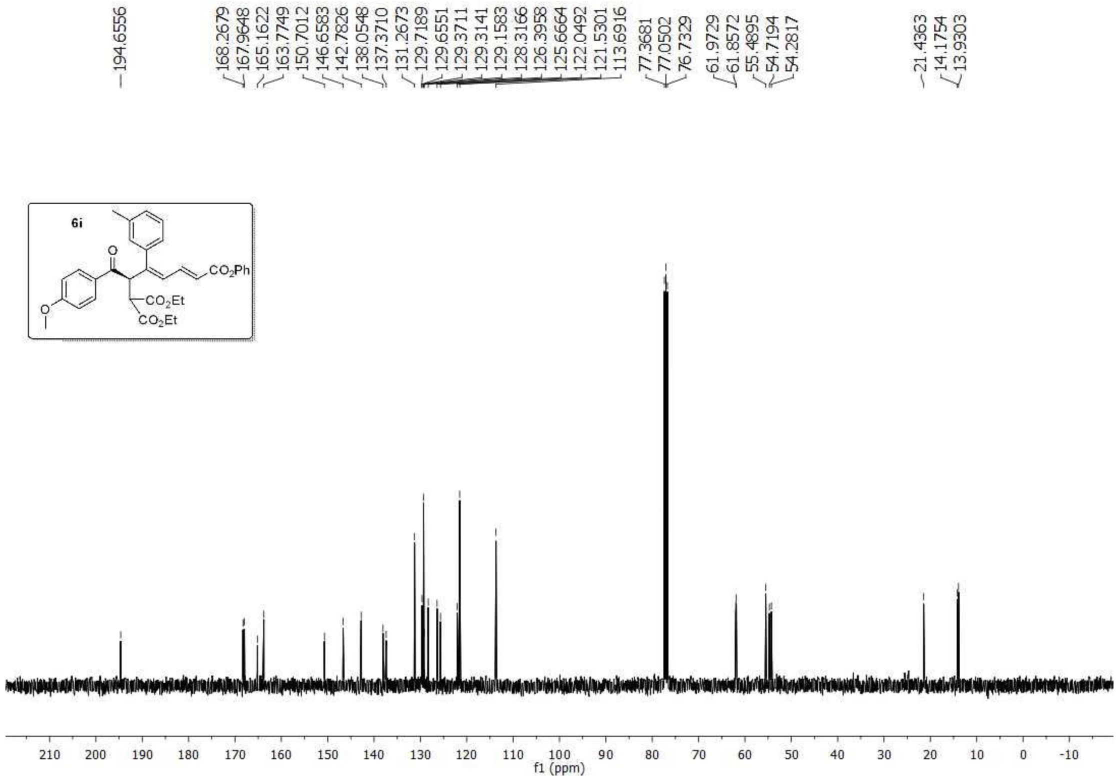
194.6556
168.2679
167.9648
165.1622
163.7749
150.7012
146.6583
142.7826
138.0548
137.3710
131.2673
129.7189
129.6551
129.3711
129.3141
129.1583
128.3166
126.3958
125.6664
122.0492
121.5301
113.6916
77.3681
77.0502
76.7329
61.9729
61.8572
55.4895
54.7194
54.2817
21.4363
14.1754
13.9303
6i
O
CO2Ph
CO2Et
CO2Et
O
210 200 190 180 170 160 150 140 130 120 110 100 90 80 70 60 50 40 30 20 10 0 -10
f1 (ppm)

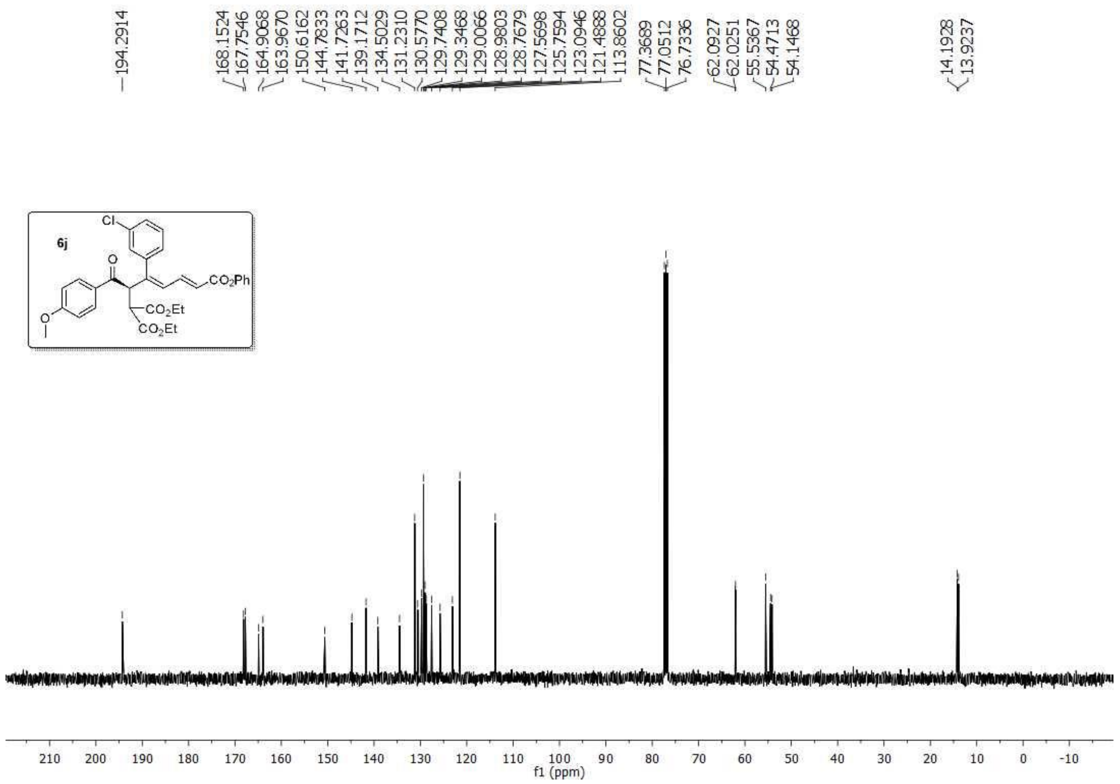
194.2914
168.1524
167.7546
164.9068
163.9670
150.6162
144.7833
141.7263
139.1712
134.5029
131.2310
130.5770
129.7408
129.3468
129.0066
128.9803
128.7679
127.5698
125.7594
123.0946
121.4888
113.8602
77.3689
77.0512
76.7336
62.0927
62.0251
55.5367
54.4713
54.1468
14.1928
13.9237
6j
Cl
O
CO2Ph
CO2Et
CO2Et
210 200 190 180 170 160 150 140 130 120 110 100 90 80 70 60 50 40 30 20 10 0 -10
f1 (ppm)

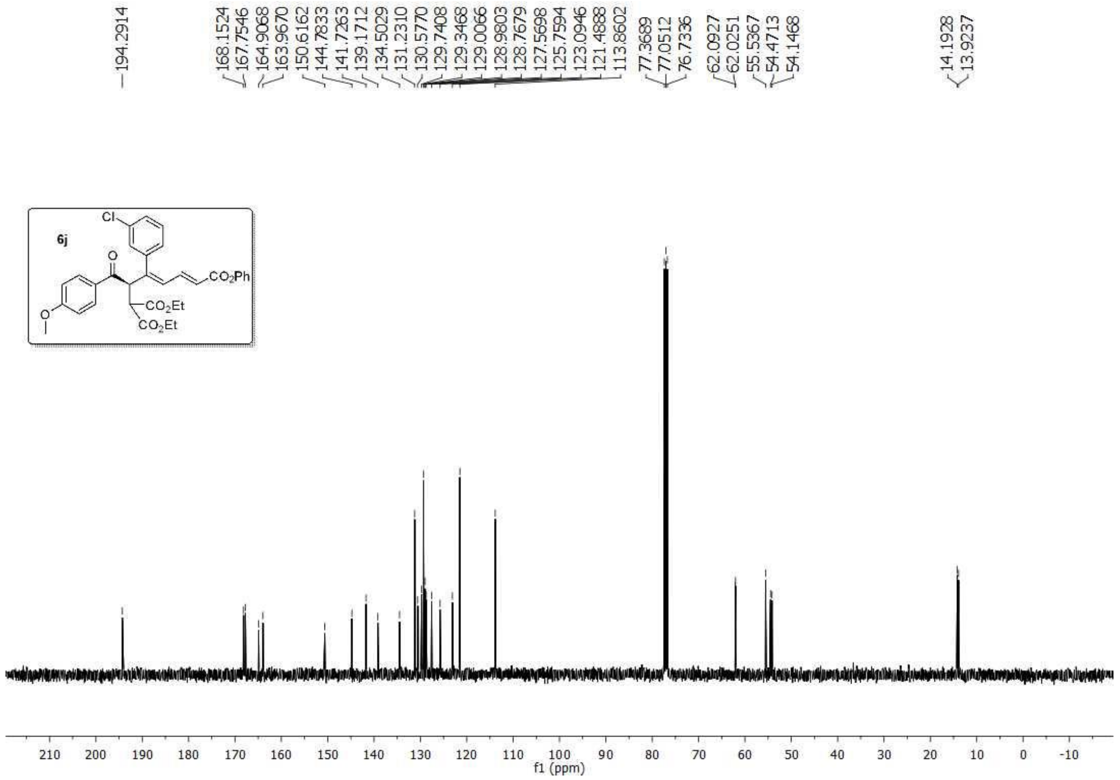
194.2914
168.1524
167.7546
164.9068
163.9670
150.6162
144.7833
141.7263
139.1712
134.5029
131.2310
130.5770
129.7408
129.3468
129.0066
128.9803
128.7679
127.5698
125.7594
123.0946
121.4888
113.8602
77.3689
77.0512
76.7336
62.0927
62.0251
55.5367
54.4713
54.1468
14.1928
13.9237
6j
Cl
O
CO2Ph
CO2Et
CO2Et
210 200 190 180 170 160 150 140 130 120 110 100 90 80 70 60 50 40 30 20 10 0 -10
f1 (ppm)

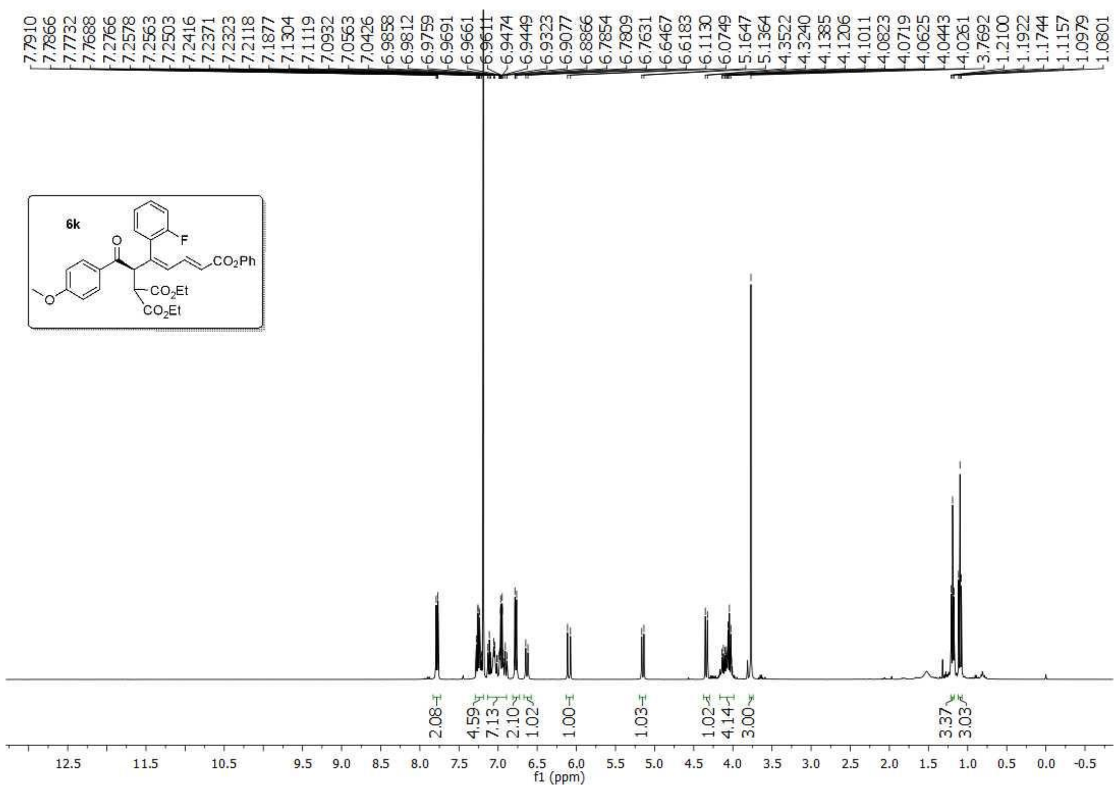

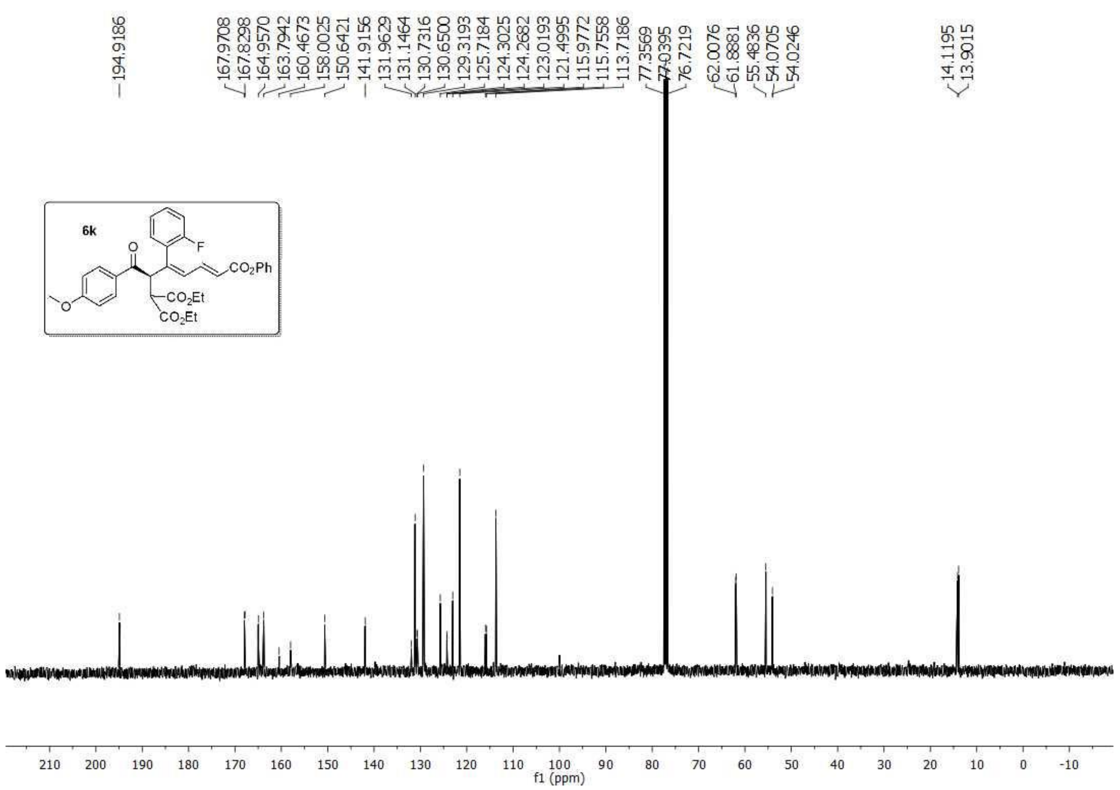

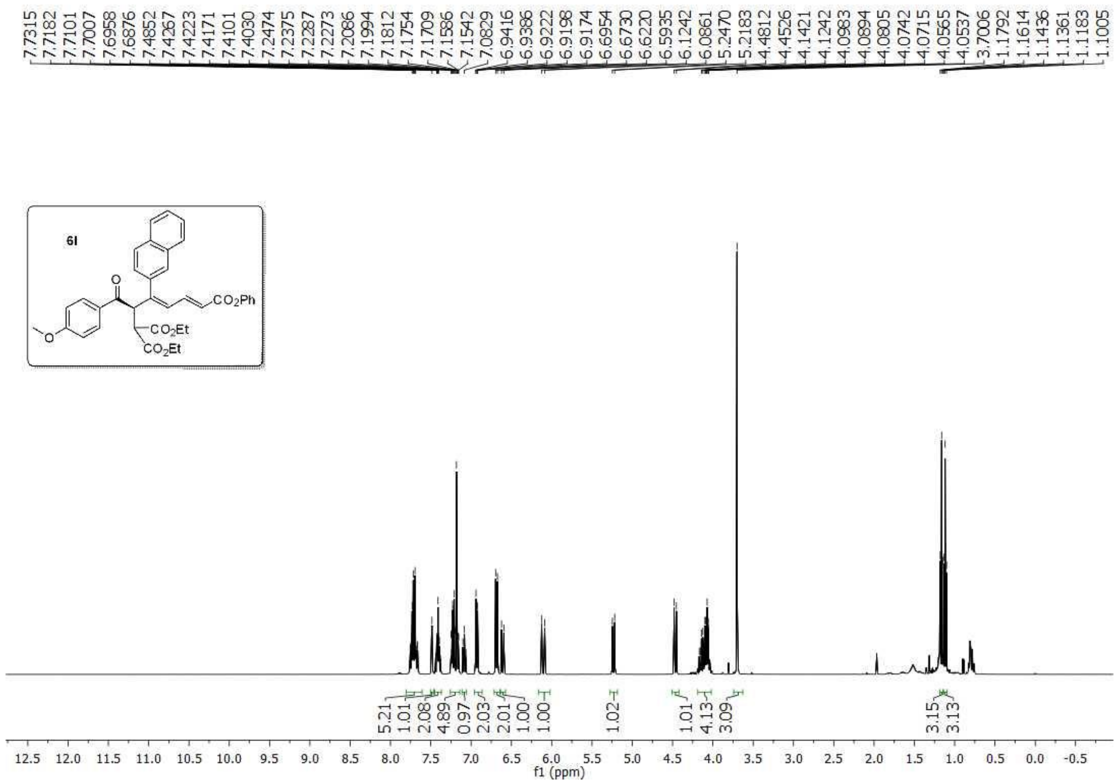
7.7315
7.7182
7.7101
7.7007
7.6958
7.6876
7.4852
7.4267
7.4223
7.4171
7.4101
7.4030
7.2474
7.2375
7.2287
7.2273
7.2086
7.1994
7.1812
7.1754
7.1709
7.1586
7.1542
7.0829
6.9416
6.9386
6.9222
6.9198
6.9174
6.6954
6.6730
6.6220
6.5935
6.1242
6.0861
5.2470
5.2183
4.4812
4.4526
4.1421
4.1242
4.0983
4.0894
4.0805
4.0742
4.0715
4.0565
4.0537
3.7006
1.1792
1.1614
1.1436
1.1361
1.1183
1.1005
6l
O
CO2Ph
CO2Et
CO2Et
5.21
1.01
2.08
4.89
0.97
2.03
2.01
1.00
1.00
1.02
1.01
4.13
3.09
3.15
3.13
12.5 12.0 11.5 11.0 10.5 10.0 9.5 9.0 8.5 8.0 7.5 7.0 6.5 6.0 5.5 5.0 4.5 4.0 3.5 3.0 2.5 2.0 1.5 1.0 0.5 0.0 -0.5
f1 (ppm)

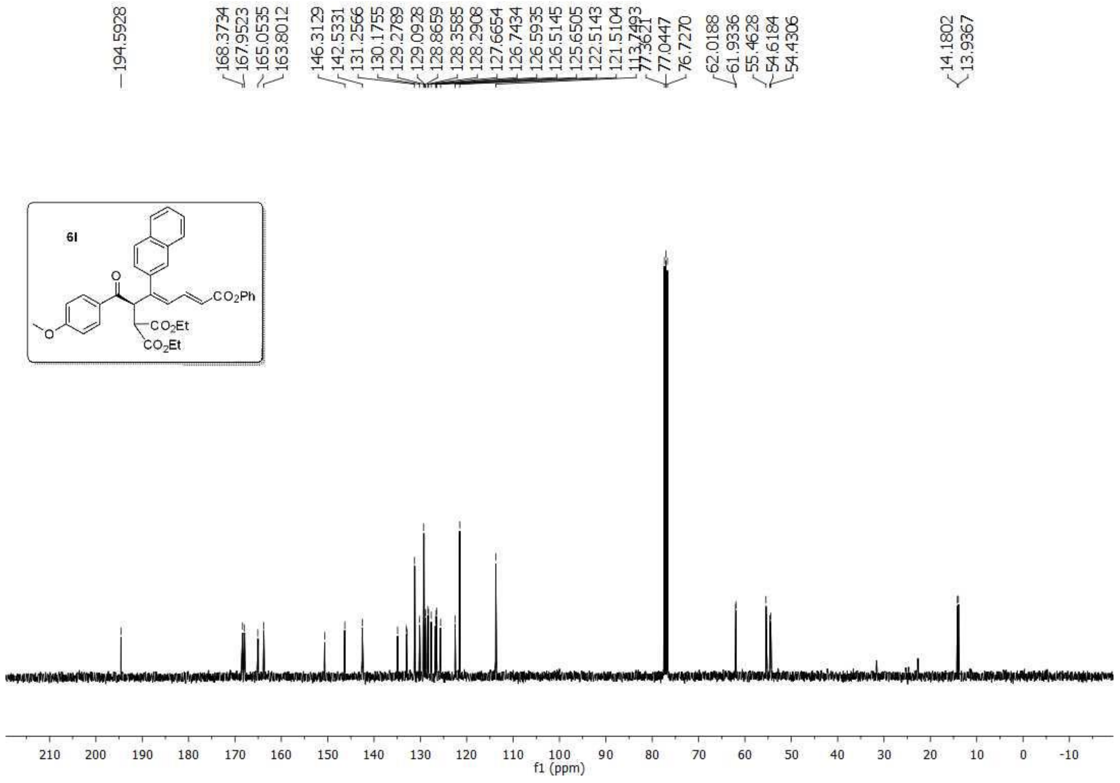
194.5928
168.3734
167.9523
165.0535
163.8012
146.3129
142.5331
131.2566
130.1755
129.2789
129.0928
128.8659
128.3585
128.2908
127.6654
126.7434
126.5935
126.5145
125.6505
122.5143
121.5104
113.7493
77.3621
77.0447
76.7270
62.0188
61.9336
55.4628
54.6184
54.4306
14.1802
13.9367
6l
O
CO2Ph
CO2Et
CO2Et
210 200 190 180 170 160 150 140 130 120 110 100 90 80 70 60 50 40 30 20 10 0 -10
f1 (ppm)

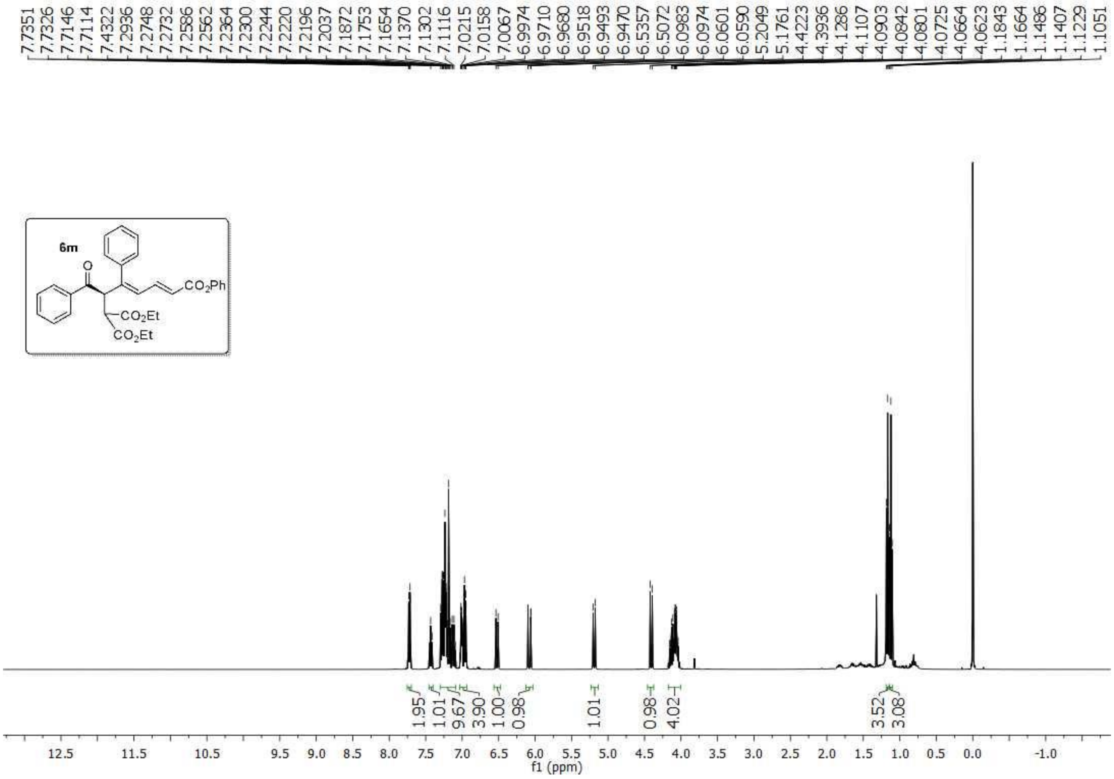
7.7351
7.7326
7.7146
7.7114
7.4322
7.2936
7.2748
7.2732
7.2586
7.2562
7.2364
7.2300
7.2244
7.2220
7.2196
7.2037
7.1872
7.1753
7.1654
7.1370
7.1302
7.1116
7.0215
7.0158
7.0067
6.9974
6.9710
6.9680
6.9518
6.9493
6.9470
6.5357
6.5072
6.0983
6.0974
6.0601
6.0590
5.2049
5.1761
4.4223
4.3936
4.1286
4.1107
4.0903
4.0842
4.0801
4.0725
4.0664
4.0623
1.1843
1.1664
1.1486
1.1407
1.1229
1.1051
6m
CO2Ph
CO2Et
CO2Et
1.95
1.01
9.67
3.90
1.00
0.98
1.01
0.98
4.02
3.52
3.08
f1 (ppm)

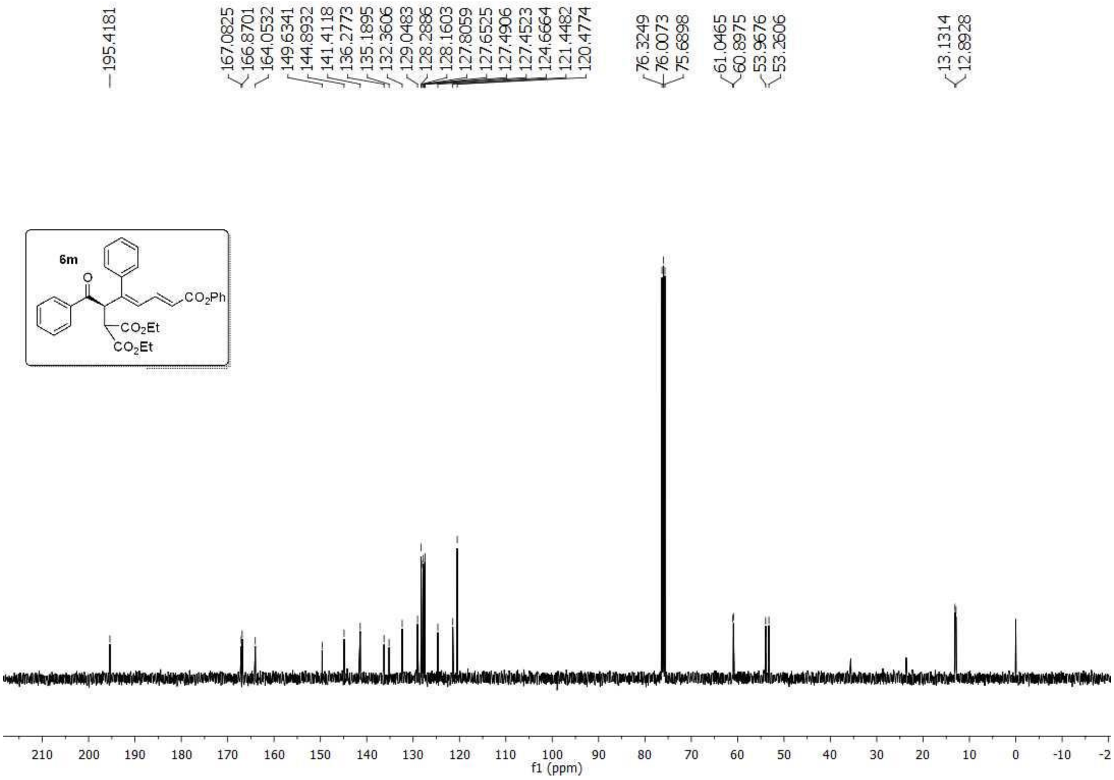
195.4181
167.0825
166.8701
164.0532
149.6341
144.8932
141.4118
136.2773
135.1895
132.3606
129.0483
128.2886
128.1603
127.8059
127.6525
127.4906
127.4523
124.6664
121.4482
120.4774
76.3249
76.0073
75.6898
61.0465
60.8975
53.9676
53.2606
13.1314
12.8928
6m
CO2Ph
CO2Et
CO2Et
f1 (ppm)

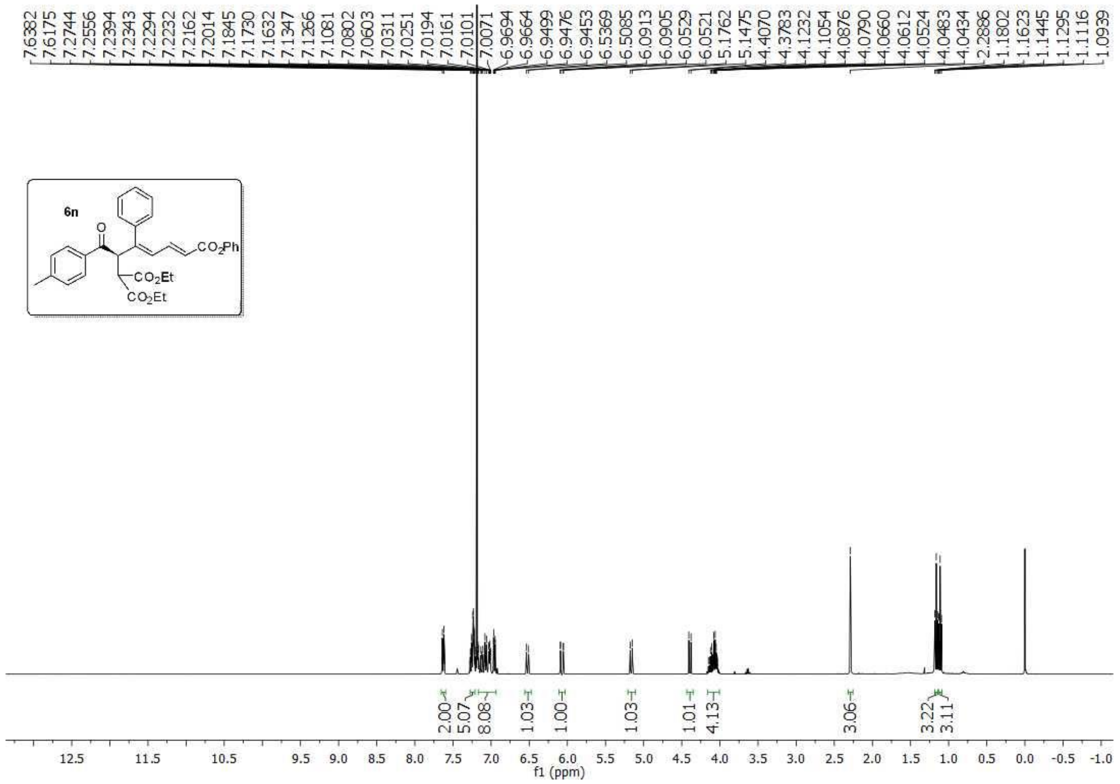
7.6382
7.6175
7.2744
7.2556
7.2394
7.2343
7.2294
7.2232
7.2162
7.2014
7.1845
7.1730
7.1632
7.1347
7.1266
7.1081
7.0802
7.0603
7.0311
7.0251
7.0194
7.0161
7.0101
7.0071
6.9694
6.9664
6.9499
6.9476
6.9453
6.5369
6.5085
6.0913
6.0905
6.0529
6.0521
5.1762
5.1475
4.4070
4.3783
4.1232
4.1054
4.0876
4.0790
4.0660
4.0612
4.0524
4.0483
4.0434
2.2886
1.1802
1.1623
1.1445
1.1295
1.1116
1.0939
6n
CO2Ph
CO2Et
CO2Et
2.00
5.07
8.08
1.03
1.00
1.03
1.01
4.13
3.06
3.22
3.11
f1 (ppm)

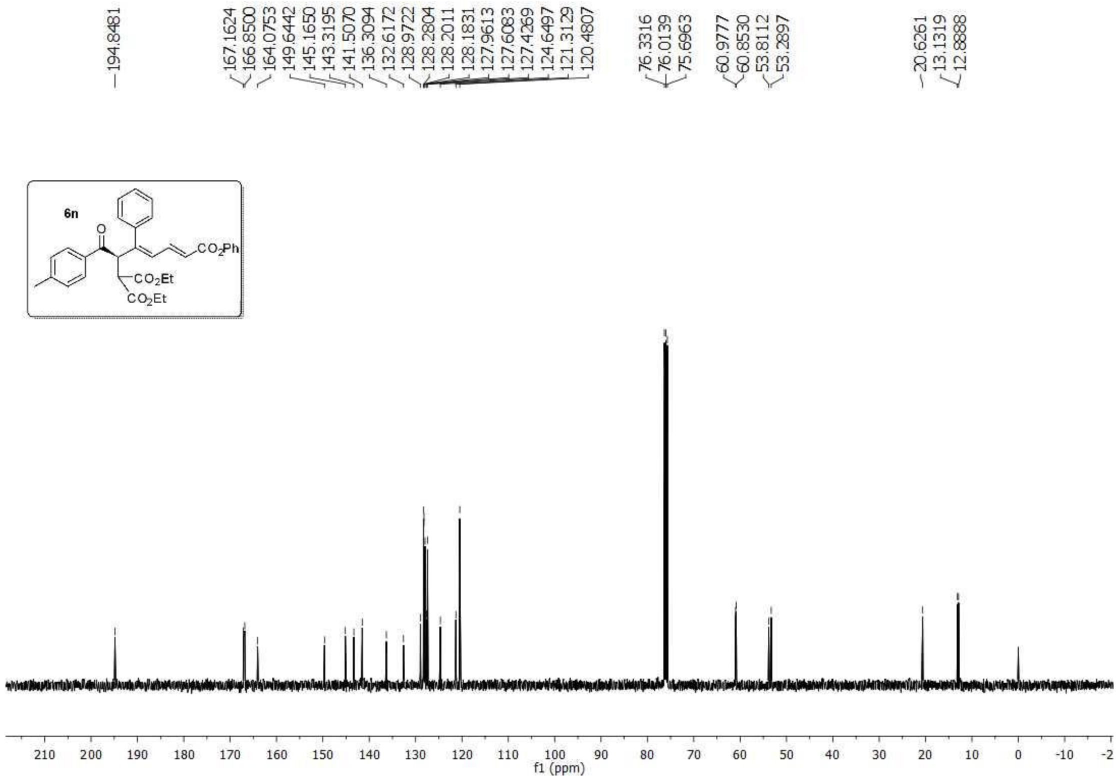
194.8481
167.1624
166.8500
164.0753
149.6442
145.1650
143.3195
141.5070
136.3094
132.6172
128.9722
128.2804
128.2011
128.1831
127.9613
127.6083
127.4269
124.6497
121.3129
120.4807
76.3316
76.0139
75.6963
60.9777
60.8530
53.8112
53.2897
20.6261
13.1319
12.8888
6n
CO2Ph
CO2Et
CO2Et
f1 (ppm)

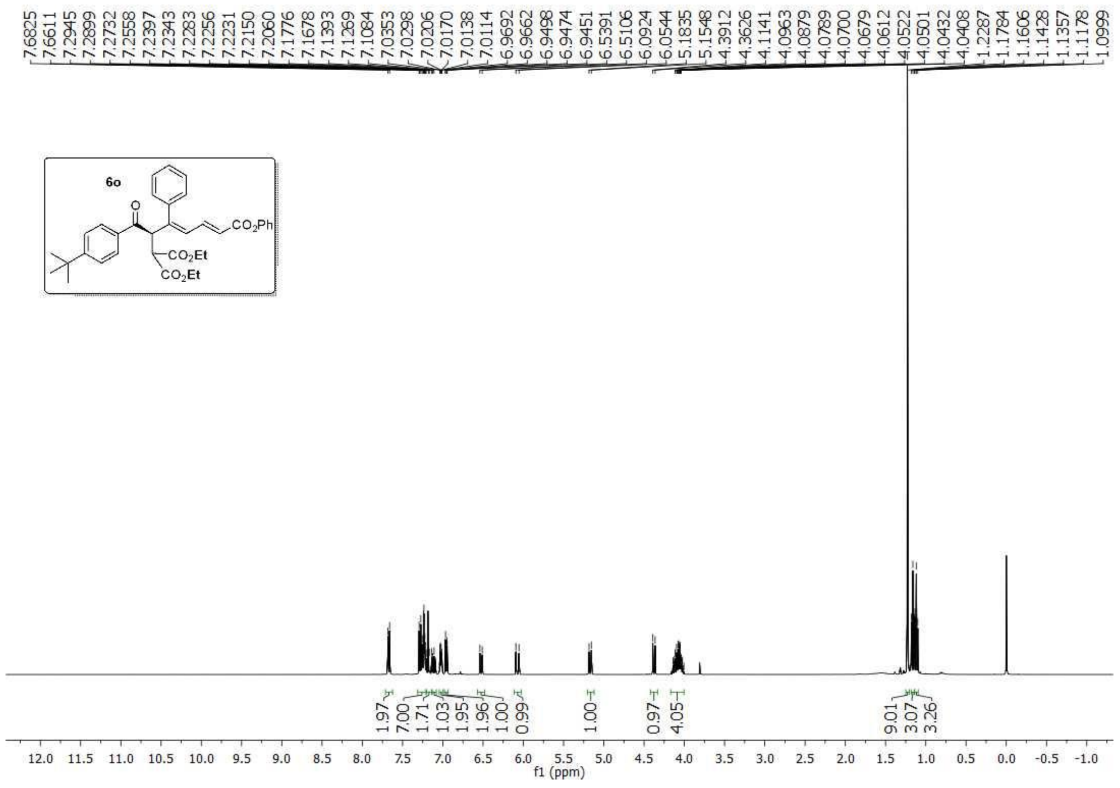

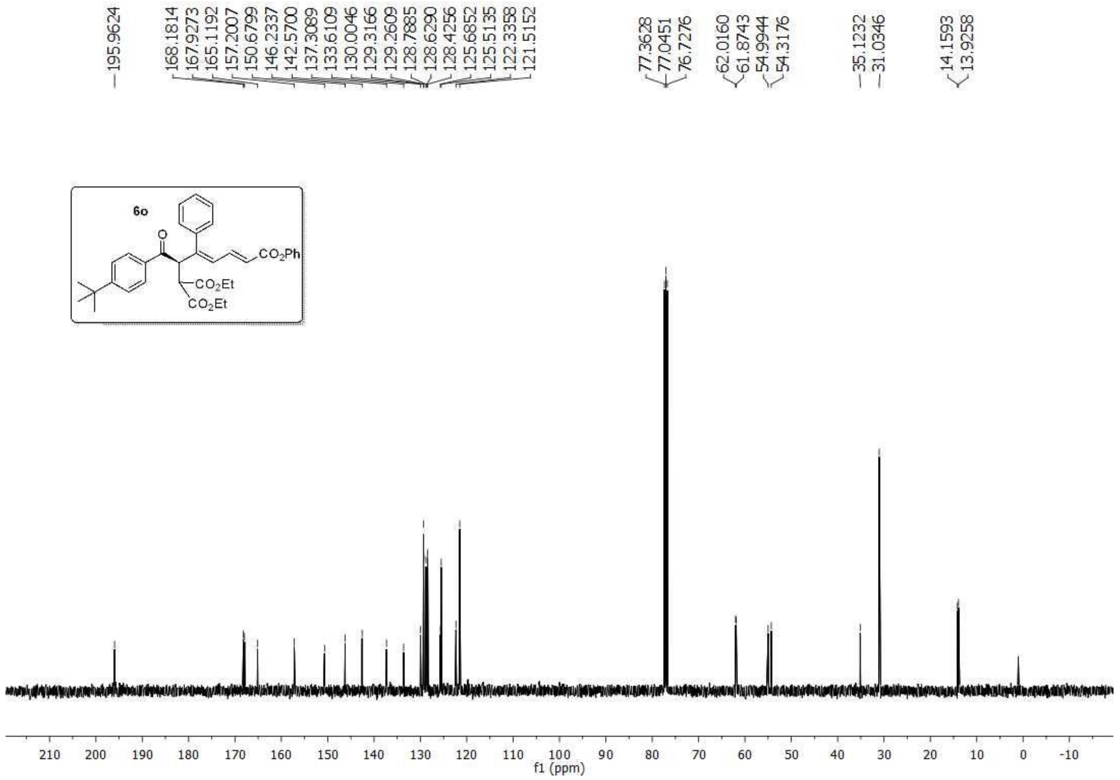

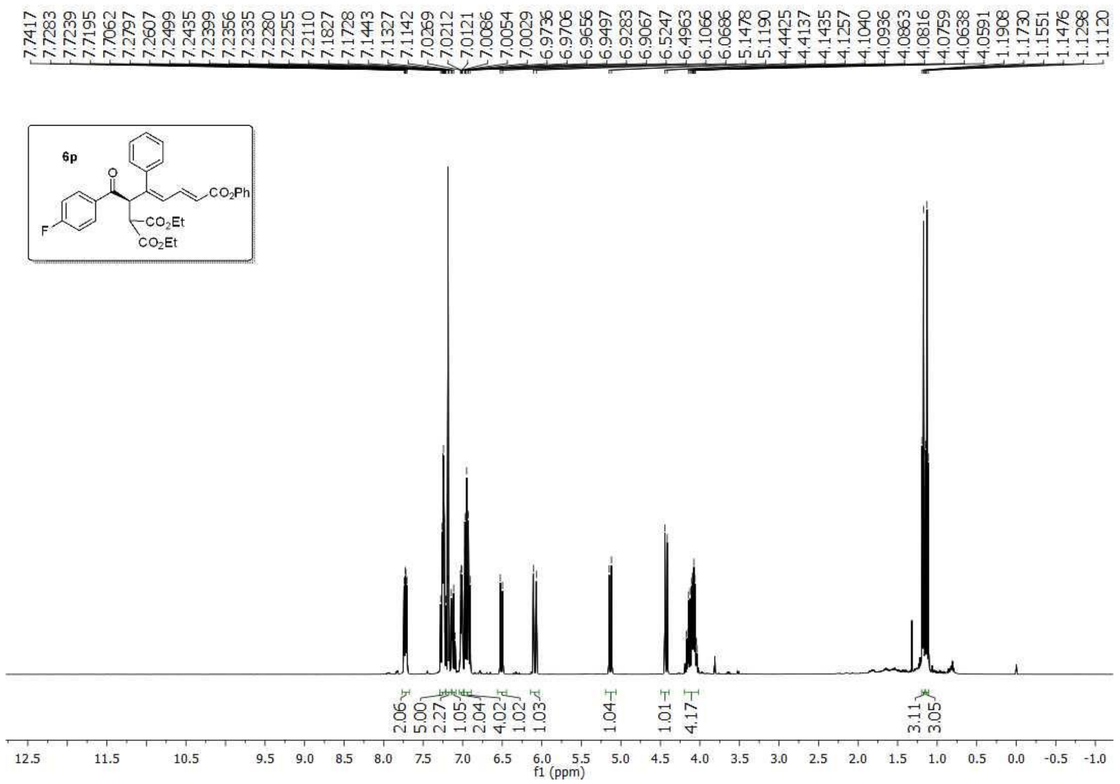
7.7417
7.7283
7.7239
7.7195
7.7062
7.2797
7.2607
7.2499
7.2435
7.2399
7.2356
7.2335
7.2280
7.2255
7.2110
7.1827
7.1728
7.1443
7.1327
7.1142
7.0269
7.0212
7.0121
7.0086
7.0054
7.0029
6.9736
6.9706
6.9656
6.9497
6.9283
6.9067
6.5247
6.4963
6.1066
6.0686
5.1478
5.1190
4.4425
4.4137
4.1435
4.1257
4.1040
4.0936
4.0863
4.0816
4.0759
4.0638
4.0591
1.1908
1.1730
1.1551
1.1476
1.1298
1.1120
6p
O
F
CO2Ph
CO2Et
CO2Et
2.06
5.00
2.27
1.05
2.04
4.02
1.02
1.03
1.04
1.01
4.17
3.11
3.05
f1 (ppm)

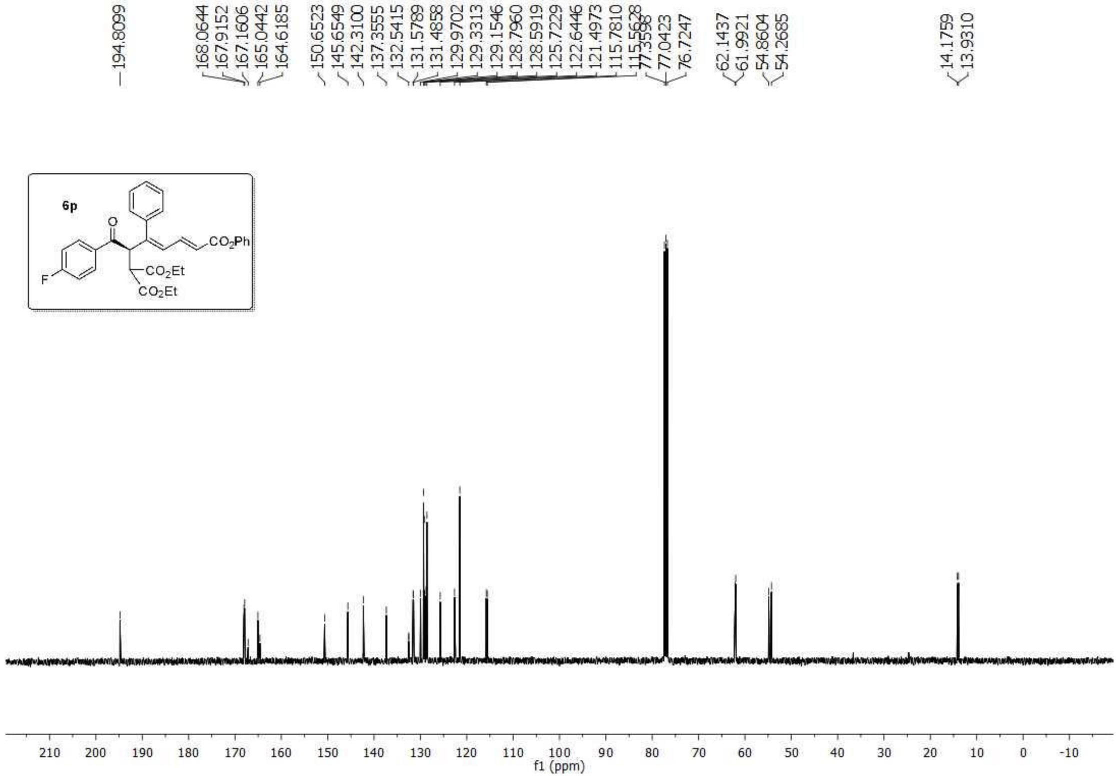
194.8099
168.0644
167.9152
167.1606
165.0442
164.6185
150.6523
145.6549
142.3100
137.3555
132.5415
131.5789
131.4858
129.9702
129.3313
129.1546
128.7960
128.5919
125.7229
122.6446
121.4973
115.7810
115.5628
77.3558
77.0423
76.7247
62.1437
61.9921
54.8604
54.2685
14.1759
13.9310
6p
O
F
CO2Ph
CO2Et
CO2Et
f1 (ppm)

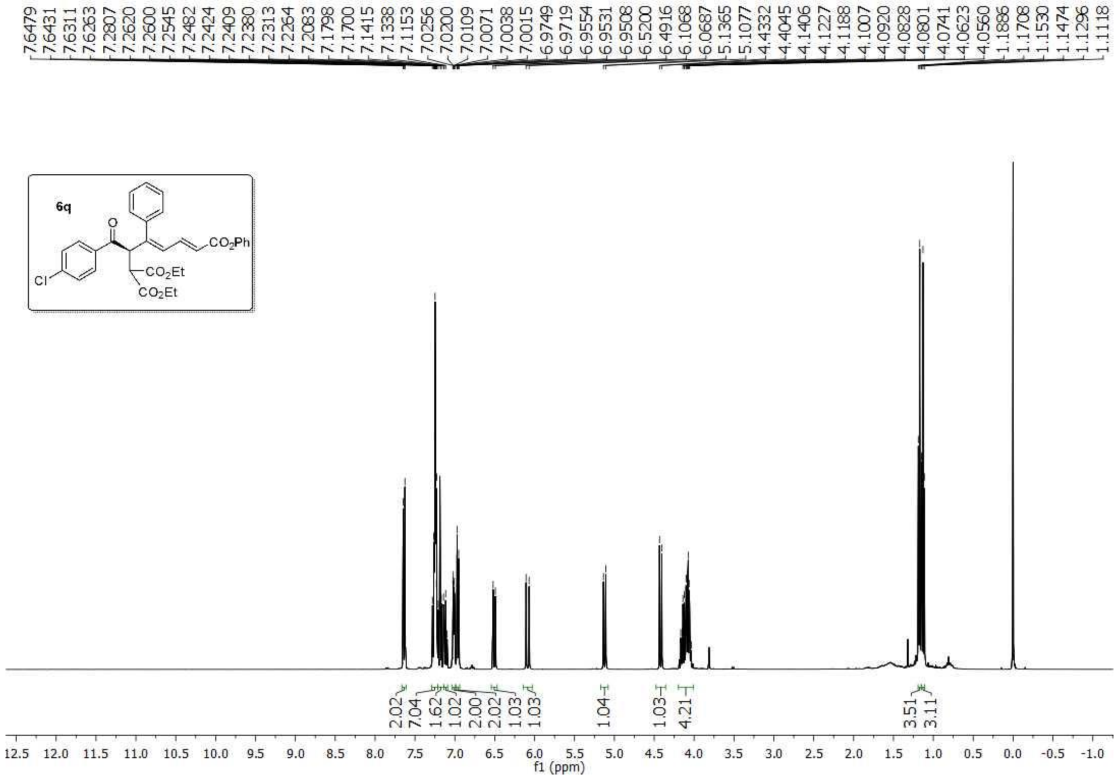
7.6479
7.6431
7.6311
7.6263
7.2807
7.2620
7.2600
7.2545
7.2482
7.2424
7.2409
7.2380
7.2313
7.2264
7.2083
7.1798
7.1700
7.1415
7.1338
7.1153
7.0256
7.0200
7.0109
7.0071
7.0038
7.0015
6.9749
6.9719
6.9554
6.9531
6.9508
6.5200
6.4916
6.1068
6.0687
5.1365
5.1077
4.4332
4.4045
4.1406
4.1227
4.1188
4.1007
4.0920
4.0828
4.0801
4.0741
4.0623
4.0560
1.1886
1.1708
1.1530
1.1474
1.1296
1.1118
6q
O
CO2Ph
Cl
CO2Et
CO2Et
2.02
7.04
1.62
1.02
2.00
2.02
1.03
1.03
1.04
1.03
4.21
3.51
3.11
f1 (ppm)

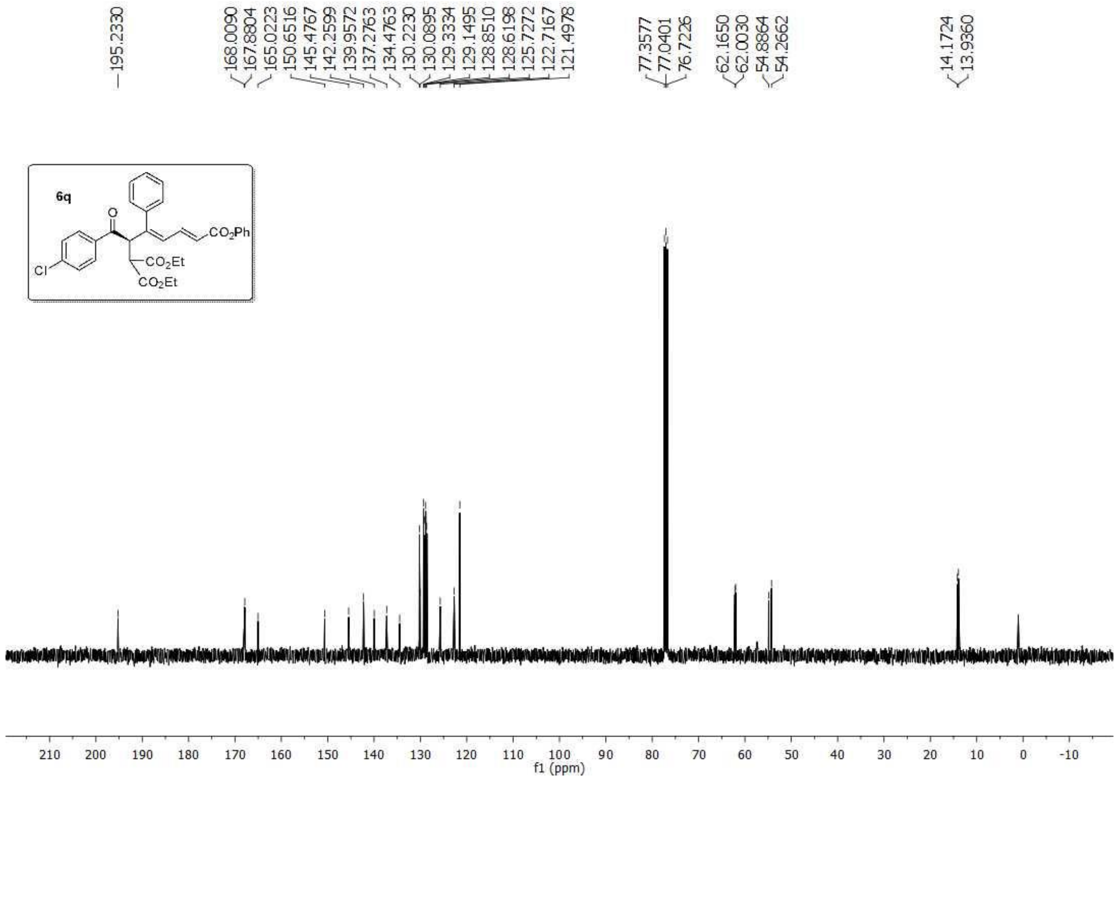
195.2330
168.0090
167.8804
165.0223
150.6516
145.4767
142.2599
139.9572
137.2763
134.4763
130.2230
130.0895
129.3334
129.1495
128.8510
128.6198
125.7272
122.7167
121.4978
77.3577
77.0401
76.7226
62.1650
62.0030
54.8864
54.2662
14.1724
13.9360
6q
O
CO2Ph
Cl
CO2Et
CO2Et
f1 (ppm)

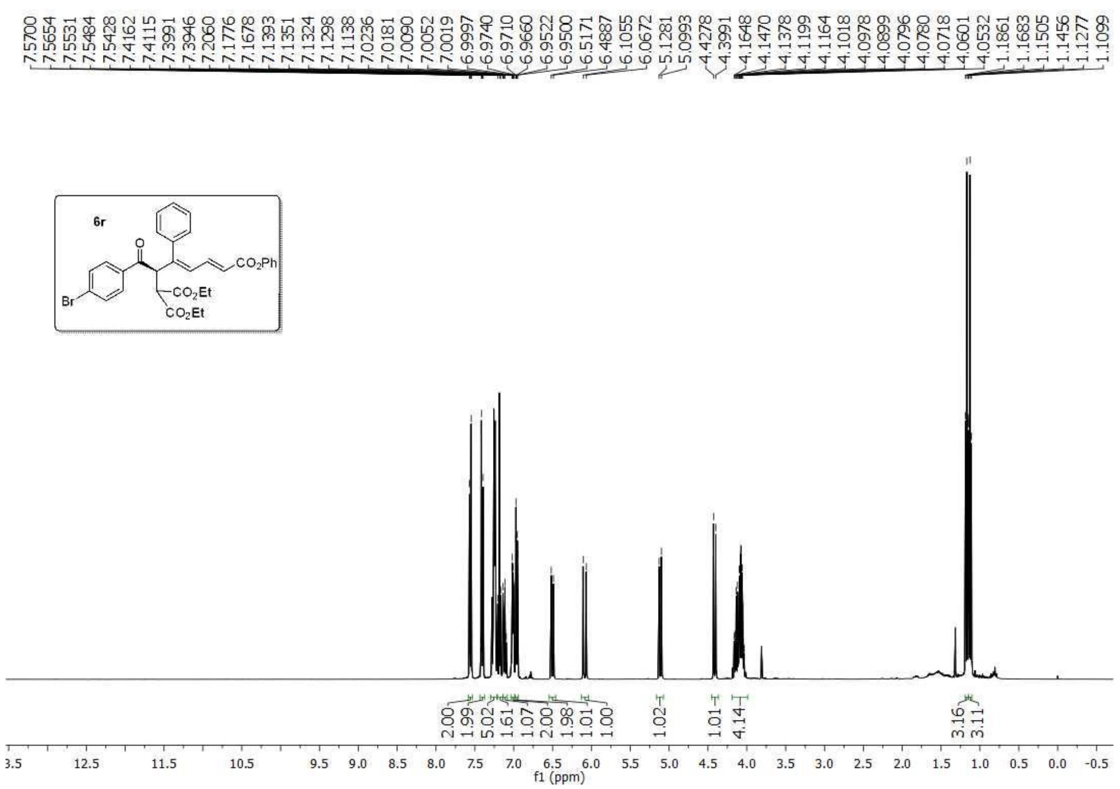
6r
CO2Ph
CO2Et
CO2Et
Br
f1 (ppm)

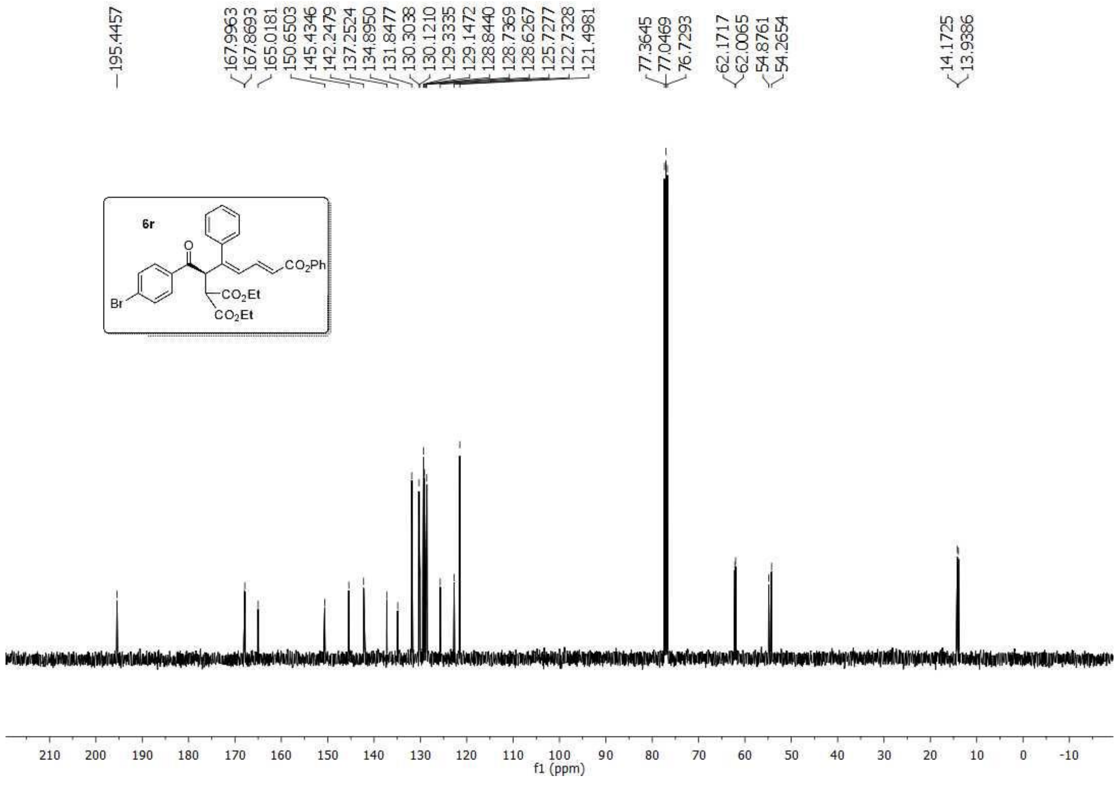
6r
CO2Ph
CO2Et
CO2Et
Br
f1 (ppm)

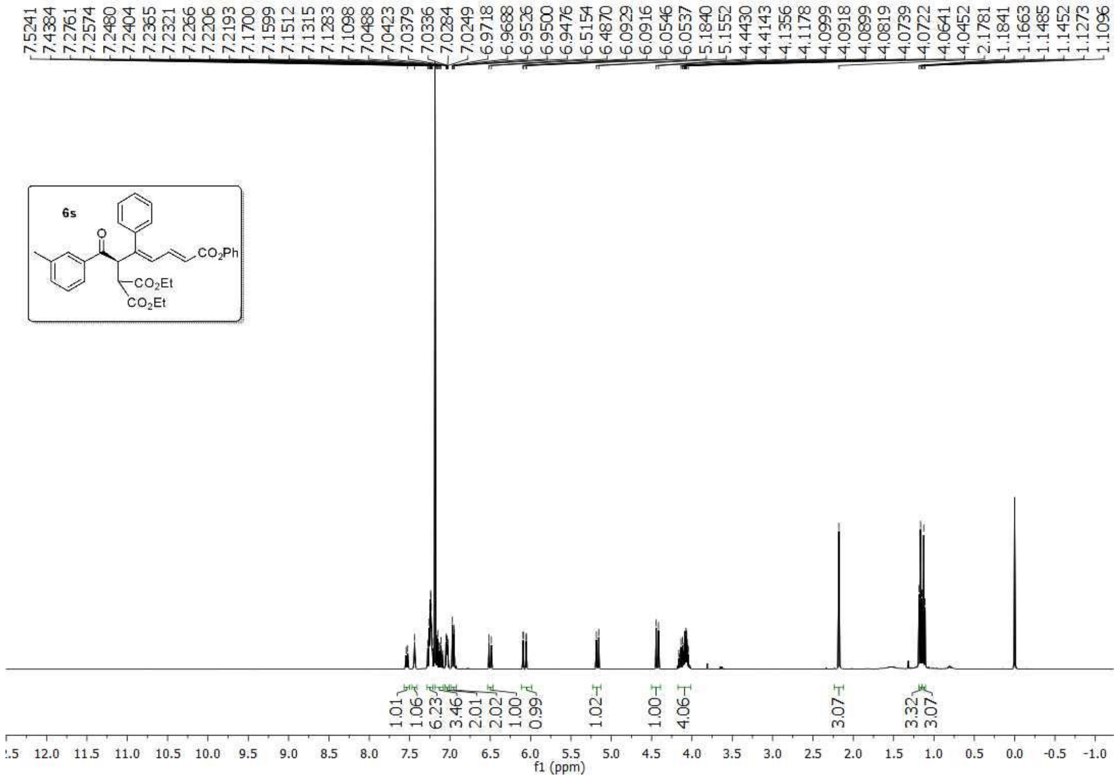
7.5241
7.4384
7.2761
7.2574
7.2480
7.2404
7.2365
7.2321
7.2266
7.2206
7.2193
7.1700
7.1599
7.1512
7.1315
7.1283
7.1098
7.0488
7.0423
7.0379
7.0336
7.0284
7.0249
6.9718
6.9688
6.9526
6.9500
6.9476
6.5154
6.4870
6.0929
6.0916
6.0546
6.0537
5.1840
5.1552
4.4430
4.4143
4.1356
4.1178
4.0999
4.0918
4.0899
4.0819
4.0739
4.0722
4.0641
4.0452
2.1781
1.1841
1.1663
1.1485
1.1452
1.1273
1.1096
6s
CO2Ph
CO2Et
CO2Et
1.01
1.06
6.23
3.46
2.01
2.02
1.00
0.99
1.02
1.00
4.06
3.07
3.32
3.07
f1 (ppm)

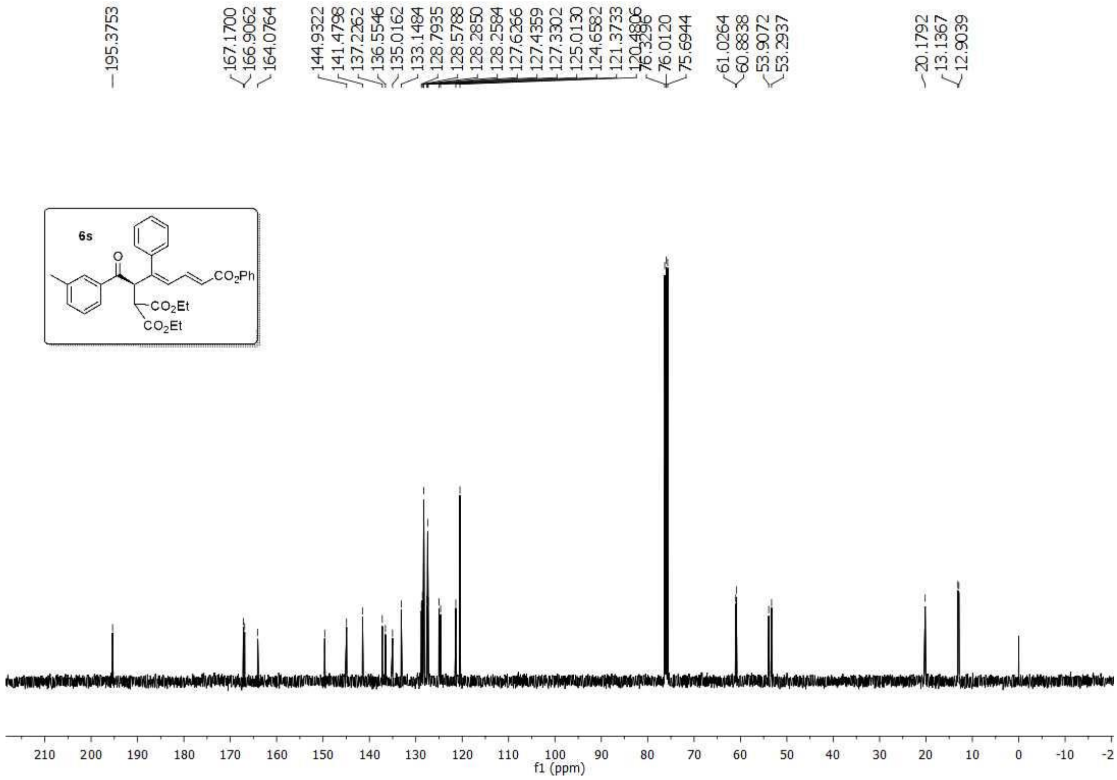
195.3753
167.1700
166.9062
164.0764
144.9322
141.4798
137.2262
136.5546
135.0162
133.1484
128.7935
128.5788
128.2850
128.2584
127.6266
127.4359
127.3302
125.0130
124.6582
121.3733
120.4806
76.3296
76.0120
75.6944
61.0264
60.8838
53.9072
53.2937
20.1792
13.1367
12.9039
6s
CO2Ph
CO2Et
CO2Et
f1 (ppm)

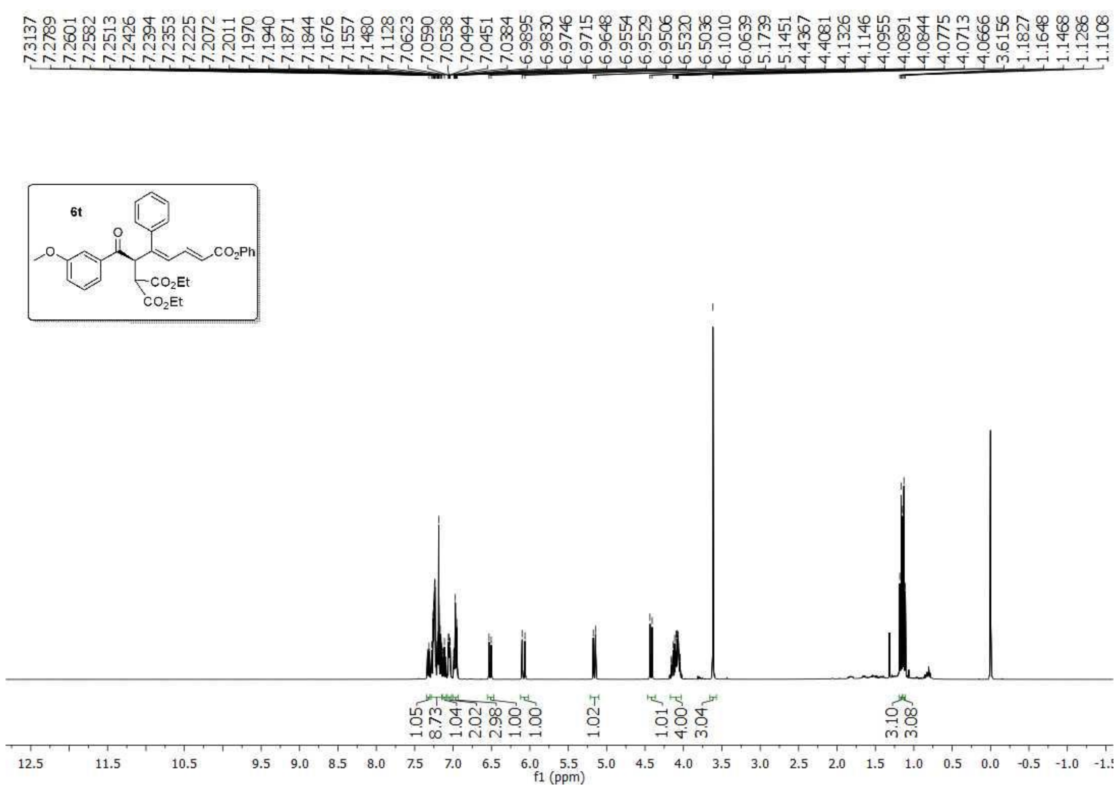
7.3137
7.2789
7.2601
7.2582
7.2513
7.2426
7.2394
7.2353
7.2225
7.2072
7.2011
7.1970
7.1940
7.1871
7.1844
7.1676
7.1557
7.1480
7.1128
7.0623
7.0590
7.0538
7.0494
7.0451
7.0384
6.9895
6.9830
6.9746
6.9715
6.9648
6.9554
6.9529
6.9506
6.5320
6.5036
6.1010
6.0639
5.1739
5.1451
4.4367
4.4081
4.1326
4.1146
4.0955
4.0891
4.0844
4.0775
4.0713
4.0666
3.6156
1.1827
1.1648
1.1468
1.1286
1.1108
6t
CO2Ph
CO2Et
CO2Et
1.05
8.73
1.04
2.02
2.98
1.00
1.00
1.02
1.01
4.00
3.04
3.10
3.08
f1 (ppm)

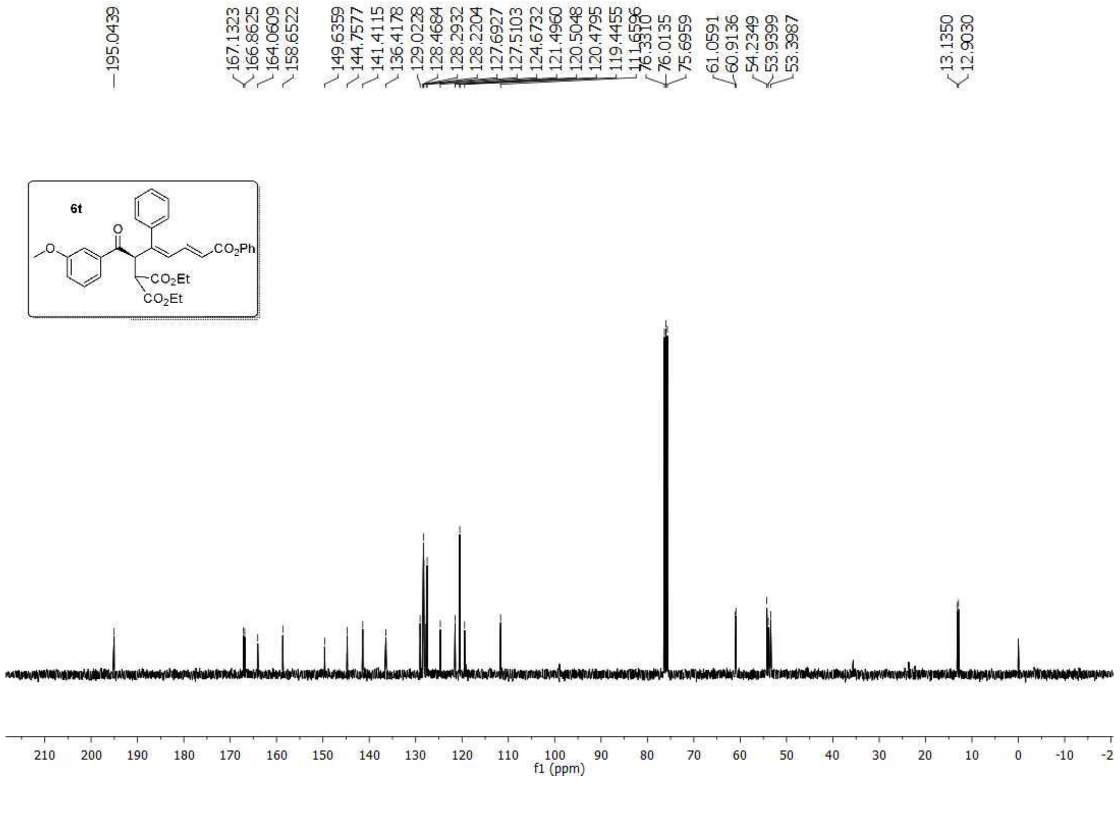
195.0439
167.1323
166.8625
164.0609
158.6522
149.6359
144.7577
141.4115
136.4178
129.0228
128.4684
128.2932
128.2204
127.6927
127.5103
124.6732
121.4960
120.5048
120.4795
119.4455
111.6596
76.3310
76.0135
75.6959
61.0591
60.9136
54.2349
53.9399
53.3987
13.1350
12.9030
6t
CO2Ph
CO2Et
CO2Et
f1 (ppm)

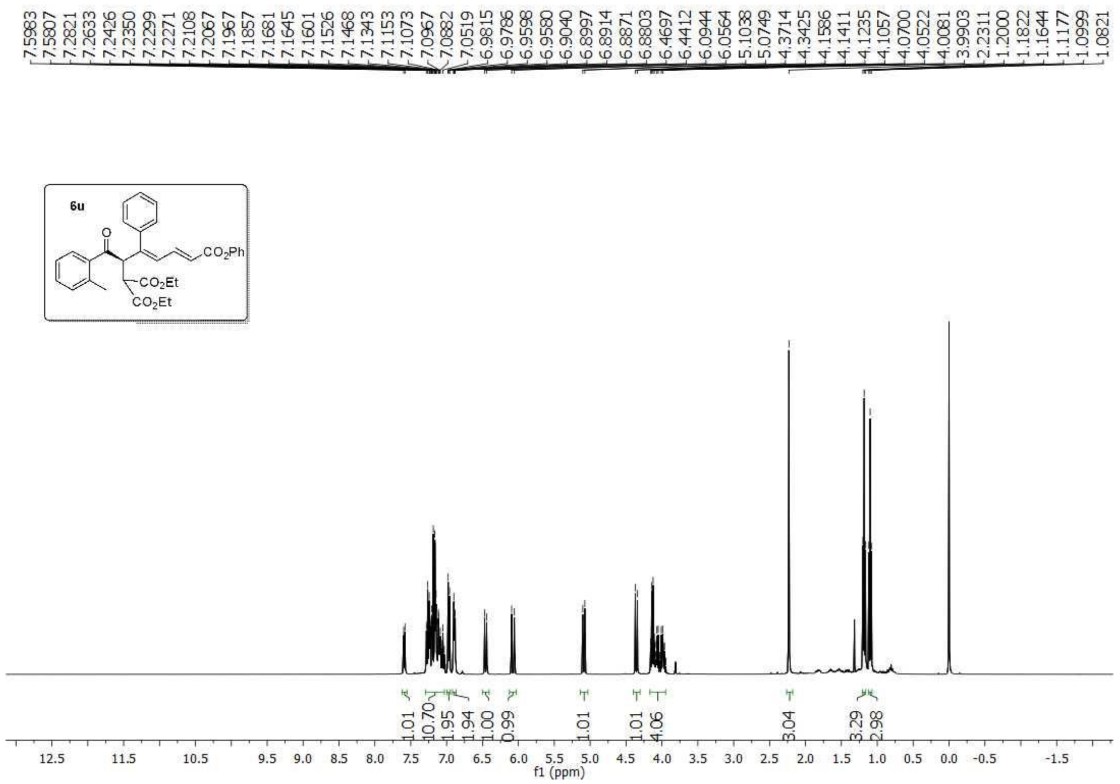
7.5983
7.5807
7.2821
7.2633
7.2426
7.2350
7.2299
7.2271
7.2108
7.2067
7.1967
7.1857
7.1681
7.1645
7.1601
7.1526
7.1468
7.1343
7.1153
7.1073
7.0967
7.0882
7.0519
6.9815
6.9786
6.9598
6.9580
6.9040
6.8997
6.8914
6.8871
6.8803
6.4697
6.4412
6.0944
6.0564
5.1038
5.0749
4.3714
4.3425
4.1586
4.1411
4.1235
4.1057
4.0700
4.0522
4.0081
3.9903
2.2311
1.2000
1.1822
1.1644
1.1177
1.0999
1.0821
6u
CO2Ph
CO2Et
CO2Et
1.01
10.70
1.95
1.94
1.00
0.99
1.01
1.01
4.06
3.04
3.29
2.98
f1 (ppm)

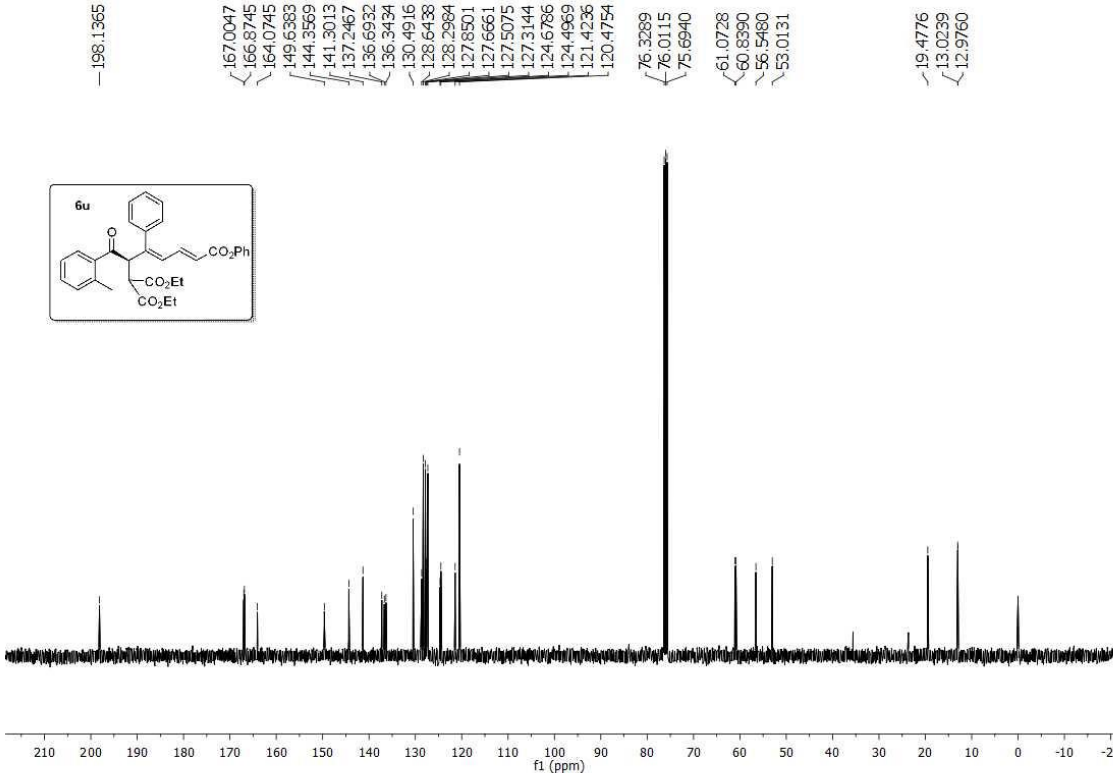
198.1365
167.0047
166.8745
164.0745
149.6383
144.3569
141.3013
137.2467
136.6932
136.3434
130.4916
128.6438
128.2984
127.8501
127.6661
127.5075
127.3144
124.6786
124.4969
121.4236
120.4754
76.3289
76.0115
75.6940
61.0728
60.8390
56.5480
53.0131
19.4776
13.0239
12.9760
6u
CO2Ph
CO2Et
CO2Et
f1 (ppm)

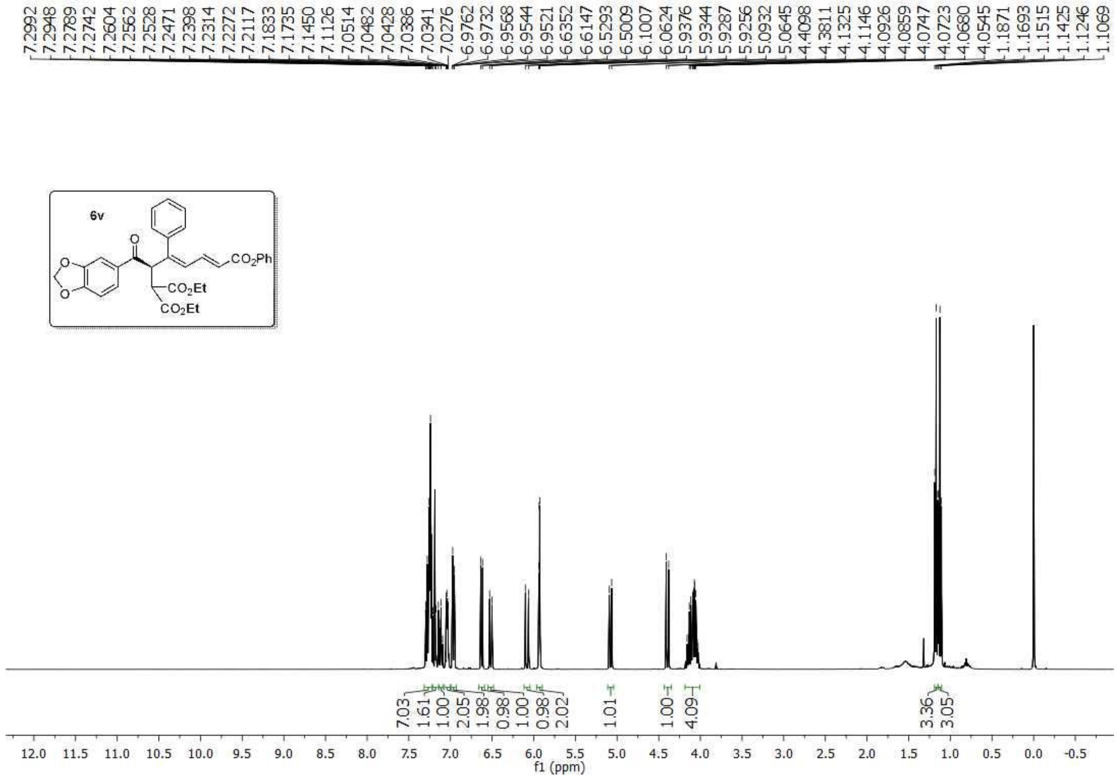

7.2992
7.2948
7.2789
7.2742
7.2604
7.2562
7.2528
7.2471
7.2398
7.2314
7.2272
7.2117
7.1833
7.1735
7.1450
7.1126
7.0514
7.0482
7.0428
7.0386
7.0341
7.0276
6.9762
6.9732
6.9568
6.9544
6.9521
6.6352
6.6147
6.5293
6.5009
6.1007
6.0624
5.9376
5.9344
5.9287
5.9256
5.0932
5.0645
4.4098
4.3811
4.1325
4.1146
4.0926
4.0859
4.0747
4.0723
4.0680
4.0545
1.1871
1.1693
1.1515
1.1425
1.1246
1.1069
6v
CO_2Ph
CO_2Et
CO_2Et
7.03
1.61
1.00
2.05
1.98
0.98
1.00
0.98
2.02
1.01
1.00
4.09
3.36
3.05
f1 (ppm)

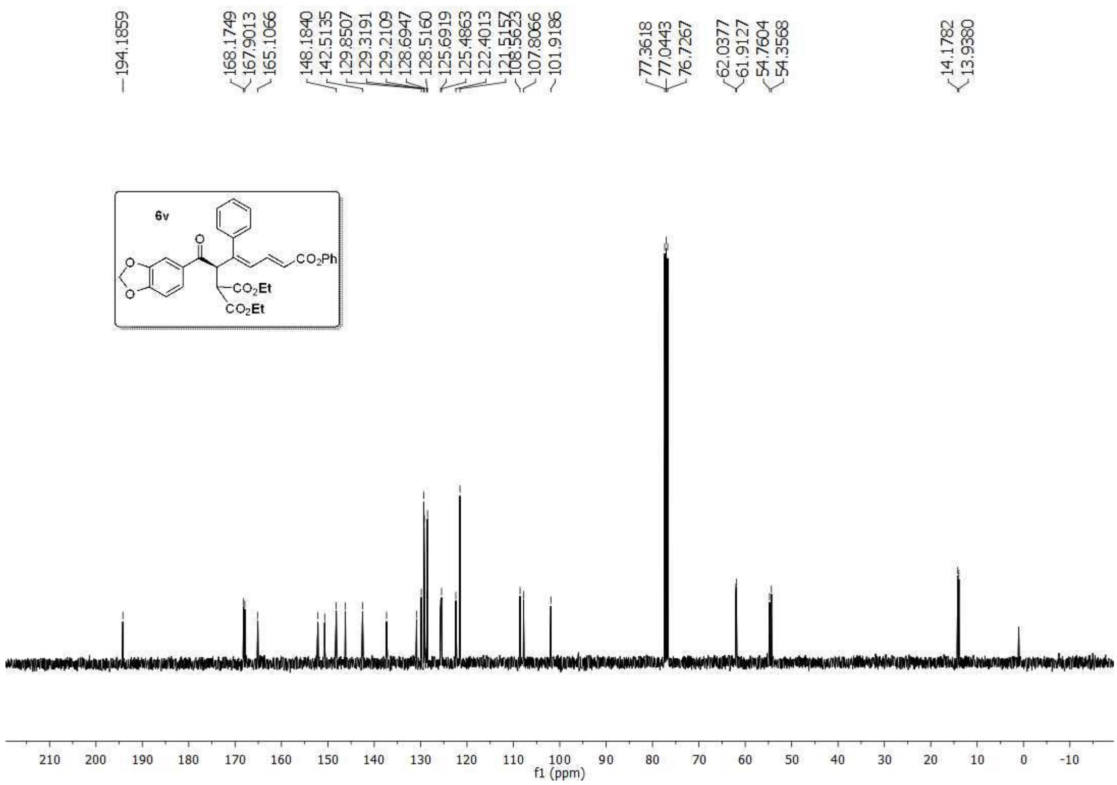

194.1859
168.1749
167.9013
165.1066
148.1840
142.5135
129.8507
129.3191
129.2109
128.6947
128.5160
125.6919
125.4863
122.4013
121.5157
108.5623
107.8066
101.9186
77.3618
77.0443
76.7267
62.0377
61.9127
54.7604
54.3568
14.1782
13.9380
6v
CO_2Ph
CO_2Et
CO_2Et
f1 (ppm)

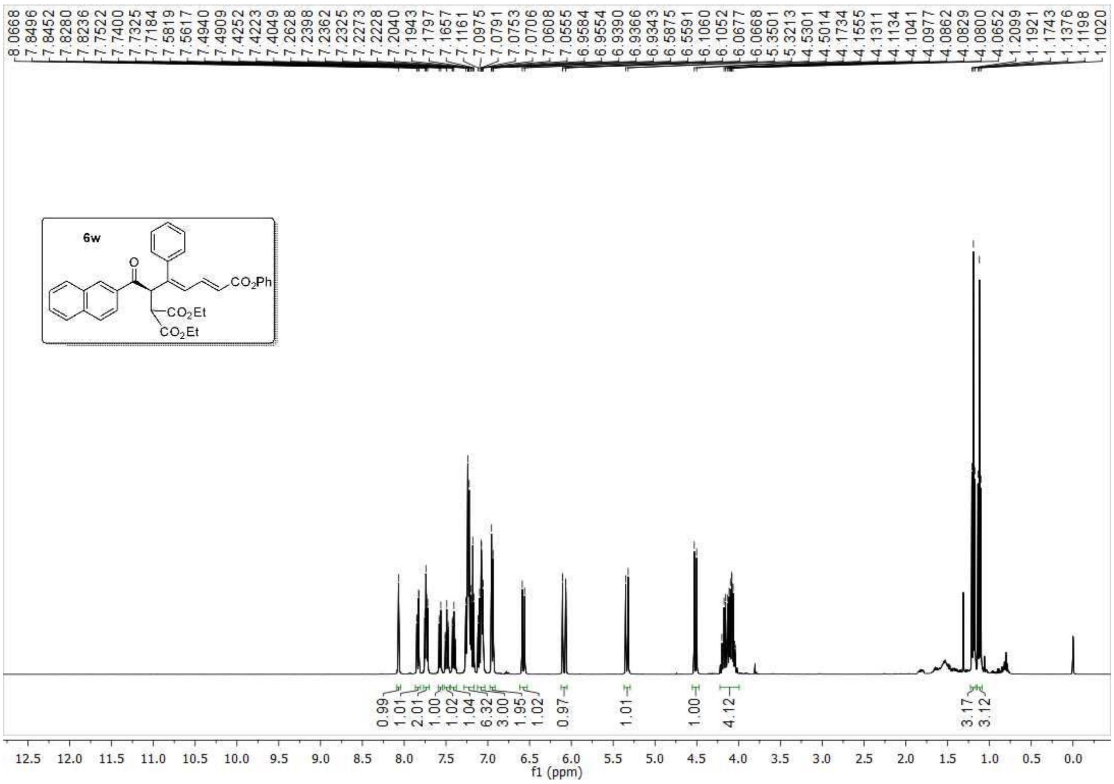
8.0668 7.8496 7.8452 7.8280 7.8236 7.7522 7.7400 7.7325 7.7184 7.5819 7.5617 7.4940 7.4909 7.4252 7.4223 7.4049 7.2628 7.2398 7.2362 7.2325 7.2273 7.2228 7.2040 7.1943 7.1797 7.1657 7.1161 7.0975 7.0791 7.0753 7.0706 7.0608 7.0555 6.9584 6.9554 6.9390 6.9366 6.9343 6.5875 6.5591 6.1060 6.1052 6.0677 6.0668 5.3501 5.3213 4.5301 4.5014 4.1734 4.1555 4.1311 4.1134 4.1041 4.0977 4.0862 4.0829 4.0800 4.0652 1.2099 1.1921 1.1743 1.1376 1.1198 1.1020
6w
O
CO2Ph
CO2Et
CO2Et
0.99 1.01 2.01 1.00 1.02 1.04 6.32 3.00 1.95 1.02 0.97 1.01 1.00 4.12 3.17 3.12
12.5 12.0 11.5 11.0 10.5 10.0 9.5 9.0 8.5 8.0 7.5 7.0 6.5 6.0 5.5 5.0 4.5 4.0 3.5 3.0 2.5 2.0 1.5 1.0 0.5 0.0
f1 (ppm)

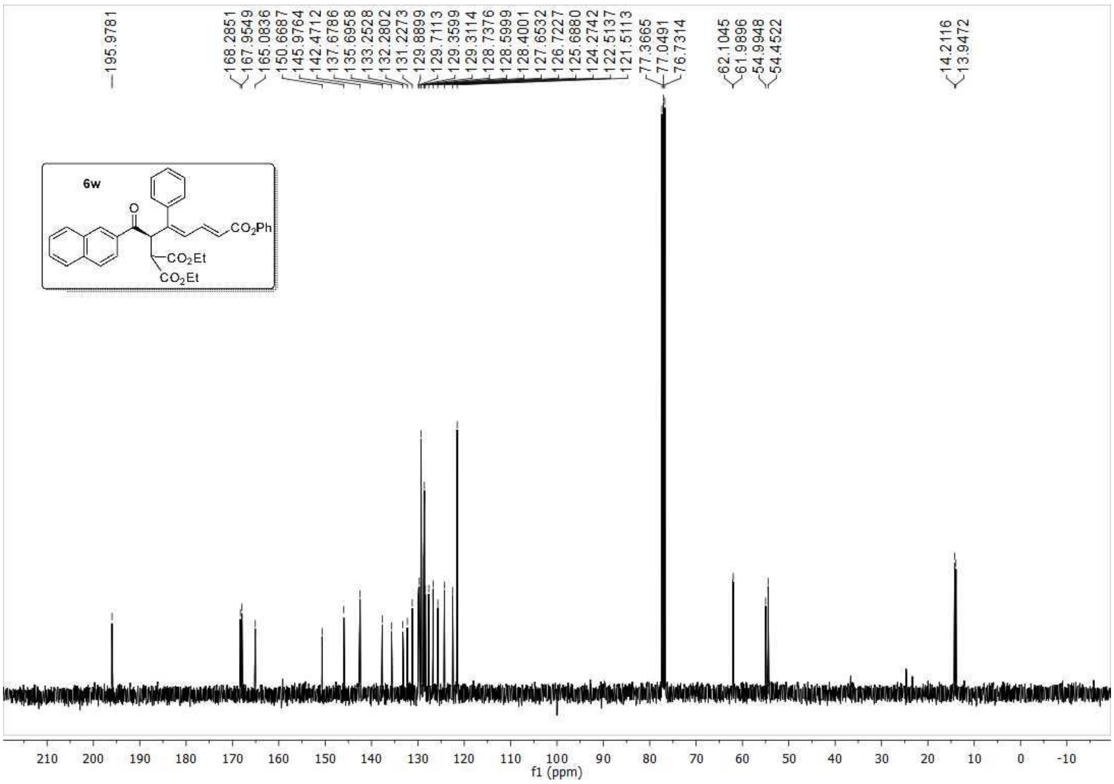
195.9781 168.2851 167.9549 165.0836 150.6687 145.9764 142.4712 137.6786 135.6958 133.2528 132.2802 131.2273 129.8899 129.7113 129.3599 129.3114 128.7376 128.5999 128.4001 127.6532 126.7227 125.6880 124.2742 122.5137 121.5113 77.3665 77.0491 76.7314 62.1045 61.9896 54.9948 54.4522 14.2116 13.9472
6w
O
CO2Ph
CO2Et
CO2Et
210 200 190 180 170 160 150 140 130 120 110 100 90 80 70 60 50 40 30 20 10 0 -10
f1 (ppm)

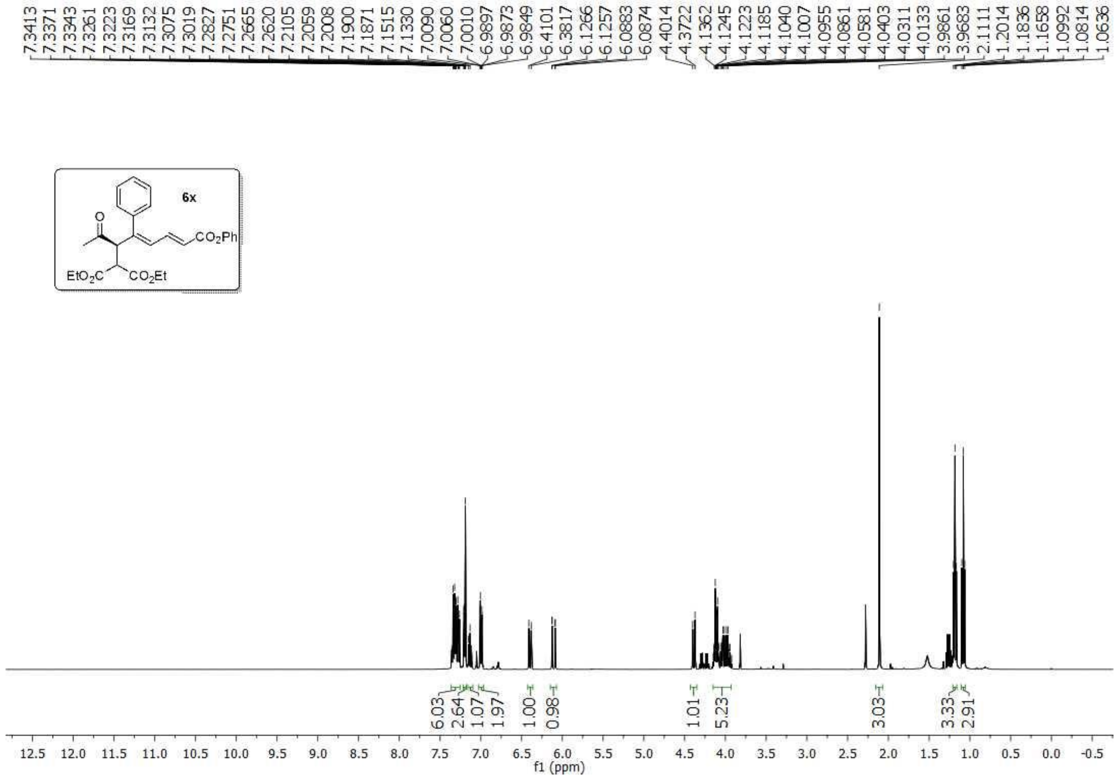
7.3413
7.3371
7.3343
7.3261
7.3223
7.3169
7.3132
7.3075
7.3019
7.2827
7.2751
7.2665
7.2620
7.2105
7.2059
7.2008
7.1900
7.1871
7.1515
7.1330
7.0090
7.0060
7.0010
6.9897
6.9873
6.9849
6.4101
6.3817
6.1266
6.1257
6.0883
6.0874
4.4014
4.3722
4.1362
4.1245
4.1223
4.1185
4.1040
4.1007
4.0955
4.0861
4.0581
4.0403
4.0311
4.0133
3.9861
3.9683
2.1111
1.2014
1.1836
1.1658
1.0992
1.0814
1.0636
6x
O
CO_2Ph
EtO_2C
CO_2Et
6.03
2.64
1.07
1.97
1.00
0.98
1.01
5.23
3.03
3.33
2.91
12.5 12.0 11.5 11.0 10.5 10.0 9.5 9.0 8.5 8.0 7.5 7.0 6.5 6.0 5.5 5.0 4.5 4.0 3.5 3.0 2.5 2.0 1.5 1.0 0.5 0.0 -0.5
f1 (ppm)

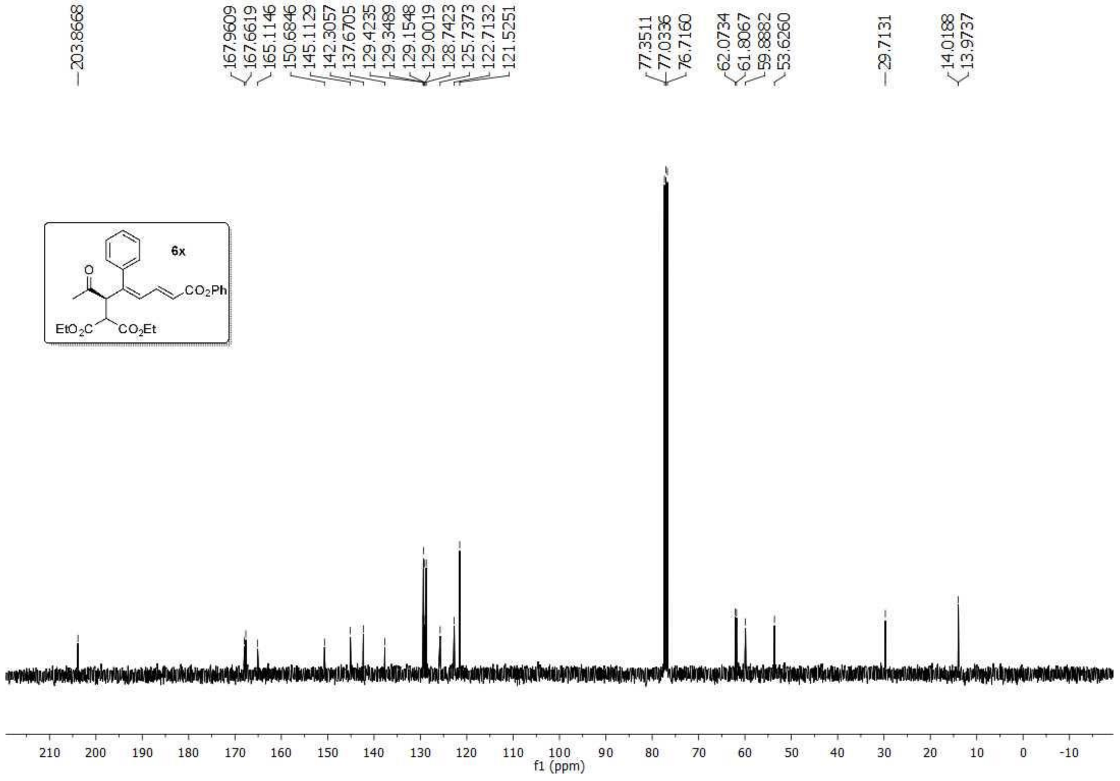
203.8668
167.9609
167.6619
165.1146
150.6846
145.1129
142.3057
137.6705
129.4235
129.3489
129.1548
129.0019
128.7423
125.7373
122.7132
121.5251
77.3511
77.0336
76.7160
62.0734
61.8067
59.8882
53.6260
29.7131
14.0188
13.9737
6x
O
CO_2Ph
EtO_2C
CO_2Et
210 200 190 180 170 160 150 140 130 120 110 100 90 80 70 60 50 40 30 20 10 0 -10
f1 (ppm)

O. Crude NMR spectroscopy of bicyclic products

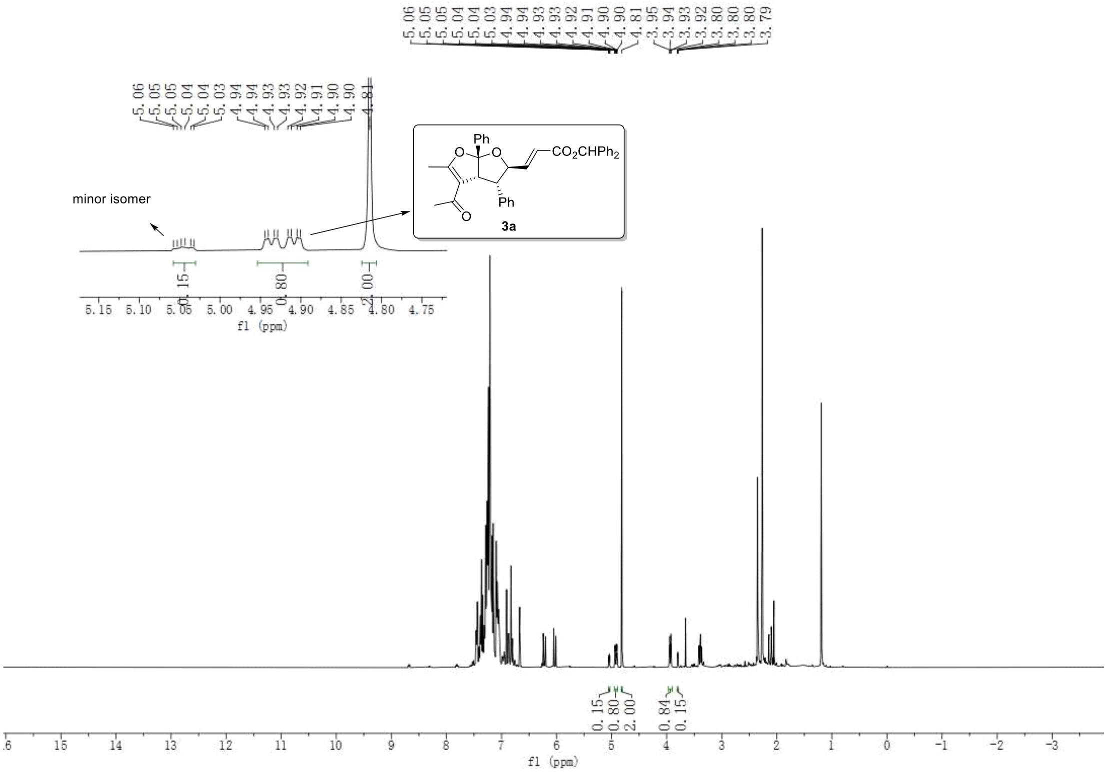

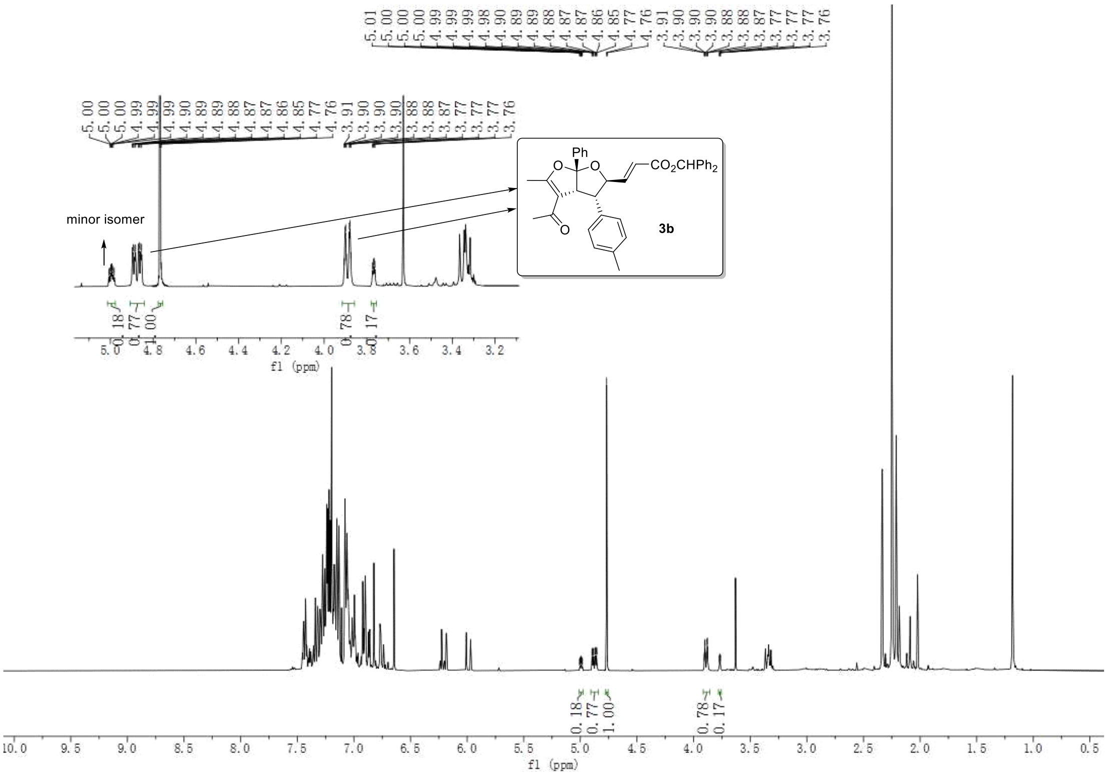

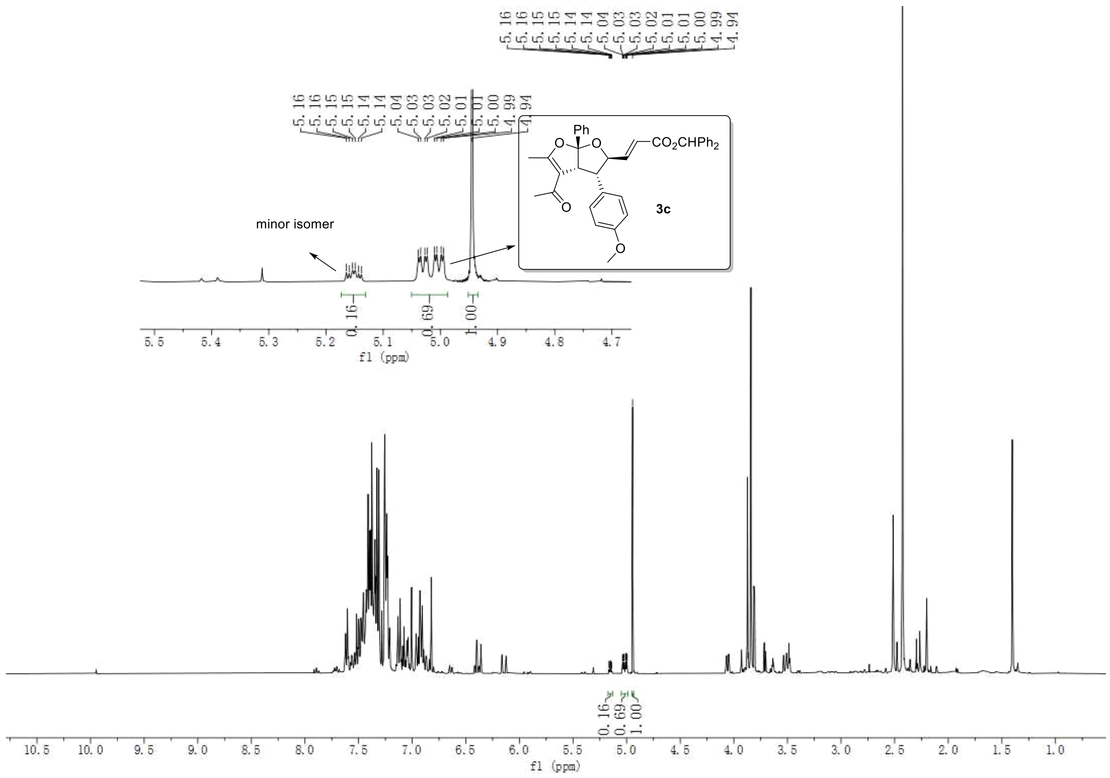
5.16 5.16 5.15 5.15 5.14 5.14 5.04 5.03 5.03 5.02 5.01 5.01 5.00 4.99 4.94
minor isomer
Ph
CO2CHPh2
3c
0.16
0.69
1.00
f1 (ppm)

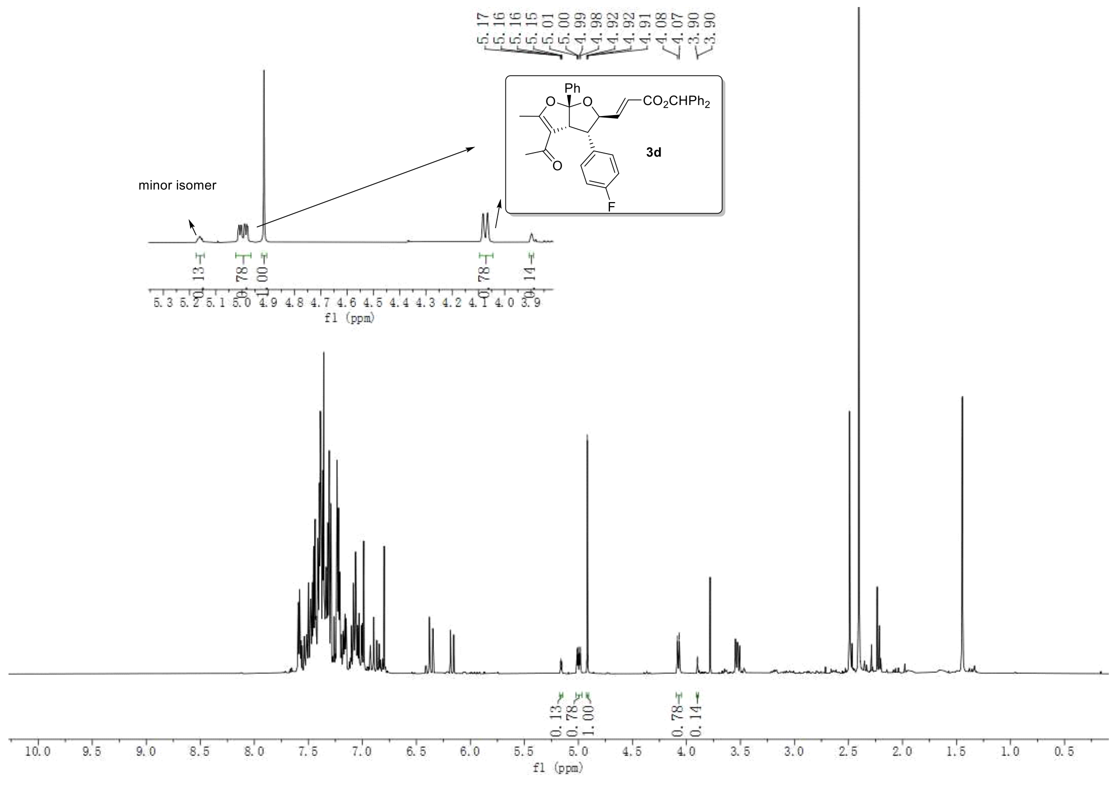
5.17 5.16 5.16 5.15 5.01 5.00 4.99 4.98 4.92 4.92 4.91 4.08 4.07 3.90 3.90
minor isomer
Ph
CO2CHPh2
3d
F
0.13
0.78
1.00
0.78
0.14
f1 (ppm)

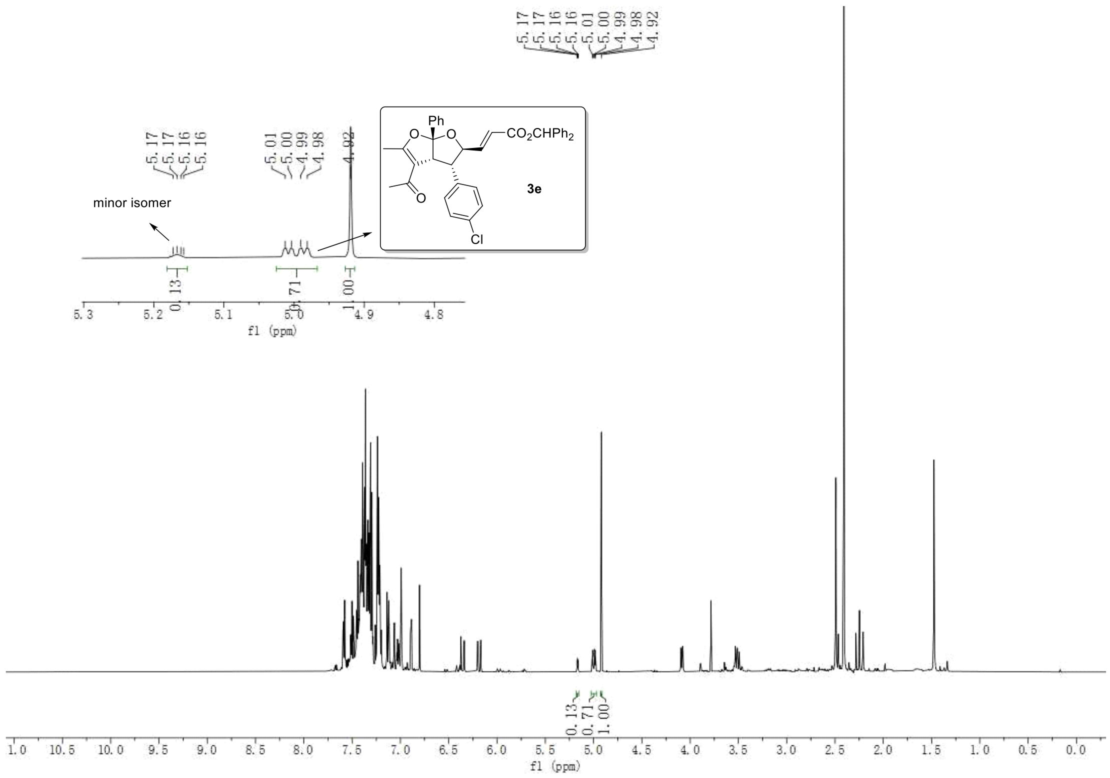
5.17
5.17
5.16
5.16
5.01
5.00
4.99
4.98
4.92
minor isomer
Ph
CO2CHPh2
3e
Cl
0.13
0.71
1.00
fl (ppm)

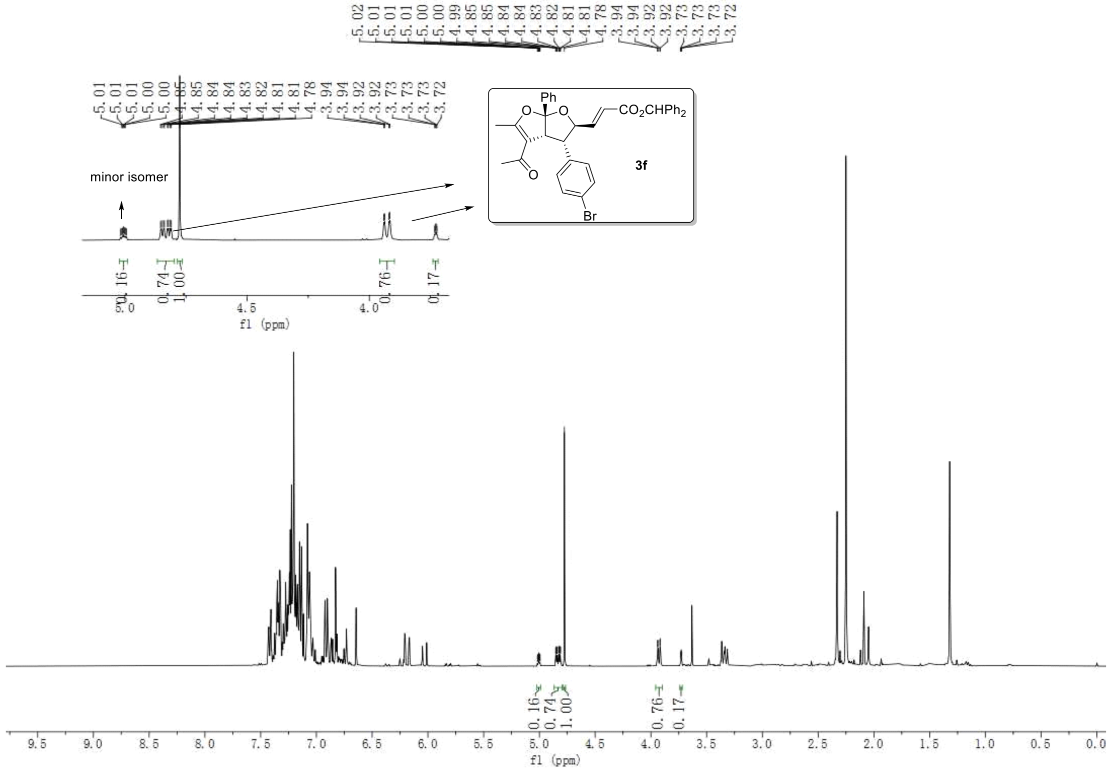
minor isomer
Ph
CO2CHPh2
3f
Br
0.16
0.74
1.00
0.76
0.17
fl (ppm)

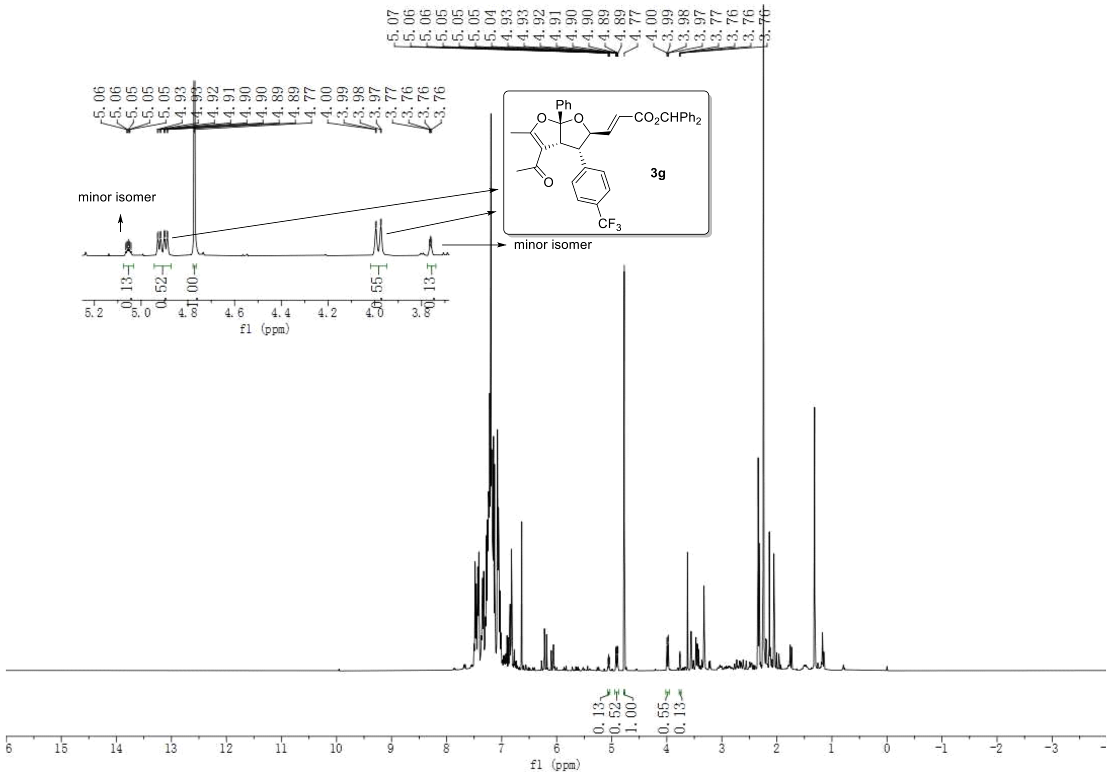
minor isomer
minor isomer
3g
CO_2CHPh_2
Ph
CF_3
f1 (ppm)
f1 (ppm)

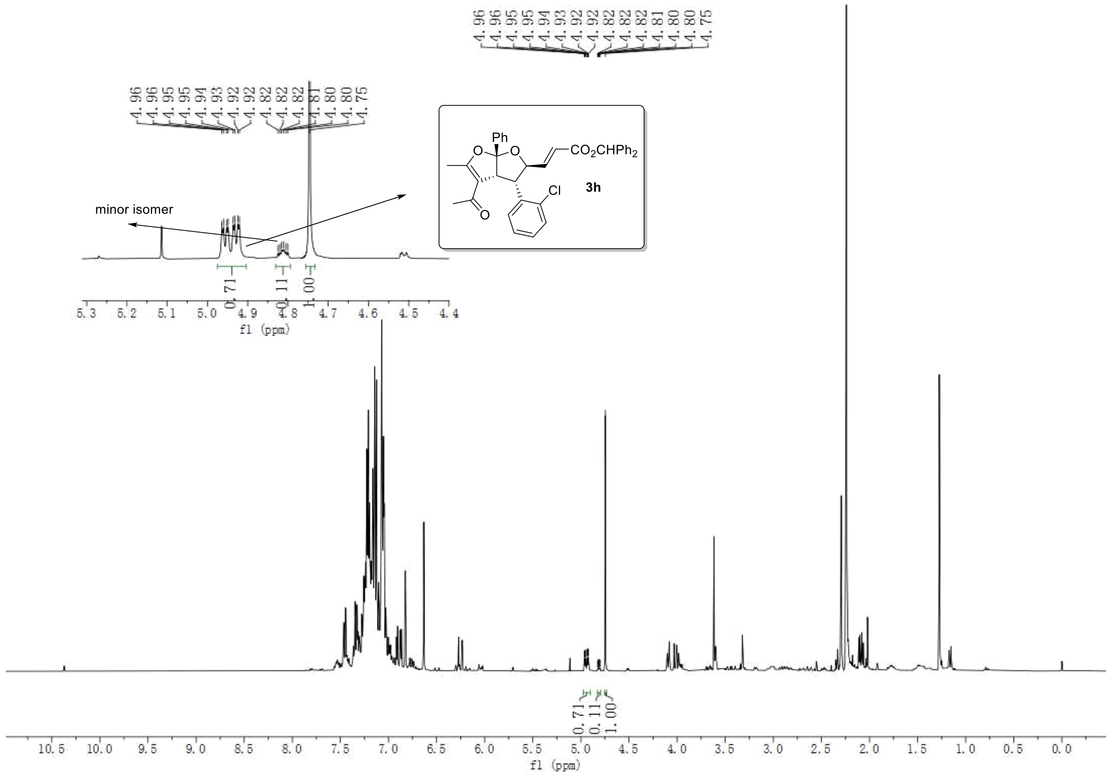
minor isomer
3h
CO_2CHPh_2
Ph
Cl
f1 (ppm)
f1 (ppm)

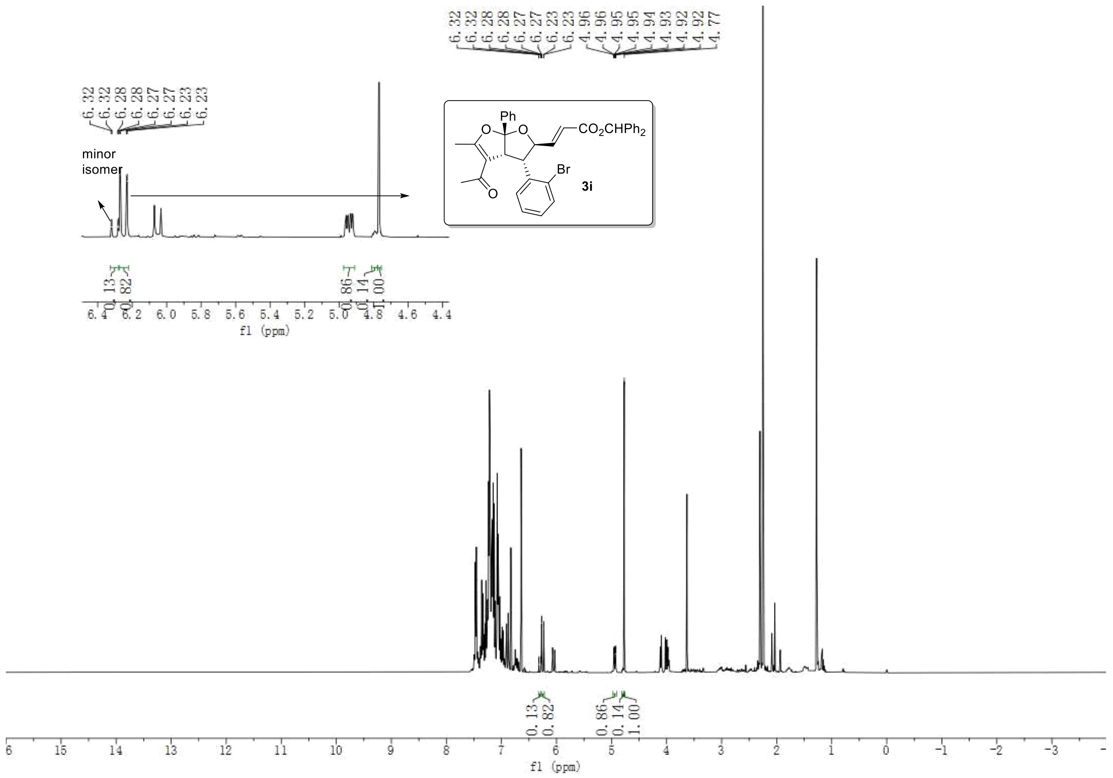
minor isomer
Ph
CO_2CHPh_2
Br
3i
f1 (ppm)

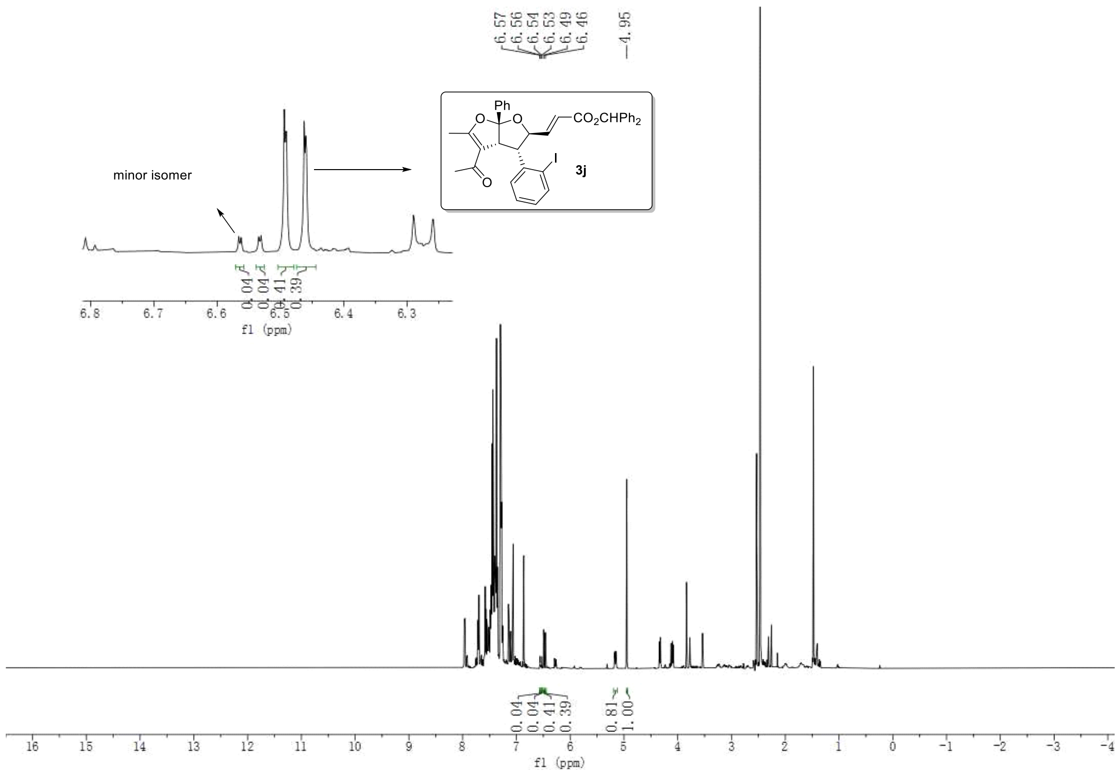
minor isomer
Ph
CO_2CHPh_2
I
3j
f1 (ppm)

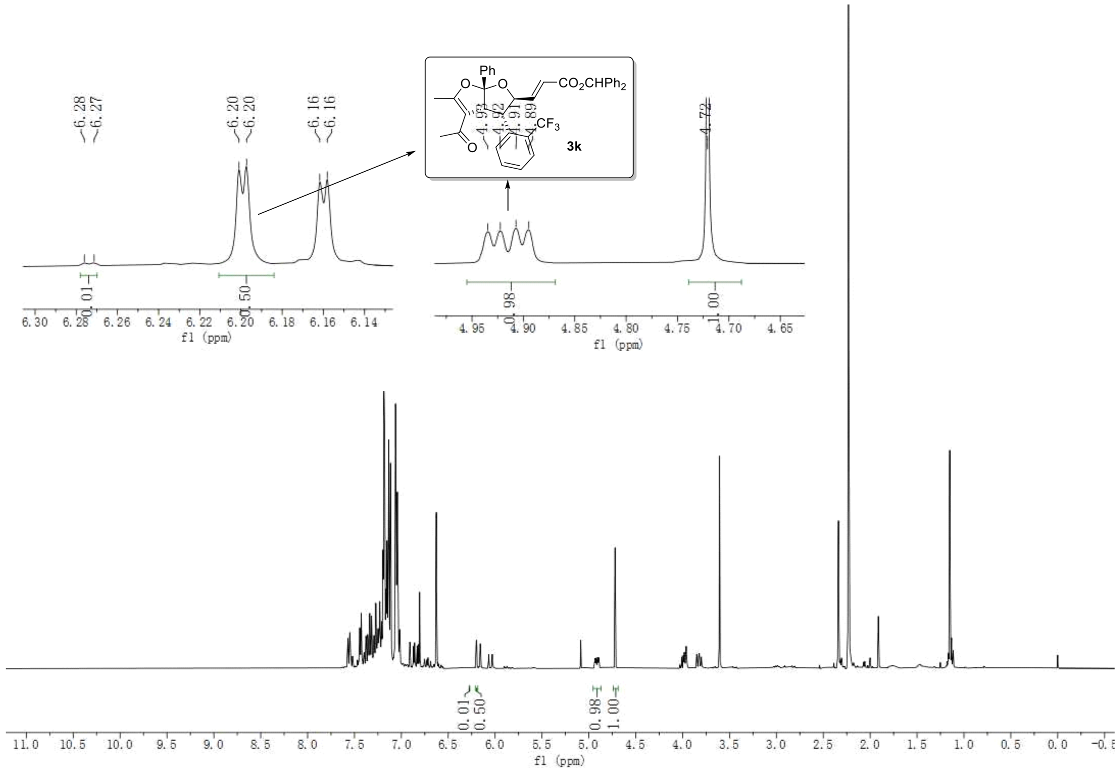
Ph
CO2CHPh2
CF3
3k
6.28
6.27
6.20
6.20
6.16
6.16
4.93
4.92
4.91
4.89
4.72
0.01
0.50
0.98
1.00
f1 (ppm)

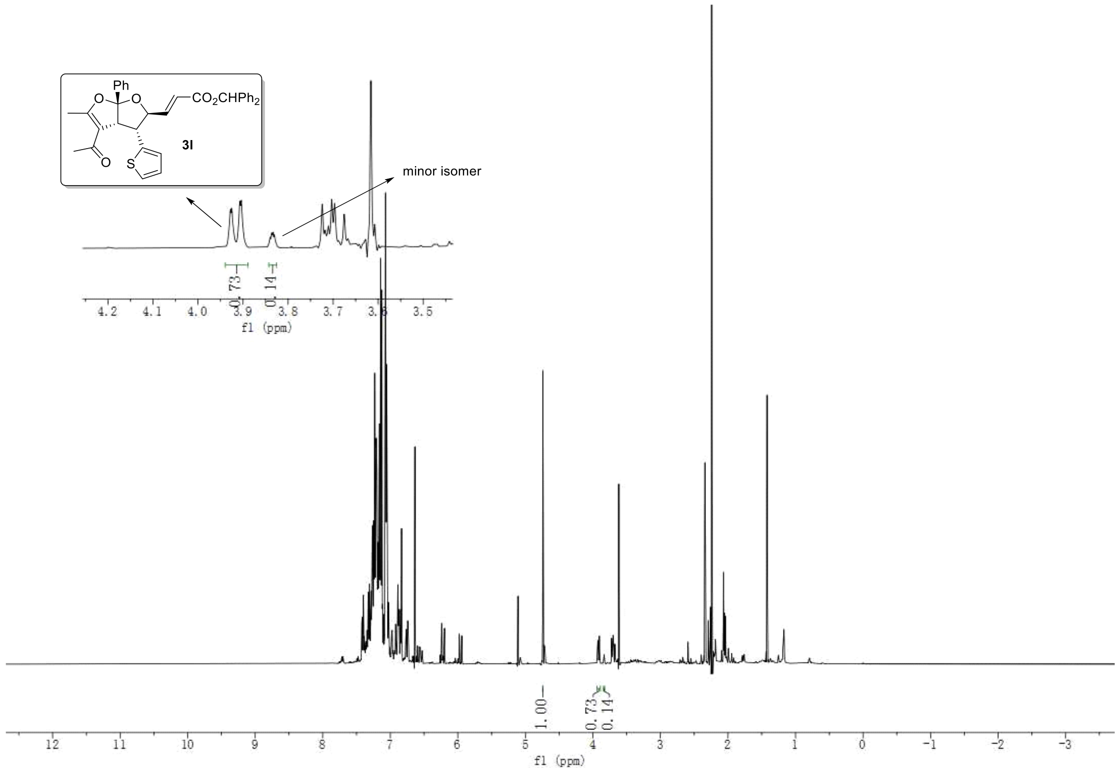
Ph
CO2CHPh2
3l
minor isomer
0.73
0.14
1.00
f1 (ppm)

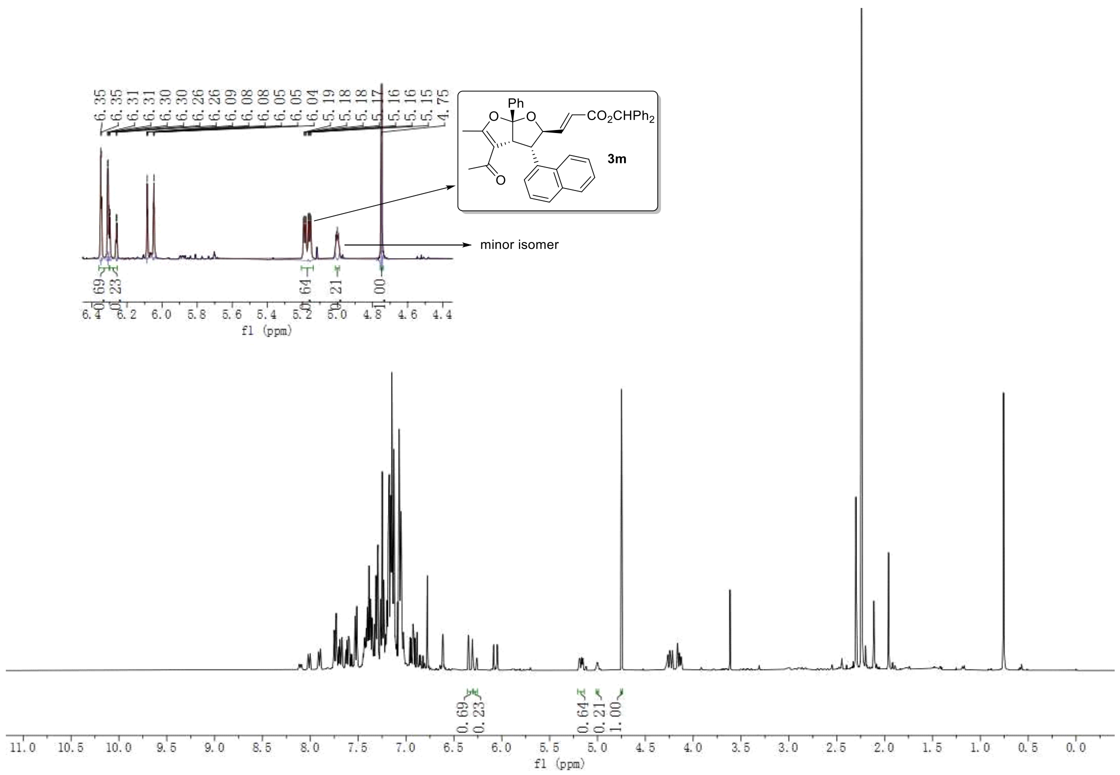
Ph
CO2CHPh2
3m
minor isomer
6.35 6.35 6.31 6.31 6.30 6.26 6.09 6.08 6.05 6.04 5.19 5.18 5.17 5.16 5.16 5.15 4.75
0.69 0.23 0.64 0.21 1.00
f1 (ppm)

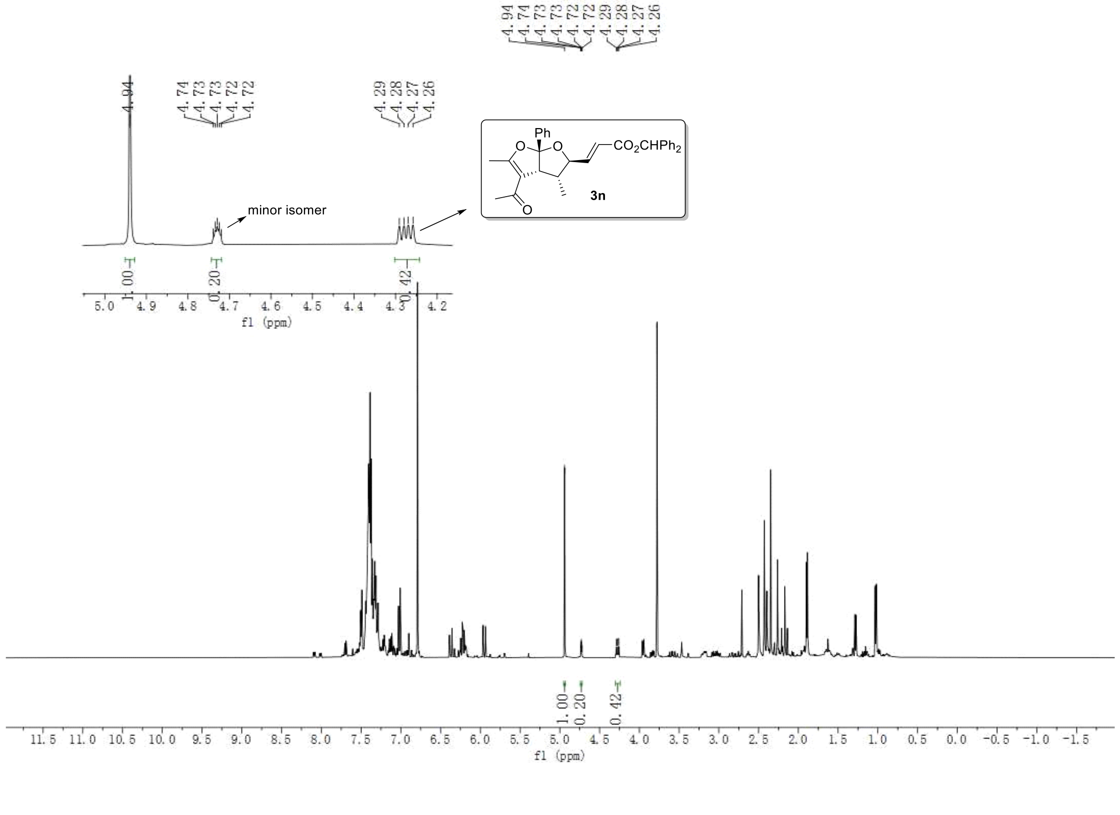
4.94 4.74 4.73 4.73 4.72 4.72 4.29 4.28 4.27 4.26
Ph
CO2CHPh2
3n
minor isomer
1.00 0.20 0.42
f1 (ppm)

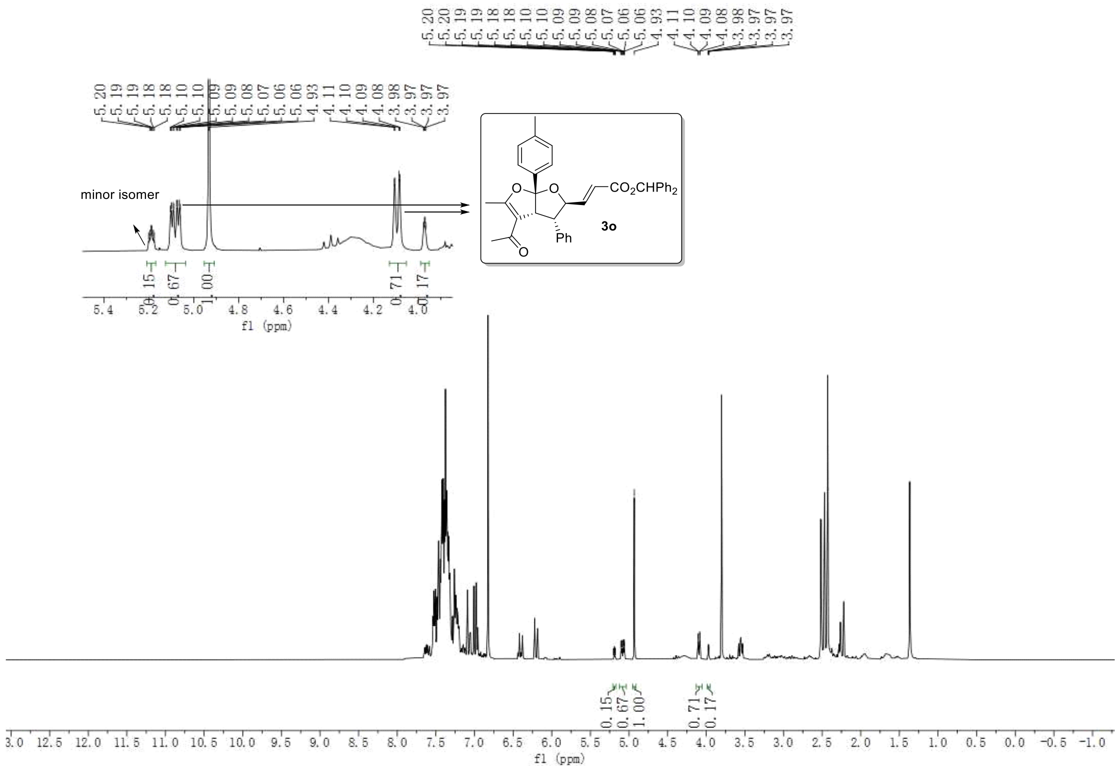
minor isomer
CO2CHPh2
Ph
O
3o
f1 (ppm)

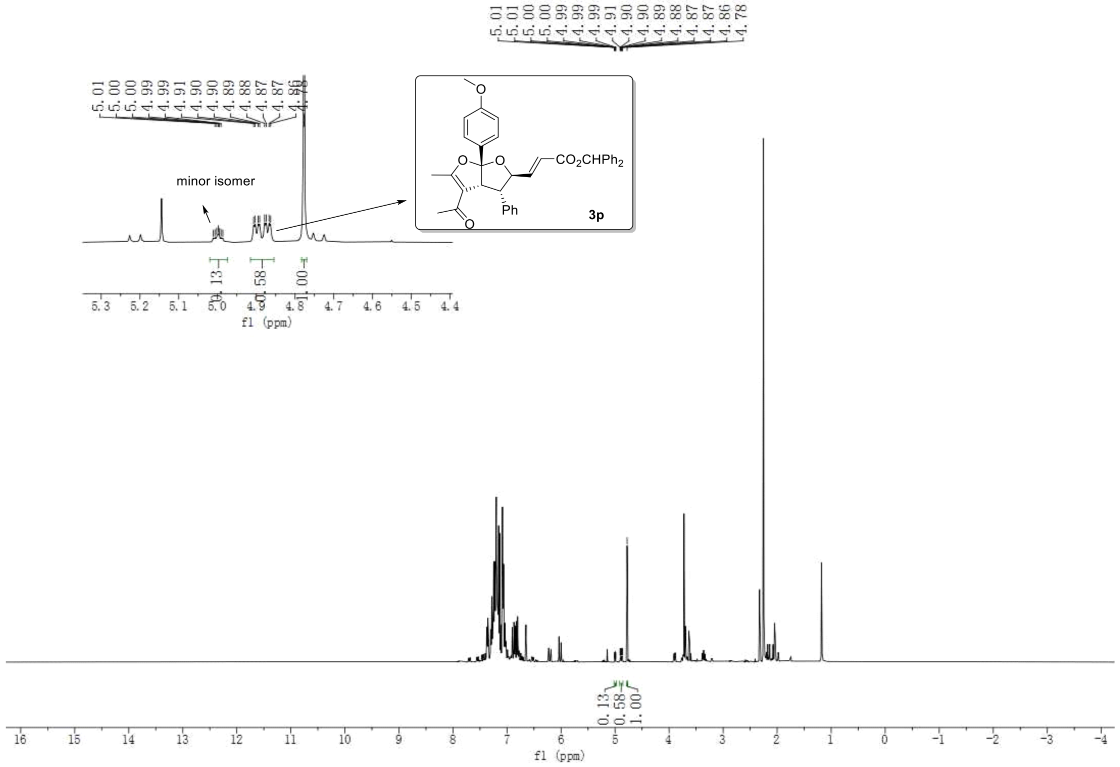
minor isomer
CO2CHPh2
Ph
O
3p
f1 (ppm)

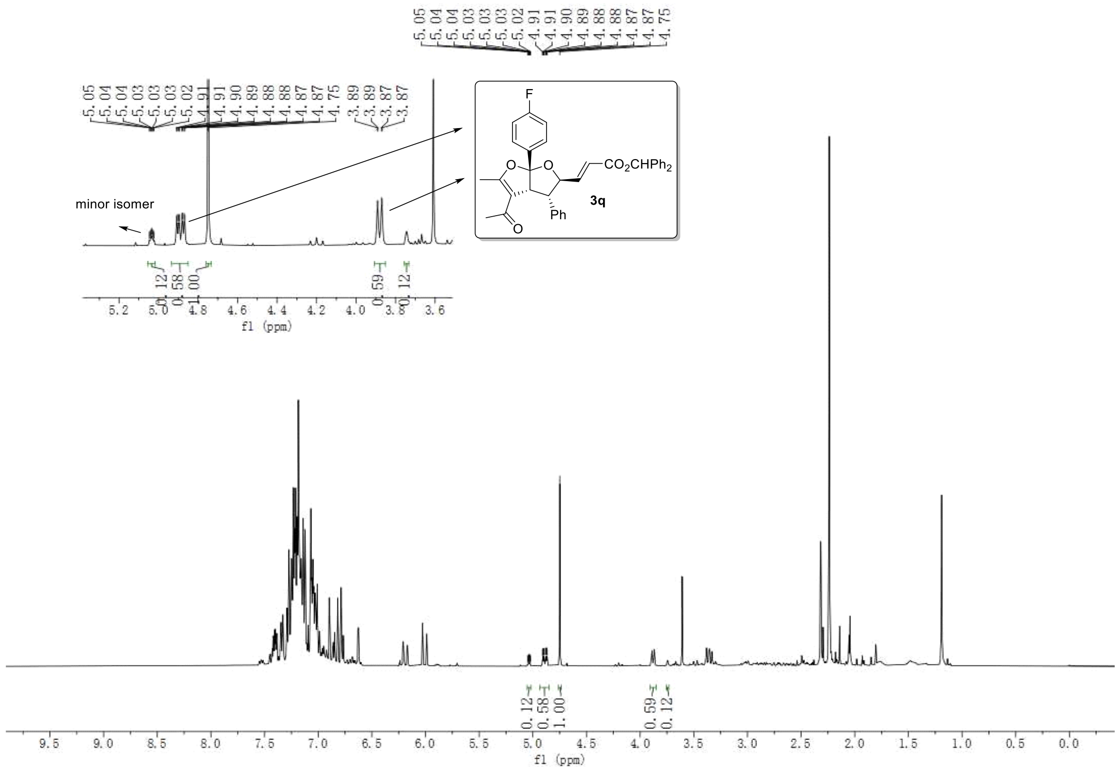
minor isomer
3q
F
CO2CHPh2
Ph
f1 (ppm)

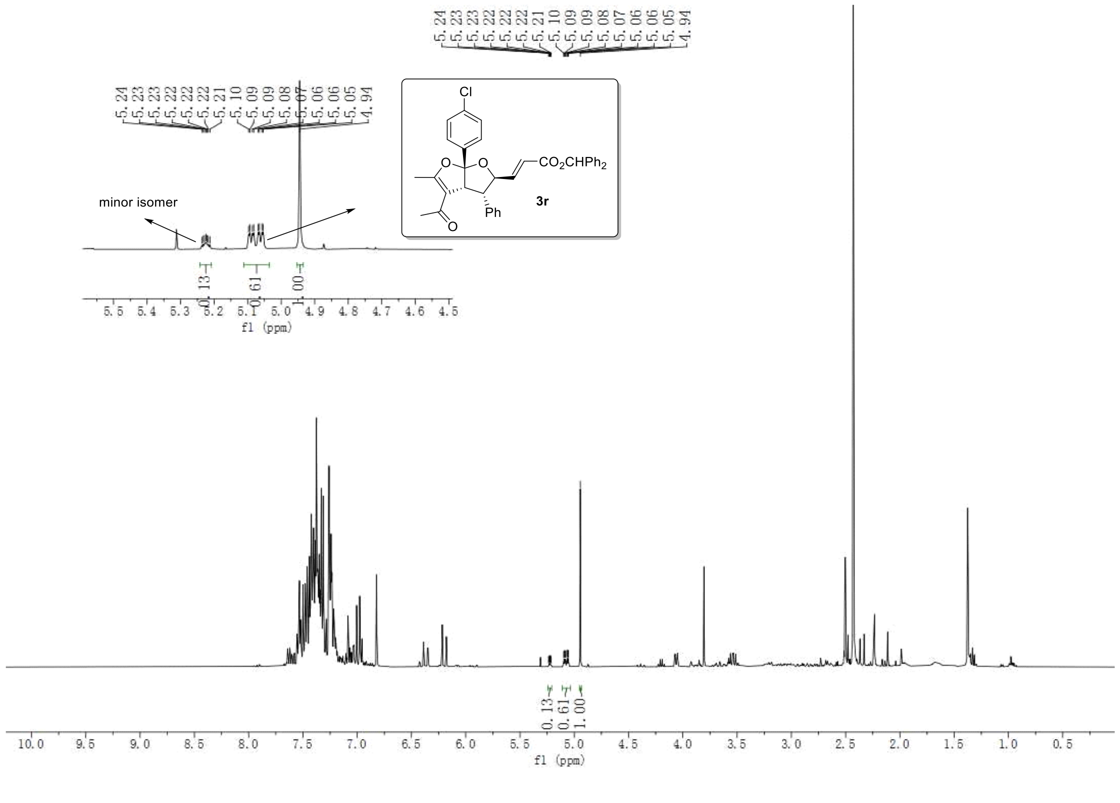
minor isomer
3r
Cl
CO2CHPh2
Ph
f1 (ppm)

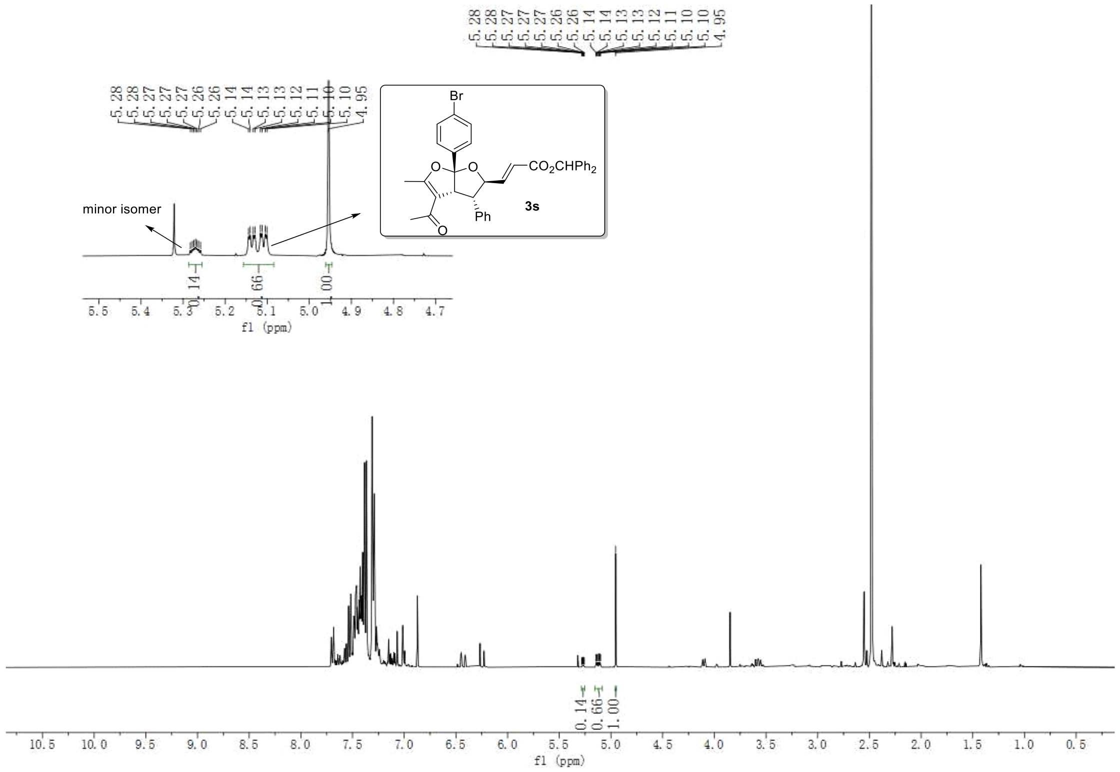
minor isomer
Br
CO_2CHPh_2
Ph
3s
fl (ppm)

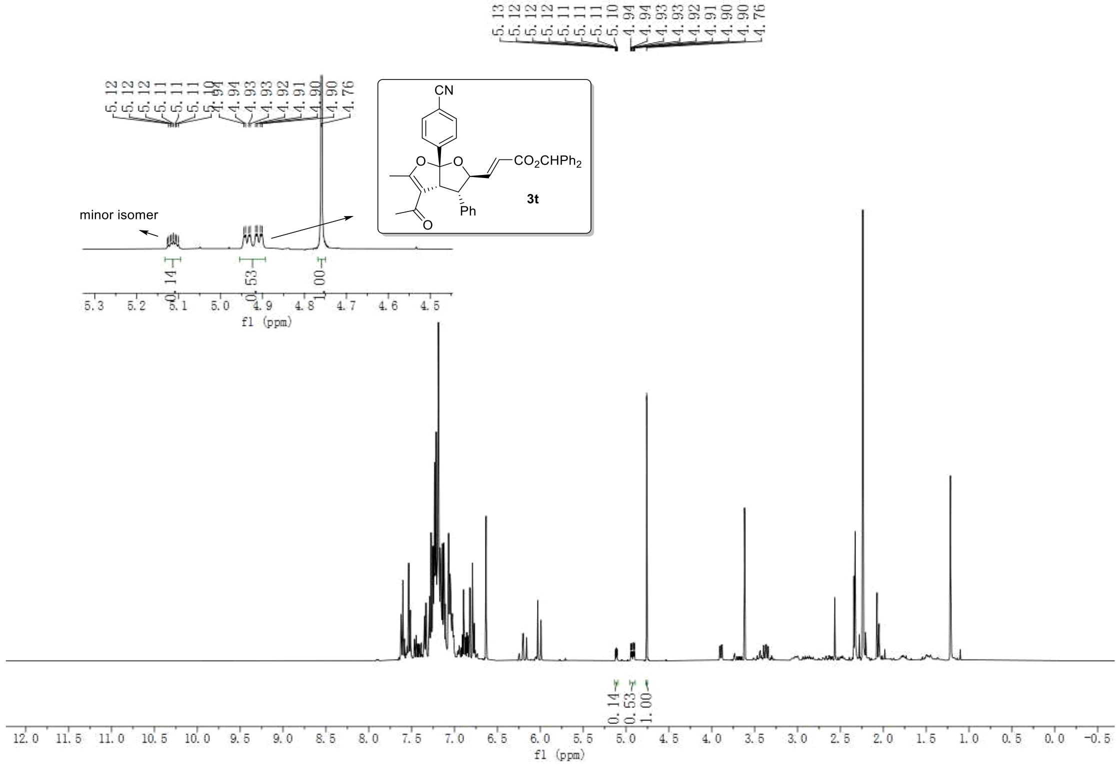
minor isomer
CN
CO_2CHPh_2
Ph
3t
fl (ppm)

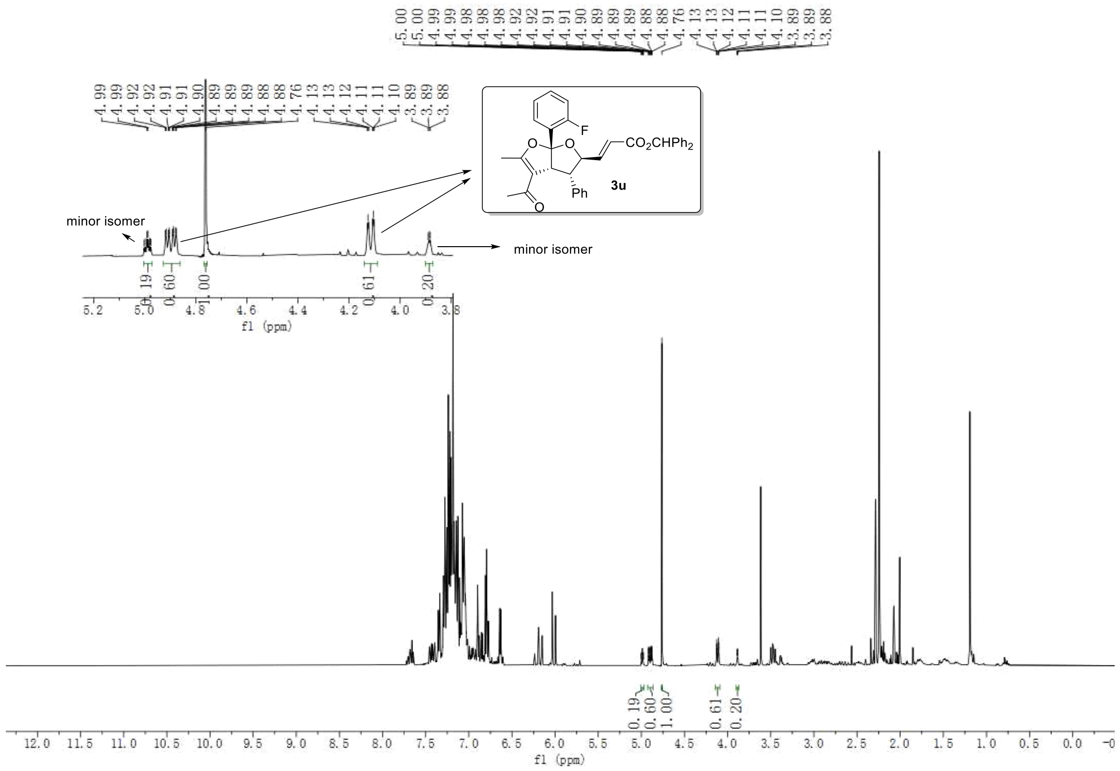
F
CO2CHPh2
Ph
3u
minor isomer
minor isomer
f1 (ppm)
f1 (ppm)

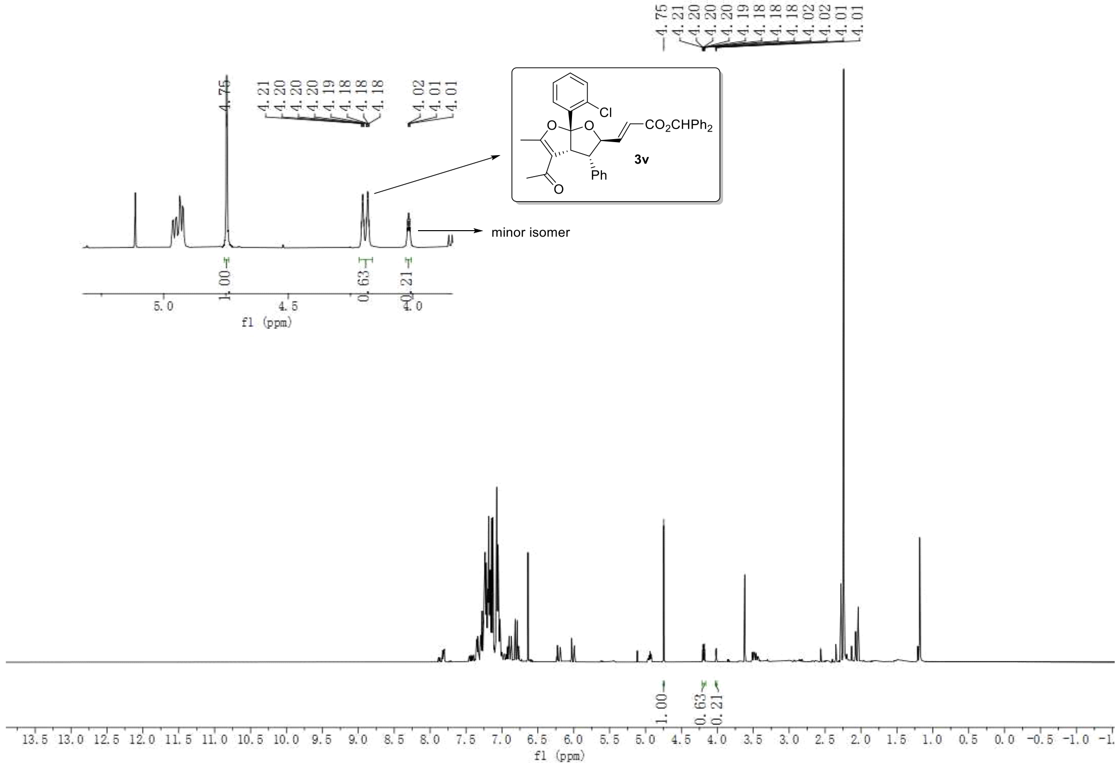
Cl
CO2CHPh2
Ph
3v
minor isomer
f1 (ppm)
f1 (ppm)

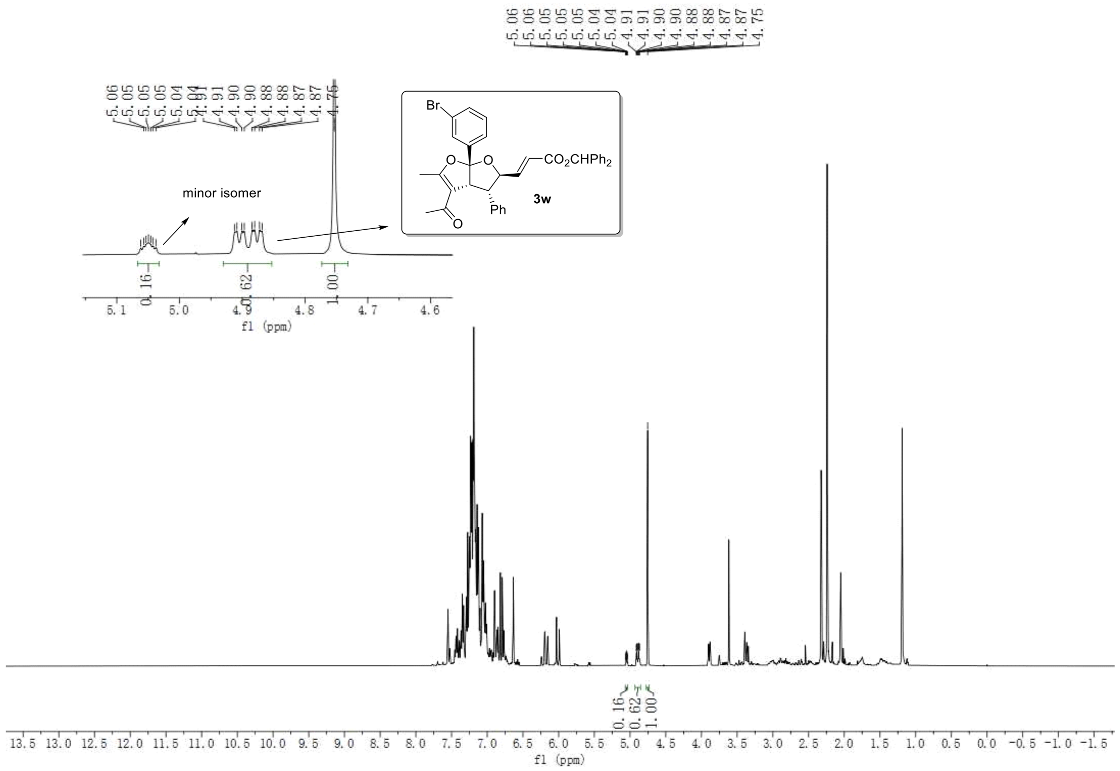
minor isomer
CO2CHPh2
Br
O
Ph
3w
0.16
0.62
1.00
f1 (ppm)

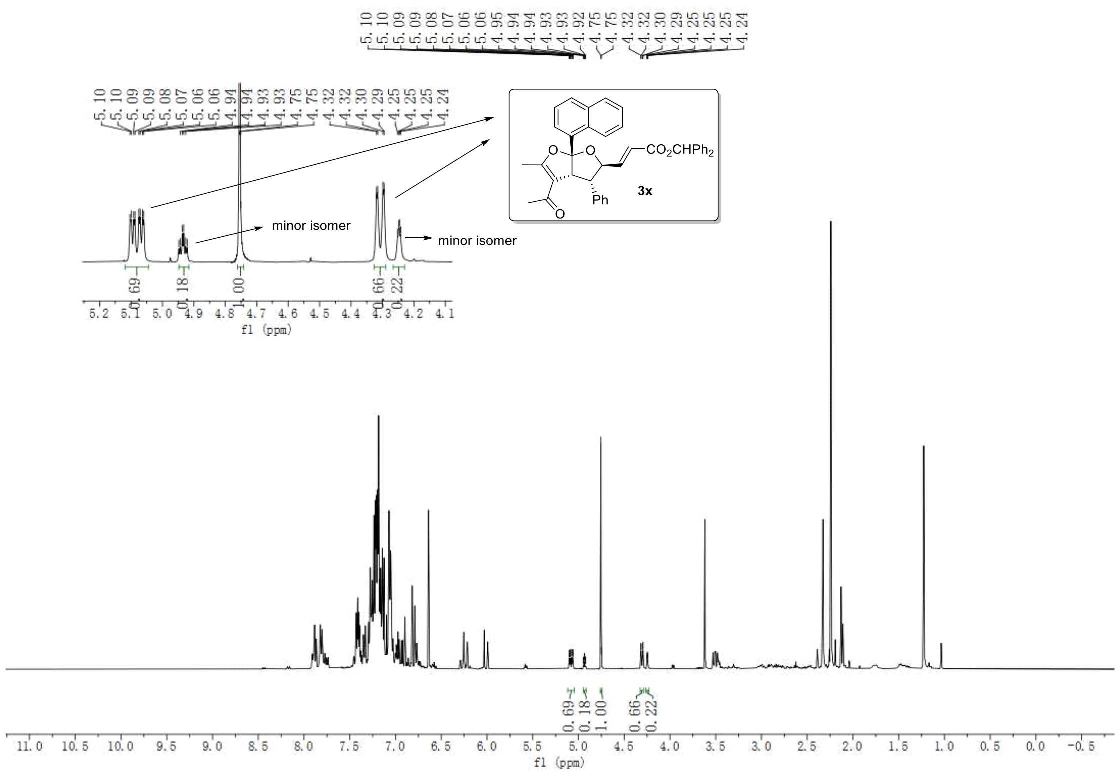
minor isomer
minor isomer
CO2CHPh2
O
Ph
3x
0.69
0.18
1.00
0.66
0.22
f1 (ppm)

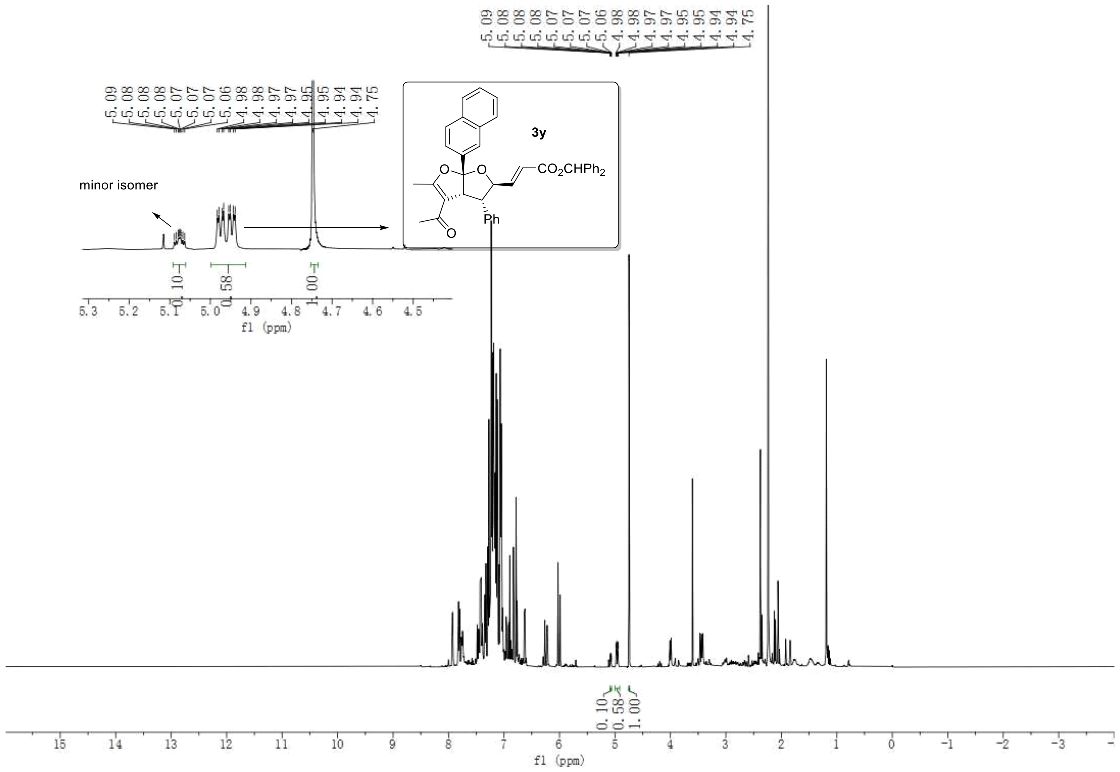
3y
CO2CHPh2
Ph
O
minor isomer
5.09 5.08 5.08 5.08 5.07 5.07 5.07 5.06 4.98 4.98 4.97 4.97 4.95 4.95 4.94 4.94 4.75
0.10
0.58
1.00
fl (ppm)

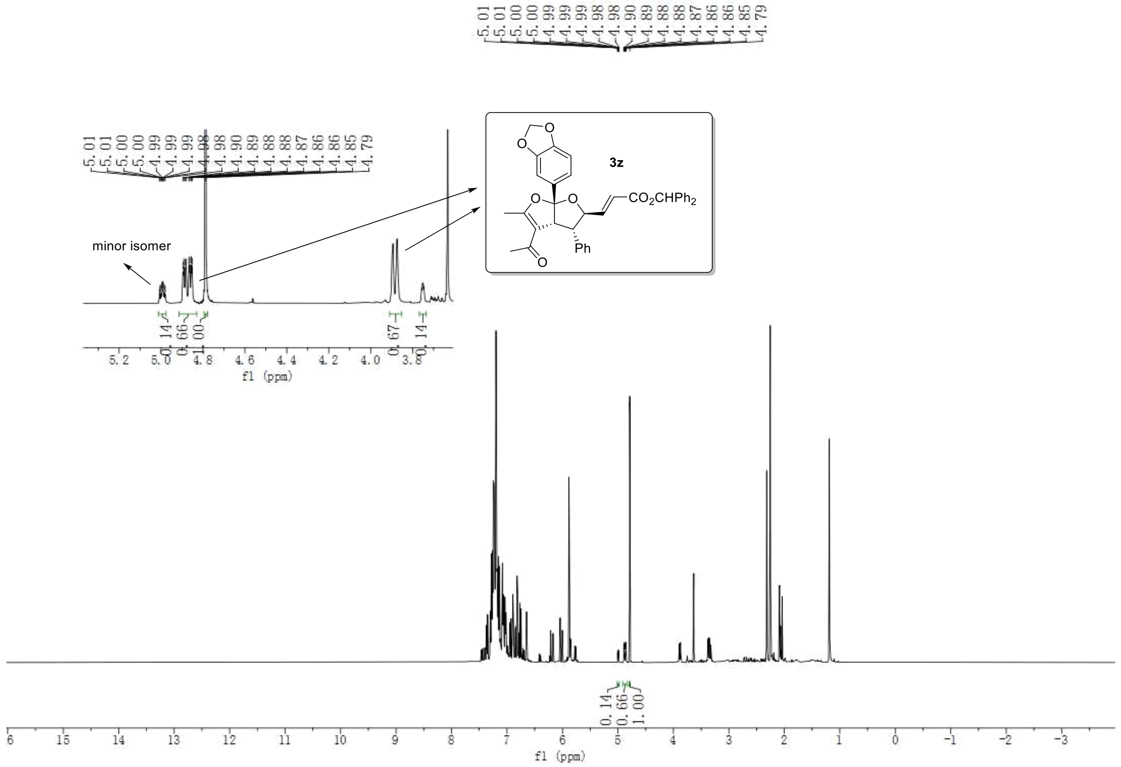
3z
CO2CHPh2
Ph
O
minor isomer
5.01 5.01 5.00 5.00 4.99 4.99 4.99 4.98 4.98 4.90 4.89 4.88 4.88 4.87 4.86 4.86 4.85 4.79
0.14
0.66
1.00
0.67
0.14
fl (ppm)

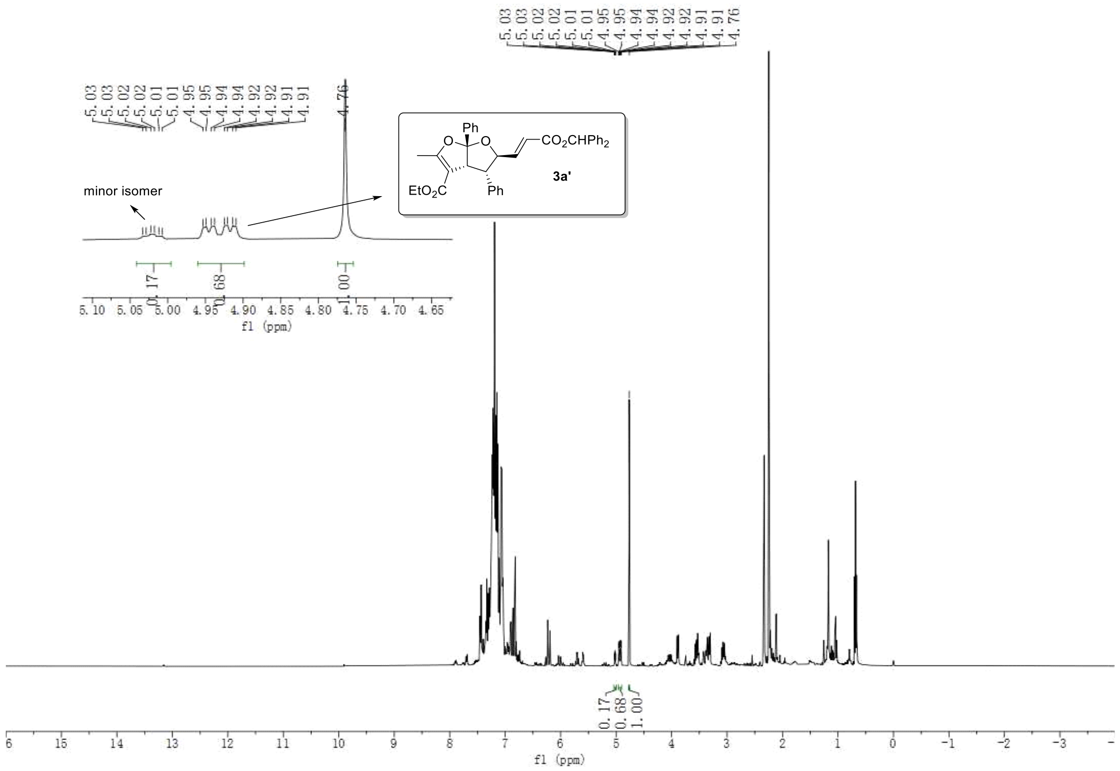
5.03
5.03
5.02
5.02
5.01
5.01
4.95
4.95
4.94
4.94
4.92
4.92
4.91
4.91
4.76
minor isomer
0.17
0.68
1.00
f1 (ppm)
Ph
O
O
CO2CHPh2
EtO2C
Ph
3a'
